# PPP1R81 correlates with the survival and cell proliferation in lower-grade glioma

**DOI:** 10.1042/BSR20230028

**Published:** 2023-05-05

**Authors:** Feng Xiao, Xinfang Jie, Xiang Zhou, Yun Guo, Gu Feng Sun, Li Lin, Guo Wen Hu, Kai Huang, Hua Guo

**Affiliations:** 1 Department of Neurosurgery, The Second Affiliated Hospital of Nanchang University, Nanchang, China; 2 Jiangxi Key Laboratory of Neurological Tumors and Cerebrovascular Diseases, Nanchang, China; 3 Jiangxi Health Commission Key Laboratory of Neurological Medicine, Nanchang, China; 4 Institute of Neuroscience, Nanchang University, Nanchang, China; 5 Department of Neurosurgery, Fuzhou First People's Hospital, Fuzhou, Jiangxi, China

**Keywords:** cell proliferation, immune infiltration, lower-grade glioma, PPP1R81, prognostic signature

## Abstract

**Background:** The specific functions of PPP1R81 has been elucidated in multiple cancers; however, its role in lower-grade glioma (LGG) remains unknown. In this research, we inspected the specific role of PPP1R81 in LGG.

**Methods:** We totally evaluated the expression pattern and prognostic role of PPP1R81 in multitudinous tumors. Subsequently, we systematically examined the connection between PPP1R81 expression and prognosis, clinical characteristics, biological functions, genetic variations, and immunological characteristics in LGG according to the Cancer Genome Atlas (TCGA) and Chinese Glioma Genome Altas (CGGA) databases. *In vitro* experiments were executed to inspect the expression level and specific roles of PPP1R81 in LGG.

**Results:** PPP1R81 was elevated in multiple tumors and was tightly linked to a poor prognosis. LGG with higher expression of PPP1R81 showed poorer prognosis compared with lower expression of PPP1R81. The results of univariate and multivariate Cox regression analyses confirmed that the expression of PPP1R81 was an independent prognostic biomarker of LGG. Immune cell infiltration, immune checkpoint genes (ICPGs), copy number alterations (CNA), and tumor mutation burden (TMB) were also closely associated with PPP1R81 expression in LGG. *In vitro* experiments demonstrated that PPP1R81 was up-regulated and closely interrelated with cell proliferation and cell cycle in LGG.

**Conclusion:** PPP1R81 was an independent prognostic signature and underlying therapeutic target for patients with LGG.

## Introduction

Gliomas, the most common brain tumor, are grouped into grade I to IV by the World Health Organization (WHO) in the light of the corresponding standard clinical features [1]. Currently, WHO grade II and III gliomas, termed LGGs and WHO grade IV gliomas (glioblastoma, GBM) remain resistant to traditional treatments [2,3]. Although LGG achieve a better survival rate than GBM, some patients tend to progress to GBM within months. Survival time in patients with LGG is strongly associated with therapeutic sensitivity [4]. Thence, it is critical to explore a novel prognostic biomarker to develop targeted LGG therapy.

PPP1R81 is also known as the cell division cycle associated protein 2. PPP1R81 can bind protein phosphatase 1 γ (PP1γ) and then regulate H3 phosphorylation [5]. A plenty of research has verified that PPP1R81 plays a significant part in cell cycle [6,7]. Numerous studies have also observed that PPP1R81 is elevated and closely associated with a worse prognosis in various cancers, including prostate [8], colorectal [9], breast [10], and liver [11] cancers. However, the association between PPP1R81 expression, prognosis, and immunological characteristics of LGG is still unknown. Thus, we conducted this study to detect whether PPP1R81 was associated with the survival and immunological characteristics of LGG.

First, we implemented a pan-cancer analysis of PPP1R81 in 33 tumors and found that the expression of PPP1R81 was significantly elevated in LGG. Afterwards, we inspected the prognostic role of PPP1R81 in LGG by bioinformatics analysis in an independent cohort of TCGA (*n*=477) and CGGA (*n*=419). According to the median expression of PPP1R81, it is grouped into high-PPP1R81 and low-PPP1R81 subtypes. The high-PPP1R81 subgroup exhibited worse prognosis than the low-PPP1R81 subgroup in both TCGA and CGGA cohorts. We implemented univariate and multivariate Cox regression analyses and detected that PPP1R81 was an independent prognostic biomarker of LGG. The nomogram model we created was able to accurately speculate the overall survival (OS) of LGG patients. On the grounds of the differentially expressed genes (DEGs), functional enrichment analyzes were performed to inspect the functional mechanism of PPP1R81 in LGG. Subsequently, Gene set variation analysis (GSVA) was also employed to examine the potential molecular pathways regulated by PPP1R81 in LGG. Additionally, the single sample GSEA algorithm (ssGSEA) was employed to inspect the connection between PPP1R81 expression and the enrichment of the 13 immune-related signatures. The relationship between immunological characteristics, including immune and stromal scores, immune infiltrating cells, and ICPGs expression, CNA burden, TMB, and PPP1R81 expression was also examined. The results illustrated that PPP1R81 expression was tightly interrelated with the immune infiltration in LGG. Eventually, we verified that PPP1R81 was elevated and vital for cell proliferation and cell cycle in LGG by performing *in vitro* experiments. In summary, combined with the above comprehensive analysis, we hypothesized that PPP1R81 was an independent prognostic biomarker and was expected to be an underlying therapeutic target of patients with LGG.

## Methods

**Figure 1:**
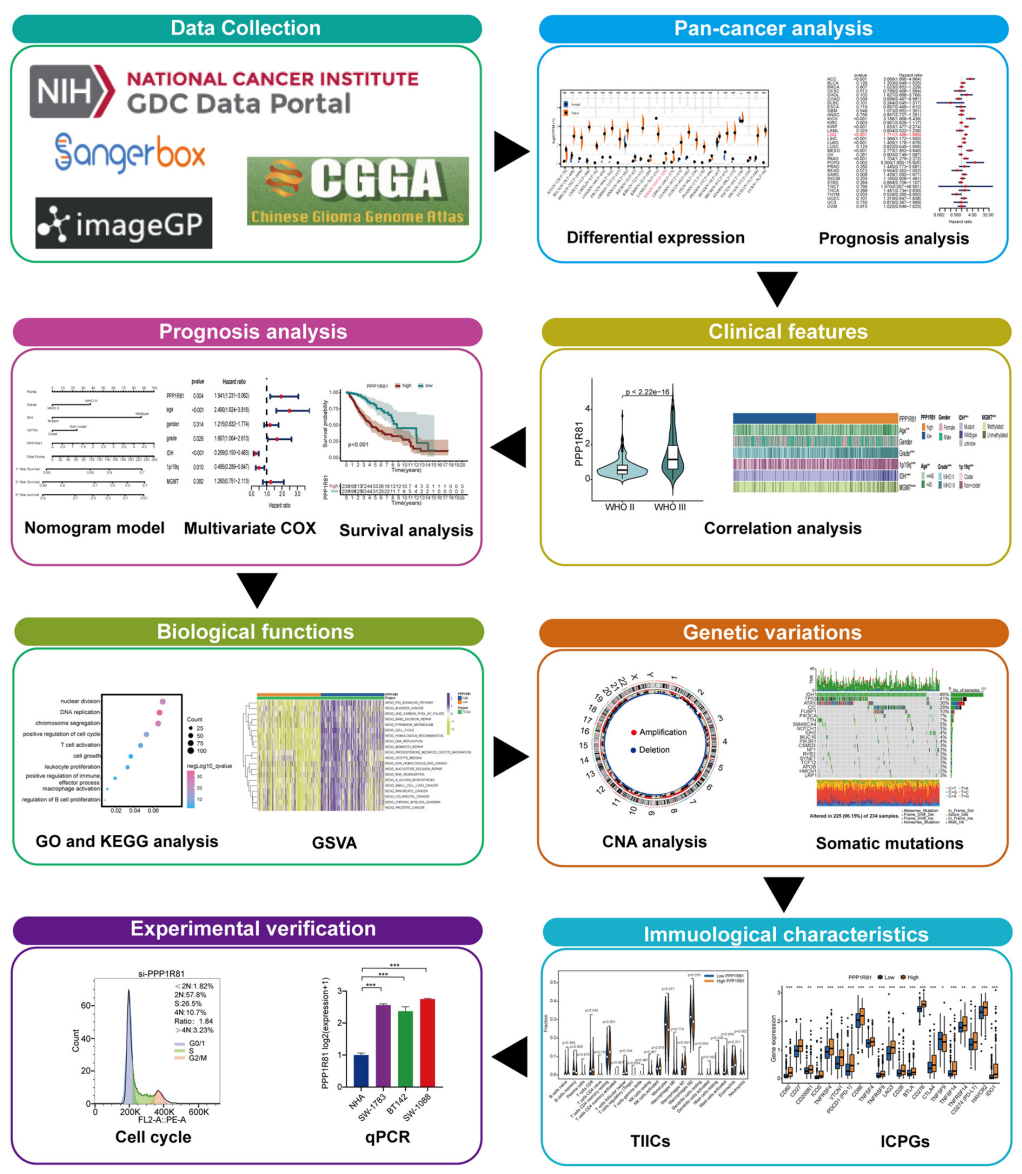
Flowchart showing the processes used in this study

The detailed flow chart of this research process is exhibited in Figure 1.

### Data gathering and processing

Gene expression, clinical information, and TMB data applied to pan-cancer analysis were attained from TCGA database. Furthermore, PPP1R81 expression data in normal tissue were acquired from Genotype-Tissue Expression (GTEx) datasets. Differential expression of PPP1R81 between TCGA and GTEx datasets was examined using Sangerbox website tools (http://sangerbox.com/).

In the present study, two independent LGG datasets were explored, including TCGA and CGGA datasets (CGGA_693). The gene expression and relevant clinical data of the LGG samples were acquired from TCGA and CGGA websites, respectively. The downloaded gene expression data were in Fragments Per Kilobase of transcript per Million (FPKM) format. It was not easy to compare because of the inconsistency of FPKM values. Therefore, we transformed the FPKM values of the two LGG datasets to Transcripts Per Kilobase Million (TPM) values by adopting the same algorithm utilized in previous studies [12,13]. Then, we transformed the TPM values by log2 to ensure easier comparison. The genomic mutation data in LGG were obtained from TCGA database.

### Patient's inclusion criteria

LGG patients in the two independent cohorts were included in the present study according to the following criteria: (1) patients with grade WHO II or III classification, (2) LGG patients with OS time >1 month, and (3) LGG patients with mRNA expression data. Based on these inclusion criteria, 477 LGG patients (Supplementary Table S1) and 419 LGG patients (Supplementary Table S2) were included in TCGA and CGGA datasets. However, to ensure consistency among 33 cancers, LGG patients with OS time <1 month were also adopted in the PPP1R81 pan-cancer analysis.

### Prognostic role of PPP1R81

LGG patients were grouped into low-PPP1R81 and high-PPP1R81 subgroups in line with the median expression of PPP1R81 in both TCGA and CGGA cohorts. To detect the predictive prognostic effect of PPP1R81 expression in the two datasets, we established receiver operating characteristic (ROC) curves and measured the area under the curve (AUC) values. The independent prognostic value of PPP1R81 expression was investigated by performing Cox regression analyses.

### Creation and verification of the nomogram model

A nomogram model was created by exploiting the R package ‘rms’ [14], in the light of common independent prognostic signatures (PPP1R81 expression, isocitrate dehydrogenase (IDH) mutation status, 1p/19q deletion, and WHO Grade) using TCGA and CGGA cohorts to forecast the OS of LGG patients. Calibration curves were implemented to examine the precision of four independent prognostic factors in forecasting the prognosis of LGG patients.

### Functional annotations and gene set variation analysis

We identified DEGs between low-PPP1R81 and high-PPP1R81 subtypes by conducting limma package [15] in both TCGA and CGGA cohorts (|log2 [fold change]>0.5 and the false discovery rate (FDR) < 0.05). In total, 1680 and 12,346 DEGs were selected in TCGA (Supplementary Table S3) and CGGA (Supplementary Table S4) cohorts, respectively. Gene Ontology (GO) and Kyoto Encyclopedia of Genes and Genomes (KEGG) analyses were executed by adopting the clusterProfiler package [16] in line with DEGs and the results were displayed using ImageGP website tools (http://www.ehbio.com/ImageGP/).

Additionally, we exploited GSVA to investigate the most enriched molecular pathways of the low-PPP1R81 and high-PPP1R81 subtypes by exploiting the limma package (|log2 [fold change]>0.5 and FDR< 0.05).

### Cell culture and lentiviruses transfection

We acquired three LGG cell lines, including SW1783, and BT142, and SW1088, from the American Type Culture Collection (ATCC). The normal human astrocyte (NHA) cell line was collected from Culture Collection of the Chinese Academy of Sciences (Shanghai, China). We cultured SW-1088 and SW-1783 cell lines with Leibovitz’s L-15 medium and 10% fetal bovine serum (Gibco). Additionally, BT142 and NHA cell lines were cultured with Dulbecco’s modified Eagle’s medium/F12 medium. The incubated conditions of these cell lines were 37°C and 5% CO_2_. We purchased lentiviruses expressing shRNA for PPP1R81 from Obio Technology (Shanghai, China). The target sequence of PPP1R81 shRNA was 5′-TGGGACTCATCCGAGCTTAAT-3′. We transfected the SW1088 cell lines with shRNA-PPP1R81 and negative control (NC) lentiviral vectors according to the protocol. The shRNA-PPP1R81 lentiviral vector generated the siRNA-PPP1R81 after entering the SW1088 cells. Then the siRNA-PPP1R81 participated in RNA interference and exerted the PPP1R81 gene silencing effect. The multiplicities of infection (MOIs) were 10 in SW1088 cells. Polybrene was utilized to promote transfection efficiency and puromycin was employed to screen out positive cells.

### Quantitative Real-Time PCR (qRT-PCR)

We dissociated total RNA from cells using the Simply P Total RNA Extraction Kit (Bioflux China), and then reverse transcribed to cDNA with HiScript III-RT SuperMix (Vazyme, China). Afterwards, qRT-PCR analysis was conducted by utilizing ChamQ Universal SYBR qPCR Master Mix (Vazyme, China). The results were processed by exploiting the 2^−∆∆CT^ method. The genes primer sequences were as follows: forward PPP1R81 primer, 5′-TGATGTCAGGTCACCAGCTACTC-3′; reverse PPP1R81 primer, 5′-GACACATCTTAACAGAGGGTTTCTT-3′; forward GAPDH primer, 5′-AACGGATTTGGTCGTATTGGG-3′, and reverse GAPDH primer, 5′-GGCAACAATATCCACTTTACCAGA-3′.

### CCK-8 assay

The transfected SW1088 cells were plated in 96-well plates at 2 × 10^3^/well and incubated. Next, we inspected the cell proliferation by Cell Counting Kit 8 assay (Glpbio, U.S.A., GK10001) in 0, 24, 48, 72, and 96 h. OD value was measured at 450 nm by enzyme labeling instrument.

### Colony formation assay

We seeded the transfected SW1088 cells in six-well plates at 2 × 10^3^/well and incubated for 14 days. Next, we stained the cells using 0.1% Crystal Violet stain solution and counted the number of colonies by ImageJ.

### EdU assay

The transfected SW1088 cells (2 × 10^4^) were plated in 24-well plates and cultured for 72 h. Afterwards, the cells were incubated with EdU reagent (BryoClick™ Edu-555, C0075S) for 2 h. The 4% paraformaldehyde and 0.3% Triton X-100 were utilized to fix the cells at room temperature for 15 min. Eventually, we stained cells using click reaction solution and the Hoechst 33342 staining at room temperature and away from light for 15 min. We quantified the EdU incorporation rate by ImageJ.

### Cell cycle analysis

We fixed transfected SW1088 cells in 70% ethanol and preserved at 4°C overnight. Afterwards, the cells were processed with RNase A containing propidium iodide (Suzhou, China) at room temperature for 30 min. We examined the cell cycle distribution by operating flow cytometry.

### CNA and somatic mutation analysis

The CNA and TMB data from LGG patients were acquired from the TCGA database. All deletions/amplifications in the entire genome were screened by GISTIC 2.0 [17]. Circos plots were used to display chromosome loss/gain alterations using the RCircos package [18]. The frequencies and types of gene mutations were investigated using the maftools package [19].

### Immunological characteristics of LGG

The ESTIMATE algorithm was implemented to assess the abundance of stromal cells (StromalScore), immune cells (ImmueScore), non-tumor compounds (ESTIMATEScore), and tumor purity [20]. CIBERSORT, a deconvolution algorithm, was executed to measure the composition of 22 types of tumor infiltrating immune cells (TIICs) in line with the median expression of PPP1R81 in LGG [21]. Furthermore, we adopted the ssGSEA algorithm to measure the abundance of 13 immune-related factors, obtained from former studies [22,23], between the two subgroups in both TCGA and CGGA cohorts. Additionally, we selected 25 ICPGs associated with potential treatment from previous studies [23,24] and identified their correlation with the expression of PPP1R81 in both TCGA and CGGA datasets.

### Statistics

The two-sided log-rank test and the Kaplan–Meier method were utilized to identify distinct prognosis between high-PPP1R81 and low-PPP1R81 expression subtypes. The ability of PPP1R81 expression to predict prognosis was further evaluated using ROC curves and AUC values. The independent prognostic role of the expression of PPP1R81 was examined by Cox regression analyses. Additionally, we executed the Student’s *t*-test to distinguish between distinct levels of immune-associated factors, including stromal score, immune score, tumor purity, CNA burden, TMB, 13 immune-associated signatures, and 25 ICPGs, between the high-PPP1R81 and low-PPP1R81 subtypes. We implemented Spearman’s or Pearson’s correlation test to examine the connection between distributed variables. We conducted all statistical analyses in the R language v4.1.0, and GraphPad Prism 8 (GraphPad Software, Inc.).

## Results

### Pan-Cancer analysis of PPP1R81

**Figure 2:**
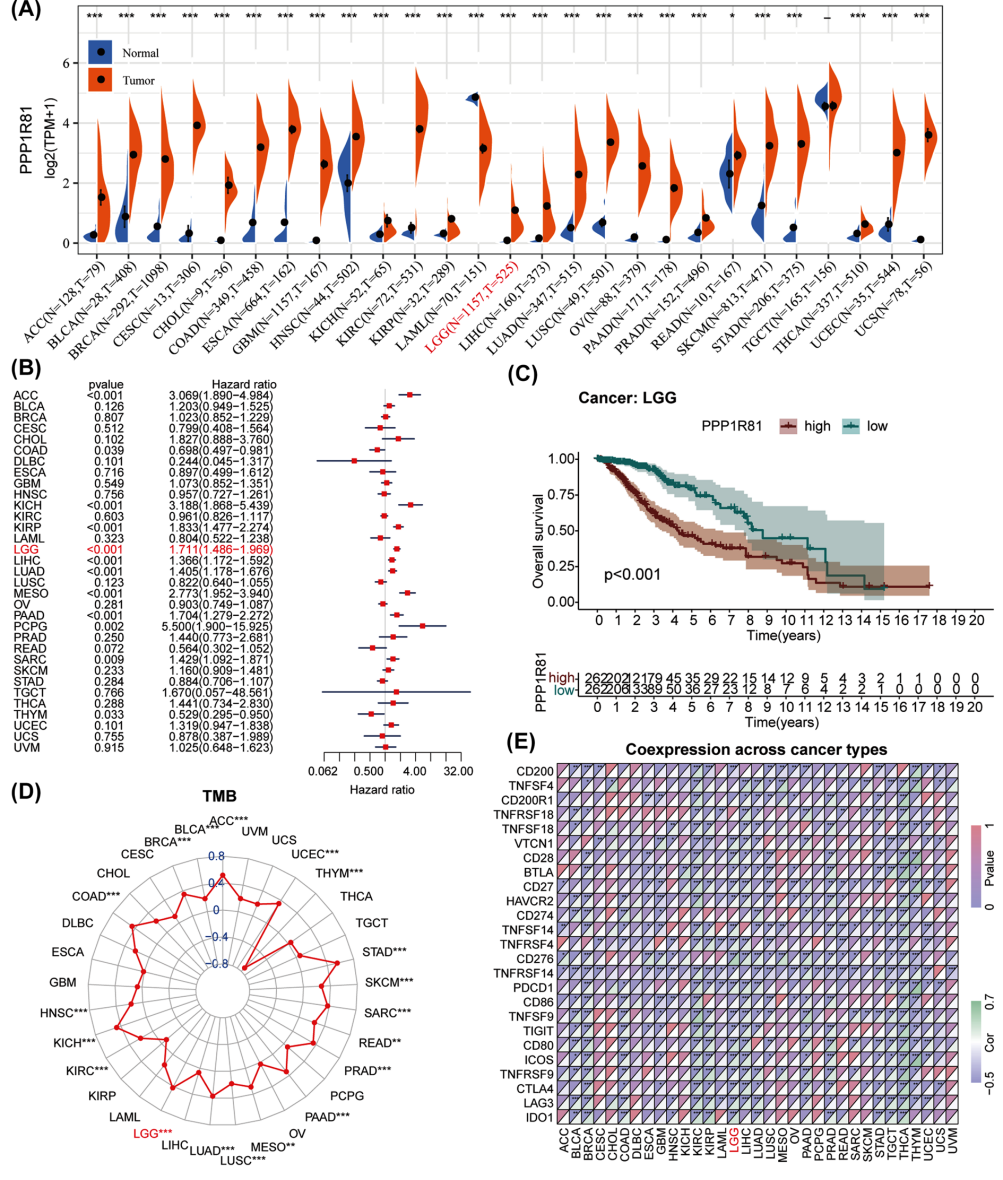
Pan-cancer analysis of PPP1R81 in 33 cancers (**A**) Distinct expression of PPP1R81 in various tumor tissues and relevant normal tissues. (**B**) Univariate Cox regression analysis of PPP1R81 expression in multiple tumors. (**C**) Kaplan–Meier analysis of PPP1R81 in pan-LGG. (**D**) Differential TMB in different cancers. (**E**) Co-expression of ICPGs in different cancers (*P<0.05, **P<0.01, ***P<0.001).

By estimating the results of pan-cancer analysis obtained from TCGA and GETx databases, we determined that PPP1R81 was abnormally expressed in multiple tumors. The comparison showed that PPP1R81 was significantly elevated in 24 cancers, including ACC, BLCA, BRCA, CESC, CHOL, COAD, ESCA, GBM, HNSC, KICH, KIRC, KIRP, LGG, LIHC, LUAD, OV, PAAD, PRAD, SKCM, STAD, TGCT, THCA, UCEC, and UCS, and slightly elevated in READ. However, the expression of PPP1R81 was depressed in LAML (Figure 2A).

We implemented a univariate Cox regression analysis to inspect the prognostic value of PPP1R81 expression across the 33 cancers. Interestingly, PPP1R81 expression was tightly interrelated with the prognosis of ACC, COAD, KICH, KIRP, LGG, LIHC, LUAD, MESO, PAAD, PCPG, SARC, and THYM (Figure 2B). Furthermore, a higher expression of PPP1R81 indicated a poorer prognosis in LGG (Figure 2C).

To evaluate genetic variation in the 33 cancer types, the connection between PPP1R81 expression and TMB was examined. The results confirmed that PPP1R81 expression was strongly linked to TMB in ACC, BLCA, BRCA, COAD, HNSC, KICH, KIRC, LGG, LUAD, LUSC, MESO, PAAD, PRAD, READ, SARC, SKCM, STAD, THYM, and UCEC (Figure 2D). Subsequently, we examined the co-expression of 25 ICPGs and PPP1R81 in the 33 cancer types and observed marked differences in expression in BLCA, BRCA, CESC, GBM, HNSC, KIRC, KIRP, LGG, LIHC, LUAD, LUSC, PAAD, PRAD, STAD, TGCT, THCA, THYM, and UCEC (Figure 2E).

Based on the above results, we investigated the clinicopathological features, prognosis, biological process, genetic variation, and immunological characteristics of PPP1R81 in LGG.

### Correlation between PPP1R81 and clinicopathological characteristics in LGG

**Figure 3:**
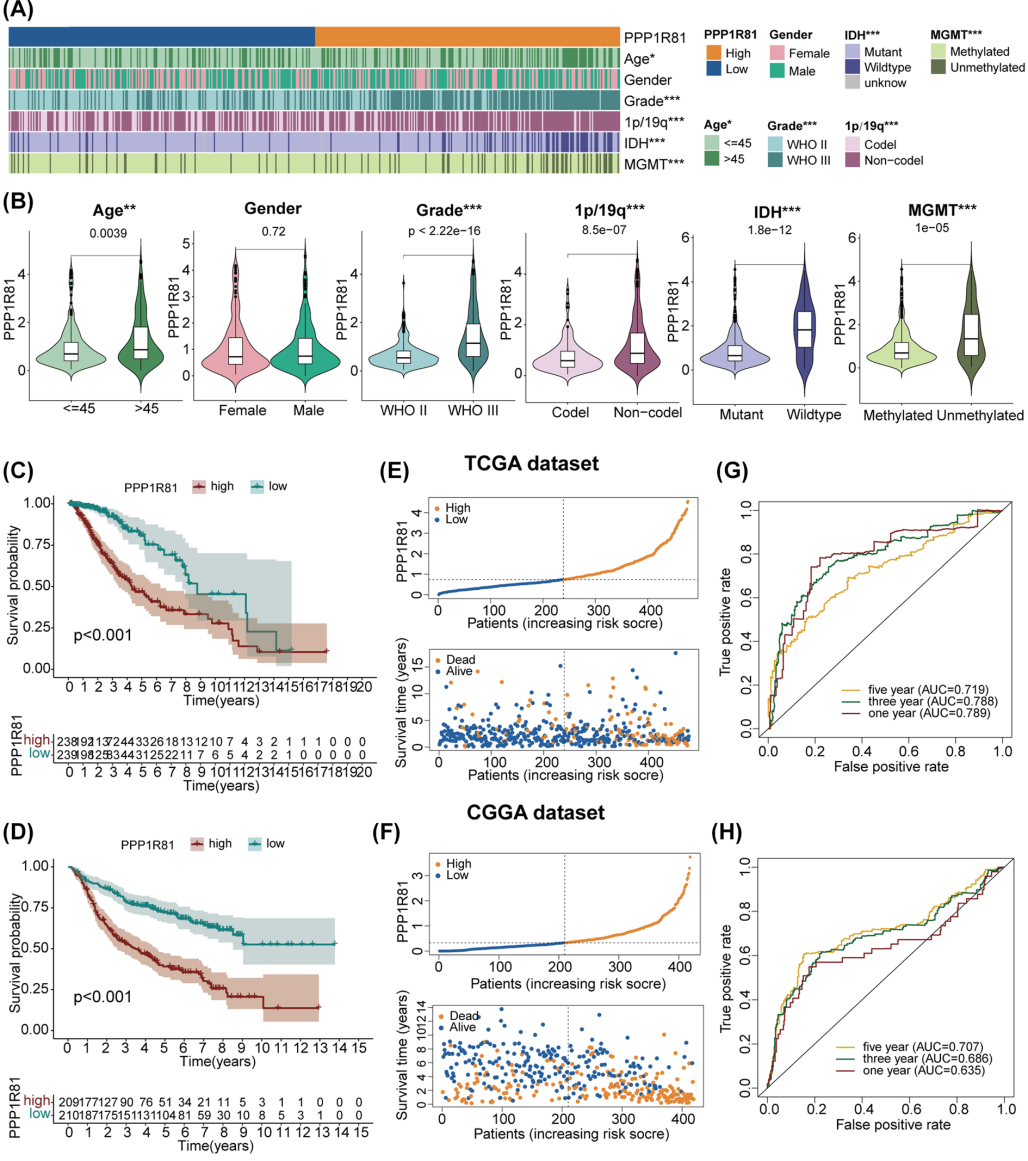
Clinical relevance of PPP1R81 in LGG patients (**A**) Connection between PPP1R81 expression and LGG clinical traits in TCGA cohort. (**B**) Analysis of the variance in PPP1R81 expression and clinical traits in TCGA dataset. Prognostic analysis of high-PPP1R81 and low-PPP1R81 subtypes in TCGA (**C**) and CGGA (**D**) cohorts. Distribution of the risk score, OS, and OS status of the high-PPP1R81 and low-PPP1R81 subtypes in TCGA (**E**) and CGGA (**F**) datasets. ROC curves reflecting the predictive capacity of the risk score in TCGA (**G**) and CGGA (**H**) cohorts (*P<0.05, **P<0.01, ***P<0.001).

The relationship between clinicopathological characteristics, including age, sex, WHO grade, IDH status, 1p/19q status and O6-methylguanine DNA methyltransferase (MGMT) methylation status, and differences in expression of PPP1R81 in LGG was explored. As shown in the heatmap (Figure 3A) and violin plots (Figure 3B), higher PPP1R81 expression was associated with older age, higher WHO grade level, IDH wildtype status, 1p/19q non-codel status, and MGMT promotor unmethylated status in TCGA dataset. Similarly, these results were also detected in CGGA dataset (Supplementary Figure S1).

### Higher expression of PPP1R81 predicted a poorer prognosis for LGG patients

We evaluated the OS between the low-PPP1R81 and high-PPP1R81 subtypes and determined that high-PPP1R81 subtype owned lower OS than in low-PPP1R81 subtype in TCGA (Figure 3C) and CGGA (Figure 3D) datasets. Subsequently, we investigated the distinct OS between the two subgroups, including WHO grade, IDH, and 1p/19q status in both TCGA and CGGA datasets. The results attested that OS was higher in low-PPP1R81 subtype than in high-PPP1R81 subtype, except for the WHO II grade cohort in TCGA dataset (Supplementary Figure S2A). Furthermore, we also examined the conjunction between PPP1R81 expression, OS status, and risk score in LGG. Up-regulated PPP1R81 correlated with a poorer OS status and higher risk score in TCGA (Figure 3E) and CGGA (Figure 3F) datasets. The ROC curves also confirmed the precision of PPP1R81 in predicting the prognosis of LGG patients. Furthermore, the AUC were 0.789/0.788/0.719, and 0.635/0.686/0.707 at 1/3/5 years in TCGA (Figure 3G) and CGGA (Figure 3H) datasets, respectively.

### Cox regression analysis and nomogram model

**Figure 4:**
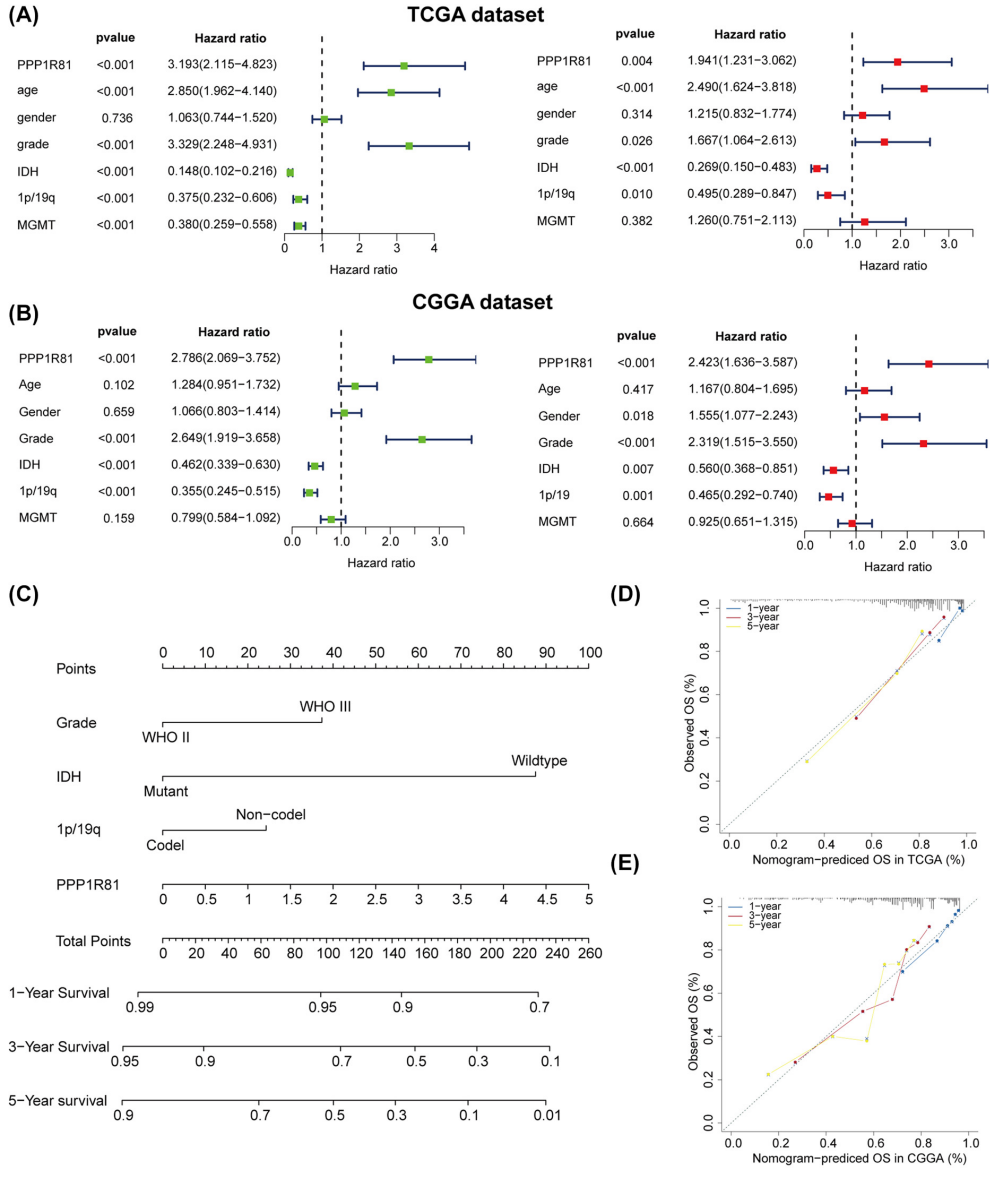
Cox regression analysis and nomogram model for LGG patients Univariate and multivariate Cox regression analysis of clinical traits and expression of PPP1R81 in TCGA (**A**) and CGGA (**B**) cohorts. Nomogram model created with WHO grade, IDH mutation, 1p/19q codel, and PPP1R81 expression in TCGA cohort (**C**). Calibration curves: confirming the accuracy of predicting 1/3/5-year OS in TCGA (**D**) and CGGA (**E**) datasets.

We implemented a univariate and multivariate Cox analysis to inspect the prognostic value of PPP1R81 expression in LGG. The results revealed that PPP1R81 expression, age, WHO grade, IDH, and 1p/19q were independent prognostic signatures for TCGA cohort (Figure 4A). Similarly, the results were also detected in the CGGA cohort (Figure 4B).

To further assess the ability of PPP1R81 in forecasting the prognosis of LGG patients, a nomogram model was conducted in line with WHO grade, PPP1R81 expression, IDH mutation, and 1p/19q codel, which were common independent prognostic factors in the two cohorts. WHO grade, PPP1R81 expression, IDH mutation, and 1p/19q codel were scored accordingly, and the total score was applied to forecast the OS of LGG patients (Figure 4C). Furthermore, calibration curves were used to affirm the precision of this model in forecasting the OS of LGG patients (Figure 4D,E). These results confirmed that the nomogram model could be exploited to accurately forecast the OS of LGG patients.

### Biological functions of PPP1R81 in LGG

**Figure 5:**
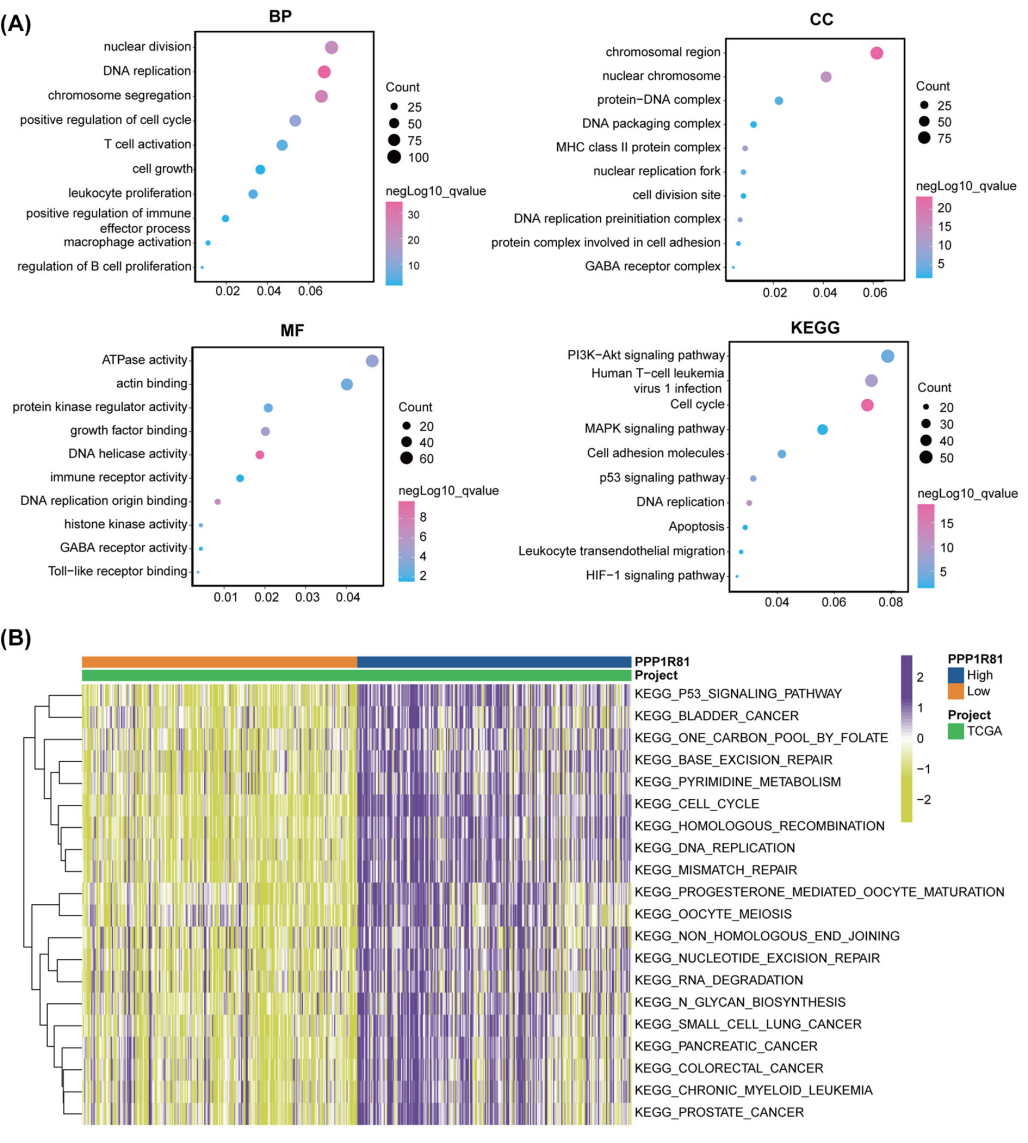
Biological functions of PPP1R81 in LGG in TCGA database (**A**) Functional enrichment analyses for PPP1R81 expression in patients with LGG. (**B**) GSVA for PPP1R81 in LGG patients.

To define the biological functions of PPP1R81 in LGG, we first identified DEGs by performing a differential expression analysis between the high-PPP1R81 and low-PPP1R81 subtypes. A total of 1680 and 12,346 DEGs were acquired and subjected to GO and KEGG analysis in TCGA and CGGA cohorts, respectively. The results affirmed that PPP1R81 was apparently correlated with biological processes (BP), including DNA replication, cell growth, and T-cell activation; cellular components (CCs), including nuclear replication fork, nuclear chromosome, and cell division; molecular functions (MF), including ATPase activity, protein kinase regulator activity, and growth factor binding; KEGG pathways, including the PI3K-Akt signaling pathway, cell cycle, and p53 signaling pathways in both TCGA (Figure 5A) and CGGA (Supplementary Figure S3A) datasets.

To further investigate the potential mechanisms associated with PPP1R81 overexpression in LGG, we employed GSVA analysis in TCGA and CGGA datasets. The results denominated that high PPP1R81 expression was tightly interrelated with hyperactivated pathways, including DNA replication, cell cycle activation, and p53 signaling pathways in both TCGA (Figure 5B) and CGGA (Supplementary Figure S3B) datasets.

### PPP1R81 correlated with genetic variations

**Figure 6:**
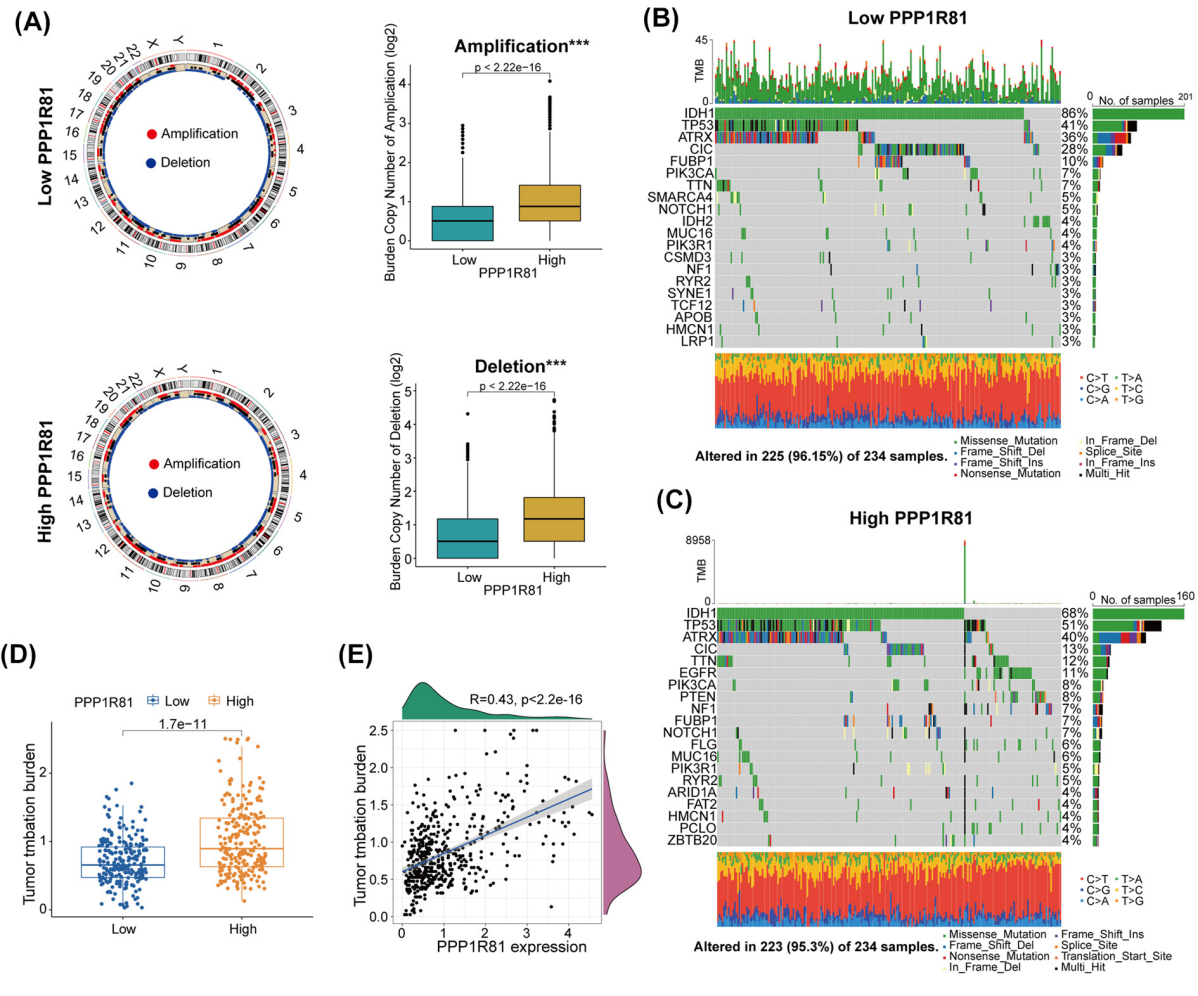
Comparisons of somatic variations between low-PPP1R81 and high-PPP1R81 expression subtypes in TCGA (**A**) Circos plots of the low-PPP1R81 and high-PPP1R81 subtypes revealed chromosome amplifications and deletions and boxplots exhibited greater burdens of copy number amplifications and deletions in the high-PPP1R81 expression subtype. The waterfall plots showing the mutated genes in the low-PPP1R81 subtype (**B**) and the high-PPP1R81 subtype (**C**). (**D,E**) TMB levels were positively linked to the expression of PPP1R81 (*P<0.05, **P<0.01, ***P<0.001).

Former research has explored the underlying role of genetic variations in regulating tumor immunity [25–27]. Therefore, we explored different genetic variations by performing CNA and somatic mutations analysis in the two subgroups. The copy number burden, gene amplifications, and deletions in high-PPP1R81 subgroup were distinctly higher than in low-PPP1R81 subgroup (Figure 6A). The somatic mutation analysis demonstrated that the genes *TP53*, *ATRX* and *NOTCH1* in the high-PPP1R81 subgroup had mutations of higher frequency than in the low-PPP1R81 subgroup, while the mutations *IDH1*, *CIC*, and *FUBP1* mutations in low-PPP1R81 subgroup were more frequent than in the high-PPP1R81 subgroup (Figure 6B,C). Besides, we ascertained that PPP1R81 expression was positively interrelated with the TMB level (Figure 6D,E).

### Correlation between PPP1R81 and immunological characteristics in LGG

**Figure 7:**
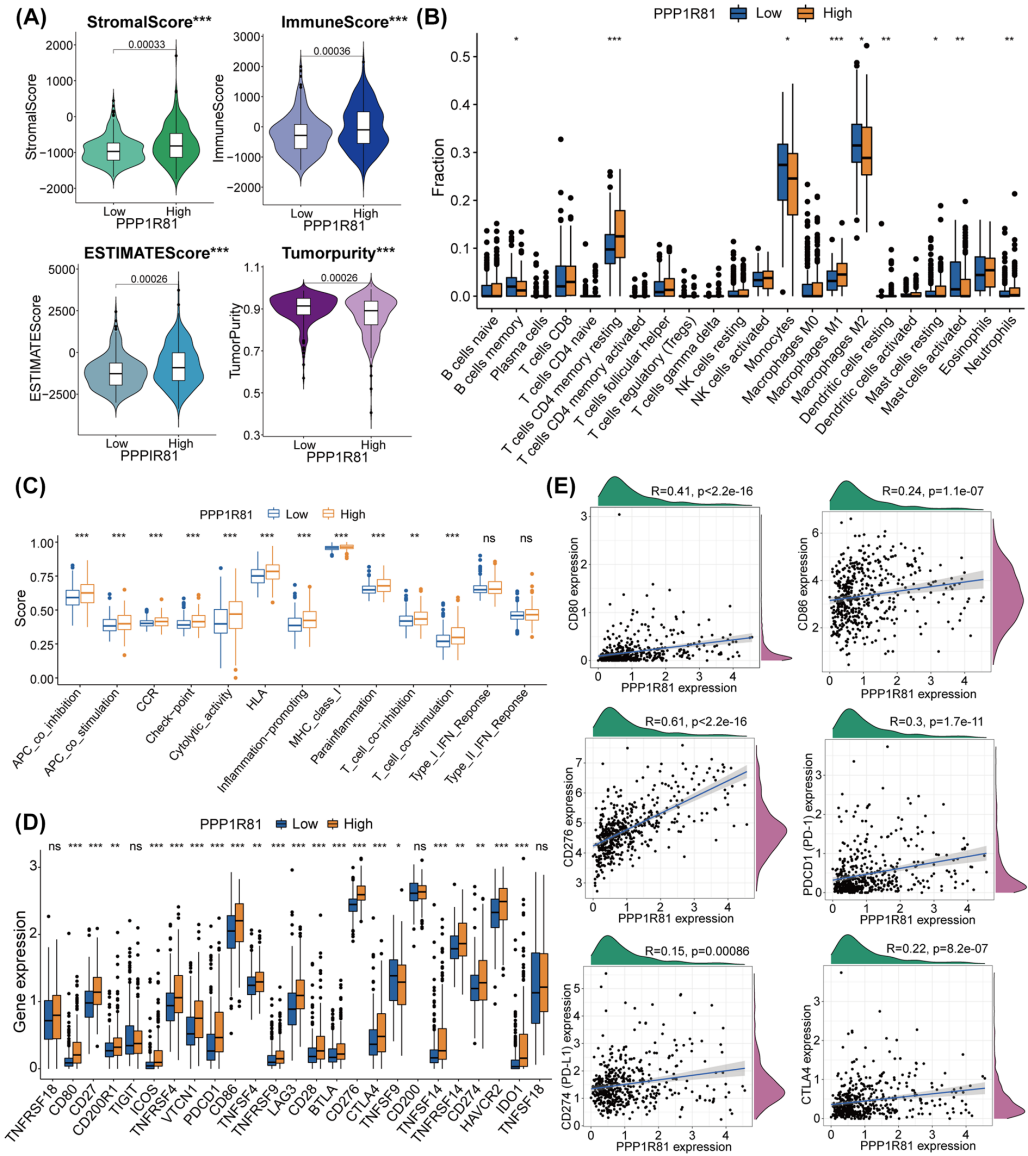
Different TIME and immunological patterns of the low-PPP1R81 and high-PPP1R81 expression subtypes in TCGA (**A**) Comparisons of the ESTIMATE, stromal, immune scores, and tumor purity between the two subtypes. (**B**) Distribution and abundance of 22 immune cells between the two subtypes. (**C**) Distinct immune-associated functions between the two subtypes. (**D**) Differential analysis of 25 ICPG expression levels between the two subtypes. (**E**) Correlation analysis between PPP1R81 expression and six common ICPGs expression (*P<0.05, **P<0.01, ***P<0.001).

The results of the GO analysis revealed an underlying association between PPP1R81, T-cell activation, and macrophage activation, which further prompted us to evaluate the association between PPP1R81 and immunological characteristics in LGG. First, we exploited the ESTIMATE algorithm tool to inspect the composition of the tumor microenvironment (TME) of the low-PPP1R81 and high-PPP1R81 subtypes. The results illustrated that PPP1R81 expression was positively connected with the ESTIMATE, immune, and stromal scores, but was inversely interrelated with tumor purity in TCGA cohort (Figure 7A). Similarly, the results were also found in CGGA dataset (Supplementary Figure S4A). Thereafter, we utilized CIBERSORT to measure the infiltration abundances of TIICs in LGG. In TCGA dataset, resting memory CD4 T cells, resting dendritic cells, M1 macrophages, and neutrophils were more enrich in high-PPP1R81 subgroup (Figure 7B). Similarly, the results were also detected in CGGA dataset (Supplementary Figure S4B).

To examine the conjunction between PPP1R81 expression and immune infiltration, we implemented the ssGSEA algorithm to measure the abundances of 13 immune-related factors. We detected that the abundances of most immune-associated signatures, such as CCR, ICPGs, and inflammation-promoting signatures, in high-PPP1R81 subtype were conspicuously higher than in low-PPP1R81 subtype in TCGA (Figure 7C) and CGGA (Supplementary Figure S4C) datasets. Furthermore, we inspected the differential expression of ICPGs in the two subtypes in TCGA (Figure 7D) and CGGA (Supplementary Figure S4D) cohorts. The results indicated that most ICPGs, including CD80, CD86, CD276, PDCD1 (PD-1), CD274 (PDL-1), and CTLA4, were positively linked to the expression of PPP1R81 (Figure 7E and Supplementary Figure S4E).

### *In vitro* validation of PPP1R81 expression in LGG samples

**Figure 8:**
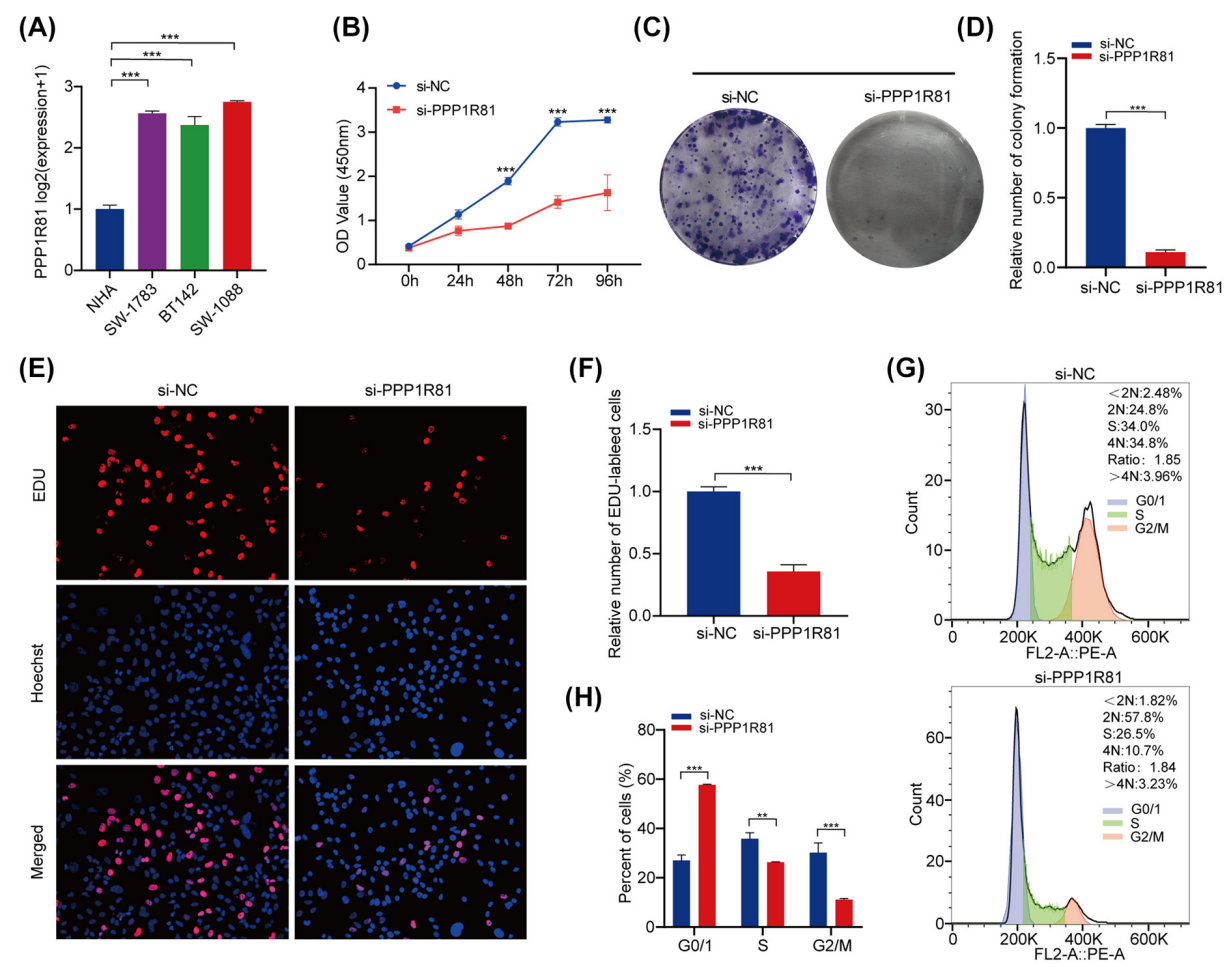
*In vitro* experiments of PPP1R81 in LGG (**A**) qRT-PCR analysis of PPP1R81 expression in LGG and NHA cell lines. (**B**) The cell viability of si-PPP1R81-transfected and si-NC-transfected SW1088 cells by CCK-8 assays. (**C,D**) Effect of down-regulation of PPP1R81 on colony formation in SW1088 cells was assessed. (**E,F**) EdU assays were executed to evaluate the cell proliferation after PPP1R81 knockdown in SW1088 cells. (**G,H**) Cell cycle assays were conducted to ascertain the cell distribution of the SW1088 cell lines after knockdown PPP1R81 (*P<0.05, **P<0.01, ***P<0.001).

We inspected the mRNA expression of PPP1R81 in NHA and three LGG cell lines. The results confirmed that PPP1R81 expression was higher in LGG cell lines than in NHA cell line (Figure 8A).

Next, we conducted functional experiments to inspect the conjunction between PPP1R81 expression and cell proliferation in LGG. CCK-8 assays indicated that the viability of SW1088 reduced obviously after down-regulating PPP1R81 (Figure 8B). Colony formation assays suggested that PPP1R81 knockdown markedly decreased cell colonies when compared with NC (Figure 8C,D). Moreover, EdU assays indicated that down-regulation of PPP1R81 expression notably inhibited SW1088 cells proliferation (Figure 8E,F). After silencing PPP1R81 in SW1088 cells, we found that the number of cells in the G0/1 phase was elevated. However, the number of cells in S and G2/M phase was decreased (Figure 8G,H). These results indicate that PPP1R81 is crucial for cell proliferation in LGG.

## Discussion

Traditional glioma therapies, such as surgery, chemotherapy, and radiotherapy, are still not very effective [28]. Wherefore, we should ascertain novel biomarkers of LGG in order to develop new targeted drugs to improve the therapeutic effect of glioma patients. PPP1R81, a cell cycle-associated protein, is closely linked to the malignant progression of various cancers [29–31]. However, the function of PPP1R81 in LGG remains unknown. First, we implemented a pan-cancer analysis of PPP1R81 in 33 cancers and found that PPP1R81 expression was positively related to poor prognosis, TMB burden, and ICPG expression in pan-LGG. Subsequently, we further comprehensively explored the connection between PPP1R81 expression, prognosis, clinical traits, genetic variations, and immunological characteristics in TCGA and CGGA LGG cohorts.

We employed KM analysis and found that higher PPP1R81 expression owned inferior prognosis in TCGA and CGGA cohorts. The Cox regression analyzes confirmed that PPP1R81 expression was an independent prognostic biomarker of LGG. Additionally, we developed a nomogram model to project the OS of LGG patients and the precision of the model was verified by calibration curves. Therefore, PPP1R81 may be a forceful prognostic biomarker of LGG.

To explore functional annotations and molecular pathways LGG associated with high-PPP1R81 and low-PPP1R81 expression subtypes, we implemented GO analysis, KEGG analysis, and GSVA in TCGA and CGGA databases. The results attested that PPP1R81 was closely related to these biological activities, such as T-cell activation, macrophage cell activation, the cell cycle, and the PI3K-Akt signaling pathway. Therefore, PPP1R81 could play a crucial part in regulation of immunity and tumor malignant progression. Due to the underlying effects of genomic mutations on the regulation of tumor immune infiltration, we performed a somatic mutation analysis and a CNA analysis. The results illustrated that the high PPP1R81 subgroup tended to have a higher TBM burden and CNA burden than the low PPP1R81 subgroup.

The TME is defined by the presence of stromal cells, infiltrating immune cells, and cancer cells [32]. Accumulating research has clarified that TME could affect the efficacy of tumor immunotherapy and chemotherapy [33–36]. Thus, we implemented CIBERSORT and ESTIMATE algorithms to identify the TIICs and the composition of TME of low-PPP1R81 and high-PPP1R81 expression subtypes. The results confirmed that the expression level of PPP1R81 was positively interrelated with the immune score, stromal score, and ESTIMATE score and inversely correlated with the tumor purity. In contrast with low-PPP1R81 expression subtype, the resting memory CD4 T cells, resting dendritic cells, M1 macrophages, and neutrophils were more enrich in high-PPP1R81 expression subtype. Additionally, we also applied the ssGSEA algorithm to examine immune functions between the two subtypes. The results illustrated that PPP1R81 was strongly interrelated with immune infiltration in TCGA and CGGA datasets. Increasing evidence has shown that immune checkpoint inhibitors have a marked therapeutic efficacy in the treatment of cancers [37,38]. Therefore, we carefully inspected the conjunction between PPP1R81 expression and ICPGs and determined that PPP1R81 expression was positively interrelated with the expression of ICPGs.

Additionally, *in vitro* experiments confirmed that PPP1R81 was elevated and essential for the cell proliferation and cell cycle in LGG. We detected that PPP1R81 was remarkably elevated in LGG cell lines when compared with NHA cell line. Additionally, we detected that the knockdown of PPP1R81 significantly impaired the proliferation abilities of LGG cells and induced cell cycle arrest. However, there are certain limitations to our research. The molecular mechanisms of PPP1R81 in LGG should be examined by conducting *in vivo* and *in vitro* experiments in the future. Future research should inspect whether PPP1R81 is a significant therapeutic target for LGG.

## Conclusion

In summary, the present study demonstrated that PPP1R81 was a robust prognostic biomarker and closely interrelated with the cell proliferation and cell cycle of LGG. PPP1R81 may represent a novel therapeutic target for LGG patients in the future.

## Supplementary Material

Supplementary Figures S1-S5

Supplementary Tables S1-S4

## Data Availability

The data analyzed in this research can be found in the TCGA (http://cancergenome.nih.gov/) and CGGA (http://www.cgga.org.cn/) websites.

## Abbreviations

ATCC: American Type Culture Collection
AUC: area under the curve
CGGA: Chinese Glioma Genome Atlas
CNA: copy number alterations
DEGs: differentially expressed genes
FDR: false discovery rate
FPKM: Fragments per Kilobase Million
GO-BP: Ontology biological process
GSVA: Gene Set Variation Analysis
GTEx: Genotype-Tissue Expression
ICPGs: immune checkpoint genes
IDH: isocitrate dehydrogenase
KEGG: Kyoto Encyclopedia of Genes and Genomes
LGG: lower-grade glioma
MGMT: 6-*O*-methylguanine-DNA methyltransferase
MOIs: multiplicities of infection
NHA: normal human astrocyte
OS: overall survival
PP1γ: phosphatase 1 γ
ROC: receiver operating characteristic
ssGSEA: single-sample gene set enrichment analysis
TCGA: The Cancer Genome Atlas
TIIC: tumor infiltrating immune cell
TMB: tumor mutation burden
TME: tumor microenvironment
TPM: Transcripts per Kilobase Million
WHO: World Health Organization
