# Supplementary material for: PPP1R81 correlates with the survival and cell proliferation in lower-grade glioma
Source: Biosci Rep. 2023 May 5;43(5):BSR20230028. doi: 10.1042/BSR20230028 (PMC10170297; doi:10.1042/BSR20230028)
Supplement: Supplementary Figures S1-S5 [file BSR-2023-0028_supp.pdf]

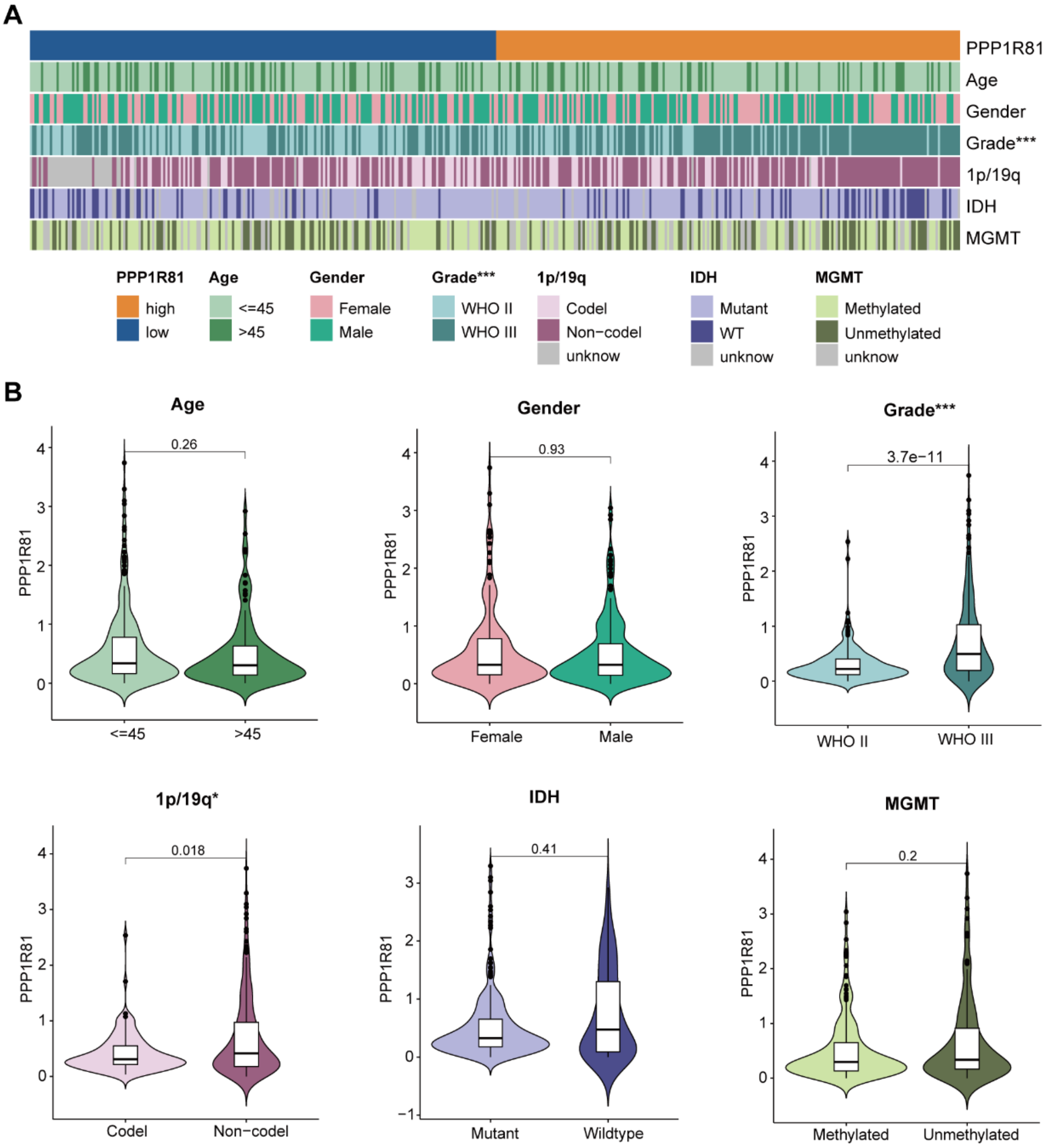

Supplementary Figure S1. Analysis of the connection between PPP1R81 and the clinical features of patients with LGG. (A) Association between PPP1R81 expression and LGG clinical features in CGGA cohort. **(B)** Analysis of variance of the expression of PPP1R81 and clinical characteristics in CGGA dataset. (*P < 0.05, **P < 0.01, ***P < 0.001).

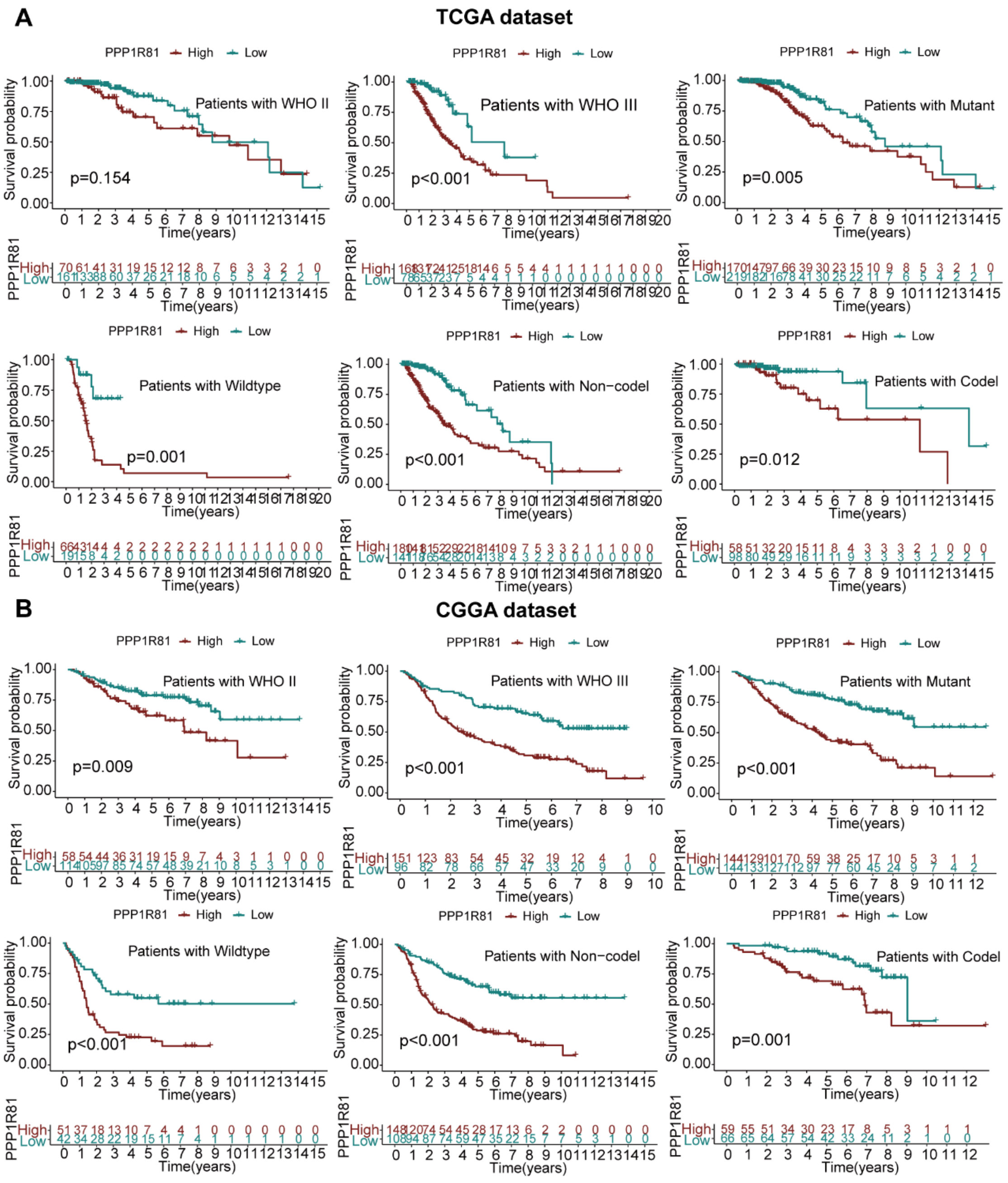

Supplementary Figure S2. Analysis of the correlation between PPP1R81 and the OS of patients with LGG. The Kaplan-Meier curves confirmed the different OS of clinical features, including WHO grade, IDH mutation status, and 1p/19q codel, of LGG patients between the low-PPP1R81 and high-PPP1R81 expression subtypes in TCGA **(A)** and CGGA **(B)** datasets.

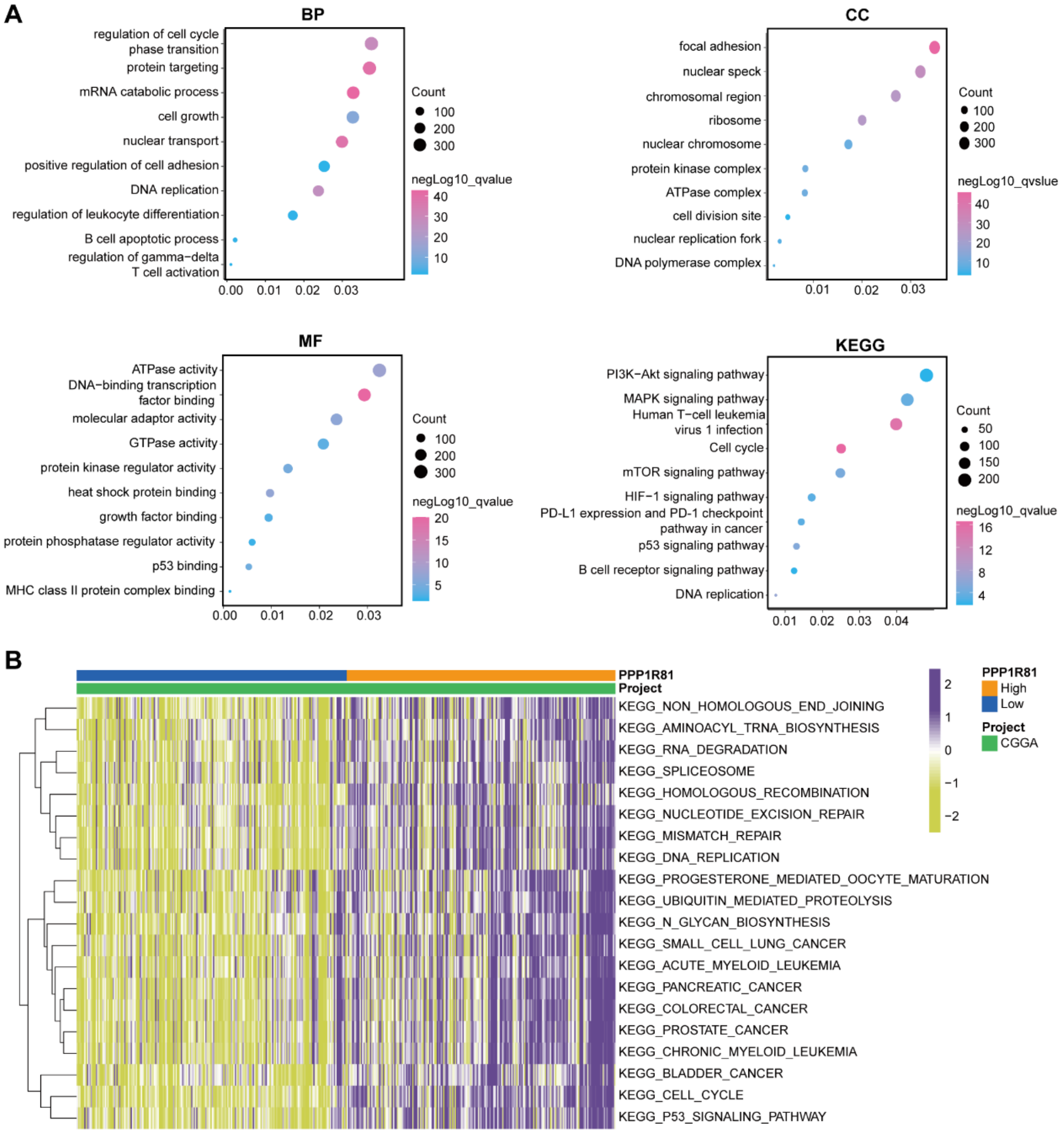

Supplementary Figure S3. Biological roles of PPP1R81 in LGG in the CGGA database. (A) Functional enrichment analyses for PPP1R81 in patients with LGG. **(B)** GSVA for PPP1R81 in patients with LGG.

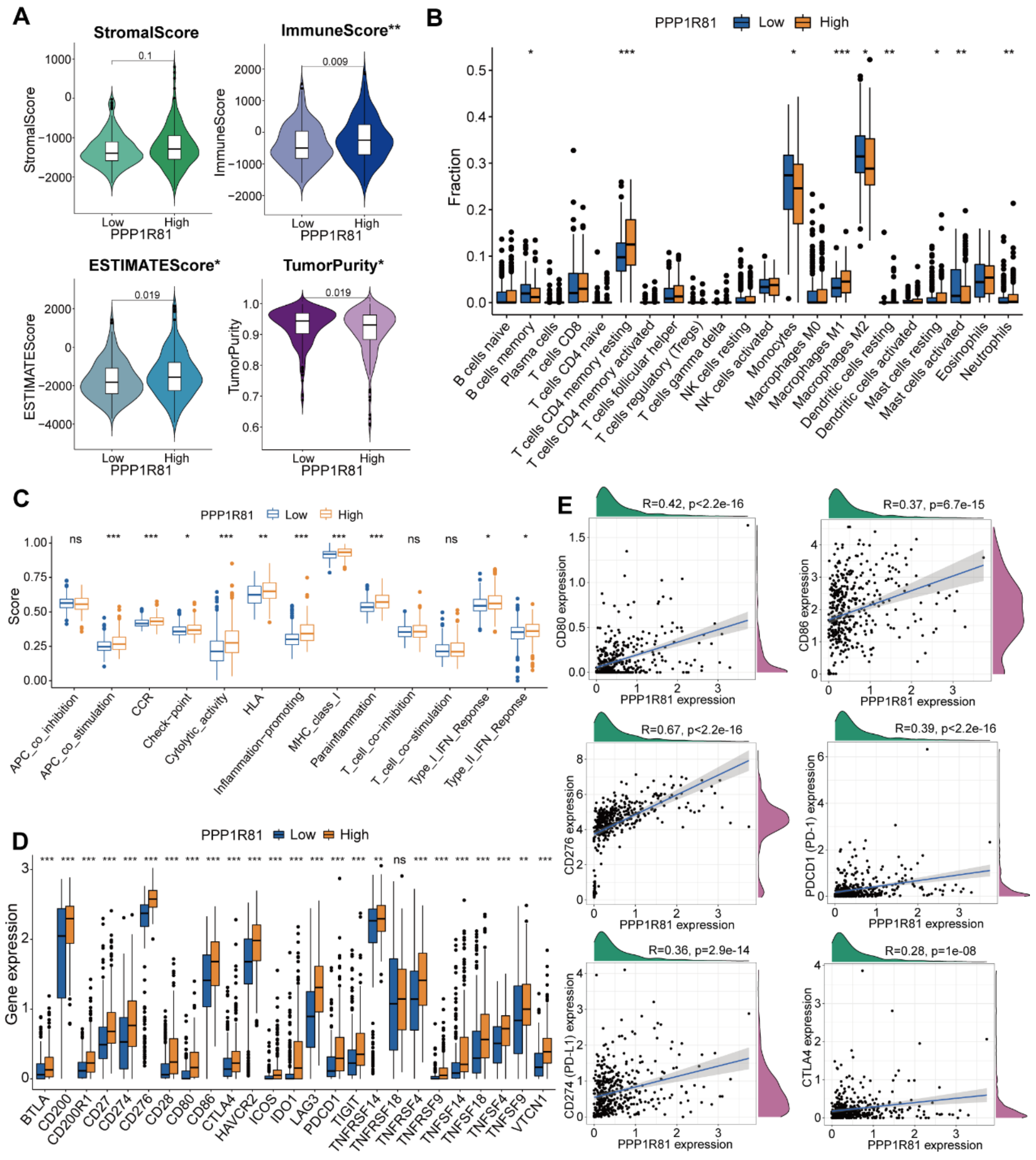

Supplementary Figure S4. Different TIME and immunological patterns of the low-PPP1R81 and high-PPP1R81 subtypes in CGGA. (A) Comparisons of the ESTIMATE, stromal, immune scores, and tumor purity between the two subtypes. **(B)** Different abundance of 22 immune cells between the two subtypes. **(C)** Differences in immune-associated functions between the two subtypes. **(D)** Differential analysis of 25 ICPG expression levels between the two subtypes. **(E)** Correlation analysis between PPP1R81 expression and six common ICPGs expression. (*P < 0.05, **P < 0.01, ***P < 0.001).
