# Supplementary material for: PPP1R81 correlates with the survival and cell proliferation in lower-grade glioma
Source: Biosci Rep. 2023 May 5;43(5):BSR20230028. doi: 10.1042/BSR20230028 (PMC10170297; doi:10.1042/BSR20230028)
Supplement: Supplementary Tables S1-S4 [file BSR-2023-0028_supp1.zip › BSR-2023-0028_suppS1.pdf]

Supplementary Table S1. Clinical features of LGG patients from TCGA

**Clinical features**		**Total (477)**	**%**
**Age**	Age <=45	287	60.17%
	Age >45	190	39.83 %
**Gender**	Female	216	45.28%
	Male	261	54.72%
**Grade**	WHO II	231	48.43%
	WHO III	246	51.57%
**1p/19q**	Non-codel	321	67.30%
	Codel	156	32.70%
**IDH**	Mutant	389	81.55%
	Wildtype	85	17.82%
	Unknow	3	0.63%
**MGMT**	Unmethylated	82	17.19%
	Methylated	395	82.81%
