# Supplementary material for: PPP1R81 correlates with the survival and cell proliferation in lower-grade glioma
Source: Biosci Rep. 2023 May 5;43(5):BSR20230028. doi: 10.1042/BSR20230028 (PMC10170297; doi:10.1042/BSR20230028)
Supplement: Supplementary Tables S1-S4 [file BSR-2023-0028_supp1.zip › BSR-2023-0028_suppS2.pdf]

Supplementary Table S2. Clinical features of LGG patients from CGGA

**Clinical features**		**Total (419)**	**%**
**Age**	Age <=45	302	72.08%
	Age >45	117	27.92%
**Gender**	Female	185	44.15%
	Male	234	55.85%
**Grade**	WHO II	172	41.05%
	WHO III	247	58.95%
**1p/19q**	Non-codel	256	61.10%
	Codel	125	29.83%
	Unknow	38	9.07%
**IDH**	Mutant	288	68.73%
	Wildtype	93	22.20%
	Unknow	38	9.07%
**MGMT**	Unmethylated	128	30.55%
	Methylated	200	47.73%
	Unknow	91	21.72%
