# Supplementary material for: PPP1R81 correlates with the survival and cell proliferation in lower-grade glioma
Source: Biosci Rep. 2023 May 5;43(5):BSR20230028. doi: 10.1042/BSR20230028 (PMC10170297; doi:10.1042/BSR20230028)
Supplement: Supplementary Tables S1-S4 [file BSR-2023-0028_supp1.zip › BSR-2023-0028_suppS3.pdf]

Supplementary Table S3. Differentially expressed genes (DEGs) in TCGA LGG cohort.

id	logFC	AveExpr	t	P.Value	adj.P.Val	B
**TOP2A**	2.807119	3.433001	26.09595	7.68E-94	4.21E-89	202.8975
**NUSAP1**	2.354027	3.640789	25.53947	3.08E-91	8.43E-87	196.9699
**TPX2**	2.207672	3.632731	25.20977	1.08E-89	1.98E-85	193.4475
**MKI67**	2.138591	2.284385	24.72325	2.10E-87	2.71E-83	188.2366
**UBE2C**	2.632374	3.433359	24.70816	2.47E-87	2.71E-83	188.0747
**CDK1**	1.994955	2.588331	24.62237	6.27E-87	5.72E-83	187.1543
**PBK**	2.403882	2.85034	24.58621	9.27E-87	7.26E-83	186.7663
**CENPF**	2.111774	2.407997	24.57168	1.09E-86	7.44E-83	186.6103
**PIMREG**	2.294878	2.583662	24.31398	1.78E-85	1.08E-81	183.8422
**KIF20A**	1.888107	1.676325	24.27901	2.60E-85	1.43E-81	183.4663
**BIRC5**	2.363094	2.716624	24.22309	4.78E-85	2.38E-81	182.865
**RRM2**	2.254023	2.367335	23.98024	6.70E-84	3.06E-80	180.2521
**FOXM1**	2.176675	3.068525	23.61386	3.61E-82	1.52E-78	176.3048
**KIFC1**	2.112007	2.842828	23.56487	6.16E-82	2.41E-78	175.7766
**BUB1**	1.675626	1.783074	23.41898	3.02E-81	1.10E-77	174.203
**CCNB2**	1.9856	2.277399	23.40111	3.67E-81	1.26E-77	174.0102
**MELK**	1.891508	1.839841	23.21069	2.93E-80	9.43E-77	171.9548
**CKAP2L**	1.659637	1.463633	23.19932	3.31E-80	1.01E-76	171.8321
**AURKB**	2.081886	2.134077	23.18745	3.77E-80	1.09E-76	171.7039
**GTSE1**	1.74835	1.879185	23.16409	4.86E-80	1.27E-76	171.4517
**NCAPG**	1.781846	1.662871	23.16369	4.88E-80	1.27E-76	171.4474
**CCNA2**	1.680661	2.538669	22.8821	1.05E-78	2.63E-75	168.4054
**IQGAP3**	1.628398	1.61023	22.85794	1.37E-78	3.27E-75	168.1443
**ASF1B**	1.921038	2.498543	22.84309	1.61E-78	3.69E-75	167.9838
**HJURP**	1.803267	1.719816	22.64496	1.40E-77	3.08E-74	165.8419
**NCAPH**	1.692955	2.207485	22.59676	2.38E-77	5.01E-74	165.3207
**CEP55**	1.550857	1.43643	22.54519	4.18E-77	8.48E-74	164.763
**NDC80**	1.78746	1.848322	22.51333	5.91E-77	1.16E-73	164.4184
**DLGAP5**	1.721752	1.366562	22.39676	2.11E-76	3.99E-73	163.1575
**KIF11**	1.504994	2.799759	22.10768	4.97E-75	9.09E-72	160.0296
**TROAP**	1.740676	1.622025	22.03426	1.11E-74	1.96E-71	159.235
**BUB1B**	1.817045	1.884441	21.86049	7.41E-74	1.27E-70	157.3542
**FAM111B**	1.568889	1.510245	21.85569	7.81E-74	1.30E-70	157.3022
**TTK**	1.524133	1.383982	21.81477	1.22E-73	1.97E-70	156.8593
**SKA3**	1.568514	1.790446	21.76368	2.14E-73	3.34E-70	156.3063
**KNL1**	1.349151	1.189545	21.6302	9.19E-73	1.40E-69	154.8613
**CDCA8**	1.75416	2.287186	21.57507	1.68E-72	2.46E-69	154.2646
**ARHGAP11A**	1.398711	2.04483	21.57364	1.70E-72	2.46E-69	154.2491
**ESPL1**	1.382031	1.199167	21.53116	2.71E-72	3.81E-69	153.7892
**KIF2C**	1.832635	2.335134	21.47455	5.03E-72	6.90E-69	153.1764
**CDCA2**	1.300647	1.069682	21.44265	7.13E-72	9.54E-69	152.8311

**HMMR**	1.378365	1.567149	21.31418	2.90E-71	3.79E-68	151.4406
**KIF4A**	1.746349	2.167521	21.2933	3.65E-71	4.65E-68	151.2145
**PTTG1**	1.792188	3.2565	21.25997	5.25E-71	6.54E-68	150.8538
**NUF2**	1.548656	2.072836	21.19237	1.10E-70	1.34E-67	150.1221
**MYBL2**	2.342082	2.573085	21.13536	2.05E-70	2.44E-67	149.5051
**DTL**	1.675831	1.994989	21.09373	3.23E-70	3.77E-67	149.0546
**CENPU**	1.772989	2.408627	20.96317	1.34E-69	1.54E-66	147.6418
**NEK2**	1.586487	1.569797	20.88404	3.19E-69	3.57E-66	146.7857
**SGO1**	1.234637	1.061803	20.85641	4.31E-69	4.73E-66	146.4868
**SKA1**	1.378686	1.273558	20.734	1.64E-68	1.76E-65	145.1627
**SPC24**	1.755235	2.235054	20.72891	1.74E-68	1.83E-65	145.1076
**KIF23**	1.445246	1.357645	20.56051	1.09E-67	1.13E-64	143.2867
**CENPA**	1.51572	1.378783	20.54224	1.33E-67	1.35E-64	143.0891
**CDC20**	2.012786	2.845754	20.38059	7.77E-67	7.74E-64	141.3419
**CDCA5**	1.592532	3.003299	20.36789	8.92E-67	8.73E-64	141.2047
**CDC25C**	1.236652	1.135558	20.36194	9.52E-67	9.16E-64	141.1404
**KIF14**	1.273352	1.092046	20.33628	1.26E-66	1.19E-63	140.8631
**SHCBP1**	1.311364	1.78694	20.33325	1.30E-66	1.21E-63	140.8304
**ASPM**	1.489913	1.135268	20.23388	3.85E-66	3.51E-63	139.7569
**PRC1**	1.448165	3.356271	20.20025	5.55E-66	4.99E-63	139.3937
**PCLAF**	1.570411	1.758283	20.07677	2.13E-65	1.89E-62	138.0604
**KIF15**	1.337943	2.34256	20.01737	4.07E-65	3.54E-62	137.4191
**ZNF367**	1.229013	2.660244	19.80392	4.16E-64	3.56E-61	135.1164
**ZWINT**	1.451199	3.591578	19.73193	9.11E-64	7.68E-61	134.3403
**NEIL3**	1.166298	0.917318	19.7041	1.23E-63	1.02E-60	134.0403
**TK1**	1.686252	2.949029	19.55649	6.14E-63	5.02E-60	132.4499
**AURKA**	1.257043	2.340212	19.54998	6.59E-63	5.31E-60	132.3798
**CDC45**	1.589339	1.87498	19.49902	1.15E-62	9.11E-60	131.831
**CCNB1**	1.420587	3.591426	19.41477	2.86E-62	2.24E-59	130.9241
**FANCD2**	1.083741	1.863117	19.34444	6.15E-62	4.74E-59	130.1673
**EXO1**	1.356597	1.301788	19.32309	7.75E-62	5.90E-59	129.9377
**RAD51**	1.206626	1.586038	19.30957	8.97E-62	6.74E-59	129.7922
**TACC3**	1.486768	2.96001	19.26339	1.48E-61	1.10E-58	129.2956
**MCM10**	1.376066	1.323006	19.12079	6.96E-61	5.08E-58	127.7629
**FANCI**	1.348842	2.663886	19.07693	1.12E-60	8.07E-58	127.2918
**RAD51AP1**	1.361337	2.390513	18.99285	2.78E-60	1.98E-57	126.389
**GINS1**	1.193711	2.847124	18.93992	4.94E-60	3.47E-57	125.8209
**ESCO2**	1.067224	0.886959	18.90302	7.36E-60	5.11E-57	125.4251
**POC1A**	1.158432	2.674527	18.71915	5.38E-59	3.69E-56	123.454
**TIMELESS**	1.205258	3.590061	18.71563	5.59E-59	3.78E-56	123.4163
**CENPE**	1.180558	1.090901	18.70679	6.15E-59	4.11E-56	123.3216
**HASPIN**	0.973289	0.862939	18.66017	1.02E-58	6.72E-56	122.8223
**CENPK**	1.226269	1.314827	18.63234	1.38E-58	8.97E-56	122.5243
**RAD54L**	1.236913	1.606793	18.50691	5.33E-58	3.44E-55	121.1823

**ECT2**	1.205804	2.808463	18.45685	9.15E-58	5.83E-55	120.647
**ORC1**	1.184656	1.391068	18.15972	2.25E-56	1.41E-53	117.475
**CENPH**	1.058648	3.175401	18.1425	2.70E-56	1.68E-53	117.2914
**EME1**	1.077193	1.870804	18.0818	5.19E-56	3.20E-53	116.6446
**DDIAS**	1.003131	1.295355	18.02684	9.37E-56	5.71E-53	116.0593
**PLK4**	1.053961	1.830197	17.96126	1.90E-55	1.14E-52	115.3612
**OIP5**	1.165752	1.851748	17.93242	2.58E-55	1.54E-52	115.0544
**E2F2**	1.348276	1.372179	17.82279	8.37E-55	4.94E-52	113.889
**CDKN3**	1.251292	2.218512	17.75352	1.76E-54	1.03E-51	113.1534
**BRCA2**	0.767088	0.820383	17.67196	4.21E-54	2.43E-51	112.2879
**BRIP1**	1.011616	0.913412	17.63825	6.04E-54	3.45E-51	111.9305
**CDC6**	1.157337	1.655195	17.63388	6.33E-54	3.58E-51	111.8841
**DEPDC1B**	1.145383	1.314785	17.62806	6.74E-54	3.77E-51	111.8225
**ERCC6L**	0.760838	0.681118	17.49348	2.84E-53	1.57E-50	110.3968
**SGO2**	0.97023	1.897193	17.48558	3.09E-53	1.69E-50	110.3132
**TCF19**	1.13006	3.274626	17.48494	3.11E-53	1.69E-50	110.3064
**TRIP13**	1.08302	2.075088	17.47632	3.41E-53	1.83E-50	110.2152
**CLSPN**	1.155969	1.15752	17.46188	3.98E-53	2.12E-50	110.0625
**LMNB1**	1.42037	4.247986	17.40028	7.68E-53	4.05E-50	109.411
**WDR62**	1.135253	1.346531	17.39328	8.28E-53	4.32E-50	109.3369
**KIF18A**	1.004011	0.976464	17.31976	1.81E-52	9.37E-50	108.5601
**CENPM**	1.261558	2.51935	17.23659	4.40E-52	2.25E-49	107.6822
**CDK2**	1.176796	3.447407	17.13267	1.33E-51	6.74E-49	106.5865
**PKMYT1**	1.262625	2.24747	17.06363	2.77E-51	1.39E-48	105.8594
**PLK1**	1.187492	2.637768	16.99438	5.77E-51	2.88E-48	105.131
**CENPI**	0.863995	1.241948	16.95401	8.86E-51	4.37E-48	104.7066
**MAD2L1**	0.995633	2.724292	16.94075	1.02E-50	4.99E-48	104.5672
**SMC4**	1.245269	2.469226	16.88578	1.83E-50	8.85E-48	103.9898
**POLQ**	0.746346	0.648036	16.88412	1.86E-50	8.93E-48	103.9724
**RTKN2**	0.910357	1.201509	16.72669	9.83E-50	4.68E-47	102.3214
**CHEK2**	0.843214	1.830043	16.69733	1.34E-49	6.33E-47	102.0139
**GINS2**	1.162181	2.548089	16.67709	1.66E-49	7.78E-47	101.8021
**GAS2L3**	1.136984	1.151583	16.65534	2.09E-49	9.70E-47	101.5745
**CKS2**	1.281744	5.186447	16.56395	5.48E-49	2.52E-46	100.6189
**PARPBP**	0.720451	1.123983	16.53019	7.82E-49	3.57E-46	100.2663
**MND1**	1.070119	1.590292	16.46786	1.51E-48	6.83E-46	99.61575
**KPNA2**	0.960285	5.592686	16.44774	1.86E-48	8.37E-46	99.40592
**MCM2**	1.164102	4.057274	16.43118	2.22E-48	9.89E-46	99.23318
**STIL**	0.888211	1.407518	16.41457	2.64E-48	1.17E-45	99.06005
**DEPDC1**	1.033512	0.770522	16.38362	3.66E-48	1.60E-45	98.73758
**NCAPG2**	0.90017	3.301794	16.35858	4.76E-48	2.07E-45	98.47675
**EZH2**	1.290799	3.368986	16.34766	5.34E-48	2.30E-45	98.36304
**CHEK1**	0.915092	2.364265	16.2838	1.04E-47	4.47E-45	97.69856
**KNTC1**	0.882596	2.097282	16.15952	3.84E-47	1.63E-44	96.40738

**ATAD2**	0.932004	3.56733	16.15778	3.91E-47	1.65E-44	96.38936
**FANCC**	0.798354	2.205319	16.10064	7.12E-47	2.98E-44	95.79669
**FAM83D**	1.29169	1.78354	16.09507	7.54E-47	3.13E-44	95.73899
**CENPL**	0.704412	2.03032	15.94164	3.75E-46	1.54E-43	94.15082
**C5orf34**	0.747785	1.629714	15.93621	3.97E-46	1.62E-43	94.09474
**XRCC2**	0.941358	1.464416	15.7867	1.88E-45	7.64E-43	92.55164
**WEE1**	1.245061	2.484842	15.78506	1.91E-45	7.72E-43	92.5347
**FANCA**	0.836297	1.487022	15.78031	2.01E-45	8.05E-43	92.48578
**MGME1**	0.694145	4.327572	15.76737	2.30E-45	9.14E-43	92.35243
**E2F7**	1.065035	0.877468	15.76457	2.37E-45	9.34E-43	92.32368
**TYMS**	1.518414	3.872871	15.73994	3.06E-45	1.20E-42	92.06992
**E2F8**	0.836708	0.758065	15.72387	3.62E-45	1.41E-42	91.90456
**CDT1**	1.220829	2.906918	15.69189	5.04E-45	1.95E-42	91.57549
**GINS4**	0.718217	1.352755	15.57041	1.78E-44	6.81E-42	90.32741
**SPAG5**	1.097169	2.899135	15.54732	2.26E-44	8.59E-42	90.0905
**ZWILCH**	0.837615	2.541989	15.51659	3.10E-44	1.17E-41	89.77538
**NEMP1**	0.839921	3.665335	15.50444	3.52E-44	1.32E-41	89.65092
**RFWD3**	0.754487	3.465605	15.49954	3.70E-44	1.38E-41	89.60072
**RACGAP1**	0.988177	4.050205	15.49791	3.76E-44	1.39E-41	89.58399
**DBF4**	0.813563	2.72411	15.46716	5.17E-44	1.90E-41	89.26898
**MCM8**	0.819632	2.131316	15.45562	5.83E-44	2.13E-41	89.15089
**CHAF1A**	0.92607	3.59669	15.43963	6.87E-44	2.49E-41	88.98719
**IKBIP**	0.864281	2.676219	15.43813	6.98E-44	2.52E-41	88.97183
**CENPW**	1.040636	2.848567	15.39726	1.06E-43	3.81E-41	88.55379
**DSN1**	0.820436	3.631657	15.33426	2.04E-43	7.25E-41	87.91014
**WDHD1**	0.766522	1.956704	15.28372	3.43E-43	1.21E-40	87.39444
**PCNA**	0.827573	6.781405	15.27898	3.60E-43	1.27E-40	87.34608
**ORC6**	0.948201	2.041639	15.27709	3.67E-43	1.28E-40	87.32682
**PRR11**	1.029599	2.816904	15.27626	3.70E-43	1.28E-40	87.31834
**CDC25A**	0.961237	2.178473	15.22941	6.00E-43	2.07E-40	86.84078
**CCDC15**	0.667126	1.570535	15.22121	6.52E-43	2.23E-40	86.75732
**HROB**	0.732011	1.726168	14.99699	6.50E-42	2.21E-39	84.4795
**E2F1**	1.196527	3.850835	14.99012	6.98E-42	2.36E-39	84.4099
**CDCA3**	0.933216	2.127872	14.98583	7.29E-42	2.45E-39	84.36644
**FBXO5**	0.929718	2.900005	14.94593	1.10E-41	3.66E-39	83.9624
**UBE2T**	1.215811	3.948774	14.91925	1.44E-41	4.78E-39	83.69248
**CASP2**	0.727947	3.590564	14.79281	5.22E-41	1.72E-38	82.41572
**BRCA1**	0.778313	2.310533	14.75998	7.29E-41	2.39E-38	82.08482
**CEP135**	0.731123	1.726993	14.75019	8.06E-41	2.63E-38	81.98621
**CKAP2**	0.964327	4.267233	14.72363	1.06E-40	3.42E-38	81.7188
**RAD18**	0.667393	2.585541	14.65353	2.15E-40	6.93E-38	81.01387
**CDCA4**	0.818557	2.764281	14.62882	2.76E-40	8.85E-38	80.76568
**KIF18B**	1.370704	2.641614	14.62083	3.00E-40	9.55E-38	80.68543
**NRM**	0.941132	4.086687	14.5898	4.10E-40	1.30E-37	80.374

**LRR1**	0.707137	2.641016	14.54355	6.55E-40	2.06E-37	79.91038
**CIP2A**	0.769987	2.020937	14.43567	1.95E-39	6.10E-37	78.83109
**TRAIP**	0.753087	2.248239	14.37658	3.53E-39	1.10E-36	78.24122
**HELLS**	0.761037	1.360265	14.37243	3.68E-39	1.14E-36	78.19982
**CENPO**	0.672145	3.332151	14.35046	4.60E-39	1.42E-36	77.98074
**LMNB2**	0.777677	5.082778	14.33216	5.53E-39	1.69E-36	77.79841
**CENPN**	0.717267	2.553642	14.31599	6.50E-39	1.98E-36	77.63731
**ARHGEF39**	0.561936	0.933505	14.3121	6.76E-39	2.05E-36	77.59859
**WDR76**	0.919281	2.811579	14.3041	7.33E-39	2.21E-36	77.51892
**KNSTRN**	0.71093	3.436277	14.23057	1.53E-38	4.59E-36	76.78776
**CDC7**	0.892374	3.490338	14.20121	2.06E-38	6.13E-36	76.49615
**POLA2**	0.598139	3.155265	14.19549	2.18E-38	6.46E-36	76.43937
**TUBB**	0.696592	8.781661	14.17146	2.77E-38	8.17E-36	76.20103
**XRN2**	0.637141	5.944902	14.16771	2.88E-38	8.44E-36	76.16385
**KIF20B**	0.660524	1.848175	14.12844	4.26E-38	1.24E-35	75.77462
**BORA**	0.591795	1.65134	14.12714	4.32E-38	1.25E-35	75.76168
**RMI2**	1.042423	2.05318	14.06455	8.08E-38	2.33E-35	75.14226
**PRIM2**	0.570656	2.516918	14.01449	1.33E-37	3.78E-35	74.64768
**RBL1**	0.718907	2.776908	13.95934	2.31E-37	6.51E-35	74.1036
**FAAP24**	0.635586	1.698821	13.93469	2.95E-37	8.28E-35	73.86073
**CDKN2C**	1.3895	4.363295	13.91941	3.43E-37	9.59E-35	73.71025
**BARD1**	0.845797	2.806078	13.87818	5.17E-37	1.44E-34	73.30459
**MCM4**	0.851488	4.814891	13.86845	5.69E-37	1.57E-34	73.20897
**MTFR2**	0.657294	1.028936	13.78272	1.33E-36	3.67E-34	72.36738
**HAUS8**	0.63187	1.996368	13.76705	1.55E-36	4.26E-34	72.21372
**MCM3**	0.665704	5.674403	13.75895	1.68E-36	4.59E-34	72.13436
**ODF2**	0.605178	4.298456	13.75591	1.74E-36	4.71E-34	72.10463
**EML4**	0.631019	3.651178	13.7419	1.99E-36	5.38E-34	71.96741
**NKIRAS2**	0.636006	4.671914	13.72696	2.31E-36	6.21E-34	71.82115
**TMPO**	0.71075	4.503526	13.71006	2.73E-36	7.30E-34	71.65573
**RNASEH2A**	0.790224	4.832033	13.66869	4.11E-36	1.09E-33	71.25137
**RFC2**	0.64635	5.197461	13.61045	7.29E-36	1.93E-33	70.68293
**POLD3**	0.538001	3.323983	13.59978	8.10E-36	2.14E-33	70.57882
**TOP3A**	0.519161	3.934503	13.59752	8.28E-36	2.17E-33	70.55687
**HMGB2**	0.859952	5.978076	13.5741	1.04E-35	2.72E-33	70.32867
**NCAPD2**	0.793476	4.845041	13.57072	1.08E-35	2.80E-33	70.29569
**AC091057.1**	0.682424	0.840037	13.52911	1.62E-35	4.20E-33	69.89079
**CCDC18**	0.682502	1.288294	13.52036	1.77E-35	4.55E-33	69.80562
**RFC3**	0.721546	3.483987	13.50599	2.04E-35	5.22E-33	69.66601
**SMC2**	0.774246	4.028361	13.47729	2.70E-35	6.88E-33	69.38715
**NUP205**	0.68834	4.598062	13.45481	3.37E-35	8.54E-33	69.16905
**NDC1**	0.65525	3.672418	13.42628	4.45E-35	1.12E-32	68.89237
**DNMT1**	0.701191	4.79685	13.4151	4.97E-35	1.24E-32	68.78398
**RCC1**	0.920265	3.96526	13.39192	6.23E-35	1.55E-32	68.55947

**C4orf46**	0.569705	2.968622	13.3898	6.36E-35	1.58E-32	68.53895
**GAS1**	1.18856	4.279858	13.38506	6.66E-35	1.64E-32	68.49311
**MXD3**	0.993264	2.218622	13.37824	7.12E-35	1.75E-32	68.42707
**TONSL**	0.776009	2.378196	13.36544	8.07E-35	1.97E-32	68.30321
**MCM5**	0.733122	4.03472	13.35762	8.71E-35	2.12E-32	68.22763
**H2AZ1**	0.546114	7.129538	13.32383	1.21E-34	2.94E-32	67.90102
**MCM6**	0.74752	4.629359	13.3221	1.23E-34	2.97E-32	67.88428
**POLE2**	0.682631	1.508822	13.29146	1.66E-34	3.99E-32	67.58855
**CKS1B**	0.659021	3.25976	13.28144	1.83E-34	4.38E-32	67.49182
**DCLRE1B**	0.680579	3.479883	13.27846	1.89E-34	4.49E-32	67.46311
**CALU**	0.832488	6.04556	13.25781	2.31E-34	5.47E-32	67.26404
**PTBP1**	0.675224	5.928092	13.21221	3.59E-34	8.45E-32	66.82488
**MMP2**	1.148769	4.856895	13.2054	3.84E-34	8.99E-32	66.7593
**POLE**	0.705468	2.445523	13.20158	3.98E-34	9.29E-32	66.7226
**KDELR2**	0.62596	6.45647	13.19365	4.30E-34	9.99E-32	66.64631
**RAD54B**	0.539097	1.025727	13.18726	4.58E-34	1.06E-31	66.58489
**NCAPD3**	0.658157	3.562738	13.16359	5.76E-34	1.33E-31	66.35741
**AC099850.4**	1.082311	3.04298	13.11475	9.25E-34	2.12E-31	65.88867
**GEN1**	0.62878	1.590542	13.08791	1.20E-33	2.74E-31	65.63135
**CBX3**	0.526017	6.764164	13.06231	1.54E-33	3.49E-31	65.38627
**DESI2**	0.633666	4.270905	13.02886	2.12E-33	4.81E-31	65.06631
**MSH6**	0.526108	3.926624	13.0209	2.29E-33	5.17E-31	64.99018
**PKN3**	0.666908	2.680462	13.00557	2.66E-33	5.95E-31	64.8437
**ZNF90**	0.556819	1.203203	12.99072	3.07E-33	6.84E-31	64.7019
**TRIM24**	0.703059	4.651188	12.98516	3.24E-33	7.18E-31	64.64878
**MASTL**	0.636726	2.897278	12.95836	4.19E-33	9.26E-31	64.39313
**C1orf112**	0.55742	1.856325	12.94519	4.76E-33	1.05E-30	64.2676
**CTPS1**	0.693129	3.763824	12.90245	7.18E-33	1.57E-30	63.86057
**LYPLA1**	0.572339	4.187569	12.89069	8.04E-33	1.76E-30	63.74867
**AC092718.4**	0.858707	3.185686	12.88988	8.10E-33	1.76E-30	63.74092
**FEN1**	0.663794	5.133955	12.88108	8.82E-33	1.91E-30	63.65724
**GJC1**	0.927348	2.055578	12.86707	1.01E-32	2.18E-30	63.52407
**COQ2**	0.525581	3.218008	12.82473	1.51E-32	3.25E-30	63.12199
**AC025176.1**	0.755894	0.61378	12.80576	1.82E-32	3.89E-30	62.94214
**COL4A1**	1.735562	4.092979	12.8042	1.84E-32	3.93E-30	62.92728
**APOBEC3B**	0.789282	1.292577	12.74806	3.15E-32	6.70E-30	62.39561
**KIF24**	0.652037	1.584216	12.72988	3.75E-32	7.94E-30	62.2237
**C18orf54**	0.669468	2.227421	12.72372	3.98E-32	8.39E-30	62.1654
**GGH**	0.779873	3.813639	12.71674	4.25E-32	8.93E-30	62.09949
**SPDL1**	0.532927	3.188464	12.7002	4.98E-32	1.04E-29	61.9432
**HAUS1**	0.5933	4.178325	12.6818	5.94E-32	1.24E-29	61.76948
**POLD1**	0.729191	3.427404	12.63467	9.30E-32	1.93E-29	61.32518
**SNRPB**	0.57261	7.415729	12.63323	9.43E-32	1.95E-29	61.31163
**FOXN4**	0.654236	0.760684	12.62077	1.06E-31	2.19E-29	61.19426

**ZNF486**	0.595937	2.588048	12.61855	1.08E-31	2.23E-29	61.17336
**ATAD5**	0.600984	1.671542	12.61407	1.13E-31	2.31E-29	61.13121
**CHST14**	0.642239	4.3409	12.57761	1.60E-31	3.26E-29	60.78827
**RFC4**	0.621808	3.905579	12.52836	2.55E-31	5.19E-29	60.32584
**SENP1**	0.530638	3.079487	12.51573	2.88E-31	5.82E-29	60.20746
**SDC1**	1.080664	2.163034	12.48383	3.89E-31	7.82E-29	59.90859
**SIKE1**	0.544057	4.208703	12.37722	1.07E-30	2.10E-28	58.91254
**MIS18A**	0.543497	4.029717	12.35332	1.33E-30	2.61E-28	58.68982
**FAM111A**	0.81913	3.287099	12.34831	1.40E-30	2.73E-28	58.64319
**EMC3-AS1**	0.537171	1.488041	12.32658	1.72E-30	3.34E-28	58.44092
**CDCA7**	1.069151	2.943438	12.28329	2.58E-30	4.99E-28	58.03855
**TMPO-AS1**	0.565263	1.542235	12.28254	2.60E-30	5.01E-28	58.03158
**PDIA4**	0.789297	6.465939	12.2799	2.66E-30	5.12E-28	58.00708
**XPR1**	0.61932	4.72983	12.27114	2.89E-30	5.54E-28	57.92577
**RECQL4**	0.836432	3.387928	12.26577	3.04E-30	5.80E-28	57.87587
**SKP2**	0.613688	3.717075	12.24417	3.72E-30	7.08E-28	57.67553
**TPM4**	0.667427	5.830605	12.21696	4.80E-30	9.07E-28	57.42342
**CEP152**	0.569537	1.252594	12.16259	7.98E-30	1.50E-27	56.92054
**IDH1**	0.68452	5.712688	12.15756	8.37E-30	1.57E-27	56.87411
**TICRR**	0.6746	1.488986	12.14364	9.53E-30	1.78E-27	56.74551
**RRM1**	0.590428	5.330784	12.14293	9.59E-30	1.78E-27	56.73894
**CHD1**	0.568869	3.318528	12.13493	1.03E-29	1.91E-27	56.66515
**RBBP8**	0.750357	3.636841	12.12985	1.08E-29	2.00E-27	56.61823
**NRAS**	0.592838	5.327374	12.12776	1.10E-29	2.03E-27	56.59902
**E2F3**	0.686675	4.012424	12.10944	1.31E-29	2.39E-27	56.43003
**AC012073.1**	0.634028	1.717554	12.10065	1.42E-29	2.58E-27	56.34908
**MEST**	1.069313	5.083993	12.09147	1.55E-29	2.80E-27	56.26448
**SLC30A7**	0.610705	2.756453	12.06644	1.96E-29	3.51E-27	56.03405
**IQGAP2**	1.255077	2.226323	12.06235	2.03E-29	3.64E-27	55.99645
**KDM4A**	0.596582	4.989873	12.06162	2.05E-29	3.65E-27	55.9897
**COL4A2**	1.496641	4.475923	12.03983	2.50E-29	4.43E-27	55.78939
**FKBP7**	0.527777	2.916738	12.00701	3.40E-29	5.99E-27	55.48803
**MCM7**	0.756872	6.427643	12.00295	3.53E-29	6.19E-27	55.45073
**LIG1**	0.665849	3.648605	12.00122	3.58E-29	6.26E-27	55.43484
**ZNF736**	0.593296	2.862204	11.99025	3.97E-29	6.88E-27	55.33431
**DEK**	0.557033	5.961526	11.98254	4.26E-29	7.37E-27	55.26354
**ALG6**	0.573467	2.872595	11.97131	4.73E-29	8.15E-27	55.16072
**PLEKHG2**	0.800448	3.523084	11.95399	5.55E-29	9.54E-27	55.00209
**FIGNL1**	0.574008	3.188704	11.93675	6.51E-29	1.12E-26	54.8443
**CNTRL**	0.622218	2.870196	11.93073	6.89E-29	1.17E-26	54.78926
**FAM126A**	0.75321	2.571375	11.92694	7.13E-29	1.21E-26	54.7546
**NANP**	0.534965	2.353693	11.91424	8.02E-29	1.34E-26	54.6385
**CCDC77**	0.574962	3.129268	11.91346	8.08E-29	1.35E-26	54.63136
**STK38**	0.582927	4.644124	11.9037	8.84E-29	1.47E-26	54.54222

**CMTM6**	0.646004	4.906582	11.88392	1.06E-28	1.76E-26	54.3616
**RCC2**	0.676082	6.260328	11.86087	1.31E-28	2.17E-26	54.15136
**FNDC3B**	0.679668	2.506928	11.83343	1.69E-28	2.77E-26	53.90141
**CD276**	0.758224	4.812796	11.82509	1.82E-28	2.99E-26	53.82547
**SMC5**	0.629357	4.018117	11.81406	2.02E-28	3.29E-26	53.72511
**C21orf58**	0.671218	2.110729	11.8085	2.13E-28	3.46E-26	53.6745
**ZGRF1**	0.551845	1.638516	11.79756	2.35E-28	3.81E-26	53.57506
**DSCC1**	0.601054	2.930166	11.79383	2.43E-28	3.92E-26	53.54119
**POFUT1**	0.54027	5.20104	11.78082	2.74E-28	4.41E-26	53.42298
**GLA**	0.545438	4.207822	11.74624	3.77E-28	6.00E-26	53.10912
**DRAXIN**	1.088022	2.467875	11.6656	7.88E-28	1.22E-25	52.37922
**URB2**	0.524002	3.292589	11.66047	8.26E-28	1.28E-25	52.33294
**KIRREL1**	0.779563	3.193358	11.63721	1.02E-27	1.57E-25	52.12292
**LIN9**	0.544033	2.276969	11.62636	1.13E-27	1.72E-25	52.02512
**NUP107**	0.545268	3.494366	11.56417	1.99E-27	2.96E-25	51.46513
**XPO5**	0.515464	4.542173	11.55745	2.11E-27	3.12E-25	51.4047
**BMP1**	0.616694	3.803299	11.5432	2.40E-27	3.54E-25	51.27667
**RPN2**	0.529045	7.785744	11.5402	2.47E-27	3.63E-25	51.24981
**PSMC3IP**	0.526808	3.041823	11.53625	2.56E-27	3.74E-25	51.21427
**SPC25**	1.156956	2.757687	11.51037	3.24E-27	4.72E-25	50.98213
**NUP188**	0.559908	4.913892	11.49793	3.62E-27	5.24E-25	50.87063
**ZNF107**	0.651161	3.082654	11.49292	3.79E-27	5.47E-25	50.82574
**HNRNPAB**	0.505065	7.056557	11.45541	5.33E-27	7.62E-25	50.48993
**JAG1**	0.908276	4.225007	11.45118	5.53E-27	7.90E-25	50.45215
**ZNF724**	0.599814	1.186245	11.42664	6.91E-27	9.81E-25	50.23287
**UBTD2**	0.509356	4.949714	11.40695	8.25E-27	1.17E-24	50.05712
**TOPBP1**	0.544079	4.488709	11.40418	8.46E-27	1.19E-24	50.03242
**MYO9B**	0.61808	5.342057	11.40229	8.61E-27	1.21E-24	50.01551
**ALDH2**	-0.62859	6.598337	-11.3714	1.14E-26	1.58E-24	49.74059
**IFNGR2**	0.571727	5.586358	11.36747	1.18E-26	1.63E-24	49.7053
**ERI1**	0.541503	3.024104	11.345	1.44E-26	1.99E-24	49.50532
**SEC61A1**	0.513258	7.54515	11.34436	1.45E-26	2.00E-24	49.49959
**PUS7**	0.570666	3.169531	11.33784	1.54E-26	2.11E-24	49.44161
**OSTC**	0.512531	5.598016	11.32116	1.79E-26	2.42E-24	49.29345
**MAP3K1**	0.757843	3.338883	11.31916	1.82E-26	2.46E-24	49.27561
**TTF2**	0.566177	1.910391	11.2995	2.17E-26	2.89E-24	49.10113
**SH3GLB1**	0.592244	5.712561	11.29597	2.24E-26	2.97E-24	49.06985
**DONSON**	0.556527	3.621304	11.28243	2.53E-26	3.35E-24	48.9497
**AC026401.3**	0.943913	3.224583	11.27212	2.77E-26	3.62E-24	48.85838
**RHNO1**	0.514152	5.098933	11.25954	3.11E-26	4.03E-24	48.7469
**PGM2**	0.626876	4.052959	11.24088	3.67E-26	4.73E-24	48.58178
**ZNF43**	0.567017	3.390362	11.22462	4.25E-26	5.42E-24	48.43797
**TEDC2**	0.702751	2.292758	11.2136	4.69E-26	5.97E-24	48.34058
**DDX39A**	0.582804	5.494411	11.19307	5.63E-26	7.11E-24	48.15934

**WDR3**	0.561995	3.665532	11.18193	6.22E-26	7.83E-24	48.06106
**DDOST**	0.525707	6.981539	11.14723	8.47E-26	1.05E-23	47.75525
**CRISPLD1**	1.009641	5.013715	11.14345	8.76E-26	1.09E-23	47.72198
**N4BP2**	0.601657	2.557105	11.14176	8.89E-26	1.10E-23	47.70716
**CASP3**	0.613717	4.967352	11.13683	9.29E-26	1.15E-23	47.6638
**MNS1**	0.693176	3.089478	11.12851	1.00E-25	1.23E-23	47.59059
**COL3A1**	1.610873	2.895843	11.11182	1.16E-25	1.41E-23	47.44386
**PTAR1**	0.589191	3.91003	11.10352	1.25E-25	1.51E-23	47.37095
**ZMYM1**	0.550774	2.637341	11.09645	1.33E-25	1.61E-23	47.30884
**DHFR**	0.717545	3.846521	11.06012	1.84E-25	2.19E-23	46.99017
**CTNND1**	0.511467	6.215612	11.05101	1.99E-25	2.36E-23	46.91035
**CD93**	1.164294	3.190174	11.04181	2.16E-25	2.55E-23	46.82976
**CDC25B**	0.560415	5.834465	11.04123	2.17E-25	2.56E-23	46.82471
**NASP**	0.598331	5.475785	11.01474	2.75E-25	3.17E-23	46.59295
**DHX40**	0.506469	5.17801	10.98767	3.49E-25	4.00E-23	46.35649
**ZIK1**	0.578067	2.996971	10.98173	3.68E-25	4.21E-23	46.30469
**HSPG2**	1.048388	2.370424	10.98012	3.73E-25	4.26E-23	46.29063
**ZNF480**	0.509221	3.265175	10.97529	3.89E-25	4.43E-23	46.24846
**CCT6A**	0.537432	7.085704	10.97421	3.93E-25	4.46E-23	46.23905
**TMEM45A**	0.729716	2.319668	10.96929	4.10E-25	4.65E-23	46.19617
**SELENON**	0.637044	7.069672	10.96555	4.24E-25	4.78E-23	46.1635
**NEDD1**	0.633408	3.599146	10.96554	4.24E-25	4.78E-23	46.16342
**ZNF468**	0.568622	2.268021	10.96373	4.31E-25	4.84E-23	46.14762
**SPIN4**	0.562663	2.309268	10.95176	4.79E-25	5.35E-23	46.04338
**LAMC1**	0.890393	4.215442	10.93381	5.61E-25	6.23E-23	45.88706
**CALD1**	0.719667	5.136988	10.92688	5.97E-25	6.61E-23	45.82669
**CDK6**	1.070136	3.493505	10.90642	7.14E-25	7.84E-23	45.64881
**SOAT1**	0.553849	4.134765	10.89751	7.72E-25	8.45E-23	45.57139
**INTS7**	0.575163	3.869493	10.8922	8.09E-25	8.84E-23	45.52527
**TNPO1**	0.547777	5.196201	10.8828	8.79E-25	9.50E-23	45.44365
**XXYLT1**	0.515756	3.622094	10.88037	8.98E-25	9.69E-23	45.42253
**CHIC2**	0.640837	4.523164	10.86825	9.99E-25	1.07E-22	45.31741
**CCNF**	0.546846	2.450973	10.86381	1.04E-24	1.11E-22	45.27896
**SLC4A7**	0.638729	2.751705	10.85646	1.11E-24	1.18E-22	45.2152
**MIS18BP1**	0.700207	2.437928	10.85268	1.15E-24	1.21E-22	45.18248
**CARHSP1**	0.620946	4.766089	10.84572	1.22E-24	1.28E-22	45.12213
**TGFBR1**	0.683804	4.704927	10.83999	1.28E-24	1.34E-22	45.0725
**POLH**	0.595842	3.485082	10.83764	1.31E-24	1.36E-22	45.05216
**PHACTR4**	0.547596	4.273942	10.82405	1.47E-24	1.53E-22	44.93456
**INCENP**	0.564991	3.928155	10.80566	1.73E-24	1.78E-22	44.77551
**RAI14**	0.542776	3.135038	10.79677	1.87E-24	1.91E-22	44.69877
**WRN**	0.56568	3.10999	10.78021	2.16E-24	2.20E-22	44.55577
**BAZ1A**	0.638654	3.44833	10.77779	2.21E-24	2.24E-22	44.53487
**PTGFRN**	0.96139	4.429092	10.7749	2.26E-24	2.29E-22	44.50995

**USP1**	0.549882	5.021592	10.76406	2.49E-24	2.50E-22	44.41647
**HMGN2**	0.587004	7.279494	10.76299	2.51E-24	2.52E-22	44.40725
**IFT81**	0.52523	3.574188	10.76027	2.57E-24	2.57E-22	44.38376
**TMEM131L**	0.607892	2.731958	10.74915	2.83E-24	2.82E-22	44.28794
**NUP160**	0.564201	4.128836	10.74711	2.88E-24	2.86E-22	44.27037
**COL1A2**	1.193528	3.450604	10.73392	3.23E-24	3.18E-22	44.15681
**CTTNBP2NL**	0.594017	3.886659	10.72739	3.42E-24	3.35E-22	44.10057
**REXO5**	0.573501	2.604042	10.71647	3.77E-24	3.67E-22	44.00662
**ZNF558**	0.517714	3.537539	10.70528	4.15E-24	4.01E-22	43.91049
**ING3**	0.511981	3.680864	10.70392	4.20E-24	4.04E-22	43.8988
**COLGALT1**	0.577982	5.090985	10.70309	4.23E-24	4.06E-22	43.89162
**IL10RB**	0.510789	4.459892	10.70206	4.27E-24	4.09E-22	43.88274
**DDX12P**	0.74485	1.484568	10.70139	4.29E-24	4.11E-22	43.87702
**PRIM1**	0.556229	3.281811	10.68608	4.91E-24	4.67E-22	43.74554
**FANCG**	0.535605	4.011992	10.684	4.99E-24	4.74E-22	43.7277
**RECQL**	0.727276	3.863868	10.67784	5.27E-24	4.99E-22	43.67482
**MYBL1**	0.54	2.09294	10.6597	6.17E-24	5.81E-22	43.51922
**DHRSX**	0.530239	4.137737	10.64119	7.24E-24	6.75E-22	43.3607
**DTYMK**	0.566253	4.903739	10.63501	7.64E-24	7.11E-22	43.3078
**LBR**	0.555315	4.834055	10.61172	9.35E-24	8.60E-22	43.10859
**GUSB**	0.554012	4.783109	10.59859	1.05E-23	9.60E-22	42.99641
**F2R**	0.955406	5.422002	10.56985	1.34E-23	1.22E-21	42.75112
**GNAI3**	0.518028	3.337705	10.55083	1.58E-23	1.43E-21	42.58904
**SIPA1L3**	0.553212	3.461086	10.54266	1.70E-23	1.53E-21	42.51944
**CBX7**	-0.63055	4.777827	-10.521	2.05E-23	1.83E-21	42.33485
**PRKDC**	0.566984	5.390801	10.5197	2.07E-23	1.85E-21	42.32413
**PRMT6**	0.513157	4.445092	10.48615	2.76E-23	2.44E-21	42.0392
**ENPEP**	0.747249	1.328918	10.47748	2.97E-23	2.62E-21	41.9656
**AC069499.2**	0.601959	1.224626	10.46591	3.29E-23	2.85E-21	41.8675
**NID1**	0.962877	4.729207	10.46277	3.38E-23	2.93E-21	41.84096
**ZNF28**	0.55925	2.723099	10.45253	3.69E-23	3.18E-21	41.75418
**ZNF45**	0.527644	3.431457	10.44845	3.82E-23	3.28E-21	41.71963
**WNT5A-AS1**	0.766723	1.638604	10.4473	3.85E-23	3.31E-21	41.70995
**IL1RAP**	0.986242	2.425706	10.42184	4.79E-23	4.07E-21	41.4946
**NADK**	0.534255	4.799937	10.41768	4.97E-23	4.21E-21	41.45936
**SBNO2**	0.590403	3.755338	10.39232	6.17E-23	5.15E-21	41.24529
**CMTM1**	0.566065	2.058808	10.38869	6.37E-23	5.29E-21	41.2146
**TRRAP**	0.536417	4.643015	10.38571	6.53E-23	5.41E-21	41.18953
**PIK3R3**	0.58823	3.999373	10.36726	7.64E-23	6.29E-21	41.03402
**B4GALT5**	0.576158	6.126581	10.35311	8.62E-23	7.03E-21	40.91482
**C1QTNF6**	0.553306	2.315082	10.34218	9.47E-23	7.65E-21	40.82287
**ZIM2-AS1**	0.590852	1.926232	10.34126	9.54E-23	7.70E-21	40.81511
**ADAM9**	0.672195	5.503002	10.34004	9.64E-23	7.77E-21	40.80488
**CDK4**	0.83745	6.24616	10.32586	1.09E-22	8.72E-21	40.68567

**PHC2**	0.501345	6.837571	10.31934	1.15E-22	9.17E-21	40.63084
**ODC1**	0.535647	6.969857	10.31912	1.15E-22	9.18E-21	40.62901
**YBX1P10**	0.637036	3.072662	10.31706	1.17E-22	9.30E-21	40.61167
**PTK7**	0.628052	3.791052	10.31303	1.21E-22	9.61E-21	40.57788
**ACTL6A**	0.528342	4.604242	10.30135	1.34E-22	1.06E-20	40.47985
**CD101**	0.733852	1.317013	10.2961	1.40E-22	1.10E-20	40.43577
**POLA1**	0.511437	3.811647	10.2663	1.80E-22	1.40E-20	40.18603
**H2AX**	0.627769	6.661801	10.26228	1.87E-22	1.44E-20	40.15242
**GPX7**	0.710622	3.866759	10.25476	1.99E-22	1.53E-20	40.08949
**TP53I3**	0.779706	4.097424	10.2512	2.05E-22	1.57E-20	40.05968
**TMEM106C**	0.618787	4.850956	10.24487	2.16E-22	1.65E-20	40.00678
**MAD2L2**	0.607491	5.287246	10.21836	2.71E-22	2.05E-20	39.78527
**RAB42**	0.769448	1.54573	10.21413	2.81E-22	2.12E-20	39.74995
**ZNF677**	0.553241	3.171554	10.21139	2.87E-22	2.16E-20	39.72709
**EFNB1**	0.528967	4.327619	10.21037	2.90E-22	2.18E-20	39.71854
**CHAF1B**	0.780061	1.800795	10.20701	2.98E-22	2.23E-20	39.69059
**LINC01224**	0.592211	0.564211	10.19629	3.26E-22	2.44E-20	39.60113
**DARS2**	0.504768	3.904434	10.19364	3.34E-22	2.49E-20	39.57908
**MAN2B1**	0.611944	4.778668	10.16373	4.30E-22	3.17E-20	39.33
**PDIA5**	0.684918	2.148924	10.15426	4.65E-22	3.41E-20	39.25127
**DTX3L**	0.743287	4.112368	10.15399	4.66E-22	3.41E-20	39.24904
**HAUS5**	0.564522	3.584366	10.14323	5.11E-22	3.71E-20	39.15956
**PARP11**	0.512683	3.333144	10.13167	5.63E-22	4.06E-20	39.06355
**SERPINB8**	0.629144	2.526972	10.12329	6.04E-22	4.34E-20	38.99399
**P3H1**	0.52469	3.008683	10.11711	6.36E-22	4.56E-20	38.94278
**ZNF217**	0.693251	2.865004	10.11365	6.55E-22	4.69E-20	38.91405
**BACH1**	0.507615	3.829978	10.09997	7.35E-22	5.21E-20	38.80065
**KDM1A**	0.559996	5.816963	10.0984	7.44E-22	5.26E-20	38.78761
**EPHB2**	0.748641	2.766203	10.08605	8.26E-22	5.79E-20	38.68533
**CHPF2**	0.514204	4.951145	10.08502	8.33E-22	5.83E-20	38.67686
**C19orf48**	0.584644	4.537642	10.08069	8.64E-22	6.03E-20	38.64101
**CMTM3**	0.66835	5.542587	10.06863	9.55E-22	6.62E-20	38.54126
**CHSY1**	0.522391	4.382366	10.06745	9.65E-22	6.67E-20	38.53145
**MICB**	0.660689	1.593246	10.05503	1.07E-21	7.38E-20	38.42882
**MPHOSPH9**	0.523803	2.59301	10.04395	1.17E-21	8.08E-20	38.33732
**HEATR1**	0.556916	3.421941	10.03588	1.26E-21	8.57E-20	38.27068
**CCNE2**	0.624417	2.177713	10.0329	1.29E-21	8.75E-20	38.24611
**NFIL3**	0.634847	4.959314	10.0294	1.33E-21	9.00E-20	38.21724
**AC010615.1**	0.637089	2.211315	10.02271	1.40E-21	9.46E-20	38.16207
**ZNF528**	0.561204	3.094803	10.02059	1.43E-21	9.59E-20	38.14458
**CAPN5**	0.810563	4.835605	10.00806	1.59E-21	1.06E-19	38.04134
**TXLNA**	0.547478	5.307038	9.991667	1.82E-21	1.21E-19	37.90641
**ETV6**	0.517181	3.548154	9.990831	1.83E-21	1.21E-19	37.89954
**CCDC34**	0.524402	3.185602	9.981226	1.98E-21	1.31E-19	37.82053

**SLC26A2**	0.638714	2.616757	9.977044	2.05E-21	1.35E-19	37.78616
**ZYX**	0.646473	6.832735	9.965655	2.26E-21	1.47E-19	37.69257
**CREB5**	0.764464	3.569243	9.964853	2.27E-21	1.48E-19	37.68599
**HYAL2**	0.517666	4.772188	9.959911	2.37E-21	1.53E-19	37.6454
**BTG3**	0.579651	5.084021	9.954951	2.47E-21	1.59E-19	37.60468
**VANGL1**	0.617174	2.03005	9.948361	2.61E-21	1.67E-19	37.5506
**FAAP100**	0.504994	4.879146	9.937719	2.85E-21	1.82E-19	37.46332
**ITGB3BP**	0.539139	2.357006	9.937452	2.86E-21	1.82E-19	37.46113
**FLNA**	0.853592	6.569307	9.923783	3.20E-21	2.02E-19	37.34911
**H3C10**	0.636525	1.04166	9.908393	3.63E-21	2.27E-19	37.22311
**CTNNAL1**	0.528412	4.292895	9.907073	3.67E-21	2.29E-19	37.21231
**TTYH3**	0.688258	6.328224	9.90555	3.72E-21	2.32E-19	37.19985
**TTC26**	0.6243	2.291466	9.883412	4.47E-21	2.76E-19	37.01886
**TMEM255A**	0.902124	4.90809	9.880993	4.56E-21	2.81E-19	36.9991
**SLC16A1**	0.572033	5.996538	9.869219	5.03E-21	3.09E-19	36.90297
**TMX1**	0.504575	5.247803	9.850916	5.85E-21	3.55E-19	36.75368
**TRAM1**	0.500863	6.118641	9.834926	6.68E-21	4.02E-19	36.6234
**FSTL1**	0.916016	4.585006	9.830705	6.91E-21	4.15E-19	36.58903
**FN1**	0.957446	5.719356	9.817741	7.69E-21	4.58E-19	36.48354
**CNPY4**	0.521864	4.842122	9.815999	7.80E-21	4.63E-19	36.46938
**POM121**	0.510533	3.978012	9.811518	8.10E-21	4.80E-19	36.43293
**CNOT1**	0.531742	4.967956	9.810413	8.17E-21	4.84E-19	36.42395
**ZC3HAV1**	0.557656	4.272567	9.799647	8.93E-21	5.25E-19	36.33646
**CLIC4**	0.68328	7.524552	9.790848	9.60E-21	5.62E-19	36.265
**TANC1**	0.599669	3.89715	9.78303	1.02E-20	5.97E-19	36.20155
**LOXL2**	0.946806	2.852381	9.776992	1.08E-20	6.27E-19	36.15256
**SLC35D1**	0.523892	3.31869	9.776179	1.08E-20	6.30E-19	36.14597
**POGLUT2**	0.532889	2.877465	9.768899	1.15E-20	6.66E-19	36.08693
**ABCD1**	0.503693	3.535013	9.757401	1.26E-20	7.29E-19	35.99376
**FEM1C**	0.504998	4.625949	9.756356	1.27E-20	7.34E-19	35.9853
**ZNF93**	0.541408	2.243119	9.753678	1.30E-20	7.49E-19	35.9636
**IGFBP2**	1.512888	3.750105	9.750735	1.34E-20	7.65E-19	35.93977
**YBX1**	0.599472	8.970929	9.737282	1.49E-20	8.46E-19	35.83089
**RNF122**	0.763158	3.934552	9.733838	1.53E-20	8.69E-19	35.80304
**GBE1**	0.555957	4.040874	9.728117	1.61E-20	9.09E-19	35.75678
**TCF3**	0.548939	5.671663	9.696927	2.08E-20	1.15E-18	35.5049
**NBPF8**	0.529109	1.785853	9.693596	2.13E-20	1.18E-18	35.47804
**BNIP3P17**	0.516904	1.296857	9.670741	2.57E-20	1.41E-18	35.29385
**AC108463.1**	0.707646	0.908222	9.669494	2.60E-20	1.42E-18	35.28381
**SAMD9**	0.826406	2.562879	9.668159	2.63E-20	1.43E-18	35.27306
**ANKRD50**	0.550342	3.488063	9.660764	2.79E-20	1.52E-18	35.21354
**TRAM2**	0.655533	3.112836	9.659903	2.81E-20	1.52E-18	35.20662
**MXRA5**	0.937768	1.475305	9.643525	3.21E-20	1.73E-18	35.07491
**CTDSP2**	0.525659	6.725789	9.643004	3.22E-20	1.73E-18	35.07072

**ITGB1**	0.65999	5.299119	9.638554	3.34E-20	1.79E-18	35.03496
**SERPINH1**	0.909496	4.26308	9.6381	3.35E-20	1.80E-18	35.03132
**ZNF354C**	0.520417	3.388189	9.634781	3.45E-20	1.85E-18	35.00465
**AC073046.1**	0.617605	1.352107	9.633622	3.48E-20	1.86E-18	34.99534
**SH3PXD2B**	0.593678	4.818953	9.608229	4.28E-20	2.26E-18	34.79157
**COL1A1**	1.38419	2.61381	9.603764	4.44E-20	2.34E-18	34.75579
**RP2**	0.563738	4.133571	9.59654	4.70E-20	2.47E-18	34.6979
**RELL1**	0.675796	3.029899	9.546845	7.04E-20	3.60E-18	34.30049
**DIAPH3**	0.507382	1.226519	9.544344	7.18E-20	3.67E-18	34.28054
**C19orf57**	0.530147	3.134104	9.543484	7.23E-20	3.68E-18	34.27367
**FREM2**	0.946801	1.510933	9.526938	8.27E-20	4.18E-18	34.14169
**DSEL**	0.684596	3.978402	9.521406	8.65E-20	4.36E-18	34.0976
**SMC1A**	0.542656	5.056728	9.504929	9.88E-20	4.96E-18	33.96638
**LDHD**	-0.59586	4.612273	-9.50298	1.00E-19	5.04E-18	33.95085
**NEURL1B**	0.69291	4.027802	9.49795	1.05E-19	5.23E-18	33.91086
**HNF4G**	0.526677	1.425357	9.48269	1.18E-19	5.88E-18	33.78953
**ST8SIA4**	0.640573	2.297743	9.481939	1.19E-19	5.92E-18	33.78356
**LAPTM4B**	0.50075	7.197817	9.480668	1.20E-19	5.97E-18	33.77346
**NDE1**	0.602803	3.308669	9.475262	1.26E-19	6.21E-18	33.73052
**TP53INP1**	0.583615	4.266985	9.468136	1.33E-19	6.55E-18	33.67394
**KDM5A**	0.543527	3.803689	9.461384	1.40E-19	6.89E-18	33.62035
**TES**	0.556244	1.777677	9.456348	1.46E-19	7.15E-18	33.58041
**HTRA3**	0.775306	1.179284	9.446637	1.58E-19	7.66E-18	33.50341
**ADAM17**	0.577193	4.443439	9.424374	1.89E-19	9.05E-18	33.3271
**ZNF92**	0.535114	4.245555	9.399405	2.31E-19	1.10E-17	33.1297
**PPP1R18**	0.554831	5.543133	9.398908	2.32E-19	1.10E-17	33.12578
**FBLIM1**	0.761331	2.228987	9.396872	2.36E-19	1.12E-17	33.10969
**FBXW8**	0.511542	3.938923	9.372877	2.86E-19	1.33E-17	32.92036
**HCG15**	0.510239	1.486459	9.369675	2.93E-19	1.37E-17	32.89513
**BICD1**	0.54443	3.076548	9.356914	3.24E-19	1.50E-17	32.7946
**CDCA7L**	0.912406	3.3403	9.354108	3.32E-19	1.53E-17	32.77251
**AL662791.1**	0.587773	1.597344	9.346445	3.53E-19	1.63E-17	32.71219
**POU3F2**	0.608466	5.369563	9.344217	3.59E-19	1.65E-17	32.69467
**BZW2**	0.545116	5.013351	9.331225	3.98E-19	1.82E-17	32.59252
**AC027130.1**	-0.81581	2.39269	-9.32261	4.27E-19	1.95E-17	32.52481
**STK36**	0.537736	4.245241	9.321027	4.32E-19	1.97E-17	32.5124
**RPS6KA3**	0.501182	3.676374	9.310453	4.70E-19	2.13E-17	32.42939
**TGIF1**	0.76781	2.741515	9.30149	5.05E-19	2.28E-17	32.35908
**TP53**	0.690017	5.448271	9.301047	5.07E-19	2.28E-17	32.3556
**PROSER3**	0.534842	2.259628	9.291763	5.45E-19	2.44E-17	32.28282
**PLXNA3**	0.511895	4.126922	9.291113	5.48E-19	2.45E-17	32.27773
**UHRF1**	0.846019	4.097236	9.290002	5.53E-19	2.47E-17	32.26902
**COL5A2**	1.016595	3.028377	9.28682	5.67E-19	2.53E-17	32.24409
**WNT5A**	0.826599	3.260585	9.283788	5.81E-19	2.59E-17	32.22035

**TUBA1C**	0.794629	3.187109	9.26478	6.76E-19	2.96E-17	32.07159
**JPT2**	0.581162	4.360031	9.2632	6.84E-19	3.00E-17	32.05923
**HS2ST1**	0.56174	4.519309	9.262042	6.91E-19	3.02E-17	32.05018
**AC011447.7**	0.576082	1.973902	9.248527	7.69E-19	3.34E-17	31.94455
**MEX3C**	0.511398	4.656312	9.247972	7.72E-19	3.35E-17	31.94022
**PTX3**	0.987138	1.996733	9.24006	8.22E-19	3.55E-17	31.87845
**VASH1**	0.515391	5.477911	9.237535	8.39E-19	3.61E-17	31.85874
**ITGA5**	0.783038	3.060782	9.236092	8.48E-19	3.65E-17	31.84748
**GNS**	0.559124	5.673419	9.209299	1.05E-18	4.43E-17	31.6386
**REEP4**	0.581755	3.975426	9.201659	1.11E-18	4.69E-17	31.57912
**AL109918.1**	0.609007	3.472043	9.194329	1.18E-18	4.95E-17	31.52208
**TSPAN12**	0.830149	4.794125	9.182523	1.30E-18	5.39E-17	31.43028
**DPEP1**	0.975389	1.047969	9.179842	1.32E-18	5.50E-17	31.40944
**DOCK11**	0.575142	3.161925	9.179342	1.33E-18	5.52E-17	31.40556
**ZNF300**	0.710023	3.758576	9.163	1.51E-18	6.24E-17	31.27865
**UGCG**	0.503799	4.238037	9.157539	1.58E-18	6.49E-17	31.23628
**PTN**	0.773934	9.333912	9.15699	1.58E-18	6.51E-17	31.23203
**ZNF436**	0.584352	4.569071	9.153322	1.63E-18	6.69E-17	31.20357
**WDR34**	0.523528	5.590765	9.150417	1.67E-18	6.84E-17	31.18105
**UBE2S**	0.603212	4.034526	9.145414	1.74E-18	7.09E-17	31.14226
**H6PD**	0.514535	4.408004	9.138754	1.83E-18	7.45E-17	31.09066
**ITGA1**	0.55317	1.892389	9.127173	2.00E-18	8.10E-17	31.00099
**TMEM140**	0.555824	4.424773	9.110046	2.29E-18	9.22E-17	30.86853
**ANTXR2**	0.632804	2.382492	9.106191	2.36E-18	9.49E-17	30.83873
**ZFP36L2**	0.635594	6.850456	9.105136	2.38E-18	9.56E-17	30.83058
**ARAP3**	0.722421	2.911708	9.079927	2.90E-18	1.15E-16	30.63599
**TMEM51**	0.592669	3.96951	9.054861	3.53E-18	1.38E-16	30.44289
**PHTF1**	0.509176	2.668078	9.054525	3.54E-18	1.38E-16	30.44031
**TMSB15A**	1.228814	3.682333	9.052089	3.61E-18	1.40E-16	30.42156
**GNL3L**	0.55936	3.363973	9.035536	4.11E-18	1.59E-16	30.29428
**GMIP**	0.525986	3.989402	9.033607	4.17E-18	1.61E-16	30.27945
**H2BC11**	0.590047	1.262854	9.032204	4.21E-18	1.63E-16	30.26867
**MRO**	-0.83198	5.440223	-9.02409	4.49E-18	1.73E-16	30.20636
**LIMA1**	0.728122	5.786333	9.022086	4.56E-18	1.75E-16	30.19097
**CBX2**	0.717018	2.441722	9.0193	4.66E-18	1.78E-16	30.16958
**LAMB1**	0.925529	3.246525	9.017838	4.71E-18	1.80E-16	30.15836
**DOT1L**	0.624692	3.906889	9.015097	4.82E-18	1.84E-16	30.13733
**STK17A**	0.546135	4.574098	9.005968	5.17E-18	1.96E-16	30.06731
**JPT1**	0.539785	6.311491	8.991247	5.80E-18	2.19E-16	29.95452
**TGFB1I1**	0.635942	3.110305	8.983926	6.14E-18	2.31E-16	29.89846
**AC007240.1**	0.516528	0.402735	8.970421	6.82E-18	2.55E-16	29.79515
**ADAM19**	0.698554	2.262554	8.968861	6.90E-18	2.57E-16	29.78323
**PCDH18**	0.625999	2.890944	8.953725	7.76E-18	2.87E-16	29.66759
**SLC4A2**	0.555595	4.936274	8.93967	8.65E-18	3.18E-16	29.56033

**SPRY1**	0.844218	3.086609	8.93331	9.09E-18	3.33E-16	29.51184
**PARP9**	0.692868	3.725618	8.926342	9.59E-18	3.50E-16	29.45874
**IL18BP**	0.554138	3.636484	8.920837	1.00E-17	3.65E-16	29.4168
**IGF2BP3**	0.674407	0.474906	8.892168	1.25E-17	4.49E-16	29.19872
**PLOD1**	0.504558	5.656816	8.886516	1.31E-17	4.66E-16	29.15579
**AC093673.1**	0.637278	4.837239	8.885664	1.31E-17	4.68E-16	29.14932
**GTF2IP23**	0.595635	2.063845	8.883265	1.34E-17	4.77E-16	29.1311
**SPINK8**	0.830171	2.335905	8.877309	1.40E-17	4.97E-16	29.0859
**SHC1**	0.519589	4.699138	8.857974	1.63E-17	5.74E-16	28.93927
**COL6A1**	0.646154	6.556066	8.84093	1.85E-17	6.49E-16	28.81022
**KLF10**	0.725369	4.277097	8.831792	1.99E-17	6.95E-16	28.7411
**MPZL1**	0.511011	5.660903	8.829596	2.02E-17	7.06E-16	28.72449
**SOGA1**	0.558586	5.376322	8.828967	2.03E-17	7.09E-16	28.71974
**STK40**	0.559193	4.700651	8.826416	2.07E-17	7.22E-16	28.70046
**PAG1**	0.512063	4.131502	8.826388	2.07E-17	7.22E-16	28.70024
**MYCBP**	0.502668	2.861202	8.80868	2.38E-17	8.22E-16	28.56651
**TRIO**	0.611546	4.858342	8.806434	2.42E-17	8.35E-16	28.54956
**ITPRIPL2**	0.564209	3.060187	8.793054	2.68E-17	9.21E-16	28.44866
**GAL3ST4**	0.640383	4.816872	8.789886	2.74E-17	9.40E-16	28.42478
**PLSCR1**	0.794682	3.731421	8.782942	2.89E-17	9.89E-16	28.37247
**TP73**	0.657689	1.052887	8.778142	3.00E-17	1.02E-15	28.33633
**ELN**	0.816915	5.094504	8.77453	3.09E-17	1.05E-15	28.30914
**PANX1**	0.524404	4.522861	8.771619	3.15E-17	1.07E-15	28.28723
**APOL4**	1.153531	2.255877	8.767485	3.26E-17	1.10E-15	28.25614
**PLAU**	1.047684	2.708551	8.759963	3.45E-17	1.16E-15	28.19958
**CASP6**	0.551117	2.930207	8.748394	3.77E-17	1.27E-15	28.11265
**MSR1**	0.936056	2.524604	8.742495	3.94E-17	1.33E-15	28.06837
**AL390728.4**	0.599697	3.550037	8.73566	4.15E-17	1.39E-15	28.01708
**ADAM12**	0.762182	1.057144	8.730547	4.32E-17	1.45E-15	27.97873
**CCND2**	0.750005	6.677452	8.729565	4.35E-17	1.46E-15	27.97136
**AC073415.1**	0.606339	1.649164	8.716651	4.80E-17	1.60E-15	27.87459
**CDH11**	0.531681	4.088063	8.712182	4.96E-17	1.65E-15	27.84112
**CD248**	0.918888	2.705331	8.694703	5.67E-17	1.87E-15	27.71034
**NDRG2**	-0.6821	9.358185	-8.68917	5.91E-17	1.94E-15	27.66899
**SHOX2**	0.936226	0.678925	8.684555	6.12E-17	2.01E-15	27.63451
**PLCE1**	0.581204	2.878906	8.673621	6.65E-17	2.17E-15	27.55287
**POGLUT3**	0.722571	3.42497	8.670737	6.80E-17	2.21E-15	27.53135
**AC090692.1**	0.901117	2.427308	8.65413	7.71E-17	2.50E-15	27.40752
**AL035461.3**	0.565117	2.655876	8.639297	8.63E-17	2.78E-15	27.29707
**SLC2A4**	-0.68719	1.952185	-8.6376	8.74E-17	2.81E-15	27.28443
**TUBA1B**	0.526779	7.966529	8.617642	1.02E-16	3.24E-15	27.13606
**PCOLCE**	0.770404	2.937605	8.597474	1.18E-16	3.74E-15	26.98638
**CASP4**	0.591975	1.777904	8.591823	1.24E-16	3.88E-15	26.94448
**HELZ2**	0.655697	2.831392	8.561089	1.56E-16	4.83E-15	26.71698

**PIM1**	0.612689	4.360932	8.550448	1.69E-16	5.20E-15	26.63835
**AC015540.1**	-0.82522	3.137744	-8.54757	1.72E-16	5.31E-15	26.61713
**CNN3**	0.63747	8.597016	8.547267	1.73E-16	5.32E-15	26.61486
**HOXC4**	0.846038	1.381108	8.544388	1.76E-16	5.43E-15	26.59361
**PODXL**	0.505455	5.558435	8.544298	1.77E-16	5.43E-15	26.59294
**HAS2**	0.874973	2.453512	8.535623	1.88E-16	5.75E-15	26.52893
**SYNE2**	0.576803	3.331708	8.53418	1.91E-16	5.81E-15	26.51828
**PIF1**	0.630829	1.463128	8.531419	1.94E-16	5.93E-15	26.49793
**CRNDE**	1.033922	1.865574	8.530534	1.96E-16	5.96E-15	26.4914
**KLHDC8A**	0.984639	4.764474	8.526773	2.01E-16	6.12E-15	26.46367
**AGRN**	0.537619	5.940864	8.526398	2.02E-16	6.13E-15	26.46091
**EIF4EBP1**	0.637777	5.754211	8.518423	2.14E-16	6.48E-15	26.40214
**MBNL3**	0.504913	1.252422	8.515091	2.20E-16	6.62E-15	26.37761
**PLAT**	0.987251	3.124572	8.500724	2.45E-16	7.32E-15	26.27188
**MIR4435-2HG**	0.641045	1.195232	8.497916	2.50E-16	7.46E-15	26.25124
**MIR3153**	0.579937	0.658353	8.483042	2.79E-16	8.27E-15	26.14194
**STAT1**	0.696472	5.881738	8.472586	3.02E-16	8.89E-15	26.0652
**NFE2L3**	0.532334	2.191293	8.470821	3.06E-16	9.00E-15	26.05225
**MT-RNR1**	-0.58295	12.94464	-8.46876	3.11E-16	9.13E-15	26.03713
**TCIM**	1.043405	4.065146	8.460706	3.30E-16	9.66E-15	25.97809
**LAP3**	0.537309	6.258886	8.455376	3.43E-16	1.00E-14	25.93903
**ZNF649**	0.520345	3.762488	8.452795	3.50E-16	1.02E-14	25.92013
**TUBB6**	0.867805	3.666378	8.431105	4.11E-16	1.18E-14	25.76143
**BTN2A2**	0.545992	2.599529	8.429207	4.17E-16	1.20E-14	25.74756
**ETNPPL**	-1.38728	6.574444	-8.42749	4.23E-16	1.21E-14	25.73502
**SOX11**	0.900625	4.173683	8.41881	4.51E-16	1.29E-14	25.6716
**BGN**	0.897058	6.503714	8.41753	4.55E-16	1.30E-14	25.66226
**SNRPGP10**	0.737267	2.268941	8.415256	4.63E-16	1.32E-14	25.64565
**TNFRSF12A**	1.183214	3.715604	8.407963	4.88E-16	1.39E-14	25.59243
**DPY19L1**	0.635152	5.007357	8.403529	5.05E-16	1.43E-14	25.5601
**ITPRIPL1**	0.702265	1.718131	8.403508	5.05E-16	1.43E-14	25.55994
**MYD88**	0.703677	3.949349	8.402911	5.07E-16	1.44E-14	25.55559
**NRP1**	0.571041	3.221988	8.398863	5.23E-16	1.48E-14	25.52608
**DDX60L**	0.536229	2.005822	8.388892	5.63E-16	1.59E-14	25.45343
**SNAI2**	0.714948	2.020504	8.385758	5.76E-16	1.62E-14	25.43061
**PLEKHA4**	1.047409	4.240709	8.376196	6.18E-16	1.73E-14	25.36101
**GNG5**	0.696071	6.620684	8.375697	6.20E-16	1.74E-14	25.35738
**RAVER1**	0.528934	4.41308	8.370917	6.43E-16	1.80E-14	25.32262
**CLIC1**	0.814733	5.867621	8.369666	6.49E-16	1.81E-14	25.31353
**GPR82**	0.634172	0.811445	8.361402	6.90E-16	1.92E-14	25.25347
**ST14**	0.84734	2.179745	8.360846	6.92E-16	1.92E-14	25.24943
**TNFRSF11B**	0.838414	1.25776	8.353471	7.31E-16	2.03E-14	25.19587
**ANXA2R**	0.55774	2.070714	8.348898	7.56E-16	2.09E-14	25.16267

**FOXD3-AS1**	1.044915	1.730808	8.348115	7.61E-16	2.10E-14	25.15699
**ALDOC**	-0.824	9.933016	-8.34141	7.99E-16	2.20E-14	25.10833
**KLF6**	0.570074	5.119543	8.33919	8.13E-16	2.23E-14	25.09225
**ATF7IP**	0.510091	5.010553	8.338516	8.17E-16	2.24E-14	25.08736
**RUNX1**	0.728115	1.738724	8.328741	8.78E-16	2.40E-14	25.01653
**ANGPT2**	0.882649	2.281965	8.302373	1.07E-15	2.88E-14	24.82574
**H2BC9**	0.511756	0.428025	8.299724	1.09E-15	2.93E-14	24.8066
**RBMS1**	0.534769	2.529808	8.297905	1.10E-15	2.96E-14	24.79346
**IQGAP1**	0.687683	4.211903	8.280544	1.25E-15	3.34E-14	24.66814
**SIPA1L2**	0.51309	4.941777	8.271479	1.34E-15	3.56E-14	24.60278
**TSSK5P**	0.610168	2.847668	8.268876	1.36E-15	3.62E-14	24.58403
**EEF1AKMT3**	0.651934	3.064262	8.267356	1.38E-15	3.66E-14	24.57308
**AC112777.1**	0.603644	1.774918	8.26274	1.43E-15	3.77E-14	24.53983
**MCUB**	0.796189	2.484559	8.262387	1.43E-15	3.78E-14	24.53728
**PTPRZ1**	0.768678	9.027271	8.258678	1.47E-15	3.87E-14	24.51058
**NT5DC2**	0.514427	5.204717	8.256782	1.49E-15	3.92E-14	24.49694
**ADGRL4**	0.6044	3.483648	8.242196	1.66E-15	4.34E-14	24.39202
**SUSD1**	0.556329	3.255505	8.23031	1.81E-15	4.72E-14	24.30664
**SEPTIN10**	0.515737	4.469935	8.221668	1.93E-15	5.01E-14	24.24461
**THBS3**	0.534534	3.798345	8.220751	1.94E-15	5.04E-14	24.23804
**NEDD9**	0.596393	3.470641	8.220284	1.94E-15	5.05E-14	24.23469
**AC015967.1**	-0.50817	1.773471	-8.21749	1.98E-15	5.15E-14	24.21464
**CLEC18B**	0.618416	1.206936	8.21188	2.07E-15	5.35E-14	24.17442
**SEC24D**	0.511872	2.35137	8.206912	2.14E-15	5.53E-14	24.13882
**SMAD1**	0.514911	4.441365	8.199175	2.27E-15	5.84E-14	24.08341
**NES**	0.832564	8.163706	8.180187	2.60E-15	6.66E-14	23.94758
**FAM20C**	0.605227	5.258398	8.178326	2.64E-15	6.74E-14	23.93428
**CYTOR**	0.656299	1.235258	8.172553	2.75E-15	7.01E-14	23.89304
**REV3L**	0.53798	4.189137	8.166891	2.87E-15	7.28E-14	23.85261
**COL6A2**	1.011951	3.579202	8.165154	2.91E-15	7.37E-14	23.84021
**MSN**	0.840273	6.114863	8.164466	2.92E-15	7.39E-14	23.8353
**NIBAN1**	0.791731	2.36081	8.153472	3.16E-15	7.98E-14	23.75688
**HPSE2**	-1.2613	3.5224	-8.14671	3.32E-15	8.35E-14	23.70868
**MMP25**	0.558498	1.637032	8.145352	3.35E-15	8.43E-14	23.69901
**EN1**	0.893471	0.787804	8.14393	3.39E-15	8.51E-14	23.68888
**ZC3HAV1L**	0.597797	1.315215	8.134371	3.63E-15	9.07E-14	23.62082
**AFAP1L1**	0.637322	2.667329	8.133291	3.66E-15	9.13E-14	23.61313
**CELSR1**	0.645616	0.966652	8.131406	3.71E-15	9.25E-14	23.59972
**HMGN2P5**	0.564857	2.462366	8.122855	3.95E-15	9.82E-14	23.5389
**GRIK3**	0.74755	5.381561	8.116197	4.14E-15	1.03E-13	23.49159
**ARC**	1.106088	5.013251	8.110919	4.30E-15	1.07E-13	23.45409
**PTGDS**	-0.95935	9.226872	-8.11005	4.33E-15	1.07E-13	23.44791
**PPP1R3B**	0.556492	2.617406	8.109394	4.35E-15	1.08E-13	23.44327
**ITGA4**	0.51785	1.068847	8.106653	4.44E-15	1.10E-13	23.42381

**PPP1R1A**	-0.8938	3.88481	-8.10607	4.46E-15	1.10E-13	23.41968
**TRIM14**	0.661072	4.039255	8.104298	4.52E-15	1.11E-13	23.40709
**LAMA2**	0.699317	2.624251	8.100497	4.64E-15	1.14E-13	23.38012
**NLGN4X**	0.598846	4.553903	8.088677	5.06E-15	1.24E-13	23.29631
**H2AC11**	0.522902	0.974113	8.081612	5.32E-15	1.30E-13	23.24626
**LCN12**	-0.58486	1.833173	-8.0765	5.52E-15	1.34E-13	23.21006
**PLA2G4A**	0.667113	3.018167	8.076039	5.54E-15	1.35E-13	23.2068
**ADPRH**	0.506114	2.24536	8.070665	5.76E-15	1.40E-13	23.16877
**ILDR2**	0.615347	3.802053	8.067985	5.87E-15	1.42E-13	23.14981
**TEAD2**	0.655039	3.015714	8.064892	6.00E-15	1.45E-13	23.12794
**MFAP2**	0.725142	1.034503	8.05905	6.26E-15	1.51E-13	23.08664
**IFI44**	0.735216	4.648992	8.049419	6.71E-15	1.61E-13	23.01861
**NAMPT**	0.694128	4.456867	8.03647	7.36E-15	1.76E-13	22.92724
**MOV10**	0.520914	3.891075	8.034855	7.45E-15	1.78E-13	22.91585
**CYS1**	-0.62737	2.886283	-8.03165	7.62E-15	1.82E-13	22.89322
**ADHFE1**	-0.53401	4.501171	-8.02764	7.84E-15	1.87E-13	22.86498
**NEK6**	0.631365	5.11183	8.024937	8.00E-15	1.90E-13	22.84595
**CTSC**	0.683304	3.079417	8.022084	8.16E-15	1.94E-13	22.82586
**HOXD9**	1.00769	0.98933	8.021517	8.20E-15	1.95E-13	22.82187
**HOXA5**	1.006317	0.731893	8.014288	8.63E-15	2.04E-13	22.77098
**RNU6-529P**	-0.76685	5.861358	-8.01208	8.77E-15	2.07E-13	22.75547
**GNB4**	0.525414	4.942769	8.009625	8.92E-15	2.11E-13	22.73817
**TNC**	1.012262	5.808766	7.990935	1.02E-14	2.39E-13	22.60682
**SYDE1**	0.531575	3.905125	7.988839	1.04E-14	2.43E-13	22.59211
**LOXL3**	0.586907	3.651295	7.981524	1.09E-14	2.54E-13	22.54077
**DGCR6**	-0.77052	3.606755	-7.97695	1.13E-14	2.62E-13	22.50869
**SAMD9L**	0.784988	3.106961	7.974861	1.14E-14	2.66E-13	22.49404
**H3P6**	0.545415	3.958916	7.967145	1.21E-14	2.80E-13	22.43997
**ANXA1**	1.298051	4.41343	7.966558	1.21E-14	2.80E-13	22.43585
**SP100**	0.576412	2.148397	7.966455	1.22E-14	2.81E-13	22.43513
**LOX**	0.644785	1.522183	7.961595	1.26E-14	2.89E-13	22.40109
**TENT5A**	0.640568	2.684452	7.954843	1.32E-14	3.02E-13	22.35384
**EHD4**	0.560546	3.238804	7.947218	1.39E-14	3.17E-13	22.3005
**COL5A1**	0.813054	1.499718	7.935178	1.52E-14	3.44E-13	22.21636
**AC064875.1**	0.762317	0.839569	7.930606	1.57E-14	3.54E-13	22.18444
**LINC01778**	0.523624	1.999462	7.916867	1.73E-14	3.89E-13	22.08859
**TFPI**	0.84656	1.817988	7.906112	1.87E-14	4.17E-13	22.01364
**MEX3A**	0.750085	4.558144	7.899505	1.96E-14	4.36E-13	21.96764
**HOXA7**	0.725619	0.491356	7.885751	2.16E-14	4.78E-13	21.87198
**TAGLN2**	0.788635	6.341035	7.882677	2.20E-14	4.88E-13	21.85062
**CRLF1**	-1.32054	4.353198	-7.87859	2.27E-14	5.02E-13	21.82224
**MCAM**	0.643703	4.555218	7.865788	2.48E-14	5.46E-13	21.73336
**MFAP3**	0.578074	3.224157	7.863504	2.52E-14	5.54E-13	21.71751
**MYL3**	-0.63345	2.710543	-7.86224	2.55E-14	5.58E-13	21.70876

**ADGRE2**	0.502915	0.986747	7.854031	2.70E-14	5.89E-13	21.65184
**TMEM71**	0.591351	1.015045	7.848868	2.80E-14	6.10E-13	21.61607
**GPX8**	0.740537	1.115361	7.848106	2.81E-14	6.13E-13	21.61079
**CROT**	0.505941	3.954054	7.844484	2.89E-14	6.26E-13	21.58572
**GRIN2C**	-0.69203	3.088455	-7.84383	2.90E-14	6.29E-13	21.58121
**RNA5SP82**	0.610928	1.556727	7.83414	3.11E-14	6.70E-13	21.51414
**MT-ND6**	-0.54178	13.43817	-7.83189	3.16E-14	6.79E-13	21.49857
**EMILIN1**	0.654463	4.410591	7.824593	3.32E-14	7.12E-13	21.44815
**RND3**	0.730884	4.229833	7.817235	3.50E-14	7.47E-13	21.39733
**GABRD**	-1.1383	4.798308	-7.81256	3.62E-14	7.70E-13	21.36504
**ADCY7**	0.543354	2.388632	7.804803	3.82E-14	8.10E-13	21.31155
**NID2**	0.640607	1.861898	7.799146	3.97E-14	8.41E-13	21.27255
**NSUN7**	0.534174	0.70158	7.798767	3.98E-14	8.43E-13	21.26994
**CEMIP2**	0.519174	2.943147	7.793387	4.14E-14	8.72E-13	21.23287
**RAB39A**	0.588102	2.437139	7.788353	4.29E-14	9.02E-13	21.19821
**MS4A6A**	0.961596	3.395942	7.78556	4.37E-14	9.19E-13	21.17898
**FZD2**	0.625819	2.537244	7.777948	4.61E-14	9.66E-13	21.12661
**TPST1**	0.512769	5.752938	7.768104	4.94E-14	1.03E-12	21.05895
**APOBEC3C**	0.716106	3.632842	7.764126	5.08E-14	1.06E-12	21.03162
**CYP27B1**	0.606833	0.842117	7.762319	5.14E-14	1.07E-12	21.01921
**LDLRAD3**	0.534511	5.799715	7.759423	5.25E-14	1.09E-12	20.99932
**EGFR**	1.060245	6.499868	7.745271	5.80E-14	1.19E-12	20.90224
**NPNT**	1.037118	2.616432	7.734168	6.26E-14	1.28E-12	20.82617
**CHTF18**	0.575737	3.194022	7.723898	6.73E-14	1.37E-12	20.75589
**CYFIP1**	0.526969	5.519419	7.72216	6.81E-14	1.39E-12	20.744
**CFH**	0.643362	2.297503	7.719417	6.94E-14	1.41E-12	20.72524
**LUM**	0.801815	1.896734	7.71679	7.07E-14	1.43E-12	20.70728
**PROS1**	0.662325	4.238369	7.711996	7.31E-14	1.48E-12	20.67452
**NOTCH3**	0.535439	4.65792	7.703779	7.74E-14	1.56E-12	20.6184
**KIAA0040**	0.819624	2.917751	7.701493	7.86E-14	1.59E-12	20.6028
**WSCD1**	0.714477	5.606061	7.701053	7.89E-14	1.59E-12	20.59979
**LAMC3**	0.580167	2.35467	7.699331	7.98E-14	1.61E-12	20.58804
**PYGL**	0.712475	3.859163	7.682326	8.99E-14	1.79E-12	20.47211
**CPE**	-0.62122	10.42988	-7.68142	9.04E-14	1.80E-12	20.46597
**ANO6**	0.584552	4.589969	7.679158	9.19E-14	1.83E-12	20.45053
**AL049871.1**	0.533318	0.629386	7.678652	9.22E-14	1.84E-12	20.44709
**CHI3L2**	1.353665	3.834745	7.666983	1.00E-13	1.98E-12	20.36768
**AASS**	0.593991	4.259575	7.666963	1.00E-13	1.98E-12	20.36755
**NFAM1**	0.588321	2.416641	7.662615	1.03E-13	2.04E-12	20.33798
**ADAMTS7**	0.548217	1.157024	7.660799	1.04E-13	2.06E-12	20.32563
**CD151**	0.519296	6.000616	7.657662	1.07E-13	2.10E-12	20.30431
**TIMP1**	1.261703	5.13141	7.654733	1.09E-13	2.14E-12	20.28442
**LHFPL2**	0.596839	4.119875	7.638047	1.22E-13	2.39E-12	20.17117
**LXN**	0.567611	2.397856	7.63645	1.23E-13	2.41E-12	20.16034

**F11R**	0.527269	2.595659	7.635694	1.24E-13	2.42E-12	20.15521
**PLBD1**	0.617155	1.901187	7.635056	1.25E-13	2.43E-12	20.15089
**MTMR11**	0.568538	3.07335	7.635001	1.25E-13	2.43E-12	20.15052
**TPD52L1**	-0.72693	3.220105	-7.63397	1.26E-13	2.45E-12	20.14353
**DDX11**	0.576934	2.797071	7.626378	1.32E-13	2.57E-12	20.09209
**DOK3**	0.535659	2.452607	7.621797	1.37E-13	2.65E-12	20.06107
**SEC61G**	0.776782	5.845392	7.60889	1.49E-13	2.88E-12	19.97374
**METTL1**	0.594095	3.787699	7.608711	1.50E-13	2.88E-12	19.97253
**FBXO2**	-0.90061	5.965213	-7.60719	1.51E-13	2.91E-12	19.96225
**FST**	0.5805	1.506319	7.606952	1.51E-13	2.92E-12	19.96064
**LHPP**	-0.54661	5.723015	-7.60602	1.52E-13	2.93E-12	19.95433
**LINC02716**	-0.63376	2.848597	-7.60355	1.55E-13	2.98E-12	19.93765
**C1orf226**	0.630931	4.174158	7.603103	1.55E-13	2.99E-12	19.93463
**IRX5**	0.596352	0.731293	7.590139	1.70E-13	3.25E-12	19.8471
**TRIM5**	0.584756	2.999682	7.578064	1.85E-13	3.52E-12	19.76567
**MMP14**	0.848011	5.13582	7.575372	1.88E-13	3.58E-12	19.74753
**TCEAL2**	-0.53803	7.519206	-7.5747	1.89E-13	3.60E-12	19.74298
**HIP1**	0.549159	5.832806	7.567903	1.98E-13	3.76E-12	19.69722
**RUNX3**	0.529101	1.505358	7.558986	2.11E-13	3.99E-12	19.63722
**S1PR3**	0.821681	3.477774	7.556075	2.15E-13	4.06E-12	19.61764
**FOXD3**	0.685433	0.896292	7.555727	2.15E-13	4.07E-12	19.61529
**HOXA6**	0.603607	0.370708	7.548669	2.26E-13	4.26E-12	19.56786
**COL14A1**	0.767885	1.806846	7.542015	2.37E-13	4.45E-12	19.52316
**GPR65**	0.673033	1.439722	7.53411	2.50E-13	4.68E-12	19.47011
**BCL3**	0.509365	2.768499	7.530759	2.56E-13	4.77E-12	19.44763
**IGFBP4**	0.706648	5.684422	7.521969	2.71E-13	5.06E-12	19.38871
**DNMBP-AS1**	-0.65771	1.45502	-7.5192	2.77E-13	5.15E-12	19.37013
**REST**	0.544432	3.007477	7.505269	3.04E-13	5.63E-12	19.27692
**OAS2**	0.772387	2.845675	7.503703	3.07E-13	5.68E-12	19.26644
**CXCL9**	0.721939	0.962416	7.478128	3.66E-13	6.68E-12	19.09564
**OLFML2A**	0.558223	2.350513	7.477515	3.67E-13	6.71E-12	19.09155
**AL031710.1**	-0.57886	1.037351	-7.47451	3.75E-13	6.84E-12	19.07149
**SLC43A3**	0.732985	2.486289	7.469548	3.88E-13	7.05E-12	19.03843
**COL18A1**	0.571109	4.224764	7.465578	3.99E-13	7.23E-12	19.01199
**OAS3**	0.784203	3.531148	7.463274	4.05E-13	7.33E-12	18.99665
**ADGRE5**	0.6482	3.356041	7.455682	4.26E-13	7.70E-12	18.94611
**OLFML3**	0.708275	5.125341	7.454598	4.29E-13	7.76E-12	18.9389
**YBX3**	0.556388	3.900431	7.454193	4.31E-13	7.78E-12	18.9362
**LSP1**	0.762391	2.157995	7.450128	4.43E-13	7.97E-12	18.90917
**VCL**	0.549694	4.279817	7.449752	4.44E-13	7.98E-12	18.90666
**PLAUR**	0.642688	2.171583	7.447944	4.49E-13	8.08E-12	18.89464
**ELK3**	0.547257	4.051212	7.438668	4.78E-13	8.57E-12	18.833
**B2M**	0.554265	9.68731	7.437058	4.84E-13	8.65E-12	18.82231
**TMSB4X**	0.505835	10.30134	7.434711	4.91E-13	8.78E-12	18.80673

**NAPSB**	1.145612	3.712827	7.429168	5.10E-13	9.10E-12	18.76994
**IGF2BP2**	0.777646	1.000981	7.422264	5.35E-13	9.51E-12	18.72415
**LATS2**	0.540862	2.18281	7.408871	5.85E-13	1.04E-11	18.63541
**EPSTI1**	0.633479	1.991575	7.406499	5.95E-13	1.05E-11	18.61971
**CA3**	0.935169	1.57169	7.403268	6.08E-13	1.07E-11	18.59833
**FCGBP**	1.195054	3.633422	7.402631	6.11E-13	1.08E-11	18.59412
**MYOF**	0.658608	3.081192	7.379811	7.12E-13	1.25E-11	18.44332
**PTGER4**	0.54106	1.383646	7.364299	7.91E-13	1.37E-11	18.34103
**AL391834.1**	-0.60497	3.501262	-7.35078	8.66E-13	1.49E-11	18.25201
**LCNL1**	-0.95393	3.173715	-7.34055	9.27E-13	1.59E-11	18.18472
**ATP6V1G2**	-0.67167	7.211446	-7.33849	9.40E-13	1.61E-11	18.17119
**SEMA5B**	0.658478	4.374555	7.331639	9.85E-13	1.68E-11	18.1262
**STEAP3**	0.913957	3.119714	7.33036	9.93E-13	1.70E-11	18.1178
**TNFRSF19**	0.765459	3.729485	7.326904	1.02E-12	1.73E-11	18.09512
**SCIMP**	0.529275	1.696929	7.324211	1.03E-12	1.76E-11	18.07745
**DUSP10**	0.597663	3.005613	7.311624	1.13E-12	1.91E-11	17.99493
**USP18**	0.538154	2.973806	7.307894	1.15E-12	1.95E-11	17.9705
**CYP46A1**	-0.55186	3.410572	-7.30527	1.17E-12	1.99E-11	17.95334
**FRMD3**	0.590034	3.373917	7.304115	1.18E-12	2.00E-11	17.94575
**AC027644.1**	0.508848	1.814383	7.303621	1.19E-12	2.01E-11	17.94252
**ZNF311**	0.546368	2.274552	7.301399	1.21E-12	2.03E-11	17.92798
**AC022432.1**	0.537748	1.27446	7.294245	1.26E-12	2.13E-11	17.88118
**CD58**	0.642168	2.953826	7.283442	1.36E-12	2.27E-11	17.81058
**PTP4A3**	0.502321	4.964352	7.283268	1.36E-12	2.27E-11	17.80944
**ACE**	0.591183	1.706208	7.274545	1.44E-12	2.41E-11	17.7525
**AC010273.3**	0.561682	1.24784	7.273995	1.45E-12	2.41E-11	17.74891
**ADAMTS15**	0.859062	2.438625	7.270361	1.48E-12	2.47E-11	17.7252
**RNU6-850P**	0.665189	2.538931	7.266886	1.52E-12	2.52E-11	17.70254
**AL354863.1**	-0.63267	1.208993	-7.2649	1.54E-12	2.55E-11	17.68957
**LIMD1**	0.575077	2.840524	7.264141	1.55E-12	2.56E-11	17.68465
**BRINP1**	-0.90603	5.027764	-7.26265	1.56E-12	2.59E-11	17.67495
**AC025171.5**	0.515424	0.913419	7.261054	1.58E-12	2.61E-11	17.66453
**VAV3**	0.755374	1.197098	7.257065	1.62E-12	2.68E-11	17.63855
**SRPX2**	0.862482	2.214176	7.250137	1.70E-12	2.79E-11	17.59345
**NAP1L2**	-0.7342	5.125218	-7.24926	1.71E-12	2.81E-11	17.58774
**ANXA2P2**	0.608893	1.262584	7.237885	1.84E-12	3.01E-11	17.51377
**PDPN**	1.237457	3.571739	7.219269	2.08E-12	3.38E-11	17.39292
**SYTL3**	0.509966	2.302507	7.217756	2.10E-12	3.41E-11	17.38311
**CXCL10**	1.092334	1.8355	7.208738	2.23E-12	3.60E-11	17.32466
**IFI16**	0.561169	5.159343	7.202143	2.33E-12	3.75E-11	17.28196
**AC010332.1**	0.540461	4.634958	7.200216	2.36E-12	3.79E-11	17.26948
**AC079946.1**	-0.59267	1.462182	-7.1996	2.37E-12	3.81E-11	17.26549
**BCYRN1**	-0.84022	2.318353	-7.19036	2.52E-12	4.03E-11	17.20575
**MYO1B**	0.564354	2.707698	7.189406	2.54E-12	4.05E-11	17.19957

**NMI**	0.538265	2.833636	7.186641	2.58E-12	4.12E-11	17.1817
**ERFE**	0.537188	1.416518	7.18599	2.60E-12	4.14E-11	17.17749
**LINC01831**	0.507367	0.761077	7.174567	2.80E-12	4.44E-11	17.10373
**PDLIM1**	0.796716	2.975107	7.173392	2.82E-12	4.47E-11	17.09615
**CETP**	0.553454	1.893449	7.16986	2.89E-12	4.56E-11	17.07336
**TFRC**	0.575327	4.53302	7.167462	2.93E-12	4.63E-11	17.0579
**SCD**	-0.62682	9.499706	-7.16289	3.02E-12	4.77E-11	17.0284
**AL731533.2**	-0.50694	2.931687	-7.16238	3.03E-12	4.78E-11	17.02513
**B3GNT5**	0.575564	1.799159	7.159813	3.08E-12	4.86E-11	17.0086
**NETO2**	0.696272	3.364656	7.158477	3.11E-12	4.90E-11	17
**NLRC5**	0.525162	2.486734	7.154647	3.19E-12	5.01E-11	16.97533
**TGFBR2**	0.516838	4.852704	7.154299	3.20E-12	5.02E-11	16.97309
**NEXN**	0.528152	2.00468	7.154094	3.20E-12	5.03E-11	16.97177
**RHOJ**	0.565423	3.654066	7.154037	3.20E-12	5.03E-11	16.9714
**LIF**	0.743974	1.160317	7.152846	3.23E-12	5.06E-11	16.96373
**EPB41**	0.537175	3.589749	7.152499	3.24E-12	5.07E-11	16.9615
**VN1R81P**	0.504684	3.022123	7.138878	3.54E-12	5.53E-11	16.87389
**HAND2**	0.590751	0.571387	7.13544	3.62E-12	5.65E-11	16.8518
**SINHCAF**	0.585669	3.407478	7.133977	3.66E-12	5.70E-11	16.8424
**TYMP**	0.87873	3.084205	7.129396	3.77E-12	5.85E-11	16.81298
**BCAT1**	0.785267	2.595445	7.128824	3.78E-12	5.87E-11	16.80931
**RHPN2**	0.644603	3.97939	7.126658	3.83E-12	5.95E-11	16.7954
**A2M**	0.523586	8.117309	7.115268	4.13E-12	6.37E-11	16.72234
**ELF4**	0.532274	2.001545	7.113152	4.19E-12	6.45E-11	16.70879
**PNMA8B**	-0.5847	4.066198	-7.10817	4.33E-12	6.65E-11	16.67686
**TEAD4**	0.713235	2.203098	7.10816	4.33E-12	6.65E-11	16.6768
**TAP1**	0.547908	5.311425	7.107891	4.34E-12	6.66E-11	16.67508
**FAIM2**	-0.58922	7.615607	-7.10583	4.40E-12	6.75E-11	16.66186
**BCL2L12**	0.500164	2.355691	7.100733	4.54E-12	6.95E-11	16.62925
**FAM20A**	0.510564	1.143957	7.09967	4.58E-12	6.99E-11	16.62245
**RASL10A**	-0.89043	4.974262	-7.09863	4.61E-12	7.04E-11	16.61582
**OSMR**	0.695442	2.944291	7.096898	4.66E-12	7.11E-11	16.60472
**HAPLN3**	0.526997	2.211201	7.095607	4.70E-12	7.17E-11	16.59646
**AC103923.1**	0.522298	1.592327	7.093451	4.77E-12	7.26E-11	16.58267
**NAMPTP1**	0.631101	1.617167	7.080976	5.17E-12	7.83E-11	16.50296
**CPVL**	0.740113	4.229588	7.078637	5.25E-12	7.94E-11	16.48803
**SNORA5C**	0.51514	2.659796	7.074743	5.39E-12	8.14E-11	16.46317
**HOXA10**	0.78095	0.70459	7.074036	5.41E-12	8.18E-11	16.45866
**FOXD1**	0.822836	2.71331	7.057048	6.04E-12	9.07E-11	16.35039
**NEDD4**	0.522572	2.666655	7.048583	6.39E-12	9.55E-11	16.29651
**GTF2I**	0.521083	4.343457	7.047351	6.44E-12	9.62E-11	16.28867
**PCDHB10**	0.520102	4.01151	7.041397	6.69E-12	9.97E-11	16.25082
**LRRC17**	0.765622	3.546479	7.037333	6.87E-12	1.02E-10	16.22499
**DPYD**	0.642205	2.458752	7.036699	6.90E-12	1.03E-10	16.22096

**PLAC8**	0.514887	0.835576	7.0316	7.13E-12	1.06E-10	16.18858
**HOXD10**	0.697533	0.54187	7.02639	7.38E-12	1.09E-10	16.15551
**ENHO**	-0.68	8.558421	-7.0245	7.47E-12	1.10E-10	16.14355
**HGF**	0.569214	1.227988	7.024162	7.49E-12	1.11E-10	16.14137
**ADARB2**	-0.77744	3.123375	-7.02255	7.56E-12	1.12E-10	16.13117
**CACNA2D3**	-0.73573	2.592313	-7.01246	8.07E-12	1.19E-10	16.06722
**SECTM1**	0.680946	1.840872	6.996832	8.93E-12	1.31E-10	15.96827
**MUC1**	0.508865	2.318378	6.990435	9.31E-12	1.36E-10	15.92784
**ITGA2**	0.632446	2.263836	6.983408	9.74E-12	1.42E-10	15.88346
**HLF**	-0.53889	4.473885	-6.97398	1.04E-11	1.50E-10	15.82398
**ANXA2**	0.797513	4.052747	6.971288	1.05E-11	1.52E-10	15.80698
**SNCG**	-0.99321	5.251109	-6.96328	1.11E-11	1.60E-10	15.75652
**LYN**	0.537973	3.651442	6.958574	1.14E-11	1.64E-10	15.72689
**LYZ**	1.003373	3.059093	6.955646	1.17E-11	1.67E-10	15.70846
**HMGCLL1**	-0.65004	2.056915	-6.95479	1.17E-11	1.68E-10	15.70309
**GABRG1**	-0.95204	3.624659	-6.9508	1.20E-11	1.72E-10	15.678
**KDELR3**	0.501692	1.531569	6.949986	1.21E-11	1.73E-10	15.67285
**SLC7A7**	0.569935	3.046834	6.945119	1.25E-11	1.78E-10	15.64225
**STK32B**	0.715303	2.232163	6.944866	1.25E-11	1.78E-10	15.64066
**ACTN1**	0.741452	4.416941	6.938263	1.30E-11	1.85E-10	15.59918
**MAP1LC3C**	0.694172	0.843333	6.938176	1.30E-11	1.86E-10	15.59863
**PTPN13**	0.519081	4.482647	6.93589	1.32E-11	1.88E-10	15.58427
**ADGRE1**	0.526014	0.71276	6.929517	1.38E-11	1.95E-10	15.54427
**ERV3-1**	0.505241	3.152083	6.910092	1.56E-11	2.19E-10	15.42254
**GIMAP2**	0.513691	3.229297	6.908691	1.58E-11	2.21E-10	15.41377
**PPP4R4**	-0.76738	2.464906	-6.90864	1.58E-11	2.21E-10	15.41342
**CARD16**	0.616782	1.865567	6.899747	1.67E-11	2.34E-10	15.35782
**MIR3682**	-0.55375	7.593545	-6.89521	1.72E-11	2.40E-10	15.3295
**PABPC1L**	0.672528	2.85166	6.892339	1.75E-11	2.44E-10	15.31153
**HPCAL4**	-1.00089	5.135069	-6.88194	1.87E-11	2.60E-10	15.24664
**OTP**	0.508597	0.316043	6.881877	1.87E-11	2.60E-10	15.24622
**HOXD13**	0.653117	0.4016	6.881203	1.88E-11	2.61E-10	15.24201
**TRAF4**	0.529186	5.539872	6.88055	1.89E-11	2.62E-10	15.23794
**LINC00634**	-0.54194	5.769415	-6.87948	1.90E-11	2.64E-10	15.23126
**SERPINE1**	1.131811	3.683263	6.879365	1.90E-11	2.64E-10	15.23055
**ZBTB42**	0.523925	1.973222	6.879263	1.90E-11	2.64E-10	15.22991
**AL357500.2**	0.505584	1.775256	6.875437	1.95E-11	2.70E-10	15.20606
**MIR3176**	0.630538	2.089692	6.874955	1.95E-11	2.71E-10	15.20305
**RBM47**	0.528667	1.699445	6.867353	2.05E-11	2.83E-10	15.15569
**TGFBI**	0.893551	3.423529	6.84929	2.30E-11	3.16E-10	15.04331
**ADGRL2**	0.633421	2.982752	6.845184	2.36E-11	3.23E-10	15.0178
**HOXD11**	0.511237	0.316366	6.842829	2.40E-11	3.28E-10	15.00317
**FOSL2**	0.597874	4.281983	6.836516	2.50E-11	3.40E-10	14.96399
**S100A11**	0.766479	6.872417	6.836368	2.50E-11	3.40E-10	14.96307

**HOXD4**	0.517913	0.707283	6.833744	2.54E-11	3.46E-10	14.94679
**FYB1**	0.67833	3.448408	6.833652	2.54E-11	3.46E-10	14.94622
**AC008760.2**	0.629447	1.174504	6.832557	2.56E-11	3.48E-10	14.93943
**SWAP70**	0.505193	3.586007	6.832507	2.56E-11	3.48E-10	14.93912
**FMOD**	1.0387	2.349088	6.832493	2.56E-11	3.48E-10	14.93903
**ADAMTS6**	0.500832	2.163008	6.827174	2.65E-11	3.59E-10	14.90605
**AC026790.2**	-0.5596	1.203788	-6.82413	2.70E-11	3.66E-10	14.88717
**FBN3**	0.593506	1.150821	6.822185	2.73E-11	3.70E-10	14.87514
**MGP**	1.08639	4.239912	6.819427	2.78E-11	3.76E-10	14.85806
**LAMA4**	0.56387	3.485331	6.818276	2.80E-11	3.79E-10	14.85093
**EMILIN2**	0.666048	1.742383	6.815748	2.85E-11	3.85E-10	14.83528
**H2BC12**	0.591796	4.44261	6.814861	2.86E-11	3.86E-10	14.82979
**AC002456.1**	0.549993	2.512223	6.81265	2.91E-11	3.91E-10	14.81611
**PDCD1LG2**	0.582358	1.551543	6.804071	3.07E-11	4.12E-10	14.76306
**GPC2**	0.600849	3.881886	6.797713	3.19E-11	4.27E-10	14.72377
**HOXC10**	0.730931	0.49417	6.797452	3.20E-11	4.28E-10	14.72216
**COL8A1**	0.733725	0.976426	6.792297	3.30E-11	4.42E-10	14.69034
**ECSCR**	0.5471	2.274969	6.791182	3.33E-11	4.44E-10	14.68345
**PTCRA**	0.50443	0.836596	6.785377	3.45E-11	4.60E-10	14.64764
**MATN2**	0.674234	5.374021	6.784464	3.47E-11	4.62E-10	14.64201
**CHST1**	-0.72359	5.465782	-6.77489	3.69E-11	4.90E-10	14.58299
**RNF135**	0.511959	3.012784	6.770628	3.79E-11	5.03E-10	14.55677
**EPHA2**	0.583553	2.474595	6.76045	4.04E-11	5.34E-10	14.49416
**TLR1**	0.575471	2.166833	6.757971	4.10E-11	5.42E-10	14.47891
**SPX**	-0.93345	3.860313	-6.75085	4.29E-11	5.66E-10	14.43518
**DCHS1**	0.5716	3.968651	6.750114	4.31E-11	5.68E-10	14.43064
**VEGFA**	0.638267	3.217954	6.749778	4.32E-11	5.69E-10	14.42858
**AC125616.1**	-0.63252	1.247207	-6.74877	4.35E-11	5.72E-10	14.42236
**FPR3**	0.776583	2.163526	6.748586	4.35E-11	5.73E-10	14.42126
**BMF**	0.555929	2.773019	6.737604	4.66E-11	6.11E-10	14.35389
**SLC25A48**	-0.86687	3.621057	-6.73751	4.67E-11	6.11E-10	14.35329
**ARHGAP18**	0.531408	2.915372	6.737374	4.67E-11	6.11E-10	14.35248
**CCND1**	0.691888	5.947241	6.733753	4.78E-11	6.25E-10	14.33028
**IRF1**	0.533033	2.779637	6.725561	5.03E-11	6.56E-10	14.2801
**FCGR2A**	0.695999	3.419206	6.7224	5.13E-11	6.69E-10	14.26075
**SGSM1**	-0.59744	3.123457	-6.71982	5.21E-11	6.79E-10	14.245
**PBX3**	0.501933	3.956936	6.717768	5.28E-11	6.87E-10	14.23242
**MARCHF9**	0.511583	4.531879	6.717292	5.30E-11	6.89E-10	14.22951
**TMEM159**	0.501107	2.668639	6.716757	5.32E-11	6.91E-10	14.22623
**CD180**	0.511211	1.590402	6.714151	5.40E-11	7.01E-10	14.2103
**CD302**	0.532402	2.270468	6.706962	5.65E-11	7.31E-10	14.16637
**PARP14**	0.536015	3.865775	6.706052	5.68E-11	7.35E-10	14.16081
**GDF15**	0.732729	1.280611	6.702052	5.83E-11	7.53E-10	14.13639
**S100A3**	0.882797	2.037544	6.701399	5.85E-11	7.55E-10	14.1324

**FCGR3A**	0.931701	5.167167	6.700548	5.88E-11	7.59E-10	14.12721
**TUBA4A**	-0.83796	4.600422	-6.69856	5.96E-11	7.68E-10	14.11509
**SEL1L3**	0.820958	2.4282	6.697828	5.98E-11	7.71E-10	14.11061
**TWIST1**	0.590346	1.597133	6.694812	6.10E-11	7.84E-10	14.09221
**METTL7B**	1.134831	4.005341	6.693394	6.15E-11	7.91E-10	14.08357
**PXDN**	0.66034	3.809472	6.686479	6.42E-11	8.24E-10	14.04142
**GBP1P1**	0.598984	1.329102	6.681291	6.64E-11	8.49E-10	14.00982
**FCGR2C**	0.525757	0.748974	6.681181	6.64E-11	8.49E-10	14.00915
**CORO6**	-0.74445	2.440833	-6.67682	6.82E-11	8.72E-10	13.98262
**SPSB4**	0.610713	3.324001	6.671541	7.05E-11	8.99E-10	13.95049
**AC074135.1**	0.55315	1.542615	6.670015	7.12E-11	9.06E-10	13.94121
**KSR2**	-0.61243	1.811504	-6.66342	7.42E-11	9.43E-10	13.90113
**OLFML2B**	0.74743	3.547267	6.658727	7.64E-11	9.69E-10	13.87263
**SPOCD1**	1.013583	2.027913	6.650702	8.03E-11	1.01E-09	13.82392
**PLVAP**	0.637224	3.975636	6.649451	8.09E-11	1.02E-09	13.81634
**USH1C**	-1.077	4.217872	-6.64735	8.20E-11	1.03E-09	13.80361
**APOL6**	0.534142	3.262207	6.641854	8.48E-11	1.07E-09	13.77029
**MTURN**	-0.53162	8.136882	-6.64054	8.55E-11	1.08E-09	13.76235
**MFNG**	0.517981	3.995888	6.637425	8.72E-11	1.10E-09	13.74346
**AL355916.2**	-1.06401	3.802927	-6.62816	9.23E-11	1.16E-09	13.68741
**AP001972.5**	-0.67988	5.443593	-6.62742	9.28E-11	1.16E-09	13.68289
**CIITA**	0.620165	1.73813	6.62036	9.69E-11	1.21E-09	13.64023
**VWF**	0.582512	4.952191	6.620104	9.71E-11	1.21E-09	13.63868
**SLAMF8**	0.634287	1.763949	6.613459	1.01E-10	1.26E-09	13.59855
**LINC00844**	-0.72393	7.730063	-6.60919	1.04E-10	1.29E-09	13.57277
**KCNN1**	-0.57545	3.713385	-6.60911	1.04E-10	1.29E-09	13.57232
**SERINC2**	0.63276	1.985175	6.608147	1.05E-10	1.30E-09	13.56648
**SFRP2**	-1.50813	5.963052	-6.60487	1.07E-10	1.32E-09	13.54672
**CTSS**	0.662497	4.451737	6.60281	1.08E-10	1.34E-09	13.5343
**CASP1**	0.598901	2.838972	6.601871	1.09E-10	1.35E-09	13.52864
**TM4SF1**	0.69297	3.691036	6.601628	1.09E-10	1.35E-09	13.52717
**SMIM3**	0.53143	4.324812	6.599142	1.11E-10	1.37E-09	13.51219
**MRC2**	0.688825	4.655127	6.598814	1.11E-10	1.37E-09	13.51021
**CDK6-AS1**	0.517584	0.532783	6.597148	1.12E-10	1.38E-09	13.50017
**ARL4C**	0.740101	4.902875	6.595332	1.13E-10	1.40E-09	13.48923
**SLC16A3**	0.535502	2.557707	6.595058	1.13E-10	1.40E-09	13.48758
**ARHGDIG**	-0.80381	4.508013	-6.59059	1.16E-10	1.44E-09	13.46065
**FILIP1L**	0.59642	2.870133	6.588237	1.18E-10	1.46E-09	13.44651
**AC141557.2**	0.530419	1.044639	6.585562	1.20E-10	1.48E-09	13.43041
**PKIB**	0.697101	1.923562	6.585016	1.21E-10	1.48E-09	13.42713
**ANXA5**	0.514289	7.942014	6.580678	1.24E-10	1.52E-09	13.40104
**FGL2**	0.679787	3.271167	6.576803	1.27E-10	1.56E-09	13.37775
**HLA-DPA1**	0.922755	5.392753	6.571096	1.31E-10	1.61E-09	13.34346
**GBP1**	0.845166	3.628924	6.566718	1.35E-10	1.65E-09	13.31718

**MIR590**	0.558379	2.254143	6.55785	1.43E-10	1.74E-09	13.26399
**SOCS2**	0.651932	2.488652	6.552645	1.47E-10	1.79E-09	13.23279
**MS4A7**	0.677761	4.043645	6.548947	1.51E-10	1.83E-09	13.21064
**TNFAIP6**	0.739623	1.729264	6.546141	1.53E-10	1.86E-09	13.19384
**H19**	0.833287	0.744637	6.542501	1.57E-10	1.89E-09	13.17206
**TGFB2**	0.927474	3.319504	6.538251	1.61E-10	1.94E-09	13.14663
**MMP9**	0.805948	1.099773	6.536753	1.62E-10	1.96E-09	13.13767
**LCP2**	0.501127	2.996934	6.5353	1.64E-10	1.97E-09	13.12899
**PNCK**	-0.60835	3.199931	-6.53199	1.67E-10	2.01E-09	13.1092
**SERPINA1**	0.791768	3.190844	6.525351	1.74E-10	2.09E-09	13.06955
**CTHRC1**	0.640166	1.786824	6.522885	1.77E-10	2.12E-09	13.05483
**PRSS12**	0.563151	1.678261	6.517027	1.83E-10	2.19E-09	13.01988
**LINC01579**	0.771087	0.97884	6.514788	1.86E-10	2.22E-09	13.00653
**SLC24A4**	-0.73856	2.562702	-6.51423	1.86E-10	2.23E-09	13.00323
**RDH10**	0.606107	3.280766	6.513952	1.87E-10	2.23E-09	13.00154
**NKX2-5**	0.536841	0.485536	6.50971	1.91E-10	2.28E-09	12.97626
**SLC25A18**	-0.58842	5.959319	-6.50794	1.94E-10	2.31E-09	12.9657
**POSTN**	1.024134	0.977807	6.505877	1.96E-10	2.33E-09	12.95343
**HS3ST4**	-0.72859	2.433887	-6.50484	1.97E-10	2.35E-09	12.94724
**PDGFD**	0.711313	1.652294	6.500755	2.02E-10	2.40E-09	12.92294
**ETS1**	0.554291	4.849539	6.500539	2.02E-10	2.40E-09	12.92165
**KLHL4**	0.697215	2.832094	6.497211	2.07E-10	2.45E-09	12.90185
**TSPYL2**	-0.53418	6.234965	-6.49543	2.09E-10	2.47E-09	12.89128
**INAVA**	0.544679	2.31993	6.493891	2.11E-10	2.50E-09	12.8821
**C2**	0.57708	2.14639	6.487366	2.19E-10	2.59E-09	12.84332
**HAR1A**	-0.64416	2.090529	-6.48333	2.25E-10	2.65E-09	12.81937
**AL354919.2**	0.781978	1.21668	6.47544	2.36E-10	2.78E-09	12.77251
**CSDC2**	-0.90146	5.90945	-6.47177	2.41E-10	2.84E-09	12.75078
**LINC00836**	-0.8868	3.282366	-6.46626	2.50E-10	2.93E-09	12.71807
**C6orf118**	0.552602	1.275695	6.453226	2.70E-10	3.16E-09	12.64093
**RGS16**	0.781161	3.364377	6.440358	2.92E-10	3.40E-09	12.56488
**AEN**	0.522836	3.465881	6.430896	3.09E-10	3.59E-09	12.50904
**PCDHB9**	0.538032	3.782152	6.4301	3.11E-10	3.60E-09	12.50434
**HLA-A**	0.508705	8.894674	6.41931	3.32E-10	3.83E-09	12.44076
**CHODL**	0.549436	1.170156	6.418675	3.33E-10	3.84E-09	12.43702
**PIK3AP1**	0.529737	3.181948	6.416752	3.37E-10	3.88E-09	12.4257
**SFRP4**	0.839025	2.894937	6.413751	3.43E-10	3.95E-09	12.40803
**GNLY**	0.562306	1.273646	6.413694	3.43E-10	3.95E-09	12.40769
**CALN1**	-0.92879	3.665769	-6.40744	3.56E-10	4.09E-09	12.37088
**NRG3**	-0.55711	3.805285	-6.40558	3.60E-10	4.14E-09	12.36
**PNMA6F**	-0.71849	1.243802	-6.40116	3.70E-10	4.24E-09	12.33398
**SOCS3**	1.012612	3.544597	6.399747	3.73E-10	4.28E-09	12.3257
**PPIC**	0.547824	3.179155	6.398903	3.75E-10	4.30E-09	12.32074
**HOTAIRM1**	0.786966	1.468628	6.39665	3.80E-10	4.35E-09	12.30751

**GLIPR1**	0.504572	2.999234	6.3961	3.82E-10	4.36E-09	12.30428
**GADD45A**	0.655759	5.414513	6.387452	4.02E-10	4.58E-09	12.25353
**HOXA3**	0.529563	0.376104	6.385813	4.06E-10	4.62E-09	12.24392
**AC018638.8**	0.558959	3.002592	6.369979	4.46E-10	5.05E-09	12.15118
**AC244517.7**	0.582057	3.585915	6.369971	4.46E-10	5.05E-09	12.15114
**LINC02217**	-0.52123	0.808667	-6.36956	4.48E-10	5.06E-09	12.14875
**RNA5SP118**	0.741124	3.137364	6.360545	4.72E-10	5.33E-09	12.09602
**CD163**	1.059446	3.178005	6.355151	4.88E-10	5.48E-09	12.06451
**MRVI1**	-0.56744	5.082622	-6.34912	5.06E-10	5.67E-09	12.0293
**HOXD-AS2**	0.586726	1.004341	6.345624	5.17E-10	5.78E-09	12.00891
**STAB1**	0.622492	4.292045	6.344044	5.22E-10	5.83E-09	11.9997
**IL17D**	-0.51534	6.23067	-6.33523	5.50E-10	6.12E-09	11.94831
**MAP3K7CL**	0.605164	1.580716	6.333181	5.57E-10	6.19E-09	11.9364
**FKBP10**	0.595647	5.804477	6.328586	5.72E-10	6.35E-09	11.90966
**MIR25**	0.557045	3.963056	6.324467	5.86E-10	6.50E-09	11.8857
**KCNIP2**	-0.88427	5.166371	-6.30938	6.42E-10	7.07E-09	11.79803
**RAC2**	0.566047	2.768979	6.307669	6.48E-10	7.14E-09	11.78811
**C1QB**	0.73428	8.076688	6.303269	6.65E-10	7.32E-09	11.76259
**SIGLEC9**	0.560805	2.324193	6.300771	6.75E-10	7.41E-09	11.74811
**HLA-DQA1**	0.933191	2.838389	6.299504	6.80E-10	7.47E-09	11.74076
**H3C6**	0.584115	1.595	6.28771	7.30E-10	7.97E-09	11.67246
**RAB32**	0.545709	3.924004	6.284762	7.43E-10	8.10E-09	11.6554
**JUN**	0.561742	7.275427	6.278622	7.70E-10	8.36E-09	11.61989
**PROM1**	0.508593	2.398151	6.27628	7.81E-10	8.47E-09	11.60636
**FKBP9**	0.573692	4.455072	6.263038	8.45E-10	9.11E-09	11.52991
**STAC**	0.63671	0.740868	6.255528	8.84E-10	9.50E-09	11.48662
**HOXA4**	0.608269	0.453057	6.254326	8.90E-10	9.56E-09	11.47969
**CCR5**	0.542857	1.403822	6.251349	9.06E-10	9.72E-09	11.46254
**FAM114A1**	0.50878	2.930968	6.245035	9.40E-10	1.01E-08	11.4262
**NNMT**	0.834777	2.000651	6.244295	9.44E-10	1.01E-08	11.42194
**AC113191.1**	0.575109	3.746229	6.242255	9.56E-10	1.02E-08	11.41021
**TGFB2-AS1**	0.743339	2.650242	6.24116	9.62E-10	1.03E-08	11.40391
**NTSR2**	-1.03643	5.281338	-6.23826	9.78E-10	1.04E-08	11.38726
**PHYHD1**	-0.56464	4.589803	-6.23718	9.85E-10	1.05E-08	11.38101
**IGDCC4**	0.631829	3.749735	6.232332	1.01E-09	1.08E-08	11.35317
**PTPRO**	0.517901	3.371056	6.226419	1.05E-09	1.12E-08	11.31922
**S100A4**	0.795114	3.284167	6.221424	1.08E-09	1.15E-08	11.29056
**TLR2**	0.700159	3.261778	6.204161	1.20E-09	1.26E-08	11.19167
**CHI3L1**	1.612682	4.577935	6.204036	1.20E-09	1.26E-08	11.19095
**PSRC1**	0.61394	5.201746	6.199951	1.23E-09	1.29E-08	11.16759
**LINC02488**	0.713091	1.21056	6.199279	1.23E-09	1.30E-08	11.16375
**PTPRC**	0.619118	2.817029	6.191225	1.29E-09	1.36E-08	11.11772
**SOX4**	0.702771	6.7989	6.186702	1.33E-09	1.39E-08	11.09189
**MT-TE**	-0.58998	1.447388	-6.18565	1.33E-09	1.40E-08	11.0859

**SLC44A5**	0.622968	2.868785	6.181421	1.37E-09	1.43E-08	11.06176
**CFI**	0.768784	3.349632	6.177598	1.40E-09	1.46E-08	11.03996
**H2BC5**	0.55022	3.770878	6.177445	1.40E-09	1.46E-08	11.03908
**SEPTIN4**	-0.58456	5.809015	-6.17744	1.40E-09	1.46E-08	11.03903
**H2AC8**	0.526382	2.303618	6.177046	1.40E-09	1.46E-08	11.03681
**TRIB2**	0.553061	6.158377	6.164284	1.51E-09	1.57E-08	10.96412
**KRT222**	-0.57962	1.166459	-6.15938	1.56E-09	1.61E-08	10.93623
**AL450311.1**	-0.59028	2.484943	-6.15123	1.63E-09	1.69E-08	10.88989
**P3H2**	0.564001	2.181249	6.150326	1.64E-09	1.70E-08	10.88477
**SCN2B**	-0.69556	3.805081	-6.1483	1.66E-09	1.72E-08	10.87325
**THBD**	0.563909	1.912897	6.14435	1.70E-09	1.75E-08	10.85085
**FBLN5**	0.656285	3.248421	6.136716	1.78E-09	1.83E-08	10.80754
**NTN1**	0.525653	5.685424	6.124465	1.91E-09	1.95E-08	10.73815
**XAF1**	0.501022	1.623593	6.124291	1.91E-09	1.96E-08	10.73717
**CABP1**	-0.75942	3.017664	-6.12324	1.92E-09	1.97E-08	10.7312
**GDF10**	-0.87135	2.906191	-6.12248	1.93E-09	1.98E-08	10.7269
**LOXL1**	0.584094	1.391924	6.122267	1.93E-09	1.98E-08	10.72572
**FAM107A**	-0.60092	9.1898	-6.12207	1.93E-09	1.98E-08	10.7246
**AL033519.1**	-0.78025	2.027954	-6.1201	1.96E-09	2.00E-08	10.71344
**LRRC25**	0.558174	2.681143	6.116364	2.00E-09	2.04E-08	10.69233
**PPP1R1B**	-0.81131	5.125547	-6.11499	2.02E-09	2.05E-08	10.68459
**ANKRD10-IT1**	0.525365	4.411486	6.11188	2.05E-09	2.09E-08	10.667
**MKX**	-0.68783	2.584784	-6.11152	2.06E-09	2.09E-08	10.66494
**DNALI1**	0.531532	4.493911	6.107348	2.11E-09	2.14E-08	10.6414
**CITED1**	0.673707	3.693564	6.106826	2.11E-09	2.14E-08	10.63846
**MMP16**	0.582787	4.554395	6.103239	2.16E-09	2.19E-08	10.61821
**LY96**	0.589152	3.893165	6.101755	2.18E-09	2.20E-08	10.60984
**IER5L**	0.514999	3.107404	6.099613	2.20E-09	2.23E-08	10.59776
**PANX2**	-0.56149	3.257257	-6.09921	2.21E-09	2.23E-08	10.59546
**MEOX2**	0.928173	1.091397	6.097081	2.24E-09	2.26E-08	10.58348
**CYTL1**	0.641424	3.571733	6.091762	2.31E-09	2.32E-08	10.5535
**MIR125B1**	0.547312	1.241249	6.089892	2.33E-09	2.35E-08	10.54297
**EN2**	0.529024	1.25664	6.088649	2.35E-09	2.36E-08	10.53597
**DMRTA2**	0.754785	0.99097	6.085426	2.39E-09	2.41E-08	10.51783
**SEMA5A**	0.624176	5.089928	6.071921	2.59E-09	2.59E-08	10.44189
**CPXM1**	0.80064	5.807333	6.068917	2.63E-09	2.63E-08	10.42501
**AL023284.4**	-0.60681	3.537336	-6.06736	2.66E-09	2.65E-08	10.41626
**C1QC**	0.669293	8.251769	6.063954	2.71E-09	2.70E-08	10.39715
**IRF7**	0.536832	3.731115	6.057937	2.80E-09	2.79E-08	10.36341
**EVC**	0.56311	1.687766	6.054591	2.86E-09	2.84E-08	10.34465
**SH2D4A**	0.517358	0.941009	6.052761	2.89E-09	2.87E-08	10.3344
**CPNE6**	-0.90902	2.798883	-6.05222	2.90E-09	2.87E-08	10.33135
**AC136475.2**	-0.57938	4.190594	-6.0514	2.91E-09	2.89E-08	10.32678

**PLIN1**	-0.60622	2.165438	-6.04324	3.05E-09	3.02E-08	10.2811
**HTR2A**	-0.64971	2.301878	-6.03754	3.15E-09	3.11E-08	10.2492
**SRGN**	0.514692	6.947666	6.035858	3.18E-09	3.14E-08	10.23981
**GBP3**	0.764568	3.427299	6.034861	3.20E-09	3.15E-08	10.23424
**VIM**	0.860121	8.379418	6.031362	3.27E-09	3.21E-08	10.21469
**HLA-H**	0.5494	5.14868	6.030449	3.29E-09	3.23E-08	10.20959
**COL11A1**	0.804549	2.706451	6.02726	3.35E-09	3.28E-08	10.19179
**GLCCI1**	0.518203	4.912206	6.018152	3.53E-09	3.45E-08	10.14097
**MS4A4A**	0.650001	3.443363	6.015794	3.57E-09	3.49E-08	10.12783
**IL13RA2**	0.961522	1.815502	6.01466	3.60E-09	3.51E-08	10.12151
**KIF6**	-0.53142	1.846149	-6.00662	3.77E-09	3.67E-08	10.07671
**SLC25A21-AS1**	-0.52935	2.823731	-6.00636	3.77E-09	3.67E-08	10.07531
**CLCF1**	0.585148	1.56726	5.999628	3.92E-09	3.81E-08	10.03783
**HLA-DRA**	0.89919	8.54253	5.99768	3.96E-09	3.85E-08	10.02701
**SLC37A2**	0.510649	2.508915	5.988241	4.18E-09	4.04E-08	9.974572
**SPATA6**	0.660819	3.373629	5.980257	4.38E-09	4.22E-08	9.930275
**CA11**	-0.59631	6.678273	-5.97994	4.39E-09	4.23E-08	9.92852
**SNTA1**	-0.58194	6.62741	-5.97094	4.62E-09	4.44E-08	9.87864
**PRLHR**	-1.00987	2.761247	-5.96892	4.67E-09	4.49E-08	9.867478
**RXRG**	-0.55719	3.010892	-5.96838	4.69E-09	4.50E-08	9.864471
**SIGLEC1**	0.628391	1.877044	5.961379	4.88E-09	4.67E-08	9.825742
**HLA-B**	0.558064	8.835342	5.949202	5.23E-09	4.99E-08	9.758465
**STC1**	0.518617	0.827924	5.943732	5.39E-09	5.13E-08	9.728278
**ABCC3**	0.76341	1.130626	5.942365	5.43E-09	5.17E-08	9.720739
**EMP3**	0.949884	3.60591	5.931178	5.79E-09	5.49E-08	9.6591
**SLA**	0.549503	3.312112	5.925385	5.98E-09	5.66E-08	9.627216
**AP000843.1**	-0.55732	1.490109	-5.91984	6.17E-09	5.83E-08	9.596716
**LRRK2**	0.51193	2.574042	5.918914	6.21E-09	5.86E-08	9.591637
**GNG12**	0.736587	4.659585	5.916494	6.29E-09	5.94E-08	9.578337
**ZBED6**	0.546455	2.132492	5.913066	6.42E-09	6.04E-08	9.559511
**MYOM1**	-0.50113	2.772312	-5.90927	6.56E-09	6.16E-08	9.538684
**ECM2**	0.725441	3.240608	5.905659	6.69E-09	6.28E-08	9.518857
**AC009533.1**	0.519569	2.056333	5.898398	6.97E-09	6.53E-08	9.479048
**DUSP6**	0.576494	4.314707	5.894676	7.12E-09	6.66E-08	9.458659
**ANKRD22**	0.772495	2.425002	5.893133	7.18E-09	6.71E-08	9.450213
**ARHGAP44**	-0.55195	2.518052	-5.89025	7.30E-09	6.82E-08	9.434425
**FSTL5**	-0.76088	2.63452	-5.88807	7.39E-09	6.89E-08	9.422513
**GSC**	0.55299	1.772439	5.886	7.48E-09	6.97E-08	9.411175
**ADA2**	0.502686	3.862322	5.870801	8.14E-09	7.55E-08	9.328135
**GRB14**	0.524939	1.031084	5.861605	8.58E-09	7.92E-08	9.277986
**HLA-DMA**	0.637108	5.169884	5.858569	8.72E-09	8.05E-08	9.261445
**FERMT3**	0.509213	4.043129	5.856788	8.81E-09	8.12E-08	9.251744
**SLC22A6**	-0.64682	2.509707	-5.85081	9.11E-09	8.38E-08	9.219212

**MACORIS**	0.522446	3.503467	5.844179	9.46E-09	8.68E-08	9.183139
**ALOX5AP**	0.744382	4.593455	5.842279	9.56E-09	8.76E-08	9.17281
**ITGAL**	0.527027	2.14138	5.840838	9.64E-09	8.82E-08	9.16498
**MIR3125**	0.563444	1.55203	5.836906	9.85E-09	9.00E-08	9.143625
**LINC02308**	0.513099	1.322708	5.831733	1.01E-08	9.26E-08	9.115543
**LMO1**	0.642952	2.856148	5.820113	1.08E-08	9.83E-08	9.052547
**GEM**	0.697058	4.202539	5.819123	1.09E-08	9.88E-08	9.047188
**SAA1**	0.685283	0.604498	5.811887	1.13E-08	1.03E-07	9.008019
**AJ011932.1**	0.543783	0.921569	5.80644	1.17E-08	1.06E-07	8.978564
**IFI6**	0.698543	8.044144	5.798704	1.22E-08	1.10E-07	8.936771
**CXCL11**	0.534497	0.882529	5.793022	1.26E-08	1.13E-07	8.906104
**EYA4**	0.500738	0.687486	5.787075	1.30E-08	1.17E-07	8.874035
**AKR1C3**	-0.56648	4.150417	-5.78493	1.32E-08	1.18E-07	8.862456
**UCP2**	0.587535	4.93057	5.782962	1.33E-08	1.19E-07	8.851875
**BCL2A1**	0.656089	2.081397	5.78284	1.33E-08	1.19E-07	8.851218
**SLC14A1**	-1.08328	4.140513	-5.77751	1.37E-08	1.23E-07	8.822518
**HOXB3**	0.54347	0.480432	5.775223	1.39E-08	1.24E-07	8.810213
**FCER1G**	0.617128	6.051402	5.772302	1.41E-08	1.26E-07	8.794502
**LINC02587**	0.81155	0.882262	5.770775	1.42E-08	1.27E-07	8.786289
**AGAP2-AS1**	0.644267	2.083904	5.768936	1.44E-08	1.28E-07	8.776404
**CD300A**	0.518493	3.567446	5.768491	1.44E-08	1.29E-07	8.774012
**C1QTNF4**	-0.60187	3.099248	-5.7557	1.55E-08	1.38E-07	8.705345
**DPYSL5**	0.500388	5.236454	5.751541	1.58E-08	1.41E-07	8.683027
**CLEC5A**	0.568711	0.9295	5.747328	1.62E-08	1.44E-07	8.660444
**HLA-DOA**	0.773473	3.467159	5.746844	1.63E-08	1.44E-07	8.657853
**ESM1**	0.599691	0.787114	5.743968	1.65E-08	1.46E-07	8.642448
**MIR10B**	0.558831	0.77298	5.739386	1.69E-08	1.50E-07	8.617921
**FAM131C**	-0.58296	2.839323	-5.73832	1.70E-08	1.50E-07	8.612224
**PLP2**	0.65067	4.643193	5.734373	1.74E-08	1.54E-07	8.591105
**LINC01561**	-0.50383	3.138455	-5.73401	1.74E-08	1.54E-07	8.589138
**FAM133A**	-0.54332	3.02335	-5.73259	1.76E-08	1.55E-07	8.581549
**KLK7**	-0.60132	1.000682	-5.72332	1.85E-08	1.63E-07	8.532042
**THBS1**	0.668852	1.945703	5.715486	1.93E-08	1.69E-07	8.490254
**SLC2A10**	0.588403	2.621868	5.710978	1.98E-08	1.73E-07	8.466229
**FOSL1**	0.58605	1.883727	5.707574	2.02E-08	1.76E-07	8.448097
**EMILIN3**	0.954308	2.07	5.707527	2.02E-08	1.76E-07	8.447848
**SMIM10L2B**	-0.60196	3.365413	-5.69874	2.12E-08	1.84E-07	8.401059
**C1QA**	0.638974	7.805767	5.696483	2.15E-08	1.86E-07	8.38908
**INSM1**	0.660596	3.402476	5.692544	2.19E-08	1.90E-07	8.368144
**MATK**	-0.59602	2.632876	-5.69112	2.21E-08	1.91E-07	8.360576
**SERPINA3**	0.547527	1.13002	5.686008	2.27E-08	1.97E-07	8.333437
**HCP5**	0.514447	2.769105	5.684204	2.29E-08	1.99E-07	8.323866
**MARVELD1**	0.506306	3.046783	5.682896	2.31E-08	2.00E-07	8.316922
**CCN4**	0.525146	0.877769	5.68245	2.32E-08	2.00E-07	8.314558

**ABI3**	0.541112	4.015679	5.666787	2.52E-08	2.17E-07	8.231566
**C1RL**	0.548664	2.788454	5.662471	2.58E-08	2.21E-07	8.208732
**SERPINI1**	-0.74368	5.383357	-5.66176	2.59E-08	2.22E-07	8.204956
**HLA-DRB1**	0.822468	7.561012	5.660649	2.61E-08	2.24E-07	8.199094
**HLA-DMB**	0.622947	3.977446	5.658909	2.63E-08	2.25E-07	8.189896
**OAS1**	0.618087	3.075679	5.656536	2.67E-08	2.28E-07	8.177358
**AL391845.2**	-0.53244	1.454129	-5.65199	2.74E-08	2.34E-07	8.153342
**AF131216.3**	-0.6105	3.516004	-5.65132	2.75E-08	2.34E-07	8.149813
**AC002428.2**	-0.60621	1.518014	-5.6501	2.76E-08	2.36E-07	8.143392
**CAV1**	0.675889	4.267502	5.643604	2.86E-08	2.44E-07	8.109091
**CALY**	-0.90856	3.121867	-5.63356	3.03E-08	2.56E-07	8.056187
**LINC01532**	-0.73873	2.896534	-5.62655	3.14E-08	2.66E-07	8.019289
**RSPH4A**	0.503732	1.514227	5.623605	3.19E-08	2.70E-07	8.003794
**SIGLEC14**	0.601696	2.59452	5.61489	3.35E-08	2.82E-07	7.958014
**GPC3**	0.558195	1.546523	5.614816	3.35E-08	2.82E-07	7.957623
**HLA-DRB5**	0.875055	5.979075	5.613056	3.38E-08	2.84E-07	7.948388
**TBX15**	0.524596	2.216734	5.607933	3.48E-08	2.92E-07	7.921508
**SLCO1A2**	-0.57387	4.11219	-5.60085	3.61E-08	3.03E-07	7.884384
**SLC11A1**	0.684085	2.618899	5.599779	3.63E-08	3.04E-07	7.878777
**CHRNA4**	-0.54567	2.726411	-5.59692	3.69E-08	3.09E-07	7.863804
**LINC02607**	-0.60081	1.080101	-5.59295	3.77E-08	3.15E-07	7.843051
**LCP1**	0.525149	4.269988	5.586789	3.90E-08	3.25E-07	7.810811
**SNCB**	-1.00447	5.719118	-5.58591	3.92E-08	3.26E-07	7.806191
**RTN1**	-0.55473	7.330221	-5.58285	3.98E-08	3.31E-07	7.790224
**TCF12**	0.51128	7.125734	5.577931	4.09E-08	3.40E-07	7.764545
**LINC01485**	-0.52519	1.535602	-5.56327	4.42E-08	3.66E-07	7.688093
**CARTPT**	-0.75065	0.854107	-5.54586	4.86E-08	4.00E-07	7.597566
**DUSP26**	-0.50424	6.175891	-5.54281	4.94E-08	4.07E-07	7.581723
**CYBB**	0.654936	4.897517	5.541732	4.97E-08	4.09E-07	7.576137
**SLC25A43**	0.520434	2.802187	5.535511	5.14E-08	4.22E-07	7.543869
**CMYA5**	0.518359	1.204779	5.533058	5.20E-08	4.27E-07	7.531151
**F5**	-0.91676	2.544825	-5.52688	5.38E-08	4.40E-07	7.499137
**NUP210**	0.529019	3.617707	5.518851	5.62E-08	4.59E-07	7.457604
**NCKAP1L**	0.502369	3.199473	5.514948	5.73E-08	4.67E-07	7.437426
**DUSP5**	0.65594	2.770853	5.513708	5.77E-08	4.70E-07	7.431019
**HLA-DPB1**	0.772598	6.011524	5.507668	5.96E-08	4.85E-07	7.399829
**BEX2**	-0.53083	6.802021	-5.50654	6.00E-08	4.87E-07	7.394015
**CNNM1**	-0.5829	2.004629	-5.50135	6.17E-08	5.00E-07	7.36724
**SOD2**	0.585852	5.461826	5.500824	6.18E-08	5.02E-07	7.364523
**CCER2**	0.572451	2.183336	5.496316	6.33E-08	5.13E-07	7.341284
**AL139393.3**	0.533794	4.0552	5.495452	6.36E-08	5.15E-07	7.336834
**AC141557.1**	0.723152	2.281883	5.493022	6.45E-08	5.22E-07	7.324316
**RNASE2**	0.701602	2.515737	5.491271	6.51E-08	5.26E-07	7.3153
**AL357992.1**	0.500445	1.247874	5.486443	6.68E-08	5.39E-07	7.290457

**KNDC1**	-0.53116	4.217204	-5.47982	6.91E-08	5.57E-07	7.256394
**PHYHIP**	-0.86717	5.263286	-5.47292	7.17E-08	5.76E-07	7.220986
**LINC01007**	-0.56814	0.60364	-5.47284	7.18E-08	5.76E-07	7.220549
**NPY**	-0.84611	4.576563	-5.4691	7.32E-08	5.87E-07	7.201338
**MIR5587**	0.571972	1.691704	5.458843	7.73E-08	6.17E-07	7.148786
**GBP2**	0.743903	3.738614	5.455887	7.85E-08	6.26E-07	7.133654
**SDS**	-0.58036	3.323076	-5.45032	8.09E-08	6.44E-07	7.10518
**RNASE6**	0.541974	4.821997	5.449946	8.10E-08	6.45E-07	7.103257
**LYPD6**	0.524959	2.280484	5.44852	8.16E-08	6.49E-07	7.095962
**AC109439.1**	-0.71173	2.178321	-5.44359	8.38E-08	6.65E-07	7.070761
**SASH3**	0.540319	3.91628	5.441603	8.47E-08	6.71E-07	7.060616
**BLM**	0.577621	2.755648	5.439964	8.54E-08	6.77E-07	7.052246
**SNORC**	-0.72962	3.79365	-5.43074	8.97E-08	7.08E-07	7.005202
**ACBD7**	-0.72627	4.429142	-5.42986	9.01E-08	7.11E-07	7.000693
**JPH3**	-0.67769	4.811673	-5.42388	9.30E-08	7.31E-07	6.970221
**AC023421.1**	-0.67434	1.686189	-5.42148	9.42E-08	7.40E-07	6.957993
**CALHM6**	0.536036	2.250708	5.420371	9.47E-08	7.44E-07	6.952366
**SALL3**	0.589618	4.082434	5.419401	9.52E-08	7.48E-07	6.947431
**C1R**	0.697611	4.910229	5.416266	9.68E-08	7.59E-07	6.931482
**HK2**	0.521051	3.805019	5.41575	9.70E-08	7.61E-07	6.928855
**APOL1**	0.509223	3.286158	5.41237	9.88E-08	7.74E-07	6.911673
**ADAMTS1**	0.523264	2.992804	5.409813	1.00E-07	7.83E-07	6.898679
**TMEM151B**	-0.73106	4.357	-5.40705	1.02E-07	7.94E-07	6.884631
**PNMA3**	-0.58755	3.707093	-5.39882	1.06E-07	8.26E-07	6.842866
**SPP1**	0.827754	8.954714	5.395452	1.08E-07	8.39E-07	6.825796
**HOXB7**	0.510948	1.113957	5.390686	1.11E-07	8.59E-07	6.801648
**TUBB4A**	-0.58103	8.092545	-5.37893	1.18E-07	9.10E-07	6.742183
**HHATL**	-0.66804	4.520328	-5.37302	1.21E-07	9.36E-07	6.71229
**PTGS1**	0.543814	3.275174	5.365039	1.27E-07	9.73E-07	6.672018
**ID3**	0.653881	8.168403	5.357887	1.31E-07	1.01E-06	6.635966
**HLA-DQB1**	0.79737	3.714582	5.354001	1.34E-07	1.03E-06	6.616398
**HRH3**	-0.76015	3.02902	-5.34746	1.39E-07	1.06E-06	6.583482
**HS3ST3B1**	0.519907	0.922992	5.346958	1.39E-07	1.06E-06	6.580958
**FEZF2**	-0.53474	2.154702	-5.34537	1.40E-07	1.07E-06	6.57297
**FLNC**	0.774776	3.533048	5.332771	1.50E-07	1.14E-06	6.509704
**HSPA7**	0.610811	1.531783	5.329146	1.53E-07	1.16E-06	6.491524
**ABCC8**	-0.70531	3.759673	-5.3273	1.54E-07	1.17E-06	6.482257
**WWTR1**	0.56197	4.407913	5.325094	1.56E-07	1.18E-06	6.471213
**NEUROD6**	-0.73817	1.396378	-5.3198	1.60E-07	1.21E-06	6.444723
**SH3GL2**	-0.66533	6.436623	-5.30812	1.70E-07	1.28E-06	6.386301
**FOXJ1**	0.739154	3.476257	5.306699	1.71E-07	1.29E-06	6.379189
**IGFBP3**	0.818471	4.637248	5.305542	1.73E-07	1.30E-06	6.373411
**C21orf62**	0.712791	2.900641	5.300509	1.77E-07	1.33E-06	6.348286
**PDGFA**	0.522911	4.277021	5.295787	1.81E-07	1.36E-06	6.324734

**AC021613.1**	-0.52477	0.936789	-5.29427	1.83E-07	1.37E-06	6.317177
**SAMSN1**	0.52745	3.303151	5.290485	1.86E-07	1.39E-06	6.298313
**ZIC1**	0.537185	3.719695	5.287446	1.89E-07	1.41E-06	6.283175
**LINC02381**	0.603081	3.792466	5.280586	1.96E-07	1.46E-06	6.249039
**IGSF9B**	0.54294	3.083786	5.272035	2.05E-07	1.52E-06	6.206543
**DIRAS3**	0.644331	3.064684	5.26545	2.12E-07	1.57E-06	6.173863
**GABBR1**	-0.50147	7.487837	-5.2633	2.15E-07	1.59E-06	6.163191
**HOXB2**	0.521543	0.827656	5.261563	2.16E-07	1.60E-06	6.154587
**SOHLH1**	-0.60031	1.431313	-5.24056	2.41E-07	1.77E-06	6.050651
**TMEM151A**	-0.61552	4.830281	-5.24041	2.41E-07	1.77E-06	6.049923
**CD74**	0.697538	9.048877	5.239006	2.43E-07	1.78E-06	6.042979
**LAIR1**	0.512778	3.112436	5.233349	2.50E-07	1.83E-06	6.015055
**EMX2OS**	-0.57328	2.934297	-5.22934	2.55E-07	1.87E-06	5.995282
**GPIHBP1**	-0.54816	4.228247	-5.22637	2.59E-07	1.89E-06	5.980628
**SSTR2**	-0.66185	3.670205	-5.22448	2.62E-07	1.91E-06	5.971358
**RUNDC3A**	-0.51308	6.23296	-5.21381	2.76E-07	2.01E-06	5.918838
**CNGA3**	0.7844	2.667483	5.201252	2.95E-07	2.13E-06	5.85714
**HMOX1**	0.501036	5.428248	5.187909	3.15E-07	2.27E-06	5.791746
**WNK2**	-0.56239	3.381495	-5.18765	3.16E-07	2.27E-06	5.790464
**SST**	-0.88741	4.30203	-5.1844	3.21E-07	2.31E-06	5.774582
**TMSB4XP8**	0.519223	4.978639	5.182057	3.25E-07	2.33E-06	5.76311
**SELL**	-0.98964	5.527587	-5.17959	3.29E-07	2.36E-06	5.75104
**GJB6**	-0.9278	2.453792	-5.1793	3.29E-07	2.36E-06	5.749647
**HIF3A**	-0.6591	3.367652	-5.17005	3.45E-07	2.47E-06	5.704466
**KCNJ3**	-0.61285	2.588248	-5.16316	3.58E-07	2.55E-06	5.670847
**AL359091.1**	-0.6191	4.306844	-5.15866	3.66E-07	2.61E-06	5.648937
**HPR**	-0.73066	3.834047	-5.15224	3.78E-07	2.69E-06	5.617651
**PSD**	-0.61663	5.445245	-5.13571	4.11E-07	2.90E-06	5.537348
**MPPED1**	-0.74589	2.108876	-5.13257	4.17E-07	2.95E-06	5.522124
**EMX2**	-0.551	3.307766	-5.13034	4.22E-07	2.98E-06	5.51133
**SLC8A2**	-0.6756	3.668358	-5.12564	4.32E-07	3.05E-06	5.488564
**HCLS1**	0.518469	4.721459	5.11194	4.63E-07	3.25E-06	5.422284
**GLIS3**	0.520678	3.087758	5.108771	4.70E-07	3.30E-06	5.406976
**ASIC2**	-0.50913	1.61334	-5.10623	4.76E-07	3.34E-06	5.394703
**GRIN1**	-0.94417	4.335483	-5.09632	5.00E-07	3.50E-06	5.346925
**ATF3**	0.568248	3.483662	5.094887	5.04E-07	3.52E-06	5.340016
**CACNG3**	-0.85039	2.441276	-5.08983	5.17E-07	3.60E-06	5.315663
**TMEM271**	-0.64386	3.3249	-5.0828	5.35E-07	3.73E-06	5.281878
**NGFR**	0.657764	3.224327	5.07772	5.49E-07	3.81E-06	5.257454
**C9orf64**	0.509671	2.930818	5.07209	5.65E-07	3.91E-06	5.230432
**EMP1**	0.67929	4.955281	5.069406	5.72E-07	3.96E-06	5.217556
**SNAP91**	-0.61553	5.164439	-5.05878	6.04E-07	4.16E-06	5.166637
**NSG2**	-0.81115	6.766044	-5.05543	6.14E-07	4.23E-06	5.1506
**TMEM130**	-0.8904	4.29666	-5.05028	6.30E-07	4.33E-06	5.125982

**C7orf57**	0.598206	0.917365	5.044463	6.48E-07	4.44E-06	5.098213
**CAMSAP3**	-0.50345	3.374895	-5.04392	6.50E-07	4.45E-06	5.095611
**AKR1C1**	-0.50261	2.247102	-5.04013	6.62E-07	4.53E-06	5.07755
**BST2**	0.51636	6.491625	5.035213	6.79E-07	4.63E-06	5.054093
**TPPP**	-0.55511	6.287595	-5.03445	6.81E-07	4.65E-06	5.050473
**AC008780.1**	-0.56296	1.188654	-5.03444	6.81E-07	4.65E-06	5.050409
**PYGM**	-0.579	3.210558	-5.02997	6.96E-07	4.75E-06	5.029114
**FPR1**	0.658697	3.922619	5.025476	7.12E-07	4.85E-06	5.007725
**S100A9**	0.797284	4.202804	5.023422	7.19E-07	4.89E-06	4.997954
**LINC01088**	-0.82807	4.420159	-5.01311	7.57E-07	5.13E-06	4.948935
**RNU6-353P**	-0.50417	1.797667	-5.00965	7.70E-07	5.21E-06	4.932523
**ITGB2**	0.553937	4.912263	4.998157	8.15E-07	5.49E-06	4.878062
**HOXD8**	0.72468	1.664381	4.995634	8.25E-07	5.55E-06	4.866118
**CCK**	-0.93845	3.519326	-4.99176	8.41E-07	5.65E-06	4.847808
**IBSP**	0.574295	0.610226	4.986365	8.64E-07	5.79E-06	4.822288
**NTM-AS1**	-0.55353	2.363872	-4.98289	8.78E-07	5.88E-06	4.805888
**MDK**	0.586734	5.356403	4.982187	8.81E-07	5.90E-06	4.802557
**AC090204.1**	0.591763	2.825728	4.980687	8.88E-07	5.94E-06	4.795477
**RTP5**	-0.75368	4.694043	-4.9761	9.08E-07	6.06E-06	4.77384
**FBXL16**	-0.55049	6.56774	-4.97584	9.09E-07	6.07E-06	4.772604
**LAPTM5**	0.516653	7.309949	4.973623	9.19E-07	6.13E-06	4.762161
**AL022313.4**	-0.83182	3.945487	-4.96623	9.53E-07	6.34E-06	4.727312
**NEUROD2**	-0.61473	1.706113	-4.95449	1.01E-06	6.70E-06	4.672125
**HAVCR2**	0.506901	4.238815	4.952935	1.02E-06	6.75E-06	4.664827
**PACSIN1**	-1.00994	4.143944	-4.94465	1.06E-06	7.00E-06	4.625964
**LINC02732**	0.589236	1.703397	4.938942	1.09E-06	7.18E-06	4.599197
**MTND6P4**	-0.51129	2.567678	-4.9303	1.14E-06	7.47E-06	4.55875
**CPNE9**	-0.51508	1.576323	-4.92505	1.17E-06	7.66E-06	4.53421
**SSTR1**	-0.69007	3.119609	-4.91761	1.21E-06	7.92E-06	4.499495
**ISG15**	0.635504	6.22974	4.916849	1.21E-06	7.95E-06	4.495922
**SOWAHA**	-0.62959	3.09552	-4.91422	1.23E-06	8.04E-06	4.483641
**IFI44L**	0.630827	3.874662	4.892804	1.36E-06	8.86E-06	4.383996
**SLC1A2**	-0.5823	7.68188	-4.89222	1.37E-06	8.88E-06	4.381277
**HSPB8**	-0.54929	7.732305	-4.88768	1.40E-06	9.05E-06	4.360232
**PCDHB7**	0.571968	2.357128	4.883351	1.43E-06	9.22E-06	4.340132
**PPP2R2C**	-0.67333	4.16648	-4.87349	1.50E-06	9.65E-06	4.294469
**S100A8**	0.688867	2.625894	4.864294	1.56E-06	1.00E-05	4.251932
**RAB34**	0.53416	3.441762	4.858039	1.61E-06	1.03E-05	4.223047
**MAL**	-0.84079	5.333544	-4.85718	1.62E-06	1.04E-05	4.219088
**CRTAC1**	-0.65601	5.545226	-4.85071	1.67E-06	1.07E-05	4.189265
**SCIN**	0.600264	2.56451	4.850441	1.67E-06	1.07E-05	4.188013
**PDIA2**	-0.55123	3.402137	-4.84125	1.75E-06	1.11E-05	4.145686
**LRRN4CL**	0.583508	1.900527	4.836085	1.79E-06	1.14E-05	4.121946
**JAKMIP1**	-0.55894	3.428747	-4.8256	1.88E-06	1.19E-05	4.073824

**GPR183**	0.530337	3.14655	4.824435	1.89E-06	1.20E-05	4.068465
**PDE1A**	-0.5167	2.760063	-4.82429	1.89E-06	1.20E-05	4.067818
**CAPG**	0.561805	5.322924	4.808538	2.04E-06	1.29E-05	3.995671
**CCL5**	0.527203	2.173303	4.785475	2.28E-06	1.42E-05	3.890455
**F13A1**	0.640218	2.34988	4.776673	2.38E-06	1.48E-05	3.850423
**C3**	0.674624	7.019144	4.772985	2.42E-06	1.50E-05	3.83367
**F2RL1**	0.559123	2.280382	4.772855	2.42E-06	1.50E-05	3.833076
**CHRNA1**	0.527929	0.941423	4.765574	2.51E-06	1.55E-05	3.800037
**TCEAL6**	-0.69805	3.521157	-4.7651	2.51E-06	1.56E-05	3.797873
**AIFM3**	-0.62127	2.935457	-4.762	2.55E-06	1.58E-05	3.783816
**NPM2**	-0.58398	2.771898	-4.75021	2.69E-06	1.66E-05	3.730471
**LGI3**	-0.60232	4.921313	-4.73584	2.88E-06	1.77E-05	3.665557
**FABP5**	0.62119	2.748321	4.719125	3.12E-06	1.90E-05	3.590324
**GOLGA7B**	-0.50473	3.954704	-4.71846	3.13E-06	1.91E-05	3.58732
**COL22A1**	0.624361	1.802242	4.71436	3.19E-06	1.94E-05	3.568919
**TF**	-0.59518	6.660295	-4.70837	3.28E-06	1.99E-05	3.542037
**SIX1**	0.524064	2.228279	4.707217	3.30E-06	2.00E-05	3.536861
**GPNMB**	0.713575	3.645654	4.705501	3.32E-06	2.02E-05	3.529168
**AK5**	-0.70751	3.925127	-4.69965	3.42E-06	2.07E-05	3.502962
**AC122707.1**	-0.61345	2.282884	-4.69813	3.44E-06	2.08E-05	3.496141
**AC084880.1**	-0.50875	3.919873	-4.69479	3.50E-06	2.11E-05	3.481206
**GPR27**	-0.56388	4.380128	-4.69089	3.56E-06	2.15E-05	3.463763
**SVOP**	-0.75436	3.244809	-4.68309	3.69E-06	2.22E-05	3.428923
**TNFSF13B**	0.635073	3.11249	4.679909	3.75E-06	2.25E-05	3.414724
**WSCD2**	-0.57818	2.092049	-4.66282	4.06E-06	2.43E-05	3.338625
**AC013553.3**	-0.50563	2.845794	-4.66113	4.09E-06	2.44E-05	3.331119
**SLC6A7**	-0.6487	1.489402	-4.65893	4.13E-06	2.47E-05	3.321345
**VSNL1**	-1.02911	4.581628	-4.65733	4.16E-06	2.48E-05	3.314232
**DES**	0.569847	1.10099	4.649445	4.32E-06	2.57E-05	3.27924
**SPRY4**	0.674984	3.298591	4.643306	4.44E-06	2.63E-05	3.252033
**PRKCG**	-0.76554	2.967166	-4.63748	4.56E-06	2.70E-05	3.226252
**NGB**	-0.6381	1.480308	-4.62798	4.77E-06	2.81E-05	3.184271
**OGDHL**	-0.60338	2.85729	-4.62173	4.91E-06	2.89E-05	3.156678
**PCDHB8**	0.506909	1.839845	4.608531	5.22E-06	3.05E-05	3.098546
**ATP2B3**	-0.61432	2.256356	-4.60393	5.33E-06	3.11E-05	3.078296
**TBR1**	-0.52622	1.408239	-4.60235	5.37E-06	3.14E-05	3.07136
**KBTBD11-OT1**	-0.52607	3.39052	-4.59687	5.50E-06	3.21E-05	3.047317
**CCN2**	0.505678	6.546828	4.592285	5.62E-06	3.28E-05	3.027201
**VCAM1**	0.70873	3.965962	4.592096	5.62E-06	3.28E-05	3.026373
**GRIN3A**	-0.60032	2.210003	-4.58951	5.69E-06	3.31E-05	3.015028
**F3**	0.580058	5.806918	4.587497	5.74E-06	3.34E-05	3.006219
**PLA2G2A**	0.55788	0.584454	4.581821	5.90E-06	3.42E-05	2.981372
**IGF2**	0.559687	3.455319	4.57254	6.15E-06	3.56E-05	2.940803

**RAB3A**	-0.59565	5.917121	-4.56426	6.39E-06	3.69E-05	2.904659
**LTF**	0.910459	1.663225	4.56108	6.48E-06	3.74E-05	2.890814
**AQP5**	0.537473	0.912133	4.559738	6.52E-06	3.76E-05	2.884965
**HS3ST1**	0.51892	2.571412	4.550858	6.79E-06	3.90E-05	2.846323
**LYVE1**	-0.59209	2.937928	-4.53961	7.15E-06	4.09E-05	2.797475
**GABRA5**	-0.70549	2.421527	-4.53679	7.24E-06	4.14E-05	2.785241
**OPALIN**	-0.90586	3.34358	-4.50515	8.36E-06	4.72E-05	2.648485
**SNAP25**	-0.84397	7.091278	-4.49772	8.64E-06	4.87E-05	2.616493
**TAFA1**	-0.57238	2.003405	-4.49568	8.72E-06	4.91E-05	2.607715
**RBFOX3**	-0.60749	2.132831	-4.49306	8.82E-06	4.96E-05	2.596463
**RASGRF1**	-0.54423	3.341665	-4.46866	9.85E-06	5.48E-05	2.491879
**SYNPR**	-0.72331	2.66604	-4.46437	1.00E-05	5.58E-05	2.473527
**DLGAP1-AS4**	-0.54095	1.591031	-4.46367	1.01E-05	5.59E-05	2.470525
**LHX5-AS1**	-0.8399	2.293097	-4.46232	1.01E-05	5.62E-05	2.464765
**SLC17A7**	-1.0131	4.603526	-4.46075	1.02E-05	5.66E-05	2.458068
**AC107398.3**	-0.50108	3.879386	-4.46063	1.02E-05	5.66E-05	2.457562
**AL049749.1**	-0.62401	2.568528	-4.45645	1.04E-05	5.76E-05	2.439728
**CHGA**	-0.78994	5.197903	-4.45366	1.05E-05	5.82E-05	2.427823
**CHRM1**	-0.66416	3.404036	-4.44725	1.08E-05	5.98E-05	2.400541
**HLA-DQB2**	0.57432	1.721505	4.444126	1.10E-05	6.05E-05	2.38723
**NEFM**	-0.90039	3.430654	-4.43924	1.12E-05	6.17E-05	2.366461
**G0S2**	0.602947	1.738457	4.435597	1.14E-05	6.27E-05	2.350974
**SULT4A1**	-0.85509	4.083634	-4.43513	1.14E-05	6.28E-05	2.348989
**IGFBP5**	0.513278	7.017678	4.43437	1.15E-05	6.30E-05	2.345766
**CAMK2A**	-0.96125	4.698479	-4.41373	1.26E-05	6.84E-05	2.258315
**MYBPC1**	-0.50717	3.226323	-4.40842	1.29E-05	6.99E-05	2.23588
**CP**	0.636951	2.598955	4.407288	1.29E-05	7.03E-05	2.231102
**LY6H**	-0.60968	5.11528	-4.40542	1.31E-05	7.08E-05	2.223209
**BEX5**	-0.62109	4.358412	-4.39616	1.36E-05	7.35E-05	2.184182
**NFIA-AS2**	0.516646	3.454964	4.389722	1.40E-05	7.54E-05	2.157075
**GRIK1**	0.571191	2.306142	4.385819	1.42E-05	7.66E-05	2.140664
**CCKBR**	-0.54639	1.657781	-4.37648	1.48E-05	7.96E-05	2.101453
**CD14**	0.538328	6.385504	4.364636	1.56E-05	8.36E-05	2.051829
**TMEM235**	-0.65222	2.645181	-4.3646	1.56E-05	8.36E-05	2.051682
**RGS1**	0.672084	4.749352	4.363849	1.57E-05	8.38E-05	2.048535
**GAP43**	0.526695	7.89634	4.363802	1.57E-05	8.38E-05	2.048339
**ETV1**	0.524845	6.415222	4.354555	1.63E-05	8.70E-05	2.009693
**SV2B**	-0.71656	2.463699	-4.34836	1.68E-05	8.92E-05	1.983859
**CD44**	0.582158	5.676043	4.338904	1.75E-05	9.27E-05	1.944451
**SYT13**	-0.73316	3.281219	-4.33349	1.79E-05	9.47E-05	1.921937
**DDN**	-0.80323	3.825831	-4.33235	1.80E-05	9.52E-05	1.917199
**ATP1A2**	-0.5528	8.784691	-4.30725	2.01E-05	0.0001052	1.813169
**CCN1**	0.657083	5.234555	4.299081	2.08E-05	0.0001087	1.779429

**CPLX2**	-0.80954	5.510399	-4.28522	2.21E-05	0.0001149	1.722339
**MT-TF**	-0.61261	4.783588	-4.28296	2.23E-05	0.0001159	1.713013
**SYT4**	-0.7588	3.556194	-4.28053	2.26E-05	0.000117	1.703037
**RPE65**	0.623131	3.469416	4.276523	2.30E-05	0.0001189	1.686575
**DAAM2**	-0.54792	7.001274	-4.2741	2.32E-05	0.0001201	1.676622
**IGFBPL1**	0.603335	2.61897	4.264465	2.42E-05	0.0001249	1.637129
**SYNGR3**	-0.62188	3.590571	-4.26318	2.43E-05	0.0001255	1.631853
**SLC12A5**	-0.65038	3.235115	-4.25982	2.47E-05	0.0001272	1.618117
**ZFR2**	-0.52242	1.770225	-4.25273	2.54E-05	0.0001308	1.589124
**PVALB**	-0.66648	1.929257	-4.25196	2.55E-05	0.0001312	1.585973
**SYN1**	-0.63356	6.005985	-4.23039	2.80E-05	0.0001428	1.498101
**MIR3151**	0.500798	2.708849	4.229949	2.80E-05	0.0001431	1.4963
**OCIAD2**	0.527585	2.83867	4.218969	2.94E-05	0.0001494	1.451722
**INA**	-0.74279	5.606853	-4.21681	2.97E-05	0.0001507	1.442969
**VIP**	-0.50566	1.728085	-4.19224	3.29E-05	0.0001656	1.343639
**VSTM2A**	-0.65251	3.376336	-4.18005	3.47E-05	0.0001737	1.294579
**PRKCB**	-0.51542	4.281533	-4.17804	3.50E-05	0.0001751	1.28651
**OR4N2**	0.5679	1.355673	4.168306	3.65E-05	0.0001816	1.247432
**MAL2**	-0.64715	2.210172	-4.16547	3.69E-05	0.0001837	1.236081
**SEC14L5**	-0.53466	2.872475	-4.16256	3.74E-05	0.0001857	1.224421
**C4B**	0.511847	3.275377	4.147218	3.99E-05	0.000197	1.163075
**NRGN**	-0.90915	6.868153	-4.13859	4.13E-05	0.0002035	1.128662
**SYN2**	-0.64565	4.598127	-4.13149	4.26E-05	0.0002091	1.100405
**SERPING1**	0.531821	5.251935	4.130979	4.27E-05	0.0002095	1.098385
**RBP4**	-0.56874	2.338123	-4.13056	4.27E-05	0.0002098	1.096726
**CAMK2B**	-0.53402	4.38013	-4.12377	4.40E-05	0.0002153	1.069752
**CELF4**	-0.59844	3.514282	-4.1232	4.41E-05	0.0002157	1.067481
**TMEM132D**	-0.52069	1.556769	-4.12044	4.46E-05	0.000218	1.056529
**EGR1**	0.645959	6.847936	4.109723	4.67E-05	0.0002271	1.014067
**SH2D5**	-0.5176	1.915997	-4.09741	4.91E-05	0.0002381	0.965409
**CPLX1**	-0.53561	5.393202	-4.08477	5.18E-05	0.0002498	0.91561
**KCNH3**	-0.53861	3.27266	-4.08105	5.26E-05	0.0002533	0.900985
**KCNJ11**	-0.54843	4.173143	-4.07762	5.33E-05	0.0002566	0.887472
**TMEM155**	-0.57365	1.942578	-4.07631	5.36E-05	0.0002578	0.882356
**CHD5**	-0.58333	3.019613	-4.07158	5.47E-05	0.0002625	0.863765
**NGEF**	-0.60911	4.162487	-4.07061	5.49E-05	0.0002635	0.859978
**FABP7**	0.565516	5.983055	4.064509	5.63E-05	0.0002698	0.836062
**GABRA1**	-0.73098	2.769563	-4.04074	6.21E-05	0.0002956	0.743213
**SULF1**	0.587817	3.206292	4.035082	6.36E-05	0.000302	0.721192
**NOG**	-0.50016	4.387801	-4.03239	6.43E-05	0.0003049	0.710738
**SPOCK3**	-0.52214	4.496098	-4.02622	6.59E-05	0.0003121	0.686765
**CLEC2L**	-0.55142	2.132832	-4.02422	6.65E-05	0.0003145	0.678976
**LINC00599**	-0.50626	3.13768	-3.98756	7.73E-05	0.0003608	0.537353
**RASAL1**	-0.53001	2.043987	-3.98497	7.81E-05	0.0003643	0.527368

**AMER3**	-0.50762	2.358925	-3.97679	8.07E-05	0.0003756	0.495959
**RBFOX1**	-0.59007	2.498488	-3.97439	8.15E-05	0.000379	0.486744
**RBP1**	0.577753	3.253077	3.965076	8.47E-05	0.0003923	0.451061
**HSPA6**	0.508303	2.065184	3.961513	8.59E-05	0.0003974	0.43743
**TESPA1**	-0.66449	1.629096	-3.95995	8.64E-05	0.0003998	0.431456
**SLC26A4-AS1**	-0.52282	1.574125	-3.95114	8.96E-05	0.0004131	0.397822
**CDK5R2**	-0.58193	4.292846	-3.92785	9.84E-05	0.0004508	0.30922
**AC062021.1**	-0.80158	3.592077	-3.92283	0.0001	0.0004592	0.290174
**NEFL**	-0.88628	4.277055	-3.88484	0.000117	0.0005268	0.146898
**SCRT1**	-0.59457	3.902986	-3.84959	0.000134	0.000597	0.015109
**TRH**	0.610075	1.839834	3.844422	0.000137	0.0006083	-0.00411
**SHISA6**	-0.59702	3.094166	-3.8303	0.000145	0.0006407	-0.05651
**FXYD7**	-0.6296	3.984488	-3.80487	0.00016	0.0007021	-0.15043
**SHISAL1**	-0.54085	3.323926	-3.77103	0.000183	0.0007908	-0.27453
**CACNG2**	-0.55897	2.731106	-3.76693	0.000186	0.0008019	-0.28947
**CRYM**	-0.65828	2.825982	-3.74429	0.000203	0.0008684	-0.37182
**LEFTY2**	0.548573	1.639638	3.742498	0.000204	0.0008737	-0.37832
**GDA**	-0.60968	2.521455	-3.73716	0.000209	0.0008906	-0.39766
**HLA-DRB6**	0.570149	3.655266	3.72771	0.000216	0.0009211	-0.43182
**SYT5**	-0.50672	3.082962	-3.7154	0.000227	0.000961	-0.4762
**FAM163B**	-0.6392	4.506696	-3.68496	0.000255	0.0010688	-0.58536
**IQSEC3**	-0.53489	3.084101	-3.6815	0.000258	0.0010816	-0.59774
**MSTN**	0.623647	3.616937	3.678335	0.000262	0.0010929	-0.60903
**AEBP1**	0.668549	5.530841	3.671927	0.000268	0.0011171	-0.63186
**CHGB**	-0.56124	5.868205	-3.66876	0.000271	0.0011298	-0.64312
**SLC6A17**	-0.62394	3.300621	-3.65847	0.000282	0.0011706	-0.67968
**MOXD1**	0.616912	1.856609	3.646537	0.000295	0.0012203	-0.72196
**ACTL6B**	-0.54155	4.580837	-3.62765	0.000317	0.0013007	-0.78861
**CAMKV**	-0.54044	4.158688	-3.62489	0.00032	0.0013132	-0.79833
**GABRB2**	-0.56484	2.185867	-3.61779	0.000329	0.0013447	-0.82327
**GABRG2**	-0.65992	3.597076	-3.61725	0.00033	0.0013468	-0.82516
**HLA-DQA2**	0.633859	2.445144	3.61167	0.000337	0.0013724	-0.84474
**CREG2**	-0.63881	2.837636	-3.59678	0.000356	0.001444	-0.89684
**TMEM125**	-0.55471	3.209537	-3.58181	0.000376	0.001519	-0.94901
**MTCO3P12**	-0.5218	4.128044	-3.56885	0.000395	0.0015861	-0.99398
**CAMK1G**	-0.60739	2.540346	-3.54545	0.000431	0.0017177	-1.07483
**GNG3**	-0.67553	5.157287	-3.52796	0.000459	0.0018214	-1.13492
**LHX5**	-0.50093	1.574881	-3.49945	0.00051	0.002002	-1.2323
**FOS**	0.507147	7.074231	3.498236	0.000512	0.0020093	-1.23643
**ACY3**	-0.52635	3.5572	-3.487	0.000534	0.0020843	-1.27458
**SYT1**	-0.68132	4.895078	-3.47477	0.000558	0.0021671	-1.31596
**CARNS1**	-0.61038	4.625117	-3.46733	0.000573	0.0022212	-1.34108
**KCNJ4**	-0.50949	3.381792	-3.40565	0.000716	0.0027097	-1.54731

**MTND1P23**	-0.51871	3.224726	-3.38419	0.000773	0.0029007	-1.61826
**NKX6-2**	-0.50734	3.579058	-3.37163	0.000808	0.0030212	-1.65955
**PHF24**	-0.53284	3.228791	-3.36748	0.00082	0.0030597	-1.67319
**NPTX1**	-0.57199	4.03567	-3.29788	0.001047	0.0038047	-1.89922
**MAP7D2**	-0.52066	2.40291	-3.25649	0.001209	0.0043234	-2.03148
**GPX3**	-0.53544	6.992094	-3.20787	0.001427	0.005016	-2.1848
**SLN**	0.568931	2.507671	3.141922	0.001783	0.0061135	-2.38926
**MBP**	-0.62632	7.448929	-3.081	0.002183	0.0073237	-2.5745
**RGS4**	-0.56899	3.56924	-3.03824	0.00251	0.0082965	-2.70244
**WIF1**	-0.51396	1.783799	-3.03459	0.00254	0.0083858	-2.71327
**MAG**	-0.60104	6.086627	-3.01015	0.00275	0.0089978	-2.78557
**KLK6**	-0.53955	4.799185	-2.98489	0.002983	0.0096757	-2.85967
**HPCA**	-0.57959	4.4652	-2.95692	0.003262	0.0104939	-2.94101
**MOBP**	-0.54665	4.674174	-2.82401	0.004942	0.0151323	-3.31749
**AQP1**	0.534012	7.46253	2.800582	0.005309	0.0161226	-3.38211
**ERMN**	-0.51343	5.360558	-2.69934	0.007195	0.0210502	-3.6554
**LINC01602**	0.563495	1.58996	2.644647	0.008448	0.0242445	-3.79896
