# Supplementary material for: PPP1R81 correlates with the survival and cell proliferation in lower-grade glioma
Source: Biosci Rep. 2023 May 5;43(5):BSR20230028. doi: 10.1042/BSR20230028 (PMC10170297; doi:10.1042/BSR20230028)
Supplement: Supplementary Tables S1-S4 [file BSR-2023-0028_supp1.zip › BSR-2023-0028_suppS4.docx]

**Supplementary Table S4.** Differentially expressed genes (DEGs) in CGGA LGG cohort.

| **id** | **logFC** | **AveExpr** | **t** | **P.Value** | **adj.P.Val** | **B** |
| --- | --- | --- | --- | --- | --- | --- |
| **TPX2** | 1.943051 | 2.046052 | 20.96161 | 3.07E-67 | 7.35E-63 | 142.3177038 |
| **CDK1** | 2.051796 | 2.242246 | 20.69368 | 4.79E-66 | 5.74E-62 | 139.5945891 |
| **UBE2C** | 2.650427 | 3.154714 | 20.60295 | 1.21E-65 | 9.70E-62 | 138.6722765 |
| **FAM64A** | 2.173822 | 2.243069 | 20.51461 | 3.00E-65 | 1.80E-61 | 137.7741257 |
| **ASF1B** | 1.715115 | 1.699275 | 20.44525 | 6.12E-65 | 2.93E-61 | 137.068998 |
| **BIRC5** | 2.018361 | 2.170801 | 20.14569 | 1.32E-63 | 5.28E-60 | 134.0233207 |
| **KIFC1** | 1.885575 | 1.930566 | 20.01873 | 4.86E-63 | 1.59E-59 | 132.7325405 |
| **PBK** | 1.873986 | 1.710772 | 20.00999 | 5.32E-63 | 1.59E-59 | 132.6436456 |
| **RRM2** | 1.905786 | 1.670867 | 19.7954 | 4.80E-62 | 1.28E-58 | 130.4622998 |
| **NUSAP1** | 2.253376 | 2.456446 | 19.56833 | 4.92E-61 | 1.18E-57 | 128.154677 |
| **MYBL2** | 1.921928 | 1.891202 | 19.39143 | 3.02E-60 | 6.58E-57 | 126.3576537 |
| **CCNB2** | 1.82215 | 1.692584 | 19.34309 | 4.95E-60 | 9.89E-57 | 125.8666795 |
| **KIAA0101** | 1.91224 | 2.200264 | 19.3037 | 7.41E-60 | 1.37E-56 | 125.4666994 |
| **TROAP** | 1.752559 | 1.648307 | 19.18569 | 2.48E-59 | 4.25E-56 | 124.2685779 |
| **AURKB** | 2.018807 | 2.257566 | 19.17502 | 2.77E-59 | 4.43E-56 | 124.160195 |
| **FOXM1** | 1.922386 | 2.230173 | 19.16836 | 2.96E-59 | 4.44E-56 | 124.0926322 |
| **NCAPH** | 1.509442 | 1.451492 | 18.8639 | 6.68E-58 | 9.43E-55 | 121.0037251 |
| **MLF1IP** | 1.719582 | 1.848303 | 18.75866 | 1.96E-57 | 2.61E-54 | 119.9368183 |
| **ZWINT** | 1.860924 | 2.55175 | 18.54544 | 1.73E-56 | 2.19E-53 | 117.776744 |
| **TK1** | 1.695711 | 2.328656 | 18.52943 | 2.04E-56 | 2.45E-53 | 117.6146442 |
| **NCAPG** | 1.333203 | 1.102514 | 18.49358 | 2.94E-56 | 3.36E-53 | 117.2517081 |
| **KIF2C** | 1.590115 | 1.551653 | 18.48252 | 3.29E-56 | 3.59E-53 | 117.1397181 |
| **NDC80** | 1.463934 | 1.272003 | 18.45263 | 4.47E-56 | 4.66E-53 | 116.8371551 |
| **KIF11** | 1.26913 | 1.251909 | 18.35344 | 1.23E-55 | 1.23E-52 | 115.8335566 |
| **GTSE1** | 1.496765 | 1.352857 | 18.31751 | 1.77E-55 | 1.70E-52 | 115.4700932 |
| **MKI67** | 1.386583 | 1.162441 | 18.23739 | 4.02E-55 | 3.70E-52 | 114.6599855 |
| **CDCA3** | 1.589751 | 2.342356 | 18.17169 | 7.85E-55 | 6.97E-52 | 113.9959808 |
| **MELK** | 1.479161 | 1.147445 | 18.14883 | 9.91E-55 | 8.48E-52 | 113.7650296 |
| **RAD51** | 1.152342 | 1.179249 | 17.99162 | 4.91E-54 | 4.06E-51 | 112.1774114 |
| **CENPA** | 1.311134 | 1.152753 | 17.9498 | 7.52E-54 | 6.01E-51 | 111.7553832 |
| **HJURP** | 1.393825 | 1.258764 | 17.93393 | 8.84E-54 | 6.68E-51 | 111.5953352 |
| **CENPK** | 1.405668 | 1.381916 | 17.93301 | 8.92E-54 | 6.68E-51 | 111.5859935 |
| **CENPF** | 1.872106 | 1.649603 | 17.85806 | 1.91E-53 | 1.39E-50 | 110.8299943 |
| **NUF2** | 1.600383 | 1.588525 | 17.80063 | 3.43E-53 | 2.42E-50 | 110.2510163 |
| **CDCA5** | 1.528832 | 2.229486 | 17.62867 | 1.97E-52 | 1.35E-49 | 108.5188674 |
| **CDCA2** | 0.82218 | 0.557806 | 17.5892 | 2.94E-52 | 1.96E-49 | 108.1216118 |
| **SPC24** | 1.539918 | 1.87492 | 17.54577 | 4.56E-52 | 2.96E-49 | 107.6846774 |
| **KIF4A** | 1.261758 | 1.01641 | 17.48649 | 8.33E-52 | 5.25E-49 | 107.0884986 |
| **ZNF367** | 1.206363 | 1.335535 | 17.45503 | 1.15E-51 | 7.04E-49 | 106.7722759 |
| **ESPL1** | 1.291719 | 1.11074 | 17.4062 | 1.88E-51 | 1.13E-48 | 106.2815083 |
| **BUB1** | 1.301108 | 1.043526 | 17.23704 | 1.04E-50 | 6.09E-48 | 104.583277 |
| **CDC45** | 1.514757 | 1.748277 | 17.22039 | 1.23E-50 | 7.04E-48 | 104.4162365 |
| **CEP55** | 0.991058 | 0.721499 | 17.17764 | 1.90E-50 | 1.06E-47 | 103.9875044 |
| **SKA3** | 1.117084 | 1.006485 | 17.08803 | 4.70E-50 | 2.56E-47 | 103.0895152 |
| **TOP2A** | 2.273335 | 3.162195 | 17.08419 | 4.89E-50 | 2.60E-47 | 103.0510719 |
| **DTL** | 1.185027 | 1.01367 | 17.05329 | 6.68E-50 | 3.48E-47 | 102.7415994 |
| **CDCA4** | 1.116229 | 1.769013 | 17.03765 | 7.82E-50 | 3.99E-47 | 102.5849492 |
| **LMNB1** | 1.965681 | 2.570836 | 17.02709 | 8.70E-50 | 4.30E-47 | 102.4792787 |
| **OIP5** | 1.085452 | 1.079441 | 17.02601 | 8.79E-50 | 4.30E-47 | 102.4684701 |
| **CDCA8** | 1.248443 | 1.115393 | 16.99867 | 1.16E-49 | 5.56E-47 | 102.1948246 |
| **AURKA** | 1.282015 | 1.492842 | 16.97818 | 1.42E-49 | 6.70E-47 | 101.9897649 |
| **CCNB1** | 1.684265 | 2.479611 | 16.87319 | 4.11E-49 | 1.89E-46 | 100.9398713 |
| **CENPH** | 1.379977 | 2.302309 | 16.85657 | 4.86E-49 | 2.20E-46 | 100.7737759 |
| **FANCD2** | 1.344078 | 1.798669 | 16.82378 | 6.76E-49 | 3.00E-46 | 100.4461023 |
| **CDC25C** | 0.911366 | 0.796923 | 16.81842 | 7.13E-49 | 3.11E-46 | 100.3925993 |
| **CCNA2** | 1.286189 | 1.570655 | 16.73724 | 1.62E-48 | 6.92E-46 | 99.5820667 |
| **RAD51AP1** | 1.434152 | 1.568774 | 16.73541 | 1.65E-48 | 6.92E-46 | 99.56389551 |
| **FANCI** | 1.511983 | 2.202384 | 16.43608 | 3.33E-47 | 1.38E-44 | 96.58214644 |
| **KIF23** | 1.247369 | 1.107501 | 16.39312 | 5.12E-47 | 2.08E-44 | 96.15511001 |
| **LRR1** | 1.126732 | 1.831938 | 16.38826 | 5.38E-47 | 2.15E-44 | 96.10679472 |
| **SKA1** | 0.859621 | 0.696449 | 16.33062 | 9.58E-47 | 3.77E-44 | 95.53430941 |
| **KIF20A** | 1.220345 | 0.956494 | 16.32472 | 1.02E-46 | 3.93E-44 | 95.47577158 |
| **ARHGAP11A** | 1.200312 | 1.174702 | 16.2391 | 2.40E-46 | 9.12E-44 | 94.62611446 |
| **TRIP13** | 1.106384 | 1.236638 | 16.18688 | 4.04E-46 | 1.51E-43 | 94.10844294 |
| **BUB1B** | 1.401952 | 1.223467 | 16.18274 | 4.21E-46 | 1.55E-43 | 94.06742778 |
| **CDC6** | 0.973978 | 1.036992 | 16.11018 | 8.69E-46 | 3.16E-43 | 93.34877154 |
| **TTK** | 1.072391 | 0.806095 | 16.10676 | 8.99E-46 | 3.22E-43 | 93.3149195 |
| **TIMELESS** | 1.508991 | 2.229409 | 16.07929 | 1.18E-45 | 4.17E-43 | 93.04306018 |
| **PTTG1** | 2.042676 | 3.827791 | 16.04274 | 1.70E-45 | 5.92E-43 | 92.68154686 |
| **MCM2** | 1.624916 | 2.591454 | 16.03707 | 1.80E-45 | 6.17E-43 | 92.62545865 |
| **EZH2** | 1.608682 | 2.441779 | 15.95191 | 4.21E-45 | 1.42E-42 | 91.78386245 |
| **MND1** | 1.083651 | 1.224305 | 15.94236 | 4.63E-45 | 1.54E-42 | 91.68951223 |
| **EXO1** | 0.962077 | 0.812753 | 15.93316 | 5.08E-45 | 1.67E-42 | 91.5987266 |
| **WDR62** | 1.071019 | 1.243151 | 15.90856 | 6.49E-45 | 2.10E-42 | 91.3558711 |
| **MAD2L1** | 1.533284 | 2.334133 | 15.88698 | 8.04E-45 | 2.57E-42 | 91.14286984 |
| **CDC25A** | 1.027079 | 1.241956 | 15.86884 | 9.63E-45 | 3.04E-42 | 90.96394694 |
| **TYMS** | 1.939422 | 3.15402 | 15.7898 | 2.11E-44 | 6.58E-42 | 90.18477217 |
| **WEE1** | 1.698982 | 2.069076 | 15.77832 | 2.37E-44 | 7.28E-42 | 90.07167709 |
| **KIF15** | 1.136047 | 1.276281 | 15.76286 | 2.76E-44 | 8.38E-42 | 89.91943314 |
| **CDC20** | 1.788139 | 2.379078 | 15.75507 | 2.98E-44 | 8.94E-42 | 89.84273478 |
| **TCF19** | 1.108423 | 1.672374 | 15.74949 | 3.15E-44 | 9.34E-42 | 89.78777539 |
| **FAM111B** | 0.941493 | 0.70158 | 15.72599 | 3.98E-44 | 1.16E-41 | 89.55648363 |
| **CKAP2L** | 1.009468 | 0.743283 | 15.72186 | 4.15E-44 | 1.20E-41 | 89.51582282 |
| **PRC1** | 1.721102 | 3.125993 | 15.71466 | 4.46E-44 | 1.27E-41 | 89.44496372 |
| **CASC5** | 0.982151 | 0.79233 | 15.6824 | 6.14E-44 | 1.73E-41 | 89.12756134 |
| **C11orf82** | 0.854485 | 0.795461 | 15.60765 | 1.29E-43 | 3.59E-41 | 88.39294314 |
| **KIF18B** | 1.572541 | 1.738782 | 15.57105 | 1.85E-43 | 5.10E-41 | 88.03354445 |
| **GINS1** | 1.1312 | 1.640286 | 15.5374 | 2.58E-43 | 7.04E-41 | 87.70321997 |
| **IQGAP3** | 1.078743 | 0.889969 | 15.49716 | 3.85E-43 | 1.04E-40 | 87.30860294 |
| **E2F2** | 0.877592 | 0.702759 | 15.46733 | 5.17E-43 | 1.38E-40 | 87.01617917 |
| **HELLS** | 1.168197 | 1.437335 | 15.43577 | 7.06E-43 | 1.84E-40 | 86.7070372 |
| **TACC3** | 1.670925 | 3.207151 | 15.43564 | 7.07E-43 | 1.84E-40 | 86.70570419 |
| **CHEK1** | 1.210774 | 2.290553 | 15.39634 | 1.04E-42 | 2.69E-40 | 86.32102107 |
| **DLGAP5** | 1.060191 | 0.728571 | 15.36006 | 1.49E-42 | 3.80E-40 | 85.9659886 |
| **ECT2** | 1.302613 | 1.82238 | 15.34835 | 1.67E-42 | 4.22E-40 | 85.85147783 |
| **GINS2** | 1.105657 | 1.65513 | 15.33725 | 1.87E-42 | 4.66E-40 | 85.74292239 |
| **SGOL1** | 0.985695 | 0.778883 | 15.33122 | 1.98E-42 | 4.90E-40 | 85.68402906 |
| **MCM10** | 0.998149 | 0.727846 | 15.31368 | 2.35E-42 | 5.76E-40 | 85.51252181 |
| **CDKN3** | 1.511536 | 2.35491 | 15.28582 | 3.10E-42 | 7.50E-40 | 85.24036934 |
| **POC1A** | 1.016566 | 1.737207 | 15.26231 | 3.91E-42 | 9.37E-40 | 85.01076776 |
| **E2F1** | 1.385743 | 2.940525 | 15.16252 | 1.04E-41 | 2.47E-39 | 84.03736732 |
| **FBXO5** | 1.297987 | 1.80499 | 15.16166 | 1.05E-41 | 2.47E-39 | 84.02897094 |
| **ORC1** | 0.708182 | 0.596572 | 15.14087 | 1.29E-41 | 3.00E-39 | 83.82642033 |
| **ASPM** | 0.990498 | 0.681821 | 15.12637 | 1.49E-41 | 3.43E-39 | 83.68518428 |
| **CENPN** | 1.372761 | 2.170652 | 15.09149 | 2.10E-41 | 4.79E-39 | 83.34558527 |
| **NEK2** | 1.111925 | 0.987279 | 15.01522 | 4.43E-41 | 1.00E-38 | 82.60385622 |
| **RCC1** | 1.450524 | 2.716795 | 15.00762 | 4.77E-41 | 1.07E-38 | 82.53005116 |
| **ESCO2** | 0.749062 | 0.563161 | 14.92637 | 1.06E-40 | 2.35E-38 | 81.74116288 |
| **CDT1** | 1.383504 | 2.370449 | 14.91928 | 1.13E-40 | 2.49E-38 | 81.6724494 |
| **IKBIP** | 1.173217 | 2.010786 | 14.89263 | 1.47E-40 | 3.21E-38 | 81.41401766 |
| **ARHGEF39** | 0.825884 | 0.907139 | 14.8769 | 1.72E-40 | 3.71E-38 | 81.26158665 |
| **FANCC** | 0.910196 | 1.586127 | 14.84899 | 2.25E-40 | 4.83E-38 | 80.99113116 |
| **DEPDC1B** | 0.919927 | 0.772514 | 14.79255 | 3.91E-40 | 8.30E-38 | 80.44481946 |
| **PLK1** | 1.31877 | 2.371121 | 14.77422 | 4.68E-40 | 9.84E-38 | 80.26751514 |
| **TMSB15A** | 2.131937 | 3.088046 | 14.70632 | 9.07E-40 | 1.89E-37 | 79.61142289 |
| **BRCA1** | 1.002933 | 1.657238 | 14.68723 | 1.09E-39 | 2.26E-37 | 79.42712093 |
| **ORC6** | 1.317071 | 2.251376 | 14.64828 | 1.60E-39 | 3.27E-37 | 79.0512769 |
| **HMMR** | 0.929051 | 0.771071 | 14.64296 | 1.68E-39 | 3.41E-37 | 78.99996917 |
| **RMI2** | 1.129162 | 1.825414 | 14.61597 | 2.18E-39 | 4.40E-37 | 78.73979924 |
| **EME1** | 1.127381 | 1.737014 | 14.61118 | 2.29E-39 | 4.57E-37 | 78.69358272 |
| **WDR76** | 1.009323 | 1.646913 | 14.59997 | 2.55E-39 | 5.06E-37 | 78.58561851 |
| **PARPBP** | 0.818111 | 0.974263 | 14.56369 | 3.63E-39 | 7.14E-37 | 78.236201 |
| **PLK4** | 1.11795 | 1.237554 | 14.52059 | 5.52E-39 | 1.08E-36 | 77.82152464 |
| **SHCBP1** | 1.001976 | 1.499995 | 14.49217 | 7.27E-39 | 1.41E-36 | 77.54825154 |
| **CDK2** | 1.795184 | 2.599844 | 14.43007 | 1.33E-38 | 2.55E-36 | 76.95184011 |
| **BRIP1** | 0.629333 | 0.489421 | 14.37789 | 2.20E-38 | 4.19E-36 | 76.45134248 |
| **RNF122** | 1.206541 | 2.065904 | 14.34763 | 2.95E-38 | 5.56E-36 | 76.1613841 |
| **SMC4** | 1.736386 | 2.322898 | 14.33968 | 3.18E-38 | 5.96E-36 | 76.08528949 |
| **RTKN2** | 0.844417 | 0.897762 | 14.24033 | 8.30E-38 | 1.54E-35 | 75.13486254 |
| **KIF14** | 0.65054 | 0.462773 | 14.21807 | 1.03E-37 | 1.90E-35 | 74.92222564 |
| **CENPL** | 0.917983 | 1.330693 | 14.21295 | 1.08E-37 | 1.98E-35 | 74.87336776 |
| **DBF4** | 1.192183 | 1.965531 | 14.16447 | 1.72E-37 | 3.13E-35 | 74.41070055 |
| **DSN1** | 1.099674 | 2.458882 | 14.12807 | 2.45E-37 | 4.41E-35 | 74.06373857 |
| **CENPW** | 1.146348 | 1.862213 | 14.11988 | 2.65E-37 | 4.74E-35 | 73.98572005 |
| **TONSL** | 1.008368 | 1.920637 | 14.0341 | 6.03E-37 | 1.07E-34 | 73.16938194 |
| **SOX11** | 1.561812 | 2.417104 | 14.00456 | 8.01E-37 | 1.41E-34 | 72.88862643 |
| **CKS2** | 1.986954 | 4.152248 | 13.97723 | 1.04E-36 | 1.82E-34 | 72.62913917 |
| **C17orf53** | 0.686573 | 1.014439 | 13.96818 | 1.14E-36 | 1.97E-34 | 72.54324335 |
| **TICRR** | 0.745689 | 0.865575 | 13.94946 | 1.36E-36 | 2.34E-34 | 72.36563614 |
| **SGOL2** | 0.83944 | 0.937486 | 13.94887 | 1.37E-36 | 2.34E-34 | 72.36009744 |
| **FAM83D** | 0.996256 | 0.858627 | 13.88897 | 2.42E-36 | 4.12E-34 | 71.79232086 |
| **TRIM24** | 1.51481 | 3.217693 | 13.86821 | 2.95E-36 | 4.99E-34 | 71.59574348 |
| **RACGAP1** | 1.449678 | 2.759442 | 13.86064 | 3.18E-36 | 5.32E-34 | 71.52413351 |
| **CENPM** | 1.408832 | 2.617066 | 13.79036 | 6.21E-36 | 1.03E-33 | 70.85964588 |
| **MASTL** | 0.934111 | 1.323428 | 13.73385 | 1.06E-35 | 1.76E-33 | 70.32627245 |
| **TMPO-AS1** | 0.535935 | 0.853399 | 13.70086 | 1.46E-35 | 2.39E-33 | 70.01529525 |
| **AC004381.6** | 0.89911 | 1.817763 | 13.62719 | 2.93E-35 | 4.78E-33 | 69.3218138 |
| **CENPO** | 1.111748 | 2.57079 | 13.6133 | 3.35E-35 | 5.42E-33 | 69.19120806 |
| **CHAF1A** | 1.386752 | 3.231313 | 13.59498 | 3.98E-35 | 6.40E-33 | 69.01906224 |
| **ZWILCH** | 0.971016 | 1.632238 | 13.50302 | 9.51E-35 | 1.52E-32 | 68.1562925 |
| **CDCA7** | 1.331962 | 1.811926 | 13.4874 | 1.10E-34 | 1.75E-32 | 68.00988768 |
| **GSG2** | 0.645163 | 0.470996 | 13.47202 | 1.27E-34 | 2.01E-32 | 67.86586872 |
| **WDHD1** | 0.811045 | 1.11829 | 13.46365 | 1.38E-34 | 2.16E-32 | 67.78755733 |
| **LMNB2** | 1.401744 | 3.953476 | 13.46139 | 1.41E-34 | 2.19E-32 | 67.76639477 |
| **CASP2** | 1.142744 | 2.421927 | 13.4453 | 1.64E-34 | 2.54E-32 | 67.6158737 |
| **GAS2L3** | 0.922605 | 0.778737 | 13.42099 | 2.06E-34 | 3.17E-32 | 67.38850531 |
| **KDELC1** | 0.9639 | 1.858425 | 13.41512 | 2.18E-34 | 3.33E-32 | 67.33368415 |
| **NCAPD2** | 1.435872 | 2.937764 | 13.41461 | 2.19E-34 | 3.33E-32 | 67.32887127 |
| **MCM6** | 1.231307 | 2.565083 | 13.40952 | 2.30E-34 | 3.47E-32 | 67.28132257 |
| **CHEK2** | 0.968454 | 1.968097 | 13.39795 | 2.57E-34 | 3.84E-32 | 67.17318324 |
| **NEIL3** | 0.615757 | 0.41896 | 13.33732 | 4.54E-34 | 6.76E-32 | 66.60737661 |
| **NKIRAS2** | 1.480581 | 3.862768 | 13.32949 | 4.89E-34 | 7.24E-32 | 66.53438206 |
| **NRM** | 1.380917 | 3.223995 | 13.32238 | 5.23E-34 | 7.69E-32 | 66.46810958 |
| **POLE2** | 0.836721 | 1.27319 | 13.30714 | 6.03E-34 | 8.82E-32 | 66.32604906 |
| **KPNA2** | 1.588287 | 3.990304 | 13.28842 | 7.19E-34 | 1.05E-31 | 66.1516729 |
| **GPC2** | 1.646472 | 3.448985 | 13.21427 | 1.44E-33 | 2.08E-31 | 65.4621277 |
| **SUV39H2** | 1.137321 | 2.169087 | 13.1987 | 1.67E-33 | 2.40E-31 | 65.31748569 |
| **CKAP2** | 1.438041 | 2.525601 | 13.19711 | 1.70E-33 | 2.42E-31 | 65.30269113 |
| **COL4A1** | 2.169565 | 2.694447 | 13.1922 | 1.77E-33 | 2.52E-31 | 65.25715647 |
| **KIF18A** | 0.627344 | 0.4766 | 13.16612 | 2.27E-33 | 3.20E-31 | 65.01512244 |
| **GPX7** | 1.329893 | 2.759919 | 13.12073 | 3.47E-33 | 4.86E-31 | 64.59435075 |
| **KIAA0922** | 0.870422 | 1.559445 | 13.10165 | 4.14E-33 | 5.78E-31 | 64.4176547 |
| **NCAPG2** | 1.273434 | 2.1439 | 13.09449 | 4.43E-33 | 6.14E-31 | 64.35145156 |
| **TMEM45A** | 1.477664 | 2.737407 | 13.08574 | 4.81E-33 | 6.62E-31 | 64.27044884 |
| **ZNF93** | 1.00959 | 1.698416 | 13.08364 | 4.90E-33 | 6.72E-31 | 64.25106659 |
| **INTS7** | 0.981696 | 2.065213 | 13.05098 | 6.65E-33 | 9.01E-31 | 63.94904346 |
| **LIN9** | 0.728139 | 1.136289 | 13.04969 | 6.73E-33 | 9.07E-31 | 63.93710084 |
| **MCM3** | 1.439298 | 3.540267 | 13.04076 | 7.31E-33 | 9.80E-31 | 63.85458897 |
| **PIM1** | 1.263173 | 2.870355 | 13.01996 | 8.88E-33 | 1.18E-30 | 63.66246466 |
| **C5orf34** | 0.685432 | 0.858695 | 13.00314 | 1.04E-32 | 1.38E-30 | 63.50721871 |
| **GAS1** | 1.759271 | 2.939165 | 13.00171 | 1.05E-32 | 1.39E-30 | 63.49400671 |
| **ATAD2** | 1.115505 | 1.822146 | 12.9745 | 1.36E-32 | 1.77E-30 | 63.24299055 |
| **UBE2T** | 1.479518 | 2.79884 | 12.97447 | 1.36E-32 | 1.77E-30 | 63.24277922 |
| **RAD18** | 0.833935 | 1.332065 | 12.95738 | 1.59E-32 | 2.06E-30 | 63.08527886 |
| **CENPI** | 0.592624 | 0.534559 | 12.94354 | 1.81E-32 | 2.32E-30 | 62.95775931 |
| **CCDC77** | 1.007298 | 2.350126 | 12.94344 | 1.81E-32 | 2.32E-30 | 62.95685819 |
| **TP53** | 1.692728 | 4.054716 | 12.93577 | 1.94E-32 | 2.48E-30 | 62.88622123 |
| **DDX12P** | 0.942759 | 1.350742 | 12.93147 | 2.02E-32 | 2.57E-30 | 62.846567 |
| **FAM72D** | 0.625314 | 0.707679 | 12.9301 | 2.05E-32 | 2.59E-30 | 62.83400469 |
| **CDKN2C** | 1.70344 | 3.752287 | 12.9139 | 2.38E-32 | 2.99E-30 | 62.68492104 |
| **C19orf40** | 0.581388 | 0.797288 | 12.91049 | 2.46E-32 | 3.07E-30 | 62.65347212 |
| **UGDH** | 1.258687 | 2.747502 | 12.88596 | 3.09E-32 | 3.84E-30 | 62.42796837 |
| **DSCC1** | 0.827766 | 1.718165 | 12.88454 | 3.13E-32 | 3.87E-30 | 62.41486127 |
| **MTFR2** | 0.621295 | 0.763379 | 12.87885 | 3.30E-32 | 4.06E-30 | 62.36259293 |
| **SERPINH1** | 1.776474 | 3.923147 | 12.84646 | 4.46E-32 | 5.45E-30 | 62.06503358 |
| **CDC7** | 1.180235 | 1.982104 | 12.81 | 6.25E-32 | 7.60E-30 | 61.73057467 |
| **NDC1** | 0.858846 | 1.823081 | 12.80873 | 6.32E-32 | 7.66E-30 | 61.71889011 |
| **CENPE** | 0.625616 | 0.475672 | 12.80801 | 6.36E-32 | 7.67E-30 | 61.71230466 |
| **MCM8** | 0.793214 | 0.984603 | 12.79456 | 7.21E-32 | 8.64E-30 | 61.58905239 |
| **RFC3** | 1.125041 | 1.939939 | 12.79063 | 7.47E-32 | 8.92E-30 | 61.55299039 |
| **DEPDC1** | 0.660531 | 0.450224 | 12.773 | 8.80E-32 | 1.04E-29 | 61.39146887 |
| **CCNF** | 0.7626 | 1.425991 | 12.75092 | 1.08E-31 | 1.27E-29 | 61.18937797 |
| **DESI2** | 1.214386 | 2.344407 | 12.73065 | 1.30E-31 | 1.53E-29 | 61.00393827 |
| **BORA** | 0.718513 | 1.08302 | 12.72729 | 1.34E-31 | 1.57E-29 | 60.97322574 |
| **DRAXIN** | 1.069136 | 1.177456 | 12.72255 | 1.40E-31 | 1.63E-29 | 60.92985116 |
| **GEN1** | 0.744518 | 1.126151 | 12.69433 | 1.82E-31 | 2.11E-29 | 60.6720349 |
| **KNSTRN** | 1.264664 | 2.660737 | 12.68565 | 1.97E-31 | 2.27E-29 | 60.59275493 |
| **BEND3** | 0.574104 | 0.86012 | 12.65449 | 2.63E-31 | 3.01E-29 | 60.30843165 |
| **ARHGAP11B** | 0.84336 | 0.867345 | 12.65118 | 2.71E-31 | 3.09E-29 | 60.27823814 |
| **MSH6** | 1.279057 | 3.133284 | 12.64397 | 2.89E-31 | 3.29E-29 | 60.21253306 |
| **RCC2** | 1.548925 | 3.98137 | 12.63492 | 3.15E-31 | 3.56E-29 | 60.13005161 |
| **ZNF300** | 1.355108 | 2.386291 | 12.63264 | 3.21E-31 | 3.61E-29 | 60.10922324 |
| **FKBP7** | 1.114387 | 1.954743 | 12.63228 | 3.22E-31 | 3.61E-29 | 60.10591562 |
| **SPC25** | 1.260137 | 1.766347 | 12.61706 | 3.71E-31 | 4.13E-29 | 59.96726434 |
| **TTI2** | 0.904701 | 2.234859 | 12.59339 | 4.61E-31 | 5.12E-29 | 59.75178641 |
| **MEX3A** | 1.482756 | 2.753119 | 12.59067 | 4.73E-31 | 5.22E-29 | 59.72701467 |
| **CBX8** | 1.074849 | 2.231604 | 12.56218 | 6.14E-31 | 6.75E-29 | 59.46787732 |
| **COL4A2** | 2.092908 | 3.586027 | 12.5464 | 7.10E-31 | 7.77E-29 | 59.32447134 |
| **C4orf46** | 0.890227 | 1.422178 | 12.5427 | 7.34E-31 | 8.00E-29 | 59.2909262 |
| **TMPO** | 1.507526 | 3.26035 | 12.52219 | 8.86E-31 | 9.57E-29 | 59.10466882 |
| **PRIM2** | 0.79187 | 1.224896 | 12.50868 | 1.00E-30 | 1.08E-28 | 58.98200501 |
| **MCM5** | 1.271842 | 3.773631 | 12.50758 | 1.01E-30 | 1.08E-28 | 58.97205226 |
| **CLSPN** | 0.793847 | 0.638339 | 12.49834 | 1.10E-30 | 1.17E-28 | 58.88821198 |
| **MMP2** | 1.569281 | 3.49254 | 12.46732 | 1.46E-30 | 1.55E-28 | 58.60705374 |
| **STIL** | 0.63874 | 0.676961 | 12.45784 | 1.60E-30 | 1.69E-28 | 58.52124378 |
| **LAMC1** | 1.49064 | 2.353916 | 12.45652 | 1.62E-30 | 1.70E-28 | 58.50927285 |
| **CCNJL** | 0.755015 | 1.052655 | 12.44968 | 1.72E-30 | 1.80E-28 | 58.44728044 |
| **KIF20B** | 0.638124 | 0.847296 | 12.44957 | 1.72E-30 | 1.80E-28 | 58.44633288 |
| **PGM2** | 1.217109 | 2.263917 | 12.43555 | 1.96E-30 | 2.03E-28 | 58.31941209 |
| **ZNF90** | 0.762914 | 1.171449 | 12.43299 | 2.00E-30 | 2.07E-28 | 58.29625139 |
| **RFC4** | 1.310242 | 3.62231 | 12.40363 | 2.62E-30 | 2.70E-28 | 58.03072937 |
| **DYRK2** | 1.112041 | 1.934793 | 12.39669 | 2.79E-30 | 2.86E-28 | 57.96800029 |
| **XRCC2** | 0.753838 | 0.706679 | 12.39492 | 2.84E-30 | 2.90E-28 | 57.95201069 |
| **TSKU** | 1.281883 | 2.708789 | 12.38236 | 3.18E-30 | 3.23E-28 | 57.83859701 |
| **RFWD3** | 1.044343 | 1.779944 | 12.38017 | 3.25E-30 | 3.29E-28 | 57.81884378 |
| **DHRSX** | 0.99233 | 2.47271 | 12.37289 | 3.47E-30 | 3.50E-28 | 57.75308582 |
| **SPPL2A** | 1.16234 | 2.769893 | 12.37067 | 3.54E-30 | 3.55E-28 | 57.73302391 |
| **COQ2** | 1.018237 | 2.501725 | 12.35786 | 3.98E-30 | 3.98E-28 | 57.6174104 |
| **ZNF764** | 0.95414 | 2.294015 | 12.33829 | 4.76E-30 | 4.73E-28 | 57.44091288 |
| **RAD54L** | 0.947314 | 1.529553 | 12.33602 | 4.86E-30 | 4.81E-28 | 57.42045453 |
| **LBR** | 1.365881 | 3.132386 | 12.3165 | 5.80E-30 | 5.72E-28 | 57.24451774 |
| **FEN1** | 1.23063 | 3.493438 | 12.31235 | 6.02E-30 | 5.92E-28 | 57.20716028 |
| **PDIA4** | 1.540177 | 4.175087 | 12.30572 | 6.40E-30 | 6.26E-28 | 57.14742471 |
| **TMEM194A** | 1.060835 | 1.768179 | 12.303 | 6.56E-30 | 6.39E-28 | 57.12298657 |
| **CTPS1** | 1.221837 | 2.938179 | 12.29056 | 7.34E-30 | 7.13E-28 | 57.01100804 |
| **ZNF625-ZNF20** | 0.617592 | 0.973885 | 12.28676 | 7.60E-30 | 7.35E-28 | 56.97675422 |
| **CLP1** | 0.972981 | 2.324801 | 12.28241 | 7.91E-30 | 7.62E-28 | 56.93760786 |
| **MAP3K1** | 1.060829 | 1.732275 | 12.281 | 8.01E-30 | 7.68E-28 | 56.92492384 |
| **FANCE** | 0.819851 | 1.805544 | 12.27333 | 8.59E-30 | 8.21E-28 | 56.85592232 |
| **BICD1** | 1.304513 | 2.503167 | 12.27112 | 8.76E-30 | 8.34E-28 | 56.83611685 |
| **RIT1** | 1.278237 | 2.983089 | 12.26361 | 9.38E-30 | 8.89E-28 | 56.76855044 |
| **BYSL** | 1.135286 | 3.149065 | 12.26165 | 9.55E-30 | 9.02E-28 | 56.7509233 |
| **TGFBR1** | 1.292365 | 2.669661 | 12.25982 | 9.71E-30 | 9.13E-28 | 56.73446187 |
| **EML4** | 1.096843 | 2.457886 | 12.24959 | 1.07E-29 | 9.98E-28 | 56.64250957 |
| **TDP1** | 0.976779 | 1.956176 | 12.24206 | 1.14E-29 | 1.06E-27 | 56.57486099 |
| **BMF** | 0.961327 | 1.588493 | 12.23621 | 1.20E-29 | 1.12E-27 | 56.52233605 |
| **CHIC2** | 1.312795 | 2.769661 | 12.22678 | 1.31E-29 | 1.21E-27 | 56.43767597 |
| **MCM4** | 1.792608 | 3.733032 | 12.22678 | 1.31E-29 | 1.21E-27 | 56.4376141 |
| **RNASEH2A** | 1.537501 | 4.319181 | 12.22537 | 1.33E-29 | 1.22E-27 | 56.42502429 |
| **HAUS1** | 1.24318 | 3.410925 | 12.20603 | 1.58E-29 | 1.45E-27 | 56.25147974 |
| **CDCA7L** | 1.283281 | 2.639851 | 12.20206 | 1.64E-29 | 1.50E-27 | 56.21582527 |
| **POLD3** | 0.951027 | 2.019377 | 12.17831 | 2.03E-29 | 1.85E-27 | 56.00287491 |
| **CDKAL1** | 0.932917 | 2.002652 | 12.17729 | 2.05E-29 | 1.86E-27 | 55.99373204 |
| **FRMD8** | 1.195464 | 3.563609 | 12.16512 | 2.29E-29 | 2.07E-27 | 55.88476769 |
| **NID1** | 1.511071 | 2.452529 | 12.16036 | 2.39E-29 | 2.15E-27 | 55.842086 |
| **RFT1** | 1.015243 | 2.597888 | 12.15605 | 2.49E-29 | 2.22E-27 | 55.80348741 |
| **PMM2** | 1.137068 | 2.382052 | 12.15594 | 2.49E-29 | 2.22E-27 | 55.80254459 |
| **CMTM3** | 1.601941 | 4.581554 | 12.1492 | 2.65E-29 | 2.35E-27 | 55.74218197 |
| **CD93** | 1.326881 | 1.590438 | 12.14648 | 2.71E-29 | 2.40E-27 | 55.71784916 |
| **C2orf44** | 0.784219 | 1.727268 | 12.14022 | 2.87E-29 | 2.53E-27 | 55.66185932 |
| **POLR2D** | 1.143384 | 2.841003 | 12.13847 | 2.92E-29 | 2.56E-27 | 55.64619096 |
| **HMGB2** | 1.967929 | 5.002706 | 12.12701 | 3.23E-29 | 2.82E-27 | 55.54368929 |
| **DDX11** | 1.300364 | 3.121827 | 12.12689 | 3.24E-29 | 2.82E-27 | 55.54254105 |
| **C10orf25** | 0.647635 | 1.257669 | 12.11901 | 3.48E-29 | 3.02E-27 | 55.47215692 |
| **PTBP1** | 1.596418 | 5.443378 | 12.10658 | 3.89E-29 | 3.37E-27 | 55.36105834 |
| **NIP7** | 1.093685 | 2.876891 | 12.10595 | 3.91E-29 | 3.37E-27 | 55.35539808 |
| **GMPS** | 1.080367 | 2.957085 | 12.10214 | 4.05E-29 | 3.48E-27 | 55.32137989 |
| **PIF1** | 1.085802 | 1.693383 | 12.09162 | 4.45E-29 | 3.81E-27 | 55.22733664 |
| **MGME1** | 1.119093 | 2.738998 | 12.08031 | 4.93E-29 | 4.21E-27 | 55.12641841 |
| **F2R** | 1.583062 | 2.959079 | 12.07093 | 5.36E-29 | 4.56E-27 | 55.04269769 |
| **EPHB2** | 1.139317 | 2.415963 | 12.05871 | 5.99E-29 | 5.07E-27 | 54.93365831 |
| **E2F3** | 1.130764 | 2.021917 | 12.05613 | 6.13E-29 | 5.18E-27 | 54.91066865 |
| **ETV6** | 0.843224 | 1.723366 | 12.0482 | 6.58E-29 | 5.54E-27 | 54.83994916 |
| **KIRREL** | 1.177393 | 1.788884 | 12.04738 | 6.63E-29 | 5.56E-27 | 54.83262216 |
| **PRIM1** | 1.061187 | 2.203429 | 12.04425 | 6.82E-29 | 5.70E-27 | 54.80471536 |
| **HN1L** | 1.270414 | 2.936641 | 12.032 | 7.62E-29 | 6.34E-27 | 54.69556687 |
| **GEMIN4** | 0.902438 | 2.272679 | 12.03123 | 7.67E-29 | 6.37E-27 | 54.68872805 |
| **ZNF646** | 0.950686 | 2.491632 | 12.01733 | 8.69E-29 | 7.18E-27 | 54.56488063 |
| **MIS18A** | 1.035441 | 2.298385 | 12.01705 | 8.72E-29 | 7.18E-27 | 54.56245194 |
| **TGFB1I1** | 1.348912 | 3.341116 | 12.01032 | 9.26E-29 | 7.59E-27 | 54.50245055 |
| **ZNF26** | 0.986781 | 2.414199 | 12.01015 | 9.27E-29 | 7.59E-27 | 54.50094209 |
| **GJC1** | 1.0192 | 1.507202 | 12.00615 | 9.61E-29 | 7.84E-27 | 54.46540494 |
| **PCNA** | 1.716361 | 4.832001 | 11.99936 | 1.02E-28 | 8.31E-27 | 54.40491875 |
| **TUBA1C** | 1.49807 | 3.067239 | 11.98388 | 1.17E-28 | 9.51E-27 | 54.26725093 |
| **NUP62** | 1.387107 | 3.757375 | 11.98171 | 1.20E-28 | 9.67E-27 | 54.24798495 |
| **UBTD2** | 1.18368 | 2.681091 | 11.96898 | 1.34E-28 | 1.08E-26 | 54.13483728 |
| **GABPB1** | 1.094641 | 2.068103 | 11.96797 | 1.35E-28 | 1.09E-26 | 54.1258284 |
| **IQGAP2** | 1.229901 | 1.532072 | 11.94848 | 1.61E-28 | 1.29E-26 | 53.95269475 |
| **DCLRE1B** | 0.89523 | 1.486536 | 11.93205 | 1.87E-28 | 1.49E-26 | 53.80686141 |
| **SDC1** | 1.014139 | 1.336942 | 11.92615 | 1.97E-28 | 1.56E-26 | 53.75449778 |
| **MMP14** | 1.773827 | 3.788923 | 11.92603 | 1.97E-28 | 1.56E-26 | 53.75343761 |
| **DCAF13** | 1.348115 | 3.355231 | 11.92418 | 2.01E-28 | 1.58E-26 | 53.73702219 |
| **ZNF486** | 0.77496 | 1.178853 | 11.91381 | 2.20E-28 | 1.73E-26 | 53.64505801 |
| **THG1L** | 0.820489 | 2.057116 | 11.91271 | 2.22E-28 | 1.74E-26 | 53.63530368 |
| **IFNAR2** | 1.106434 | 3.160552 | 11.91086 | 2.26E-28 | 1.77E-26 | 53.61895474 |
| **RPIA** | 1.035309 | 2.817052 | 11.90523 | 2.38E-28 | 1.85E-26 | 53.5690438 |
| **FAM60A** | 1.394406 | 2.350433 | 11.90303 | 2.43E-28 | 1.88E-26 | 53.54950015 |
| **NRAS** | 1.222207 | 2.932298 | 11.8997 | 2.50E-28 | 1.93E-26 | 53.52000812 |
| **SERPINB8** | 0.987949 | 1.70362 | 11.88187 | 2.93E-28 | 2.26E-26 | 53.36206686 |
| **LDLRAD3** | 1.330131 | 3.575585 | 11.88139 | 2.94E-28 | 2.26E-26 | 53.35780286 |
| **CD276** | 1.368632 | 4.36075 | 11.86154 | 3.52E-28 | 2.69E-26 | 53.18211282 |
| **UCK2** | 1.275695 | 3.696033 | 11.86039 | 3.55E-28 | 2.71E-26 | 53.17190934 |
| **CMTM6** | 1.281362 | 2.662544 | 11.85515 | 3.72E-28 | 2.83E-26 | 53.125552 |
| **ELAVL1** | 1.402846 | 3.86006 | 11.85513 | 3.72E-28 | 2.83E-26 | 53.12540099 |
| **EFNB1** | 1.076572 | 2.812205 | 11.84989 | 3.90E-28 | 2.95E-26 | 53.07902171 |
| **NUP155** | 0.891388 | 2.019698 | 11.84033 | 4.25E-28 | 3.20E-26 | 52.99454554 |
| **C18orf54** | 0.628261 | 0.963849 | 11.83447 | 4.48E-28 | 3.37E-26 | 52.94271213 |
| **RP5-991G20.4** | 0.512924 | 0.909309 | 11.83043 | 4.64E-28 | 3.48E-26 | 52.90704849 |
| **VASP** | 1.366558 | 3.67811 | 11.8291 | 4.70E-28 | 3.51E-26 | 52.89531055 |
| **SKP2** | 0.903243 | 1.876975 | 11.81505 | 5.33E-28 | 3.97E-26 | 52.77119414 |
| **SIX1** | 1.002682 | 1.143691 | 11.80874 | 5.64E-28 | 4.18E-26 | 52.71544932 |
| **GMEB1** | 0.800802 | 1.647505 | 11.80775 | 5.68E-28 | 4.21E-26 | 52.70673275 |
| **VANGL1** | 0.620814 | 0.819654 | 11.80487 | 5.83E-28 | 4.30E-26 | 52.6813551 |
| **PFAS** | 1.209369 | 3.262018 | 11.80471 | 5.84E-28 | 4.30E-26 | 52.67993365 |
| **SOX4** | 1.990851 | 4.906706 | 11.79644 | 6.29E-28 | 4.61E-26 | 52.60689306 |
| **CKAP4** | 1.20052 | 3.464378 | 11.79609 | 6.31E-28 | 4.61E-26 | 52.60387483 |
| **PRR11** | 1.025679 | 1.282235 | 11.78631 | 6.88E-28 | 5.02E-26 | 52.51753985 |
| **CHST14** | 1.278525 | 3.000962 | 11.77897 | 7.35E-28 | 5.34E-26 | 52.45284179 |
| **ZNF800** | 1.016695 | 2.412931 | 11.77793 | 7.42E-28 | 5.37E-26 | 52.44363293 |
| **CAPN5** | 1.521186 | 3.536273 | 11.77652 | 7.51E-28 | 5.42E-26 | 52.43127681 |
| **HMGB3** | 1.451488 | 4.251563 | 11.76578 | 8.26E-28 | 5.95E-26 | 52.33655433 |
| **FBLIM1** | 1.521099 | 2.203992 | 11.75775 | 8.88E-28 | 6.37E-26 | 52.26582254 |
| **URB2** | 0.812464 | 1.422525 | 11.74838 | 9.65E-28 | 6.89E-26 | 52.18327625 |
| **IL10RB** | 1.120769 | 2.996233 | 11.74827 | 9.66E-28 | 6.89E-26 | 52.18236138 |
| **ZNF695** | 0.595383 | 0.713696 | 11.74518 | 9.93E-28 | 7.06E-26 | 52.15516918 |
| **NOP14** | 0.97571 | 2.830667 | 11.73188 | 1.12E-27 | 7.93E-26 | 52.03810698 |
| **MMS22L** | 0.699454 | 1.096474 | 11.73042 | 1.13E-27 | 8.01E-26 | 52.02520247 |
| **MYCBP** | 0.886186 | 2.000258 | 11.72914 | 1.14E-27 | 8.07E-26 | 52.0139803 |
| **LEPRE1** | 1.194582 | 3.681615 | 11.70799 | 1.38E-27 | 9.69E-26 | 51.8279953 |
| **ZNF496** | 1.139825 | 3.420314 | 11.70678 | 1.40E-27 | 9.76E-26 | 51.8173635 |
| **MCAM** | 1.65364 | 3.763047 | 11.70066 | 1.47E-27 | 1.03E-25 | 51.76356022 |
| **TIPIN** | 0.963176 | 2.283075 | 11.69582 | 1.54E-27 | 1.07E-25 | 51.72102729 |
| **ZC3HAV1** | 1.008494 | 2.286756 | 11.69475 | 1.55E-27 | 1.08E-25 | 51.71158588 |
| **SMARCD1** | 1.535489 | 4.350207 | 11.69428 | 1.56E-27 | 1.08E-25 | 51.70750173 |
| **TCEB3** | 1.096661 | 2.858478 | 11.69166 | 1.60E-27 | 1.10E-25 | 51.68442611 |
| **ZNF669** | 0.839615 | 1.637721 | 11.68759 | 1.66E-27 | 1.14E-25 | 51.64871259 |
| **ITGB1** | 1.803306 | 4.455815 | 11.68005 | 1.77E-27 | 1.21E-25 | 51.58247014 |
| **GDAP2** | 0.612489 | 1.294568 | 11.66987 | 1.94E-27 | 1.32E-25 | 51.49311408 |
| **ATAD5** | 0.503563 | 0.668595 | 11.66553 | 2.01E-27 | 1.37E-25 | 51.45508474 |
| **E2F7** | 0.502129 | 0.374909 | 11.66008 | 2.11E-27 | 1.44E-25 | 51.40723619 |
| **ZBED4** | 0.86343 | 1.743713 | 11.65754 | 2.16E-27 | 1.46E-25 | 51.38494926 |
| **MEST** | 1.666925 | 4.019712 | 11.64375 | 2.44E-27 | 1.65E-25 | 51.26407806 |
| **MIDN** | 1.600198 | 5.190418 | 11.63824 | 2.57E-27 | 1.73E-25 | 51.21576842 |
| **POLR1B** | 1.170745 | 2.390265 | 11.63444 | 2.65E-27 | 1.78E-25 | 51.18243488 |
| **ATP7A** | 0.599759 | 1.105755 | 11.62563 | 2.87E-27 | 1.92E-25 | 51.10526752 |
| **APEX2** | 0.908114 | 2.548249 | 11.62123 | 2.98E-27 | 1.99E-25 | 51.06673018 |
| **ITGB3BP** | 1.152516 | 2.867132 | 11.61918 | 3.04E-27 | 2.02E-25 | 51.04881962 |
| **XXYLT1** | 1.177573 | 2.449716 | 11.61842 | 3.06E-27 | 2.03E-25 | 51.04216154 |
| **SIPA1L3** | 1.049661 | 2.460189 | 11.61653 | 3.11E-27 | 2.06E-25 | 51.02562188 |
| **TTC27** | 0.853904 | 2.126793 | 11.61372 | 3.19E-27 | 2.11E-25 | 51.00099153 |
| **AC099522.1** | 1.477191 | 2.053474 | 11.61221 | 3.23E-27 | 2.13E-25 | 50.9877886 |
| **DTX3L** | 0.976418 | 2.044744 | 11.60903 | 3.32E-27 | 2.18E-25 | 50.95999056 |
| **ZSWIM4** | 1.254609 | 2.323506 | 11.60492 | 3.45E-27 | 2.26E-25 | 50.92401583 |
| **CEP135** | 0.77992 | 1.072895 | 11.59535 | 3.75E-27 | 2.45E-25 | 50.84025567 |
| **PLAGL2** | 0.786206 | 1.485417 | 11.59178 | 3.87E-27 | 2.52E-25 | 50.80905822 |
| **TOPBP1** | 1.289682 | 2.699697 | 11.59147 | 3.88E-27 | 2.52E-25 | 50.80628532 |
| **NCAPD3** | 1.264039 | 2.806822 | 11.59059 | 3.91E-27 | 2.53E-25 | 50.79865788 |
| **ZNF79** | 0.655579 | 1.54632 | 11.58764 | 4.01E-27 | 2.59E-25 | 50.77280021 |
| **STK17A** | 1.297835 | 2.915481 | 11.58122 | 4.25E-27 | 2.74E-25 | 50.71672943 |
| **NFIL3** | 1.188117 | 2.508881 | 11.56804 | 4.77E-27 | 3.07E-25 | 50.60154847 |
| **ODF2** | 1.270388 | 3.514335 | 11.5632 | 4.98E-27 | 3.19E-25 | 50.55922729 |
| **SNHG16** | 1.263588 | 4.070307 | 11.56215 | 5.03E-27 | 3.22E-25 | 50.55004538 |
| **PKN3** | 0.806242 | 1.771263 | 11.55579 | 5.32E-27 | 3.39E-25 | 50.49453359 |
| **PIK3R3** | 1.035014 | 2.511942 | 11.55327 | 5.44E-27 | 3.46E-25 | 50.47248432 |
| **INTS9** | 0.991986 | 2.7986 | 11.5457 | 5.81E-27 | 3.69E-25 | 50.40644026 |
| **GINS4** | 0.985545 | 1.39681 | 11.5428 | 5.96E-27 | 3.77E-25 | 50.38109806 |
| **DSEL** | 1.138279 | 2.203069 | 11.54209 | 6.00E-27 | 3.79E-25 | 50.37496553 |
| **GLE1** | 1.007184 | 3.034859 | 11.53981 | 6.12E-27 | 3.85E-25 | 50.35506666 |
| **SOGA1** | 1.112066 | 3.206631 | 11.5363 | 6.32E-27 | 3.97E-25 | 50.32438889 |
| **EHD4** | 1.156228 | 2.167407 | 11.52979 | 6.69E-27 | 4.19E-25 | 50.26760179 |
| **GRIK3** | 1.531772 | 3.19829 | 11.51584 | 7.57E-27 | 4.72E-25 | 50.14601612 |
| **TP53INP1** | 1.035089 | 2.15978 | 11.51547 | 7.59E-27 | 4.73E-25 | 50.14274011 |
| **ZBTB9** | 0.913857 | 2.370196 | 11.51306 | 7.75E-27 | 4.82E-25 | 50.12173385 |
| **NANP** | 0.555488 | 0.96093 | 11.51258 | 7.79E-27 | 4.82E-25 | 50.11758913 |
| **DEK** | 1.5367 | 4.01762 | 11.50496 | 8.33E-27 | 5.15E-25 | 50.05116173 |
| **LYPLA1** | 1.33283 | 3.077324 | 11.50408 | 8.39E-27 | 5.17E-25 | 50.04353889 |
| **CARHSP1** | 1.379568 | 4.497234 | 11.50069 | 8.65E-27 | 5.32E-25 | 50.01399971 |
| **ZNF689** | 0.839847 | 2.07623 | 11.48341 | 1.01E-26 | 6.17E-25 | 49.86353767 |
| **KNTC1** | 1.209499 | 2.547168 | 11.47758 | 1.06E-26 | 6.48E-25 | 49.81275508 |
| **MYD88** | 1.27143 | 2.374239 | 11.47415 | 1.09E-26 | 6.66E-25 | 49.78294116 |
| **TMEM255A** | 1.486297 | 3.032772 | 11.47212 | 1.11E-26 | 6.77E-25 | 49.76524902 |
| **C21orf58** | 0.930089 | 2.085893 | 11.47025 | 1.13E-26 | 6.86E-25 | 49.74905321 |
| **ALDH18A1** | 1.276071 | 3.750814 | 11.4696 | 1.14E-26 | 6.88E-25 | 49.74340365 |
| **POLG2** | 1.005673 | 2.621518 | 11.46279 | 1.21E-26 | 7.28E-25 | 49.68413377 |
| **RP11-73E17.2** | 0.788981 | 1.565819 | 11.46263 | 1.21E-26 | 7.28E-25 | 49.68278425 |
| **CANT1** | 1.283682 | 3.680391 | 11.46068 | 1.23E-26 | 7.39E-25 | 49.66582898 |
| **CDK4** | 1.832463 | 6.151173 | 11.45807 | 1.26E-26 | 7.54E-25 | 49.64313159 |
| **CHAMP1** | 1.017512 | 2.552834 | 11.45508 | 1.29E-26 | 7.72E-25 | 49.61707715 |
| **UBXN2A** | 0.923963 | 2.540287 | 11.45203 | 1.33E-26 | 7.91E-25 | 49.59058642 |
| **SMARCE1** | 1.582899 | 4.665947 | 11.45057 | 1.34E-26 | 7.98E-25 | 49.57791932 |
| **FN1** | 1.942708 | 5.343146 | 11.45054 | 1.34E-26 | 7.98E-25 | 49.57767331 |
| **C1orf112** | 0.836278 | 1.556092 | 11.44473 | 1.41E-26 | 8.37E-25 | 49.52718594 |
| **ZNF3** | 1.252524 | 3.621405 | 11.44319 | 1.43E-26 | 8.47E-25 | 49.51378059 |
| **EXO5** | 0.918617 | 1.987054 | 11.43912 | 1.49E-26 | 8.75E-25 | 49.47841103 |
| **NAA38** | 1.256908 | 3.652134 | 11.43715 | 1.51E-26 | 8.88E-25 | 49.46132423 |
| **GMIP** | 1.139732 | 3.06552 | 11.43574 | 1.53E-26 | 8.97E-25 | 49.44906882 |
| **IDH1** | 1.575979 | 4.425152 | 11.42906 | 1.62E-26 | 9.49E-25 | 49.39103953 |
| **TOP3A** | 1.135965 | 3.752509 | 11.42819 | 1.64E-26 | 9.54E-25 | 49.38351899 |
| **SF3A3** | 1.357344 | 4.092221 | 11.42758 | 1.64E-26 | 9.57E-25 | 49.37823288 |
| **ZNF124** | 0.670505 | 1.639334 | 11.40223 | 2.05E-26 | 1.19E-24 | 49.15827258 |
| **RRM1** | 1.342728 | 3.234975 | 11.40035 | 2.09E-26 | 1.21E-24 | 49.14198391 |
| **NUP37** | 0.924302 | 2.673768 | 11.3987 | 2.12E-26 | 1.22E-24 | 49.12768346 |
| **ZNF200** | 0.797553 | 1.719522 | 11.3983 | 2.13E-26 | 1.22E-24 | 49.12415713 |
| **MYBL1** | 0.797909 | 1.381748 | 11.38913 | 2.30E-26 | 1.32E-24 | 49.04472809 |
| **TCF3** | 1.698913 | 5.184376 | 11.38085 | 2.48E-26 | 1.42E-24 | 48.97297254 |
| **KATNA1** | 0.894857 | 2.796397 | 11.38056 | 2.48E-26 | 1.42E-24 | 48.97041438 |
| **AFAP1L1** | 0.936074 | 1.462943 | 11.37874 | 2.52E-26 | 1.44E-24 | 48.95470781 |
| **DDX20** | 0.858373 | 1.773065 | 11.37788 | 2.54E-26 | 1.44E-24 | 48.94724965 |
| **NID2** | 1.183698 | 1.893736 | 11.3726 | 2.66E-26 | 1.51E-24 | 48.90147374 |
| **POLR3D** | 1.133554 | 3.254866 | 11.37145 | 2.69E-26 | 1.52E-24 | 48.89151841 |
| **PSMC3IP** | 1.0967 | 2.612559 | 11.37031 | 2.72E-26 | 1.53E-24 | 48.88168617 |
| **LIMA1** | 1.652505 | 3.88899 | 11.36511 | 2.84E-26 | 1.60E-24 | 48.83667655 |
| **TOE1** | 0.835409 | 2.280419 | 11.3649 | 2.85E-26 | 1.60E-24 | 48.8348512 |
| **NDE1** | 1.354316 | 2.672965 | 11.36365 | 2.88E-26 | 1.61E-24 | 48.82405307 |
| **HSPG2** | 1.519026 | 3.066067 | 11.36148 | 2.93E-26 | 1.64E-24 | 48.80525134 |
| **FSTL1** | 1.619742 | 3.243293 | 11.36112 | 2.94E-26 | 1.64E-24 | 48.80210625 |
| **HELZ2** | 1.10203 | 2.077574 | 11.35838 | 3.02E-26 | 1.68E-24 | 48.77840248 |
| **PRPF4** | 1.073441 | 2.937022 | 11.35452 | 3.12E-26 | 1.73E-24 | 48.74500739 |
| **DOT1L** | 1.316994 | 3.698138 | 11.35337 | 3.15E-26 | 1.74E-24 | 48.73504858 |
| **TOR1B** | 1.202864 | 3.148288 | 11.3503 | 3.24E-26 | 1.79E-24 | 48.70850509 |
| **SPSB4** | 1.246238 | 2.79469 | 11.34925 | 3.27E-26 | 1.80E-24 | 48.69943656 |
| **CEP57L1** | 0.894641 | 2.174328 | 11.34845 | 3.29E-26 | 1.80E-24 | 48.69251798 |
| **ACTL6A** | 1.478764 | 3.760711 | 11.3484 | 3.29E-26 | 1.80E-24 | 48.69204248 |
| **LOXL2** | 1.387146 | 2.035991 | 11.34823 | 3.29E-26 | 1.80E-24 | 48.69061647 |
| **KDM4A** | 1.218173 | 2.995729 | 11.34591 | 3.36E-26 | 1.84E-24 | 48.67056267 |
| **ATP6V0A2** | 0.971972 | 2.351137 | 11.34484 | 3.39E-26 | 1.85E-24 | 48.66123502 |
| **RP3-428L16.2** | 1.422737 | 2.82999 | 11.34379 | 3.43E-26 | 1.86E-24 | 48.65217816 |
| **ISG20L2** | 1.119065 | 3.007549 | 11.34216 | 3.47E-26 | 1.88E-24 | 48.6380897 |
| **C12orf4** | 1.021564 | 2.603084 | 11.34089 | 3.51E-26 | 1.90E-24 | 48.62714855 |
| **BCL6B** | 1.010176 | 1.705582 | 11.3369 | 3.64E-26 | 1.96E-24 | 48.59260413 |
| **RAI14** | 1.06732 | 2.090112 | 11.3347 | 3.71E-26 | 2.00E-24 | 48.57357809 |
| **SLC26A2** | 0.803856 | 1.119179 | 11.33413 | 3.73E-26 | 2.00E-24 | 48.56864187 |
| **LCORL** | 0.815605 | 1.674421 | 11.33108 | 3.83E-26 | 2.05E-24 | 48.54234074 |
| **MTPAP** | 0.94479 | 2.294636 | 11.32952 | 3.88E-26 | 2.08E-24 | 48.52881308 |
| **ASXL1** | 1.165343 | 3.792079 | 11.32362 | 4.09E-26 | 2.18E-24 | 48.47782256 |
| **ZNF286B** | 0.961645 | 1.806947 | 11.32059 | 4.20E-26 | 2.24E-24 | 48.45165851 |
| **STK10** | 1.101485 | 3.207843 | 11.3188 | 4.26E-26 | 2.27E-24 | 48.43623024 |
| **C4orf21** | 0.820624 | 1.478434 | 11.31537 | 4.39E-26 | 2.33E-24 | 48.40659884 |
| **ITPRIPL1** | 0.747493 | 0.875155 | 11.31398 | 4.44E-26 | 2.35E-24 | 48.39453591 |
| **EFNA2** | 1.139445 | 2.09983 | 11.29618 | 5.19E-26 | 2.73E-24 | 48.24090856 |
| **ZNF649** | 1.13705 | 2.227152 | 11.28199 | 5.88E-26 | 3.08E-24 | 48.11848048 |
| **BACE2** | 0.95627 | 1.963278 | 11.27867 | 6.05E-26 | 3.17E-24 | 48.08986985 |
| **FAM105B** | 0.797708 | 1.810097 | 11.27836 | 6.06E-26 | 3.17E-24 | 48.08723194 |
| **DNAAF2** | 0.951041 | 2.219636 | 11.27563 | 6.21E-26 | 3.24E-24 | 48.06364726 |
| **TGS1** | 0.907549 | 1.949366 | 11.27297 | 6.36E-26 | 3.31E-24 | 48.04077288 |
| **SPDL1** | 1.122494 | 2.284706 | 11.27248 | 6.38E-26 | 3.31E-24 | 48.03648831 |
| **ZBTB12** | 1.244479 | 2.869542 | 11.26479 | 6.83E-26 | 3.53E-24 | 47.97026823 |
| **MYO1C** | 1.093964 | 3.116842 | 11.26415 | 6.86E-26 | 3.54E-24 | 47.96472771 |
| **STK38** | 0.961032 | 2.642537 | 11.2586 | 7.20E-26 | 3.71E-24 | 47.91690168 |
| **BTN2A2** | 1.12552 | 2.122906 | 11.25526 | 7.42E-26 | 3.81E-24 | 47.8881902 |
| **CEP89** | 0.946569 | 2.83823 | 11.25491 | 7.44E-26 | 3.81E-24 | 47.88518083 |
| **CNIH4** | 1.316823 | 4.159271 | 11.25426 | 7.48E-26 | 3.83E-24 | 47.87956278 |
| **KLF10** | 1.181097 | 2.189089 | 11.25382 | 7.51E-26 | 3.83E-24 | 47.87579526 |
| **RHNO1** | 1.18257 | 3.369936 | 11.24929 | 7.81E-26 | 3.98E-24 | 47.83675839 |
| **ITGA5** | 1.18663 | 2.348691 | 11.24109 | 8.39E-26 | 4.26E-24 | 47.76619891 |
| **B4GALT5** | 1.389239 | 3.262545 | 11.24034 | 8.45E-26 | 4.28E-24 | 47.75969691 |
| **FAM57A** | 1.154749 | 3.302211 | 11.23917 | 8.53E-26 | 4.32E-24 | 47.74966112 |
| **SMC2** | 1.127522 | 2.026645 | 11.23734 | 8.67E-26 | 4.38E-24 | 47.73390248 |
| **UGGT1** | 1.008681 | 2.626645 | 11.23343 | 8.97E-26 | 4.52E-24 | 47.70026932 |
| **SEPN1** | 1.484614 | 4.340696 | 11.22984 | 9.25E-26 | 4.65E-24 | 47.66938085 |
| **RBM15B** | 1.13301 | 3.191994 | 11.22764 | 9.43E-26 | 4.73E-24 | 47.65041808 |
| **NUP188** | 1.225671 | 3.295997 | 11.22565 | 9.60E-26 | 4.80E-24 | 47.6333506 |
| **MAPK7** | 1.298681 | 3.479583 | 11.22372 | 9.76E-26 | 4.88E-24 | 47.61672008 |
| **TANC1** | 1.296904 | 2.906902 | 11.22075 | 1.00E-25 | 4.99E-24 | 47.59119228 |
| **C2orf68** | 1.203036 | 3.807736 | 11.21476 | 1.06E-25 | 5.25E-24 | 47.53973445 |
| **MAK16** | 0.886121 | 2.132104 | 11.21229 | 1.08E-25 | 5.35E-24 | 47.51847237 |
| **EPB41** | 1.166407 | 2.245546 | 11.20771 | 1.12E-25 | 5.56E-24 | 47.47914108 |
| **TEX10** | 1.011444 | 2.889863 | 11.20421 | 1.16E-25 | 5.72E-24 | 47.4490103 |
| **ZNF20** | 0.594667 | 1.060082 | 11.20152 | 1.18E-25 | 5.84E-24 | 47.42595385 |
| **COLGALT1** | 1.3749 | 4.364475 | 11.19668 | 1.23E-25 | 6.08E-24 | 47.3843473 |
| **RP11-698N11.2** | 0.98245 | 1.333233 | 11.18638 | 1.35E-25 | 6.63E-24 | 47.29587478 |
| **BFAR** | 1.205956 | 3.627886 | 11.18408 | 1.38E-25 | 6.75E-24 | 47.2761829 |
| **CCDC50** | 1.322461 | 3.147304 | 11.18376 | 1.38E-25 | 6.76E-24 | 47.27341657 |
| **ATF5** | 1.17432 | 3.261333 | 11.18236 | 1.40E-25 | 6.83E-24 | 47.26144745 |
| **CCND1** | 1.672982 | 4.887598 | 11.17814 | 1.45E-25 | 7.07E-24 | 47.22516311 |
| **CTD-2201E18.3** | 0.606609 | 1.156921 | 11.17279 | 1.52E-25 | 7.39E-24 | 47.17929353 |
| **GNAI3** | 1.353726 | 3.578374 | 11.16397 | 1.64E-25 | 7.96E-24 | 47.10365105 |
| **GALNT1** | 1.326086 | 3.429728 | 11.16081 | 1.69E-25 | 8.16E-24 | 47.07656585 |
| **SYDE1** | 1.166012 | 2.806606 | 11.159 | 1.71E-25 | 8.26E-24 | 47.06104604 |
| **SFMBT1** | 0.726144 | 1.625153 | 11.15885 | 1.71E-25 | 8.26E-24 | 47.05978349 |
| **CREB5** | 1.3062 | 2.827751 | 11.15883 | 1.71E-25 | 8.26E-24 | 47.05954802 |
| **CALU** | 1.588328 | 3.854935 | 11.15839 | 1.72E-25 | 8.27E-24 | 47.05578868 |
| **DKC1** | 1.361801 | 3.762264 | 11.155 | 1.77E-25 | 8.50E-24 | 47.02676457 |
| **AGBL5** | 1.035601 | 3.211617 | 11.15102 | 1.83E-25 | 8.78E-24 | 46.99263504 |
| **CASP3** | 1.302335 | 3.16032 | 11.15047 | 1.84E-25 | 8.80E-24 | 46.98790703 |
| **BEND7** | 1.11428 | 2.584542 | 11.14565 | 1.92E-25 | 9.16E-24 | 46.94666204 |
| **MED20** | 1.101234 | 2.584172 | 11.14492 | 1.93E-25 | 9.20E-24 | 46.9403848 |
| **STK40** | 1.124846 | 3.456914 | 11.13876 | 2.04E-25 | 9.69E-24 | 46.88758971 |
| **BAZ1A** | 1.125801 | 2.278104 | 11.13592 | 2.09E-25 | 9.91E-24 | 46.86326896 |
| **DERL1** | 1.236993 | 3.843642 | 11.13532 | 2.10E-25 | 9.94E-24 | 46.85811334 |
| **RP11-705C15.2** | 0.739603 | 1.595836 | 11.13455 | 2.12E-25 | 9.99E-24 | 46.85157435 |
| **ZNF829** | 0.531038 | 0.895719 | 11.13133 | 2.18E-25 | 1.02E-23 | 46.82395157 |
| **ZNF726** | 0.797514 | 1.47928 | 11.1263 | 2.27E-25 | 1.07E-23 | 46.78096276 |
| **POFUT1** | 1.08086 | 3.105643 | 11.12606 | 2.28E-25 | 1.07E-23 | 46.77891257 |
| **POP1** | 0.590791 | 1.21818 | 11.12162 | 2.37E-25 | 1.11E-23 | 46.74088768 |
| **ETS1** | 1.546872 | 3.126922 | 11.12135 | 2.37E-25 | 1.11E-23 | 46.73857548 |
| **OSTC** | 1.501689 | 4.285915 | 11.12127 | 2.37E-25 | 1.11E-23 | 46.73785962 |
| **DCBLD2** | 0.983325 | 2.025021 | 11.11983 | 2.40E-25 | 1.12E-23 | 46.72555476 |
| **ALG6** | 0.861844 | 2.237107 | 11.11818 | 2.44E-25 | 1.13E-23 | 46.71143784 |
| **MED26** | 0.901663 | 2.456771 | 11.1176 | 2.45E-25 | 1.13E-23 | 46.70651448 |
| **POLR1A** | 0.957597 | 2.427755 | 11.11748 | 2.45E-25 | 1.13E-23 | 46.70544716 |
| **SLC30A6** | 0.784666 | 1.978583 | 11.11746 | 2.45E-25 | 1.13E-23 | 46.7052676 |
| **CBX5** | 1.332753 | 3.968031 | 11.11701 | 2.46E-25 | 1.14E-23 | 46.70140444 |
| **PLVAP** | 1.501124 | 2.75724 | 11.11618 | 2.48E-25 | 1.14E-23 | 46.69436095 |
| **IRF1** | 1.205181 | 2.570421 | 11.11079 | 2.60E-25 | 1.19E-23 | 46.64822961 |
| **ZNF606** | 1.133995 | 2.649929 | 11.10895 | 2.64E-25 | 1.21E-23 | 46.63250349 |
| **DOCK11** | 0.74048 | 1.163381 | 11.10125 | 2.82E-25 | 1.29E-23 | 46.56665105 |
| **MEX3B** | 1.118832 | 1.94628 | 11.10091 | 2.83E-25 | 1.29E-23 | 46.56372691 |
| **DCP2** | 0.740367 | 2.346542 | 11.1 | 2.85E-25 | 1.30E-23 | 46.55600637 |
| **TFDP2** | 1.128479 | 3.052352 | 11.09711 | 2.93E-25 | 1.33E-23 | 46.53127171 |
| **HAUS8** | 1.053466 | 2.841713 | 11.09626 | 2.95E-25 | 1.34E-23 | 46.52404565 |
| **BCL10** | 0.704819 | 1.559038 | 11.09609 | 2.95E-25 | 1.34E-23 | 46.52253446 |
| **TNFRSF12A** | 1.843303 | 3.384459 | 11.09587 | 2.96E-25 | 1.34E-23 | 46.52068589 |
| **PDCD4-AS1** | 0.805856 | 1.803933 | 11.0956 | 2.96E-25 | 1.34E-23 | 46.51834952 |
| **PGGT1B** | 1.019943 | 2.284662 | 11.09261 | 3.04E-25 | 1.37E-23 | 46.49285598 |
| **IPO4** | 1.04104 | 2.946046 | 11.08757 | 3.18E-25 | 1.43E-23 | 46.44975563 |
| **AMMECR1L** | 0.940822 | 2.377431 | 11.08723 | 3.19E-25 | 1.43E-23 | 46.44682716 |
| **ZNF766** | 0.751988 | 2.045797 | 11.0839 | 3.28E-25 | 1.47E-23 | 46.41841432 |
| **ZNF92** | 1.103854 | 2.183266 | 11.08326 | 3.30E-25 | 1.48E-23 | 46.41290191 |
| **BZW2** | 1.294525 | 3.500611 | 11.08175 | 3.34E-25 | 1.49E-23 | 46.40001604 |
| **VEGFA** | 1.974011 | 4.369446 | 11.0781 | 3.45E-25 | 1.54E-23 | 46.368883 |
| **HNRNPAB** | 1.611657 | 5.837506 | 11.07705 | 3.48E-25 | 1.55E-23 | 46.35986461 |
| **IL10RB-AS1** | 0.863714 | 2.355708 | 11.07686 | 3.48E-25 | 1.55E-23 | 46.3582603 |
| **MSANTD3** | 1.102692 | 3.358432 | 11.07584 | 3.52E-25 | 1.56E-23 | 46.34958169 |
| **INCENP** | 1.204173 | 2.427243 | 11.07532 | 3.53E-25 | 1.56E-23 | 46.34512535 |
| **TXLNA** | 1.089244 | 3.403025 | 11.07475 | 3.55E-25 | 1.57E-23 | 46.34025546 |
| **TBC1D16** | 1.13763 | 3.286206 | 11.07325 | 3.59E-25 | 1.58E-23 | 46.32747019 |
| **SENP1** | 0.78765 | 1.572303 | 11.0721 | 3.63E-25 | 1.59E-23 | 46.31763873 |
| **SLC16A1** | 1.693101 | 3.895005 | 11.07209 | 3.63E-25 | 1.59E-23 | 46.31756497 |
| **DARS2** | 0.852274 | 1.763613 | 11.06913 | 3.73E-25 | 1.63E-23 | 46.29231639 |
| **ADAMTS7** | 1.022088 | 1.477209 | 11.06692 | 3.80E-25 | 1.66E-23 | 46.27342119 |
| **HAUS3** | 0.969932 | 2.282178 | 11.06225 | 3.95E-25 | 1.72E-23 | 46.23354379 |
| **BTG3** | 1.423029 | 3.70856 | 11.06222 | 3.95E-25 | 1.72E-23 | 46.23335123 |
| **ZNF670** | 0.690241 | 1.124101 | 11.05983 | 4.04E-25 | 1.76E-23 | 46.21297323 |
| **WDR4** | 0.950928 | 2.548687 | 11.05929 | 4.06E-25 | 1.76E-23 | 46.20830862 |
| **NOL10** | 0.91864 | 2.724319 | 11.05433 | 4.23E-25 | 1.84E-23 | 46.16601231 |
| **GATAD2A** | 1.486127 | 3.976501 | 11.05199 | 4.32E-25 | 1.86E-23 | 46.1460498 |
| **ZNF43** | 1.04324 | 2.121445 | 11.05186 | 4.32E-25 | 1.86E-23 | 46.14494242 |
| **NONO** | 1.627537 | 6.240673 | 11.05166 | 4.33E-25 | 1.86E-23 | 46.14320568 |
| **CSNK1G1** | 0.575618 | 1.554315 | 11.05054 | 4.37E-25 | 1.88E-23 | 46.13372744 |
| **POLR3C** | 1.014957 | 3.07418 | 11.04714 | 4.50E-25 | 1.93E-23 | 46.10469146 |
| **RNPEP** | 1.263059 | 3.892573 | 11.04669 | 4.52E-25 | 1.93E-23 | 46.10089951 |
| **EPHB3** | 1.336681 | 3.295726 | 11.04657 | 4.53E-25 | 1.93E-23 | 46.0998408 |
| **PCDH18** | 0.971503 | 1.464535 | 11.04608 | 4.54E-25 | 1.94E-23 | 46.09564123 |
| **PPM1D** | 1.123835 | 1.985113 | 11.04507 | 4.58E-25 | 1.95E-23 | 46.08703463 |
| **ZKSCAN4** | 0.779787 | 1.597435 | 11.04491 | 4.59E-25 | 1.95E-23 | 46.08566271 |
| **XPO5** | 1.015838 | 3.413348 | 11.04275 | 4.68E-25 | 1.98E-23 | 46.06726533 |
| **WHSC1** | 1.249959 | 3.618372 | 11.03266 | 5.10E-25 | 2.16E-23 | 45.98131098 |
| **PAXIP1** | 0.892625 | 1.907872 | 11.03249 | 5.11E-25 | 2.16E-23 | 45.97986586 |
| **NOP2** | 1.265195 | 4.048447 | 11.03152 | 5.15E-25 | 2.18E-23 | 45.97157322 |
| **ZNF35** | 0.723567 | 1.670732 | 11.02361 | 5.52E-25 | 2.32E-23 | 45.90417413 |
| **SHMT2** | 1.496051 | 5.483072 | 11.02332 | 5.53E-25 | 2.33E-23 | 45.90174763 |
| **TRA2B** | 1.53854 | 5.202482 | 11.02117 | 5.63E-25 | 2.37E-23 | 45.88340399 |
| **ANTXR2** | 1.063089 | 2.086807 | 11.02098 | 5.64E-25 | 2.37E-23 | 45.88182704 |
| **TMEM51** | 1.076005 | 2.613642 | 11.01568 | 5.91E-25 | 2.47E-23 | 45.83669863 |
| **NME6** | 0.975325 | 2.803613 | 11.01463 | 5.96E-25 | 2.49E-23 | 45.82776882 |
| **HDAC2** | 1.653958 | 4.795559 | 11.01456 | 5.96E-25 | 2.49E-23 | 45.8271657 |
| **ODC1** | 1.606226 | 4.92781 | 11.01416 | 5.98E-25 | 2.49E-23 | 45.82376074 |
| **ZNF85** | 0.903886 | 1.77799 | 11.01074 | 6.16E-25 | 2.56E-23 | 45.79460549 |
| **GCC1** | 0.939035 | 2.380377 | 11.0087 | 6.27E-25 | 2.60E-23 | 45.77726331 |
| **VAV2** | 1.385164 | 2.963993 | 11.00465 | 6.49E-25 | 2.69E-23 | 45.74286082 |
| **CCDC150** | 0.80689 | 1.299632 | 11.00316 | 6.58E-25 | 2.72E-23 | 45.73017845 |
| **ZNF677** | 1.303684 | 3.093665 | 10.99677 | 6.95E-25 | 2.87E-23 | 45.67575584 |
| **LAPTM4B** | 1.662217 | 5.097539 | 10.99525 | 7.04E-25 | 2.90E-23 | 45.66285029 |
| **LIG3** | 1.143886 | 2.83914 | 10.99512 | 7.05E-25 | 2.90E-23 | 45.66174122 |
| **FLT4** | 0.996422 | 1.781209 | 10.99012 | 7.36E-25 | 3.02E-23 | 45.61928158 |
| **NFATC3** | 0.956628 | 2.150581 | 10.98999 | 7.37E-25 | 3.02E-23 | 45.61816689 |
| **SH2B3** | 1.085369 | 2.135327 | 10.98738 | 7.53E-25 | 3.08E-23 | 45.59592976 |
| **PLEKHG2** | 1.360598 | 3.24337 | 10.9845 | 7.72E-25 | 3.15E-23 | 45.57143483 |
| **TOR4A** | 0.792359 | 1.510178 | 10.98431 | 7.74E-25 | 3.15E-23 | 45.56989309 |
| **MYC** | 1.625653 | 3.746631 | 10.98107 | 7.95E-25 | 3.24E-23 | 45.5423062 |
| **GART** | 1.249195 | 3.959865 | 10.98045 | 8.00E-25 | 3.25E-23 | 45.53707691 |
| **MAST2** | 1.115847 | 3.436591 | 10.97943 | 8.07E-25 | 3.27E-23 | 45.52837283 |
| **SOAT1** | 0.93732 | 2.095964 | 10.97812 | 8.16E-25 | 3.30E-23 | 45.51725949 |
| **TP53I3** | 1.355475 | 3.839509 | 10.97317 | 8.51E-25 | 3.44E-23 | 45.47520829 |
| **IL18BP** | 1.158589 | 2.858111 | 10.97081 | 8.69E-25 | 3.51E-23 | 45.45515163 |
| **C11orf95** | 1.149808 | 3.085803 | 10.96906 | 8.82E-25 | 3.55E-23 | 45.44026196 |
| **ZNF77** | 0.641143 | 1.39183 | 10.96695 | 8.98E-25 | 3.61E-23 | 45.42240447 |
| **UBN1** | 0.989751 | 2.729568 | 10.96362 | 9.24E-25 | 3.71E-23 | 45.39406319 |
| **TP53RK** | 0.811066 | 2.268232 | 10.96185 | 9.38E-25 | 3.76E-23 | 45.37905846 |
| **DNMT1** | 1.33761 | 4.814766 | 10.95029 | 1.04E-24 | 4.15E-23 | 45.28091674 |
| **C7orf49** | 1.14855 | 3.990804 | 10.94954 | 1.04E-24 | 4.17E-23 | 45.27462211 |
| **DDX19B** | 1.121592 | 3.494409 | 10.94834 | 1.05E-24 | 4.20E-23 | 45.26441699 |
| **NOTCH3** | 1.347263 | 3.02871 | 10.948 | 1.06E-24 | 4.20E-23 | 45.26148843 |
| **FAM222B** | 0.973404 | 2.482555 | 10.94793 | 1.06E-24 | 4.20E-23 | 45.26093834 |
| **RIOK1** | 1.040424 | 2.832157 | 10.94725 | 1.06E-24 | 4.22E-23 | 45.25518336 |
| **PIGW** | 0.929026 | 1.860649 | 10.94718 | 1.06E-24 | 4.22E-23 | 45.25453895 |
| **CCDC15** | 0.550976 | 0.820374 | 10.94647 | 1.07E-24 | 4.24E-23 | 45.24852018 |
| **USP1** | 1.22299 | 2.797991 | 10.94614 | 1.07E-24 | 4.24E-23 | 45.24575366 |
| **CCDC102B** | 0.870106 | 2.398957 | 10.94577 | 1.08E-24 | 4.25E-23 | 45.24261557 |
| **SLC43A3** | 1.235177 | 2.444865 | 10.94354 | 1.10E-24 | 4.32E-23 | 45.22370827 |
| **POGLUT1** | 0.972297 | 2.544804 | 10.94322 | 1.10E-24 | 4.33E-23 | 45.22096715 |
| **ZNF100** | 0.833315 | 1.544797 | 10.94282 | 1.10E-24 | 4.34E-23 | 45.21761235 |
| **KTI12** | 0.909237 | 2.371032 | 10.93653 | 1.17E-24 | 4.57E-23 | 45.16420775 |
| **DDX52** | 0.964145 | 2.458293 | 10.93374 | 1.19E-24 | 4.67E-23 | 45.14055731 |
| **TXNDC5** | 1.353712 | 3.549575 | 10.93344 | 1.20E-24 | 4.68E-23 | 45.13802278 |
| **BLOC1S5** | 0.805452 | 1.883992 | 10.92059 | 1.34E-24 | 5.20E-23 | 45.02916473 |
| **TRIB2** | 1.675387 | 4.161766 | 10.91846 | 1.36E-24 | 5.29E-23 | 45.01106269 |
| **NXT1** | 1.156335 | 3.553739 | 10.91594 | 1.39E-24 | 5.40E-23 | 44.98973439 |
| **ZNF883** | 0.926791 | 2.34896 | 10.91479 | 1.40E-24 | 5.44E-23 | 44.98001434 |
| **SMARCC1** | 1.185585 | 3.119602 | 10.90955 | 1.47E-24 | 5.68E-23 | 44.93567278 |
| **EFTUD2** | 1.23812 | 4.847862 | 10.90779 | 1.49E-24 | 5.76E-23 | 44.92074727 |
| **SCAF4** | 1.030492 | 2.763386 | 10.90665 | 1.51E-24 | 5.81E-23 | 44.91112146 |
| **TDG** | 1.080121 | 2.544065 | 10.90074 | 1.58E-24 | 6.10E-23 | 44.86109051 |
| **BACH1** | 0.834229 | 2.195461 | 10.90027 | 1.59E-24 | 6.11E-23 | 44.85710115 |
| **DCAF12** | 1.297129 | 3.175058 | 10.89992 | 1.60E-24 | 6.12E-23 | 44.85412268 |
| **ZFP36L2** | 1.591978 | 4.486195 | 10.89958 | 1.60E-24 | 6.13E-23 | 44.85121211 |
| **NOA1** | 1.068756 | 2.685947 | 10.89682 | 1.64E-24 | 6.27E-23 | 44.82788204 |
| **FBXO46** | 0.95601 | 2.564812 | 10.89587 | 1.65E-24 | 6.31E-23 | 44.81981482 |
| **TRAIP** | 0.878283 | 2.09016 | 10.89434 | 1.67E-24 | 6.38E-23 | 44.80691536 |
| **ERI1** | 0.892413 | 1.835841 | 10.89082 | 1.72E-24 | 6.57E-23 | 44.77709939 |
| **ZC3H10** | 0.890574 | 2.419618 | 10.88979 | 1.74E-24 | 6.61E-23 | 44.76839138 |
| **CALD1** | 1.549392 | 4.148699 | 10.88789 | 1.77E-24 | 6.71E-23 | 44.752312 |
| **MIR4435-1HG** | 1.531696 | 3.230822 | 10.88524 | 1.81E-24 | 6.85E-23 | 44.72996478 |
| **RNF2** | 0.959829 | 2.240375 | 10.88477 | 1.82E-24 | 6.87E-23 | 44.72598347 |
| **CSTF1** | 1.003562 | 3.001177 | 10.88344 | 1.84E-24 | 6.94E-23 | 44.71471234 |
| **XRN2** | 1.373519 | 3.05656 | 10.87814 | 1.92E-24 | 7.25E-23 | 44.66988836 |
| **PARD6G** | 0.739598 | 1.76886 | 10.87733 | 1.94E-24 | 7.29E-23 | 44.66303442 |
| **UTP15** | 0.892531 | 1.807271 | 10.87676 | 1.95E-24 | 7.31E-23 | 44.65826219 |
| **RBM12** | 1.253023 | 2.989493 | 10.87158 | 2.03E-24 | 7.63E-23 | 44.61442204 |
| **ZNF2** | 0.558185 | 1.270437 | 10.87085 | 2.05E-24 | 7.67E-23 | 44.60824688 |
| **GBE1** | 1.117507 | 2.346889 | 10.87043 | 2.05E-24 | 7.67E-23 | 44.60474399 |
| **ZNF616** | 0.75447 | 1.364727 | 10.87043 | 2.05E-24 | 7.67E-23 | 44.60468641 |
| **NUP160** | 1.159579 | 2.67414 | 10.86551 | 2.14E-24 | 7.99E-23 | 44.56317722 |
| **SHC1** | 1.426479 | 4.186314 | 10.85901 | 2.26E-24 | 8.43E-23 | 44.50823629 |
| **ZEB1-AS1** | 1.131446 | 3.496346 | 10.85592 | 2.32E-24 | 8.64E-23 | 44.4821735 |
| **MRPL42** | 1.427999 | 3.45097 | 10.85465 | 2.35E-24 | 8.72E-23 | 44.47146265 |
| **ING1** | 1.08387 | 3.195145 | 10.85427 | 2.36E-24 | 8.73E-23 | 44.46824098 |
| **YTHDF2** | 1.19857 | 3.788935 | 10.85422 | 2.36E-24 | 8.73E-23 | 44.46780682 |
| **ZNF398** | 0.747038 | 1.798922 | 10.85309 | 2.38E-24 | 8.80E-23 | 44.45829098 |
| **MPZL1** | 1.569331 | 4.485932 | 10.84799 | 2.49E-24 | 9.18E-23 | 44.41519855 |
| **ANKRD50** | 0.886954 | 1.556379 | 10.8466 | 2.52E-24 | 9.27E-23 | 44.40347611 |
| **IFNGR2** | 1.244949 | 4.05425 | 10.84438 | 2.57E-24 | 9.43E-23 | 44.38470541 |
| **E2F6** | 1.078919 | 2.804423 | 10.84423 | 2.57E-24 | 9.43E-23 | 44.38347909 |
| **PTCD1** | 0.852207 | 2.621582 | 10.84305 | 2.59E-24 | 9.51E-23 | 44.37347952 |
| **GGH** | 1.196833 | 3.476976 | 10.84245 | 2.61E-24 | 9.55E-23 | 44.368478 |
| **TBCCD1** | 0.970963 | 2.124867 | 10.83853 | 2.70E-24 | 9.86E-23 | 44.33538816 |
| **COMMD2** | 1.371629 | 3.083791 | 10.83838 | 2.70E-24 | 9.86E-23 | 44.33409051 |
| **CCDC138** | 0.596865 | 1.537739 | 10.83739 | 2.72E-24 | 9.92E-23 | 44.32574111 |
| **ZNF586** | 0.639441 | 1.404196 | 10.83695 | 2.73E-24 | 9.95E-23 | 44.32205243 |
| **RECQL** | 1.392984 | 2.395052 | 10.83632 | 2.75E-24 | 9.96E-23 | 44.31672678 |
| **PANX1** | 1.106215 | 2.202605 | 10.83625 | 2.75E-24 | 9.96E-23 | 44.3161285 |
| **HOXD3** | 1.057943 | 1.03026 | 10.83619 | 2.75E-24 | 9.96E-23 | 44.31564704 |
| **EMC1** | 1.184674 | 3.379485 | 10.83114 | 2.87E-24 | 1.04E-22 | 44.27307971 |
| **CYB5RL** | 0.713488 | 1.204671 | 10.83099 | 2.88E-24 | 1.04E-22 | 44.27178426 |
| **UBL7-AS1** | 0.556921 | 1.206454 | 10.83078 | 2.88E-24 | 1.04E-22 | 44.26997944 |
| **SLC30A7** | 0.713177 | 1.272806 | 10.83025 | 2.89E-24 | 1.04E-22 | 44.26550488 |
| **ZNF74** | 1.155991 | 3.40728 | 10.83016 | 2.90E-24 | 1.04E-22 | 44.26481749 |
| **ZSCAN16** | 0.818772 | 2.216114 | 10.82991 | 2.90E-24 | 1.04E-22 | 44.26263111 |
| **ZEB1** | 1.625401 | 4.5199 | 10.82976 | 2.91E-24 | 1.04E-22 | 44.26136905 |
| **TRIM16** | 1.027402 | 3.202445 | 10.82862 | 2.93E-24 | 1.05E-22 | 44.25183191 |
| **ATP11C** | 0.813448 | 1.683334 | 10.82255 | 3.09E-24 | 1.10E-22 | 44.20063506 |
| **HNRNPLL** | 1.255601 | 3.264819 | 10.82125 | 3.13E-24 | 1.12E-22 | 44.1896731 |
| **BRPF1** | 1.098364 | 2.606178 | 10.82079 | 3.14E-24 | 1.12E-22 | 44.18582013 |
| **HIC1** | 0.91064 | 1.839562 | 10.82002 | 3.16E-24 | 1.12E-22 | 44.17932756 |
| **RARA** | 1.288499 | 3.768552 | 10.81875 | 3.19E-24 | 1.13E-22 | 44.16861015 |
| **RBBP8** | 1.100623 | 3.199137 | 10.81486 | 3.30E-24 | 1.17E-22 | 44.13582661 |
| **HUS1** | 1.038856 | 2.401315 | 10.81485 | 3.30E-24 | 1.17E-22 | 44.13570147 |
| **TSR1** | 1.168168 | 2.996668 | 10.8124 | 3.37E-24 | 1.19E-22 | 44.11511179 |
| **ILDR2** | 1.091357 | 2.096811 | 10.81223 | 3.37E-24 | 1.19E-22 | 44.11365278 |
| **CHST11** | 1.451042 | 3.403051 | 10.80485 | 3.59E-24 | 1.27E-22 | 44.05147027 |
| **TUBB** | 1.745417 | 7.096527 | 10.80295 | 3.65E-24 | 1.29E-22 | 44.03548977 |
| **SOX13** | 1.325036 | 3.949197 | 10.79966 | 3.76E-24 | 1.32E-22 | 44.0077701 |
| **DPP9** | 1.121527 | 3.769294 | 10.79701 | 3.84E-24 | 1.35E-22 | 43.98547051 |
| **C14orf93** | 0.908363 | 2.738817 | 10.79581 | 3.88E-24 | 1.36E-22 | 43.97540255 |
| **PDIA5** | 0.992078 | 1.862626 | 10.79224 | 4.00E-24 | 1.40E-22 | 43.94527925 |
| **AAED1** | 0.792408 | 1.821847 | 10.79041 | 4.06E-24 | 1.42E-22 | 43.92993643 |
| **PHACTR4** | 0.959422 | 2.171287 | 10.79007 | 4.08E-24 | 1.42E-22 | 43.92707884 |
| **ZNF480** | 0.727699 | 1.719357 | 10.78739 | 4.17E-24 | 1.45E-22 | 43.90449276 |
| **ZNF75A** | 1.095578 | 3.05715 | 10.7872 | 4.18E-24 | 1.45E-22 | 43.9029256 |
| **SUZ12** | 1.053467 | 2.469245 | 10.78709 | 4.18E-24 | 1.45E-22 | 43.90196307 |
| **SLC39A1** | 1.36324 | 4.706108 | 10.78589 | 4.22E-24 | 1.46E-22 | 43.89184541 |
| **NADK** | 1.328481 | 4.531173 | 10.78583 | 4.23E-24 | 1.46E-22 | 43.89139554 |
| **PSKH1** | 1.120449 | 3.511125 | 10.7848 | 4.26E-24 | 1.48E-22 | 43.88274829 |
| **NRP1** | 1.270223 | 2.681342 | 10.78236 | 4.35E-24 | 1.50E-22 | 43.86222319 |
| **KLHL7** | 1.380772 | 3.986904 | 10.77952 | 4.46E-24 | 1.54E-22 | 43.83831739 |
| **CTDSP2** | 1.480512 | 4.477894 | 10.77912 | 4.47E-24 | 1.54E-22 | 43.83495185 |
| **HLX** | 0.901283 | 1.911344 | 10.77712 | 4.55E-24 | 1.56E-22 | 43.81808367 |
| **VEZF1** | 1.399032 | 3.898405 | 10.77698 | 4.56E-24 | 1.56E-22 | 43.81696084 |
| **RAP2B** | 0.89854 | 2.011144 | 10.77647 | 4.58E-24 | 1.57E-22 | 43.81261875 |
| **POLA1** | 0.813662 | 1.886051 | 10.77396 | 4.68E-24 | 1.60E-22 | 43.79156382 |
| **SLC37A3** | 1.14893 | 3.554842 | 10.77363 | 4.69E-24 | 1.60E-22 | 43.788768 |
| **BARD1** | 0.747094 | 1.245417 | 10.77275 | 4.72E-24 | 1.61E-22 | 43.78133432 |
| **PDS5A** | 1.09968 | 3.177651 | 10.77261 | 4.73E-24 | 1.61E-22 | 43.78022493 |
| **TES** | 0.735194 | 0.959903 | 10.76604 | 5.00E-24 | 1.70E-22 | 43.72497347 |
| **NOTCH1** | 1.368974 | 4.255952 | 10.76523 | 5.04E-24 | 1.71E-22 | 43.71814183 |
| **LRRC59** | 1.335731 | 4.255669 | 10.7651 | 5.04E-24 | 1.71E-22 | 43.71703564 |
| **UNK** | 1.297588 | 4.113912 | 10.76437 | 5.07E-24 | 1.72E-22 | 43.71095873 |
| **DHODH** | 0.876694 | 2.735503 | 10.76378 | 5.10E-24 | 1.72E-22 | 43.70597988 |
| **PLXNA3** | 1.377826 | 3.696474 | 10.76311 | 5.13E-24 | 1.73E-22 | 43.70030013 |
| **TTC39C** | 0.845658 | 2.447248 | 10.76191 | 5.18E-24 | 1.75E-22 | 43.69024635 |
| **ZNF436** | 1.186807 | 2.482687 | 10.76175 | 5.19E-24 | 1.75E-22 | 43.68889474 |
| **BMP1** | 1.176877 | 3.56467 | 10.76163 | 5.19E-24 | 1.75E-22 | 43.68788613 |
| **PRMT6** | 1.139302 | 2.541797 | 10.75877 | 5.32E-24 | 1.79E-22 | 43.66383285 |
| **TULP3** | 1.07607 | 2.709541 | 10.75758 | 5.37E-24 | 1.80E-22 | 43.65383601 |
| **CCDC18** | 0.755316 | 1.272429 | 10.7527 | 5.60E-24 | 1.88E-22 | 43.61284108 |
| **RPN2** | 1.531498 | 5.213965 | 10.74667 | 5.90E-24 | 1.97E-22 | 43.5622503 |
| **TFPI** | 1.254199 | 1.76125 | 10.74666 | 5.90E-24 | 1.97E-22 | 43.56213206 |
| **GAL3ST4** | 1.201161 | 3.43669 | 10.74599 | 5.93E-24 | 1.98E-22 | 43.55650474 |
| **ZNF227** | 1.056718 | 2.697617 | 10.74461 | 6.00E-24 | 2.00E-22 | 43.54495554 |
| **ADNP** | 1.219633 | 3.435422 | 10.74374 | 6.04E-24 | 2.00E-22 | 43.53767513 |
| **ABCC1** | 1.002145 | 2.180212 | 10.74373 | 6.05E-24 | 2.00E-22 | 43.53755145 |
| **BRIX1** | 1.102865 | 3.27186 | 10.74373 | 6.05E-24 | 2.00E-22 | 43.53753453 |
| **UTP11L** | 1.237956 | 3.902658 | 10.74301 | 6.08E-24 | 2.01E-22 | 43.53151296 |
| **RPRD1B** | 1.005412 | 2.635775 | 10.73987 | 6.25E-24 | 2.07E-22 | 43.50513382 |
| **MMP15** | 1.216394 | 3.400544 | 10.73911 | 6.29E-24 | 2.08E-22 | 43.49882105 |
| **SFT2D2** | 0.916075 | 2.146872 | 10.73876 | 6.31E-24 | 2.08E-22 | 43.49587211 |
| **RAB42** | 0.800181 | 0.89314 | 10.73629 | 6.44E-24 | 2.12E-22 | 43.47510888 |
| **KDM2B** | 1.166587 | 3.261295 | 10.73501 | 6.51E-24 | 2.14E-22 | 43.46434173 |
| **TRIB1** | 1.039131 | 2.43666 | 10.73442 | 6.54E-24 | 2.15E-22 | 43.45942274 |
| **ZNF700** | 0.833622 | 1.91551 | 10.73433 | 6.55E-24 | 2.15E-22 | 43.45869563 |
| **SYNCRIP** | 1.409913 | 4.037885 | 10.73313 | 6.61E-24 | 2.17E-22 | 43.44861978 |
| **CNTLN** | 0.677935 | 1.268217 | 10.73243 | 6.65E-24 | 2.18E-22 | 43.44274526 |
| **KIAA1524** | 0.790617 | 1.00647 | 10.73218 | 6.67E-24 | 2.18E-22 | 43.44061132 |
| **TRIOBP** | 1.195489 | 3.46988 | 10.73126 | 6.72E-24 | 2.19E-22 | 43.4329074 |
| **ACLY** | 1.446174 | 4.554897 | 10.72785 | 6.92E-24 | 2.25E-22 | 43.4043072 |
| **SZRD1** | 1.39151 | 4.695196 | 10.72645 | 7.00E-24 | 2.27E-22 | 43.39252803 |
| **MED1** | 1.02849 | 2.859953 | 10.72266 | 7.23E-24 | 2.35E-22 | 43.36081431 |
| **ZNF526** | 0.637574 | 1.523011 | 10.72245 | 7.24E-24 | 2.35E-22 | 43.3590245 |
| **RPP40** | 0.999011 | 2.560597 | 10.72189 | 7.28E-24 | 2.35E-22 | 43.354341 |
| **CDK6** | 1.100103 | 1.776212 | 10.7213 | 7.31E-24 | 2.36E-22 | 43.34939345 |
| **GNS** | 1.255061 | 3.513577 | 10.72069 | 7.35E-24 | 2.37E-22 | 43.3442939 |
| **CTPS2** | 0.978916 | 2.718435 | 10.72069 | 7.35E-24 | 2.37E-22 | 43.3442435 |
| **RP1-223E5.4** | 0.755563 | 1.813694 | 10.71627 | 7.63E-24 | 2.46E-22 | 43.3071724 |
| **DHX37** | 0.880059 | 2.918822 | 10.71424 | 7.76E-24 | 2.50E-22 | 43.2901855 |
| **FAM117A** | 0.864794 | 2.072054 | 10.71312 | 7.84E-24 | 2.52E-22 | 43.28077716 |
| **PTK7** | 1.327347 | 3.682218 | 10.71258 | 7.87E-24 | 2.52E-22 | 43.27626672 |
| **TM4SF1** | 1.369151 | 3.083323 | 10.70944 | 8.09E-24 | 2.59E-22 | 43.24999273 |
| **SOX12** | 1.491343 | 4.484309 | 10.70835 | 8.16E-24 | 2.61E-22 | 43.24081953 |
| **WDR43** | 1.060182 | 2.615275 | 10.70615 | 8.32E-24 | 2.66E-22 | 43.22235389 |
| **MICALL1** | 1.07464 | 3.051894 | 10.70324 | 8.52E-24 | 2.72E-22 | 43.19801208 |
| **UBFD1** | 1.499611 | 3.900596 | 10.70301 | 8.54E-24 | 2.72E-22 | 43.19605882 |
| **ZNF260** | 0.954181 | 2.112345 | 10.70248 | 8.58E-24 | 2.73E-22 | 43.19165483 |
| **KDM5B** | 1.124217 | 2.704702 | 10.70244 | 8.58E-24 | 2.73E-22 | 43.19133032 |
| **ZNF212** | 0.973965 | 2.954391 | 10.70097 | 8.69E-24 | 2.76E-22 | 43.17896103 |
| **ZNF16** | 0.754859 | 1.991332 | 10.7003 | 8.74E-24 | 2.77E-22 | 43.17338012 |
| **RBMS2** | 0.803734 | 1.713312 | 10.69403 | 9.22E-24 | 2.92E-22 | 43.12088802 |
| **TPM4** | 1.624712 | 5.432625 | 10.69377 | 9.24E-24 | 2.92E-22 | 43.11868907 |
| **RP1-152L7.5** | 0.886765 | 1.452241 | 10.69368 | 9.24E-24 | 2.92E-22 | 43.11798045 |
| **PRRC1** | 1.090312 | 2.885996 | 10.69284 | 9.31E-24 | 2.93E-22 | 43.11089856 |
| **ZNF140** | 1.089658 | 2.949799 | 10.69219 | 9.36E-24 | 2.95E-22 | 43.1054756 |
| **POLE3** | 1.311737 | 4.035981 | 10.69002 | 9.53E-24 | 3.00E-22 | 43.08730975 |
| **FGD1** | 1.02499 | 2.80112 | 10.6899 | 9.54E-24 | 3.00E-22 | 43.0862847 |
| **ZBED1** | 1.018312 | 2.976473 | 10.68815 | 9.69E-24 | 3.04E-22 | 43.07167259 |
| **CBFB** | 1.329522 | 3.416421 | 10.67982 | 1.04E-23 | 3.25E-22 | 43.00194866 |
| **SLC35A4** | 1.295862 | 4.373987 | 10.67917 | 1.05E-23 | 3.27E-22 | 42.99648101 |
| **NUP107** | 1.268129 | 2.917733 | 10.67708 | 1.06E-23 | 3.32E-22 | 42.97905779 |
| **CTC-260E6.6** | 0.597674 | 0.767383 | 10.67555 | 1.08E-23 | 3.36E-22 | 42.96624997 |
| **GTF3C4** | 0.915184 | 2.016549 | 10.67512 | 1.08E-23 | 3.37E-22 | 42.96264154 |
| **DAXX** | 1.226717 | 4.350945 | 10.67501 | 1.08E-23 | 3.37E-22 | 42.96172967 |
| **MOSPD1** | 0.805155 | 2.10783 | 10.66818 | 1.15E-23 | 3.56E-22 | 42.90457528 |
| **ZIK1** | 0.982471 | 1.624927 | 10.6681 | 1.15E-23 | 3.56E-22 | 42.90394762 |
| **SCFD2** | 1.127469 | 2.553906 | 10.66551 | 1.17E-23 | 3.63E-22 | 42.88224381 |
| **NHEJ1** | 0.777042 | 1.777594 | 10.66503 | 1.18E-23 | 3.64E-22 | 42.87824874 |
| **FNDC3B** | 1.014415 | 2.047232 | 10.66279 | 1.20E-23 | 3.71E-22 | 42.859541 |
| **FDXACB1** | 0.603721 | 1.375638 | 10.6617 | 1.21E-23 | 3.74E-22 | 42.85038792 |
| **MOB3A** | 1.164823 | 3.977221 | 10.66156 | 1.21E-23 | 3.74E-22 | 42.84926199 |
| **C8orf76** | 1.04439 | 3.248998 | 10.66061 | 1.22E-23 | 3.76E-22 | 42.84133429 |
| **ABCB7** | 0.958551 | 2.726289 | 10.66043 | 1.22E-23 | 3.76E-22 | 42.83982538 |
| **SPSB1** | 1.085108 | 2.908318 | 10.655 | 1.28E-23 | 3.94E-22 | 42.79438794 |
| **FANCF** | 0.79995 | 1.73902 | 10.6546 | 1.29E-23 | 3.94E-22 | 42.79111397 |
| **PRPF40A** | 1.296718 | 3.242371 | 10.6545 | 1.29E-23 | 3.94E-22 | 42.79023516 |
| **PSMD11** | 1.320115 | 4.483077 | 10.65363 | 1.30E-23 | 3.97E-22 | 42.78298474 |
| **RPAP3** | 0.938432 | 2.664936 | 10.653 | 1.30E-23 | 3.98E-22 | 42.77771575 |
| **TRAF4** | 1.539256 | 5.164687 | 10.6528 | 1.31E-23 | 3.99E-22 | 42.77606404 |
| **COX10-AS1** | 0.945755 | 2.270761 | 10.65051 | 1.33E-23 | 4.06E-22 | 42.75686794 |
| **NKX2-2** | 1.3514 | 3.595288 | 10.64895 | 1.35E-23 | 4.10E-22 | 42.74384541 |
| **BCDIN3D** | 0.906575 | 2.316737 | 10.64891 | 1.35E-23 | 4.10E-22 | 42.74356683 |
| **ZNF45** | 0.916436 | 1.686304 | 10.64876 | 1.35E-23 | 4.10E-22 | 42.74227695 |
| **LRRC42** | 1.051534 | 3.114586 | 10.64657 | 1.38E-23 | 4.17E-22 | 42.72398032 |
| **QTRTD1** | 1.020581 | 1.934711 | 10.64367 | 1.41E-23 | 4.27E-22 | 42.69980062 |
| **VASH1** | 1.249867 | 4.442537 | 10.64333 | 1.42E-23 | 4.27E-22 | 42.69695696 |
| **WDR3** | 1.17607 | 1.817318 | 10.64322 | 1.42E-23 | 4.27E-22 | 42.69606305 |
| **ZC3H4** | 1.125374 | 3.227704 | 10.64318 | 1.42E-23 | 4.27E-22 | 42.69568114 |
| **RNF138** | 1.294651 | 2.870839 | 10.64313 | 1.42E-23 | 4.27E-22 | 42.69531361 |
| **MFNG** | 1.147459 | 3.057722 | 10.64087 | 1.45E-23 | 4.35E-22 | 42.67636287 |
| **EXT1** | 1.009956 | 2.143532 | 10.63875 | 1.47E-23 | 4.42E-22 | 42.65868945 |
| **ZDHHC15** | 0.932418 | 1.606513 | 10.63853 | 1.47E-23 | 4.42E-22 | 42.65688184 |
| **RP11-110G21.1** | 0.81478 | 2.337937 | 10.63452 | 1.52E-23 | 4.57E-22 | 42.62342228 |
| **WDR77** | 1.067344 | 3.457302 | 10.63312 | 1.54E-23 | 4.62E-22 | 42.61169372 |
| **GPN1** | 1.1928 | 3.728501 | 10.63286 | 1.55E-23 | 4.62E-22 | 42.60950119 |
| **CBX3** | 1.722124 | 5.748057 | 10.63249 | 1.55E-23 | 4.63E-22 | 42.60644272 |
| **PDIK1L** | 0.783296 | 1.767373 | 10.62984 | 1.59E-23 | 4.73E-22 | 42.5843558 |
| **MLX** | 1.163836 | 3.90192 | 10.62886 | 1.60E-23 | 4.76E-22 | 42.57616674 |
| **DCAF16** | 1.203401 | 3.236087 | 10.62823 | 1.61E-23 | 4.78E-22 | 42.57090145 |
| **C19orf55** | 1.104667 | 2.263826 | 10.62745 | 1.62E-23 | 4.81E-22 | 42.56437313 |
| **POLH** | 0.880529 | 1.572139 | 10.62639 | 1.63E-23 | 4.85E-22 | 42.55556788 |
| **TGIF1** | 1.343807 | 2.898053 | 10.62609 | 1.64E-23 | 4.85E-22 | 42.55305076 |
| **WARS2** | 0.691922 | 1.708975 | 10.62343 | 1.67E-23 | 4.96E-22 | 42.53087518 |
| **TMEM39B** | 0.992892 | 3.254899 | 10.61622 | 1.78E-23 | 5.26E-22 | 42.47072699 |
| **METTL21B** | 0.934719 | 1.751111 | 10.61616 | 1.78E-23 | 5.26E-22 | 42.47021281 |
| **HAS2** | 0.906649 | 1.012948 | 10.61512 | 1.80E-23 | 5.30E-22 | 42.46158155 |
| **C17orf103** | 0.737563 | 1.813743 | 10.61452 | 1.80E-23 | 5.32E-22 | 42.45658473 |
| **ZDHHC5** | 0.994867 | 3.320969 | 10.61101 | 1.86E-23 | 5.47E-22 | 42.42729549 |
| **HIP1** | 1.269877 | 3.48753 | 10.60757 | 1.91E-23 | 5.62E-22 | 42.39860702 |
| **DCP1B** | 0.887318 | 2.344642 | 10.6075 | 1.92E-23 | 5.62E-22 | 42.39806417 |
| **CHSY1** | 1.058903 | 2.354712 | 10.60477 | 1.96E-23 | 5.74E-22 | 42.37532314 |
| **POM121C** | 1.188253 | 3.853328 | 10.60348 | 1.98E-23 | 5.79E-22 | 42.36450706 |
| **C11orf84** | 1.17066 | 3.587001 | 10.60333 | 1.98E-23 | 5.79E-22 | 42.36328531 |
| **ZNF266** | 1.189578 | 3.455126 | 10.60106 | 2.02E-23 | 5.90E-22 | 42.34439003 |
| **APOBEC3B** | 0.599654 | 0.64457 | 10.60081 | 2.03E-23 | 5.90E-22 | 42.3423053 |
| **KIAA0586** | 0.797408 | 2.082632 | 10.60037 | 2.03E-23 | 5.91E-22 | 42.33862294 |
| **TMA16** | 1.0561 | 2.616208 | 10.59935 | 2.05E-23 | 5.95E-22 | 42.33014583 |
| **PDGFRA** | 1.7525 | 4.153528 | 10.59421 | 2.14E-23 | 6.21E-22 | 42.28736004 |
| **EIF4E2** | 1.204178 | 4.268651 | 10.59295 | 2.17E-23 | 6.27E-22 | 42.27683834 |
| **RP2** | 1.080786 | 1.950603 | 10.59204 | 2.18E-23 | 6.31E-22 | 42.26928164 |
| **N4BP2** | 0.707012 | 1.059084 | 10.58996 | 2.22E-23 | 6.41E-22 | 42.25189647 |
| **ZC3HC1** | 0.998838 | 3.405539 | 10.58982 | 2.22E-23 | 6.41E-22 | 42.25075744 |
| **ZNF263** | 1.149547 | 4.002329 | 10.58956 | 2.23E-23 | 6.41E-22 | 42.24857865 |
| **PNO1** | 0.929884 | 2.643662 | 10.58944 | 2.23E-23 | 6.41E-22 | 42.24760333 |
| **ELMOD2** | 0.865849 | 2.321222 | 10.58938 | 2.23E-23 | 6.41E-22 | 42.24712809 |
| **TAGLN2** | 1.720602 | 5.263175 | 10.58536 | 2.31E-23 | 6.63E-22 | 42.21366197 |
| **ST8SIA4** | 0.685371 | 1.065736 | 10.58486 | 2.32E-23 | 6.65E-22 | 42.20946251 |
| **CASP6** | 0.961255 | 2.159794 | 10.5847 | 2.32E-23 | 6.65E-22 | 42.20809855 |
| **MSI1** | 1.345486 | 3.587257 | 10.58286 | 2.36E-23 | 6.74E-22 | 42.19284806 |
| **LBH** | 1.480319 | 2.983827 | 10.58121 | 2.39E-23 | 6.83E-22 | 42.17905336 |
| **PCDH12** | 0.742363 | 1.637225 | 10.58046 | 2.41E-23 | 6.87E-22 | 42.17286994 |
| **KPNB1** | 1.66326 | 5.045316 | 10.58028 | 2.41E-23 | 6.87E-22 | 42.17131574 |
| **ZCCHC7** | 0.827522 | 2.586029 | 10.5788 | 2.44E-23 | 6.95E-22 | 42.15904546 |
| **TMEM19** | 1.020473 | 2.792997 | 10.57771 | 2.46E-23 | 7.00E-22 | 42.14993048 |
| **ACTR3** | 1.558592 | 4.077249 | 10.57705 | 2.48E-23 | 7.03E-22 | 42.14449001 |
| **ITGA1** | 0.792164 | 1.293117 | 10.57704 | 2.48E-23 | 7.03E-22 | 42.14435644 |
| **FADD** | 1.147267 | 3.14515 | 10.57538 | 2.51E-23 | 7.12E-22 | 42.13055522 |
| **ABL1** | 1.430682 | 3.957559 | 10.57435 | 2.53E-23 | 7.17E-22 | 42.12200605 |
| **N4BP1** | 0.853468 | 2.594387 | 10.57309 | 2.56E-23 | 7.23E-22 | 42.11150243 |
| **ANGPT2** | 1.077842 | 1.353007 | 10.57303 | 2.56E-23 | 7.23E-22 | 42.11104303 |
| **DHX40** | 1.354797 | 3.416954 | 10.57292 | 2.56E-23 | 7.23E-22 | 42.11012256 |
| **FAM111A** | 1.143744 | 2.69733 | 10.57227 | 2.58E-23 | 7.26E-22 | 42.10474115 |
| **GALNT10** | 1.199321 | 3.626457 | 10.57003 | 2.63E-23 | 7.39E-22 | 42.08611705 |
| **MKS1** | 1.071947 | 3.549276 | 10.56674 | 2.70E-23 | 7.59E-22 | 42.05875353 |
| **NOM1** | 0.885312 | 2.485675 | 10.56205 | 2.81E-23 | 7.88E-22 | 42.01968277 |
| **ZNF528** | 1.007239 | 2.71339 | 10.562 | 2.81E-23 | 7.88E-22 | 42.01931307 |
| **TMEM209** | 1.088475 | 2.581113 | 10.5618 | 2.81E-23 | 7.88E-22 | 42.01766291 |
| **SIAH2** | 1.050767 | 3.128896 | 10.55796 | 2.91E-23 | 8.13E-22 | 41.98569925 |
| **PGD** | 1.4735 | 5.312789 | 10.55707 | 2.93E-23 | 8.18E-22 | 41.97829717 |
| **MCM7** | 1.609748 | 5.915164 | 10.55701 | 2.93E-23 | 8.18E-22 | 41.97784158 |
| **PUS7** | 0.876102 | 1.671483 | 10.55264 | 3.04E-23 | 8.48E-22 | 41.94147853 |
| **NUDT19** | 0.649241 | 1.747025 | 10.55236 | 3.05E-23 | 8.48E-22 | 41.93919332 |
| **TBL2** | 1.136969 | 3.773244 | 10.55234 | 3.05E-23 | 8.48E-22 | 41.93901759 |
| **CTC-260E6.10** | 0.543127 | 0.542886 | 10.55066 | 3.09E-23 | 8.59E-22 | 41.92509807 |
| **PGBD2** | 0.596786 | 1.231283 | 10.55044 | 3.10E-23 | 8.59E-22 | 41.9231967 |
| **TAF5** | 0.576883 | 1.305618 | 10.55023 | 3.10E-23 | 8.59E-22 | 41.92148352 |
| **NSUN4** | 0.907154 | 2.403027 | 10.55014 | 3.10E-23 | 8.59E-22 | 41.92077672 |
| **ENAH** | 1.563071 | 4.015169 | 10.55012 | 3.10E-23 | 8.59E-22 | 41.92057759 |
| **YBX3** | 1.469993 | 4.195939 | 10.54927 | 3.13E-23 | 8.64E-22 | 41.91354948 |
| **ZNF239** | 0.87752 | 1.940699 | 10.54635 | 3.20E-23 | 8.84E-22 | 41.88927565 |
| **TRMT12** | 0.824832 | 2.350783 | 10.54587 | 3.22E-23 | 8.87E-22 | 41.88526635 |
| **SMC5** | 1.045908 | 2.287297 | 10.5456 | 3.23E-23 | 8.88E-22 | 41.88302941 |
| **ZNF174** | 0.909326 | 2.69639 | 10.54529 | 3.23E-23 | 8.89E-22 | 41.88047208 |
| **PRPF38A** | 1.195592 | 3.679245 | 10.54524 | 3.23E-23 | 8.89E-22 | 41.88003852 |
| **SPATA5L1** | 0.938088 | 2.777819 | 10.54349 | 3.28E-23 | 9.01E-22 | 41.86551353 |
| **BMS1** | 0.738329 | 2.275419 | 10.54268 | 3.31E-23 | 9.06E-22 | 41.85873248 |
| **SRSF10** | 1.482766 | 4.194995 | 10.53899 | 3.41E-23 | 9.33E-22 | 41.82812095 |
| **C10orf2** | 0.959165 | 2.566254 | 10.53834 | 3.43E-23 | 9.37E-22 | 41.82273296 |
| **CTTNBP2NL** | 0.952582 | 1.897736 | 10.53807 | 3.44E-23 | 9.38E-22 | 41.82052806 |
| **RP1-74M1.3** | 0.634694 | 0.81975 | 10.53655 | 3.48E-23 | 9.49E-22 | 41.80791181 |
| **ZNF527** | 0.660193 | 1.550551 | 10.53505 | 3.52E-23 | 9.60E-22 | 41.79542031 |
| **MTA2** | 1.287585 | 4.731067 | 10.53465 | 3.54E-23 | 9.62E-22 | 41.7920741 |
| **UMPS** | 0.933714 | 3.181929 | 10.52902 | 3.71E-23 | 1.01E-21 | 41.74538442 |
| **NOC3L** | 0.860576 | 2.083573 | 10.52889 | 3.71E-23 | 1.01E-21 | 41.74432336 |
| **ZNF259** | 1.036413 | 3.500409 | 10.52478 | 3.84E-23 | 1.04E-21 | 41.7101539 |
| **C3orf38** | 0.953857 | 2.439461 | 10.52311 | 3.90E-23 | 1.06E-21 | 41.69635876 |
| **PPP1R18** | 1.361978 | 4.568214 | 10.52259 | 3.91E-23 | 1.06E-21 | 41.69203212 |
| **MIS18BP1** | 0.934884 | 1.63006 | 10.52168 | 3.94E-23 | 1.07E-21 | 41.68447249 |
| **TEFM** | 0.978844 | 2.513869 | 10.52152 | 3.95E-23 | 1.07E-21 | 41.6831339 |
| **KDELR2** | 1.365914 | 4.592908 | 10.52027 | 3.99E-23 | 1.08E-21 | 41.67277699 |
| **SETD1B** | 0.894066 | 2.208485 | 10.51905 | 4.03E-23 | 1.09E-21 | 41.66265278 |
| **FAM35A** | 0.919934 | 2.763978 | 10.51155 | 4.29E-23 | 1.16E-21 | 41.60046722 |
| **BCOR** | 0.962301 | 2.515476 | 10.50966 | 4.36E-23 | 1.17E-21 | 41.58478485 |
| **GRWD1** | 1.131255 | 3.134956 | 10.50776 | 4.43E-23 | 1.19E-21 | 41.5690296 |
| **STX2** | 1.054152 | 2.879962 | 10.50721 | 4.45E-23 | 1.19E-21 | 41.56445356 |
| **TAF1A** | 0.88744 | 1.633874 | 10.50524 | 4.53E-23 | 1.21E-21 | 41.54816908 |
| **NEURL1B** | 1.006261 | 2.147159 | 10.50032 | 4.72E-23 | 1.26E-21 | 41.50740079 |
| **ENPEP** | 0.667076 | 0.675589 | 10.49984 | 4.74E-23 | 1.27E-21 | 41.50338678 |
| **PRKD1** | 1.056385 | 2.631816 | 10.49774 | 4.82E-23 | 1.29E-21 | 41.48604484 |
| **CLIC4** | 1.728323 | 4.761395 | 10.49394 | 4.98E-23 | 1.33E-21 | 41.45457194 |
| **TBC1D31** | 0.559964 | 1.510919 | 10.49013 | 5.14E-23 | 1.37E-21 | 41.42300207 |
| **ZNF880** | 0.907153 | 2.789562 | 10.48997 | 5.14E-23 | 1.37E-21 | 41.42164384 |
| **GCN1L1** | 1.106356 | 3.83063 | 10.48989 | 5.15E-23 | 1.37E-21 | 41.42097911 |
| **RAD1** | 1.017746 | 2.693826 | 10.48938 | 5.17E-23 | 1.37E-21 | 41.41676913 |
| **MAPK1IP1L** | 1.222229 | 3.293917 | 10.48856 | 5.21E-23 | 1.38E-21 | 41.41002049 |
| **WSCD1** | 1.383616 | 4.865916 | 10.48641 | 5.30E-23 | 1.40E-21 | 41.39214781 |
| **SLC7A6** | 0.969216 | 2.424497 | 10.48473 | 5.38E-23 | 1.42E-21 | 41.37830743 |
| **GANAB** | 1.491192 | 5.335999 | 10.4835 | 5.43E-23 | 1.44E-21 | 41.36806926 |
| **FBXW2** | 1.193209 | 2.972238 | 10.48207 | 5.50E-23 | 1.45E-21 | 41.35629491 |
| **TMX1** | 1.352704 | 3.479613 | 10.48145 | 5.53E-23 | 1.46E-21 | 41.35113685 |
| **ADAT1** | 1.09377 | 3.515461 | 10.48111 | 5.54E-23 | 1.46E-21 | 41.34832121 |
| **EYA3** | 0.744002 | 1.86116 | 10.4802 | 5.58E-23 | 1.47E-21 | 41.34074616 |
| **MFAP2** | 1.3242 | 1.626434 | 10.47912 | 5.63E-23 | 1.48E-21 | 41.33185761 |
| **TTF2** | 0.776894 | 1.368884 | 10.4777 | 5.70E-23 | 1.50E-21 | 41.32009175 |
| **GSPT1** | 1.338657 | 3.778289 | 10.47737 | 5.72E-23 | 1.50E-21 | 41.31735374 |
| **ARF6** | 1.414476 | 3.983138 | 10.47724 | 5.72E-23 | 1.50E-21 | 41.31631187 |
| **RNF4** | 1.37068 | 4.060291 | 10.4768 | 5.74E-23 | 1.50E-21 | 41.31267164 |
| **TRIO** | 1.701491 | 4.919154 | 10.47604 | 5.78E-23 | 1.51E-21 | 41.30636867 |
| **RGS16** | 1.123402 | 1.813597 | 10.47411 | 5.88E-23 | 1.53E-21 | 41.29037076 |
| **PODXL** | 1.277767 | 3.404059 | 10.47282 | 5.94E-23 | 1.55E-21 | 41.27974818 |
| **MTSS1** | 1.425449 | 4.113884 | 10.47275 | 5.94E-23 | 1.55E-21 | 41.27917148 |
| **DLEU1** | 0.990741 | 2.365869 | 10.4698 | 6.09E-23 | 1.59E-21 | 41.25471907 |
| **DPP3** | 1.153936 | 3.959191 | 10.46856 | 6.16E-23 | 1.60E-21 | 41.24448006 |
| **DNAJC9** | 1.131974 | 3.084771 | 10.46666 | 6.25E-23 | 1.62E-21 | 41.22877522 |
| **CRISPLD1** | 1.48067 | 2.652045 | 10.46581 | 6.30E-23 | 1.63E-21 | 41.2217406 |
| **C8orf58** | 0.842127 | 2.750266 | 10.46442 | 6.37E-23 | 1.65E-21 | 41.2102006 |
| **TRIM26** | 1.216071 | 3.961181 | 10.4643 | 6.38E-23 | 1.65E-21 | 41.20927863 |
| **TEAD2** | 1.080622 | 2.029289 | 10.46292 | 6.45E-23 | 1.67E-21 | 41.19783651 |
| **HMGA1** | 1.417345 | 4.93328 | 10.46204 | 6.50E-23 | 1.68E-21 | 41.19056849 |
| **CPSF6** | 1.277467 | 3.593496 | 10.46176 | 6.52E-23 | 1.68E-21 | 41.18826863 |
| **ZNF131** | 1.128539 | 3.529918 | 10.45848 | 6.70E-23 | 1.73E-21 | 41.16113096 |
| **LYAR** | 1.042241 | 3.209188 | 10.45765 | 6.74E-23 | 1.74E-21 | 41.15426081 |
| **ZNF558** | 1.246572 | 2.703151 | 10.45764 | 6.74E-23 | 1.74E-21 | 41.1541575 |
| **TRAM2** | 0.819786 | 1.463376 | 10.45574 | 6.85E-23 | 1.76E-21 | 41.1385187 |
| **AAR2** | 1.067506 | 3.550498 | 10.45436 | 6.93E-23 | 1.78E-21 | 41.12710488 |
| **FKBP10** | 1.668105 | 5.357169 | 10.45192 | 7.08E-23 | 1.81E-21 | 41.10689136 |
| **SH3D19** | 1.190049 | 3.087275 | 10.44939 | 7.23E-23 | 1.85E-21 | 41.08600702 |
| **SERBP1** | 1.628619 | 5.223998 | 10.44939 | 7.23E-23 | 1.85E-21 | 41.08598023 |
| **CNTRL** | 0.894361 | 1.878547 | 10.44905 | 7.25E-23 | 1.85E-21 | 41.0832297 |
| **ZNF146** | 1.401481 | 3.7475 | 10.4481 | 7.30E-23 | 1.87E-21 | 41.07532287 |
| **VRK1** | 0.847497 | 2.517365 | 10.44557 | 7.46E-23 | 1.90E-21 | 41.05442457 |
| **AC012307.3** | 0.580947 | 1.82735 | 10.44453 | 7.53E-23 | 1.92E-21 | 41.04584997 |
| **UBA6-AS1** | 0.856375 | 2.554542 | 10.44405 | 7.56E-23 | 1.92E-21 | 41.04188745 |
| **HLCS** | 0.888167 | 1.865074 | 10.44139 | 7.73E-23 | 1.96E-21 | 41.01996908 |
| **SLC31A1** | 1.074298 | 2.626622 | 10.43878 | 7.90E-23 | 2.01E-21 | 40.99838366 |
| **MTBP** | 0.679718 | 1.102655 | 10.4384 | 7.92E-23 | 2.01E-21 | 40.99521743 |
| **ZMYM1** | 0.745859 | 1.228139 | 10.43754 | 7.98E-23 | 2.02E-21 | 40.98812279 |
| **PARS2** | 0.64158 | 1.546268 | 10.43753 | 7.98E-23 | 2.02E-21 | 40.98803502 |
| **DDX60L** | 0.901815 | 1.904942 | 10.43719 | 8.00E-23 | 2.02E-21 | 40.98522717 |
| **PELI1** | 1.316773 | 3.145681 | 10.43633 | 8.06E-23 | 2.04E-21 | 40.97817586 |
| **ZBTB5** | 0.908641 | 2.22948 | 10.435 | 8.15E-23 | 2.06E-21 | 40.96717221 |
| **RAB8A** | 1.233684 | 3.121034 | 10.4329 | 8.29E-23 | 2.09E-21 | 40.94987053 |
| **ERP44** | 0.895551 | 2.502491 | 10.43283 | 8.30E-23 | 2.09E-21 | 40.94922525 |
| **CD248** | 1.298316 | 1.877178 | 10.43128 | 8.41E-23 | 2.12E-21 | 40.93648616 |
| **ZNF623** | 0.978637 | 2.171967 | 10.43102 | 8.43E-23 | 2.12E-21 | 40.93433046 |
| **MYNN** | 0.918273 | 2.14909 | 10.42968 | 8.52E-23 | 2.14E-21 | 40.92324328 |
| **PHTF2** | 0.890376 | 2.383181 | 10.42868 | 8.59E-23 | 2.16E-21 | 40.91498949 |
| **SETD1A** | 0.90301 | 2.939859 | 10.42755 | 8.67E-23 | 2.17E-21 | 40.9056753 |
| **RFX5** | 1.379107 | 4.079887 | 10.42691 | 8.72E-23 | 2.18E-21 | 40.90036908 |
| **RBM15** | 0.634308 | 1.326339 | 10.42688 | 8.72E-23 | 2.18E-21 | 40.90012776 |
| **ZNF202** | 0.90692 | 2.229925 | 10.42638 | 8.76E-23 | 2.19E-21 | 40.89605362 |
| **ZNF114** | 0.639688 | 1.266449 | 10.42214 | 9.07E-23 | 2.26E-21 | 40.86104632 |
| **POU3F2** | 1.330779 | 3.100377 | 10.42208 | 9.08E-23 | 2.26E-21 | 40.86058506 |
| **PLSCR1** | 1.390225 | 3.167278 | 10.42169 | 9.11E-23 | 2.27E-21 | 40.85731667 |
| **RFC2** | 1.107724 | 3.863029 | 10.42033 | 9.21E-23 | 2.29E-21 | 40.8461033 |
| **ZMYM4** | 1.038447 | 2.822856 | 10.41839 | 9.36E-23 | 2.32E-21 | 40.83012764 |
| **ZNF267** | 0.8153 | 1.738926 | 10.41838 | 9.36E-23 | 2.32E-21 | 40.83003916 |
| **MED28** | 1.102294 | 3.083134 | 10.41835 | 9.37E-23 | 2.32E-21 | 40.82975723 |
| **AP3M1** | 1.253644 | 2.978887 | 10.41681 | 9.49E-23 | 2.35E-21 | 40.81710372 |
| **ELTD1** | 0.927599 | 1.767054 | 10.41653 | 9.51E-23 | 2.35E-21 | 40.81473265 |
| **NTN1** | 1.495804 | 3.32409 | 10.41579 | 9.57E-23 | 2.37E-21 | 40.80869967 |
| **CRY1** | 1.31026 | 3.031345 | 10.41436 | 9.68E-23 | 2.39E-21 | 40.79691155 |
| **CARD8** | 1.162923 | 3.236169 | 10.41211 | 9.87E-23 | 2.43E-21 | 40.77830354 |
| **ITPRIPL2** | 1.004002 | 1.577986 | 10.41065 | 9.99E-23 | 2.46E-21 | 40.76625147 |
| **FOXK2** | 1.035358 | 3.729107 | 10.40808 | 1.02E-22 | 2.51E-21 | 40.74513813 |
| **SRSF3** | 1.625897 | 5.329854 | 10.40738 | 1.03E-22 | 2.52E-21 | 40.73935895 |
| **DLL4** | 0.913298 | 1.640367 | 10.40676 | 1.03E-22 | 2.53E-21 | 40.73420885 |
| **TEX261** | 1.315015 | 4.076036 | 10.40434 | 1.05E-22 | 2.58E-21 | 40.71423676 |
| **DPAGT1** | 1.297983 | 3.771885 | 10.40425 | 1.05E-22 | 2.58E-21 | 40.71355759 |
| **LPCAT3** | 1.086089 | 3.606486 | 10.40377 | 1.06E-22 | 2.59E-21 | 40.70957971 |
| **NSMCE2** | 0.98468 | 3.318426 | 10.40323 | 1.06E-22 | 2.60E-21 | 40.70510274 |
| **SAMD9** | 0.714864 | 0.979985 | 10.40236 | 1.07E-22 | 2.61E-21 | 40.69795123 |
| **STAT3** | 1.450658 | 4.339806 | 10.40235 | 1.07E-22 | 2.61E-21 | 40.69791556 |
| **C2CD3** | 1.014488 | 2.856056 | 10.39757 | 1.11E-22 | 2.71E-21 | 40.65852506 |
| **IFNAR1** | 1.280205 | 3.416398 | 10.39497 | 1.14E-22 | 2.77E-21 | 40.6370938 |
| **ZNF384** | 1.393237 | 4.153105 | 10.39461 | 1.14E-22 | 2.78E-21 | 40.6340924 |
| **TRIM14** | 1.103606 | 2.482621 | 10.39398 | 1.15E-22 | 2.79E-21 | 40.62893203 |
| **PAK2** | 1.218191 | 3.535112 | 10.38981 | 1.19E-22 | 2.88E-21 | 40.59459129 |
| **SH3GLB1** | 1.365053 | 3.49539 | 10.38766 | 1.21E-22 | 2.93E-21 | 40.57689669 |
| **SMARCAL1** | 0.821921 | 2.777116 | 10.38727 | 1.21E-22 | 2.94E-21 | 40.57367965 |
| **VMP1** | 1.39559 | 4.435831 | 10.3852 | 1.23E-22 | 2.99E-21 | 40.55663413 |
| **MKI67IP** | 1.293556 | 4.092256 | 10.38466 | 1.24E-22 | 3.00E-21 | 40.55219105 |
| **TRMT5** | 0.936601 | 2.246433 | 10.38459 | 1.24E-22 | 3.00E-21 | 40.55160156 |
| **HAT1** | 1.281549 | 3.789822 | 10.3837 | 1.25E-22 | 3.02E-21 | 40.54429329 |
| **TMEM106C** | 1.407472 | 4.216256 | 10.38322 | 1.26E-22 | 3.03E-21 | 40.5403189 |
| **ELK3** | 0.9792 | 2.010936 | 10.38249 | 1.26E-22 | 3.04E-21 | 40.53434006 |
| **FBXW8** | 1.057389 | 1.889847 | 10.38174 | 1.27E-22 | 3.06E-21 | 40.52816845 |
| **NUP35** | 0.952904 | 2.750792 | 10.38128 | 1.28E-22 | 3.07E-21 | 40.52437644 |
| **ESCO1** | 0.800628 | 1.889359 | 10.37982 | 1.29E-22 | 3.10E-21 | 40.51235876 |
| **IFI30** | 1.726689 | 4.454588 | 10.37793 | 1.31E-22 | 3.15E-21 | 40.49675155 |
| **USB1** | 1.28522 | 4.164739 | 10.37674 | 1.33E-22 | 3.17E-21 | 40.48701546 |
| **TNFAIP8** | 0.719137 | 0.99817 | 10.37665 | 1.33E-22 | 3.17E-21 | 40.48624612 |
| **ZBTB42** | 0.672554 | 0.964968 | 10.37634 | 1.33E-22 | 3.18E-21 | 40.48369915 |
| **USP39** | 1.339358 | 4.349911 | 10.37568 | 1.34E-22 | 3.19E-21 | 40.47828492 |
| **CHFR** | 1.088433 | 3.679201 | 10.37343 | 1.36E-22 | 3.25E-21 | 40.45974764 |
| **FAM86A** | 0.892906 | 3.014683 | 10.37302 | 1.37E-22 | 3.26E-21 | 40.45636795 |
| **ZKSCAN5** | 0.821013 | 2.462701 | 10.37199 | 1.38E-22 | 3.28E-21 | 40.44792369 |
| **SPIN4** | 0.630448 | 1.019657 | 10.37144 | 1.38E-22 | 3.29E-21 | 40.44338535 |
| **BZW1** | 1.518105 | 4.786653 | 10.37045 | 1.40E-22 | 3.32E-21 | 40.43527819 |
| **HNRNPUL1** | 1.551412 | 5.514773 | 10.36989 | 1.40E-22 | 3.33E-21 | 40.43063209 |
| **NUDCD1** | 0.874285 | 1.783575 | 10.36955 | 1.41E-22 | 3.34E-21 | 40.42787422 |
| **ALG3** | 1.100314 | 3.947335 | 10.36846 | 1.42E-22 | 3.36E-21 | 40.41885919 |
| **TOR3A** | 1.115856 | 3.580479 | 10.36817 | 1.42E-22 | 3.37E-21 | 40.41648473 |
| **MEX3D** | 1.459338 | 3.825694 | 10.36665 | 1.44E-22 | 3.41E-21 | 40.40400195 |
| **AEN** | 1.020711 | 2.805301 | 10.36657 | 1.44E-22 | 3.41E-21 | 40.40331562 |
| **XRCC4** | 0.808838 | 2.104533 | 10.36622 | 1.45E-22 | 3.41E-21 | 40.40049659 |
| **CNOT1** | 1.174696 | 4.210037 | 10.36252 | 1.49E-22 | 3.52E-21 | 40.37006791 |
| **CREB1** | 1.111287 | 2.626646 | 10.36169 | 1.50E-22 | 3.54E-21 | 40.36319846 |
| **B4GALT3** | 1.204277 | 4.126202 | 10.35927 | 1.53E-22 | 3.60E-21 | 40.3433078 |
| **ZFP69** | 0.725277 | 1.796347 | 10.35924 | 1.53E-22 | 3.60E-21 | 40.34305748 |
| **HSPA14** | 1.147448 | 3.218854 | 10.35911 | 1.53E-22 | 3.60E-21 | 40.34203548 |
| **SMC6** | 0.994456 | 2.214612 | 10.35694 | 1.56E-22 | 3.67E-21 | 40.32415144 |
| **NETO2** | 1.161092 | 2.337615 | 10.35615 | 1.57E-22 | 3.69E-21 | 40.31767849 |
| **C16orf87** | 1.027836 | 2.198326 | 10.35335 | 1.61E-22 | 3.77E-21 | 40.29466417 |
| **ZSCAN9** | 1.004376 | 2.998291 | 10.35215 | 1.63E-22 | 3.80E-21 | 40.28477182 |
| **ZNF562** | 0.830617 | 2.43121 | 10.35123 | 1.64E-22 | 3.83E-21 | 40.27727222 |
| **ZFP69B** | 0.607836 | 0.901282 | 10.34842 | 1.68E-22 | 3.92E-21 | 40.25419336 |
| **DBR1** | 1.097645 | 2.653337 | 10.34773 | 1.69E-22 | 3.94E-21 | 40.24846885 |
| **VGLL4** | 1.284622 | 4.358047 | 10.3462 | 1.71E-22 | 3.98E-21 | 40.23596091 |
| **GEMIN6** | 0.748163 | 2.486133 | 10.34364 | 1.75E-22 | 4.06E-21 | 40.21487862 |
| **PDCL** | 1.125603 | 2.816833 | 10.34352 | 1.75E-22 | 4.06E-21 | 40.21390854 |
| **EFTUD1** | 0.865374 | 2.236008 | 10.34121 | 1.78E-22 | 4.14E-21 | 40.19495245 |
| **RP11-84E24.3** | 0.791611 | 1.424858 | 10.33806 | 1.83E-22 | 4.24E-21 | 40.16904252 |
| **RARS2** | 1.02651 | 3.146992 | 10.33431 | 1.89E-22 | 4.37E-21 | 40.13831556 |
| **ZNF416** | 0.549927 | 1.358547 | 10.33422 | 1.89E-22 | 4.37E-21 | 40.13759044 |
| **DEDD** | 1.211476 | 3.944894 | 10.33407 | 1.89E-22 | 4.37E-21 | 40.1362891 |
| **DCTN5** | 1.053479 | 3.736045 | 10.33388 | 1.89E-22 | 4.37E-21 | 40.13474386 |
| **SRBD1** | 0.809594 | 1.892166 | 10.33325 | 1.90E-22 | 4.39E-21 | 40.12963107 |
| **NCOA5** | 1.169536 | 3.663069 | 10.33176 | 1.93E-22 | 4.44E-21 | 40.11734289 |
| **LRP6** | 1.033798 | 2.638653 | 10.33144 | 1.93E-22 | 4.45E-21 | 40.11471796 |
| **FAM126A** | 1.086365 | 1.961545 | 10.33128 | 1.93E-22 | 4.45E-21 | 40.11339118 |
| **SEPSECS** | 0.683381 | 1.568671 | 10.33113 | 1.94E-22 | 4.45E-21 | 40.11223754 |
| **PLOD1** | 1.28622 | 4.500145 | 10.33109 | 1.94E-22 | 4.45E-21 | 40.11183897 |
| **PHF19** | 1.232587 | 4.138906 | 10.33034 | 1.95E-22 | 4.47E-21 | 40.10569784 |
| **HILPDA** | 1.103919 | 2.812817 | 10.3302 | 1.95E-22 | 4.47E-21 | 40.10459648 |
| **INSM1** | 1.410882 | 2.310252 | 10.33011 | 1.95E-22 | 4.47E-21 | 40.10386139 |
| **OPA3** | 0.752007 | 1.914641 | 10.32905 | 1.97E-22 | 4.51E-21 | 40.09513238 |
| **UBIAD1** | 0.980646 | 2.486703 | 10.32641 | 2.01E-22 | 4.60E-21 | 40.07347553 |
| **UFD1L** | 1.371774 | 4.851 | 10.32562 | 2.03E-22 | 4.63E-21 | 40.0670228 |
| **CNOT11** | 1.134887 | 3.054011 | 10.32493 | 2.04E-22 | 4.65E-21 | 40.0613359 |
| **ALDH1B1** | 0.96254 | 2.591408 | 10.32435 | 2.05E-22 | 4.67E-21 | 40.05655036 |
| **BST1** | 0.749534 | 1.316523 | 10.32271 | 2.08E-22 | 4.73E-21 | 40.04310408 |
| **PYCR1** | 1.376215 | 4.658531 | 10.32169 | 2.09E-22 | 4.77E-21 | 40.03473426 |
| **XPR1** | 1.09038 | 2.477962 | 10.32099 | 2.11E-22 | 4.79E-21 | 40.02903638 |
| **ZNF232** | 0.834457 | 2.502672 | 10.3179 | 2.16E-22 | 4.91E-21 | 40.00363863 |
| **SNIP1** | 0.86218 | 2.005193 | 10.31601 | 2.20E-22 | 4.98E-21 | 39.98819772 |
| **DCAF7** | 1.361054 | 4.057954 | 10.31568 | 2.20E-22 | 4.99E-21 | 39.98541596 |
| **PRSS23** | 1.437429 | 2.775162 | 10.31513 | 2.21E-22 | 5.01E-21 | 39.98093502 |
| **C17orf80** | 0.998261 | 2.866098 | 10.31321 | 2.25E-22 | 5.08E-21 | 39.96518022 |
| **HEATR6** | 1.020715 | 2.418069 | 10.31316 | 2.25E-22 | 5.08E-21 | 39.96481012 |
| **MCL1** | 1.491662 | 5.09036 | 10.31282 | 2.25E-22 | 5.09E-21 | 39.96200294 |
| **HDGF** | 1.31727 | 6.304226 | 10.31265 | 2.26E-22 | 5.09E-21 | 39.96057772 |
| **C17orf70** | 1.114138 | 4.20544 | 10.31264 | 2.26E-22 | 5.09E-21 | 39.96049263 |
| **LINS** | 0.88789 | 2.098275 | 10.31235 | 2.26E-22 | 5.10E-21 | 39.95815231 |
| **CREB3L2** | 0.824334 | 1.854229 | 10.31156 | 2.28E-22 | 5.12E-21 | 39.95168352 |
| **MBTD1** | 0.929166 | 2.369018 | 10.31138 | 2.28E-22 | 5.13E-21 | 39.95022608 |
| **YRDC** | 1.041464 | 2.726735 | 10.31066 | 2.30E-22 | 5.15E-21 | 39.94428859 |
| **CASP4** | 1.069436 | 2.353234 | 10.31054 | 2.30E-22 | 5.15E-21 | 39.94332648 |
| **PMS2P1** | 0.865899 | 2.171576 | 10.30994 | 2.31E-22 | 5.17E-21 | 39.93840232 |
| **NUPL2** | 1.388653 | 4.520262 | 10.30889 | 2.33E-22 | 5.21E-21 | 39.92982451 |
| **SESN2** | 0.889813 | 2.766369 | 10.30818 | 2.34E-22 | 5.24E-21 | 39.92396951 |
| **KDM1A** | 1.312167 | 4.681214 | 10.30809 | 2.34E-22 | 5.24E-21 | 39.9232257 |
| **MLH1** | 1.117151 | 3.474823 | 10.30784 | 2.35E-22 | 5.25E-21 | 39.92120204 |
| **SLC25A51** | 0.906157 | 2.136051 | 10.30762 | 2.35E-22 | 5.25E-21 | 39.91941152 |
| **PLAU** | 1.350621 | 1.955436 | 10.30756 | 2.36E-22 | 5.25E-21 | 39.91892409 |
| **SEC22A** | 1.032288 | 2.346688 | 10.30703 | 2.37E-22 | 5.27E-21 | 39.91458647 |
| **ZSCAN25** | 0.886657 | 2.135177 | 10.30219 | 2.46E-22 | 5.48E-21 | 39.87490284 |
| **BAZ1B** | 1.139041 | 3.671415 | 10.30097 | 2.49E-22 | 5.53E-21 | 39.86491171 |
| **PCBD2** | 0.805899 | 2.154242 | 10.29945 | 2.52E-22 | 5.59E-21 | 39.85246488 |
| **RAVER1** | 1.438997 | 3.326297 | 10.29855 | 2.54E-22 | 5.63E-21 | 39.84505998 |
| **PLEKHA8** | 0.765817 | 1.773653 | 10.29846 | 2.54E-22 | 5.63E-21 | 39.84432636 |
| **METTL2A** | 0.996261 | 2.757496 | 10.29774 | 2.56E-22 | 5.66E-21 | 39.83846418 |
| **PELO** | 0.815369 | 2.077733 | 10.29755 | 2.56E-22 | 5.66E-21 | 39.83690416 |
| **CCNJ** | 0.827785 | 1.357759 | 10.29233 | 2.67E-22 | 5.90E-21 | 39.79413528 |
| **EFNA1** | 1.233545 | 3.717044 | 10.29213 | 2.68E-22 | 5.90E-21 | 39.7924997 |
| **FGFR1OP** | 0.819128 | 1.755282 | 10.2913 | 2.70E-22 | 5.94E-21 | 39.7857243 |
| **FAM132B** | 0.950089 | 1.629123 | 10.29116 | 2.70E-22 | 5.94E-21 | 39.7845087 |
| **PLAA** | 1.0269 | 2.733757 | 10.29009 | 2.72E-22 | 5.99E-21 | 39.77575132 |
| **DHFR** | 1.335961 | 3.34729 | 10.28893 | 2.75E-22 | 6.04E-21 | 39.76626418 |
| **ZNF561** | 1.192464 | 2.732323 | 10.28613 | 2.81E-22 | 6.18E-21 | 39.74337153 |
| **CCDC71** | 0.914868 | 3.125197 | 10.28581 | 2.82E-22 | 6.19E-21 | 39.74071339 |
| **COPG2** | 1.246367 | 4.384532 | 10.28514 | 2.84E-22 | 6.22E-21 | 39.73528679 |
| **ZNF256** | 0.726075 | 1.337689 | 10.28364 | 2.87E-22 | 6.29E-21 | 39.72298147 |
| **SCARF2** | 1.086341 | 3.172656 | 10.28308 | 2.89E-22 | 6.31E-21 | 39.71839617 |
| **SBNO2** | 1.145531 | 3.465597 | 10.28136 | 2.93E-22 | 6.40E-21 | 39.70429388 |
| **SPRTN** | 0.814872 | 1.811062 | 10.28113 | 2.93E-22 | 6.40E-21 | 39.70240169 |
| **PBX3** | 1.114352 | 2.356279 | 10.28071 | 2.94E-22 | 6.42E-21 | 39.69904291 |
| **ZNF282** | 1.08014 | 3.836787 | 10.28032 | 2.95E-22 | 6.43E-21 | 39.69582397 |
| **LAP3** | 1.554465 | 5.184042 | 10.27481 | 3.09E-22 | 6.73E-21 | 39.65069568 |
| **ANXA2** | 1.805506 | 4.971998 | 10.27401 | 3.11E-22 | 6.76E-21 | 39.6441633 |
| **ZKSCAN2** | 0.56452 | 1.615003 | 10.27398 | 3.11E-22 | 6.76E-21 | 39.64393583 |
| **STAT1** | 1.503883 | 3.802864 | 10.27176 | 3.17E-22 | 6.88E-21 | 39.6257888 |
| **PARP11** | 0.909219 | 1.723603 | 10.27173 | 3.17E-22 | 6.88E-21 | 39.62552106 |
| **TMEM69** | 1.093843 | 2.88655 | 10.27051 | 3.20E-22 | 6.94E-21 | 39.61558423 |
| **KCTD5** | 1.403955 | 4.055182 | 10.27039 | 3.21E-22 | 6.94E-21 | 39.61458475 |
| **PDLIM1** | 1.335164 | 2.591957 | 10.26981 | 3.22E-22 | 6.97E-21 | 39.60987123 |
| **ITPRIP** | 0.821245 | 1.721348 | 10.26632 | 3.31E-22 | 7.17E-21 | 39.58130369 |
| **OXSR1** | 1.043861 | 2.725972 | 10.26477 | 3.36E-22 | 7.25E-21 | 39.56866556 |
| **SPCS3** | 1.246748 | 3.457944 | 10.26437 | 3.37E-22 | 7.27E-21 | 39.56535577 |
| **ATF7IP** | 1.299222 | 2.412227 | 10.26384 | 3.38E-22 | 7.30E-21 | 39.56103801 |
| **EP400NL** | 0.835344 | 2.52391 | 10.26257 | 3.42E-22 | 7.37E-21 | 39.55063873 |
| **MED17** | 1.117513 | 3.056796 | 10.26031 | 3.48E-22 | 7.50E-21 | 39.53214656 |
| **HEATR3** | 0.855055 | 2.35029 | 10.25934 | 3.51E-22 | 7.55E-21 | 39.52424703 |
| **BCORL1** | 0.749635 | 1.45264 | 10.25902 | 3.52E-22 | 7.57E-21 | 39.52165935 |
| **ZBTB10** | 0.869303 | 2.021517 | 10.25799 | 3.55E-22 | 7.62E-21 | 39.51317913 |
| **CMTR2** | 0.9709 | 2.165044 | 10.25776 | 3.56E-22 | 7.63E-21 | 39.51128818 |
| **ZNF333** | 1.114375 | 3.261705 | 10.25521 | 3.63E-22 | 7.79E-21 | 39.49047371 |
| **BTN3A2** | 1.346856 | 3.366582 | 10.25513 | 3.64E-22 | 7.79E-21 | 39.48984314 |
| **ING3** | 1.243815 | 2.733777 | 10.25479 | 3.65E-22 | 7.80E-21 | 39.48709734 |
| **PHC2** | 1.464333 | 5.780425 | 10.25282 | 3.71E-22 | 7.92E-21 | 39.47097182 |
| **TLK2** | 1.049416 | 3.433002 | 10.25276 | 3.71E-22 | 7.92E-21 | 39.47047594 |
| **CCT5** | 1.599476 | 5.5354 | 10.25228 | 3.72E-22 | 7.94E-21 | 39.46656245 |
| **VIM** | 2.192535 | 7.770252 | 10.25146 | 3.75E-22 | 7.99E-21 | 39.45982446 |
| **DDX23** | 1.27451 | 4.941628 | 10.25025 | 3.79E-22 | 8.06E-21 | 39.44995688 |
| **ZBTB39** | 0.739595 | 1.252957 | 10.24908 | 3.82E-22 | 8.13E-21 | 39.44039671 |
| **NR2C2AP** | 1.073144 | 3.796981 | 10.24853 | 3.84E-22 | 8.16E-21 | 39.43587767 |
| **LZIC** | 1.091761 | 3.264973 | 10.24788 | 3.86E-22 | 8.19E-21 | 39.43057717 |
| **TMSB15B** | 1.056471 | 2.666006 | 10.24785 | 3.86E-22 | 8.19E-21 | 39.43032889 |
| **COIL** | 0.898962 | 2.629077 | 10.24552 | 3.94E-22 | 8.34E-21 | 39.4113733 |
| **ZNF548** | 1.110839 | 2.140459 | 10.2453 | 3.94E-22 | 8.34E-21 | 39.40957671 |
| **DHRS13** | 1.209505 | 2.651198 | 10.24526 | 3.95E-22 | 8.34E-21 | 39.40923505 |
| **EXOSC9** | 1.104829 | 3.486272 | 10.24416 | 3.98E-22 | 8.41E-21 | 39.40022877 |
| **ARAP3** | 1.04039 | 2.223431 | 10.2435 | 4.00E-22 | 8.45E-21 | 39.3948635 |
| **BTN3A1** | 1.247399 | 2.978981 | 10.24307 | 4.02E-22 | 8.47E-21 | 39.39132567 |
| **TEAD4** | 1.109303 | 1.67514 | 10.24245 | 4.04E-22 | 8.51E-21 | 39.38627429 |
| **GTF3C2** | 1.232198 | 4.576223 | 10.23981 | 4.13E-22 | 8.69E-21 | 39.36472455 |
| **BTF3L4** | 1.440853 | 4.40783 | 10.23958 | 4.14E-22 | 8.70E-21 | 39.3628183 |
| **METTL8** | 0.861268 | 1.967148 | 10.23849 | 4.17E-22 | 8.77E-21 | 39.35389848 |
| **FANCA** | 1.103919 | 3.196574 | 10.23765 | 4.20E-22 | 8.82E-21 | 39.34705142 |
| **CCDC97** | 1.065838 | 3.324074 | 10.23764 | 4.20E-22 | 8.82E-21 | 39.34701949 |
| **BTG1** | 1.451109 | 4.490179 | 10.23607 | 4.26E-22 | 8.92E-21 | 39.33420063 |
| **TRERF1** | 0.715581 | 1.357187 | 10.23565 | 4.27E-22 | 8.95E-21 | 39.33076285 |
| **TRIM25** | 1.054429 | 2.904852 | 10.23513 | 4.29E-22 | 8.98E-21 | 39.32654654 |
| **TGFBRAP1** | 0.888804 | 2.414532 | 10.23425 | 4.32E-22 | 9.04E-21 | 39.31932291 |
| **C16orf72** | 0.798323 | 2.570587 | 10.23355 | 4.35E-22 | 9.08E-21 | 39.31361244 |
| **SIKE1** | 1.17599 | 3.747492 | 10.22946 | 4.50E-22 | 9.38E-21 | 39.28027153 |
| **EXT2** | 1.159714 | 3.567332 | 10.2292 | 4.51E-22 | 9.39E-21 | 39.27814052 |
| **FAM210A** | 0.875641 | 2.030727 | 10.22914 | 4.51E-22 | 9.39E-21 | 39.27762293 |
| **SEC24D** | 0.900979 | 1.586258 | 10.22872 | 4.52E-22 | 9.42E-21 | 39.27418608 |
| **DYRK3** | 0.71161 | 1.363861 | 10.22759 | 4.57E-22 | 9.49E-21 | 39.26502439 |
| **PRKRIR** | 1.270248 | 3.427966 | 10.22756 | 4.57E-22 | 9.49E-21 | 39.26472464 |
| **STT3A** | 1.359592 | 4.340842 | 10.22658 | 4.60E-22 | 9.56E-21 | 39.25672471 |
| **ZFP64** | 0.885253 | 2.248482 | 10.22597 | 4.63E-22 | 9.60E-21 | 39.25175882 |
| **QSER1** | 0.926855 | 2.092744 | 10.22565 | 4.64E-22 | 9.62E-21 | 39.24920189 |
| **CCND2** | 1.336312 | 3.935494 | 10.22451 | 4.68E-22 | 9.70E-21 | 39.23983772 |
| **TXNRD1** | 1.386299 | 3.758981 | 10.22226 | 4.77E-22 | 9.87E-21 | 39.22154702 |
| **AJUBA** | 0.815843 | 1.317415 | 10.21941 | 4.89E-22 | 1.01E-20 | 39.19825449 |
| **BAK1** | 0.860216 | 2.854263 | 10.21865 | 4.92E-22 | 1.02E-20 | 39.19209166 |
| **GSK3B** | 1.159339 | 2.954743 | 10.21841 | 4.93E-22 | 1.02E-20 | 39.19015802 |
| **USP6NL** | 0.992628 | 2.358163 | 10.21529 | 5.05E-22 | 1.04E-20 | 39.16467664 |
| **METTL4** | 0.788435 | 2.025811 | 10.21449 | 5.09E-22 | 1.05E-20 | 39.15818838 |
| **CHAF1B** | 0.581951 | 0.995369 | 10.21185 | 5.20E-22 | 1.07E-20 | 39.13667951 |
| **BLMH** | 1.1559 | 3.88254 | 10.2117 | 5.21E-22 | 1.07E-20 | 39.13539311 |
| **SRCAP** | 1.106187 | 3.817357 | 10.21152 | 5.21E-22 | 1.07E-20 | 39.13391431 |
| **SIAH1** | 1.244197 | 3.788991 | 10.21052 | 5.26E-22 | 1.08E-20 | 39.12581276 |
| **STXBP4** | 0.85509 | 1.378725 | 10.20916 | 5.32E-22 | 1.09E-20 | 39.11470756 |
| **ZNF639** | 1.23478 | 3.517417 | 10.20907 | 5.32E-22 | 1.09E-20 | 39.11398256 |
| **RP11-649A18.12** | 0.601495 | 1.068516 | 10.20577 | 5.47E-22 | 1.12E-20 | 39.08707559 |
| **COL3A1** | 1.637525 | 2.023636 | 10.20513 | 5.50E-22 | 1.12E-20 | 39.08190317 |
| **XRCC5** | 1.333777 | 5.33031 | 10.20481 | 5.51E-22 | 1.13E-20 | 39.07923751 |
| **NEDD1** | 0.99974 | 1.637272 | 10.20434 | 5.53E-22 | 1.13E-20 | 39.07545268 |
| **UBE2J1** | 1.158974 | 3.50363 | 10.20379 | 5.56E-22 | 1.13E-20 | 39.07098317 |
| **PTX3** | 0.9732 | 1.078333 | 10.20346 | 5.57E-22 | 1.14E-20 | 39.06826386 |
| **E2F5** | 1.167428 | 2.669576 | 10.20153 | 5.66E-22 | 1.15E-20 | 39.05251711 |
| **TUBB6** | 1.380811 | 4.369832 | 10.20152 | 5.66E-22 | 1.15E-20 | 39.05250593 |
| **SSR1** | 1.33028 | 4.275448 | 10.20137 | 5.67E-22 | 1.15E-20 | 39.05125776 |
| **ZNF614** | 0.818945 | 1.579852 | 10.20059 | 5.71E-22 | 1.16E-20 | 39.04487201 |
| **ZNF696** | 0.940055 | 2.901398 | 10.20027 | 5.72E-22 | 1.16E-20 | 39.04232739 |
| **PHLDA1** | 1.563358 | 4.491139 | 10.19989 | 5.74E-22 | 1.16E-20 | 39.03918588 |
| **UHRF1BP1** | 0.731785 | 1.51964 | 10.19641 | 5.91E-22 | 1.20E-20 | 39.0108487 |
| **REEP4** | 1.033328 | 3.234387 | 10.19568 | 5.94E-22 | 1.20E-20 | 39.00487877 |
| **CYB5R4** | 0.936892 | 1.595125 | 10.19553 | 5.95E-22 | 1.20E-20 | 39.00367011 |
| **C5orf15** | 1.213879 | 3.553575 | 10.19534 | 5.96E-22 | 1.20E-20 | 39.00214964 |
| **CHRAC1** | 0.974023 | 2.684849 | 10.19466 | 5.99E-22 | 1.21E-20 | 38.99657813 |
| **TCF7L1** | 1.21404 | 3.446485 | 10.19459 | 6.00E-22 | 1.21E-20 | 38.99601977 |
| **ZNF107** | 0.867041 | 1.338981 | 10.19407 | 6.02E-22 | 1.21E-20 | 38.99180771 |
| **CHST3** | 1.071806 | 3.001787 | 10.19328 | 6.06E-22 | 1.22E-20 | 38.98533751 |
| **CWC22** | 0.945246 | 2.539599 | 10.19323 | 6.06E-22 | 1.22E-20 | 38.98498907 |
| **DISC1** | 0.823061 | 1.768398 | 10.19189 | 6.13E-22 | 1.23E-20 | 38.97407909 |
| **ZNF250** | 0.886926 | 2.572161 | 10.19096 | 6.18E-22 | 1.24E-20 | 38.96645084 |
| **CORO1C** | 1.4753 | 4.910802 | 10.19017 | 6.22E-22 | 1.25E-20 | 38.96004149 |
| **ZBTB2** | 0.807982 | 1.840679 | 10.1886 | 6.30E-22 | 1.26E-20 | 38.94725824 |
| **FIP1L1** | 1.252564 | 4.279214 | 10.18859 | 6.30E-22 | 1.26E-20 | 38.94722022 |
| **UAP1** | 1.048275 | 3.296853 | 10.18817 | 6.32E-22 | 1.26E-20 | 38.94378255 |
| **DDX39A** | 1.424975 | 5.821177 | 10.18805 | 6.33E-22 | 1.26E-20 | 38.94282121 |
| **ZNF598** | 1.234506 | 4.260001 | 10.18655 | 6.41E-22 | 1.28E-20 | 38.93060934 |
| **NRP2** | 1.250898 | 3.152483 | 10.18647 | 6.41E-22 | 1.28E-20 | 38.92994635 |
| **CREBBP** | 1.101205 | 3.393067 | 10.18416 | 6.53E-22 | 1.30E-20 | 38.91116278 |
| **FAM193A** | 1.143465 | 3.38132 | 10.18159 | 6.67E-22 | 1.33E-20 | 38.89019606 |
| **ST7L** | 0.952488 | 3.326846 | 10.18138 | 6.69E-22 | 1.33E-20 | 38.88852277 |
| **RSU1** | 1.449128 | 3.74259 | 10.18098 | 6.71E-22 | 1.33E-20 | 38.88527245 |
| **TTL** | 1.022659 | 3.285097 | 10.1809 | 6.71E-22 | 1.33E-20 | 38.88461738 |
| **YBX1** | 2.134775 | 7.79885 | 10.18019 | 6.75E-22 | 1.34E-20 | 38.87880246 |
| **ARL5B** | 0.905833 | 1.79727 | 10.17995 | 6.76E-22 | 1.34E-20 | 38.87685482 |
| **SMC1A** | 1.179478 | 2.727444 | 10.17952 | 6.79E-22 | 1.35E-20 | 38.87342865 |
| **KDM6B** | 1.023784 | 2.860035 | 10.17718 | 6.92E-22 | 1.37E-20 | 38.8543572 |
| **RARS** | 0.829595 | 3.904494 | 10.17716 | 6.92E-22 | 1.37E-20 | 38.85422368 |
| **NAGA** | 1.054513 | 2.963446 | 10.17645 | 6.96E-22 | 1.38E-20 | 38.84840139 |
| **KLHL13** | 1.033415 | 2.208646 | 10.17645 | 6.96E-22 | 1.38E-20 | 38.84838179 |
| **ZNF439** | 0.912499 | 2.215332 | 10.1755 | 7.02E-22 | 1.38E-20 | 38.84073294 |
| **ARID1A** | 1.318448 | 3.399035 | 10.17532 | 7.03E-22 | 1.39E-20 | 38.83922593 |
| **ZNF71** | 0.74066 | 1.680209 | 10.17401 | 7.10E-22 | 1.40E-20 | 38.82855756 |
| **NT5C3A** | 1.216232 | 3.438081 | 10.17277 | 7.18E-22 | 1.41E-20 | 38.81847096 |
| **SASS6** | 0.507724 | 0.924607 | 10.17231 | 7.20E-22 | 1.42E-20 | 38.81473233 |
| **RIPK1** | 0.879209 | 2.593735 | 10.17201 | 7.22E-22 | 1.42E-20 | 38.81229239 |
| **C15orf41** | 0.731147 | 1.570405 | 10.17136 | 7.26E-22 | 1.43E-20 | 38.80706767 |
| **DUSP12** | 1.221949 | 3.978369 | 10.16941 | 7.38E-22 | 1.45E-20 | 38.79119594 |
| **GPR161** | 0.854263 | 2.236859 | 10.16939 | 7.38E-22 | 1.45E-20 | 38.7909762 |
| **STX6** | 1.01997 | 3.543693 | 10.16795 | 7.47E-22 | 1.46E-20 | 38.7792864 |
| **ZNF790** | 0.746803 | 1.86971 | 10.16761 | 7.49E-22 | 1.47E-20 | 38.77655369 |
| **COX10** | 0.77002 | 2.127209 | 10.16562 | 7.61E-22 | 1.49E-20 | 38.76037075 |
| **ILF2** | 1.59467 | 5.903112 | 10.1654 | 7.63E-22 | 1.49E-20 | 38.75853646 |
| **ECE1** | 1.192028 | 3.693077 | 10.16508 | 7.65E-22 | 1.49E-20 | 38.75598847 |
| **MIOS** | 1.021755 | 2.753797 | 10.16489 | 7.66E-22 | 1.49E-20 | 38.75439815 |
| **RP11-277P12.20** | 0.741066 | 0.977598 | 10.16325 | 7.76E-22 | 1.51E-20 | 38.74108334 |
| **WRN** | 0.817542 | 1.474677 | 10.16111 | 7.90E-22 | 1.54E-20 | 38.72371167 |
| **ZNF195** | 1.0436 | 3.076314 | 10.16105 | 7.90E-22 | 1.54E-20 | 38.72323481 |
| **ITGA4** | 0.666204 | 0.658195 | 10.16067 | 7.93E-22 | 1.54E-20 | 38.72010745 |
| **APOL4** | 1.028397 | 1.280866 | 10.15864 | 8.06E-22 | 1.57E-20 | 38.70360343 |
| **TAF4** | 0.947388 | 2.194283 | 10.15625 | 8.22E-22 | 1.60E-20 | 38.68421524 |
| **ZNF22** | 1.288754 | 3.610281 | 10.15565 | 8.26E-22 | 1.60E-20 | 38.67938669 |
| **EXOSC3** | 1.11418 | 3.298001 | 10.15325 | 8.43E-22 | 1.63E-20 | 38.65986872 |
| **SRPK1** | 0.984075 | 2.685771 | 10.15246 | 8.48E-22 | 1.64E-20 | 38.65342338 |
| **SRP72** | 1.339862 | 4.276209 | 10.15165 | 8.54E-22 | 1.65E-20 | 38.6468466 |
| **TMEM43** | 1.274664 | 3.631989 | 10.15094 | 8.59E-22 | 1.66E-20 | 38.641132 |
| **HAPLN3** | 1.010495 | 2.48721 | 10.15 | 8.66E-22 | 1.67E-20 | 38.63347792 |
| **ZNF48** | 0.896064 | 3.037918 | 10.14823 | 8.78E-22 | 1.70E-20 | 38.61911785 |
| **ARID2** | 0.764326 | 1.994979 | 10.14694 | 8.88E-22 | 1.71E-20 | 38.60861708 |
| **MPZ** | 0.902519 | 1.831939 | 10.14356 | 9.13E-22 | 1.76E-20 | 38.58120224 |
| **HNRNPF** | 1.463463 | 4.793642 | 10.14318 | 9.15E-22 | 1.76E-20 | 38.57812677 |
| **DDOST** | 1.459757 | 5.318371 | 10.14234 | 9.22E-22 | 1.77E-20 | 38.57131135 |
| **SUV420H1** | 1.179278 | 3.602812 | 10.1417 | 9.27E-22 | 1.78E-20 | 38.56608911 |
| **TMEM39A** | 1.059905 | 2.599507 | 10.14102 | 9.32E-22 | 1.79E-20 | 38.56055948 |
| **FAM127C** | 1.045593 | 3.053627 | 10.14053 | 9.36E-22 | 1.79E-20 | 38.55661681 |
| **KBTBD2** | 1.151663 | 3.297729 | 10.13829 | 9.53E-22 | 1.83E-20 | 38.53844009 |
| **ZNF549** | 0.655989 | 1.537565 | 10.13823 | 9.53E-22 | 1.83E-20 | 38.53794435 |
| **PPP4R1** | 1.052937 | 4.094164 | 10.13816 | 9.54E-22 | 1.83E-20 | 38.53735235 |
| **SET** | 1.535675 | 6.152115 | 10.13333 | 9.93E-22 | 1.90E-20 | 38.49819471 |
| **PTPN9** | 1.071755 | 3.086799 | 10.13178 | 1.01E-21 | 1.92E-20 | 38.48560554 |
| **TFB2M** | 0.86073 | 2.199774 | 10.13124 | 1.01E-21 | 1.93E-20 | 38.48117044 |
| **ZBTB33** | 1.006271 | 2.49592 | 10.13083 | 1.01E-21 | 1.93E-20 | 38.47789873 |
| **ZNF584** | 0.979533 | 2.619996 | 10.13077 | 1.01E-21 | 1.93E-20 | 38.47739026 |
| **NCBP1** | 0.970061 | 2.739381 | 10.12886 | 1.03E-21 | 1.96E-20 | 38.46188802 |
| **RYK** | 1.05406 | 3.026714 | 10.12859 | 1.03E-21 | 1.96E-20 | 38.45973955 |
| **ZNF574** | 0.888153 | 2.622741 | 10.127 | 1.05E-21 | 1.99E-20 | 38.44682571 |
| **ZNF502** | 0.741426 | 1.710195 | 10.12598 | 1.05E-21 | 2.00E-20 | 38.438538 |
| **DGCR8** | 1.097282 | 3.549904 | 10.12596 | 1.05E-21 | 2.00E-20 | 38.43839777 |
| **C2orf43** | 0.944627 | 2.385883 | 10.12593 | 1.05E-21 | 2.00E-20 | 38.43817245 |
| **PXDN** | 1.481266 | 3.298918 | 10.12502 | 1.06E-21 | 2.01E-20 | 38.43075883 |
| **SOX2** | 1.842378 | 6.763813 | 10.12367 | 1.07E-21 | 2.04E-20 | 38.41982859 |
| **PROS1** | 1.188152 | 2.607963 | 10.12118 | 1.10E-21 | 2.08E-20 | 38.39964845 |
| **GTF2E1** | 0.855831 | 1.908104 | 10.12085 | 1.10E-21 | 2.08E-20 | 38.39696171 |
| **STK32B** | 1.022328 | 1.37385 | 10.12032 | 1.10E-21 | 2.09E-20 | 38.39264141 |
| **ZW10** | 0.774193 | 1.911326 | 10.11905 | 1.12E-21 | 2.11E-20 | 38.38239485 |
| **PDE7A** | 0.956459 | 2.501923 | 10.11691 | 1.14E-21 | 2.14E-20 | 38.36504055 |
| **RP11-126K1.6** | 1.182227 | 3.480393 | 10.11578 | 1.15E-21 | 2.16E-20 | 38.35589722 |
| **AC006115.3** | 0.595678 | 1.058981 | 10.11509 | 1.15E-21 | 2.17E-20 | 38.35025048 |
| **CENPQ** | 0.888413 | 2.139131 | 10.11497 | 1.15E-21 | 2.17E-20 | 38.34928173 |
| **PDCL3** | 1.113146 | 3.683223 | 10.11412 | 1.16E-21 | 2.19E-20 | 38.34237409 |
| **PI4K2B** | 0.878807 | 2.674018 | 10.11196 | 1.18E-21 | 2.22E-20 | 38.32489144 |
| **MAPKAPK2** | 1.247367 | 4.584055 | 10.10874 | 1.21E-21 | 2.28E-20 | 38.29878168 |
| **LSG1** | 1.169743 | 3.465889 | 10.10608 | 1.24E-21 | 2.33E-20 | 38.27725739 |
| **PKNOX1** | 0.72076 | 2.349353 | 10.10581 | 1.24E-21 | 2.33E-20 | 38.27507567 |
| **NUP50** | 1.02503 | 3.015814 | 10.10356 | 1.27E-21 | 2.37E-20 | 38.25683577 |
| **INTS5** | 0.87942 | 3.034853 | 10.10354 | 1.27E-21 | 2.37E-20 | 38.25669675 |
| **PARP16** | 0.694574 | 2.067307 | 10.10294 | 1.27E-21 | 2.38E-20 | 38.25186027 |
| **MSN** | 1.58556 | 3.98674 | 10.10281 | 1.28E-21 | 2.38E-20 | 38.25078533 |
| **DHX15** | 1.390615 | 4.64542 | 10.10236 | 1.28E-21 | 2.39E-20 | 38.24712995 |
| **EIF2B1** | 1.235556 | 4.292822 | 10.10199 | 1.28E-21 | 2.39E-20 | 38.24413067 |
| **NUDT5** | 1.269141 | 4.375999 | 10.10197 | 1.28E-21 | 2.39E-20 | 38.2439767 |
| **ARL6IP6** | 1.213171 | 3.183614 | 10.10107 | 1.29E-21 | 2.41E-20 | 38.23668368 |
| **LINC00511** | 1.372676 | 4.375863 | 10.10056 | 1.30E-21 | 2.42E-20 | 38.23258724 |
| **PLEKHA2** | 0.910271 | 2.960245 | 10.09877 | 1.32E-21 | 2.45E-20 | 38.21805175 |
| **ZKSCAN3** | 0.571667 | 1.217174 | 10.09805 | 1.33E-21 | 2.46E-20 | 38.21222006 |
| **ERBB2** | 0.951785 | 2.999508 | 10.09801 | 1.33E-21 | 2.46E-20 | 38.21195571 |
| **PTP4A1** | 1.28086 | 3.824414 | 10.09791 | 1.33E-21 | 2.46E-20 | 38.21113678 |
| **CTDSPL2** | 1.139245 | 2.323438 | 10.09778 | 1.33E-21 | 2.46E-20 | 38.21003879 |
| **SBK1** | 1.163105 | 3.431718 | 10.0967 | 1.34E-21 | 2.48E-20 | 38.20129823 |
| **UBAP2L** | 1.407883 | 5.143063 | 10.09574 | 1.35E-21 | 2.50E-20 | 38.19355839 |
| **PRPF18** | 0.964063 | 2.786452 | 10.09569 | 1.35E-21 | 2.50E-20 | 38.19316359 |
| **RFC5** | 0.984117 | 2.711665 | 10.09447 | 1.37E-21 | 2.52E-20 | 38.18323377 |
| **HAS3** | 0.551783 | 0.837393 | 10.09379 | 1.37E-21 | 2.54E-20 | 38.17775821 |
| **HEATR2** | 1.148335 | 2.809366 | 10.09206 | 1.39E-21 | 2.57E-20 | 38.163758 |
| **PLAT** | 1.458832 | 2.668197 | 10.09159 | 1.40E-21 | 2.58E-20 | 38.15992954 |
| **BAMBI** | 1.286233 | 3.184597 | 10.09081 | 1.41E-21 | 2.59E-20 | 38.15363498 |
| **SPAG5** | 1.12215 | 3.094764 | 10.09048 | 1.41E-21 | 2.60E-20 | 38.15095156 |
| **ZBTB11-AS1** | 0.529173 | 1.271545 | 10.09025 | 1.41E-21 | 2.60E-20 | 38.14914556 |
| **BNIP2** | 1.225089 | 3.230514 | 10.08885 | 1.43E-21 | 2.63E-20 | 38.13781943 |
| **COPB2** | 1.403985 | 4.392872 | 10.08753 | 1.45E-21 | 2.66E-20 | 38.12707448 |
| **RMI1** | 0.896632 | 1.698585 | 10.08577 | 1.47E-21 | 2.69E-20 | 38.11290338 |
| **TMEM237** | 1.076948 | 2.907235 | 10.08533 | 1.47E-21 | 2.70E-20 | 38.10934166 |
| **SOCS4** | 0.908087 | 2.033449 | 10.08508 | 1.47E-21 | 2.70E-20 | 38.10727123 |
| **GPR89A** | 0.949917 | 3.108618 | 10.08479 | 1.48E-21 | 2.71E-20 | 38.10490425 |
| **RP11-1055B8.7** | 1.208297 | 3.121998 | 10.08293 | 1.50E-21 | 2.75E-20 | 38.08986748 |
| **GOSR1** | 1.157488 | 3.369731 | 10.08249 | 1.51E-21 | 2.76E-20 | 38.08636439 |
| **CHD7** | 1.572921 | 3.794813 | 10.08199 | 1.51E-21 | 2.76E-20 | 38.08226511 |
| **ZNF207** | 1.438479 | 4.795208 | 10.08185 | 1.51E-21 | 2.77E-20 | 38.08112574 |
| **CDK12** | 1.068406 | 2.417668 | 10.08177 | 1.52E-21 | 2.77E-20 | 38.08055636 |
| **ASB7** | 0.673631 | 1.612564 | 10.08152 | 1.52E-21 | 2.77E-20 | 38.07849033 |
| **TNFRSF10B** | 1.169188 | 2.990107 | 10.08148 | 1.52E-21 | 2.77E-20 | 38.0781349 |
| **ADAM9** | 1.480517 | 3.514333 | 10.0811 | 1.52E-21 | 2.77E-20 | 38.075113 |
| **CCDC109B** | 1.287217 | 2.325628 | 10.08076 | 1.53E-21 | 2.78E-20 | 38.07234444 |
| **DUSP10** | 0.868302 | 1.609359 | 10.08013 | 1.54E-21 | 2.79E-20 | 38.06722487 |
| **ZNF253** | 1.013842 | 1.894752 | 10.07969 | 1.54E-21 | 2.80E-20 | 38.06371296 |
| **ORAI2** | 1.246933 | 4.076777 | 10.07938 | 1.55E-21 | 2.80E-20 | 38.06121102 |
| **RUVBL1** | 1.221332 | 4.346328 | 10.07855 | 1.56E-21 | 2.82E-20 | 38.05450302 |
| **DCUN1D5** | 1.077868 | 3.771911 | 10.07718 | 1.57E-21 | 2.85E-20 | 38.0433908 |
| **RFC1** | 1.168441 | 3.373119 | 10.07716 | 1.57E-21 | 2.85E-20 | 38.04322526 |
| **RQCD1** | 1.121209 | 3.496564 | 10.07673 | 1.58E-21 | 2.86E-20 | 38.0397248 |
| **PPP1R8** | 1.133276 | 3.89916 | 10.07587 | 1.59E-21 | 2.87E-20 | 38.03282496 |
| **SPRY1** | 1.370886 | 2.307391 | 10.07586 | 1.59E-21 | 2.87E-20 | 38.0327025 |
| **RP11-182L21.6** | 0.804217 | 2.239071 | 10.07336 | 1.62E-21 | 2.93E-20 | 38.01249615 |
| **HEATR1** | 0.955641 | 1.632805 | 10.07328 | 1.62E-21 | 2.93E-20 | 38.01183252 |
| **HNRNPA3** | 1.698505 | 6.354234 | 10.07307 | 1.63E-21 | 2.93E-20 | 38.01014605 |
| **MTFR1** | 0.896042 | 2.305313 | 10.07176 | 1.64E-21 | 2.96E-20 | 37.99959359 |
| **TRAFD1** | 1.138054 | 3.946499 | 10.07167 | 1.65E-21 | 2.96E-20 | 37.99888518 |
| **RAF1** | 1.441131 | 4.793147 | 10.07001 | 1.67E-21 | 3.00E-20 | 37.98543252 |
| **SURF4** | 1.308236 | 4.987176 | 10.06999 | 1.67E-21 | 3.00E-20 | 37.98528861 |
| **CEP120** | 0.817858 | 2.045833 | 10.06983 | 1.67E-21 | 3.00E-20 | 37.98397291 |
| **S1PR3** | 1.24495 | 2.060331 | 10.06979 | 1.67E-21 | 3.00E-20 | 37.98368758 |
| **H6PD** | 0.889869 | 2.419922 | 10.06845 | 1.69E-21 | 3.03E-20 | 37.97285522 |
| **MRE11A** | 0.897195 | 1.690274 | 10.06537 | 1.73E-21 | 3.11E-20 | 37.94796882 |
| **MFSD9** | 0.6731 | 1.312806 | 10.065 | 1.74E-21 | 3.11E-20 | 37.94498881 |
| **RCL1** | 0.843551 | 2.413208 | 10.06496 | 1.74E-21 | 3.11E-20 | 37.94464436 |
| **URB1** | 0.871559 | 2.336527 | 10.06491 | 1.74E-21 | 3.11E-20 | 37.94423516 |
| **MLEC** | 1.352316 | 4.654915 | 10.06356 | 1.76E-21 | 3.14E-20 | 37.93329891 |
| **SHQ1** | 0.628596 | 1.632795 | 10.0633 | 1.76E-21 | 3.15E-20 | 37.93121013 |
| **ANAPC1** | 0.853904 | 2.383209 | 10.06154 | 1.79E-21 | 3.19E-20 | 37.91699226 |
| **PAICS** | 1.486321 | 4.318033 | 10.061 | 1.80E-21 | 3.20E-20 | 37.91263784 |
| **ZCCHC3** | 0.916574 | 2.641002 | 10.06054 | 1.80E-21 | 3.21E-20 | 37.90891205 |
| **G3BP1** | 1.413759 | 3.897066 | 10.05961 | 1.82E-21 | 3.23E-20 | 37.9013879 |
| **NT5DC2** | 1.457427 | 5.262655 | 10.05934 | 1.82E-21 | 3.24E-20 | 37.89927297 |
| **ZNF627** | 0.981407 | 2.279219 | 10.05856 | 1.83E-21 | 3.26E-20 | 37.8929134 |
| **CNOT6** | 0.969191 | 2.54053 | 10.05851 | 1.83E-21 | 3.26E-20 | 37.89255183 |
| **NOL8** | 1.101956 | 3.533499 | 10.05784 | 1.84E-21 | 3.27E-20 | 37.88711026 |
| **GPR4** | 0.621667 | 1.173522 | 10.05721 | 1.85E-21 | 3.29E-20 | 37.88200811 |
| **RPF1** | 1.162219 | 3.793603 | 10.0571 | 1.85E-21 | 3.29E-20 | 37.88115294 |
| **FBXO10** | 0.887082 | 2.865744 | 10.05701 | 1.86E-21 | 3.29E-20 | 37.88045481 |
| **GABPB1-AS1** | 1.198282 | 3.047419 | 10.05613 | 1.87E-21 | 3.31E-20 | 37.87334903 |
| **RNFT1** | 1.103938 | 2.268049 | 10.05555 | 1.88E-21 | 3.32E-20 | 37.86863251 |
| **RSL1D1** | 1.392107 | 4.67736 | 10.05546 | 1.88E-21 | 3.32E-20 | 37.8679234 |
| **INTS12** | 1.106814 | 3.233462 | 10.05497 | 1.89E-21 | 3.33E-20 | 37.86395149 |
| **MCMBP** | 1.188451 | 3.190936 | 10.05343 | 1.91E-21 | 3.37E-20 | 37.85149965 |
| **DPH2** | 1.070562 | 3.440786 | 10.05309 | 1.92E-21 | 3.38E-20 | 37.84877712 |
| **PHF13** | 0.90236 | 2.477286 | 10.05246 | 1.93E-21 | 3.39E-20 | 37.84368717 |
| **RSPRY1** | 1.280608 | 3.122605 | 10.05225 | 1.93E-21 | 3.40E-20 | 37.84195256 |
| **GEMIN5** | 0.797386 | 1.902636 | 10.05177 | 1.94E-21 | 3.41E-20 | 37.8380955 |
| **PIAS4** | 1.018503 | 3.960568 | 10.05118 | 1.95E-21 | 3.42E-20 | 37.83334877 |
| **POLR1C** | 1.173668 | 4.480397 | 10.05114 | 1.95E-21 | 3.42E-20 | 37.83303444 |
| **ZNF595** | 1.036001 | 1.519503 | 10.04997 | 1.97E-21 | 3.45E-20 | 37.82360336 |
| **SSRP1** | 1.437099 | 5.111143 | 10.04791 | 2.00E-21 | 3.50E-20 | 37.80697892 |
| **CTD-2267D19.2** | 0.719036 | 2.077475 | 10.04708 | 2.01E-21 | 3.53E-20 | 37.80023742 |
| **CHD1** | 1.122142 | 2.808246 | 10.04685 | 2.02E-21 | 3.53E-20 | 37.79839634 |
| **NOP58** | 1.260264 | 4.327422 | 10.04541 | 2.04E-21 | 3.57E-20 | 37.78681578 |
| **LEPROTL1** | 1.157195 | 3.71742 | 10.04479 | 2.05E-21 | 3.58E-20 | 37.78180645 |
| **CENPJ** | 0.882701 | 1.953378 | 10.04289 | 2.08E-21 | 3.63E-20 | 37.76646903 |
| **RNF168** | 0.683976 | 1.829481 | 10.04281 | 2.08E-21 | 3.63E-20 | 37.76575939 |
| **ZNF431** | 0.885602 | 2.462762 | 10.0428 | 2.08E-21 | 3.63E-20 | 37.76571748 |
| **RBMX** | 1.49449 | 5.723199 | 10.04231 | 2.09E-21 | 3.65E-20 | 37.76177677 |
| **FAM114A2** | 0.885387 | 2.571046 | 10.04118 | 2.11E-21 | 3.68E-20 | 37.75267552 |
| **DLEU2** | 0.706899 | 1.519608 | 10.03856 | 2.16E-21 | 3.75E-20 | 37.73150131 |
| **TIGD7** | 1.032922 | 2.645871 | 10.03808 | 2.17E-21 | 3.77E-20 | 37.72759877 |
| **ZNF551** | 0.652818 | 1.364748 | 10.03803 | 2.17E-21 | 3.77E-20 | 37.72726779 |
| **TAF2** | 1.100565 | 2.229508 | 10.03702 | 2.19E-21 | 3.79E-20 | 37.71909899 |
| **PGAP1** | 1.044353 | 2.202344 | 10.037 | 2.19E-21 | 3.79E-20 | 37.71894561 |
| **SUCO** | 0.988265 | 2.920077 | 10.03532 | 2.22E-21 | 3.84E-20 | 37.70538943 |
| **WDR1** | 1.401061 | 5.564145 | 10.03529 | 2.22E-21 | 3.84E-20 | 37.70514918 |
| **GNL3L** | 1.012306 | 1.92195 | 10.03387 | 2.24E-21 | 3.88E-20 | 37.69366037 |
| **SLC38A9** | 0.749078 | 2.316161 | 10.03383 | 2.24E-21 | 3.88E-20 | 37.69334326 |
| **RHPN2** | 1.102158 | 2.274199 | 10.0327 | 2.26E-21 | 3.91E-20 | 37.68424228 |
| **GPATCH2** | 0.768596 | 1.822536 | 10.03226 | 2.27E-21 | 3.92E-20 | 37.68068067 |
| **SMAD4** | 1.290649 | 3.476198 | 10.03208 | 2.28E-21 | 3.93E-20 | 37.67927593 |
| **RP11-303E16.2** | 0.779889 | 1.695205 | 10.03121 | 2.29E-21 | 3.95E-20 | 37.67224922 |
| **ZNF268** | 0.885962 | 1.954629 | 10.03076 | 2.30E-21 | 3.96E-20 | 37.66859012 |
| **METTL1** | 1.108969 | 3.481144 | 10.02933 | 2.33E-21 | 4.01E-20 | 37.65706151 |
| **ARPC5** | 1.514492 | 5.23271 | 10.02877 | 2.34E-21 | 4.02E-20 | 37.65258712 |
| **SKA2** | 1.540524 | 5.181623 | 10.02862 | 2.34E-21 | 4.03E-20 | 37.65135444 |
| **CALHM2** | 0.935474 | 2.537084 | 10.02819 | 2.35E-21 | 4.04E-20 | 37.64788448 |
| **TIGD5** | 1.07168 | 3.424903 | 10.02816 | 2.35E-21 | 4.04E-20 | 37.6476605 |
| **DIAPH1** | 1.240611 | 3.906795 | 10.02715 | 2.37E-21 | 4.07E-20 | 37.63947392 |
| **METTL18** | 0.776026 | 2.455843 | 10.02634 | 2.38E-21 | 4.09E-20 | 37.63299129 |
| **PEX13** | 0.943648 | 2.592332 | 10.02606 | 2.39E-21 | 4.10E-20 | 37.6307092 |
| **LSM12** | 1.199471 | 3.718379 | 10.025 | 2.41E-21 | 4.13E-20 | 37.62217372 |
| **USP42** | 0.897146 | 2.131571 | 10.02165 | 2.48E-21 | 4.24E-20 | 37.59514426 |
| **SETD8** | 1.049477 | 3.566099 | 10.02035 | 2.50E-21 | 4.28E-20 | 37.58469955 |
| **KAT7** | 1.412124 | 3.847708 | 10.01984 | 2.51E-21 | 4.30E-20 | 37.58059893 |
| **GTPBP4** | 1.152022 | 3.504542 | 10.01923 | 2.53E-21 | 4.32E-20 | 37.57569895 |
| **PATL1** | 1.15531 | 2.542302 | 10.01831 | 2.55E-21 | 4.34E-20 | 37.56829896 |
| **AC005562.1** | 0.625245 | 1.584571 | 10.0181 | 2.55E-21 | 4.35E-20 | 37.56659366 |
| **SS18** | 1.409708 | 3.475926 | 10.0175 | 2.56E-21 | 4.37E-20 | 37.56176776 |
| **RBBP4** | 1.787974 | 4.411115 | 10.01737 | 2.57E-21 | 4.37E-20 | 37.56073118 |
| **ATF1** | 0.958356 | 2.29276 | 10.01719 | 2.57E-21 | 4.37E-20 | 37.55922765 |
| **TUBG1** | 1.24757 | 4.106634 | 10.01716 | 2.57E-21 | 4.37E-20 | 37.55899116 |
| **PARP9** | 1.202651 | 2.903826 | 10.01614 | 2.59E-21 | 4.40E-20 | 37.55077326 |
| **WARS** | 1.450625 | 4.801788 | 10.01466 | 2.62E-21 | 4.45E-20 | 37.53885346 |
| **RBM8A** | 1.399516 | 5.593382 | 10.01416 | 2.63E-21 | 4.47E-20 | 37.53483624 |
| **LXN** | 0.973547 | 1.716307 | 10.01313 | 2.66E-21 | 4.50E-20 | 37.52651154 |
| **H2AFV** | 1.628072 | 6.295982 | 10.01132 | 2.70E-21 | 4.57E-20 | 37.51199311 |
| **RP1-228H13.5** | 0.582703 | 1.096615 | 10.01064 | 2.71E-21 | 4.59E-20 | 37.50645025 |
| **YTHDF3** | 1.073558 | 3.128449 | 10.00934 | 2.74E-21 | 4.64E-20 | 37.49600594 |
| **IKBKE** | 0.691184 | 1.644698 | 10.00877 | 2.75E-21 | 4.65E-20 | 37.49145637 |
| **CEP78** | 1.05006 | 2.760212 | 10.00838 | 2.76E-21 | 4.67E-20 | 37.48826317 |
| **HAUS5** | 1.038931 | 3.196551 | 10.00805 | 2.77E-21 | 4.67E-20 | 37.48559585 |
| **ZNF845** | 0.580984 | 0.89567 | 10.00647 | 2.80E-21 | 4.73E-20 | 37.47288143 |
| **HAUS6** | 0.903535 | 1.996081 | 10.00612 | 2.81E-21 | 4.74E-20 | 37.47011159 |
| **ERLIN1** | 1.105776 | 2.22854 | 10.00462 | 2.85E-21 | 4.80E-20 | 37.45803708 |
| **SPPL3** | 1.111237 | 4.179678 | 10.00297 | 2.89E-21 | 4.86E-20 | 37.44476646 |
| **FAM91A1** | 1.139647 | 2.641073 | 10.00152 | 2.92E-21 | 4.91E-20 | 37.43305878 |
| **LOXL3** | 1.033198 | 2.867846 | 10.00105 | 2.93E-21 | 4.93E-20 | 37.42925253 |
| **SDCCAG3** | 1.259693 | 4.57276 | 10.00093 | 2.93E-21 | 4.93E-20 | 37.42829959 |
| **MPPE1** | 1.170799 | 3.829642 | 10.00028 | 2.95E-21 | 4.95E-20 | 37.4230956 |
| **MKRN3** | 1.073183 | 2.153673 | 9.99902 | 2.98E-21 | 5.00E-20 | 37.41294576 |
| **ANO6** | 1.146517 | 2.381579 | 9.998644 | 2.99E-21 | 5.01E-20 | 37.40992493 |
| **PTAR1** | 1.041238 | 2.408355 | 9.998288 | 3.00E-21 | 5.02E-20 | 37.40705945 |
| **POT1** | 1.075799 | 2.896075 | 9.997964 | 3.01E-21 | 5.03E-20 | 37.40445387 |
| **TBRG4** | 1.142448 | 4.870921 | 9.996767 | 3.03E-21 | 5.07E-20 | 37.39481828 |
| **ERCC8** | 0.7777 | 2.126863 | 9.996481 | 3.04E-21 | 5.08E-20 | 37.39251425 |
| **SARM1** | 1.104171 | 3.011348 | 9.996119 | 3.05E-21 | 5.09E-20 | 37.3896022 |
| **CDC42EP3** | 0.880588 | 1.502584 | 9.995655 | 3.06E-21 | 5.11E-20 | 37.38587121 |
| **SH3KBP1** | 1.134167 | 3.369896 | 9.994999 | 3.08E-21 | 5.13E-20 | 37.38059375 |
| **GMEB2** | 0.768238 | 2.57746 | 9.994898 | 3.08E-21 | 5.13E-20 | 37.37978276 |
| **RNGTT** | 0.736719 | 1.987404 | 9.994877 | 3.08E-21 | 5.13E-20 | 37.37961179 |
| **ZNF557** | 0.572621 | 1.070741 | 9.994732 | 3.09E-21 | 5.13E-20 | 37.37844923 |
| **LRRC37BP1** | 0.916759 | 2.673611 | 9.993405 | 3.12E-21 | 5.19E-20 | 37.36777506 |
| **CAV1** | 1.273738 | 2.614093 | 9.992538 | 3.14E-21 | 5.22E-20 | 37.36079836 |
| **STYX** | 0.633105 | 1.596548 | 9.989706 | 3.21E-21 | 5.34E-20 | 37.33801986 |
| **INO80** | 0.894645 | 2.398735 | 9.989541 | 3.22E-21 | 5.34E-20 | 37.33669667 |
| **BPNT1** | 1.03198 | 3.413259 | 9.988652 | 3.24E-21 | 5.38E-20 | 37.32954514 |
| **ANTXR1** | 1.402803 | 4.043732 | 9.987971 | 3.26E-21 | 5.40E-20 | 37.32406907 |
| **CUL4A** | 1.2335 | 3.794473 | 9.987143 | 3.28E-21 | 5.44E-20 | 37.31740684 |
| **NIPA2** | 1.275883 | 3.518304 | 9.986327 | 3.30E-21 | 5.47E-20 | 37.31084367 |
| **METTL2B** | 1.003334 | 3.303584 | 9.985838 | 3.32E-21 | 5.49E-20 | 37.30691651 |
| **CCNK** | 1.163194 | 3.510684 | 9.98467 | 3.35E-21 | 5.53E-20 | 37.2975274 |
| **RP11-500C11.3** | 0.960416 | 2.126507 | 9.984183 | 3.36E-21 | 5.55E-20 | 37.29360921 |
| **TTC26** | 0.694313 | 1.129305 | 9.984003 | 3.37E-21 | 5.56E-20 | 37.29216089 |
| **ZNF778** | 0.880424 | 1.775348 | 9.983943 | 3.37E-21 | 5.56E-20 | 37.29168252 |
| **ZNF217** | 0.850983 | 1.312725 | 9.983103 | 3.39E-21 | 5.59E-20 | 37.284923 |
| **MAGOHB** | 1.154322 | 3.762568 | 9.982634 | 3.41E-21 | 5.61E-20 | 37.28115219 |
| **LEPREL4** | 0.960021 | 3.490643 | 9.981346 | 3.44E-21 | 5.66E-20 | 37.27080019 |
| **RBM7** | 1.104057 | 2.85497 | 9.981261 | 3.44E-21 | 5.66E-20 | 37.27011955 |
| **GLB1** | 1.037966 | 3.244986 | 9.980886 | 3.45E-21 | 5.68E-20 | 37.26710335 |
| **SP1** | 1.04716 | 2.437981 | 9.980154 | 3.47E-21 | 5.71E-20 | 37.26122127 |
| **PUS3** | 0.877439 | 2.208946 | 9.980104 | 3.48E-21 | 5.71E-20 | 37.26081886 |
| **POLR2A** | 1.189686 | 4.027362 | 9.979781 | 3.49E-21 | 5.72E-20 | 37.25822054 |
| **ZFP82** | 0.719931 | 1.975474 | 9.979684 | 3.49E-21 | 5.72E-20 | 37.25744463 |
| **SIN3A** | 1.095078 | 2.814728 | 9.978913 | 3.51E-21 | 5.75E-20 | 37.25124787 |
| **SOWAHC** | 0.843095 | 1.647044 | 9.978829 | 3.51E-21 | 5.75E-20 | 37.2505715 |
| **ZNF891** | 0.530774 | 0.924688 | 9.976566 | 3.58E-21 | 5.85E-20 | 37.23238257 |
| **MYO1B** | 1.11341 | 2.340693 | 9.97505 | 3.62E-21 | 5.92E-20 | 37.22020388 |
| **ZNF568** | 0.927516 | 2.157405 | 9.973893 | 3.66E-21 | 5.97E-20 | 37.2109103 |
| **ERGIC1** | 1.401723 | 4.579965 | 9.973186 | 3.68E-21 | 6.00E-20 | 37.20522808 |
| **CTD-2267D19.3** | 0.604044 | 0.802579 | 9.973157 | 3.68E-21 | 6.00E-20 | 37.20499496 |
| **CSTF2** | 0.992715 | 2.737823 | 9.973078 | 3.68E-21 | 6.00E-20 | 37.20436142 |
| **TMEM229B** | 1.205593 | 3.539927 | 9.9718 | 3.72E-21 | 6.05E-20 | 37.19409216 |
| **MAPK14** | 1.060883 | 3.016415 | 9.971516 | 3.73E-21 | 6.06E-20 | 37.19181298 |
| **SH3BP4** | 1.091927 | 3.055463 | 9.971335 | 3.73E-21 | 6.07E-20 | 37.1903558 |
| **RNASEH1** | 1.041116 | 2.384849 | 9.9707 | 3.75E-21 | 6.10E-20 | 37.18525752 |
| **SNAPC1** | 0.66983 | 1.711048 | 9.969489 | 3.79E-21 | 6.15E-20 | 37.17553099 |
| **C12orf5** | 1.183737 | 2.726129 | 9.969382 | 3.79E-21 | 6.15E-20 | 37.17467042 |
| **RP11-115C21.2** | 0.918515 | 2.698482 | 9.96875 | 3.81E-21 | 6.18E-20 | 37.16959534 |
| **ZNF320** | 0.971723 | 2.69403 | 9.968525 | 3.82E-21 | 6.19E-20 | 37.16778071 |
| **ZNF121** | 0.994932 | 3.115202 | 9.967769 | 3.84E-21 | 6.22E-20 | 37.16171121 |
| **TSPAN9** | 0.850399 | 2.765019 | 9.967517 | 3.85E-21 | 6.23E-20 | 37.15969081 |
| **CTNS** | 0.958155 | 3.088883 | 9.967331 | 3.86E-21 | 6.23E-20 | 37.15819761 |
| **SGPL1** | 1.093427 | 3.125085 | 9.967278 | 3.86E-21 | 6.23E-20 | 37.15777096 |
| **CTHRC1** | 1.262564 | 1.701083 | 9.967128 | 3.86E-21 | 6.24E-20 | 37.1565629 |
| **IGF2R** | 1.115227 | 2.529101 | 9.96637 | 3.89E-21 | 6.27E-20 | 37.15047921 |
| **ZNF362** | 1.310201 | 3.304114 | 9.965597 | 3.91E-21 | 6.31E-20 | 37.14426542 |
| **TIPRL** | 1.249424 | 3.061376 | 9.964225 | 3.96E-21 | 6.37E-20 | 37.13325148 |
| **KCTD20** | 1.279755 | 3.543621 | 9.96384 | 3.97E-21 | 6.39E-20 | 37.13015688 |
| **TFAM** | 1.205345 | 2.614576 | 9.963594 | 3.98E-21 | 6.40E-20 | 37.12818749 |
| **RPS2P46** | 1.539871 | 3.960335 | 9.962833 | 4.00E-21 | 6.43E-20 | 37.12207353 |
| **TTPAL** | 0.775938 | 2.100021 | 9.962388 | 4.02E-21 | 6.45E-20 | 37.11850125 |
| **ZNF440** | 0.769129 | 1.577422 | 9.961093 | 4.06E-21 | 6.51E-20 | 37.10810118 |
| **MFSD1** | 1.309468 | 3.900563 | 9.960026 | 4.09E-21 | 6.56E-20 | 37.09953896 |
| **GRAP** | 0.727155 | 1.847588 | 9.960022 | 4.09E-21 | 6.56E-20 | 37.09950529 |
| **DNMT3A** | 0.956139 | 2.423597 | 9.959432 | 4.11E-21 | 6.59E-20 | 37.09477176 |
| **FAM109A** | 0.917107 | 2.954258 | 9.959219 | 4.12E-21 | 6.59E-20 | 37.09306412 |
| **BLOC1S4** | 1.052184 | 3.576546 | 9.959166 | 4.12E-21 | 6.59E-20 | 37.09263791 |
| **POM121** | 1.27744 | 3.089129 | 9.957222 | 4.19E-21 | 6.69E-20 | 37.07702767 |
| **INTS8** | 1.082654 | 3.173849 | 9.956445 | 4.21E-21 | 6.73E-20 | 37.07079389 |
| **NUP153** | 0.970566 | 2.412218 | 9.956011 | 4.23E-21 | 6.75E-20 | 37.06731339 |
| **ENTPD1** | 0.911151 | 2.809956 | 9.95444 | 4.28E-21 | 6.83E-20 | 37.05470324 |
| **LETM1** | 0.984247 | 3.513207 | 9.954162 | 4.29E-21 | 6.84E-20 | 37.05247379 |
| **PCBP1-AS1** | 1.031919 | 3.661964 | 9.954122 | 4.29E-21 | 6.84E-20 | 37.05215503 |
| **RP11-473I1.9** | 0.863739 | 1.947216 | 9.954102 | 4.30E-21 | 6.84E-20 | 37.05199261 |
| **NUAK2** | 0.52955 | 0.757203 | 9.95328 | 4.32E-21 | 6.88E-20 | 37.04539289 |
| **VWA9** | 1.119835 | 3.499947 | 9.952227 | 4.36E-21 | 6.93E-20 | 37.03694658 |
| **ELL** | 0.810019 | 2.335089 | 9.951835 | 4.37E-21 | 6.95E-20 | 37.0338024 |
| **LSM14A** | 1.558363 | 4.574484 | 9.951797 | 4.38E-21 | 6.95E-20 | 37.0334955 |
| **PAFAH2** | 0.740762 | 1.843244 | 9.951466 | 4.39E-21 | 6.96E-20 | 37.03084176 |
| **MEN1** | 1.103468 | 4.010669 | 9.950102 | 4.44E-21 | 7.04E-20 | 37.01989507 |
| **MORC4** | 0.788795 | 1.792442 | 9.94844 | 4.50E-21 | 7.13E-20 | 37.00656623 |
| **MAPRE1** | 1.571019 | 5.202914 | 9.948396 | 4.50E-21 | 7.13E-20 | 37.00621391 |
| **COPZ1** | 1.407497 | 5.778328 | 9.947644 | 4.53E-21 | 7.16E-20 | 37.00017611 |
| **FAM86DP** | 0.819347 | 2.58875 | 9.946683 | 4.56E-21 | 7.22E-20 | 36.9924653 |
| **UHRF1** | 1.263966 | 2.769047 | 9.946467 | 4.57E-21 | 7.22E-20 | 36.9907344 |
| **MNS1** | 0.853571 | 1.741969 | 9.945684 | 4.60E-21 | 7.26E-20 | 36.9844547 |
| **ANKLE2** | 1.088975 | 3.835094 | 9.945667 | 4.60E-21 | 7.26E-20 | 36.98432352 |
| **ZFP37** | 0.791564 | 1.466279 | 9.945527 | 4.61E-21 | 7.26E-20 | 36.98320083 |
| **DOLPP1** | 1.082389 | 3.474621 | 9.945084 | 4.62E-21 | 7.29E-20 | 36.97964774 |
| **DSCR3** | 1.128541 | 3.615657 | 9.94388 | 4.67E-21 | 7.35E-20 | 36.96998764 |
| **KLHDC8A** | 1.524581 | 3.788409 | 9.94355 | 4.68E-21 | 7.36E-20 | 36.96733781 |
| **SMEK1** | 1.262804 | 3.895031 | 9.943043 | 4.70E-21 | 7.38E-20 | 36.96327773 |
| **PAPD7** | 0.973446 | 3.124734 | 9.942412 | 4.72E-21 | 7.42E-20 | 36.95821352 |
| **GTPBP8** | 1.02991 | 2.463581 | 9.941625 | 4.75E-21 | 7.46E-20 | 36.95190269 |
| **TBX15** | 0.669465 | 0.958534 | 9.940704 | 4.79E-21 | 7.51E-20 | 36.94451644 |
| **SEPHS1** | 1.487451 | 4.834449 | 9.937535 | 4.91E-21 | 7.70E-20 | 36.91910923 |
| **ZNF346** | 0.867394 | 2.973688 | 9.93714 | 4.93E-21 | 7.72E-20 | 36.91594427 |
| **THRAP3** | 1.195559 | 4.177259 | 9.936839 | 4.94E-21 | 7.73E-20 | 36.91353591 |
| **ADAM19** | 0.754987 | 1.300594 | 9.936206 | 4.97E-21 | 7.76E-20 | 36.90846063 |
| **NFATC2IP** | 1.183905 | 3.116485 | 9.936003 | 4.98E-21 | 7.77E-20 | 36.90683305 |
| **LMAN2L** | 1.013357 | 3.327706 | 9.935693 | 4.99E-21 | 7.79E-20 | 36.90435071 |
| **MCPH1** | 0.798932 | 2.248026 | 9.935636 | 4.99E-21 | 7.79E-20 | 36.90388785 |
| **H2AFY** | 1.544062 | 6.122438 | 9.935326 | 5.00E-21 | 7.80E-20 | 36.90140476 |
| **LNX2** | 0.512284 | 0.937357 | 9.935152 | 5.01E-21 | 7.81E-20 | 36.90000743 |
| **MCM9** | 0.764582 | 1.556519 | 9.934122 | 5.05E-21 | 7.87E-20 | 36.89175388 |
| **PHF8** | 1.030033 | 2.438594 | 9.933744 | 5.07E-21 | 7.89E-20 | 36.88872314 |
| **TIGD2** | 0.591391 | 1.317316 | 9.933184 | 5.09E-21 | 7.92E-20 | 36.88423791 |
| **RDH10** | 1.149368 | 2.058211 | 9.932508 | 5.12E-21 | 7.95E-20 | 36.87881778 |
| **LARP4B** | 1.017064 | 3.187493 | 9.932341 | 5.13E-21 | 7.96E-20 | 36.87748146 |
| **KIAA1586** | 0.888478 | 1.879152 | 9.931888 | 5.14E-21 | 7.98E-20 | 36.8738543 |
| **PRKD3** | 1.076298 | 2.456756 | 9.930866 | 5.19E-21 | 8.05E-20 | 36.86565824 |
| **DZIP1L** | 0.802142 | 1.555669 | 9.930362 | 5.21E-21 | 8.07E-20 | 36.86161897 |
| **TMED5** | 1.259445 | 3.276507 | 9.930051 | 5.22E-21 | 8.09E-20 | 36.8591274 |
| **ZNF229** | 0.885073 | 1.499938 | 9.928516 | 5.29E-21 | 8.18E-20 | 36.84683324 |
| **MINPP1** | 0.905059 | 2.649249 | 9.927024 | 5.35E-21 | 8.28E-20 | 36.83487529 |
| **CIRH1A** | 1.085324 | 3.649577 | 9.926775 | 5.36E-21 | 8.29E-20 | 36.83288756 |
| **S1PR2** | 0.805788 | 1.074288 | 9.926363 | 5.38E-21 | 8.31E-20 | 36.82957911 |
| **RC3H2** | 0.897459 | 2.962158 | 9.925624 | 5.41E-21 | 8.36E-20 | 36.82366318 |
| **CDH11** | 1.50018 | 2.895766 | 9.925068 | 5.44E-21 | 8.39E-20 | 36.81921226 |
| **ZNF345** | 0.65806 | 1.698188 | 9.924858 | 5.45E-21 | 8.40E-20 | 36.8175248 |
| **PA2G4** | 1.390662 | 5.282222 | 9.924712 | 5.45E-21 | 8.40E-20 | 36.81636013 |
| **TMEM159** | 0.907076 | 1.926002 | 9.924585 | 5.46E-21 | 8.41E-20 | 36.81533846 |
| **RAD23B** | 1.353919 | 4.513977 | 9.924107 | 5.48E-21 | 8.43E-20 | 36.81151012 |
| **EXOSC6** | 0.946982 | 3.36188 | 9.923607 | 5.50E-21 | 8.46E-20 | 36.80750443 |
| **FAM136A** | 1.055881 | 3.811215 | 9.923401 | 5.51E-21 | 8.47E-20 | 36.80585422 |
| **IFT81** | 1.1022 | 2.595785 | 9.923279 | 5.52E-21 | 8.47E-20 | 36.80487925 |
| **DHX35** | 0.841972 | 2.439194 | 9.921576 | 5.59E-21 | 8.59E-20 | 36.79124056 |
| **LRRC57** | 0.845295 | 2.247671 | 9.92123 | 5.61E-21 | 8.61E-20 | 36.7884713 |
| **PARP4** | 0.957427 | 2.47498 | 9.920685 | 5.63E-21 | 8.64E-20 | 36.78410563 |
| **HYAL2** | 1.206088 | 4.038029 | 9.920623 | 5.64E-21 | 8.64E-20 | 36.78361136 |
| **ZMPSTE24** | 1.167346 | 3.326175 | 9.920416 | 5.65E-21 | 8.65E-20 | 36.78195384 |
| **ZNF462** | 1.015282 | 2.379368 | 9.919888 | 5.67E-21 | 8.68E-20 | 36.77772447 |
| **CCDC174** | 0.954814 | 2.792106 | 9.919557 | 5.69E-21 | 8.69E-20 | 36.77507002 |
| **HNRNPA0** | 1.582175 | 5.537514 | 9.91953 | 5.69E-21 | 8.69E-20 | 36.77485348 |
| **RUNX1** | 0.943939 | 1.570228 | 9.919344 | 5.70E-21 | 8.70E-20 | 36.77336677 |
| **TTC31** | 1.04258 | 3.146102 | 9.918982 | 5.71E-21 | 8.71E-20 | 36.77046889 |
| **KLRG1** | 0.715248 | 1.601203 | 9.91896 | 5.71E-21 | 8.71E-20 | 36.77029318 |
| **PTPN1** | 1.111501 | 2.993814 | 9.918915 | 5.72E-21 | 8.71E-20 | 36.76993165 |
| **DUSP16** | 1.091218 | 1.974542 | 9.918795 | 5.72E-21 | 8.71E-20 | 36.76897185 |
| **KCTD10** | 1.056367 | 3.190536 | 9.918751 | 5.72E-21 | 8.71E-20 | 36.76862111 |
| **PTP4A3** | 1.386918 | 4.911076 | 9.918024 | 5.76E-21 | 8.76E-20 | 36.76279876 |
| **SH3PXD2B** | 0.992917 | 2.684508 | 9.917776 | 5.77E-21 | 8.77E-20 | 36.76080848 |
| **NAA15** | 0.937887 | 2.187708 | 9.917485 | 5.78E-21 | 8.79E-20 | 36.75848465 |
| **THAP1** | 0.698089 | 1.970871 | 9.917346 | 5.79E-21 | 8.79E-20 | 36.75736669 |
| **UBE2I** | 1.367398 | 5.363556 | 9.915655 | 5.87E-21 | 8.91E-20 | 36.74382888 |
| **MOCS3** | 0.734311 | 1.970714 | 9.915279 | 5.89E-21 | 8.93E-20 | 36.74081576 |
| **STT3B** | 1.185187 | 3.756344 | 9.915183 | 5.89E-21 | 8.93E-20 | 36.74005494 |
| **FKBP15** | 1.056147 | 2.916996 | 9.913212 | 5.99E-21 | 9.07E-20 | 36.72427422 |
| **AGO1** | 0.766722 | 2.286872 | 9.911901 | 6.05E-21 | 9.15E-20 | 36.71377739 |
| **LHFPL2** | 1.080309 | 2.623196 | 9.909724 | 6.16E-21 | 9.31E-20 | 36.69635997 |
| **FYTTD1** | 1.346074 | 3.618866 | 9.909475 | 6.17E-21 | 9.32E-20 | 36.69436713 |
| **PDCD11** | 0.839712 | 3.050794 | 9.907903 | 6.25E-21 | 9.44E-20 | 36.68178106 |
| **YIPF4** | 1.050778 | 3.308279 | 9.906782 | 6.31E-21 | 9.52E-20 | 36.67281622 |
| **NUP54** | 0.918009 | 3.295667 | 9.905477 | 6.37E-21 | 9.61E-20 | 36.66237423 |
| **RBBP9** | 0.971348 | 2.531058 | 9.904832 | 6.41E-21 | 9.66E-20 | 36.65721356 |
| **ZSCAN2** | 0.73228 | 2.413117 | 9.901569 | 6.58E-21 | 9.91E-20 | 36.6311099 |
| **MOB1A** | 1.163386 | 3.263811 | 9.900159 | 6.65E-21 | 1.00E-19 | 36.61983245 |
| **RP11-815I9.4** | 0.68165 | 1.119385 | 9.900051 | 6.66E-21 | 1.00E-19 | 36.6189699 |
| **ZNF678** | 0.692072 | 1.444541 | 9.898957 | 6.72E-21 | 1.01E-19 | 36.61021831 |
| **CTNND1** | 1.420252 | 4.14478 | 9.89862 | 6.74E-21 | 1.01E-19 | 36.60752896 |
| **ELF2** | 1.037216 | 2.87686 | 9.896516 | 6.85E-21 | 1.03E-19 | 36.59070021 |
| **CASC15** | 1.031888 | 2.224035 | 9.896054 | 6.88E-21 | 1.03E-19 | 36.58700373 |
| **SLC25A24** | 0.738148 | 1.169628 | 9.895633 | 6.90E-21 | 1.04E-19 | 36.58364378 |
| **BBS9** | 0.671923 | 2.207373 | 9.895137 | 6.93E-21 | 1.04E-19 | 36.57967328 |
| **BRD4** | 1.216692 | 3.848508 | 9.894887 | 6.94E-21 | 1.04E-19 | 36.57767596 |
| **WDR41** | 1.151788 | 3.53711 | 9.894553 | 6.96E-21 | 1.04E-19 | 36.57500208 |
| **IREB2** | 1.049783 | 2.489433 | 9.893272 | 7.04E-21 | 1.05E-19 | 36.56476802 |
| **COQ7** | 1.059626 | 3.692041 | 9.892705 | 7.07E-21 | 1.06E-19 | 36.56023084 |
| **ZNF718** | 0.913313 | 1.706455 | 9.891808 | 7.12E-21 | 1.06E-19 | 36.55306127 |
| **KHSRP** | 1.677807 | 6.356757 | 9.891765 | 7.12E-21 | 1.06E-19 | 36.5527153 |
| **CDH5** | 1.028489 | 2.098607 | 9.889702 | 7.24E-21 | 1.08E-19 | 36.53622452 |
| **NAA50** | 1.400362 | 3.782454 | 9.888323 | 7.32E-21 | 1.09E-19 | 36.52520544 |
| **TMEM68** | 0.932909 | 2.744898 | 9.88815 | 7.33E-21 | 1.09E-19 | 36.52382461 |
| **GCNT2** | 0.735877 | 1.489532 | 9.888028 | 7.34E-21 | 1.09E-19 | 36.52284613 |
| **S100PBP** | 0.984614 | 2.899876 | 9.887896 | 7.35E-21 | 1.09E-19 | 36.52179432 |
| **PPP1R10** | 1.251663 | 4.400278 | 9.887799 | 7.36E-21 | 1.09E-19 | 36.52101509 |
| **C18orf21** | 1.03176 | 3.534673 | 9.884709 | 7.54E-21 | 1.12E-19 | 36.4963281 |
| **ZNRF1** | 0.983465 | 3.539391 | 9.884666 | 7.54E-21 | 1.12E-19 | 36.49598435 |
| **ALG2** | 1.097444 | 2.976776 | 9.884312 | 7.57E-21 | 1.12E-19 | 36.49315735 |
| **FAM129A** | 0.813262 | 1.028111 | 9.883824 | 7.60E-21 | 1.13E-19 | 36.48925119 |
| **SMNDC1** | 0.960218 | 2.29705 | 9.882341 | 7.69E-21 | 1.14E-19 | 36.47740312 |
| **UNG** | 1.24514 | 3.513111 | 9.879802 | 7.85E-21 | 1.16E-19 | 36.4571227 |
| **DONSON** | 1.03136 | 3.057739 | 9.879516 | 7.87E-21 | 1.16E-19 | 36.45483896 |
| **MIS12** | 0.986569 | 2.769034 | 9.879208 | 7.88E-21 | 1.17E-19 | 36.45237656 |
| **ARL2BP** | 1.454028 | 4.525742 | 9.878552 | 7.93E-21 | 1.17E-19 | 36.44713692 |
| **GTPBP3** | 1.1101 | 3.749132 | 9.877971 | 7.96E-21 | 1.18E-19 | 36.44250152 |
| **CPT2** | 0.870337 | 2.548899 | 9.877895 | 7.97E-21 | 1.18E-19 | 36.44189138 |
| **CTSC** | 1.379463 | 3.847065 | 9.875584 | 8.12E-21 | 1.20E-19 | 36.42343883 |
| **ZNF620** | 0.610796 | 1.336731 | 9.874527 | 8.19E-21 | 1.21E-19 | 36.41499778 |
| **MGAT2** | 1.186524 | 2.541449 | 9.874056 | 8.22E-21 | 1.21E-19 | 36.41123587 |
| **FAM120A** | 1.094128 | 3.794345 | 9.873978 | 8.23E-21 | 1.21E-19 | 36.41061476 |
| **PAXBP1** | 1.342194 | 3.729729 | 9.873939 | 8.23E-21 | 1.21E-19 | 36.4103024 |
| **BUB3** | 1.422123 | 4.655007 | 9.873331 | 8.27E-21 | 1.22E-19 | 36.40544405 |
| **PLAUR** | 1.142653 | 2.383759 | 9.873014 | 8.29E-21 | 1.22E-19 | 36.40291792 |
| **PPP1R15B** | 1.110723 | 2.762485 | 9.872087 | 8.35E-21 | 1.23E-19 | 36.39551066 |
| **PXN** | 1.344688 | 4.544225 | 9.871796 | 8.37E-21 | 1.23E-19 | 36.39318635 |
| **MOB1B** | 0.906143 | 2.323072 | 9.871708 | 8.38E-21 | 1.23E-19 | 36.39248608 |
| **PM20D2** | 0.998755 | 2.1475 | 9.870781 | 8.44E-21 | 1.24E-19 | 36.38508579 |
| **FAIM3** | 0.716808 | 1.066631 | 9.869078 | 8.56E-21 | 1.26E-19 | 36.371492 |
| **MDC1** | 1.044506 | 3.345418 | 9.868247 | 8.62E-21 | 1.26E-19 | 36.3648593 |
| **IFI16** | 1.506418 | 4.390276 | 9.86703 | 8.70E-21 | 1.27E-19 | 36.35514363 |
| **CNTROB** | 1.039435 | 3.990105 | 9.866319 | 8.75E-21 | 1.28E-19 | 36.34946976 |
| **TOR1A** | 1.148492 | 3.846373 | 9.866159 | 8.76E-21 | 1.28E-19 | 36.34819205 |
| **UCP2** | 1.476088 | 4.116079 | 9.865662 | 8.80E-21 | 1.29E-19 | 36.34422727 |
| **MSH2** | 1.089485 | 2.704333 | 9.864291 | 8.90E-21 | 1.30E-19 | 36.33328673 |
| **ZNF628** | 0.838606 | 2.547578 | 9.861628 | 9.09E-21 | 1.33E-19 | 36.31203943 |
| **VPS54** | 0.88993 | 2.589262 | 9.860774 | 9.15E-21 | 1.34E-19 | 36.30522514 |
| **KDR** | 0.943898 | 1.577378 | 9.860707 | 9.16E-21 | 1.34E-19 | 36.30469063 |
| **SPAST** | 0.958097 | 2.684786 | 9.858339 | 9.33E-21 | 1.36E-19 | 36.28579951 |
| **C12orf49** | 1.12996 | 3.182247 | 9.858339 | 9.33E-21 | 1.36E-19 | 36.28579529 |
| **LRRC41** | 1.18369 | 3.968727 | 9.857935 | 9.36E-21 | 1.36E-19 | 36.28257274 |
| **EPB41L4A-AS2** | 0.740541 | 1.359041 | 9.857826 | 9.37E-21 | 1.36E-19 | 36.28170467 |
| **SLC35B2** | 1.249935 | 4.63598 | 9.857193 | 9.42E-21 | 1.37E-19 | 36.27666109 |
| **SLC25A17** | 0.975669 | 3.17096 | 9.85595 | 9.52E-21 | 1.38E-19 | 36.26674129 |
| **PLCE1** | 0.877979 | 1.777066 | 9.855569 | 9.55E-21 | 1.39E-19 | 36.26370838 |
| **ZNF235** | 0.646872 | 1.270064 | 9.853983 | 9.67E-21 | 1.40E-19 | 36.25105955 |
| **DOLK** | 1.001321 | 3.431569 | 9.851918 | 9.83E-21 | 1.43E-19 | 36.23459224 |
| **RBM3** | 1.563037 | 5.777111 | 9.851757 | 9.84E-21 | 1.43E-19 | 36.2333042 |
| **USP24** | 1.073226 | 2.663453 | 9.851146 | 9.89E-21 | 1.43E-19 | 36.22843305 |
| **TCF7L2** | 0.971689 | 2.882722 | 9.851101 | 9.90E-21 | 1.43E-19 | 36.22807764 |
| **GUSBP1** | 0.874491 | 2.637602 | 9.851055 | 9.90E-21 | 1.43E-19 | 36.22770818 |
| **METAP1** | 1.074054 | 2.995777 | 9.84823 | 1.01E-20 | 1.46E-19 | 36.20518195 |
| **TMCO1** | 1.508402 | 4.980707 | 9.845695 | 1.03E-20 | 1.49E-19 | 36.18497561 |
| **GTF3C3** | 1.037812 | 2.750151 | 9.845484 | 1.04E-20 | 1.49E-19 | 36.18329348 |
| **RCOR1** | 0.806436 | 1.944138 | 9.845135 | 1.04E-20 | 1.50E-19 | 36.18051125 |
| **PTBP3** | 0.803806 | 1.730455 | 9.84499 | 1.04E-20 | 1.50E-19 | 36.17936116 |
| **TMED2** | 1.56802 | 4.671741 | 9.843453 | 1.05E-20 | 1.52E-19 | 36.16710798 |
| **ARFGAP3** | 1.087705 | 3.479414 | 9.842349 | 1.06E-20 | 1.53E-19 | 36.15830956 |
| **ALKBH8** | 0.793037 | 1.657716 | 9.841639 | 1.07E-20 | 1.54E-19 | 36.15265707 |
| **IPO8** | 1.023464 | 3.089279 | 9.84119 | 1.07E-20 | 1.54E-19 | 36.14907522 |
| **IL1RAP** | 1.111691 | 1.693469 | 9.841087 | 1.07E-20 | 1.54E-19 | 36.14825673 |
| **SAP130** | 0.994117 | 2.65907 | 9.841028 | 1.07E-20 | 1.54E-19 | 36.14778328 |
| **NSL1** | 1.401498 | 4.150452 | 9.840449 | 1.08E-20 | 1.55E-19 | 36.14316788 |
| **ZNF254** | 0.984859 | 2.196528 | 9.840289 | 1.08E-20 | 1.55E-19 | 36.14189818 |
| **ZNF629** | 1.046993 | 1.81632 | 9.839756 | 1.08E-20 | 1.55E-19 | 36.13765315 |
| **GRAMD1A** | 1.263902 | 4.823293 | 9.839695 | 1.09E-20 | 1.55E-19 | 36.13716529 |
| **PCMTD2** | 1.549066 | 4.417796 | 9.839507 | 1.09E-20 | 1.56E-19 | 36.13566775 |
| **CLPB** | 0.815152 | 3.356844 | 9.83912 | 1.09E-20 | 1.56E-19 | 36.13258435 |
| **LINC00467** | 0.878767 | 2.68888 | 9.839101 | 1.09E-20 | 1.56E-19 | 36.13242748 |
| **SPATS2** | 0.982789 | 2.964747 | 9.838579 | 1.09E-20 | 1.56E-19 | 36.12826999 |
| **C1orf106** | 1.117522 | 2.65614 | 9.836261 | 1.12E-20 | 1.59E-19 | 36.10980246 |
| **MSL2** | 0.973268 | 2.41957 | 9.835576 | 1.12E-20 | 1.60E-19 | 36.10434671 |
| **C3orf17** | 1.156024 | 3.350435 | 9.834597 | 1.13E-20 | 1.61E-19 | 36.09655044 |
| **PTDSS1** | 1.278641 | 4.468402 | 9.834425 | 1.13E-20 | 1.61E-19 | 36.09518485 |
| **FAM76B** | 1.156745 | 2.634306 | 9.834185 | 1.13E-20 | 1.62E-19 | 36.09326707 |
| **TTI1** | 1.013664 | 2.860369 | 9.83398 | 1.14E-20 | 1.62E-19 | 36.09164083 |
| **GTF2H2C** | 1.209773 | 2.786909 | 9.833776 | 1.14E-20 | 1.62E-19 | 36.09001185 |
| **TNPO1** | 1.311372 | 3.360718 | 9.832536 | 1.15E-20 | 1.63E-19 | 36.08013799 |
| **VHL** | 1.222764 | 3.940807 | 9.832168 | 1.15E-20 | 1.64E-19 | 36.07720421 |
| **ABT1** | 0.978469 | 3.172263 | 9.83209 | 1.15E-20 | 1.64E-19 | 36.07658188 |
| **DCHS1** | 1.060118 | 2.465869 | 9.831863 | 1.16E-20 | 1.64E-19 | 36.07477818 |
| **SKIL** | 1.022198 | 2.615725 | 9.831612 | 1.16E-20 | 1.64E-19 | 36.0727815 |
| **DUSP6** | 1.421748 | 3.022549 | 9.831373 | 1.16E-20 | 1.64E-19 | 36.07087797 |
| **SLC20A1** | 1.066397 | 3.398764 | 9.831214 | 1.16E-20 | 1.65E-19 | 36.06961031 |
| **TMEM5** | 1.071702 | 3.031434 | 9.830335 | 1.17E-20 | 1.66E-19 | 36.06260672 |
| **SEC61A1** | 1.405994 | 5.858828 | 9.829457 | 1.18E-20 | 1.67E-19 | 36.05561909 |
| **PSMD12** | 1.1792 | 3.21607 | 9.829364 | 1.18E-20 | 1.67E-19 | 36.05488098 |
| **MAP2K3** | 0.960477 | 3.048439 | 9.829323 | 1.18E-20 | 1.67E-19 | 36.05454852 |
| **NAMPT** | 1.480353 | 3.473736 | 9.829184 | 1.18E-20 | 1.67E-19 | 36.05344317 |
| **SEC31A** | 1.219632 | 4.466496 | 9.828012 | 1.19E-20 | 1.68E-19 | 36.04410929 |
| **SNX6** | 1.253959 | 4.271981 | 9.827337 | 1.20E-20 | 1.69E-19 | 36.038735 |
| **CYP20A1** | 0.894992 | 1.929737 | 9.827198 | 1.20E-20 | 1.69E-19 | 36.03763202 |
| **PRDX4** | 1.478517 | 5.240451 | 9.826584 | 1.21E-20 | 1.70E-19 | 36.03274086 |
| **KLHL8** | 0.824178 | 1.972432 | 9.826173 | 1.21E-20 | 1.70E-19 | 36.02947302 |
| **TMEM8A** | 1.213164 | 4.411426 | 9.826095 | 1.21E-20 | 1.70E-19 | 36.02885075 |
| **SRGAP1** | 0.891074 | 1.936907 | 9.824794 | 1.22E-20 | 1.72E-19 | 36.01849577 |
| **CAAP1** | 0.890389 | 2.41351 | 9.824121 | 1.23E-20 | 1.73E-19 | 36.0131365 |
| **ACPL2** | 1.217272 | 3.351699 | 9.824034 | 1.23E-20 | 1.73E-19 | 36.01244269 |
| **DHX33** | 0.962739 | 2.305285 | 9.823645 | 1.24E-20 | 1.73E-19 | 36.0093456 |
| **TANGO6** | 0.647605 | 2.079663 | 9.823504 | 1.24E-20 | 1.73E-19 | 36.00822392 |
| **GID8** | 1.141317 | 3.752797 | 9.822043 | 1.25E-20 | 1.75E-19 | 35.99659754 |
| **FASTKD2** | 0.881498 | 2.45113 | 9.820885 | 1.26E-20 | 1.77E-19 | 35.98738238 |
| **PIK3AP1** | 0.809078 | 1.552543 | 9.820777 | 1.26E-20 | 1.77E-19 | 35.98652436 |
| **MEX3C** | 1.275094 | 3.102264 | 9.819857 | 1.27E-20 | 1.78E-19 | 35.97919831 |
| **FAM104A** | 0.994488 | 3.397651 | 9.819854 | 1.27E-20 | 1.78E-19 | 35.97917571 |
| **TMED10** | 1.599297 | 5.639358 | 9.818938 | 1.28E-20 | 1.79E-19 | 35.97189148 |
| **MYCN** | 1.319442 | 2.772381 | 9.818621 | 1.29E-20 | 1.80E-19 | 35.96936929 |
| **MTHFSD** | 0.925367 | 3.212198 | 9.817678 | 1.30E-20 | 1.81E-19 | 35.96186599 |
| **VPS33A** | 0.854053 | 2.792217 | 9.816824 | 1.31E-20 | 1.82E-19 | 35.95506754 |
| **ARRDC3** | 1.169701 | 2.671487 | 9.816701 | 1.31E-20 | 1.82E-19 | 35.95409277 |
| **SRPRB** | 1.088925 | 3.995933 | 9.816468 | 1.31E-20 | 1.82E-19 | 35.95223361 |
| **LCMT2** | 0.822334 | 2.21627 | 9.816175 | 1.31E-20 | 1.83E-19 | 35.94990611 |
| **AKAP8** | 1.00466 | 3.289239 | 9.816081 | 1.31E-20 | 1.83E-19 | 35.94915811 |
| **APBB2** | 1.339334 | 3.83174 | 9.813715 | 1.34E-20 | 1.86E-19 | 35.93033497 |
| **ETAA1** | 0.760897 | 1.976298 | 9.813338 | 1.34E-20 | 1.87E-19 | 35.92733395 |
| **PHF21A** | 0.954479 | 3.495962 | 9.813232 | 1.34E-20 | 1.87E-19 | 35.92649454 |
| **SLC30A5** | 1.070802 | 3.683744 | 9.812986 | 1.35E-20 | 1.87E-19 | 35.92453881 |
| **H2AFZ** | 1.734256 | 6.619347 | 9.812272 | 1.35E-20 | 1.88E-19 | 35.91886258 |
| **SAMHD1** | 1.10556 | 3.36871 | 9.812266 | 1.35E-20 | 1.88E-19 | 35.91881368 |
| **FZD2** | 0.830048 | 1.635953 | 9.811855 | 1.36E-20 | 1.88E-19 | 35.91554591 |
| **SMYD4** | 0.779513 | 2.104356 | 9.811668 | 1.36E-20 | 1.88E-19 | 35.91405838 |
| **EPC1** | 0.934772 | 3.224147 | 9.811479 | 1.36E-20 | 1.89E-19 | 35.91255238 |
| **OGFOD1** | 1.314135 | 3.186516 | 9.810528 | 1.37E-20 | 1.90E-19 | 35.90498991 |
| **NFE2L3** | 0.763092 | 1.549182 | 9.809798 | 1.38E-20 | 1.91E-19 | 35.89918103 |
| **USP13** | 0.635034 | 1.580878 | 9.809225 | 1.39E-20 | 1.92E-19 | 35.89462374 |
| **TRNT1** | 0.995333 | 2.918034 | 9.808428 | 1.40E-20 | 1.93E-19 | 35.88828447 |
| **ADSL** | 1.072873 | 3.932599 | 9.807788 | 1.40E-20 | 1.94E-19 | 35.8832007 |
| **EXOG** | 0.962765 | 2.387876 | 9.807738 | 1.40E-20 | 1.94E-19 | 35.88280013 |
| **TMEM79** | 0.804783 | 2.506981 | 9.807171 | 1.41E-20 | 1.94E-19 | 35.87829138 |
| **GGA3** | 1.111439 | 3.802452 | 9.807143 | 1.41E-20 | 1.94E-19 | 35.87807252 |
| **CNN3** | 1.963008 | 6.044042 | 9.806819 | 1.41E-20 | 1.95E-19 | 35.87549557 |
| **SCO1** | 0.96909 | 3.147472 | 9.805649 | 1.43E-20 | 1.97E-19 | 35.86619393 |
| **EPS8** | 1.112068 | 2.804802 | 9.805577 | 1.43E-20 | 1.97E-19 | 35.86562117 |
| **DR1** | 1.151998 | 2.589279 | 9.805097 | 1.43E-20 | 1.97E-19 | 35.86180632 |
| **RELA** | 1.235241 | 4.847539 | 9.803703 | 1.45E-20 | 1.99E-19 | 35.85072243 |
| **MMAA** | 0.550965 | 1.419618 | 9.803676 | 1.45E-20 | 1.99E-19 | 35.85051024 |
| **DDX18** | 1.306684 | 3.666802 | 9.80287 | 1.46E-20 | 2.00E-19 | 35.84409804 |
| **TYW1** | 0.885411 | 2.475822 | 9.80138 | 1.48E-20 | 2.03E-19 | 35.83225889 |
| **POLR1E** | 0.943895 | 3.290015 | 9.798751 | 1.51E-20 | 2.07E-19 | 35.81136563 |
| **RP11-15H20.6** | 0.806998 | 2.752902 | 9.798426 | 1.51E-20 | 2.07E-19 | 35.80878046 |
| **TNFAIP3** | 0.973599 | 1.593581 | 9.798312 | 1.51E-20 | 2.07E-19 | 35.80787161 |
| **LARP4** | 0.887023 | 2.455448 | 9.797295 | 1.53E-20 | 2.09E-19 | 35.79979523 |
| **MOGS** | 1.084457 | 4.431178 | 9.79497 | 1.56E-20 | 2.13E-19 | 35.78131772 |
| **CAV2** | 1.059319 | 1.942851 | 9.794934 | 1.56E-20 | 2.13E-19 | 35.78103341 |
| **CAND1** | 1.331578 | 3.899313 | 9.793634 | 1.57E-20 | 2.15E-19 | 35.77070153 |
| **CTCF** | 1.130037 | 3.54898 | 9.791784 | 1.60E-20 | 2.18E-19 | 35.7560074 |
| **NOX4** | 0.736571 | 1.043937 | 9.7914 | 1.60E-20 | 2.19E-19 | 35.75295676 |
| **RP11-196G18.22** | 0.814408 | 1.567406 | 9.790966 | 1.61E-20 | 2.19E-19 | 35.74950904 |
| **PGBD1** | 0.74855 | 2.151019 | 9.790659 | 1.61E-20 | 2.20E-19 | 35.7470755 |
| **SLC35C1** | 0.749404 | 2.403628 | 9.790342 | 1.62E-20 | 2.20E-19 | 35.7445579 |
| **PLOD3** | 1.317913 | 5.233501 | 9.789217 | 1.63E-20 | 2.22E-19 | 35.73561666 |
| **IPO9** | 1.026115 | 3.810956 | 9.788464 | 1.64E-20 | 2.23E-19 | 35.72964038 |
| **ZCCHC10** | 0.947088 | 3.076819 | 9.787708 | 1.65E-20 | 2.24E-19 | 35.72363655 |
| **AL357673.1** | 0.648724 | 1.393755 | 9.785131 | 1.68E-20 | 2.29E-19 | 35.70317474 |
| **SETD6** | 1.033243 | 3.526641 | 9.784553 | 1.69E-20 | 2.30E-19 | 35.69858114 |
| **HNRNPH1** | 1.619011 | 6.71067 | 9.782795 | 1.72E-20 | 2.33E-19 | 35.68462675 |
| **CEP170** | 1.354305 | 4.017276 | 9.781252 | 1.74E-20 | 2.36E-19 | 35.67237457 |
| **ZNF816** | 0.67566 | 1.194226 | 9.780922 | 1.74E-20 | 2.36E-19 | 35.66975816 |
| **C5orf28** | 0.862579 | 2.132051 | 9.780099 | 1.75E-20 | 2.38E-19 | 35.66322231 |
| **CPSF3** | 1.11917 | 3.858535 | 9.780034 | 1.75E-20 | 2.38E-19 | 35.66271326 |
| **QSOX2** | 0.934658 | 2.88727 | 9.779038 | 1.77E-20 | 2.40E-19 | 35.65480398 |
| **MED12** | 1.069818 | 2.953997 | 9.777156 | 1.80E-20 | 2.43E-19 | 35.63986515 |
| **LRP5** | 1.096225 | 3.772443 | 9.777143 | 1.80E-20 | 2.43E-19 | 35.63976341 |
| **DISP1** | 0.600041 | 1.504385 | 9.776801 | 1.80E-20 | 2.43E-19 | 35.63705162 |
| **TCEANC2** | 0.679171 | 1.39397 | 9.776445 | 1.81E-20 | 2.44E-19 | 35.63422379 |
| **SLAIN2** | 1.118204 | 2.379765 | 9.776232 | 1.81E-20 | 2.44E-19 | 35.63253887 |
| **COG8** | 1.062928 | 3.298048 | 9.775694 | 1.82E-20 | 2.45E-19 | 35.62826646 |
| **ZNF613** | 0.544709 | 1.375737 | 9.774455 | 1.84E-20 | 2.48E-19 | 35.61843592 |
| **RNF26** | 1.100985 | 3.631316 | 9.774147 | 1.84E-20 | 2.48E-19 | 35.61599348 |
| **CEP57** | 1.187505 | 3.466543 | 9.773975 | 1.84E-20 | 2.48E-19 | 35.61462585 |
| **LRCH3** | 1.080042 | 3.347567 | 9.77239 | 1.87E-20 | 2.51E-19 | 35.60205453 |
| **TLDC1** | 0.697711 | 2.013166 | 9.771716 | 1.88E-20 | 2.52E-19 | 35.59670568 |
| **GSTCD** | 0.767324 | 1.639678 | 9.771219 | 1.88E-20 | 2.53E-19 | 35.59276238 |
| **RALA** | 1.252457 | 4.044992 | 9.770725 | 1.89E-20 | 2.54E-19 | 35.58884374 |
| **CDC42SE1** | 1.438002 | 4.606662 | 9.769161 | 1.92E-20 | 2.57E-19 | 35.5764383 |
| **IRAK4** | 0.835107 | 2.212373 | 9.768957 | 1.92E-20 | 2.58E-19 | 35.57481469 |
| **URI1** | 1.205305 | 4.084006 | 9.768705 | 1.92E-20 | 2.58E-19 | 35.57282256 |
| **ANP32A** | 1.513689 | 5.982919 | 9.768345 | 1.93E-20 | 2.59E-19 | 35.56996106 |
| **RPL7L1** | 1.250685 | 4.545919 | 9.76803 | 1.93E-20 | 2.59E-19 | 35.56746627 |
| **RAD54B** | 0.796485 | 1.634611 | 9.767514 | 1.94E-20 | 2.60E-19 | 35.56337118 |
| **DNA2** | 0.618787 | 1.109057 | 9.767255 | 1.94E-20 | 2.60E-19 | 35.56132149 |
| **TADA2A** | 0.95445 | 2.972708 | 9.765093 | 1.98E-20 | 2.65E-19 | 35.54417076 |
| **SLC35F5** | 1.079884 | 2.706135 | 9.764914 | 1.98E-20 | 2.65E-19 | 35.54275588 |
| **GTPBP10** | 0.928767 | 2.311552 | 9.764643 | 1.99E-20 | 2.65E-19 | 35.54060656 |
| **HS2ST1** | 1.084314 | 2.618592 | 9.763691 | 2.00E-20 | 2.67E-19 | 35.53305851 |
| **ZNF512** | 1.210231 | 4.173619 | 9.763383 | 2.01E-20 | 2.68E-19 | 35.53061501 |
| **YME1L1** | 1.315673 | 3.85378 | 9.763089 | 2.01E-20 | 2.68E-19 | 35.5282843 |
| **CDH2** | 1.361746 | 3.746695 | 9.762468 | 2.02E-20 | 2.70E-19 | 35.52336298 |
| **PHTF1** | 0.930074 | 2.608918 | 9.761927 | 2.03E-20 | 2.71E-19 | 35.51906758 |
| **AHCY** | 1.294712 | 5.026628 | 9.761682 | 2.03E-20 | 2.71E-19 | 35.51712507 |
| **RPAP2** | 0.702233 | 1.919948 | 9.760826 | 2.05E-20 | 2.73E-19 | 35.51034125 |
| **PRRX1** | 1.438347 | 3.870414 | 9.760739 | 2.05E-20 | 2.73E-19 | 35.50964959 |
| **GORAB** | 1.097691 | 2.386653 | 9.758004 | 2.09E-20 | 2.79E-19 | 35.48797392 |
| **PYGL** | 1.201992 | 2.619454 | 9.757453 | 2.10E-20 | 2.80E-19 | 35.48360622 |
| **ZNF567** | 0.702232 | 1.533131 | 9.75706 | 2.11E-20 | 2.80E-19 | 35.48049375 |
| **SRF** | 1.019825 | 3.484541 | 9.755657 | 2.13E-20 | 2.83E-19 | 35.46937204 |
| **ADPGK** | 0.929069 | 3.780472 | 9.754001 | 2.16E-20 | 2.87E-19 | 35.45624744 |
| **SCAF8** | 0.943442 | 2.604853 | 9.753977 | 2.16E-20 | 2.87E-19 | 35.45606046 |
| **SF3B4** | 1.27174 | 4.314945 | 9.753902 | 2.17E-20 | 2.87E-19 | 35.45546756 |
| **PDIA6** | 1.498637 | 5.271432 | 9.753161 | 2.18E-20 | 2.89E-19 | 35.44958955 |
| **AC117395.1** | 1.066618 | 1.933336 | 9.749191 | 2.25E-20 | 2.98E-19 | 35.4181377 |
| **PLK3** | 1.001905 | 2.88884 | 9.748713 | 2.26E-20 | 2.99E-19 | 35.41435578 |
| **DCAF10** | 0.863373 | 2.201657 | 9.748388 | 2.26E-20 | 2.99E-19 | 35.41178095 |
| **DENND6A** | 0.85554 | 2.232624 | 9.748185 | 2.27E-20 | 3.00E-19 | 35.41016733 |
| **GGA2** | 1.082462 | 3.424258 | 9.747206 | 2.28E-20 | 3.02E-19 | 35.40241345 |
| **WDR75** | 1.088284 | 3.472673 | 9.746828 | 2.29E-20 | 3.03E-19 | 35.39942296 |
| **ACTN4** | 1.565346 | 6.284178 | 9.745774 | 2.31E-20 | 3.05E-19 | 35.39107672 |
| **ZNF746** | 1.097894 | 3.29712 | 9.744526 | 2.33E-20 | 3.08E-19 | 35.38119361 |
| **SMARCAD1** | 1.203957 | 2.622925 | 9.743874 | 2.35E-20 | 3.09E-19 | 35.37602678 |
| **C11orf30** | 1.036418 | 2.440075 | 9.742806 | 2.37E-20 | 3.12E-19 | 35.36757265 |
| **ORAI1** | 0.897566 | 2.677632 | 9.74255 | 2.37E-20 | 3.12E-19 | 35.36554352 |
| **ZFP3** | 0.800151 | 1.864579 | 9.742462 | 2.37E-20 | 3.12E-19 | 35.36484772 |
| **KIAA0100** | 1.1571 | 3.970496 | 9.740543 | 2.41E-20 | 3.17E-19 | 35.34964809 |
| **NMD3** | 1.176978 | 3.637193 | 9.740191 | 2.42E-20 | 3.18E-19 | 35.34686495 |
| **GNE** | 1.005272 | 2.412197 | 9.739614 | 2.43E-20 | 3.19E-19 | 35.34229889 |
| **SOCS3** | 1.447864 | 2.11963 | 9.739408 | 2.43E-20 | 3.19E-19 | 35.34066614 |
| **DIDO1** | 1.04138 | 3.448824 | 9.739217 | 2.44E-20 | 3.20E-19 | 35.33915535 |
| **9-Sep** | 1.451058 | 6.175726 | 9.739177 | 2.44E-20 | 3.20E-19 | 35.33883996 |
| **ANXA1** | 1.913109 | 3.830793 | 9.738899 | 2.44E-20 | 3.20E-19 | 35.33663635 |
| **C12orf23** | 1.156583 | 3.547428 | 9.738471 | 2.45E-20 | 3.21E-19 | 35.33325178 |
| **MSTO1** | 1.219765 | 4.37818 | 9.737441 | 2.47E-20 | 3.23E-19 | 35.32509269 |
| **ADPRH** | 0.676584 | 1.251069 | 9.737108 | 2.48E-20 | 3.24E-19 | 35.32245855 |
| **MTMR2** | 1.086189 | 3.057385 | 9.737091 | 2.48E-20 | 3.24E-19 | 35.3223272 |
| **HNRNPR** | 1.419797 | 5.344447 | 9.736903 | 2.48E-20 | 3.24E-19 | 35.32083663 |
| **CNPY4** | 1.092523 | 3.630236 | 9.736663 | 2.49E-20 | 3.25E-19 | 35.31893835 |
| **CIAO1** | 1.330928 | 4.708343 | 9.735183 | 2.52E-20 | 3.28E-19 | 35.30721865 |
| **RAB27A** | 0.729305 | 1.656146 | 9.734696 | 2.53E-20 | 3.30E-19 | 35.30336976 |
| **RAE1** | 0.949451 | 3.400475 | 9.733861 | 2.54E-20 | 3.32E-19 | 35.29675883 |
| **GTF2IRD2** | 1.171288 | 4.159792 | 9.733386 | 2.55E-20 | 3.33E-19 | 35.2930033 |
| **MMP16** | 1.07828 | 2.128681 | 9.733163 | 2.56E-20 | 3.33E-19 | 35.29123546 |
| **ZNF274** | 1.043144 | 3.08735 | 9.73191 | 2.58E-20 | 3.36E-19 | 35.28132213 |
| **WTAP** | 1.303472 | 4.507393 | 9.73174 | 2.59E-20 | 3.37E-19 | 35.27997657 |
| **OAS3** | 1.111894 | 2.059478 | 9.731407 | 2.59E-20 | 3.37E-19 | 35.27734027 |
| **TMEM201** | 0.85196 | 2.921482 | 9.731031 | 2.60E-20 | 3.38E-19 | 35.27436621 |
| **PECR** | 0.843063 | 2.431083 | 9.730808 | 2.61E-20 | 3.38E-19 | 35.27259955 |
| **HN1** | 1.861877 | 6.946496 | 9.73005 | 2.62E-20 | 3.40E-19 | 35.2666015 |
| **NARG2** | 0.959138 | 2.962279 | 9.729833 | 2.63E-20 | 3.41E-19 | 35.26488364 |
| **C7orf73** | 1.268679 | 4.356607 | 9.728641 | 2.65E-20 | 3.44E-19 | 35.25545563 |
| **EPC2** | 1.08031 | 2.686751 | 9.728625 | 2.65E-20 | 3.44E-19 | 35.25532777 |
| **FLVCR1** | 0.793078 | 1.894065 | 9.728559 | 2.65E-20 | 3.44E-19 | 35.25480457 |
| **PRUNE** | 0.887324 | 2.546984 | 9.728243 | 2.66E-20 | 3.44E-19 | 35.25231004 |
| **RPLP0P6** | 0.851865 | 2.099458 | 9.726933 | 2.69E-20 | 3.48E-19 | 35.24194645 |
| **BICD2** | 0.964215 | 2.971371 | 9.72583 | 2.71E-20 | 3.51E-19 | 35.23322405 |
| **ZNF704** | 1.113999 | 2.561355 | 9.723645 | 2.76E-20 | 3.57E-19 | 35.21594051 |
| **KDM2A** | 1.038454 | 3.367259 | 9.723431 | 2.76E-20 | 3.57E-19 | 35.21424632 |
| **C8orf4** | 1.231786 | 2.234373 | 9.722885 | 2.78E-20 | 3.58E-19 | 35.20993231 |
| **ZNF542** | 0.883119 | 2.392574 | 9.722256 | 2.79E-20 | 3.60E-19 | 35.20495551 |
| **ZNF501** | 0.925463 | 1.864839 | 9.72224 | 2.79E-20 | 3.60E-19 | 35.20482894 |
| **RBBP5** | 0.7869 | 1.875231 | 9.722187 | 2.79E-20 | 3.60E-19 | 35.20440649 |
| **ZXDC** | 1.033 | 2.830426 | 9.722047 | 2.80E-20 | 3.60E-19 | 35.20329857 |
| **CCNE2** | 0.90847 | 1.360397 | 9.721012 | 2.82E-20 | 3.63E-19 | 35.19511353 |
| **KDM5A** | 1.018541 | 2.184401 | 9.720227 | 2.84E-20 | 3.65E-19 | 35.18891245 |
| **NOL9** | 0.758796 | 2.295489 | 9.718758 | 2.87E-20 | 3.69E-19 | 35.17729404 |
| **IGSF9** | 0.557301 | 0.76532 | 9.718617 | 2.87E-20 | 3.69E-19 | 35.17618431 |
| **NMI** | 0.970648 | 2.348723 | 9.718497 | 2.88E-20 | 3.69E-19 | 35.17522912 |
| **COX18** | 0.908475 | 2.673333 | 9.717956 | 2.89E-20 | 3.71E-19 | 35.1709556 |
| **DENR** | 1.148144 | 3.801355 | 9.717489 | 2.90E-20 | 3.72E-19 | 35.1672649 |
| **ZNF730** | 0.544285 | 0.636009 | 9.71599 | 2.93E-20 | 3.76E-19 | 35.15540981 |
| **PTPN12** | 1.336188 | 3.84169 | 9.715651 | 2.94E-20 | 3.77E-19 | 35.15273118 |
| **RNF216** | 1.001535 | 3.556065 | 9.713979 | 2.98E-20 | 3.82E-19 | 35.13951464 |
| **CRTAP** | 1.258562 | 4.61113 | 9.713708 | 2.99E-20 | 3.83E-19 | 35.13737885 |
| **STK35** | 0.774052 | 2.18024 | 9.712284 | 3.02E-20 | 3.87E-19 | 35.12611846 |
| **PCDHB10** | 1.078024 | 2.354242 | 9.711919 | 3.03E-20 | 3.88E-19 | 35.12323794 |
| **C16orf59** | 0.898835 | 2.509848 | 9.711168 | 3.05E-20 | 3.90E-19 | 35.11730463 |
| **TRIAP1** | 1.11168 | 3.848267 | 9.710314 | 3.07E-20 | 3.92E-19 | 35.11055084 |
| **TMEM37** | 0.626645 | 1.120205 | 9.7096 | 3.09E-20 | 3.94E-19 | 35.10491533 |
| **TRAM1** | 1.556282 | 3.538287 | 9.709035 | 3.10E-20 | 3.96E-19 | 35.10045223 |
| **BTG2** | 1.409488 | 4.857245 | 9.708409 | 3.12E-20 | 3.98E-19 | 35.09550335 |
| **RELT** | 0.908154 | 2.91968 | 9.706188 | 3.17E-20 | 4.05E-19 | 35.07795506 |
| **CDC73** | 0.967575 | 2.502404 | 9.703399 | 3.25E-20 | 4.14E-19 | 35.05592232 |
| **DET1** | 0.698859 | 1.856611 | 9.703156 | 3.25E-20 | 4.14E-19 | 35.05400944 |
| **AGPAT6** | 1.25245 | 5.027407 | 9.702625 | 3.27E-20 | 4.16E-19 | 35.04981504 |
| **GNPDA1** | 1.188197 | 4.114817 | 9.701544 | 3.29E-20 | 4.19E-19 | 35.04127901 |
| **QRSL1** | 0.843086 | 2.530276 | 9.700782 | 3.31E-20 | 4.21E-19 | 35.03525976 |
| **TRMT6** | 0.934286 | 2.518497 | 9.700598 | 3.32E-20 | 4.22E-19 | 35.0338016 |
| **PPHLN1** | 1.161515 | 4.112392 | 9.700522 | 3.32E-20 | 4.22E-19 | 35.0332015 |
| **ZNF738** | 1.185769 | 2.721953 | 9.700252 | 3.33E-20 | 4.23E-19 | 35.03107115 |
| **DNAJC2** | 1.022614 | 3.368089 | 9.700064 | 3.33E-20 | 4.23E-19 | 35.02958867 |
| **PCGF3** | 1.120705 | 3.864951 | 9.699457 | 3.35E-20 | 4.25E-19 | 35.02479147 |
| **FAF2** | 1.040462 | 3.088379 | 9.699154 | 3.36E-20 | 4.26E-19 | 35.02240513 |
| **PPP6R3** | 1.066768 | 3.860095 | 9.698682 | 3.37E-20 | 4.27E-19 | 35.01867213 |
| **MTRR** | 1.295886 | 3.24537 | 9.698532 | 3.37E-20 | 4.27E-19 | 35.01749285 |
| **WDR5B** | 0.705843 | 1.659673 | 9.698035 | 3.39E-20 | 4.29E-19 | 35.01356572 |
| **ABCB4** | 0.578379 | 0.858437 | 9.697097 | 3.41E-20 | 4.32E-19 | 35.00616527 |
| **ZNF569** | 0.701279 | 1.65734 | 9.696553 | 3.43E-20 | 4.33E-19 | 35.00186445 |
| **PARP1** | 1.366627 | 5.251517 | 9.696539 | 3.43E-20 | 4.33E-19 | 35.00175962 |
| **AEBP2** | 0.918234 | 2.69314 | 9.696032 | 3.44E-20 | 4.35E-19 | 34.9977566 |
| **MAD2L2** | 1.506804 | 5.675717 | 9.695529 | 3.46E-20 | 4.36E-19 | 34.99378078 |
| **USP38** | 0.832068 | 2.123707 | 9.69519 | 3.47E-20 | 4.37E-19 | 34.99110867 |
| **PAK1IP1** | 1.046344 | 2.774142 | 9.694603 | 3.48E-20 | 4.39E-19 | 34.98647644 |
| **FAM175A** | 0.682204 | 1.489096 | 9.694474 | 3.49E-20 | 4.39E-19 | 34.98545887 |
| **GTPBP2** | 1.155804 | 4.060521 | 9.693199 | 3.52E-20 | 4.44E-19 | 34.9753925 |
| **TSPAN12** | 1.311996 | 2.817059 | 9.692987 | 3.53E-20 | 4.44E-19 | 34.97371668 |
| **TCTN3** | 0.944036 | 3.277738 | 9.691891 | 3.56E-20 | 4.48E-19 | 34.96506789 |
| **RP11-443B20.1** | 0.563898 | 1.046523 | 9.690992 | 3.58E-20 | 4.51E-19 | 34.95796864 |
| **NFRKB** | 0.859254 | 2.720494 | 9.689584 | 3.63E-20 | 4.56E-19 | 34.94686396 |
| **AGPS** | 1.053529 | 2.450274 | 9.685195 | 3.75E-20 | 4.71E-19 | 34.91222866 |
| **GNA13** | 1.208688 | 3.372152 | 9.684828 | 3.77E-20 | 4.73E-19 | 34.90933677 |
| **CPXM1** | 1.634083 | 4.321717 | 9.684657 | 3.77E-20 | 4.73E-19 | 34.90798795 |
| **LRRC8D** | 1.172564 | 3.089068 | 9.683623 | 3.80E-20 | 4.77E-19 | 34.8998342 |
| **SUMF1** | 0.89479 | 3.011586 | 9.683534 | 3.80E-20 | 4.77E-19 | 34.89913193 |
| **HELQ** | 0.749292 | 1.844681 | 9.68288 | 3.82E-20 | 4.79E-19 | 34.89396723 |
| **POU3F3** | 1.388288 | 4.885709 | 9.682161 | 3.85E-20 | 4.81E-19 | 34.8883001 |
| **CEP63** | 1.000068 | 3.411628 | 9.681913 | 3.85E-20 | 4.82E-19 | 34.88634032 |
| **MTERFD1** | 0.981643 | 3.077351 | 9.681719 | 3.86E-20 | 4.83E-19 | 34.88481327 |
| **NUP43** | 0.858012 | 2.754263 | 9.68046 | 3.90E-20 | 4.87E-19 | 34.87488383 |
| **SNAP29** | 0.931963 | 3.176109 | 9.679865 | 3.92E-20 | 4.89E-19 | 34.87019194 |
| **RAP1B** | 1.390885 | 4.605008 | 9.67885 | 3.95E-20 | 4.93E-19 | 34.86218412 |
| **OLFML3** | 1.241788 | 3.741128 | 9.678833 | 3.95E-20 | 4.93E-19 | 34.862053 |
| **ARID1B** | 1.110594 | 2.784603 | 9.678008 | 3.98E-20 | 4.96E-19 | 34.85554558 |
| **PIP5K1A** | 1.206534 | 3.242669 | 9.677079 | 4.01E-20 | 4.99E-19 | 34.84822065 |
| **SLC25A13** | 1.025008 | 2.78867 | 9.675476 | 4.06E-20 | 5.05E-19 | 34.83558704 |
| **ZNF112** | 0.690878 | 1.466384 | 9.673143 | 4.13E-20 | 5.14E-19 | 34.81719021 |
| **TMCC1** | 1.011862 | 3.119156 | 9.672492 | 4.16E-20 | 5.17E-19 | 34.81205844 |
| **ASB16-AS1** | 0.656641 | 1.840453 | 9.670919 | 4.21E-20 | 5.23E-19 | 34.79966464 |
| **SP3** | 1.39671 | 3.296658 | 9.670862 | 4.21E-20 | 5.23E-19 | 34.79921717 |
| **CDV3** | 1.39975 | 4.866904 | 9.67053 | 4.22E-20 | 5.24E-19 | 34.79659398 |
| **C1orf21** | 1.11303 | 3.32383 | 9.669171 | 4.27E-20 | 5.30E-19 | 34.78588352 |
| **DPY19L3** | 1.16632 | 3.288632 | 9.668227 | 4.30E-20 | 5.33E-19 | 34.77844967 |
| **CREB3L4** | 0.998198 | 2.936403 | 9.667064 | 4.34E-20 | 5.38E-19 | 34.76928203 |
| **ABCE1** | 1.259177 | 3.239444 | 9.665817 | 4.38E-20 | 5.43E-19 | 34.75945584 |
| **PRRC2B** | 1.341004 | 5.562128 | 9.664588 | 4.43E-20 | 5.48E-19 | 34.74977297 |
| **WDR36** | 0.78798 | 1.791621 | 9.663737 | 4.46E-20 | 5.52E-19 | 34.74307361 |
| **HMOX1** | 1.4498 | 3.773971 | 9.66346 | 4.47E-20 | 5.53E-19 | 34.74089138 |
| **LLPH** | 1.169637 | 4.074092 | 9.661059 | 4.55E-20 | 5.63E-19 | 34.72197443 |
| **RAB35** | 1.176324 | 4.053625 | 9.660719 | 4.57E-20 | 5.64E-19 | 34.71929639 |
| **UBP1** | 1.162771 | 3.651881 | 9.659933 | 4.59E-20 | 5.68E-19 | 34.71310776 |
| **HPS3** | 0.855876 | 2.684232 | 9.659444 | 4.61E-20 | 5.69E-19 | 34.70925409 |
| **HSD17B11** | 1.194986 | 3.539081 | 9.658911 | 4.63E-20 | 5.72E-19 | 34.70505528 |
| **UBE2H** | 1.324959 | 4.74075 | 9.658462 | 4.65E-20 | 5.73E-19 | 34.70152604 |
| **H2AFX** | 1.358435 | 5.993781 | 9.658344 | 4.65E-20 | 5.74E-19 | 34.70059447 |
| **ZNF230** | 0.526254 | 0.992911 | 9.658285 | 4.66E-20 | 5.74E-19 | 34.70012734 |
| **PCYT1A** | 1.108894 | 3.479971 | 9.657027 | 4.70E-20 | 5.79E-19 | 34.6902264 |
| **ALYREF** | 1.444246 | 5.583602 | 9.656099 | 4.74E-20 | 5.83E-19 | 34.68291919 |
| **NOP16** | 1.039151 | 3.364788 | 9.655597 | 4.76E-20 | 5.85E-19 | 34.67896369 |
| **SQLE** | 1.16608 | 3.659533 | 9.655355 | 4.77E-20 | 5.86E-19 | 34.67705553 |
| **DDX19A** | 1.045733 | 3.929938 | 9.654544 | 4.80E-20 | 5.89E-19 | 34.6706716 |
| **RNF216P1** | 0.898413 | 3.124165 | 9.654185 | 4.81E-20 | 5.91E-19 | 34.66784884 |
| **ADAR** | 1.344768 | 4.594906 | 9.653435 | 4.84E-20 | 5.94E-19 | 34.66194406 |
| **SMG8** | 1.012532 | 2.080545 | 9.652437 | 4.88E-20 | 5.98E-19 | 34.6540867 |
| **SMURF1** | 0.915214 | 2.474367 | 9.652366 | 4.88E-20 | 5.98E-19 | 34.65352281 |
| **MARS2** | 0.775346 | 1.411238 | 9.652158 | 4.89E-20 | 5.99E-19 | 34.65188824 |
| **RPS6KB1** | 1.025357 | 2.742883 | 9.651591 | 4.91E-20 | 6.01E-19 | 34.6474248 |
| **ZNF491** | 0.563227 | 1.215236 | 9.651376 | 4.92E-20 | 6.02E-19 | 34.64573296 |
| **ALKBH1** | 0.711185 | 2.072578 | 9.650006 | 4.97E-20 | 6.08E-19 | 34.63495373 |
| **GLCCI1** | 1.22273 | 3.345692 | 9.649764 | 4.98E-20 | 6.09E-19 | 34.63304895 |
| **HSPA5** | 1.509843 | 5.511147 | 9.649621 | 4.99E-20 | 6.10E-19 | 34.63191879 |
| **SLC39A6** | 1.388196 | 4.130167 | 9.648339 | 5.04E-20 | 6.16E-19 | 34.62183086 |
| **PSME3** | 1.480328 | 4.930243 | 9.647997 | 5.05E-20 | 6.17E-19 | 34.61914189 |
| **FAM117B** | 0.674575 | 1.649982 | 9.647871 | 5.06E-20 | 6.17E-19 | 34.61814524 |
| **SOS1** | 1.030924 | 2.466384 | 9.647637 | 5.07E-20 | 6.18E-19 | 34.61630419 |
| **LYN** | 1.020546 | 2.365214 | 9.646353 | 5.12E-20 | 6.24E-19 | 34.60619912 |
| **GOLGA2** | 1.187395 | 4.212134 | 9.64588 | 5.14E-20 | 6.26E-19 | 34.60247831 |
| **KIAA0355** | 0.909089 | 2.241461 | 9.645023 | 5.17E-20 | 6.30E-19 | 34.59573564 |
| **COL1A2** | 1.462009 | 3.030676 | 9.644669 | 5.19E-20 | 6.31E-19 | 34.59294769 |
| **EDC3** | 0.936125 | 3.554469 | 9.644607 | 5.19E-20 | 6.31E-19 | 34.59246554 |
| **SIPA1L2** | 1.088997 | 3.003775 | 9.64459 | 5.19E-20 | 6.31E-19 | 34.59232832 |
| **CNOT2** | 1.214897 | 4.131972 | 9.643618 | 5.23E-20 | 6.36E-19 | 34.58468575 |
| **PHF6** | 1.104203 | 2.584972 | 9.64345 | 5.24E-20 | 6.37E-19 | 34.58336223 |
| **MTIF2** | 0.917757 | 3.063429 | 9.643388 | 5.24E-20 | 6.37E-19 | 34.58287122 |
| **SLX4** | 0.69107 | 1.867412 | 9.642063 | 5.30E-20 | 6.43E-19 | 34.57244645 |
| **TOP1** | 1.412979 | 3.681943 | 9.640249 | 5.38E-20 | 6.52E-19 | 34.55817801 |
| **KIAA0040** | 0.896672 | 1.3631 | 9.640151 | 5.38E-20 | 6.52E-19 | 34.55740759 |
| **ZNF660** | 0.581106 | 1.26949 | 9.640083 | 5.38E-20 | 6.52E-19 | 34.55687669 |
| **CPSF2** | 0.907533 | 2.806547 | 9.639956 | 5.39E-20 | 6.53E-19 | 34.55587506 |
| **GALR1** | 0.861917 | 0.949871 | 9.63983 | 5.39E-20 | 6.53E-19 | 34.55488254 |
| **RPS2P5** | 1.888424 | 5.909079 | 9.639355 | 5.41E-20 | 6.55E-19 | 34.55114668 |
| **UTP18** | 1.058905 | 3.974374 | 9.637951 | 5.47E-20 | 6.62E-19 | 34.54010897 |
| **GPN3** | 1.041392 | 3.281095 | 9.637566 | 5.49E-20 | 6.64E-19 | 34.53708111 |
| **ZFYVE26** | 0.822581 | 2.250069 | 9.635537 | 5.58E-20 | 6.74E-19 | 34.52112487 |
| **KHDRBS1** | 1.340102 | 5.136988 | 9.634466 | 5.63E-20 | 6.80E-19 | 34.51270072 |
| **GPR173** | 1.098716 | 3.267967 | 9.633496 | 5.67E-20 | 6.85E-19 | 34.50507872 |
| **IRAK1** | 1.25092 | 5.213131 | 9.63338 | 5.68E-20 | 6.85E-19 | 34.50416017 |
| **MFSD11** | 0.895562 | 3.428911 | 9.633203 | 5.69E-20 | 6.85E-19 | 34.50277154 |
| **NUP98** | 1.034658 | 3.104594 | 9.633094 | 5.69E-20 | 6.86E-19 | 34.50191244 |
| **VCL** | 1.160091 | 2.579188 | 9.632673 | 5.71E-20 | 6.87E-19 | 34.49860666 |
| **PARG** | 0.83494 | 2.356618 | 9.632672 | 5.71E-20 | 6.87E-19 | 34.49859347 |
| **MXD3** | 1.317053 | 4.048079 | 9.631754 | 5.75E-20 | 6.92E-19 | 34.4913826 |
| **UBALD2** | 1.597245 | 5.874825 | 9.631548 | 5.76E-20 | 6.93E-19 | 34.48975856 |
| **AK2** | 1.353272 | 5.126642 | 9.63095 | 5.79E-20 | 6.96E-19 | 34.4850584 |
| **NEK6** | 1.207394 | 3.555386 | 9.630762 | 5.80E-20 | 6.96E-19 | 34.48358141 |
| **KIAA0907** | 1.178351 | 3.786276 | 9.630561 | 5.81E-20 | 6.97E-19 | 34.48200019 |
| **LRIG2** | 0.669364 | 1.502793 | 9.630511 | 5.81E-20 | 6.97E-19 | 34.48161261 |
| **RPGRIP1L** | 0.717185 | 1.753236 | 9.629086 | 5.88E-20 | 7.05E-19 | 34.47040984 |
| **MMRN2** | 0.828798 | 1.905918 | 9.628563 | 5.90E-20 | 7.07E-19 | 34.46629546 |
| **C1QTNF6** | 0.870203 | 2.269161 | 9.628442 | 5.91E-20 | 7.08E-19 | 34.46534615 |
| **PBRM1** | 1.098827 | 2.545733 | 9.62817 | 5.92E-20 | 7.09E-19 | 34.46321156 |
| **ZDHHC7** | 1.102165 | 3.627403 | 9.627889 | 5.93E-20 | 7.10E-19 | 34.46099692 |
| **SLFN12** | 0.568322 | 0.860671 | 9.627314 | 5.96E-20 | 7.13E-19 | 34.45647997 |
| **CAP1** | 1.485969 | 5.220342 | 9.626668 | 5.99E-20 | 7.16E-19 | 34.45140077 |
| **ZNF671** | 0.839466 | 2.634344 | 9.626261 | 6.01E-20 | 7.18E-19 | 34.44820325 |
| **KIN** | 0.939305 | 2.876889 | 9.626017 | 6.02E-20 | 7.19E-19 | 34.44628958 |
| **SEC24A** | 0.84698 | 1.541142 | 9.625589 | 6.04E-20 | 7.21E-19 | 34.44292328 |
| **PNRC2** | 1.331256 | 4.062855 | 9.623928 | 6.12E-20 | 7.31E-19 | 34.4298737 |
| **ZNF500** | 0.843363 | 2.725293 | 9.623137 | 6.16E-20 | 7.35E-19 | 34.4236579 |
| **ZNF285** | 0.688887 | 1.621923 | 9.620941 | 6.27E-20 | 7.48E-19 | 34.40640686 |
| **DHX57** | 0.915572 | 2.768916 | 9.620179 | 6.31E-20 | 7.52E-19 | 34.4004217 |
| **ADAM12** | 0.777549 | 0.775709 | 9.619946 | 6.32E-20 | 7.53E-19 | 34.39859306 |
| **CCNT1** | 0.833617 | 1.734028 | 9.618953 | 6.37E-20 | 7.58E-19 | 34.39079098 |
| **RNF139** | 1.134651 | 3.429419 | 9.618059 | 6.42E-20 | 7.63E-19 | 34.38376495 |
| **CDK13** | 0.955317 | 2.973384 | 9.616969 | 6.47E-20 | 7.69E-19 | 34.37520548 |
| **FAM122C** | 0.800549 | 1.919876 | 9.615665 | 6.54E-20 | 7.77E-19 | 34.36496774 |
| **ZNF383** | 0.681046 | 1.435923 | 9.614953 | 6.58E-20 | 7.81E-19 | 34.35937306 |
| **C1orf131** | 1.111081 | 3.10094 | 9.613134 | 6.67E-20 | 7.92E-19 | 34.34508968 |
| **GTF2B** | 1.076759 | 3.500787 | 9.612604 | 6.70E-20 | 7.95E-19 | 34.34092873 |
| **RAC2** | 0.99523 | 1.804304 | 9.612416 | 6.71E-20 | 7.96E-19 | 34.33945809 |
| **MAPKAPK5** | 1.015803 | 3.50571 | 9.611623 | 6.75E-20 | 8.00E-19 | 34.33323233 |
| **TET3** | 0.815634 | 1.706686 | 9.610778 | 6.80E-20 | 8.05E-19 | 34.32659524 |
| **ERAP1** | 1.09799 | 2.63073 | 9.610117 | 6.83E-20 | 8.09E-19 | 34.32140416 |
| **STK4** | 0.935441 | 2.274831 | 9.608681 | 6.91E-20 | 8.18E-19 | 34.31013836 |
| **CBLB** | 0.954492 | 2.323046 | 9.608163 | 6.94E-20 | 8.21E-19 | 34.30607236 |
| **EIF4EBP2** | 1.085888 | 3.691932 | 9.60802 | 6.95E-20 | 8.22E-19 | 34.30494877 |
| **MAML1** | 0.898305 | 3.173785 | 9.606957 | 7.01E-20 | 8.28E-19 | 34.29660409 |
| **RP11-398K22.12** | 0.76343 | 1.988118 | 9.606505 | 7.03E-20 | 8.31E-19 | 34.29305288 |
| **ZNF468** | 0.773665 | 1.186871 | 9.606024 | 7.06E-20 | 8.33E-19 | 34.28928271 |
| **GNL3** | 1.339584 | 4.273314 | 9.605481 | 7.09E-20 | 8.37E-19 | 34.28501759 |
| **DVL2** | 1.211576 | 4.454523 | 9.605256 | 7.10E-20 | 8.38E-19 | 34.28325343 |
| **DCLRE1A** | 0.762574 | 1.662242 | 9.604747 | 7.13E-20 | 8.41E-19 | 34.27926033 |
| **AC004797.1** | 0.861764 | 1.350824 | 9.604252 | 7.16E-20 | 8.44E-19 | 34.27537604 |
| **POLR2B** | 1.423981 | 3.897579 | 9.604032 | 7.17E-20 | 8.45E-19 | 34.27364715 |
| **SCMH1** | 0.885178 | 3.024211 | 9.60397 | 7.18E-20 | 8.45E-19 | 34.27316092 |
| **TIMM8A** | 0.704523 | 2.158222 | 9.60385 | 7.18E-20 | 8.45E-19 | 34.27221951 |
| **EIF2AK3** | 0.988834 | 2.035817 | 9.603068 | 7.23E-20 | 8.50E-19 | 34.26608966 |
| **CYFIP1** | 1.289803 | 4.268808 | 9.601233 | 7.33E-20 | 8.62E-19 | 34.25169089 |
| **MAFF** | 1.038297 | 2.426902 | 9.600805 | 7.36E-20 | 8.65E-19 | 34.24833385 |
| **HP1BP3** | 1.5072 | 5.2091 | 9.60068 | 7.37E-20 | 8.65E-19 | 34.24735219 |
| **SGCB** | 1.350862 | 4.033755 | 9.600641 | 7.37E-20 | 8.65E-19 | 34.24704342 |
| **FAM200A** | 0.733096 | 2.157019 | 9.600612 | 7.37E-20 | 8.65E-19 | 34.24681901 |
| **WDR53** | 0.811113 | 2.769415 | 9.600373 | 7.38E-20 | 8.66E-19 | 34.24494508 |
| **DNAJB5** | 1.285501 | 4.355319 | 9.60035 | 7.39E-20 | 8.66E-19 | 34.24475977 |
| **ZSCAN32** | 0.860994 | 2.823156 | 9.598311 | 7.51E-20 | 8.79E-19 | 34.22876401 |
| **TSTD2** | 1.211356 | 2.295272 | 9.597711 | 7.54E-20 | 8.83E-19 | 34.22406114 |
| **CRNKL1** | 0.910154 | 3.12924 | 9.597222 | 7.57E-20 | 8.86E-19 | 34.22022826 |
| **RPN1** | 1.298103 | 5.463243 | 9.597196 | 7.57E-20 | 8.86E-19 | 34.22001712 |
| **C19orf48** | 1.193306 | 4.148005 | 9.59692 | 7.59E-20 | 8.87E-19 | 34.21785291 |
| **FAM120C** | 0.760186 | 2.594796 | 9.596336 | 7.63E-20 | 8.91E-19 | 34.21327667 |
| **ZNF473** | 0.694416 | 1.819152 | 9.595607 | 7.67E-20 | 8.96E-19 | 34.20755926 |
| **HDAC1** | 1.298805 | 4.323519 | 9.59472 | 7.72E-20 | 9.02E-19 | 34.20060254 |
| **MEMO1** | 1.019734 | 3.622093 | 9.592403 | 7.87E-20 | 9.18E-19 | 34.18243432 |
| **ZBTB24** | 0.546224 | 1.516581 | 9.591608 | 7.92E-20 | 9.23E-19 | 34.17620073 |
| **PTCHD3P1** | 1.099709 | 3.495518 | 9.591535 | 7.92E-20 | 9.23E-19 | 34.17563188 |
| **GEMIN2** | 0.940313 | 2.947065 | 9.591068 | 7.95E-20 | 9.26E-19 | 34.17196873 |
| **MSR1** | 1.178751 | 1.945667 | 9.59064 | 7.98E-20 | 9.29E-19 | 34.16861052 |
| **ZNF394** | 0.831811 | 3.261683 | 9.590415 | 7.99E-20 | 9.30E-19 | 34.16684569 |
| **ZBTB8A** | 0.526127 | 1.061111 | 9.590318 | 8.00E-20 | 9.31E-19 | 34.16608762 |
| **H2AFY2** | 1.274121 | 3.890902 | 9.59004 | 8.02E-20 | 9.31E-19 | 34.16390468 |
| **SPECC1L** | 0.878344 | 3.18465 | 9.590021 | 8.02E-20 | 9.31E-19 | 34.16375788 |
| **PVT1** | 0.910325 | 1.488297 | 9.590017 | 8.02E-20 | 9.31E-19 | 34.16372634 |
| **TARBP2** | 1.111069 | 4.372112 | 9.588874 | 8.09E-20 | 9.40E-19 | 34.15476984 |
| **TMCO6** | 0.959029 | 2.697205 | 9.58873 | 8.10E-20 | 9.40E-19 | 34.15363535 |
| **SIGMAR1** | 1.263324 | 4.799712 | 9.588054 | 8.14E-20 | 9.45E-19 | 34.14833995 |
| **AIMP1** | 1.155199 | 3.856305 | 9.586727 | 8.23E-20 | 9.54E-19 | 34.13793969 |
| **DAP** | 1.309949 | 4.437979 | 9.585033 | 8.34E-20 | 9.67E-19 | 34.12465912 |
| **ZCCHC4** | 0.690035 | 1.713669 | 9.584808 | 8.36E-20 | 9.68E-19 | 34.12289593 |
| **RPA1** | 1.152693 | 3.872667 | 9.584688 | 8.36E-20 | 9.68E-19 | 34.12196018 |
| **ZC4H2** | 1.040124 | 3.317476 | 9.583895 | 8.42E-20 | 9.74E-19 | 34.1157422 |
| **KANSL1** | 1.087795 | 3.339269 | 9.583579 | 8.44E-20 | 9.76E-19 | 34.11326345 |
| **ASCC3** | 0.942783 | 2.417492 | 9.582933 | 8.48E-20 | 9.81E-19 | 34.10820419 |
| **ARID3A** | 0.869611 | 2.00179 | 9.582725 | 8.50E-20 | 9.82E-19 | 34.10657819 |
| **HMGN1** | 1.69639 | 6.371358 | 9.582637 | 8.50E-20 | 9.82E-19 | 34.10588469 |
| **ZBTB49** | 0.591862 | 1.589409 | 9.582479 | 8.51E-20 | 9.83E-19 | 34.10464573 |
| **IRF2BPL** | 1.355883 | 3.558556 | 9.582446 | 8.52E-20 | 9.83E-19 | 34.10438541 |
| **DDX21** | 1.203993 | 2.640738 | 9.582381 | 8.52E-20 | 9.83E-19 | 34.10388118 |
| **SIM2** | 0.919444 | 1.549168 | 9.581961 | 8.55E-20 | 9.85E-19 | 34.10058747 |
| **SLC43A2** | 1.096609 | 4.227471 | 9.58046 | 8.65E-20 | 9.97E-19 | 34.08882942 |
| **APH1A** | 1.363852 | 5.757951 | 9.579714 | 8.70E-20 | 1.00E-18 | 34.08298582 |
| **SP2** | 1.014506 | 2.851806 | 9.578264 | 8.80E-20 | 1.01E-18 | 34.07162687 |
| **TAF5L** | 0.982768 | 2.824844 | 9.577907 | 8.83E-20 | 1.02E-18 | 34.06882706 |
| **TM4SF18** | 0.932299 | 1.642457 | 9.57783 | 8.83E-20 | 1.02E-18 | 34.06822615 |
| **LINC00662** | 1.036392 | 2.844404 | 9.57735 | 8.87E-20 | 1.02E-18 | 34.06446857 |
| **SPTY2D1** | 0.658989 | 1.474745 | 9.576383 | 8.94E-20 | 1.03E-18 | 34.05689021 |
| **NDOR1** | 0.861172 | 2.779965 | 9.575719 | 8.98E-20 | 1.03E-18 | 34.0516905 |
| **CNNM3** | 0.943799 | 3.235129 | 9.574846 | 9.04E-20 | 1.04E-18 | 34.04485434 |
| **PATZ1** | 1.178278 | 4.273969 | 9.573862 | 9.12E-20 | 1.05E-18 | 34.0371506 |
| **NAA40** | 1.09466 | 3.353587 | 9.572977 | 9.18E-20 | 1.05E-18 | 34.03021525 |
| **LAMA4** | 1.257688 | 3.815878 | 9.572791 | 9.19E-20 | 1.05E-18 | 34.0287617 |
| **ANKRD13A** | 1.038335 | 3.549821 | 9.572074 | 9.25E-20 | 1.06E-18 | 34.02314389 |
| **ELAC1** | 0.841487 | 2.527498 | 9.57097 | 9.33E-20 | 1.07E-18 | 34.0145025 |
| **ZSCAN21** | 0.903452 | 2.897587 | 9.569921 | 9.41E-20 | 1.08E-18 | 34.00629067 |
| **OTUD6B** | 0.919069 | 2.516467 | 9.569268 | 9.45E-20 | 1.08E-18 | 34.00117833 |
| **TFRC** | 1.161337 | 3.13221 | 9.569181 | 9.46E-20 | 1.08E-18 | 34.0004934 |
| **ABCD1** | 0.878211 | 2.824505 | 9.569124 | 9.47E-20 | 1.08E-18 | 34.00004638 |
| **GNPAT** | 1.100012 | 3.985358 | 9.566614 | 9.66E-20 | 1.10E-18 | 33.98040517 |
| **WDR26** | 1.226531 | 3.677434 | 9.566564 | 9.66E-20 | 1.10E-18 | 33.9800111 |
| **KCNE3** | 0.776126 | 1.187348 | 9.565678 | 9.73E-20 | 1.11E-18 | 33.97307752 |
| **TMEM127** | 1.118586 | 3.704942 | 9.564552 | 9.81E-20 | 1.12E-18 | 33.96426208 |
| **DOPEY2** | 0.817534 | 1.608964 | 9.563679 | 9.88E-20 | 1.13E-18 | 33.95743007 |
| **AKT2** | 1.376836 | 5.250329 | 9.563392 | 9.91E-20 | 1.13E-18 | 33.95518482 |
| **CUL4B** | 1.081369 | 3.300759 | 9.563067 | 9.93E-20 | 1.13E-18 | 33.95264349 |
| **ZNF684** | 0.605527 | 1.630554 | 9.559662 | 1.02E-19 | 1.16E-18 | 33.92599588 |
| **PTPRZ1** | 1.970384 | 6.734974 | 9.559326 | 1.02E-19 | 1.16E-18 | 33.92336722 |
| **ADNP2** | 0.884818 | 1.993013 | 9.559103 | 1.02E-19 | 1.17E-18 | 33.92162841 |
| **CTSK** | 1.121597 | 2.61261 | 9.558561 | 1.03E-19 | 1.17E-18 | 33.917388 |
| **ZNF566** | 0.820893 | 2.321057 | 9.557635 | 1.04E-19 | 1.18E-18 | 33.91014084 |
| **ZXDB** | 0.784895 | 1.705718 | 9.55762 | 1.04E-19 | 1.18E-18 | 33.91002406 |
| **SPRED1** | 1.086901 | 2.709256 | 9.557202 | 1.04E-19 | 1.18E-18 | 33.9067532 |
| **TRIM65** | 0.927279 | 3.368176 | 9.556139 | 1.05E-19 | 1.19E-18 | 33.89843876 |
| **RBMS1** | 1.125848 | 2.250024 | 9.55602 | 1.05E-19 | 1.19E-18 | 33.89750589 |
| **WDR89** | 0.679968 | 2.342642 | 9.553856 | 1.07E-19 | 1.21E-18 | 33.88057953 |
| **DDX47** | 1.219728 | 4.166985 | 9.553853 | 1.07E-19 | 1.21E-18 | 33.88056022 |
| **CEP76** | 0.681117 | 1.460073 | 9.553378 | 1.07E-19 | 1.21E-18 | 33.87684535 |
| **ARRDC4** | 1.144522 | 2.939931 | 9.553027 | 1.08E-19 | 1.22E-18 | 33.87409798 |
| **KLF3** | 1.104233 | 2.721893 | 9.551346 | 1.09E-19 | 1.23E-18 | 33.86095751 |
| **TM9SF4** | 1.151432 | 4.042292 | 9.550926 | 1.09E-19 | 1.24E-18 | 33.85766825 |
| **TSEN34** | 1.201675 | 4.501871 | 9.549756 | 1.10E-19 | 1.25E-18 | 33.84852276 |
| **SNRPB** | 1.521337 | 6.302245 | 9.549318 | 1.11E-19 | 1.25E-18 | 33.84510148 |
| **CLIC1** | 1.489832 | 4.838714 | 9.549115 | 1.11E-19 | 1.25E-18 | 33.84351153 |
| **POGK** | 1.268679 | 3.549713 | 9.54888 | 1.11E-19 | 1.25E-18 | 33.84167169 |
| **CSK** | 1.200058 | 4.849378 | 9.547964 | 1.12E-19 | 1.26E-18 | 33.83451522 |
| **DUSP11** | 0.963368 | 2.975505 | 9.54695 | 1.13E-19 | 1.27E-18 | 33.82658455 |
| **SCYL2** | 1.1118 | 2.596844 | 9.545384 | 1.14E-19 | 1.29E-18 | 33.8143493 |
| **LCP2** | 1.163939 | 2.583607 | 9.545094 | 1.15E-19 | 1.29E-18 | 33.81207888 |
| **FOXJ2** | 0.84549 | 2.375945 | 9.544657 | 1.15E-19 | 1.29E-18 | 33.80866365 |
| **GLA** | 1.064915 | 3.734536 | 9.544479 | 1.15E-19 | 1.29E-18 | 33.80727347 |
| **KIF7** | 0.833647 | 2.641022 | 9.544028 | 1.15E-19 | 1.30E-18 | 33.80374827 |
| **ALDH3A2** | 1.213222 | 4.317261 | 9.54311 | 1.16E-19 | 1.31E-18 | 33.79657057 |
| **MYH9** | 1.37333 | 4.33653 | 9.542654 | 1.17E-19 | 1.31E-18 | 33.79300819 |
| **CAPRIN1** | 1.315513 | 5.042202 | 9.54213 | 1.17E-19 | 1.32E-18 | 33.78891805 |
| **CNOT10** | 0.902952 | 3.219366 | 9.541359 | 1.18E-19 | 1.32E-18 | 33.78288874 |
| **NUFIP2** | 0.912023 | 2.526345 | 9.540593 | 1.19E-19 | 1.33E-18 | 33.77690286 |
| **PCDHB13** | 0.551815 | 0.877333 | 9.540396 | 1.19E-19 | 1.33E-18 | 33.77536864 |
| **FAM72A** | 0.683209 | 2.101863 | 9.540272 | 1.19E-19 | 1.33E-18 | 33.77439475 |
| **KANSL2** | 1.151682 | 3.521523 | 9.540136 | 1.19E-19 | 1.33E-18 | 33.77333071 |
| **ERO1L** | 0.989311 | 2.688041 | 9.539548 | 1.20E-19 | 1.34E-18 | 33.7687414 |
| **PDF** | 0.926802 | 3.447952 | 9.539432 | 1.20E-19 | 1.34E-18 | 33.76783128 |
| **ATL3** | 0.923873 | 2.567098 | 9.537618 | 1.22E-19 | 1.36E-18 | 33.75366166 |
| **EIF3M** | 1.561419 | 5.876075 | 9.537464 | 1.22E-19 | 1.36E-18 | 33.75245879 |
| **CHERP** | 1.27184 | 4.46833 | 9.537426 | 1.22E-19 | 1.36E-18 | 33.7521572 |
| **REPIN1** | 1.412501 | 5.992035 | 9.536889 | 1.22E-19 | 1.36E-18 | 33.74796938 |
| **MECP2** | 0.91156 | 3.32426 | 9.536817 | 1.22E-19 | 1.37E-18 | 33.74739964 |
| **CKLF** | 1.47423 | 5.479443 | 9.536401 | 1.23E-19 | 1.37E-18 | 33.74415197 |
| **OSTM1** | 1.008037 | 3.234496 | 9.535625 | 1.23E-19 | 1.38E-18 | 33.7380887 |
| **RP11-332H14.2** | 0.623421 | 1.321636 | 9.534473 | 1.25E-19 | 1.39E-18 | 33.72909522 |
| **NFYC** | 1.152158 | 4.202131 | 9.534244 | 1.25E-19 | 1.39E-18 | 33.7273079 |
| **RCAN1** | 1.412984 | 4.686589 | 9.534099 | 1.25E-19 | 1.39E-18 | 33.72617069 |
| **TMEM199** | 1.261236 | 3.949508 | 9.533998 | 1.25E-19 | 1.39E-18 | 33.7253844 |
| **AIDA** | 1.145688 | 3.766822 | 9.533926 | 1.25E-19 | 1.39E-18 | 33.72481837 |
| **TRMT2B** | 0.707559 | 1.840479 | 9.533545 | 1.25E-19 | 1.40E-18 | 33.72184484 |
| **CXorf38** | 0.945673 | 1.717604 | 9.533178 | 1.26E-19 | 1.40E-18 | 33.71897922 |
| **CCDC90B** | 1.243898 | 4.538139 | 9.533002 | 1.26E-19 | 1.40E-18 | 33.71760274 |
| **BAHD1** | 0.956356 | 2.94093 | 9.532999 | 1.26E-19 | 1.40E-18 | 33.71758142 |
| **FKBP14** | 0.937413 | 1.949164 | 9.532836 | 1.26E-19 | 1.40E-18 | 33.7163121 |
| **PSPH** | 1.029257 | 3.31168 | 9.53281 | 1.26E-19 | 1.40E-18 | 33.71610845 |
| **G2E3** | 0.808139 | 1.743925 | 9.532667 | 1.26E-19 | 1.40E-18 | 33.71499329 |
| **ARHGEF18** | 1.015574 | 3.718757 | 9.532259 | 1.27E-19 | 1.41E-18 | 33.71180675 |
| **NR2F2** | 1.200371 | 2.23986 | 9.532171 | 1.27E-19 | 1.41E-18 | 33.71111462 |
| **TRIM27** | 1.130223 | 4.442899 | 9.531583 | 1.27E-19 | 1.41E-18 | 33.70652687 |
| **IER5** | 1.257758 | 3.96893 | 9.531548 | 1.27E-19 | 1.41E-18 | 33.70625107 |
| **MFHAS1** | 0.943465 | 2.334029 | 9.531144 | 1.28E-19 | 1.42E-18 | 33.70309615 |
| **RBM33** | 0.955616 | 3.594112 | 9.530589 | 1.28E-19 | 1.42E-18 | 33.698759 |
| **TMEM131** | 0.984081 | 2.75115 | 9.530514 | 1.29E-19 | 1.42E-18 | 33.69817414 |
| **SMARCA5** | 1.175522 | 3.572471 | 9.530278 | 1.29E-19 | 1.42E-18 | 33.69633529 |
| **HNRNPU** | 1.428019 | 5.986832 | 9.530054 | 1.29E-19 | 1.42E-18 | 33.69458336 |
| **GALNT2** | 1.228479 | 4.319324 | 9.529721 | 1.29E-19 | 1.43E-18 | 33.69198573 |
| **IARS** | 1.218096 | 3.894864 | 9.529533 | 1.30E-19 | 1.43E-18 | 33.6905172 |
| **RANBP10** | 0.892122 | 2.556571 | 9.529209 | 1.30E-19 | 1.43E-18 | 33.68798572 |
| **IGF2BP3** | 0.901347 | 0.651278 | 9.527937 | 1.31E-19 | 1.45E-18 | 33.67805853 |
| **FOXN2** | 1.059393 | 2.218423 | 9.526115 | 1.33E-19 | 1.47E-18 | 33.66383038 |
| **CDC27** | 1.091135 | 3.189343 | 9.525391 | 1.34E-19 | 1.47E-18 | 33.65818327 |
| **E2F4** | 1.111848 | 4.579182 | 9.525014 | 1.34E-19 | 1.48E-18 | 33.65523586 |
| **BTN3A3** | 1.166054 | 2.896939 | 9.523092 | 1.36E-19 | 1.50E-18 | 33.64023817 |
| **PGAM5** | 1.070813 | 3.361177 | 9.522846 | 1.37E-19 | 1.50E-18 | 33.63831656 |
| **TTC23** | 0.810136 | 2.584873 | 9.520749 | 1.39E-19 | 1.53E-18 | 33.62195359 |
| **INTS6** | 0.893559 | 2.460659 | 9.520729 | 1.39E-19 | 1.53E-18 | 33.62179413 |
| **TMEM33** | 1.009063 | 2.822405 | 9.520637 | 1.39E-19 | 1.53E-18 | 33.62107555 |
| **ASB6** | 0.936222 | 3.461775 | 9.520538 | 1.39E-19 | 1.53E-18 | 33.62030523 |
| **SPRYD4** | 0.652412 | 2.049307 | 9.520197 | 1.39E-19 | 1.53E-18 | 33.61764352 |
| **MFAP3** | 1.05481 | 2.365205 | 9.519643 | 1.40E-19 | 1.54E-18 | 33.61332249 |
| **PRDM4** | 1.012275 | 2.633746 | 9.518627 | 1.41E-19 | 1.55E-18 | 33.60539804 |
| **CENPC** | 0.846182 | 1.68548 | 9.518385 | 1.41E-19 | 1.55E-18 | 33.60350659 |
| **CRAMP1L** | 0.994968 | 2.915175 | 9.517723 | 1.42E-19 | 1.56E-18 | 33.59833987 |
| **AFAP1** | 1.134592 | 2.897246 | 9.517604 | 1.42E-19 | 1.56E-18 | 33.59741161 |
| **VANGL2** | 1.246267 | 3.454665 | 9.516513 | 1.44E-19 | 1.57E-18 | 33.58890236 |
| **DDX28** | 0.703537 | 2.54724 | 9.516455 | 1.44E-19 | 1.57E-18 | 33.58845143 |
| **AXIN1** | 0.999823 | 3.678307 | 9.516423 | 1.44E-19 | 1.57E-18 | 33.58820014 |
| **ZNF10** | 0.898776 | 2.125269 | 9.515988 | 1.44E-19 | 1.58E-18 | 33.58480521 |
| **SUSD1** | 0.833534 | 2.267975 | 9.51563 | 1.45E-19 | 1.58E-18 | 33.58201468 |
| **RHOJ** | 1.052622 | 2.377139 | 9.515451 | 1.45E-19 | 1.58E-18 | 33.5806167 |
| **AC009948.5** | 1.014574 | 2.697389 | 9.514819 | 1.46E-19 | 1.59E-18 | 33.57568601 |
| **MAML2** | 1.166549 | 2.613985 | 9.514812 | 1.46E-19 | 1.59E-18 | 33.5756378 |
| **EHMT1** | 1.105256 | 4.332266 | 9.513813 | 1.47E-19 | 1.60E-18 | 33.56784414 |
| **FEM1C** | 0.969939 | 2.166591 | 9.513499 | 1.47E-19 | 1.60E-18 | 33.56539432 |
| **ZNF791** | 0.864236 | 2.101959 | 9.513039 | 1.48E-19 | 1.61E-18 | 33.56180733 |
| **NFKB1** | 0.929903 | 2.372163 | 9.509783 | 1.51E-19 | 1.65E-18 | 33.53641585 |
| **LPCAT1** | 1.317558 | 4.712708 | 9.509414 | 1.52E-19 | 1.65E-18 | 33.53353376 |
| **GYS1** | 0.980534 | 3.442558 | 9.509026 | 1.52E-19 | 1.66E-18 | 33.53050852 |
| **GABPA** | 1.028476 | 2.512177 | 9.508868 | 1.53E-19 | 1.66E-18 | 33.52928263 |
| **H3F3B** | 1.879439 | 8.848224 | 9.50878 | 1.53E-19 | 1.66E-18 | 33.52859229 |
| **YEATS2** | 1.019435 | 3.356273 | 9.50844 | 1.53E-19 | 1.66E-18 | 33.52594203 |
| **FBXL12** | 1.035919 | 3.188187 | 9.507916 | 1.54E-19 | 1.67E-18 | 33.52186101 |
| **HMGN4** | 1.519434 | 3.378281 | 9.50774 | 1.54E-19 | 1.67E-18 | 33.52048551 |
| **SETD5** | 1.51663 | 4.477007 | 9.507104 | 1.55E-19 | 1.68E-18 | 33.51552759 |
| **ARL5A** | 1.235723 | 3.279241 | 9.50681 | 1.55E-19 | 1.68E-18 | 33.51323681 |
| **ZNF707** | 0.900337 | 3.198699 | 9.506631 | 1.55E-19 | 1.68E-18 | 33.51184278 |
| **TRIB3** | 0.919694 | 2.320178 | 9.506179 | 1.56E-19 | 1.69E-18 | 33.50831223 |
| **AKT1** | 1.348495 | 5.558285 | 9.505751 | 1.56E-19 | 1.69E-18 | 33.50498135 |
| **PROSER1** | 0.911397 | 2.734736 | 9.504937 | 1.57E-19 | 1.70E-18 | 33.49863004 |
| **HNRNPA1P16** | 0.564758 | 0.942654 | 9.504392 | 1.58E-19 | 1.71E-18 | 33.49438188 |
| **SMEK2** | 1.187585 | 3.637806 | 9.503521 | 1.59E-19 | 1.72E-18 | 33.48759498 |
| **ZNF180** | 0.648826 | 1.293667 | 9.502607 | 1.60E-19 | 1.73E-18 | 33.48047166 |
| **CTBP1-AS2** | 1.174973 | 2.927597 | 9.501285 | 1.62E-19 | 1.75E-18 | 33.47017083 |
| **POC5** | 0.805852 | 2.092719 | 9.50097 | 1.62E-19 | 1.75E-18 | 33.46771594 |
| **TMEM117** | 0.755806 | 2.059577 | 9.500281 | 1.63E-19 | 1.76E-18 | 33.46234293 |
| **CCRL2** | 0.581529 | 1.085418 | 9.499355 | 1.64E-19 | 1.77E-18 | 33.45513167 |
| **TMEM173** | 1.071907 | 3.227742 | 9.499163 | 1.65E-19 | 1.78E-18 | 33.45363426 |
| **HNRNPL** | 1.556635 | 6.230659 | 9.498949 | 1.65E-19 | 1.78E-18 | 33.4519703 |
| **RP11-98D18.9** | 0.798567 | 1.896346 | 9.4969 | 1.68E-19 | 1.81E-18 | 33.43599935 |
| **FBXO28** | 0.928037 | 2.500591 | 9.495531 | 1.70E-19 | 1.83E-18 | 33.42534144 |
| **OLFML2A** | 0.770699 | 1.121569 | 9.495041 | 1.70E-19 | 1.83E-18 | 33.42152284 |
| **SMURF2** | 0.994382 | 2.438247 | 9.493373 | 1.72E-19 | 1.85E-18 | 33.40852852 |
| **NCK2** | 1.344885 | 4.012111 | 9.492324 | 1.74E-19 | 1.87E-18 | 33.40035569 |
| **TLE3** | 1.096479 | 4.380965 | 9.491969 | 1.74E-19 | 1.87E-18 | 33.3975964 |
| **CDC25B** | 1.236234 | 4.629873 | 9.491614 | 1.75E-19 | 1.88E-18 | 33.39483051 |
| **HBEGF** | 0.932924 | 2.799462 | 9.490234 | 1.77E-19 | 1.90E-18 | 33.38407935 |
| **ZNF354C** | 0.814799 | 1.328406 | 9.489657 | 1.78E-19 | 1.90E-18 | 33.37959046 |
| **LTV1** | 0.994062 | 3.007927 | 9.489067 | 1.78E-19 | 1.91E-18 | 33.37499312 |
| **ZNF311** | 0.603727 | 1.035461 | 9.48821 | 1.80E-19 | 1.92E-18 | 33.36832263 |
| **DNAJB11** | 1.380461 | 5.109387 | 9.488076 | 1.80E-19 | 1.93E-18 | 33.36727581 |
| **CASP8AP2** | 0.764435 | 2.033465 | 9.487986 | 1.80E-19 | 1.93E-18 | 33.3665763 |
| **NLN** | 0.925083 | 2.39165 | 9.487354 | 1.81E-19 | 1.93E-18 | 33.36165374 |
| **GOLGA1** | 0.8606 | 2.745029 | 9.487271 | 1.81E-19 | 1.93E-18 | 33.36100786 |
| **EDEM2** | 0.902963 | 3.237568 | 9.486169 | 1.83E-19 | 1.95E-18 | 33.3524292 |
| **IQCB1** | 0.985618 | 2.664038 | 9.484578 | 1.85E-19 | 1.97E-18 | 33.34004708 |
| **SRSF9** | 1.633118 | 6.219498 | 9.484048 | 1.86E-19 | 1.98E-18 | 33.33591996 |
| **ZNF41** | 0.658845 | 1.560049 | 9.483585 | 1.86E-19 | 1.99E-18 | 33.33231981 |
| **SMN1** | 1.254922 | 3.588865 | 9.483232 | 1.87E-19 | 1.99E-18 | 33.32957202 |
| **RMDN1** | 1.090901 | 4.025525 | 9.482118 | 1.88E-19 | 2.01E-18 | 33.32089591 |
| **PARN** | 0.930062 | 2.998729 | 9.480985 | 1.90E-19 | 2.03E-18 | 33.31207763 |
| **UTP20** | 0.61338 | 1.242422 | 9.480399 | 1.91E-19 | 2.03E-18 | 33.30752057 |
| **FCGR2A** | 1.23198 | 2.798823 | 9.480191 | 1.91E-19 | 2.04E-18 | 33.30590295 |
| **ACBD3** | 1.169826 | 2.888732 | 9.479704 | 1.92E-19 | 2.04E-18 | 33.30211628 |
| **SYNRG** | 0.938638 | 2.696369 | 9.478803 | 1.93E-19 | 2.06E-18 | 33.29510231 |
| **TM7SF3** | 1.411065 | 3.878351 | 9.477484 | 1.95E-19 | 2.08E-18 | 33.2848426 |
| **TMEM123** | 1.362591 | 4.328954 | 9.477081 | 1.96E-19 | 2.08E-18 | 33.28169989 |
| **GSR** | 1.019778 | 3.260846 | 9.476653 | 1.97E-19 | 2.09E-18 | 33.2783739 |
| **LAMB1** | 1.250378 | 3.190925 | 9.476564 | 1.97E-19 | 2.09E-18 | 33.27768264 |
| **MYEF2** | 1.384409 | 4.354478 | 9.475205 | 1.99E-19 | 2.11E-18 | 33.26711061 |
| **TAF1B** | 0.87504 | 1.887309 | 9.475205 | 1.99E-19 | 2.11E-18 | 33.26710903 |
| **IDE** | 0.68766 | 2.007067 | 9.475006 | 1.99E-19 | 2.11E-18 | 33.26556066 |
| **EIF2S3** | 1.296638 | 4.785867 | 9.4749 | 1.99E-19 | 2.11E-18 | 33.26473402 |
| **CFI** | 1.422663 | 2.379499 | 9.473883 | 2.01E-19 | 2.13E-18 | 33.25682338 |
| **ZBTB40** | 0.773969 | 2.019879 | 9.473708 | 2.01E-19 | 2.13E-18 | 33.25546206 |
| **ZNF319** | 0.901625 | 2.466942 | 9.473171 | 2.02E-19 | 2.14E-18 | 33.25128346 |
| **SNRNP40** | 1.112387 | 3.758651 | 9.472457 | 2.03E-19 | 2.15E-18 | 33.24573305 |
| **RP11-420L9.5** | 0.870005 | 1.834091 | 9.472293 | 2.04E-19 | 2.15E-18 | 33.24445793 |
| **RBM23** | 1.057956 | 4.505891 | 9.472048 | 2.04E-19 | 2.16E-18 | 33.24255448 |
| **PPP4R2** | 1.109566 | 2.901829 | 9.471879 | 2.04E-19 | 2.16E-18 | 33.24124019 |
| **CASK** | 1.163009 | 3.371828 | 9.471059 | 2.06E-19 | 2.17E-18 | 33.23486217 |
| **MIER3** | 0.902775 | 2.09414 | 9.470505 | 2.07E-19 | 2.18E-18 | 33.2305514 |
| **THUMPD3** | 1.069286 | 4.065291 | 9.470416 | 2.07E-19 | 2.18E-18 | 33.22985672 |
| **LIMS1** | 1.352317 | 3.216222 | 9.470261 | 2.07E-19 | 2.18E-18 | 33.22865381 |
| **CTD-3018O17.3** | 1.01785 | 2.932617 | 9.470121 | 2.07E-19 | 2.18E-18 | 33.22756883 |
| **DNAJC11** | 1.055332 | 3.773064 | 9.469803 | 2.08E-19 | 2.19E-18 | 33.22509611 |
| **ALG10** | 0.533078 | 0.754617 | 9.469752 | 2.08E-19 | 2.19E-18 | 33.22469454 |
| **APEX1** | 1.597619 | 6.49103 | 9.468668 | 2.10E-19 | 2.21E-18 | 33.21626596 |
| **UBE2D3** | 1.553334 | 5.686765 | 9.468103 | 2.10E-19 | 2.22E-18 | 33.21187244 |
| **RFX7** | 0.860268 | 1.902843 | 9.467964 | 2.11E-19 | 2.22E-18 | 33.21079579 |
| **GORASP2** | 1.257276 | 4.117809 | 9.467395 | 2.12E-19 | 2.23E-18 | 33.20636656 |
| **ERN1** | 0.617975 | 1.312759 | 9.466676 | 2.13E-19 | 2.24E-18 | 33.20077586 |
| **UBR7** | 1.058655 | 3.393378 | 9.46625 | 2.14E-19 | 2.24E-18 | 33.19746533 |
| **RPTOR** | 0.914045 | 3.48192 | 9.465858 | 2.14E-19 | 2.25E-18 | 33.19441615 |
| **STAG1** | 1.079922 | 2.172439 | 9.465808 | 2.14E-19 | 2.25E-18 | 33.19403284 |
| **MESDC1** | 1.108843 | 2.850632 | 9.465554 | 2.15E-19 | 2.25E-18 | 33.19205723 |
| **DAB2** | 1.080134 | 2.767381 | 9.465427 | 2.15E-19 | 2.25E-18 | 33.19106775 |
| **DDX27** | 1.036814 | 3.832451 | 9.464504 | 2.17E-19 | 2.27E-18 | 33.1838901 |
| **IFT52** | 1.088484 | 4.133239 | 9.464152 | 2.17E-19 | 2.27E-18 | 33.18115812 |
| **DRAM2** | 1.206839 | 3.690512 | 9.463028 | 2.19E-19 | 2.29E-18 | 33.17241896 |
| **ZKSCAN8** | 1.027034 | 2.299476 | 9.46209 | 2.21E-19 | 2.31E-18 | 33.16512892 |
| **ZNF532** | 1.435825 | 4.016227 | 9.461938 | 2.21E-19 | 2.31E-18 | 33.16394685 |
| **ZNF626** | 0.869265 | 1.93021 | 9.461134 | 2.22E-19 | 2.33E-18 | 33.15769969 |
| **DHX29** | 0.870798 | 2.539461 | 9.460428 | 2.24E-19 | 2.34E-18 | 33.15221661 |
| **SF3B3** | 1.389011 | 5.705757 | 9.458598 | 2.27E-19 | 2.37E-18 | 33.13799564 |
| **SOCS6** | 1.119453 | 2.690877 | 9.458381 | 2.27E-19 | 2.37E-18 | 33.13630784 |
| **UTP3** | 0.992454 | 3.283437 | 9.458079 | 2.28E-19 | 2.38E-18 | 33.13395884 |
| **MLXIP** | 0.877532 | 3.448597 | 9.457568 | 2.29E-19 | 2.39E-18 | 33.12998743 |
| **ZNF12** | 0.849656 | 2.325204 | 9.456342 | 2.31E-19 | 2.41E-18 | 33.12046397 |
| **PIGF** | 1.080057 | 3.565694 | 9.456235 | 2.31E-19 | 2.41E-18 | 33.11963462 |
| **CEP41** | 1.001063 | 2.543344 | 9.455794 | 2.32E-19 | 2.42E-18 | 33.11620389 |
| **TMEM86A** | 0.719425 | 2.123922 | 9.455366 | 2.33E-19 | 2.42E-18 | 33.11287891 |
| **CISH** | 0.559268 | 0.8361 | 9.45421 | 2.35E-19 | 2.44E-18 | 33.10390352 |
| **SMC3** | 1.309115 | 3.227284 | 9.453837 | 2.36E-19 | 2.45E-18 | 33.10100663 |
| **MPHOSPH10** | 1.03653 | 3.380335 | 9.453772 | 2.36E-19 | 2.45E-18 | 33.10049996 |
| **BMP8B** | 0.945551 | 1.473032 | 9.450939 | 2.41E-19 | 2.51E-18 | 33.07849706 |
| **MAP3K3** | 0.978854 | 2.912462 | 9.450154 | 2.42E-19 | 2.52E-18 | 33.07240043 |
| **KRBOX4** | 0.926235 | 2.92911 | 9.448752 | 2.45E-19 | 2.55E-18 | 33.06150799 |
| **CABLES2** | 0.64197 | 1.786356 | 9.448293 | 2.46E-19 | 2.55E-18 | 33.0579493 |
| **SLC35F6** | 0.890425 | 3.309156 | 9.447796 | 2.47E-19 | 2.56E-18 | 33.05408736 |
| **FIGNL1** | 0.882781 | 1.947744 | 9.44748 | 2.48E-19 | 2.57E-18 | 33.05163158 |
| **TBC1D25** | 0.925912 | 2.981602 | 9.446915 | 2.49E-19 | 2.58E-18 | 33.04724808 |
| **SETDB1** | 1.059741 | 4.088212 | 9.445915 | 2.51E-19 | 2.60E-18 | 33.03948556 |
| **ST3GAL2** | 0.922067 | 3.532639 | 9.444366 | 2.54E-19 | 2.63E-18 | 33.02745815 |
| **ATP13A3** | 1.168984 | 3.154908 | 9.443894 | 2.55E-19 | 2.64E-18 | 33.02379731 |
| **SRR** | 1.166437 | 3.556588 | 9.443791 | 2.55E-19 | 2.64E-18 | 33.02299239 |
| **HNRNPK** | 1.666835 | 6.965067 | 9.443487 | 2.55E-19 | 2.64E-18 | 33.02063742 |
| **FRMD6** | 0.946312 | 2.018779 | 9.442357 | 2.58E-19 | 2.67E-18 | 33.01186267 |
| **RRAGC** | 1.006165 | 3.166234 | 9.44136 | 2.60E-19 | 2.69E-18 | 33.00412654 |
| **ADAM10** | 1.168636 | 3.459837 | 9.441175 | 2.60E-19 | 2.69E-18 | 33.00268848 |
| **EMC8** | 1.041099 | 3.878795 | 9.441071 | 2.60E-19 | 2.69E-18 | 33.00188059 |
| **SLCO5A1** | 0.543374 | 0.73889 | 9.44099 | 2.61E-19 | 2.69E-18 | 33.0012549 |
| **AKIRIN2** | 1.401524 | 4.390781 | 9.439921 | 2.63E-19 | 2.71E-18 | 32.99295886 |
| **DDX50** | 0.976967 | 3.358259 | 9.439204 | 2.64E-19 | 2.73E-18 | 32.98739431 |
| **KDM5C** | 1.151858 | 4.137561 | 9.438407 | 2.66E-19 | 2.74E-18 | 32.9812094 |
| **ZFAND6** | 1.272907 | 5.027057 | 9.438268 | 2.66E-19 | 2.74E-18 | 32.98012962 |
| **COPS7B** | 1.087981 | 4.049985 | 9.438131 | 2.66E-19 | 2.74E-18 | 32.97906478 |
| **MTERF** | 0.861831 | 2.277717 | 9.437703 | 2.67E-19 | 2.75E-18 | 32.97574845 |
| **SEPHS2** | 1.059683 | 3.994199 | 9.437375 | 2.68E-19 | 2.76E-18 | 32.97319977 |
| **ZNF17** | 0.738613 | 1.53583 | 9.437328 | 2.68E-19 | 2.76E-18 | 32.97283743 |
| **PHF12** | 1.287325 | 3.77867 | 9.437271 | 2.68E-19 | 2.76E-18 | 32.97239793 |
| **CAPZA1** | 1.268436 | 3.59655 | 9.437021 | 2.69E-19 | 2.76E-18 | 32.9704538 |
| **SNX1** | 1.407875 | 4.866814 | 9.436279 | 2.70E-19 | 2.78E-18 | 32.96469574 |
| **SPTSSA** | 1.185896 | 3.916719 | 9.435831 | 2.71E-19 | 2.79E-18 | 32.96121848 |
| **CFLAR** | 0.982555 | 3.598231 | 9.434997 | 2.73E-19 | 2.80E-18 | 32.95475115 |
| **RGP1** | 0.999949 | 2.648361 | 9.433857 | 2.76E-19 | 2.83E-18 | 32.94590816 |
| **UBXN2B** | 1.029055 | 2.984391 | 9.433245 | 2.77E-19 | 2.84E-18 | 32.94116307 |
| **DERL2** | 1.120921 | 4.348929 | 9.43229 | 2.79E-19 | 2.86E-18 | 32.9337563 |
| **MAP7D3** | 0.62051 | 1.367666 | 9.431879 | 2.80E-19 | 2.87E-18 | 32.93056613 |
| **YTHDF1** | 1.11458 | 4.172536 | 9.431227 | 2.81E-19 | 2.88E-18 | 32.92550813 |
| **FZR1** | 1.070945 | 4.387074 | 9.430583 | 2.83E-19 | 2.90E-18 | 32.92051539 |
| **SRC** | 1.149557 | 3.649161 | 9.430296 | 2.83E-19 | 2.90E-18 | 32.91829057 |
| **DBN1** | 1.342773 | 5.8621 | 9.429715 | 2.85E-19 | 2.91E-18 | 32.91378234 |
| **IRF2BP2** | 1.381379 | 4.429342 | 9.428826 | 2.87E-19 | 2.93E-18 | 32.90688577 |
| **VCP** | 1.257647 | 5.429932 | 9.428732 | 2.87E-19 | 2.93E-18 | 32.90615753 |
| **ILF3** | 1.575196 | 6.511843 | 9.428251 | 2.88E-19 | 2.94E-18 | 32.90242621 |
| **RAN** | 1.708442 | 6.620291 | 9.428147 | 2.88E-19 | 2.94E-18 | 32.90162144 |
| **RGS19** | 1.112408 | 3.649153 | 9.427573 | 2.90E-19 | 2.96E-18 | 32.89717044 |
| **ZNF555** | 0.626971 | 1.251506 | 9.427551 | 2.90E-19 | 2.96E-18 | 32.89700014 |
| **ZFYVE1** | 0.946507 | 3.107132 | 9.427069 | 2.91E-19 | 2.97E-18 | 32.89326375 |
| **CELF1** | 1.19953 | 3.793968 | 9.426884 | 2.91E-19 | 2.97E-18 | 32.89182828 |
| **FAM49B** | 1.296941 | 4.519004 | 9.425488 | 2.94E-19 | 3.00E-18 | 32.88100507 |
| **BCL9** | 0.966164 | 2.276005 | 9.425462 | 2.94E-19 | 3.00E-18 | 32.88080782 |
| **CSNK1A1** | 1.529708 | 4.547739 | 9.425079 | 2.95E-19 | 3.01E-18 | 32.87783141 |
| **NBPF1** | 1.206918 | 3.649406 | 9.42463 | 2.96E-19 | 3.02E-18 | 32.87435151 |
| **SLC38A2** | 1.284114 | 4.006666 | 9.424532 | 2.97E-19 | 3.02E-18 | 32.87359596 |
| **ZC3H8** | 0.810745 | 2.315587 | 9.424234 | 2.97E-19 | 3.02E-18 | 32.87128627 |
| **MED30** | 1.068866 | 3.275592 | 9.42377 | 2.98E-19 | 3.03E-18 | 32.86768448 |
| **TMEM2** | 0.991975 | 2.068487 | 9.423343 | 2.99E-19 | 3.04E-18 | 32.86437438 |
| **YEATS4** | 1.115864 | 3.158154 | 9.422751 | 3.01E-19 | 3.05E-18 | 32.85978568 |
| **PIGC** | 1.012969 | 3.471324 | 9.422351 | 3.02E-19 | 3.06E-18 | 32.85669189 |
| **DTYMK** | 1.268253 | 4.638692 | 9.422272 | 3.02E-19 | 3.06E-18 | 32.85607242 |
| **SNX4** | 1.285336 | 3.452266 | 9.422229 | 3.02E-19 | 3.06E-18 | 32.85573967 |
| **LBX2-AS1** | 0.563587 | 0.993846 | 9.421884 | 3.03E-19 | 3.07E-18 | 32.85307055 |
| **WDR5** | 1.044841 | 3.974115 | 9.421617 | 3.03E-19 | 3.08E-18 | 32.85100209 |
| **CCT6A** | 1.527622 | 5.748712 | 9.421553 | 3.04E-19 | 3.08E-18 | 32.85050586 |
| **PPP2R2A** | 1.207144 | 3.720005 | 9.420174 | 3.07E-19 | 3.11E-18 | 32.83981573 |
| **ZNF454** | 0.562704 | 1.039171 | 9.420002 | 3.07E-19 | 3.11E-18 | 32.8384804 |
| **SLC38A7** | 0.817293 | 3.121705 | 9.419326 | 3.09E-19 | 3.13E-18 | 32.83324673 |
| **MBD2** | 0.934626 | 2.835623 | 9.419211 | 3.09E-19 | 3.13E-18 | 32.83234989 |
| **PAIP1** | 1.345788 | 4.540073 | 9.419199 | 3.09E-19 | 3.13E-18 | 32.83225844 |
| **GPX8** | 0.743254 | 0.765203 | 9.418399 | 3.11E-19 | 3.14E-18 | 32.82605739 |
| **WIZ** | 1.304189 | 4.288751 | 9.416955 | 3.15E-19 | 3.18E-18 | 32.81486948 |
| **ZNF347** | 0.644026 | 1.090198 | 9.416712 | 3.15E-19 | 3.18E-18 | 32.81298792 |
| **DYNLT1** | 1.632281 | 6.245619 | 9.414369 | 3.21E-19 | 3.24E-18 | 32.79483361 |
| **ZBED3** | 1.063563 | 2.990377 | 9.41333 | 3.24E-19 | 3.27E-18 | 32.78679025 |
| **NSD1** | 0.865223 | 2.446972 | 9.413129 | 3.24E-19 | 3.27E-18 | 32.78523097 |
| **XPOT** | 1.23814 | 3.671697 | 9.412502 | 3.26E-19 | 3.28E-18 | 32.78037336 |
| **UBE2N** | 1.417178 | 4.628436 | 9.412106 | 3.27E-19 | 3.29E-18 | 32.77730641 |
| **MIR4453** | 0.836717 | 1.524194 | 9.41111 | 3.30E-19 | 3.32E-18 | 32.7695879 |
| **SCAMP4** | 1.250588 | 4.714445 | 9.410401 | 3.31E-19 | 3.33E-18 | 32.76410193 |
| **USP21** | 1.233634 | 4.029775 | 9.409943 | 3.33E-19 | 3.35E-18 | 32.76055453 |
| **LATS2** | 0.61723 | 0.862917 | 9.40968 | 3.33E-19 | 3.35E-18 | 32.75851325 |
| **WHSC1L1** | 1.161339 | 3.934488 | 9.407735 | 3.38E-19 | 3.40E-18 | 32.74345334 |
| **CEBPG** | 1.01435 | 3.18332 | 9.407321 | 3.40E-19 | 3.41E-18 | 32.74025007 |
| **CSMD2** | 0.977647 | 1.726561 | 9.407256 | 3.40E-19 | 3.41E-18 | 32.73974529 |
| **10-Sep** | 1.071076 | 2.959422 | 9.407244 | 3.40E-19 | 3.41E-18 | 32.73965517 |
| **RBM38** | 1.119727 | 3.961394 | 9.406597 | 3.41E-19 | 3.43E-18 | 32.7346456 |
| **RP5-1136G13.2** | 0.558796 | 1.577661 | 9.406558 | 3.42E-19 | 3.43E-18 | 32.7343425 |
| **BCL9L** | 1.227752 | 2.494543 | 9.406461 | 3.42E-19 | 3.43E-18 | 32.73359222 |
| **GAR1** | 1.211617 | 4.438004 | 9.405857 | 3.43E-19 | 3.44E-18 | 32.7289121 |
| **CNOT7** | 1.225732 | 3.675171 | 9.404854 | 3.46E-19 | 3.47E-18 | 32.72114807 |
| **C1orf226** | 1.078589 | 2.264928 | 9.404654 | 3.47E-19 | 3.47E-18 | 32.71959785 |
| **CDR2L** | 0.953302 | 2.917733 | 9.403768 | 3.49E-19 | 3.49E-18 | 32.71274517 |
| **ZNF674-AS1** | 0.639121 | 1.609342 | 9.403622 | 3.50E-19 | 3.50E-18 | 32.71161223 |
| **DLGAP4** | 1.21624 | 4.803006 | 9.40357 | 3.50E-19 | 3.50E-18 | 32.71120931 |
| **RP11-422P24.11** | 0.532303 | 1.28905 | 9.402865 | 3.52E-19 | 3.51E-18 | 32.70575432 |
| **RPAP1** | 0.877649 | 3.411277 | 9.402856 | 3.52E-19 | 3.51E-18 | 32.70568533 |
| **SLC10A3** | 0.811599 | 2.58985 | 9.401929 | 3.54E-19 | 3.54E-18 | 32.69850788 |
| **BRD3** | 1.270584 | 3.21928 | 9.401448 | 3.56E-19 | 3.55E-18 | 32.69478489 |
| **BAG5** | 0.937375 | 2.899167 | 9.400383 | 3.59E-19 | 3.58E-18 | 32.6865394 |
| **VPS37A** | 1.055717 | 3.048448 | 9.400186 | 3.59E-19 | 3.58E-18 | 32.68501638 |
| **IGFBP2** | 1.925202 | 5.05056 | 9.399927 | 3.60E-19 | 3.59E-18 | 32.68301422 |
| **ZBTB46** | 0.678884 | 2.258791 | 9.39955 | 3.61E-19 | 3.60E-18 | 32.68009156 |
| **PTN** | 1.896147 | 7.017233 | 9.399121 | 3.62E-19 | 3.61E-18 | 32.67677749 |
| **CGGBP1** | 1.254897 | 3.720946 | 9.3991 | 3.62E-19 | 3.61E-18 | 32.67661222 |
| **WIPF1** | 1.138505 | 3.438488 | 9.398678 | 3.63E-19 | 3.62E-18 | 32.67334939 |
| **MTHFD2L** | 0.896838 | 3.02684 | 9.398535 | 3.64E-19 | 3.62E-18 | 32.67223779 |
| **IGSF9B** | 1.15145 | 2.926273 | 9.398525 | 3.64E-19 | 3.62E-18 | 32.67216222 |
| **ZNF69** | 0.598572 | 1.449235 | 9.397959 | 3.65E-19 | 3.63E-18 | 32.66778168 |
| **FTSJ3** | 1.049949 | 4.189846 | 9.397471 | 3.67E-19 | 3.64E-18 | 32.66400698 |
| **SLBP** | 1.160186 | 4.038518 | 9.397227 | 3.68E-19 | 3.65E-18 | 32.66211727 |
| **KCTD11** | 0.806197 | 2.506557 | 9.396842 | 3.69E-19 | 3.66E-18 | 32.65913566 |
| **VPS37C** | 0.843517 | 3.290036 | 9.396222 | 3.70E-19 | 3.67E-18 | 32.65434048 |
| **HAUS2** | 0.998286 | 2.526737 | 9.395993 | 3.71E-19 | 3.68E-18 | 32.65257224 |
| **OAS2** | 0.881666 | 1.78565 | 9.395764 | 3.72E-19 | 3.68E-18 | 32.65080014 |
| **CEP152** | 0.552608 | 0.761732 | 9.395132 | 3.74E-19 | 3.70E-18 | 32.64590653 |
| **TGFBI** | 1.579522 | 3.867639 | 9.39392 | 3.77E-19 | 3.73E-18 | 32.63653105 |
| **ZNF681** | 0.65947 | 1.150493 | 9.393914 | 3.77E-19 | 3.73E-18 | 32.6364844 |
| **HNRNPD** | 1.690964 | 6.538789 | 9.393822 | 3.77E-19 | 3.73E-18 | 32.635771 |
| **SRSF1** | 1.48172 | 5.507387 | 9.39345 | 3.79E-19 | 3.74E-18 | 32.63289992 |
| **ZCCHC14** | 1.020095 | 2.977329 | 9.393102 | 3.80E-19 | 3.75E-18 | 32.63020784 |
| **UGCG** | 0.948691 | 2.308013 | 9.3917 | 3.84E-19 | 3.79E-18 | 32.61936231 |
| **ETNK1** | 0.965385 | 2.869306 | 9.391069 | 3.86E-19 | 3.81E-18 | 32.61447585 |
| **WNK3** | 0.574634 | 1.095074 | 9.390599 | 3.87E-19 | 3.82E-18 | 32.6108466 |
| **SALL1** | 1.378131 | 3.595518 | 9.389598 | 3.90E-19 | 3.85E-18 | 32.60310465 |
| **MSTO2P** | 0.847699 | 2.2077 | 9.389486 | 3.91E-19 | 3.85E-18 | 32.60223816 |
| **CMTM1** | 1.0831 | 1.912535 | 9.389442 | 3.91E-19 | 3.85E-18 | 32.60189453 |
| **PPP2R5E** | 0.99621 | 2.622006 | 9.389266 | 3.91E-19 | 3.86E-18 | 32.60053221 |
| **ZNF585A** | 0.825608 | 2.799753 | 9.3888 | 3.93E-19 | 3.87E-18 | 32.59692755 |
| **SYNE2** | 1.205297 | 3.529801 | 9.388789 | 3.93E-19 | 3.87E-18 | 32.59684932 |
| **NSUN3** | 0.734573 | 1.751864 | 9.388485 | 3.94E-19 | 3.87E-18 | 32.59449506 |
| **ETV3** | 0.928801 | 2.077706 | 9.388204 | 3.94E-19 | 3.88E-18 | 32.59231939 |
| **KCMF1** | 1.223362 | 3.245011 | 9.387974 | 3.95E-19 | 3.89E-18 | 32.59054271 |
| **PPP1CC** | 1.43544 | 5.119171 | 9.387234 | 3.97E-19 | 3.91E-18 | 32.58482382 |
| **MED13** | 0.922738 | 2.490073 | 9.387077 | 3.98E-19 | 3.91E-18 | 32.58361168 |
| **AREL1** | 1.003832 | 2.633309 | 9.384496 | 4.06E-19 | 3.99E-18 | 32.56365151 |
| **PPRC1** | 0.875867 | 3.217199 | 9.384352 | 4.07E-19 | 3.99E-18 | 32.56253965 |
| **MAVS** | 1.081515 | 2.586177 | 9.38404 | 4.08E-19 | 4.00E-18 | 32.56012631 |
| **TMEM169** | 0.922019 | 1.925675 | 9.383145 | 4.10E-19 | 4.03E-18 | 32.55320733 |
| **ZNF410** | 1.21447 | 3.377165 | 9.3827 | 4.12E-19 | 4.04E-18 | 32.54977024 |
| **HCCS** | 0.944977 | 2.798013 | 9.381255 | 4.17E-19 | 4.08E-18 | 32.53860336 |
| **EAF1** | 1.057073 | 2.683124 | 9.381056 | 4.17E-19 | 4.09E-18 | 32.5370623 |
| **TRMT44** | 0.768706 | 2.523699 | 9.380885 | 4.18E-19 | 4.09E-18 | 32.53573678 |
| **CTNNAL1** | 0.919861 | 3.208911 | 9.380614 | 4.19E-19 | 4.10E-18 | 32.53364832 |
| **NUBP1** | 1.138976 | 4.288314 | 9.380575 | 4.19E-19 | 4.10E-18 | 32.5333411 |
| **DCBLD1** | 0.822697 | 1.976726 | 9.379311 | 4.23E-19 | 4.14E-18 | 32.5235766 |
| **ZSCAN29** | 1.134463 | 2.68267 | 9.378932 | 4.24E-19 | 4.15E-18 | 32.52064937 |
| **ROBO4** | 0.907602 | 2.834753 | 9.37891 | 4.24E-19 | 4.15E-18 | 32.52047773 |
| **MRRF** | 0.950275 | 3.103495 | 9.378848 | 4.24E-19 | 4.15E-18 | 32.51999874 |
| **PDCD2** | 1.125659 | 4.168654 | 9.378416 | 4.26E-19 | 4.16E-18 | 32.51666056 |
| **ELK4** | 0.745868 | 1.679986 | 9.378153 | 4.27E-19 | 4.17E-18 | 32.51462576 |
| **FAM120AOS** | 1.156306 | 3.877548 | 9.377779 | 4.28E-19 | 4.18E-18 | 32.51173688 |
| **KLF6** | 1.238713 | 3.154664 | 9.37775 | 4.28E-19 | 4.18E-18 | 32.5115102 |
| **ACTG1** | 1.580492 | 9.492147 | 9.377445 | 4.29E-19 | 4.18E-18 | 32.50915673 |
| **EIF4A1** | 1.635281 | 7.45573 | 9.37744 | 4.29E-19 | 4.18E-18 | 32.5091156 |
| **ADORA2A** | 0.762433 | 2.391837 | 9.377295 | 4.30E-19 | 4.19E-18 | 32.50800019 |
| **MORC3** | 0.972528 | 2.74275 | 9.377251 | 4.30E-19 | 4.19E-18 | 32.50765531 |
| **TOPORS** | 0.895062 | 2.131641 | 9.37721 | 4.30E-19 | 4.19E-18 | 32.50733852 |
| **TNFAIP1** | 1.213958 | 3.559365 | 9.376821 | 4.31E-19 | 4.20E-18 | 32.50433768 |
| **RB1** | 1.331332 | 3.103803 | 9.376301 | 4.33E-19 | 4.21E-18 | 32.50031514 |
| **ETF1** | 1.139354 | 3.865176 | 9.375853 | 4.35E-19 | 4.22E-18 | 32.49685786 |
| **LEO1** | 0.981128 | 3.09181 | 9.375851 | 4.35E-19 | 4.22E-18 | 32.49684114 |
| **CBL** | 0.863045 | 2.005752 | 9.374222 | 4.40E-19 | 4.28E-18 | 32.48425771 |
| **LINC00863** | 0.752547 | 1.919539 | 9.373005 | 4.44E-19 | 4.31E-18 | 32.47485513 |
| **SMU1** | 1.177027 | 3.867697 | 9.372292 | 4.47E-19 | 4.34E-18 | 32.46934145 |
| **KANSL3** | 0.946573 | 3.552375 | 9.372187 | 4.47E-19 | 4.34E-18 | 32.46853601 |
| **ZNF610** | 0.778503 | 1.718887 | 9.371621 | 4.49E-19 | 4.36E-18 | 32.46416423 |
| **FOXO3B** | 0.755591 | 0.913504 | 9.371394 | 4.50E-19 | 4.36E-18 | 32.46241243 |
| **RP11-452L6.5** | 0.649672 | 1.582944 | 9.371026 | 4.51E-19 | 4.37E-18 | 32.45957003 |
| **ZNF397** | 1.064978 | 3.235257 | 9.370551 | 4.53E-19 | 4.39E-18 | 32.45589748 |
| **OSBP** | 1.0971 | 3.242853 | 9.369985 | 4.55E-19 | 4.40E-18 | 32.45152654 |
| **PPAT** | 0.892978 | 1.879416 | 9.369828 | 4.56E-19 | 4.41E-18 | 32.45031661 |
| **DOK3** | 0.912531 | 2.438627 | 9.369716 | 4.56E-19 | 4.41E-18 | 32.44944819 |
| **TTYH3** | 1.457666 | 4.70349 | 9.367951 | 4.62E-19 | 4.47E-18 | 32.43582153 |
| **NCL** | 1.570852 | 6.277866 | 9.366618 | 4.67E-19 | 4.52E-18 | 32.42552576 |
| **GMCL1** | 0.942139 | 2.21695 | 9.366184 | 4.69E-19 | 4.53E-18 | 32.42217819 |
| **ADPRM** | 0.76732 | 2.535068 | 9.365695 | 4.71E-19 | 4.54E-18 | 32.41839897 |
| **C16orf80** | 1.256168 | 4.691877 | 9.36558 | 4.71E-19 | 4.55E-18 | 32.41750966 |
| **MAP4K4** | 1.457783 | 5.348142 | 9.36472 | 4.74E-19 | 4.58E-18 | 32.41087214 |
| **ZNF687** | 0.96856 | 3.641604 | 9.364535 | 4.75E-19 | 4.58E-18 | 32.40944453 |
| **KANK2** | 1.207619 | 3.550132 | 9.364105 | 4.76E-19 | 4.59E-18 | 32.40612466 |
| **CNOT8** | 1.196271 | 4.058021 | 9.363996 | 4.77E-19 | 4.60E-18 | 32.40527881 |
| **WNT5A** | 0.973134 | 1.731086 | 9.362654 | 4.82E-19 | 4.64E-18 | 32.39492426 |
| **EPHA2** | 0.875922 | 1.373469 | 9.361957 | 4.85E-19 | 4.67E-18 | 32.38954211 |
| **TAP1** | 1.255417 | 4.164727 | 9.361512 | 4.86E-19 | 4.68E-18 | 32.38610619 |
| **ADAT2** | 0.667854 | 1.723907 | 9.360811 | 4.89E-19 | 4.70E-18 | 32.3806975 |
| **POLM** | 0.933195 | 3.18146 | 9.360492 | 4.90E-19 | 4.71E-18 | 32.37823429 |
| **SH3BP5L** | 0.985237 | 3.779596 | 9.360189 | 4.91E-19 | 4.72E-18 | 32.37589912 |
| **RP11-27I1.4** | 0.708759 | 1.014388 | 9.360166 | 4.91E-19 | 4.72E-18 | 32.37572268 |
| **NOB1** | 1.154855 | 4.286601 | 9.359685 | 4.93E-19 | 4.74E-18 | 32.37200738 |
| **GPSM2** | 1.302764 | 3.531486 | 9.3596 | 4.94E-19 | 4.74E-18 | 32.37134984 |
| **ZNF350** | 0.705938 | 2.01179 | 9.359246 | 4.95E-19 | 4.75E-18 | 32.36861894 |
| **PHF20** | 1.082609 | 3.318557 | 9.358765 | 4.97E-19 | 4.77E-18 | 32.3649066 |
| **EMC3** | 1.274303 | 4.666985 | 9.35832 | 4.99E-19 | 4.78E-18 | 32.36147322 |
| **CHCHD3** | 1.269406 | 4.512236 | 9.358196 | 4.99E-19 | 4.78E-18 | 32.36051944 |
| **CSDE1** | 1.522768 | 5.812377 | 9.357998 | 5.00E-19 | 4.79E-18 | 32.3589915 |
| **CRLS1** | 1.197816 | 4.351769 | 9.357915 | 5.00E-19 | 4.79E-18 | 32.35834701 |
| **MPV17L2** | 0.94978 | 3.529463 | 9.357778 | 5.01E-19 | 4.79E-18 | 32.35728714 |
| **WAPAL** | 0.904972 | 2.903365 | 9.357639 | 5.01E-19 | 4.80E-18 | 32.35621885 |
| **C1orf109** | 1.305714 | 3.031687 | 9.357259 | 5.03E-19 | 4.81E-18 | 32.35328891 |
| **DDX5** | 1.670407 | 6.928001 | 9.35722 | 5.03E-19 | 4.81E-18 | 32.35298847 |
| **NFE2L2** | 1.250345 | 4.443904 | 9.356657 | 5.05E-19 | 4.83E-18 | 32.34864054 |
| **MIF4GD** | 1.150913 | 4.250559 | 9.35576 | 5.09E-19 | 4.86E-18 | 32.34171798 |
| **ENG** | 1.195452 | 4.096472 | 9.355245 | 5.11E-19 | 4.88E-18 | 32.3377456 |
| **SLC25A32** | 0.891017 | 2.500528 | 9.355124 | 5.11E-19 | 4.88E-18 | 32.33681245 |
| **NCSTN** | 1.343836 | 4.428745 | 9.353418 | 5.18E-19 | 4.94E-18 | 32.32365108 |
| **KRR1** | 1.118471 | 2.621673 | 9.353365 | 5.18E-19 | 4.94E-18 | 32.32324569 |
| **ARF4** | 1.472853 | 5.333717 | 9.353031 | 5.20E-19 | 4.96E-18 | 32.32066892 |
| **IGFBP4** | 1.397512 | 4.454106 | 9.352128 | 5.23E-19 | 4.99E-18 | 32.31370106 |
| **CPNE3** | 1.202516 | 3.450004 | 9.351335 | 5.27E-19 | 5.02E-18 | 32.30758612 |
| **FASTKD5** | 0.79319 | 2.068198 | 9.351002 | 5.28E-19 | 5.03E-18 | 32.30501506 |
| **MANEAL** | 1.085345 | 3.689181 | 9.349052 | 5.36E-19 | 5.10E-18 | 32.28997384 |
| **CTD-2630F21.1** | 0.630721 | 1.337206 | 9.348627 | 5.38E-19 | 5.12E-18 | 32.28669802 |
| **NCK1** | 1.154727 | 2.848316 | 9.348625 | 5.38E-19 | 5.12E-18 | 32.28668287 |
| **U2AF2** | 1.350522 | 5.992815 | 9.34773 | 5.42E-19 | 5.15E-18 | 32.27978024 |
| **CEP104** | 0.958703 | 2.656598 | 9.346933 | 5.45E-19 | 5.18E-18 | 32.27363348 |
| **ELF4** | 0.564075 | 0.763025 | 9.346496 | 5.47E-19 | 5.20E-18 | 32.27026995 |
| **ZNF142** | 0.926926 | 2.440193 | 9.345855 | 5.50E-19 | 5.22E-18 | 32.2653276 |
| **TRDMT1** | 0.663193 | 1.294844 | 9.345754 | 5.50E-19 | 5.22E-18 | 32.26454888 |
| **RABIF** | 0.760332 | 2.587758 | 9.345712 | 5.50E-19 | 5.22E-18 | 32.26422462 |
| **DYRK1A** | 1.194639 | 3.581823 | 9.345684 | 5.50E-19 | 5.22E-18 | 32.26400994 |
| **WDR92** | 0.803375 | 2.098134 | 9.345221 | 5.52E-19 | 5.24E-18 | 32.26043429 |
| **JAK1** | 1.277245 | 4.07409 | 9.344411 | 5.56E-19 | 5.27E-18 | 32.25419315 |
| **CIAPIN1** | 1.130539 | 4.434969 | 9.343743 | 5.59E-19 | 5.30E-18 | 32.24904391 |
| **ZNF134** | 1.00758 | 2.031917 | 9.343675 | 5.59E-19 | 5.30E-18 | 32.24851485 |
| **CRK** | 1.111548 | 3.973725 | 9.343331 | 5.61E-19 | 5.31E-18 | 32.24586668 |
| **MBD4** | 0.928867 | 3.101713 | 9.343299 | 5.61E-19 | 5.31E-18 | 32.24562279 |
| **LRRC28** | 0.739101 | 2.607886 | 9.342766 | 5.63E-19 | 5.33E-18 | 32.24151017 |
| **ZNF672** | 0.940918 | 3.766437 | 9.342583 | 5.64E-19 | 5.33E-18 | 32.24010061 |
| **ZFP30** | 0.83542 | 2.036143 | 9.342524 | 5.64E-19 | 5.33E-18 | 32.23964547 |
| **C6orf47** | 0.87327 | 3.049179 | 9.341802 | 5.67E-19 | 5.36E-18 | 32.23408024 |
| **FZD1** | 0.878892 | 1.859374 | 9.341497 | 5.69E-19 | 5.37E-18 | 32.23172846 |
| **SF3A2** | 1.34726 | 5.475948 | 9.34122 | 5.70E-19 | 5.38E-18 | 32.22959746 |
| **BTN2A1** | 1.057753 | 3.293795 | 9.339168 | 5.79E-19 | 5.47E-18 | 32.21378205 |
| **TRIT1** | 0.915996 | 2.98419 | 9.33882 | 5.81E-19 | 5.48E-18 | 32.21109985 |
| **JUN** | 1.55378 | 5.52055 | 9.33807 | 5.84E-19 | 5.51E-18 | 32.20532102 |
| **DNAJC14** | 1.163154 | 2.736447 | 9.337737 | 5.86E-19 | 5.52E-18 | 32.20275552 |
| **TMEM181** | 0.990596 | 3.005639 | 9.336966 | 5.89E-19 | 5.55E-18 | 32.1968117 |
| **SNX2** | 1.208425 | 3.827053 | 9.336901 | 5.89E-19 | 5.55E-18 | 32.19630858 |
| **PPP1R3B** | 0.598632 | 1.028107 | 9.336766 | 5.90E-19 | 5.56E-18 | 32.19526941 |
| **CCDC86** | 1.122921 | 3.74276 | 9.336631 | 5.91E-19 | 5.56E-18 | 32.19423352 |
| **TYSND1** | 0.80501 | 2.841272 | 9.336525 | 5.91E-19 | 5.56E-18 | 32.19341624 |
| **PCCB** | 1.169314 | 4.735905 | 9.33636 | 5.92E-19 | 5.57E-18 | 32.19213861 |
| **HBP1** | 1.127797 | 3.557791 | 9.335355 | 5.97E-19 | 5.61E-18 | 32.18439609 |
| **TNIP2** | 1.166307 | 4.357622 | 9.334959 | 5.98E-19 | 5.63E-18 | 32.18134642 |
| **C7orf55-LUC7L2** | 1.380782 | 5.36541 | 9.334816 | 5.99E-19 | 5.63E-18 | 32.18024533 |
| **PPT2** | 1.07438 | 3.70971 | 9.334548 | 6.00E-19 | 5.64E-18 | 32.178185 |
| **BIRC2** | 1.043464 | 3.627227 | 9.334326 | 6.01E-19 | 5.65E-18 | 32.17647422 |
| **MTF2** | 0.900374 | 2.316036 | 9.334064 | 6.03E-19 | 5.66E-18 | 32.17445296 |
| **PHC1** | 1.417829 | 4.189318 | 9.334015 | 6.03E-19 | 5.66E-18 | 32.17407242 |
| **PLXNA1** | 0.995522 | 3.254287 | 9.330644 | 6.19E-19 | 5.80E-18 | 32.14811165 |
| **XPO1** | 1.222743 | 4.691871 | 9.330278 | 6.21E-19 | 5.82E-18 | 32.1452864 |
| **LYSMD1** | 0.792081 | 2.540423 | 9.329888 | 6.23E-19 | 5.83E-18 | 32.14228396 |
| **VPRBP** | 1.009761 | 2.474519 | 9.32987 | 6.23E-19 | 5.83E-18 | 32.14214987 |
| **GK** | 0.795619 | 1.803714 | 9.329627 | 6.24E-19 | 5.84E-18 | 32.14027319 |
| **CDC5L** | 0.894268 | 2.642412 | 9.329384 | 6.25E-19 | 5.85E-18 | 32.13840365 |
| **USP10** | 1.22897 | 4.165635 | 9.327397 | 6.35E-19 | 5.93E-18 | 32.12310692 |
| **GATC** | 1.093697 | 2.5075 | 9.326378 | 6.40E-19 | 5.98E-18 | 32.11525959 |
| **USP15** | 1.032698 | 3.711565 | 9.32545 | 6.45E-19 | 6.02E-18 | 32.10811499 |
| **GLOD4** | 1.354433 | 5.006835 | 9.324687 | 6.48E-19 | 6.05E-18 | 32.10223739 |
| **RWDD4** | 0.931219 | 2.676397 | 9.324437 | 6.50E-19 | 6.06E-18 | 32.10031281 |
| **P4HA1** | 1.06112 | 3.258216 | 9.323676 | 6.54E-19 | 6.10E-18 | 32.09445171 |
| **SLC4A7** | 0.892171 | 1.732904 | 9.323121 | 6.56E-19 | 6.12E-18 | 32.09017951 |
| **TBC1D22B** | 0.67418 | 2.130581 | 9.32206 | 6.62E-19 | 6.17E-18 | 32.08201537 |
| **FKTN** | 0.903801 | 2.052629 | 9.320389 | 6.71E-19 | 6.25E-18 | 32.06915647 |
| **ZNF607** | 0.585727 | 1.690795 | 9.320075 | 6.72E-19 | 6.26E-18 | 32.06674094 |
| **LL22NC03-80A10.6** | 0.782018 | 2.115115 | 9.319829 | 6.74E-19 | 6.27E-18 | 32.06484701 |
| **SMARCA1** | 1.061909 | 3.368813 | 9.318577 | 6.80E-19 | 6.33E-18 | 32.05520737 |
| **NCOA3** | 0.7669 | 1.96781 | 9.318341 | 6.81E-19 | 6.34E-18 | 32.05338999 |
| **C11orf57** | 0.996406 | 3.277192 | 9.318009 | 6.83E-19 | 6.35E-18 | 32.05084024 |
| **PLCB3** | 0.908252 | 3.183725 | 9.315852 | 6.95E-19 | 6.46E-18 | 32.03424048 |
| **PRKAR2A** | 1.22578 | 2.708495 | 9.314999 | 6.99E-19 | 6.50E-18 | 32.02767711 |
| **PWP1** | 1.081279 | 3.830287 | 9.314653 | 7.01E-19 | 6.51E-18 | 32.02501855 |
| **ARHGAP19** | 0.745271 | 1.988517 | 9.313293 | 7.09E-19 | 6.58E-18 | 32.01455295 |
| **ZNF644** | 1.022943 | 3.373046 | 9.313146 | 7.10E-19 | 6.58E-18 | 32.0134254 |
| **MOB4** | 1.154067 | 3.321084 | 9.312699 | 7.12E-19 | 6.60E-18 | 32.00998837 |
| **AGPAT5** | 1.227028 | 3.890087 | 9.312235 | 7.15E-19 | 6.63E-18 | 32.0064184 |
| **AC009506.1** | 0.743462 | 2.25227 | 9.312156 | 7.15E-19 | 6.63E-18 | 32.00580567 |
| **NPLOC4** | 1.035063 | 4.366341 | 9.312064 | 7.16E-19 | 6.63E-18 | 32.00510371 |
| **POLDIP3** | 1.331117 | 4.887463 | 9.310231 | 7.26E-19 | 6.72E-18 | 31.99100122 |
| **RAB11A** | 1.533911 | 5.544338 | 9.309328 | 7.31E-19 | 6.77E-18 | 31.98405427 |
| **SCLT1** | 0.617106 | 1.837578 | 9.309053 | 7.33E-19 | 6.78E-18 | 31.98194091 |
| **MRPS10** | 0.997554 | 3.489378 | 9.308561 | 7.35E-19 | 6.80E-18 | 31.97816165 |
| **RAPGEF1** | 1.229629 | 4.146521 | 9.308177 | 7.38E-19 | 6.82E-18 | 31.9752104 |
| **ZNF507** | 0.775325 | 1.745977 | 9.307951 | 7.39E-19 | 6.83E-18 | 31.97346788 |
| **UXS1** | 1.05561 | 3.651183 | 9.307358 | 7.42E-19 | 6.86E-18 | 31.96890617 |
| **FOSL2** | 1.20254 | 2.644081 | 9.307318 | 7.43E-19 | 6.86E-18 | 31.96859771 |
| **C17orf58** | 0.905234 | 2.915046 | 9.307255 | 7.43E-19 | 6.86E-18 | 31.96811822 |
| **SUPT16H** | 1.2181 | 4.738394 | 9.306965 | 7.45E-19 | 6.87E-18 | 31.96588476 |
| **FXR1** | 1.217945 | 4.759058 | 9.30688 | 7.45E-19 | 6.87E-18 | 31.9652309 |
| **TAF12** | 0.948769 | 3.065686 | 9.306703 | 7.46E-19 | 6.88E-18 | 31.96387379 |
| **TCF4** | 1.327921 | 4.394609 | 9.306212 | 7.49E-19 | 6.90E-18 | 31.96009646 |
| **GMPPB** | 0.915292 | 2.422343 | 9.304961 | 7.56E-19 | 6.97E-18 | 31.95048299 |
| **UBE2K** | 1.132421 | 3.87947 | 9.304676 | 7.58E-19 | 6.98E-18 | 31.94828792 |
| **MAD2L1BP** | 1.004183 | 3.664845 | 9.303941 | 7.62E-19 | 7.02E-18 | 31.9426367 |
| **SRSF7** | 1.416055 | 5.371702 | 9.303749 | 7.64E-19 | 7.03E-18 | 31.94115969 |
| **MANEA** | 0.796953 | 1.406149 | 9.303434 | 7.65E-19 | 7.04E-18 | 31.93873847 |
| **SDE2** | 1.047886 | 2.299824 | 9.302855 | 7.69E-19 | 7.07E-18 | 31.93428815 |
| **ZNF761** | 0.608497 | 1.560986 | 9.30225 | 7.73E-19 | 7.10E-18 | 31.92963739 |
| **CSNK2A1** | 1.198881 | 4.20144 | 9.302095 | 7.73E-19 | 7.11E-18 | 31.9284439 |
| **ORC5** | 0.79918 | 2.870334 | 9.302071 | 7.74E-19 | 7.11E-18 | 31.92826312 |
| **MAU2** | 1.223418 | 4.080597 | 9.302023 | 7.74E-19 | 7.11E-18 | 31.92789167 |
| **NCKAP5L** | 1.007844 | 3.657674 | 9.301071 | 7.80E-19 | 7.16E-18 | 31.92057933 |
| **RBM18** | 0.888905 | 2.395993 | 9.299986 | 7.86E-19 | 7.22E-18 | 31.91223485 |
| **WDR12** | 1.066957 | 3.95982 | 9.297372 | 8.02E-19 | 7.36E-18 | 31.89215049 |
| **HIST2H2BC** | 0.761585 | 1.152996 | 9.297212 | 8.03E-19 | 7.37E-18 | 31.89092216 |
| **SPG21** | 1.200452 | 4.724828 | 9.29684 | 8.06E-19 | 7.39E-18 | 31.88806478 |
| **DDX31** | 0.752092 | 2.638227 | 9.296649 | 8.07E-19 | 7.39E-18 | 31.88659764 |
| **RNF20** | 1.185561 | 3.876965 | 9.296316 | 8.09E-19 | 7.41E-18 | 31.88403288 |
| **BCL6** | 1.413973 | 4.555636 | 9.29592 | 8.12E-19 | 7.43E-18 | 31.88099604 |
| **AP4E1** | 0.557587 | 1.296597 | 9.295752 | 8.13E-19 | 7.44E-18 | 31.87970073 |
| **ZNF234** | 0.729137 | 1.395422 | 9.294629 | 8.20E-19 | 7.50E-18 | 31.87107511 |
| **TGIF2** | 0.81145 | 2.009982 | 9.294299 | 8.22E-19 | 7.52E-18 | 31.86854164 |
| **SMG7** | 1.040627 | 3.650861 | 9.294116 | 8.23E-19 | 7.52E-18 | 31.86713277 |
| **CSE1L** | 1.055396 | 4.044825 | 9.294015 | 8.24E-19 | 7.53E-18 | 31.86636159 |
| **TMEM214** | 1.04444 | 4.029652 | 9.293558 | 8.27E-19 | 7.55E-18 | 31.86284782 |
| **DCUN1D3** | 0.548046 | 1.492625 | 9.293284 | 8.28E-19 | 7.56E-18 | 31.86074149 |
| **NUS1** | 0.965698 | 2.925065 | 9.292271 | 8.35E-19 | 7.62E-18 | 31.85295984 |
| **KLHL42** | 0.989704 | 3.022535 | 9.291887 | 8.37E-19 | 7.64E-18 | 31.85001239 |
| **SOX9** | 1.49536 | 4.510598 | 9.289783 | 8.51E-19 | 7.76E-18 | 31.83385295 |
| **NUB1** | 1.118655 | 4.371525 | 9.289492 | 8.53E-19 | 7.78E-18 | 31.8316155 |
| **DNAJC1** | 0.880525 | 2.944336 | 9.28924 | 8.55E-19 | 7.79E-18 | 31.82968272 |
| **GATAD2B** | 0.927111 | 2.829079 | 9.288463 | 8.60E-19 | 7.84E-18 | 31.82371331 |
| **GFPT1** | 0.843459 | 2.134099 | 9.288196 | 8.62E-19 | 7.85E-18 | 31.82166252 |
| **GPATCH1** | 0.692662 | 2.230134 | 9.286782 | 8.71E-19 | 7.93E-18 | 31.81080829 |
| **EIF2S1** | 1.463037 | 4.500693 | 9.286763 | 8.72E-19 | 7.93E-18 | 31.8106602 |
| **SRRD** | 0.789263 | 2.55037 | 9.286487 | 8.73E-19 | 7.95E-18 | 31.80854426 |
| **USP7** | 1.163469 | 4.312354 | 9.28621 | 8.75E-19 | 7.96E-18 | 31.80641945 |
| **KLHL20** | 1.182144 | 2.413777 | 9.28545 | 8.81E-19 | 8.00E-18 | 31.80058219 |
| **GDI2** | 1.330252 | 5.460091 | 9.285406 | 8.81E-19 | 8.00E-18 | 31.80024576 |
| **TMEM185B** | 1.004567 | 2.136741 | 9.284499 | 8.87E-19 | 8.06E-18 | 31.79328243 |
| **TPST1** | 1.187639 | 3.938496 | 9.284396 | 8.88E-19 | 8.06E-18 | 31.79249101 |
| **CD44** | 1.624421 | 4.175911 | 9.283446 | 8.94E-19 | 8.12E-18 | 31.78519634 |
| **XBP1** | 1.183377 | 4.076245 | 9.283377 | 8.95E-19 | 8.12E-18 | 31.7846703 |
| **ZNF184** | 0.751886 | 1.703216 | 9.28305 | 8.97E-19 | 8.14E-18 | 31.78216049 |
| **ASAP1** | 1.166746 | 3.102381 | 9.282253 | 9.03E-19 | 8.18E-18 | 31.77603899 |
| **TMEM140** | 0.975083 | 2.738632 | 9.281931 | 9.05E-19 | 8.20E-18 | 31.77356804 |
| **CLIC2** | 0.654743 | 1.371412 | 9.280164 | 9.18E-19 | 8.31E-18 | 31.76000308 |
| **LARS** | 1.219804 | 4.496937 | 9.279463 | 9.23E-19 | 8.35E-18 | 31.7546289 |
| **TP73** | 0.667078 | 0.914699 | 9.278725 | 9.28E-19 | 8.40E-18 | 31.74896153 |
| **ZNF510** | 0.850271 | 1.968605 | 9.278705 | 9.28E-19 | 8.40E-18 | 31.74881229 |
| **SEC23IP** | 0.917722 | 2.360524 | 9.278455 | 9.30E-19 | 8.41E-18 | 31.74688939 |
| **GOLT1B** | 1.027976 | 3.112855 | 9.278416 | 9.30E-19 | 8.41E-18 | 31.74659149 |
| **ZNF599** | 0.782968 | 2.592411 | 9.277417 | 9.37E-19 | 8.47E-18 | 31.73892889 |
| **EXTL3** | 1.003381 | 3.852612 | 9.277382 | 9.38E-19 | 8.47E-18 | 31.73865959 |
| **TIGD1** | 0.77959 | 2.337026 | 9.277028 | 9.40E-19 | 8.49E-18 | 31.73593807 |
| **TANK** | 1.156351 | 3.75912 | 9.276648 | 9.43E-19 | 8.51E-18 | 31.73302822 |
| **RCOR3** | 1.083992 | 3.707303 | 9.272251 | 9.76E-19 | 8.81E-18 | 31.69929137 |
| **EI24** | 1.215137 | 4.805802 | 9.272177 | 9.76E-19 | 8.81E-18 | 31.69872829 |
| **SKI** | 1.318095 | 3.474998 | 9.27169 | 9.80E-19 | 8.84E-18 | 31.69499313 |
| **SSH1** | 0.876686 | 2.149877 | 9.271341 | 9.83E-19 | 8.86E-18 | 31.69231763 |
| **MIEF1** | 0.978649 | 3.205162 | 9.270967 | 9.86E-19 | 8.88E-18 | 31.6894497 |
| **MARK1** | 0.929447 | 2.346292 | 9.270683 | 9.88E-19 | 8.90E-18 | 31.6872709 |
| **CNKSR3** | 0.942169 | 1.976114 | 9.270452 | 9.90E-19 | 8.91E-18 | 31.6854941 |
| **C1orf174** | 0.863264 | 2.725693 | 9.269013 | 1.00E-18 | 9.01E-18 | 31.67445841 |
| **MBD6** | 1.288101 | 4.109492 | 9.26892 | 1.00E-18 | 9.01E-18 | 31.67374443 |
| **RP11-196G18.23** | 0.754458 | 2.174604 | 9.26853 | 1.00E-18 | 9.03E-18 | 31.67075579 |
| **LMAN1** | 1.156784 | 2.914607 | 9.268182 | 1.01E-18 | 9.06E-18 | 31.66809184 |
| **MUL1** | 0.997752 | 3.491489 | 9.268029 | 1.01E-18 | 9.06E-18 | 31.66691698 |
| **TADA2B** | 0.958936 | 2.791017 | 9.268026 | 1.01E-18 | 9.06E-18 | 31.66689011 |
| **COL6A1** | 1.490816 | 5.979227 | 9.267727 | 1.01E-18 | 9.08E-18 | 31.6646012 |
| **BACH2** | 0.54534 | 0.997219 | 9.267073 | 1.02E-18 | 9.12E-18 | 31.65958271 |
| **GNG5** | 1.553632 | 5.227983 | 9.266567 | 1.02E-18 | 9.15E-18 | 31.65570364 |
| **TRMT10C** | 0.92337 | 3.256097 | 9.266103 | 1.02E-18 | 9.18E-18 | 31.65214697 |
| **VTI1A** | 0.896334 | 2.344465 | 9.263679 | 1.04E-18 | 9.35E-18 | 31.63356445 |
| **SREK1** | 1.419487 | 3.618826 | 9.26345 | 1.04E-18 | 9.37E-18 | 31.6318094 |
| **CBLL1** | 0.923222 | 2.567701 | 9.26282 | 1.05E-18 | 9.41E-18 | 31.62698156 |
| **WWTR1** | 1.314251 | 2.928032 | 9.262424 | 1.05E-18 | 9.43E-18 | 31.62394712 |
| **MRGBP** | 0.926707 | 3.409298 | 9.262305 | 1.05E-18 | 9.44E-18 | 31.62303326 |
| **EEA1** | 0.85342 | 1.731282 | 9.261196 | 1.06E-18 | 9.52E-18 | 31.61453606 |
| **ECSCR** | 1.286059 | 3.499307 | 9.260848 | 1.07E-18 | 9.54E-18 | 31.61186584 |
| **JRKL** | 0.955522 | 1.88349 | 9.260689 | 1.07E-18 | 9.55E-18 | 31.61064519 |
| **PACRGL** | 0.951636 | 3.226899 | 9.260201 | 1.07E-18 | 9.58E-18 | 31.60690705 |
| **CEP250** | 0.838683 | 3.106494 | 9.258666 | 1.08E-18 | 9.69E-18 | 31.59514246 |
| **AGRN** | 1.366056 | 5.949204 | 9.257707 | 1.09E-18 | 9.76E-18 | 31.58779283 |
| **SLC29A1** | 1.18888 | 3.709298 | 9.257441 | 1.09E-18 | 9.78E-18 | 31.58575531 |
| **NAF1** | 0.830126 | 1.918494 | 9.25678 | 1.10E-18 | 9.82E-18 | 31.58069317 |
| **SSFA2** | 1.402208 | 4.29617 | 9.256327 | 1.10E-18 | 9.86E-18 | 31.57722584 |
| **CHPF2** | 0.960544 | 3.748352 | 9.256054 | 1.11E-18 | 9.87E-18 | 31.57512778 |
| **SH3BP2** | 1.181174 | 4.600164 | 9.253204 | 1.13E-18 | 1.01E-17 | 31.55329542 |
| **IPMK** | 0.709046 | 1.344913 | 9.25288 | 1.13E-18 | 1.01E-17 | 31.55081991 |
| **TATDN2** | 0.971989 | 3.833416 | 9.25208 | 1.14E-18 | 1.02E-17 | 31.54468836 |
| **HKR1** | 1.081707 | 3.96856 | 9.25183 | 1.14E-18 | 1.02E-17 | 31.54277587 |
| **TAF1** | 1.0471 | 2.442523 | 9.251701 | 1.14E-18 | 1.02E-17 | 31.54178829 |
| **NABP1** | 0.908688 | 1.948082 | 9.251389 | 1.15E-18 | 1.02E-17 | 31.53939597 |
| **USP4** | 1.058142 | 3.84829 | 9.250735 | 1.15E-18 | 1.03E-17 | 31.53439094 |
| **GOLGA3** | 0.839403 | 3.328755 | 9.250459 | 1.16E-18 | 1.03E-17 | 31.53226998 |
| **TROVE2** | 1.211748 | 2.43038 | 9.25022 | 1.16E-18 | 1.03E-17 | 31.53044255 |
| **BPTF** | 1.119504 | 3.765031 | 9.249885 | 1.16E-18 | 1.03E-17 | 31.52787719 |
| **CTC-459F4.3** | 0.789661 | 2.541441 | 9.249703 | 1.16E-18 | 1.03E-17 | 31.52648029 |
| **TMEM218** | 1.157999 | 3.898128 | 9.249698 | 1.16E-18 | 1.03E-17 | 31.52644798 |
| **LINC01003** | 1.122445 | 3.733326 | 9.248658 | 1.17E-18 | 1.04E-17 | 31.51848213 |
| **TMEM167A** | 1.318155 | 4.146502 | 9.247341 | 1.18E-18 | 1.05E-17 | 31.50839379 |
| **PCNX** | 0.778957 | 2.119796 | 9.247198 | 1.19E-18 | 1.05E-17 | 31.50730523 |
| **NAA35** | 0.975197 | 2.718847 | 9.24658 | 1.19E-18 | 1.06E-17 | 31.5025711 |
| **METTL14** | 0.950918 | 2.242209 | 9.245735 | 1.20E-18 | 1.06E-17 | 31.49610096 |
| **VSIG10** | 0.858856 | 2.458917 | 9.244886 | 1.21E-18 | 1.07E-17 | 31.48960164 |
| **YARS2** | 0.801636 | 2.538193 | 9.24443 | 1.21E-18 | 1.07E-17 | 31.48610782 |
| **CRCP** | 1.081631 | 3.946759 | 9.244284 | 1.21E-18 | 1.07E-17 | 31.48498995 |
| **PPM1G** | 1.265943 | 5.379396 | 9.243846 | 1.22E-18 | 1.08E-17 | 31.48163963 |
| **ELMSAN1** | 0.997637 | 2.444376 | 9.243386 | 1.22E-18 | 1.08E-17 | 31.47812017 |
| **NUP210** | 0.847313 | 2.0398 | 9.241498 | 1.24E-18 | 1.10E-17 | 31.46367141 |
| **FGFR1** | 1.25293 | 3.909801 | 9.241049 | 1.24E-18 | 1.10E-17 | 31.46023204 |
| **ZBTB6** | 0.773179 | 2.089717 | 9.240878 | 1.25E-18 | 1.10E-17 | 31.45892733 |
| **CHD9** | 1.0197 | 3.187284 | 9.239764 | 1.26E-18 | 1.11E-17 | 31.45040161 |
| **KIAA1430** | 1.136203 | 2.454779 | 9.239606 | 1.26E-18 | 1.11E-17 | 31.44919187 |
| **ARHGAP12** | 1.17875 | 4.24715 | 9.239305 | 1.26E-18 | 1.11E-17 | 31.44688774 |
| **KDELR1** | 1.328853 | 5.070731 | 9.238815 | 1.27E-18 | 1.12E-17 | 31.44313259 |
| **VPS25** | 1.255366 | 5.063204 | 9.237427 | 1.28E-18 | 1.13E-17 | 31.43251637 |
| **SHMT1** | 0.883366 | 2.913155 | 9.237118 | 1.28E-18 | 1.13E-17 | 31.43015171 |
| **ZNF461** | 0.847159 | 1.950643 | 9.236993 | 1.28E-18 | 1.13E-17 | 31.42919679 |
| **CLINT1** | 1.222028 | 3.220729 | 9.236149 | 1.29E-18 | 1.14E-17 | 31.4227399 |
| **PCNXL3** | 0.962099 | 3.703986 | 9.235995 | 1.29E-18 | 1.14E-17 | 31.4215625 |
| **PPIH** | 1.156405 | 4.46669 | 9.235931 | 1.29E-18 | 1.14E-17 | 31.42106776 |
| **ABCF2** | 1.049127 | 3.977139 | 9.234836 | 1.31E-18 | 1.15E-17 | 31.41269208 |
| **NUDT21** | 1.337653 | 4.030287 | 9.234452 | 1.31E-18 | 1.15E-17 | 31.40975853 |
| **GPR65** | 0.70332 | 0.900561 | 9.233052 | 1.32E-18 | 1.16E-17 | 31.39905054 |
| **PALLD** | 1.321261 | 3.497717 | 9.232955 | 1.32E-18 | 1.16E-17 | 31.39830202 |
| **PRKRA** | 1.185027 | 4.325359 | 9.231284 | 1.34E-18 | 1.18E-17 | 31.38552393 |
| **CTBP2** | 1.235313 | 3.727316 | 9.230689 | 1.35E-18 | 1.18E-17 | 31.38097062 |
| **FAM114A1** | 0.950196 | 2.041868 | 9.230575 | 1.35E-18 | 1.18E-17 | 31.38010503 |
| **STAT5B** | 1.168332 | 3.840471 | 9.230408 | 1.35E-18 | 1.19E-17 | 31.37882548 |
| **RNF121** | 1.042916 | 3.560292 | 9.230351 | 1.35E-18 | 1.19E-17 | 31.37838873 |
| **ITGB1P1** | 0.564193 | 0.542033 | 9.230346 | 1.35E-18 | 1.19E-17 | 31.37835075 |
| **PHF23** | 1.133932 | 4.440221 | 9.230121 | 1.35E-18 | 1.19E-17 | 31.37662995 |
| **REST** | 0.785041 | 1.380925 | 9.230108 | 1.35E-18 | 1.19E-17 | 31.37653009 |
| **TSN** | 1.163706 | 4.328976 | 9.229994 | 1.36E-18 | 1.19E-17 | 31.37565623 |
| **CECR5** | 1.221847 | 4.678057 | 9.229693 | 1.36E-18 | 1.19E-17 | 31.37335408 |
| **NES** | 1.38772 | 6.087941 | 9.229359 | 1.36E-18 | 1.19E-17 | 31.37080489 |
| **ARMCX6** | 1.402308 | 4.335303 | 9.229298 | 1.36E-18 | 1.19E-17 | 31.37033552 |
| **UBE2R2** | 1.09983 | 4.050045 | 9.227749 | 1.38E-18 | 1.21E-17 | 31.35848878 |
| **TBC1D19** | 0.634581 | 1.470339 | 9.227237 | 1.38E-18 | 1.21E-17 | 31.3545807 |
| **CLK2** | 1.257055 | 4.348355 | 9.226322 | 1.39E-18 | 1.22E-17 | 31.34758146 |
| **IVNS1ABP** | 1.281524 | 4.702475 | 9.225696 | 1.40E-18 | 1.22E-17 | 31.34279935 |
| **ADH5** | 1.303207 | 4.617631 | 9.225286 | 1.41E-18 | 1.23E-17 | 31.33966216 |
| **AC125232.1** | 0.861144 | 1.655142 | 9.225112 | 1.41E-18 | 1.23E-17 | 31.33833003 |
| **ZNF736** | 0.850381 | 2.307909 | 9.22456 | 1.41E-18 | 1.23E-17 | 31.33411357 |
| **ZNFX1** | 0.817118 | 2.643688 | 9.224482 | 1.41E-18 | 1.23E-17 | 31.33351906 |
| **KIAA1958** | 0.720016 | 1.415505 | 9.224287 | 1.42E-18 | 1.23E-17 | 31.33202327 |
| **ZNF559** | 1.026195 | 3.699118 | 9.22288 | 1.43E-18 | 1.25E-17 | 31.32126996 |
| **ARNT** | 0.947766 | 2.726241 | 9.222879 | 1.43E-18 | 1.25E-17 | 31.32125977 |
| **C4orf33** | 0.874574 | 2.541615 | 9.222555 | 1.44E-18 | 1.25E-17 | 31.31878369 |
| **PVR** | 0.813217 | 2.630974 | 9.222286 | 1.44E-18 | 1.25E-17 | 31.31673374 |
| **SNX16** | 0.849349 | 1.83008 | 9.222225 | 1.44E-18 | 1.25E-17 | 31.3162665 |
| **BCLAF1** | 1.254837 | 4.011271 | 9.221734 | 1.44E-18 | 1.26E-17 | 31.31250844 |
| **ZNF326** | 1.165275 | 3.303587 | 9.221632 | 1.45E-18 | 1.26E-17 | 31.31173091 |
| **DNAJB1** | 1.39929 | 5.236616 | 9.221541 | 1.45E-18 | 1.26E-17 | 31.31103421 |
| **JOSD1** | 0.953699 | 4.00715 | 9.22118 | 1.45E-18 | 1.26E-17 | 31.30827625 |
| **UBA6** | 1.112175 | 2.499455 | 9.221105 | 1.45E-18 | 1.26E-17 | 31.30770559 |
| **MBTPS2** | 0.89695 | 2.1688 | 9.221105 | 1.45E-18 | 1.26E-17 | 31.30770528 |
| **ANKRD49** | 0.867165 | 2.224519 | 9.221005 | 1.45E-18 | 1.26E-17 | 31.30694061 |
| **SNRPD3** | 1.353898 | 5.384036 | 9.220638 | 1.46E-18 | 1.26E-17 | 31.30413653 |
| **ZNF485** | 0.541376 | 1.156339 | 9.220635 | 1.46E-18 | 1.26E-17 | 31.30411547 |
| **SCIMP** | 0.657953 | 1.183938 | 9.219456 | 1.47E-18 | 1.28E-17 | 31.29509982 |
| **MCM3AP** | 1.155275 | 4.775471 | 9.219351 | 1.47E-18 | 1.28E-17 | 31.29430206 |
| **RPF2** | 0.880531 | 3.270541 | 9.219308 | 1.47E-18 | 1.28E-17 | 31.29397048 |
| **CRNDE** | 1.433873 | 2.421471 | 9.218788 | 1.48E-18 | 1.28E-17 | 31.29000266 |
| **MBTPS1** | 1.168701 | 4.467024 | 9.217299 | 1.50E-18 | 1.29E-17 | 31.27862099 |
| **LIX1L** | 1.048633 | 3.48229 | 9.217217 | 1.50E-18 | 1.30E-17 | 31.27799563 |
| **ZNF605** | 0.806532 | 2.039244 | 9.217048 | 1.50E-18 | 1.30E-17 | 31.27670683 |
| **TNRC18** | 1.298169 | 4.390087 | 9.21694 | 1.50E-18 | 1.30E-17 | 31.275882 |
| **AVL9** | 0.849812 | 2.868947 | 9.216677 | 1.50E-18 | 1.30E-17 | 31.27387083 |
| **ZC3H3** | 0.811083 | 3.096935 | 9.216506 | 1.50E-18 | 1.30E-17 | 31.27256228 |
| **GNG12** | 1.236243 | 2.401721 | 9.216105 | 1.51E-18 | 1.30E-17 | 31.26949868 |
| **CXorf24** | 0.872955 | 1.526635 | 9.216084 | 1.51E-18 | 1.30E-17 | 31.26933955 |
| **FAM72B** | 0.582959 | 1.672162 | 9.215963 | 1.51E-18 | 1.30E-17 | 31.26841367 |
| **INPPL1** | 1.407248 | 5.484153 | 9.215379 | 1.52E-18 | 1.31E-17 | 31.26395218 |
| **PSMD5** | 0.916921 | 2.535535 | 9.214692 | 1.53E-18 | 1.32E-17 | 31.25870376 |
| **PCSK7** | 1.104144 | 4.517174 | 9.21468 | 1.53E-18 | 1.32E-17 | 31.25861424 |
| **HIST2H2BE** | 0.908847 | 2.977619 | 9.214368 | 1.53E-18 | 1.32E-17 | 31.25623112 |
| **UPRT** | 0.924914 | 2.560135 | 9.214136 | 1.53E-18 | 1.32E-17 | 31.25445553 |
| **AGA** | 0.975931 | 2.809998 | 9.213379 | 1.54E-18 | 1.33E-17 | 31.24867275 |
| **OXNAD1** | 0.624255 | 2.177358 | 9.213096 | 1.54E-18 | 1.33E-17 | 31.24651314 |
| **PACSIN2** | 1.085298 | 4.085862 | 9.213075 | 1.54E-18 | 1.33E-17 | 31.24635165 |
| **CAMSAP1** | 1.057812 | 3.224843 | 9.212873 | 1.55E-18 | 1.33E-17 | 31.24480864 |
| **UBE3C** | 1.055911 | 3.229755 | 9.212738 | 1.55E-18 | 1.33E-17 | 31.24378395 |
| **VTA1** | 1.253932 | 3.160151 | 9.212588 | 1.55E-18 | 1.33E-17 | 31.24263219 |
| **ZC3H18** | 1.073007 | 4.345901 | 9.212571 | 1.55E-18 | 1.33E-17 | 31.24250666 |
| **ADO** | 1.049732 | 3.666798 | 9.212491 | 1.55E-18 | 1.33E-17 | 31.24189039 |
| **C19orf54** | 0.805082 | 2.694996 | 9.211837 | 1.56E-18 | 1.34E-17 | 31.23689544 |
| **GTF2H3** | 1.172668 | 2.985357 | 9.21183 | 1.56E-18 | 1.34E-17 | 31.23684476 |
| **EIF4G2** | 1.635718 | 6.142413 | 9.211362 | 1.57E-18 | 1.34E-17 | 31.23327517 |
| **POLR3E** | 1.119652 | 4.064419 | 9.211026 | 1.57E-18 | 1.35E-17 | 31.23070648 |
| **SRPR** | 1.125643 | 3.882671 | 9.211022 | 1.57E-18 | 1.35E-17 | 31.23067587 |
| **RBAK** | 0.7683 | 1.830533 | 9.209991 | 1.58E-18 | 1.36E-17 | 31.22280387 |
| **LCLAT1** | 0.549182 | 1.665805 | 9.2096 | 1.59E-18 | 1.36E-17 | 31.21981898 |
| **C9orf85** | 0.796991 | 2.744647 | 9.209479 | 1.59E-18 | 1.36E-17 | 31.21889385 |
| **TIFA** | 0.51413 | 0.78875 | 9.209203 | 1.59E-18 | 1.36E-17 | 31.21678337 |
| **TMEM87B** | 0.628818 | 1.570942 | 9.208619 | 1.60E-18 | 1.37E-17 | 31.21232779 |
| **SNRPB2** | 1.212988 | 4.555292 | 9.207535 | 1.61E-18 | 1.38E-17 | 31.20404618 |
| **PABPC4** | 1.262127 | 5.062772 | 9.207457 | 1.61E-18 | 1.38E-17 | 31.20345403 |
| **PRKDC** | 1.409776 | 3.495001 | 9.206914 | 1.62E-18 | 1.39E-17 | 31.19930952 |
| **C17orf62** | 1.221415 | 5.683667 | 9.20683 | 1.62E-18 | 1.39E-17 | 31.1986639 |
| **RAB8B** | 1.107545 | 2.719905 | 9.206359 | 1.63E-18 | 1.39E-17 | 31.19506666 |
| **GAPVD1** | 0.929451 | 2.882797 | 9.205721 | 1.64E-18 | 1.40E-17 | 31.19019635 |
| **TUG1** | 1.219064 | 4.274425 | 9.205707 | 1.64E-18 | 1.40E-17 | 31.19008983 |
| **LRRN1** | 1.617134 | 3.787158 | 9.205365 | 1.64E-18 | 1.40E-17 | 31.1874806 |
| **SUPT3H** | 0.623139 | 2.089335 | 9.205196 | 1.64E-18 | 1.40E-17 | 31.18619185 |
| **WBP11** | 1.21263 | 3.875566 | 9.204965 | 1.65E-18 | 1.40E-17 | 31.18443187 |
| **GOLPH3** | 1.18583 | 4.045626 | 9.204492 | 1.65E-18 | 1.41E-17 | 31.18081474 |
| **FXN** | 0.861038 | 2.821921 | 9.203895 | 1.66E-18 | 1.41E-17 | 31.17626482 |
| **EIF2AK2** | 1.039315 | 2.665582 | 9.203381 | 1.67E-18 | 1.42E-17 | 31.17234029 |
| **PTRF** | 1.257413 | 3.578685 | 9.20323 | 1.67E-18 | 1.42E-17 | 31.17118893 |
| **FOXN4** | 0.517245 | 0.617947 | 9.202294 | 1.68E-18 | 1.43E-17 | 31.16404251 |
| **C5orf24** | 1.228332 | 3.345593 | 9.202282 | 1.68E-18 | 1.43E-17 | 31.1639503 |
| **HMGN2P5** | 1.395393 | 3.971321 | 9.201331 | 1.69E-18 | 1.44E-17 | 31.15669378 |
| **SERP1** | 1.384936 | 5.425493 | 9.200772 | 1.70E-18 | 1.45E-17 | 31.15242696 |
| **RNF145** | 1.251836 | 4.19119 | 9.200237 | 1.71E-18 | 1.45E-17 | 31.14834176 |
| **CASP8** | 0.758069 | 1.343453 | 9.200218 | 1.71E-18 | 1.45E-17 | 31.14819627 |
| **FBXL4** | 0.560235 | 1.490734 | 9.198423 | 1.73E-18 | 1.47E-17 | 31.13450212 |
| **VARS** | 1.213169 | 5.188298 | 9.198339 | 1.73E-18 | 1.47E-17 | 31.13386174 |
| **FPGT** | 0.807647 | 1.65414 | 9.197677 | 1.74E-18 | 1.48E-17 | 31.12881384 |
| **ZNF786** | 0.774633 | 1.902956 | 9.197147 | 1.75E-18 | 1.48E-17 | 31.12476809 |
| **RHOQ** | 1.186527 | 4.200419 | 9.196927 | 1.75E-18 | 1.49E-17 | 31.12309178 |
| **ZBTB34** | 0.5636 | 1.25652 | 9.196839 | 1.75E-18 | 1.49E-17 | 31.12241459 |
| **TMEM198B** | 1.108279 | 4.095571 | 9.196482 | 1.76E-18 | 1.49E-17 | 31.11969708 |
| **ZNF101** | 0.585691 | 1.976266 | 9.196115 | 1.76E-18 | 1.49E-17 | 31.11689149 |
| **MAP3K7** | 0.966488 | 2.929932 | 9.195919 | 1.76E-18 | 1.50E-17 | 31.11539709 |
| **ZNF721** | 1.023071 | 2.814067 | 9.195493 | 1.77E-18 | 1.50E-17 | 31.11215015 |
| **ZNF624** | 0.579275 | 1.235097 | 9.195134 | 1.78E-18 | 1.50E-17 | 31.10940673 |
| **ERF** | 1.31789 | 4.885725 | 9.194819 | 1.78E-18 | 1.51E-17 | 31.10700485 |
| **DPY19L4** | 1.077427 | 2.893312 | 9.193231 | 1.80E-18 | 1.52E-17 | 31.09489465 |
| **DHX16** | 0.923276 | 3.679904 | 9.193202 | 1.80E-18 | 1.52E-17 | 31.09467336 |
| **MS4A6A** | 1.617497 | 3.950823 | 9.192024 | 1.82E-18 | 1.54E-17 | 31.08568803 |
| **NARS2** | 0.874776 | 2.967671 | 9.191921 | 1.82E-18 | 1.54E-17 | 31.08490339 |
| **EXOSC2** | 0.943685 | 3.916533 | 9.191896 | 1.82E-18 | 1.54E-17 | 31.0847145 |
| **INIP** | 0.982818 | 2.576371 | 9.191521 | 1.83E-18 | 1.54E-17 | 31.08185441 |
| **UTP14A** | 0.776544 | 2.913898 | 9.191317 | 1.83E-18 | 1.54E-17 | 31.08029513 |
| **ZNF830** | 1.02173 | 3.372523 | 9.191002 | 1.83E-18 | 1.55E-17 | 31.07789783 |
| **RP11-145M9.4** | 1.161626 | 3.931439 | 9.190746 | 1.84E-18 | 1.55E-17 | 31.07594635 |
| **BET1** | 1.023346 | 3.207064 | 9.190671 | 1.84E-18 | 1.55E-17 | 31.07536779 |
| **YES1** | 1.121707 | 2.920857 | 9.190317 | 1.84E-18 | 1.55E-17 | 31.0726691 |
| **TRIP11** | 0.794641 | 1.851479 | 9.190307 | 1.84E-18 | 1.55E-17 | 31.0725953 |
| **RP11-299J3.8** | 0.886293 | 2.772029 | 9.188946 | 1.86E-18 | 1.57E-17 | 31.06222132 |
| **FAM208B** | 0.838949 | 3.134453 | 9.188216 | 1.87E-18 | 1.58E-17 | 31.05665143 |
| **MFAP1** | 1.015429 | 3.72963 | 9.188102 | 1.87E-18 | 1.58E-17 | 31.05578262 |
| **RP4-739H11.4** | 0.794839 | 1.225213 | 9.187885 | 1.88E-18 | 1.58E-17 | 31.05413038 |
| **ZNF117** | 0.857988 | 2.049136 | 9.186913 | 1.89E-18 | 1.59E-17 | 31.0467167 |
| **RIPK2** | 1.024738 | 2.781338 | 9.186749 | 1.89E-18 | 1.59E-17 | 31.04546571 |
| **GIT2** | 1.048764 | 2.909431 | 9.186138 | 1.90E-18 | 1.60E-17 | 31.04081167 |
| **TMEM97** | 1.007158 | 3.124142 | 9.185145 | 1.92E-18 | 1.61E-17 | 31.03324238 |
| **RAB2B** | 1.042042 | 3.130022 | 9.18489 | 1.92E-18 | 1.61E-17 | 31.03129415 |
| **CCDC58** | 1.200821 | 3.800869 | 9.183083 | 1.95E-18 | 1.64E-17 | 31.01752508 |
| **RRN3** | 1.134602 | 3.264617 | 9.182574 | 1.96E-18 | 1.64E-17 | 31.01364105 |
| **CSNK1E** | 1.387211 | 6.380051 | 9.181967 | 1.97E-18 | 1.65E-17 | 31.00901848 |
| **VEZT** | 1.039116 | 3.679154 | 9.18195 | 1.97E-18 | 1.65E-17 | 31.00889253 |
| **RP11-319G6.1** | 0.709587 | 1.724544 | 9.181637 | 1.97E-18 | 1.65E-17 | 31.00650523 |
| **CTD-2162K18.5** | 0.830459 | 2.496601 | 9.181193 | 1.98E-18 | 1.66E-17 | 31.00311893 |
| **BOD1** | 1.161271 | 4.623604 | 9.180445 | 1.99E-18 | 1.67E-17 | 30.99741752 |
| **FAM46A** | 0.722422 | 1.217147 | 9.179301 | 2.01E-18 | 1.68E-17 | 30.98870113 |
| **ZNF419** | 0.864253 | 2.679199 | 9.178877 | 2.01E-18 | 1.69E-17 | 30.98547288 |
| **GANC** | 0.915244 | 2.537538 | 9.178843 | 2.01E-18 | 1.69E-17 | 30.98521624 |
| **NUP85** | 1.05383 | 4.231263 | 9.178401 | 2.02E-18 | 1.69E-17 | 30.98184303 |
| **CSNK1G3** | 0.823295 | 2.320273 | 9.177433 | 2.04E-18 | 1.70E-17 | 30.9744705 |
| **ICMT** | 0.935322 | 3.280915 | 9.177388 | 2.04E-18 | 1.70E-17 | 30.9741282 |
| **GPR107** | 1.0311 | 3.505889 | 9.176844 | 2.04E-18 | 1.71E-17 | 30.96998777 |
| **CRTC3** | 0.959958 | 3.145842 | 9.176748 | 2.05E-18 | 1.71E-17 | 30.96925531 |
| **SLC16A3** | 1.191076 | 3.404938 | 9.175171 | 2.07E-18 | 1.73E-17 | 30.95724038 |
| **MON1B** | 1.075089 | 3.517601 | 9.174805 | 2.08E-18 | 1.73E-17 | 30.95445403 |
| **FHL3** | 0.940012 | 3.276765 | 9.174456 | 2.08E-18 | 1.74E-17 | 30.95179741 |
| **ARIH1** | 0.971335 | 3.025048 | 9.174428 | 2.08E-18 | 1.74E-17 | 30.95157834 |
| **AC084219.4** | 0.690549 | 1.28534 | 9.174332 | 2.09E-18 | 1.74E-17 | 30.95084756 |
| **C14orf119** | 1.049875 | 3.44074 | 9.174154 | 2.09E-18 | 1.74E-17 | 30.9494923 |
| **COPS2** | 1.197073 | 3.478251 | 9.173789 | 2.09E-18 | 1.75E-17 | 30.94671651 |
| **HIATL1** | 1.162013 | 3.176377 | 9.173714 | 2.10E-18 | 1.75E-17 | 30.94614222 |
| **SRFBP1** | 0.670543 | 1.388941 | 9.172234 | 2.12E-18 | 1.76E-17 | 30.93487045 |
| **TSPAN14** | 1.287731 | 3.842354 | 9.172203 | 2.12E-18 | 1.76E-17 | 30.93463405 |
| **PCGF6** | 0.761303 | 2.090468 | 9.17214 | 2.12E-18 | 1.77E-17 | 30.93415373 |
| **CERK** | 0.944751 | 3.60053 | 9.171788 | 2.13E-18 | 1.77E-17 | 30.93147825 |
| **SLC33A1** | 0.929127 | 2.18784 | 9.17175 | 2.13E-18 | 1.77E-17 | 30.93118275 |
| **SDC3** | 1.620417 | 6.431957 | 9.171534 | 2.13E-18 | 1.77E-17 | 30.92953696 |
| **HIF1A** | 1.391066 | 4.329997 | 9.171194 | 2.14E-18 | 1.78E-17 | 30.92695421 |
| **MPLKIP** | 0.597334 | 1.81633 | 9.171028 | 2.14E-18 | 1.78E-17 | 30.9256899 |
| **RNF115** | 0.914598 | 2.705584 | 9.17037 | 2.15E-18 | 1.79E-17 | 30.92067573 |
| **ZBTB26** | 0.744138 | 1.755614 | 9.169611 | 2.16E-18 | 1.80E-17 | 30.91489899 |
| **DPYSL3** | 1.413096 | 6.245139 | 9.169424 | 2.17E-18 | 1.80E-17 | 30.913477 |
| **CCDC59** | 1.19401 | 4.12554 | 9.167346 | 2.20E-18 | 1.83E-17 | 30.89765545 |
| **ABHD15** | 0.715771 | 1.730677 | 9.166055 | 2.22E-18 | 1.84E-17 | 30.88782678 |
| **FAM208A** | 1.0816 | 3.519617 | 9.16576 | 2.23E-18 | 1.85E-17 | 30.8855795 |
| **SNRNP200** | 1.353893 | 5.7369 | 9.164153 | 2.26E-18 | 1.87E-17 | 30.87335181 |
| **YIPF5** | 1.067707 | 2.721669 | 9.164004 | 2.26E-18 | 1.87E-17 | 30.87221164 |
| **YBX1P1** | 0.615756 | 1.005649 | 9.163684 | 2.26E-18 | 1.87E-17 | 30.86977678 |
| **TCF12** | 1.703043 | 4.945635 | 9.163642 | 2.26E-18 | 1.87E-17 | 30.86946199 |
| **EIF4EBP1** | 1.540313 | 5.415328 | 9.162385 | 2.29E-18 | 1.89E-17 | 30.85989255 |
| **GGPS1** | 1.014419 | 3.952779 | 9.162316 | 2.29E-18 | 1.89E-17 | 30.85936962 |
| **WWC3** | 0.761389 | 2.527028 | 9.161788 | 2.30E-18 | 1.90E-17 | 30.8553475 |
| **ADCY9** | 0.751105 | 1.869428 | 9.161384 | 2.30E-18 | 1.91E-17 | 30.85227772 |
| **AGO4** | 0.864266 | 2.235125 | 9.160533 | 2.32E-18 | 1.92E-17 | 30.84580137 |
| **ZNF19** | 0.68608 | 1.555513 | 9.15984 | 2.33E-18 | 1.93E-17 | 30.84052765 |
| **ADCK4** | 1.038742 | 4.616602 | 9.158472 | 2.36E-18 | 1.95E-17 | 30.83011867 |
| **HMGXB3** | 1.137354 | 3.050696 | 9.157748 | 2.37E-18 | 1.96E-17 | 30.82461455 |
| **C1GALT1C1** | 0.886739 | 2.695301 | 9.157345 | 2.38E-18 | 1.96E-17 | 30.82154292 |
| **IWS1** | 1.072109 | 3.686368 | 9.156834 | 2.39E-18 | 1.97E-17 | 30.81765485 |
| **CTDSP1** | 1.160856 | 5.088108 | 9.15683 | 2.39E-18 | 1.97E-17 | 30.81763097 |
| **7-Mar** | 1.304248 | 3.462245 | 9.156335 | 2.40E-18 | 1.98E-17 | 30.81386008 |
| **TSPAN31** | 1.40193 | 4.588206 | 9.156116 | 2.40E-18 | 1.98E-17 | 30.8121981 |
| **CCDC117** | 0.787753 | 1.999709 | 9.155623 | 2.41E-18 | 1.99E-17 | 30.80844326 |
| **STAU1** | 1.226473 | 4.242807 | 9.155313 | 2.41E-18 | 1.99E-17 | 30.80608522 |
| **TMEM248** | 1.12162 | 4.281786 | 9.154937 | 2.42E-18 | 1.99E-17 | 30.80322704 |
| **METTL23** | 1.12918 | 4.282105 | 9.154534 | 2.43E-18 | 2.00E-17 | 30.80016259 |
| **EDEM1** | 0.807543 | 1.758578 | 9.154375 | 2.43E-18 | 2.00E-17 | 30.79895128 |
| **BCL3** | 0.979615 | 2.420783 | 9.153923 | 2.44E-18 | 2.01E-17 | 30.79551919 |
| **ZNF773** | 0.912152 | 1.764416 | 9.153514 | 2.45E-18 | 2.01E-17 | 30.79240847 |
| **IPO5** | 1.226665 | 4.169792 | 9.152942 | 2.46E-18 | 2.02E-17 | 30.7880566 |
| **AGO2** | 0.852339 | 1.620921 | 9.152284 | 2.47E-18 | 2.03E-17 | 30.78305225 |
| **SUV39H1** | 0.67941 | 2.424821 | 9.152265 | 2.47E-18 | 2.03E-17 | 30.78290369 |
| **ZBED5** | 1.09689 | 3.810474 | 9.151911 | 2.48E-18 | 2.04E-17 | 30.78021572 |
| **HIC2** | 0.564585 | 1.496667 | 9.15182 | 2.48E-18 | 2.04E-17 | 30.77952304 |
| **DHX32** | 0.891878 | 2.895661 | 9.151148 | 2.49E-18 | 2.05E-17 | 30.77441185 |
| **MKNK2** | 1.168216 | 4.499404 | 9.151108 | 2.49E-18 | 2.05E-17 | 30.77410642 |
| **POLE** | 1.010273 | 3.404278 | 9.150588 | 2.50E-18 | 2.05E-17 | 30.77015281 |
| **EP400** | 0.885413 | 2.547132 | 9.150231 | 2.51E-18 | 2.06E-17 | 30.76744239 |
| **KRI1** | 1.024357 | 4.07426 | 9.149862 | 2.52E-18 | 2.06E-17 | 30.76463718 |
| **KIAA0247** | 1.07504 | 2.667868 | 9.14923 | 2.53E-18 | 2.07E-17 | 30.75983384 |
| **FBXO38** | 0.949804 | 2.938376 | 9.148338 | 2.55E-18 | 2.09E-17 | 30.75305245 |
| **RP5-874C20.3** | 0.88826 | 2.816281 | 9.147904 | 2.56E-18 | 2.09E-17 | 30.74974655 |
| **COQ5** | 1.055446 | 4.034357 | 9.147452 | 2.57E-18 | 2.10E-17 | 30.74631505 |
| **RANBP1** | 1.510605 | 6.188932 | 9.147301 | 2.57E-18 | 2.10E-17 | 30.74516456 |
| **PIBF1** | 0.718317 | 2.030637 | 9.146225 | 2.59E-18 | 2.12E-17 | 30.7369855 |
| **PKD2** | 1.110542 | 2.440187 | 9.144254 | 2.63E-18 | 2.15E-17 | 30.72200504 |
| **TLN1** | 1.337055 | 4.812197 | 9.144105 | 2.63E-18 | 2.15E-17 | 30.72087598 |
| **RHOBTB1** | 0.783702 | 1.999498 | 9.143279 | 2.65E-18 | 2.16E-17 | 30.71459792 |
| **PDE5A** | 0.627501 | 1.330207 | 9.140863 | 2.70E-18 | 2.20E-17 | 30.69623843 |
| **SPICE1** | 0.842487 | 1.457155 | 9.140541 | 2.71E-18 | 2.21E-17 | 30.69378712 |
| **RPAIN** | 1.204439 | 4.885624 | 9.140021 | 2.72E-18 | 2.22E-17 | 30.68983593 |
| **RINT1** | 0.929686 | 2.56652 | 9.139939 | 2.72E-18 | 2.22E-17 | 30.68921337 |
| **MSH3** | 0.699007 | 1.562292 | 9.139096 | 2.74E-18 | 2.23E-17 | 30.68281444 |
| **BMI1** | 1.29913 | 3.752976 | 9.138723 | 2.74E-18 | 2.24E-17 | 30.67997511 |
| **NAB2** | 1.077041 | 3.693902 | 9.138429 | 2.75E-18 | 2.24E-17 | 30.67774794 |
| **SLC40A1** | 1.20301 | 2.853111 | 9.137839 | 2.76E-18 | 2.25E-17 | 30.67325935 |
| **EXOC7** | 1.218661 | 5.2315 | 9.137561 | 2.77E-18 | 2.25E-17 | 30.67115092 |
| **SPDYE3** | 0.511819 | 1.091499 | 9.137517 | 2.77E-18 | 2.25E-17 | 30.67081443 |
| **UBE2E3** | 1.399335 | 5.121386 | 9.137316 | 2.77E-18 | 2.26E-17 | 30.66929174 |
| **DLG5** | 1.350792 | 3.625891 | 9.137052 | 2.78E-18 | 2.26E-17 | 30.66728419 |
| **HSD11B2** | 0.62333 | 1.149083 | 9.136804 | 2.79E-18 | 2.26E-17 | 30.66539748 |
| **ZNF330** | 1.088411 | 3.553099 | 9.13639 | 2.79E-18 | 2.27E-17 | 30.66225898 |
| **CRLF3** | 0.695259 | 1.875532 | 9.136278 | 2.80E-18 | 2.27E-17 | 30.66140774 |
| **TBC1D15** | 1.207192 | 3.375677 | 9.13619 | 2.80E-18 | 2.27E-17 | 30.66074017 |
| **ZFP36L1** | 1.577114 | 4.905661 | 9.135508 | 2.81E-18 | 2.28E-17 | 30.65555431 |
| **ARHGAP18** | 0.689824 | 1.310981 | 9.134805 | 2.83E-18 | 2.30E-17 | 30.6502183 |
| **RP11-262H14.1** | 0.830144 | 1.71427 | 9.134768 | 2.83E-18 | 2.30E-17 | 30.64993696 |
| **ST14** | 0.805939 | 1.25855 | 9.133382 | 2.86E-18 | 2.32E-17 | 30.63941263 |
| **FBXL20** | 0.765667 | 1.711594 | 9.133321 | 2.86E-18 | 2.32E-17 | 30.6389483 |
| **RNF44** | 1.268812 | 4.057636 | 9.132637 | 2.88E-18 | 2.33E-17 | 30.63375675 |
| **FPR3** | 0.80049 | 1.011825 | 9.132397 | 2.88E-18 | 2.34E-17 | 30.63193162 |
| **EXTL2** | 0.963568 | 2.711222 | 9.132157 | 2.89E-18 | 2.34E-17 | 30.63011024 |
| **GSPT2** | 0.922736 | 2.830251 | 9.132055 | 2.89E-18 | 2.34E-17 | 30.62933142 |
| **SUFU** | 0.770411 | 2.612822 | 9.131733 | 2.90E-18 | 2.34E-17 | 30.62688725 |
| **TSHZ1** | 1.108603 | 2.875955 | 9.131521 | 2.90E-18 | 2.35E-17 | 30.6252824 |
| **NGFR** | 1.340849 | 2.281603 | 9.131461 | 2.90E-18 | 2.35E-17 | 30.62482593 |
| **STAMBP** | 1.257085 | 4.407087 | 9.131364 | 2.90E-18 | 2.35E-17 | 30.62408867 |
| **ANKRD16** | 0.789898 | 2.785137 | 9.131281 | 2.91E-18 | 2.35E-17 | 30.62345795 |
| **PALD1** | 1.120198 | 3.485254 | 9.131244 | 2.91E-18 | 2.35E-17 | 30.62317908 |
| **ANXA5** | 1.732454 | 6.162068 | 9.130253 | 2.93E-18 | 2.37E-17 | 30.61564855 |
| **GJD3** | 0.563941 | 0.586928 | 9.129082 | 2.96E-18 | 2.39E-17 | 30.6067618 |
| **CS** | 1.296716 | 5.206154 | 9.128475 | 2.97E-18 | 2.40E-17 | 30.60215309 |
| **ITFG2** | 1.04136 | 4.222523 | 9.128124 | 2.98E-18 | 2.40E-17 | 30.59949019 |
| **MTDH** | 1.245854 | 3.495893 | 9.128121 | 2.98E-18 | 2.40E-17 | 30.59946325 |
| **TET2** | 0.75662 | 1.675579 | 9.127635 | 2.99E-18 | 2.41E-17 | 30.59577578 |
| **PIAS1** | 1.006384 | 2.426903 | 9.127543 | 2.99E-18 | 2.41E-17 | 30.59508072 |
| **SLC35D1** | 0.726404 | 1.349952 | 9.127134 | 3.00E-18 | 2.42E-17 | 30.59197017 |
| **FCHO2** | 0.869314 | 2.067364 | 9.127092 | 3.00E-18 | 2.42E-17 | 30.5916555 |
| **REEP3** | 1.057608 | 2.684854 | 9.125995 | 3.03E-18 | 2.44E-17 | 30.58333105 |
| **ATAD2B** | 0.660162 | 1.40121 | 9.125823 | 3.03E-18 | 2.44E-17 | 30.58202306 |
| **MRPS30** | 0.91139 | 3.254612 | 9.125658 | 3.04E-18 | 2.44E-17 | 30.5807715 |
| **ARHGEF40** | 1.180115 | 4.660677 | 9.124727 | 3.06E-18 | 2.46E-17 | 30.57370151 |
| **PDCD6IP** | 1.409047 | 4.30987 | 9.124667 | 3.06E-18 | 2.46E-17 | 30.57325037 |
| **C9orf41** | 0.598925 | 1.741051 | 9.124628 | 3.06E-18 | 2.46E-17 | 30.57295001 |
| **MTF1** | 0.677306 | 1.481429 | 9.124013 | 3.07E-18 | 2.47E-17 | 30.56828723 |
| **MED6** | 0.882623 | 3.265677 | 9.12252 | 3.11E-18 | 2.50E-17 | 30.55695883 |
| **ADAM17** | 1.219613 | 2.122816 | 9.122132 | 3.12E-18 | 2.50E-17 | 30.55401131 |
| **HSDL1** | 0.991058 | 3.805767 | 9.121082 | 3.14E-18 | 2.52E-17 | 30.54604363 |
| **ARMCX5** | 0.770537 | 1.844062 | 9.120793 | 3.15E-18 | 2.53E-17 | 30.54385318 |
| **RP11-163N6.2** | 0.721204 | 1.263282 | 9.120017 | 3.17E-18 | 2.54E-17 | 30.53796483 |
| **ERGIC2** | 1.269424 | 3.838196 | 9.119883 | 3.17E-18 | 2.54E-17 | 30.5369484 |
| **RP11-640M9.2** | 1.127099 | 2.005226 | 9.119764 | 3.18E-18 | 2.55E-17 | 30.53604981 |
| **GID4** | 0.696716 | 1.975418 | 9.118017 | 3.22E-18 | 2.58E-17 | 30.52279081 |
| **C2orf42** | 0.761243 | 2.773229 | 9.117877 | 3.22E-18 | 2.58E-17 | 30.52173097 |
| **TFCP2** | 1.06194 | 3.334898 | 9.117813 | 3.22E-18 | 2.58E-17 | 30.5212429 |
| **SRSF2** | 1.418836 | 5.812014 | 9.117607 | 3.23E-18 | 2.58E-17 | 30.51968104 |
| **ZNF862** | 0.985869 | 3.08871 | 9.117593 | 3.23E-18 | 2.58E-17 | 30.51957395 |
| **NAV1** | 1.044934 | 3.378466 | 9.116849 | 3.25E-18 | 2.60E-17 | 30.51393404 |
| **B4GALT1** | 0.788403 | 2.037258 | 9.116331 | 3.26E-18 | 2.61E-17 | 30.51000385 |
| **RFX1** | 0.813306 | 2.481224 | 9.116295 | 3.26E-18 | 2.61E-17 | 30.50973042 |
| **TM9SF2** | 1.219653 | 4.356438 | 9.115259 | 3.29E-18 | 2.63E-17 | 30.50187839 |
| **ARHGDIB** | 1.505411 | 5.244538 | 9.115009 | 3.29E-18 | 2.63E-17 | 30.49997779 |
| **ZNF316** | 1.242971 | 3.997049 | 9.114288 | 3.31E-18 | 2.65E-17 | 30.49451375 |
| **WBP5** | 1.463551 | 5.105168 | 9.113944 | 3.32E-18 | 2.65E-17 | 30.49190462 |
| **TRIM39** | 0.836305 | 2.686576 | 9.113802 | 3.33E-18 | 2.65E-17 | 30.49082387 |
| **SEC24B** | 0.922636 | 2.399194 | 9.113628 | 3.33E-18 | 2.66E-17 | 30.48950411 |
| **PSMD2** | 1.449954 | 6.377724 | 9.11362 | 3.33E-18 | 2.66E-17 | 30.48944675 |
| **ABCB10** | 0.707931 | 1.971394 | 9.112859 | 3.35E-18 | 2.67E-17 | 30.48367444 |
| **MAEA** | 1.136847 | 4.531914 | 9.112272 | 3.36E-18 | 2.68E-17 | 30.47922706 |
| **RPUSD4** | 0.937113 | 3.39471 | 9.110644 | 3.41E-18 | 2.72E-17 | 30.46688343 |
| **OSBPL8** | 1.131723 | 2.882457 | 9.110353 | 3.41E-18 | 2.72E-17 | 30.46467074 |
| **SART3** | 1.139484 | 3.885998 | 9.109911 | 3.43E-18 | 2.73E-17 | 30.46132557 |
| **MRPL44** | 0.93637 | 3.552788 | 9.109554 | 3.44E-18 | 2.73E-17 | 30.45862067 |
| **MTCH2** | 1.162399 | 4.686307 | 9.109523 | 3.44E-18 | 2.73E-17 | 30.45837948 |
| **INSR** | 0.787181 | 2.497831 | 9.109119 | 3.45E-18 | 2.74E-17 | 30.45531721 |
| **TRAF5** | 0.77528 | 1.669225 | 9.109068 | 3.45E-18 | 2.74E-17 | 30.45493631 |
| **HTRA3** | 0.856182 | 1.184497 | 9.108715 | 3.46E-18 | 2.75E-17 | 30.45225631 |
| **STAG2** | 1.182811 | 3.901671 | 9.108352 | 3.47E-18 | 2.76E-17 | 30.44950713 |
| **TRIM5** | 0.78488 | 1.859176 | 9.10799 | 3.48E-18 | 2.76E-17 | 30.44675832 |
| **CD97** | 0.963004 | 2.639953 | 9.107621 | 3.49E-18 | 2.77E-17 | 30.44396598 |
| **USPL1** | 0.911626 | 2.535761 | 9.107599 | 3.49E-18 | 2.77E-17 | 30.4437963 |
| **ISY1-RAB43** | 0.732636 | 1.200157 | 9.10693 | 3.51E-18 | 2.78E-17 | 30.43872984 |
| **IL17RA** | 0.714074 | 2.025391 | 9.106308 | 3.52E-18 | 2.80E-17 | 30.43400978 |
| **BANP** | 0.837477 | 3.367768 | 9.105216 | 3.55E-18 | 2.82E-17 | 30.42573751 |
| **SP110** | 0.893941 | 2.730165 | 9.105022 | 3.56E-18 | 2.82E-17 | 30.42427005 |
| **DEGS1** | 1.321691 | 4.729218 | 9.104849 | 3.56E-18 | 2.82E-17 | 30.42295934 |
| **ARHGEF19** | 0.58341 | 1.109387 | 9.104199 | 3.58E-18 | 2.84E-17 | 30.4180334 |
| **ACVR2A** | 0.677176 | 1.78088 | 9.102484 | 3.63E-18 | 2.87E-17 | 30.40503349 |
| **TMEM256-PLSCR3** | 1.098072 | 4.213125 | 9.101406 | 3.66E-18 | 2.90E-17 | 30.39686435 |
| **CD34** | 0.981004 | 2.829014 | 9.100994 | 3.67E-18 | 2.90E-17 | 30.39374711 |
| **TWF1** | 1.389014 | 4.080515 | 9.100481 | 3.68E-18 | 2.92E-17 | 30.38986036 |
| **ANKRD52** | 0.922144 | 3.102922 | 9.100105 | 3.69E-18 | 2.92E-17 | 30.38700914 |
| **TWSG1** | 1.081459 | 2.764126 | 9.100021 | 3.70E-18 | 2.92E-17 | 30.38637264 |
| **EEF2K** | 0.981715 | 2.60331 | 9.099611 | 3.71E-18 | 2.93E-17 | 30.38326961 |
| **ERCC6L2** | 1.084853 | 2.510323 | 9.099603 | 3.71E-18 | 2.93E-17 | 30.38321142 |
| **ABCD3** | 1.082396 | 3.438248 | 9.097935 | 3.76E-18 | 2.97E-17 | 30.37057613 |
| **MAP3K2** | 0.953636 | 2.395549 | 9.097508 | 3.77E-18 | 2.98E-17 | 30.36733579 |
| **LRRC37A4P** | 0.840536 | 2.14139 | 9.097105 | 3.78E-18 | 2.98E-17 | 30.36428329 |
| **NELFCD** | 1.278098 | 5.395063 | 9.096918 | 3.79E-18 | 2.99E-17 | 30.36286757 |
| **FCF1** | 1.112726 | 3.291413 | 9.096672 | 3.79E-18 | 2.99E-17 | 30.36100554 |
| **APOBEC3C** | 0.859015 | 1.826575 | 9.095732 | 3.82E-18 | 3.01E-17 | 30.35389033 |
| **MAP3K7CL** | 0.828818 | 1.446453 | 9.094096 | 3.87E-18 | 3.05E-17 | 30.34149695 |
| **PAPSS1** | 1.277142 | 4.459757 | 9.093467 | 3.89E-18 | 3.06E-17 | 30.33673342 |
| **MALT1** | 0.730927 | 1.695115 | 9.092539 | 3.92E-18 | 3.08E-17 | 30.32971006 |
| **SNX20** | 0.552496 | 0.923787 | 9.091835 | 3.94E-18 | 3.10E-17 | 30.32437794 |
| **KIAA1009** | 0.700678 | 1.623029 | 9.091407 | 3.95E-18 | 3.11E-17 | 30.3211425 |
| **TMEM57** | 0.823474 | 3.085658 | 9.091259 | 3.95E-18 | 3.11E-17 | 30.32002044 |
| **RPUSD2** | 0.75509 | 2.830085 | 9.090911 | 3.97E-18 | 3.12E-17 | 30.31738102 |
| **IQCC** | 0.56472 | 1.745004 | 9.090188 | 3.99E-18 | 3.13E-17 | 30.31191092 |
| **DYNC1LI1** | 1.039204 | 3.83913 | 9.089679 | 4.00E-18 | 3.15E-17 | 30.30806185 |
| **ZNF782** | 0.604346 | 1.162388 | 9.088752 | 4.03E-18 | 3.17E-17 | 30.30104127 |
| **CSNK2A2** | 1.019585 | 3.943353 | 9.088581 | 4.04E-18 | 3.17E-17 | 30.29974801 |
| **DCAF17** | 0.867134 | 1.990733 | 9.088381 | 4.04E-18 | 3.17E-17 | 30.29823024 |
| **NELFA** | 1.16301 | 4.572111 | 9.088325 | 4.04E-18 | 3.17E-17 | 30.29781076 |
| **CCDC71L** | 0.943157 | 2.438521 | 9.086669 | 4.10E-18 | 3.21E-17 | 30.28527828 |
| **STIP1** | 1.275257 | 5.575336 | 9.085081 | 4.15E-18 | 3.25E-17 | 30.27325866 |
| **PAG1** | 0.951736 | 1.905895 | 9.084832 | 4.15E-18 | 3.26E-17 | 30.27137409 |
| **SERINC2** | 1.028465 | 1.729467 | 9.084044 | 4.18E-18 | 3.28E-17 | 30.26541526 |
| **UNC119B** | 1.070384 | 3.517881 | 9.08285 | 4.22E-18 | 3.31E-17 | 30.25637374 |
| **LRPPRC** | 1.066147 | 4.013772 | 9.081951 | 4.25E-18 | 3.33E-17 | 30.24957411 |
| **PLEK2** | 0.528832 | 0.597946 | 9.081781 | 4.25E-18 | 3.33E-17 | 30.24829147 |
| **PNRC1** | 1.169722 | 4.211286 | 9.081714 | 4.26E-18 | 3.33E-17 | 30.24778132 |
| **RNF19B** | 0.948882 | 2.580863 | 9.081686 | 4.26E-18 | 3.33E-17 | 30.24757167 |
| **HSD3B7** | 0.799546 | 2.213743 | 9.081633 | 4.26E-18 | 3.33E-17 | 30.24717381 |
| **ABHD13** | 0.676943 | 1.620276 | 9.081526 | 4.26E-18 | 3.33E-17 | 30.24636022 |
| **SYAP1** | 0.771161 | 2.575291 | 9.081075 | 4.28E-18 | 3.34E-17 | 30.24295118 |
| **LIMD1-AS1** | 0.88546 | 1.752422 | 9.080981 | 4.28E-18 | 3.34E-17 | 30.24223801 |
| **MRS2** | 0.952153 | 3.010162 | 9.08096 | 4.28E-18 | 3.34E-17 | 30.24207847 |
| **BPGM** | 1.134049 | 3.83032 | 9.079998 | 4.31E-18 | 3.36E-17 | 30.23480498 |
| **CUL5** | 0.84097 | 2.697991 | 9.079993 | 4.31E-18 | 3.36E-17 | 30.23476414 |
| **GUSB** | 1.161459 | 4.644439 | 9.079826 | 4.32E-18 | 3.37E-17 | 30.23349682 |
| **LEPROT** | 1.124959 | 3.427589 | 9.079307 | 4.33E-18 | 3.38E-17 | 30.22957291 |
| **RBBP6** | 1.068943 | 4.272389 | 9.079072 | 4.34E-18 | 3.39E-17 | 30.22780052 |
| **GNPNAT1** | 0.846884 | 2.023134 | 9.078361 | 4.37E-18 | 3.40E-17 | 30.222423 |
| **ZNF318** | 0.848927 | 2.64923 | 9.078321 | 4.37E-18 | 3.40E-17 | 30.22211829 |
| **PXMP4** | 0.798603 | 2.10241 | 9.077548 | 4.39E-18 | 3.42E-17 | 30.21626696 |
| **ZADH2** | 0.988055 | 2.886554 | 9.077176 | 4.41E-18 | 3.43E-17 | 30.2134567 |
| **EIF4E** | 1.6062 | 3.877168 | 9.077172 | 4.41E-18 | 3.43E-17 | 30.21342476 |
| **FBXO42** | 0.915433 | 2.851489 | 9.076662 | 4.42E-18 | 3.44E-17 | 30.20956632 |
| **ZNF354A** | 0.84712 | 2.30002 | 9.076316 | 4.44E-18 | 3.45E-17 | 30.20695018 |
| **TARS** | 1.256184 | 4.951589 | 9.076312 | 4.44E-18 | 3.45E-17 | 30.20692068 |
| **UHRF2** | 1.032586 | 3.024925 | 9.076212 | 4.44E-18 | 3.45E-17 | 30.20617005 |
| **TMEM182** | 0.587987 | 1.300393 | 9.075563 | 4.46E-18 | 3.46E-17 | 30.20125641 |
| **CBX4** | 1.319634 | 4.137067 | 9.075416 | 4.47E-18 | 3.47E-17 | 30.20014926 |
| **OSER1** | 1.017751 | 3.824538 | 9.075091 | 4.48E-18 | 3.47E-17 | 30.19769224 |
| **NKAP** | 0.876972 | 3.173519 | 9.074007 | 4.51E-18 | 3.50E-17 | 30.18949253 |
| **SMAD5** | 0.923023 | 2.65684 | 9.07341 | 4.54E-18 | 3.52E-17 | 30.18498305 |
| **PLEKHA8P1** | 0.741615 | 1.232856 | 9.073112 | 4.55E-18 | 3.52E-17 | 30.18272684 |
| **HMGN2** | 1.699584 | 7.073683 | 9.073033 | 4.55E-18 | 3.53E-17 | 30.18213272 |
| **MARVELD1** | 0.914306 | 1.991385 | 9.072136 | 4.58E-18 | 3.55E-17 | 30.17534421 |
| **PREP** | 0.9412 | 2.872958 | 9.071658 | 4.60E-18 | 3.56E-17 | 30.17173185 |
| **IMMP2L** | 0.969534 | 3.076321 | 9.071026 | 4.62E-18 | 3.58E-17 | 30.16695624 |
| **VKORC1** | 1.356775 | 5.633854 | 9.070798 | 4.63E-18 | 3.58E-17 | 30.16523401 |
| **REV3L** | 1.014609 | 3.084796 | 9.070377 | 4.64E-18 | 3.59E-17 | 30.16205318 |
| **BGN** | 1.470435 | 4.639208 | 9.069023 | 4.69E-18 | 3.63E-17 | 30.15181339 |
| **FAM172A** | 0.924201 | 2.746528 | 9.068686 | 4.70E-18 | 3.64E-17 | 30.14926904 |
| **TBL1XR1** | 1.149093 | 3.430804 | 9.068358 | 4.71E-18 | 3.64E-17 | 30.14678997 |
| **NAB1** | 1.053956 | 3.347444 | 9.068086 | 4.72E-18 | 3.65E-17 | 30.14473795 |
| **RBM28** | 1.098413 | 3.790741 | 9.068072 | 4.73E-18 | 3.65E-17 | 30.14462573 |
| **GNG2** | 1.399636 | 4.315056 | 9.067881 | 4.73E-18 | 3.65E-17 | 30.14318366 |
| **NXT2** | 1.039875 | 2.693558 | 9.067802 | 4.73E-18 | 3.66E-17 | 30.14258852 |
| **AC034193.5** | 0.604601 | 1.20614 | 9.067343 | 4.75E-18 | 3.67E-17 | 30.13911913 |
| **PIGM** | 0.851925 | 1.825701 | 9.0667 | 4.77E-18 | 3.68E-17 | 30.13426076 |
| **ERCC4** | 0.693266 | 1.507295 | 9.066266 | 4.79E-18 | 3.69E-17 | 30.13098321 |
| **CCNYL1** | 0.78262 | 2.001124 | 9.06575 | 4.81E-18 | 3.71E-17 | 30.12708054 |
| **PAPOLA** | 1.222867 | 4.85138 | 9.065605 | 4.82E-18 | 3.71E-17 | 30.12598258 |
| **PYGO2** | 0.96927 | 3.703167 | 9.065334 | 4.83E-18 | 3.72E-17 | 30.12393657 |
| **BRAP** | 0.790909 | 2.928256 | 9.065163 | 4.83E-18 | 3.72E-17 | 30.12264389 |
| **PMPCB** | 1.313063 | 4.532569 | 9.065009 | 4.84E-18 | 3.72E-17 | 30.12147853 |
| **STRN4** | 1.183545 | 5.005764 | 9.064362 | 4.86E-18 | 3.74E-17 | 30.11659267 |
| **DDX46** | 1.198611 | 3.412436 | 9.063574 | 4.89E-18 | 3.76E-17 | 30.11063891 |
| **ARL4C** | 1.159817 | 3.082277 | 9.062843 | 4.92E-18 | 3.78E-17 | 30.10511753 |
| **ATF6** | 0.800468 | 2.376738 | 9.061608 | 4.97E-18 | 3.82E-17 | 30.09578826 |
| **TIMP2** | 1.591447 | 6.454959 | 9.061277 | 4.98E-18 | 3.83E-17 | 30.09328595 |
| **CDYL** | 0.818182 | 3.06136 | 9.059884 | 5.03E-18 | 3.87E-17 | 30.08276209 |
| **CNOT6L** | 0.691382 | 1.862914 | 9.059698 | 5.04E-18 | 3.87E-17 | 30.08135758 |
| **SF1** | 1.414868 | 6.203903 | 9.059538 | 5.04E-18 | 3.87E-17 | 30.08014986 |
| **ZNF420** | 0.738967 | 2.177243 | 9.059331 | 5.05E-18 | 3.88E-17 | 30.07858845 |
| **PPIL4** | 1.066881 | 3.060462 | 9.058839 | 5.07E-18 | 3.89E-17 | 30.07487494 |
| **PYROXD1** | 0.717456 | 2.044075 | 9.058827 | 5.07E-18 | 3.89E-17 | 30.07478068 |
| **SP4** | 0.721514 | 1.43548 | 9.058659 | 5.08E-18 | 3.90E-17 | 30.07351143 |
| **TSC22D2** | 0.935632 | 2.404991 | 9.058268 | 5.09E-18 | 3.91E-17 | 30.07056028 |
| **ARL4A** | 1.170718 | 3.695898 | 9.058024 | 5.10E-18 | 3.91E-17 | 30.06871926 |
| **SKIV2L2** | 1.139382 | 3.611834 | 9.057935 | 5.11E-18 | 3.91E-17 | 30.06804516 |
| **TRIM21** | 0.788981 | 2.235304 | 9.057399 | 5.13E-18 | 3.93E-17 | 30.06400041 |
| **TSFM** | 1.218634 | 4.351165 | 9.057373 | 5.13E-18 | 3.93E-17 | 30.0637975 |
| **TMEM63B** | 1.087248 | 4.227194 | 9.056623 | 5.16E-18 | 3.95E-17 | 30.05814075 |
| **SLC15A4** | 0.942709 | 2.948789 | 9.056499 | 5.16E-18 | 3.95E-17 | 30.05720076 |
| **CDC123** | 1.310864 | 5.230626 | 9.055344 | 5.21E-18 | 3.99E-17 | 30.04847872 |
| **DPY19L1** | 1.095991 | 2.794825 | 9.055115 | 5.22E-18 | 3.99E-17 | 30.04674789 |
| **HCFC1** | 1.028357 | 4.0672 | 9.054482 | 5.24E-18 | 4.01E-17 | 30.04197376 |
| **PCOLCE** | 1.299839 | 4.080249 | 9.054437 | 5.25E-18 | 4.01E-17 | 30.04163137 |
| **DTWD1** | 1.129343 | 3.306562 | 9.054225 | 5.25E-18 | 4.01E-17 | 30.04003083 |
| **PCNXL4** | 1.135116 | 3.581287 | 9.053985 | 5.26E-18 | 4.02E-17 | 30.03821642 |
| **TRNAU1AP** | 0.911598 | 3.540045 | 9.053478 | 5.28E-18 | 4.04E-17 | 30.03439319 |
| **FAM122A** | 0.872403 | 2.472551 | 9.053113 | 5.30E-18 | 4.05E-17 | 30.03163274 |
| **SNAP23** | 1.055939 | 3.729622 | 9.052278 | 5.33E-18 | 4.07E-17 | 30.02533026 |
| **MBD5** | 0.865371 | 2.399781 | 9.052206 | 5.34E-18 | 4.07E-17 | 30.02478784 |
| **ECHDC1** | 1.17892 | 4.296554 | 9.051636 | 5.36E-18 | 4.09E-17 | 30.02048337 |
| **ZIC1** | 1.35718 | 3.547186 | 9.051531 | 5.36E-18 | 4.09E-17 | 30.01969515 |
| **TFDP1** | 1.201002 | 4.464666 | 9.051431 | 5.37E-18 | 4.09E-17 | 30.01893787 |
| **GRSF1** | 1.177233 | 4.364496 | 9.051411 | 5.37E-18 | 4.09E-17 | 30.01878889 |
| **TRIP12** | 1.09487 | 3.607372 | 9.047876 | 5.52E-18 | 4.20E-17 | 29.99210661 |
| **NIN** | 0.872434 | 2.823168 | 9.047506 | 5.53E-18 | 4.21E-17 | 29.98931371 |
| **FCGR3A** | 1.463237 | 4.045347 | 9.046855 | 5.56E-18 | 4.23E-17 | 29.98440035 |
| **ZNF429** | 1.043784 | 2.791312 | 9.046766 | 5.56E-18 | 4.23E-17 | 29.98373055 |
| **DIS3** | 0.926205 | 2.352266 | 9.046133 | 5.59E-18 | 4.25E-17 | 29.97895158 |
| **RCSD1** | 0.905419 | 2.146646 | 9.045325 | 5.62E-18 | 4.28E-17 | 29.97285494 |
| **ZNF765** | 0.672447 | 1.265565 | 9.044494 | 5.66E-18 | 4.30E-17 | 29.96659189 |
| **TUBD1** | 0.658884 | 2.162324 | 9.043846 | 5.69E-18 | 4.32E-17 | 29.96169619 |
| **ATG12** | 1.124751 | 4.172525 | 9.043505 | 5.70E-18 | 4.33E-17 | 29.95913085 |
| **ZBTB21** | 0.682443 | 1.519746 | 9.043347 | 5.71E-18 | 4.34E-17 | 29.95793205 |
| **SSR2** | 1.458579 | 6.577232 | 9.04275 | 5.74E-18 | 4.35E-17 | 29.95343283 |
| **SCLY** | 0.824165 | 3.185005 | 9.042471 | 5.75E-18 | 4.36E-17 | 29.95132717 |
| **LATS1** | 0.788569 | 1.950577 | 9.041671 | 5.78E-18 | 4.39E-17 | 29.94529086 |
| **ACP2** | 1.093489 | 4.197537 | 9.04166 | 5.78E-18 | 4.39E-17 | 29.94521213 |
| **ESAM** | 1.120859 | 3.835269 | 9.040856 | 5.82E-18 | 4.41E-17 | 29.93914497 |
| **CRIPT** | 0.983326 | 3.5088 | 9.039501 | 5.88E-18 | 4.46E-17 | 29.92892622 |
| **PNN** | 1.303607 | 4.673422 | 9.039483 | 5.88E-18 | 4.46E-17 | 29.92878927 |
| **DFFA** | 0.896171 | 3.297358 | 9.039445 | 5.88E-18 | 4.46E-17 | 29.92850379 |
| **ZNRD1** | 1.141223 | 4.319931 | 9.039084 | 5.90E-18 | 4.47E-17 | 29.9257839 |
| **CTNNB1** | 1.466833 | 5.201467 | 9.038058 | 5.95E-18 | 4.50E-17 | 29.91804522 |
| **AKAP2** | 0.948506 | 1.966558 | 9.037258 | 5.98E-18 | 4.53E-17 | 29.91201027 |
| **PCDHB14** | 0.886226 | 2.176764 | 9.03711 | 5.99E-18 | 4.53E-17 | 29.91089491 |
| **TSHZ3** | 0.710015 | 1.645676 | 9.037097 | 5.99E-18 | 4.53E-17 | 29.91079832 |
| **SNRNP27** | 1.327332 | 4.312782 | 9.036832 | 6.00E-18 | 4.54E-17 | 29.90879759 |
| **PRR3** | 0.978315 | 3.686708 | 9.036131 | 6.03E-18 | 4.56E-17 | 29.90351674 |
| **KIF5B** | 1.245363 | 4.297837 | 9.035931 | 6.04E-18 | 4.57E-17 | 29.90200647 |
| **RAD17** | 0.924729 | 2.788703 | 9.035502 | 6.06E-18 | 4.58E-17 | 29.89877234 |
| **ZC3H14** | 0.986122 | 3.587953 | 9.035393 | 6.07E-18 | 4.58E-17 | 29.89794635 |
| **HNRNPM** | 1.397979 | 6.328526 | 9.03534 | 6.07E-18 | 4.58E-17 | 29.89754565 |
| **WBSCR16** | 1.024813 | 4.513425 | 9.035002 | 6.09E-18 | 4.59E-17 | 29.8950018 |
| **COPB1** | 1.290699 | 3.759373 | 9.034807 | 6.10E-18 | 4.60E-17 | 29.89352971 |
| **SH3RF3** | 0.946377 | 3.492664 | 9.034278 | 6.12E-18 | 4.61E-17 | 29.88954357 |
| **HMGB1** | 1.789973 | 7.163225 | 9.034259 | 6.12E-18 | 4.61E-17 | 29.88939759 |
| **DAG1** | 1.217905 | 4.315659 | 9.033587 | 6.15E-18 | 4.64E-17 | 29.88433161 |
| **BLOC1S6** | 1.178019 | 3.852922 | 9.032514 | 6.20E-18 | 4.67E-17 | 29.87624326 |
| **RP11-220I1.1** | 1.128919 | 3.314234 | 9.032136 | 6.22E-18 | 4.69E-17 | 29.87339446 |
| **NLGN4X** | 1.070949 | 2.352599 | 9.032049 | 6.23E-18 | 4.69E-17 | 29.87273649 |
| **FKBP9** | 1.167126 | 3.369823 | 9.031859 | 6.24E-18 | 4.69E-17 | 29.87131126 |
| **HLA-DRA** | 1.944697 | 5.370658 | 9.031792 | 6.24E-18 | 4.69E-17 | 29.87080198 |
| **BLOC1S3** | 0.72115 | 2.466371 | 9.031689 | 6.24E-18 | 4.70E-17 | 29.87002975 |
| **CD4** | 1.096214 | 3.305617 | 9.03057 | 6.30E-18 | 4.73E-17 | 29.86159185 |
| **UBE2D1** | 0.868038 | 2.782125 | 9.030374 | 6.31E-18 | 4.74E-17 | 29.86011214 |
| **SPON2** | 1.30405 | 2.782415 | 9.029664 | 6.34E-18 | 4.76E-17 | 29.85476348 |
| **SRSF11** | 1.639686 | 5.228849 | 9.02956 | 6.35E-18 | 4.77E-17 | 29.85398079 |
| **FLI1** | 0.923477 | 2.463972 | 9.028976 | 6.37E-18 | 4.79E-17 | 29.84958244 |
| **LINC00665** | 0.975967 | 3.573905 | 9.028328 | 6.41E-18 | 4.81E-17 | 29.84470044 |
| **PPP4R1L** | 0.613753 | 1.084988 | 9.028177 | 6.41E-18 | 4.81E-17 | 29.84355916 |
| **TIA1** | 1.400566 | 4.434981 | 9.028117 | 6.42E-18 | 4.81E-17 | 29.84311034 |
| **NRIP1** | 0.829851 | 1.815565 | 9.027875 | 6.43E-18 | 4.82E-17 | 29.84128043 |
| **P4HB** | 1.515441 | 7.043168 | 9.0277 | 6.44E-18 | 4.83E-17 | 29.83996547 |
| **ZFP91** | 1.131719 | 3.477267 | 9.027369 | 6.45E-18 | 4.84E-17 | 29.83747138 |
| **HNRNPA1** | 1.901658 | 8.643184 | 9.026908 | 6.48E-18 | 4.85E-17 | 29.83399915 |
| **XKR6** | 0.669864 | 1.662655 | 9.025613 | 6.54E-18 | 4.90E-17 | 29.82423654 |
| **TMX3** | 1.164701 | 3.200984 | 9.0254 | 6.55E-18 | 4.91E-17 | 29.82263207 |
| **SNW1** | 1.182 | 4.146238 | 9.024399 | 6.60E-18 | 4.94E-17 | 29.81509722 |
| **INVS** | 0.776592 | 1.751343 | 9.02439 | 6.60E-18 | 4.94E-17 | 29.81502307 |
| **CRKL** | 1.182928 | 4.050379 | 9.024311 | 6.61E-18 | 4.94E-17 | 29.81443129 |
| **TTC13** | 0.880773 | 2.969471 | 9.023511 | 6.65E-18 | 4.97E-17 | 29.80840291 |
| **SUMO2** | 1.638912 | 6.53782 | 9.023255 | 6.66E-18 | 4.98E-17 | 29.80647372 |
| **HERPUD2** | 0.92515 | 3.074387 | 9.022691 | 6.69E-18 | 5.00E-17 | 29.80222828 |
| **ELP3** | 0.972599 | 3.994681 | 9.021734 | 6.74E-18 | 5.03E-17 | 29.79501712 |
| **USP14** | 1.030638 | 3.169027 | 9.021303 | 6.76E-18 | 5.05E-17 | 29.79177471 |
| **LASP1** | 1.202424 | 4.881694 | 9.021286 | 6.76E-18 | 5.05E-17 | 29.79164399 |
| **ZNF136** | 0.776712 | 1.637854 | 9.0202 | 6.82E-18 | 5.09E-17 | 29.78346907 |
| **HNRNPH3** | 1.590613 | 5.910877 | 9.019888 | 6.83E-18 | 5.10E-17 | 29.78111586 |
| **FBRSL1** | 1.396544 | 5.123718 | 9.019791 | 6.84E-18 | 5.10E-17 | 29.78038599 |
| **SLC16A2** | 1.056227 | 3.0686 | 9.019467 | 6.86E-18 | 5.11E-17 | 29.77794686 |
| **AP1G1** | 1.129714 | 3.357857 | 9.01906 | 6.88E-18 | 5.13E-17 | 29.77487997 |
| **VSTM4** | 0.714438 | 1.796358 | 9.017474 | 6.96E-18 | 5.19E-17 | 29.76294215 |
| **TMEM168** | 1.054177 | 2.321425 | 9.017415 | 6.96E-18 | 5.19E-17 | 29.76249479 |
| **ZNF287** | 0.765374 | 2.522064 | 9.016832 | 6.99E-18 | 5.21E-17 | 29.75810271 |
| **COL1A1** | 1.539136 | 2.246543 | 9.016812 | 7.00E-18 | 5.21E-17 | 29.75795859 |
| **TOR1AIP2** | 0.772445 | 2.907731 | 9.01673 | 7.00E-18 | 5.21E-17 | 29.75733473 |
| **MNT** | 0.977729 | 3.035805 | 9.015846 | 7.05E-18 | 5.24E-17 | 29.75068491 |
| **UBA2** | 1.231806 | 4.512055 | 9.015346 | 7.07E-18 | 5.26E-17 | 29.74692093 |
| **ZMYND19** | 1.090964 | 4.383204 | 9.01532 | 7.08E-18 | 5.26E-17 | 29.74671951 |
| **MAGT1** | 1.031375 | 3.081419 | 9.015017 | 7.09E-18 | 5.27E-17 | 29.74444547 |
| **RP11-295G20.2** | 0.697717 | 1.829766 | 9.015002 | 7.09E-18 | 5.27E-17 | 29.74432921 |
| **TPM3** | 1.362131 | 6.189768 | 9.014487 | 7.12E-18 | 5.29E-17 | 29.74045313 |
| **FUBP3** | 1.326389 | 4.458586 | 9.014392 | 7.13E-18 | 5.29E-17 | 29.73973728 |
| **CHST12** | 1.212638 | 3.948475 | 9.014294 | 7.13E-18 | 5.30E-17 | 29.73900117 |
| **HEATR5A** | 0.889105 | 3.260278 | 9.013936 | 7.15E-18 | 5.31E-17 | 29.73630299 |
| **RBM17** | 1.324615 | 5.212959 | 9.01258 | 7.23E-18 | 5.36E-17 | 29.72610161 |
| **FAM32A** | 1.263119 | 5.302306 | 9.011263 | 7.30E-18 | 5.41E-17 | 29.71618286 |
| **MIER1** | 0.893753 | 2.379671 | 9.009704 | 7.39E-18 | 5.48E-17 | 29.70445043 |
| **TMEM50A** | 1.31369 | 4.936978 | 9.009621 | 7.39E-18 | 5.48E-17 | 29.70382636 |
| **S100A11** | 1.68501 | 5.756728 | 9.009527 | 7.40E-18 | 5.48E-17 | 29.70312455 |
| **DDR1** | 1.541483 | 7.349603 | 9.008683 | 7.44E-18 | 5.52E-17 | 29.69677095 |
| **HECTD3** | 0.970036 | 3.718977 | 9.008227 | 7.47E-18 | 5.53E-17 | 29.69334163 |
| **LYZ** | 1.294063 | 1.913963 | 9.007578 | 7.51E-18 | 5.56E-17 | 29.68845721 |
| **YPEL1** | 0.903691 | 2.614001 | 9.007198 | 7.53E-18 | 5.57E-17 | 29.68559445 |
| **MEAF6** | 1.312735 | 4.731833 | 9.007065 | 7.54E-18 | 5.58E-17 | 29.6845972 |
| **MIER2** | 0.937352 | 3.659867 | 9.006984 | 7.54E-18 | 5.58E-17 | 29.68398897 |
| **LCP1** | 0.987338 | 2.586027 | 9.00638 | 7.58E-18 | 5.60E-17 | 29.67944417 |
| **SEC24C** | 1.102295 | 4.512692 | 9.006255 | 7.58E-18 | 5.61E-17 | 29.67849991 |
| **FOXD2-AS1** | 0.690628 | 0.765616 | 9.005856 | 7.61E-18 | 5.62E-17 | 29.67550467 |
| **PCDHB16** | 0.76292 | 1.411235 | 9.005536 | 7.62E-18 | 5.63E-17 | 29.67309579 |
| **SNX33** | 0.736035 | 2.572052 | 9.005042 | 7.65E-18 | 5.65E-17 | 29.66937999 |
| **RAP2C** | 0.993232 | 2.534389 | 9.003643 | 7.74E-18 | 5.71E-17 | 29.65885537 |
| **CDC42** | 1.513686 | 5.857493 | 9.002433 | 7.81E-18 | 5.76E-17 | 29.64975732 |
| **RBM48** | 0.928515 | 2.978089 | 9.002286 | 7.82E-18 | 5.77E-17 | 29.64864873 |
| **DSE** | 0.842022 | 1.633777 | 9.001973 | 7.84E-18 | 5.78E-17 | 29.64629617 |
| **ST8SIA1** | 1.039645 | 3.543045 | 9.001861 | 7.84E-18 | 5.78E-17 | 29.64545225 |
| **PARP14** | 0.892293 | 2.11464 | 9.001038 | 7.89E-18 | 5.82E-17 | 29.6392628 |
| **ARID5A** | 1.106681 | 3.912152 | 9.000944 | 7.90E-18 | 5.82E-17 | 29.63855938 |
| **CCDC14** | 1.239578 | 3.861567 | 9.000797 | 7.91E-18 | 5.82E-17 | 29.63744781 |
| **PWWP2A** | 0.790558 | 2.302006 | 9.000735 | 7.91E-18 | 5.82E-17 | 29.63698857 |
| **AGFG1** | 1.184215 | 3.527063 | 9.000496 | 7.92E-18 | 5.83E-17 | 29.63518634 |
| **AMOTL2** | 1.287119 | 3.800472 | 8.999554 | 7.98E-18 | 5.87E-17 | 29.62810662 |
| **CCDC47** | 1.364671 | 4.571096 | 8.999017 | 8.01E-18 | 5.89E-17 | 29.62406276 |
| **ZNF24** | 1.039023 | 3.787705 | 8.998997 | 8.02E-18 | 5.89E-17 | 29.62391718 |
| **TFAP4** | 0.899362 | 2.858577 | 8.998988 | 8.02E-18 | 5.89E-17 | 29.62384978 |
| **U2SURP** | 1.269492 | 3.875408 | 8.998806 | 8.03E-18 | 5.90E-17 | 29.62247925 |
| **IRF9** | 1.389962 | 5.593757 | 8.998391 | 8.05E-18 | 5.92E-17 | 29.61935596 |
| **BAX** | 1.304106 | 6.299302 | 8.998145 | 8.07E-18 | 5.93E-17 | 29.61750968 |
| **ASCL1** | 1.430204 | 4.18422 | 8.996687 | 8.16E-18 | 5.99E-17 | 29.60654581 |
| **NUPL1** | 1.038697 | 2.968408 | 8.996403 | 8.18E-18 | 6.00E-17 | 29.60441634 |
| **MRPL3** | 1.354471 | 4.564986 | 8.996355 | 8.18E-18 | 6.00E-17 | 29.60405404 |
| **IST1** | 1.330827 | 5.982983 | 8.995988 | 8.20E-18 | 6.02E-17 | 29.60129311 |
| **ANGEL2** | 1.081055 | 3.504731 | 8.995978 | 8.20E-18 | 6.02E-17 | 29.60121558 |
| **INTS4** | 0.948015 | 3.75592 | 8.995926 | 8.21E-18 | 6.02E-17 | 29.60082631 |
| **TM2D2** | 1.106964 | 4.430124 | 8.994114 | 8.32E-18 | 6.10E-17 | 29.58720874 |
| **SUPT20H** | 1.006404 | 3.631813 | 8.993859 | 8.34E-18 | 6.11E-17 | 29.58529395 |
| **IPO7** | 1.235331 | 3.876673 | 8.99299 | 8.39E-18 | 6.15E-17 | 29.57876269 |
| **SRSF4** | 1.373722 | 4.778659 | 8.992815 | 8.40E-18 | 6.15E-17 | 29.57744801 |
| **GXYLT1** | 0.620928 | 1.306573 | 8.992639 | 8.41E-18 | 6.16E-17 | 29.57612561 |
| **FAM206A** | 0.885411 | 2.907512 | 8.992627 | 8.41E-18 | 6.16E-17 | 29.57603267 |
| **AQR** | 0.889308 | 2.327066 | 8.992327 | 8.43E-18 | 6.17E-17 | 29.57377959 |
| **BCHE** | 1.52128 | 4.51406 | 8.99214 | 8.45E-18 | 6.18E-17 | 29.572371 |
| **FIZ1** | 0.954315 | 3.61235 | 8.989772 | 8.60E-18 | 6.29E-17 | 29.55458081 |
| **ZMYM5** | 0.774918 | 1.974995 | 8.989584 | 8.61E-18 | 6.30E-17 | 29.55316775 |
| **TNPO3** | 1.255383 | 2.769973 | 8.989508 | 8.62E-18 | 6.30E-17 | 29.55259415 |
| **COL6A2** | 1.476642 | 3.759329 | 8.989311 | 8.63E-18 | 6.30E-17 | 29.5511145 |
| **RP11-464F9.20** | 0.626931 | 1.368695 | 8.989265 | 8.63E-18 | 6.31E-17 | 29.55076956 |
| **ZNF324B** | 0.637591 | 1.558746 | 8.988333 | 8.69E-18 | 6.35E-17 | 29.5437693 |
| **TAF8** | 0.747228 | 2.816036 | 8.987451 | 8.75E-18 | 6.39E-17 | 29.53713809 |
| **DHX8** | 0.964239 | 3.974807 | 8.986566 | 8.81E-18 | 6.43E-17 | 29.53048976 |
| **TBC1D7** | 0.999709 | 3.849627 | 8.985859 | 8.86E-18 | 6.46E-17 | 29.52517773 |
| **TMEM60** | 0.991587 | 3.646869 | 8.985036 | 8.92E-18 | 6.50E-17 | 29.51900025 |
| **HMG20B** | 1.377648 | 5.852858 | 8.98423 | 8.97E-18 | 6.54E-17 | 29.51294179 |
| **STIM2** | 1.005916 | 3.613782 | 8.984029 | 8.98E-18 | 6.55E-17 | 29.51143209 |
| **TNFAIP6** | 0.774569 | 0.936852 | 8.983845 | 9.00E-18 | 6.55E-17 | 29.51005518 |
| **NDUFAF7** | 0.80091 | 2.620831 | 8.983825 | 9.00E-18 | 6.55E-17 | 29.50990255 |
| **CLEC14A** | 0.849682 | 2.387562 | 8.983204 | 9.04E-18 | 6.58E-17 | 29.5052385 |
| **LYRM2** | 1.062326 | 3.535864 | 8.982727 | 9.07E-18 | 6.61E-17 | 29.50165939 |
| **KDM4B** | 1.02194 | 4.072641 | 8.98268 | 9.08E-18 | 6.61E-17 | 29.50130543 |
| **DDX55** | 1.048538 | 3.382021 | 8.982539 | 9.09E-18 | 6.61E-17 | 29.50024643 |
| **ZNF197** | 0.878364 | 2.817736 | 8.981867 | 9.13E-18 | 6.64E-17 | 29.495196 |
| **ZNF585B** | 0.826199 | 2.003621 | 8.981693 | 9.15E-18 | 6.65E-17 | 29.49389054 |
| **MARCKS** | 1.466377 | 6.501258 | 8.980251 | 9.25E-18 | 6.72E-17 | 29.48306133 |
| **WDR55** | 0.891301 | 3.17616 | 8.978535 | 9.37E-18 | 6.81E-17 | 29.47018046 |
| **LOX** | 0.596292 | 0.841197 | 8.977945 | 9.41E-18 | 6.84E-17 | 29.46574868 |
| **TMEM164** | 1.035132 | 3.085488 | 8.977026 | 9.48E-18 | 6.88E-17 | 29.45885207 |
| **FDX1** | 0.827656 | 2.802335 | 8.976678 | 9.50E-18 | 6.90E-17 | 29.45624132 |
| **BIVM** | 1.013058 | 3.249575 | 8.976247 | 9.53E-18 | 6.92E-17 | 29.45300577 |
| **AARS2** | 0.752449 | 2.35528 | 8.975719 | 9.57E-18 | 6.94E-17 | 29.44904189 |
| **PHKB** | 0.977941 | 4.000131 | 8.97494 | 9.63E-18 | 6.98E-17 | 29.44319684 |
| **RALGAPB** | 0.926082 | 2.646781 | 8.974809 | 9.64E-18 | 6.99E-17 | 29.44221481 |
| **TMEM99** | 0.823807 | 2.847881 | 8.974516 | 9.66E-18 | 7.00E-17 | 29.44001152 |
| **AP5B1** | 0.697301 | 1.8003 | 8.973025 | 9.77E-18 | 7.08E-17 | 29.42882606 |
| **ISY1** | 0.931608 | 3.775465 | 8.97292 | 9.78E-18 | 7.08E-17 | 29.4280348 |
| **ALMS1** | 0.85 | 2.593582 | 8.972805 | 9.79E-18 | 7.09E-17 | 29.42717152 |
| **EHD1** | 1.227905 | 4.956699 | 8.972012 | 9.85E-18 | 7.13E-17 | 29.42122368 |
| **TRIM69** | 1.128387 | 4.065436 | 8.971764 | 9.86E-18 | 7.14E-17 | 29.41935961 |
| **15-Sep** | 1.473748 | 5.270606 | 8.970985 | 9.92E-18 | 7.18E-17 | 29.41351696 |
| **GRK5** | 0.831603 | 2.489403 | 8.969961 | 1.00E-17 | 7.23E-17 | 29.40583165 |
| **LMAN2** | 1.293459 | 5.822676 | 8.969206 | 1.01E-17 | 7.27E-17 | 29.40017154 |
| **PRKAB1** | 0.982029 | 3.976507 | 8.968889 | 1.01E-17 | 7.29E-17 | 29.39779274 |
| **PHF2** | 1.173526 | 3.759936 | 8.968747 | 1.01E-17 | 7.29E-17 | 29.3967259 |
| **SETD2** | 0.932868 | 2.773664 | 8.96859 | 1.01E-17 | 7.30E-17 | 29.39554509 |
| **NGLY1** | 0.90436 | 3.448795 | 8.968383 | 1.01E-17 | 7.31E-17 | 29.39399302 |
| **NASP** | 1.394544 | 5.7753 | 8.967443 | 1.02E-17 | 7.36E-17 | 29.3869467 |
| **BROX** | 1.033196 | 3.740139 | 8.966561 | 1.03E-17 | 7.41E-17 | 29.38032401 |
| **COMMD8** | 0.919781 | 2.811701 | 8.966417 | 1.03E-17 | 7.41E-17 | 29.3792472 |
| **DDX3X** | 1.463092 | 4.356311 | 8.966244 | 1.03E-17 | 7.42E-17 | 29.37794782 |
| **RRS1** | 1.015654 | 3.587395 | 8.966036 | 1.03E-17 | 7.43E-17 | 29.37639043 |
| **UTP23** | 1.09756 | 2.66901 | 8.963137 | 1.05E-17 | 7.59E-17 | 29.35465054 |
| **MRPS35** | 0.977467 | 4.096235 | 8.963122 | 1.05E-17 | 7.59E-17 | 29.35453775 |
| **MSANTD2** | 0.884441 | 2.495943 | 8.963098 | 1.05E-17 | 7.59E-17 | 29.35435416 |
| **COL5A2** | 1.03526 | 1.791834 | 8.963055 | 1.05E-17 | 7.59E-17 | 29.35403563 |
| **TBCE** | 0.849635 | 3.142059 | 8.962776 | 1.06E-17 | 7.60E-17 | 29.35194165 |
| **POLI** | 0.942196 | 2.912035 | 8.962633 | 1.06E-17 | 7.61E-17 | 29.35086622 |
| **CASC7** | 0.841614 | 2.185402 | 8.961877 | 1.06E-17 | 7.65E-17 | 29.34520455 |
| **ZCCHC6** | 0.697394 | 2.342217 | 8.961559 | 1.07E-17 | 7.67E-17 | 29.34281529 |
| **EXOC2** | 0.928134 | 2.846036 | 8.961086 | 1.07E-17 | 7.69E-17 | 29.33926819 |
| **METTL6** | 0.797936 | 3.368051 | 8.960276 | 1.08E-17 | 7.74E-17 | 29.33320066 |
| **TTC5** | 0.875902 | 2.188547 | 8.960046 | 1.08E-17 | 7.75E-17 | 29.33147038 |
| **KHDRBS3** | 1.303526 | 5.288681 | 8.95914 | 1.09E-17 | 7.80E-17 | 29.32467743 |
| **EP300** | 1.054422 | 2.337352 | 8.958667 | 1.09E-17 | 7.83E-17 | 29.3211366 |
| **ZNF14** | 0.566959 | 1.526236 | 8.95858 | 1.09E-17 | 7.83E-17 | 29.32048112 |
| **FARP2** | 0.890335 | 3.004487 | 8.957545 | 1.10E-17 | 7.89E-17 | 29.31272211 |
| **GBP1** | 1.241963 | 2.344128 | 8.956847 | 1.11E-17 | 7.93E-17 | 29.3074907 |
| **NSMAF** | 1.02941 | 3.315748 | 8.956615 | 1.11E-17 | 7.94E-17 | 29.30575628 |
| **SPTLC1** | 1.334472 | 3.812019 | 8.956478 | 1.11E-17 | 7.95E-17 | 29.30472954 |
| **SF3A1** | 1.343184 | 4.444657 | 8.956341 | 1.11E-17 | 7.95E-17 | 29.30369791 |
| **SOD2** | 1.571413 | 5.566907 | 8.956187 | 1.11E-17 | 7.96E-17 | 29.30254461 |
| **TMLHE** | 0.836133 | 2.213564 | 8.95561 | 1.12E-17 | 7.99E-17 | 29.29822043 |
| **ZNF655** | 1.181588 | 3.972877 | 8.955023 | 1.12E-17 | 8.03E-17 | 29.2938238 |
| **TPP2** | 1.141993 | 3.270169 | 8.954557 | 1.12E-17 | 8.05E-17 | 29.29033224 |
| **C12orf65** | 1.010307 | 3.85179 | 8.954301 | 1.13E-17 | 8.06E-17 | 29.2884154 |
| **ZNF304** | 0.723125 | 1.645331 | 8.95429 | 1.13E-17 | 8.06E-17 | 29.28832762 |
| **UBE3B** | 0.915202 | 3.096757 | 8.954111 | 1.13E-17 | 8.07E-17 | 29.2869902 |
| **SLC25A15** | 0.824409 | 1.52526 | 8.952615 | 1.14E-17 | 8.16E-17 | 29.27578239 |
| **PLCG1** | 1.334732 | 5.121128 | 8.952254 | 1.14E-17 | 8.18E-17 | 29.2730704 |
| **PPP6C** | 1.014279 | 3.516755 | 8.951563 | 1.15E-17 | 8.22E-17 | 29.26789906 |
| **PRKAA1** | 1.105007 | 2.971655 | 8.951002 | 1.16E-17 | 8.25E-17 | 29.26369338 |
| **H3F3A** | 1.881992 | 7.837204 | 8.95088 | 1.16E-17 | 8.26E-17 | 29.26277792 |
| **EPSTI1** | 0.696369 | 1.324761 | 8.950532 | 1.16E-17 | 8.28E-17 | 29.26017152 |
| **FBXO11** | 1.193701 | 3.827406 | 8.950368 | 1.16E-17 | 8.29E-17 | 29.25894324 |
| **TRRAP** | 0.94541 | 2.263127 | 8.949749 | 1.17E-17 | 8.32E-17 | 29.25430944 |
| **TMEM41A** | 0.704012 | 2.593607 | 8.94953 | 1.17E-17 | 8.34E-17 | 29.25266859 |
| **ZNF512B** | 0.938932 | 3.420433 | 8.948732 | 1.18E-17 | 8.38E-17 | 29.24668515 |
| **CTB-131K11.1** | 1.066274 | 2.22923 | 8.947569 | 1.19E-17 | 8.45E-17 | 29.23798065 |
| **LINC00526** | 0.799253 | 2.327382 | 8.947545 | 1.19E-17 | 8.45E-17 | 29.23780061 |
| **TAF11** | 0.958315 | 3.905387 | 8.947331 | 1.19E-17 | 8.47E-17 | 29.23619519 |
| **IKBKB** | 1.019985 | 4.088513 | 8.947084 | 1.19E-17 | 8.48E-17 | 29.23434355 |
| **GTF2IRD2B** | 1.048454 | 4.003257 | 8.947074 | 1.19E-17 | 8.48E-17 | 29.23426728 |
| **PRR12** | 0.921636 | 2.95565 | 8.946812 | 1.19E-17 | 8.49E-17 | 29.23230539 |
| **SPRED2** | 1.20751 | 2.937088 | 8.946303 | 1.20E-17 | 8.52E-17 | 29.22849805 |
| **DIP2B** | 1.080037 | 3.09305 | 8.946277 | 1.20E-17 | 8.52E-17 | 29.22829981 |
| **KPNA1** | 1.061643 | 3.118027 | 8.946066 | 1.20E-17 | 8.53E-17 | 29.22672149 |
| **KIAA1033** | 1.06336 | 2.763978 | 8.945578 | 1.20E-17 | 8.56E-17 | 29.22306607 |
| **SLC7A6OS** | 0.858796 | 3.007501 | 8.945165 | 1.21E-17 | 8.59E-17 | 29.21997177 |
| **MAN2B1** | 1.181424 | 4.499247 | 8.944753 | 1.21E-17 | 8.61E-17 | 29.21688615 |
| **MYL12A** | 1.432833 | 5.067211 | 8.943881 | 1.22E-17 | 8.66E-17 | 29.21035735 |
| **EGR1** | 1.707731 | 4.242786 | 8.943745 | 1.22E-17 | 8.67E-17 | 29.20933811 |
| **OSGEPL1** | 0.871013 | 2.549416 | 8.943409 | 1.22E-17 | 8.69E-17 | 29.20682106 |
| **CMTR1** | 1.012809 | 4.045872 | 8.942989 | 1.23E-17 | 8.72E-17 | 29.20368292 |
| **DNMBP** | 0.752324 | 1.892411 | 8.942884 | 1.23E-17 | 8.72E-17 | 29.20289285 |
| **SNTB2** | 0.685579 | 1.441799 | 8.942834 | 1.23E-17 | 8.72E-17 | 29.20251707 |
| **AADAT** | 0.92227 | 2.857683 | 8.941455 | 1.24E-17 | 8.81E-17 | 29.19219651 |
| **PTCD3** | 1.288364 | 3.950315 | 8.941453 | 1.24E-17 | 8.81E-17 | 29.19217958 |
| **TNFRSF21** | 1.333915 | 4.334345 | 8.941213 | 1.24E-17 | 8.82E-17 | 29.19037939 |
| **WRAP73** | 0.951809 | 3.457806 | 8.941121 | 1.25E-17 | 8.83E-17 | 29.18969597 |
| **APPBP2** | 1.047188 | 2.91881 | 8.940453 | 1.25E-17 | 8.87E-17 | 29.18469136 |
| **TPR** | 1.202182 | 3.539766 | 8.940442 | 1.25E-17 | 8.87E-17 | 29.18461199 |
| **ARMC7** | 0.795528 | 3.049903 | 8.938902 | 1.27E-17 | 8.97E-17 | 29.17308252 |
| **KCTD9** | 0.847768 | 1.860399 | 8.938518 | 1.27E-17 | 8.99E-17 | 29.17020587 |
| **MAGOH** | 1.269012 | 4.808795 | 8.938277 | 1.27E-17 | 9.00E-17 | 29.16840402 |
| **ECD** | 0.888639 | 2.704375 | 8.937923 | 1.28E-17 | 9.03E-17 | 29.16575957 |
| **USP48** | 1.022725 | 3.319961 | 8.937123 | 1.28E-17 | 9.08E-17 | 29.15976723 |
| **PCBP2** | 1.65378 | 7.241076 | 8.93692 | 1.29E-17 | 9.09E-17 | 29.15825267 |
| **WDFY1** | 0.998481 | 2.804454 | 8.936915 | 1.29E-17 | 9.09E-17 | 29.15821365 |
| **SELRC1** | 0.857831 | 2.327064 | 8.936818 | 1.29E-17 | 9.09E-17 | 29.15748758 |
| **CITED2** | 1.191646 | 3.450833 | 8.936454 | 1.29E-17 | 9.11E-17 | 29.15476003 |
| **PDXDC1** | 1.080415 | 3.38806 | 8.93623 | 1.29E-17 | 9.13E-17 | 29.15308835 |
| **RIOK3** | 0.974346 | 3.539334 | 8.936145 | 1.29E-17 | 9.13E-17 | 29.15244649 |
| **PIGN** | 0.906032 | 1.942343 | 8.934875 | 1.31E-17 | 9.22E-17 | 29.1429463 |
| **CAST** | 1.106542 | 3.503945 | 8.93412 | 1.31E-17 | 9.27E-17 | 29.1372958 |
| **RAD51D** | 0.805927 | 2.968988 | 8.933476 | 1.32E-17 | 9.31E-17 | 29.13248181 |
| **FGD5** | 0.637286 | 1.290724 | 8.933207 | 1.32E-17 | 9.33E-17 | 29.13046279 |
| **SUSD2** | 0.67949 | 1.334078 | 8.931727 | 1.34E-17 | 9.43E-17 | 29.11939591 |
| **ZNF445** | 0.85009 | 2.107015 | 8.93132 | 1.34E-17 | 9.45E-17 | 29.11635257 |
| **CWC25** | 0.901092 | 2.865545 | 8.930855 | 1.35E-17 | 9.48E-17 | 29.11286697 |
| **RNF114** | 1.189 | 4.666767 | 8.930176 | 1.35E-17 | 9.53E-17 | 29.1077942 |
| **CCT8** | 1.34907 | 5.25919 | 8.929922 | 1.36E-17 | 9.55E-17 | 29.10588969 |
| **SNRPA** | 1.251728 | 5.023 | 8.929661 | 1.36E-17 | 9.56E-17 | 29.10393745 |
| **TWISTNB** | 0.777769 | 1.919651 | 8.929227 | 1.36E-17 | 9.59E-17 | 29.10069289 |
| **RP11-390E23.6** | 0.777294 | 2.280719 | 8.929159 | 1.36E-17 | 9.59E-17 | 29.10018193 |
| **LRP12** | 0.883594 | 2.005062 | 8.92912 | 1.36E-17 | 9.59E-17 | 29.09988995 |
| **SCYL3** | 0.669796 | 1.617688 | 8.927754 | 1.38E-17 | 9.69E-17 | 29.08967532 |
| **DLST** | 1.024505 | 4.151475 | 8.92774 | 1.38E-17 | 9.69E-17 | 29.08957501 |
| **PUM1** | 1.174521 | 3.745337 | 8.926671 | 1.39E-17 | 9.77E-17 | 29.08157868 |
| **ZNF430** | 0.744218 | 1.567916 | 8.926412 | 1.39E-17 | 9.78E-17 | 29.07964201 |
| **ZNF329** | 0.764775 | 2.232502 | 8.926031 | 1.40E-17 | 9.81E-17 | 29.07679024 |
| **ARHGAP25** | 0.795276 | 2.113551 | 8.925665 | 1.40E-17 | 9.83E-17 | 29.07405826 |
| **SLC5A6** | 1.020915 | 3.613488 | 8.925643 | 1.40E-17 | 9.83E-17 | 29.07388898 |
| **RP11-33B1.1** | 0.923211 | 3.207098 | 8.924929 | 1.41E-17 | 9.88E-17 | 29.06855096 |
| **RANBP3** | 1.158834 | 5.295108 | 8.924357 | 1.41E-17 | 9.92E-17 | 29.06427589 |
| **RSBN1** | 0.75877 | 1.63061 | 8.923915 | 1.42E-17 | 9.95E-17 | 29.06096996 |
| **GADD45A** | 1.488435 | 4.5243 | 8.923847 | 1.42E-17 | 9.95E-17 | 29.0604643 |
| **PGM3** | 1.007484 | 3.090503 | 8.923556 | 1.42E-17 | 9.97E-17 | 29.05828392 |
| **ACVR1** | 0.997948 | 2.550856 | 8.923509 | 1.42E-17 | 9.97E-17 | 29.05793312 |
| **ABHD5** | 0.99988 | 2.48129 | 8.923465 | 1.42E-17 | 9.97E-17 | 29.0576086 |
| **NLE1** | 0.811136 | 3.036878 | 8.923158 | 1.43E-17 | 9.99E-17 | 29.05531132 |
| **ANKRD13C** | 0.841501 | 2.519049 | 8.922614 | 1.43E-17 | 1.00E-16 | 29.05124489 |
| **ZUFSP** | 0.72499 | 2.563378 | 8.922258 | 1.44E-17 | 1.01E-16 | 29.04858017 |
| **IRAK1BP1** | 0.506393 | 1.382087 | 8.921674 | 1.44E-17 | 1.01E-16 | 29.0442188 |
| **PDSS2** | 0.6495 | 2.101555 | 8.920468 | 1.46E-17 | 1.02E-16 | 29.03520193 |
| **SUDS3** | 0.934313 | 2.863415 | 8.919739 | 1.47E-17 | 1.02E-16 | 29.02975649 |
| **ATF6B** | 1.379483 | 5.149064 | 8.919528 | 1.47E-17 | 1.03E-16 | 29.02818012 |
| **FAM185A** | 0.621876 | 1.882575 | 8.919078 | 1.47E-17 | 1.03E-16 | 29.02481362 |
| **UBTF** | 1.146811 | 5.107908 | 8.918824 | 1.48E-17 | 1.03E-16 | 29.02291154 |
| **TMEM183A** | 1.117789 | 4.358781 | 8.918765 | 1.48E-17 | 1.03E-16 | 29.02247253 |
| **ZNF618** | 0.623245 | 1.039777 | 8.918634 | 1.48E-17 | 1.03E-16 | 29.02149633 |
| **LIMD1** | 0.855973 | 1.515426 | 8.917923 | 1.49E-17 | 1.04E-16 | 29.01617947 |
| **LRIF1** | 0.871909 | 2.386262 | 8.9175 | 1.49E-17 | 1.04E-16 | 29.01302012 |
| **RAB10** | 1.332492 | 4.835035 | 8.917283 | 1.49E-17 | 1.04E-16 | 29.01139595 |
| **CSRNP2** | 1.098054 | 3.259872 | 8.916807 | 1.50E-17 | 1.04E-16 | 29.00783939 |
| **ZSCAN30** | 0.987582 | 3.006039 | 8.916477 | 1.50E-17 | 1.05E-16 | 29.00537929 |
| **TP53BP1** | 0.955385 | 3.620059 | 8.916033 | 1.51E-17 | 1.05E-16 | 29.00205874 |
| **ZNF516** | 0.737905 | 2.611955 | 8.915605 | 1.51E-17 | 1.05E-16 | 28.99886114 |
| **PGS1** | 1.083212 | 3.991093 | 8.914939 | 1.52E-17 | 1.06E-16 | 28.99388488 |
| **HMGXB4** | 1.052034 | 2.333349 | 8.914789 | 1.52E-17 | 1.06E-16 | 28.99276638 |
| **HNRNPA2B1** | 1.574342 | 8.090575 | 8.914556 | 1.52E-17 | 1.06E-16 | 28.9910192 |
| **CDR2** | 0.798741 | 2.021848 | 8.913384 | 1.54E-17 | 1.07E-16 | 28.98226653 |
| **ZNF550** | 0.713605 | 1.402747 | 8.913329 | 1.54E-17 | 1.07E-16 | 28.98185869 |
| **DPH3** | 0.866984 | 2.676641 | 8.913281 | 1.54E-17 | 1.07E-16 | 28.98150119 |
| **DNAJC3** | 0.821787 | 2.501717 | 8.912362 | 1.55E-17 | 1.08E-16 | 28.9746317 |
| **RECK** | 0.625262 | 1.613645 | 8.911407 | 1.56E-17 | 1.08E-16 | 28.96749931 |
| **RP11-379H18.1** | 0.77008 | 1.656688 | 8.911086 | 1.56E-17 | 1.09E-16 | 28.96509894 |
| **ARHGAP27** | 0.911872 | 2.839825 | 8.909214 | 1.59E-17 | 1.10E-16 | 28.95111916 |
| **TRIM4** | 1.033928 | 2.903639 | 8.909102 | 1.59E-17 | 1.10E-16 | 28.95028642 |
| **BSDC1** | 1.124382 | 4.65658 | 8.908665 | 1.59E-17 | 1.11E-16 | 28.94702236 |
| **SCP2** | 1.528673 | 5.448456 | 8.908519 | 1.60E-17 | 1.11E-16 | 28.94593047 |
| **TRAF7** | 1.114077 | 4.701488 | 8.907237 | 1.61E-17 | 1.12E-16 | 28.93635988 |
| **EIF3A** | 1.342866 | 4.260576 | 8.906417 | 1.62E-17 | 1.12E-16 | 28.93023475 |
| **RBM12B** | 0.978926 | 1.78947 | 8.906194 | 1.62E-17 | 1.13E-16 | 28.92856877 |
| **TBC1D23** | 0.950037 | 3.091165 | 8.905839 | 1.63E-17 | 1.13E-16 | 28.92591586 |
| **STARD3NL** | 1.211364 | 4.671769 | 8.905447 | 1.63E-17 | 1.13E-16 | 28.92298927 |
| **BIN3** | 0.896582 | 3.832137 | 8.903942 | 1.65E-17 | 1.14E-16 | 28.91175515 |
| **SCARB2** | 1.420066 | 5.159944 | 8.903791 | 1.65E-17 | 1.15E-16 | 28.91062669 |
| **LACTB** | 0.761951 | 2.315448 | 8.903629 | 1.66E-17 | 1.15E-16 | 28.9094182 |
| **YTHDC1** | 1.192144 | 4.269932 | 8.90332 | 1.66E-17 | 1.15E-16 | 28.90710951 |
| **THUMPD1** | 1.041604 | 3.123354 | 8.903151 | 1.66E-17 | 1.15E-16 | 28.90584872 |
| **MGAT1** | 1.136032 | 5.437866 | 8.902883 | 1.66E-17 | 1.15E-16 | 28.90385428 |
| **NRBP1** | 1.146607 | 4.878796 | 8.901622 | 1.68E-17 | 1.16E-16 | 28.89444056 |
| **C17orf85** | 1.036612 | 3.399146 | 8.901512 | 1.68E-17 | 1.16E-16 | 28.89362106 |
| **C20orf112** | 0.878302 | 2.939703 | 8.90141 | 1.68E-17 | 1.16E-16 | 28.89285843 |
| **ARHGAP17** | 0.983051 | 3.852332 | 8.900837 | 1.69E-17 | 1.17E-16 | 28.88857636 |
| **UBE2G2** | 1.29705 | 5.059176 | 8.899277 | 1.71E-17 | 1.18E-16 | 28.87693435 |
| **GPBP1L1** | 1.147016 | 3.766979 | 8.899207 | 1.71E-17 | 1.18E-16 | 28.87641471 |
| **ZNF789** | 0.932569 | 3.170801 | 8.898404 | 1.72E-17 | 1.19E-16 | 28.87042582 |
| **PDE12** | 0.773464 | 2.112104 | 8.898382 | 1.72E-17 | 1.19E-16 | 28.87025759 |
| **ABCF1** | 1.137274 | 4.718552 | 8.897865 | 1.73E-17 | 1.19E-16 | 28.86639972 |
| **SLC44A5** | 0.918586 | 1.64464 | 8.897503 | 1.73E-17 | 1.20E-16 | 28.863699 |
| **BRCC3** | 0.850577 | 2.266349 | 8.897393 | 1.74E-17 | 1.20E-16 | 28.86287991 |
| **PKN2** | 0.947107 | 2.384545 | 8.896683 | 1.74E-17 | 1.20E-16 | 28.85758197 |
| **EIF2B2** | 1.087966 | 4.254999 | 8.896044 | 1.75E-17 | 1.21E-16 | 28.85281488 |
| **TRMT13** | 1.010106 | 2.418597 | 8.895606 | 1.76E-17 | 1.21E-16 | 28.84954999 |
| **ZNF433** | 0.651681 | 1.748789 | 8.895155 | 1.77E-17 | 1.22E-16 | 28.84618627 |
| **ZNF570** | 0.733531 | 1.139191 | 8.894488 | 1.77E-17 | 1.22E-16 | 28.84120795 |
| **CYTH3** | 1.075486 | 3.251022 | 8.894362 | 1.78E-17 | 1.22E-16 | 28.8402677 |
| **MSH5** | 1.089074 | 3.286964 | 8.893636 | 1.79E-17 | 1.23E-16 | 28.83484945 |
| **TMEM110** | 0.813586 | 2.675031 | 8.893471 | 1.79E-17 | 1.23E-16 | 28.83362064 |
| **SH3RF3-AS1** | 0.598469 | 1.194888 | 8.891431 | 1.82E-17 | 1.25E-16 | 28.81840752 |
| **NUP133** | 0.963703 | 3.635703 | 8.891114 | 1.82E-17 | 1.25E-16 | 28.81604241 |
| **RP11-347C12.2** | 0.770213 | 1.827902 | 8.890752 | 1.83E-17 | 1.25E-16 | 28.81334379 |
| **PMS2CL** | 0.834533 | 3.699985 | 8.890167 | 1.83E-17 | 1.26E-16 | 28.80897535 |
| **ZC3H7A** | 1.05054 | 3.830698 | 8.889943 | 1.84E-17 | 1.26E-16 | 28.80730487 |
| **CLDN12** | 1.041261 | 3.180979 | 8.889346 | 1.84E-17 | 1.27E-16 | 28.80285369 |
| **PSMB2** | 1.141012 | 4.503002 | 8.888752 | 1.85E-17 | 1.27E-16 | 28.7984295 |
| **IARS2** | 1.046691 | 3.577994 | 8.888493 | 1.86E-17 | 1.27E-16 | 28.79649933 |
| **STRADA** | 1.136387 | 4.711403 | 8.888225 | 1.86E-17 | 1.28E-16 | 28.79449749 |
| **NFIC** | 1.15543 | 3.922193 | 8.887861 | 1.87E-17 | 1.28E-16 | 28.79178136 |
| **HARBI1** | 0.591443 | 1.5317 | 8.886973 | 1.88E-17 | 1.29E-16 | 28.78516443 |
| **ARMC10** | 1.095714 | 3.507026 | 8.886429 | 1.89E-17 | 1.29E-16 | 28.78110804 |
| **SCAMP2** | 1.153666 | 4.731699 | 8.886292 | 1.89E-17 | 1.29E-16 | 28.78008969 |
| **SLC25A36** | 1.314012 | 4.027351 | 8.886009 | 1.89E-17 | 1.30E-16 | 28.77797636 |
| **ENTPD4** | 1.031414 | 2.894623 | 8.885267 | 1.90E-17 | 1.30E-16 | 28.77244527 |
| **CCAR2** | 1.263796 | 6.148129 | 8.883722 | 1.92E-17 | 1.32E-16 | 28.76092953 |
| **EMCN** | 0.656618 | 1.626178 | 8.883671 | 1.93E-17 | 1.32E-16 | 28.76055071 |
| **ZNF827** | 1.096367 | 2.740649 | 8.883276 | 1.93E-17 | 1.32E-16 | 28.75760438 |
| **STK3** | 0.797786 | 1.967329 | 8.882103 | 1.95E-17 | 1.33E-16 | 28.74886379 |
| **IL17RD** | 0.932315 | 2.541449 | 8.881523 | 1.96E-17 | 1.34E-16 | 28.74453639 |
| **IGSF6** | 1.029788 | 2.261111 | 8.881151 | 1.96E-17 | 1.34E-16 | 28.74176726 |
| **ZNF132** | 0.577553 | 1.314507 | 8.880944 | 1.97E-17 | 1.34E-16 | 28.74022203 |
| **DCP1A** | 0.787574 | 2.013099 | 8.880912 | 1.97E-17 | 1.34E-16 | 28.73998537 |
| **TVP23B** | 1.033579 | 3.216947 | 8.880904 | 1.97E-17 | 1.34E-16 | 28.73992813 |
| **MGAT4A** | 0.862228 | 2.699828 | 8.88079 | 1.97E-17 | 1.34E-16 | 28.73907558 |
| **WDR33** | 0.873666 | 3.440191 | 8.880069 | 1.98E-17 | 1.35E-16 | 28.73370398 |
| **TMEM251** | 1.020551 | 3.824599 | 8.879884 | 1.98E-17 | 1.35E-16 | 28.73232284 |
| **ZMIZ1** | 1.379244 | 4.283194 | 8.879728 | 1.98E-17 | 1.35E-16 | 28.73116176 |
| **PIK3CA** | 0.943176 | 2.099718 | 8.879602 | 1.99E-17 | 1.35E-16 | 28.7302263 |
| **SOX6** | 1.156661 | 3.002585 | 8.87939 | 1.99E-17 | 1.36E-16 | 28.72864363 |
| **RNF24** | 0.792376 | 2.882643 | 8.879017 | 1.99E-17 | 1.36E-16 | 28.72586491 |
| **AASDH** | 0.698202 | 2.020768 | 8.878953 | 2.00E-17 | 1.36E-16 | 28.72539059 |
| **WDR81** | 0.881104 | 3.664669 | 8.878779 | 2.00E-17 | 1.36E-16 | 28.72409411 |
| **DPH6** | 0.779253 | 1.917853 | 8.878675 | 2.00E-17 | 1.36E-16 | 28.72331876 |
| **VIPAS39** | 0.826527 | 2.928031 | 8.878547 | 2.00E-17 | 1.36E-16 | 28.72236292 |
| **ZSCAN12** | 0.648535 | 1.167177 | 8.877796 | 2.01E-17 | 1.37E-16 | 28.71677269 |
| **MAGI1** | 1.081437 | 3.1364 | 8.877275 | 2.02E-17 | 1.38E-16 | 28.71288487 |
| **PSMD3** | 1.252159 | 5.762905 | 8.877041 | 2.02E-17 | 1.38E-16 | 28.71114703 |
| **HOMEZ** | 0.635545 | 1.981566 | 8.876831 | 2.03E-17 | 1.38E-16 | 28.70958191 |
| **DNM2** | 1.278077 | 5.531355 | 8.876544 | 2.03E-17 | 1.38E-16 | 28.70744193 |
| **THBD** | 0.686205 | 0.871978 | 8.87631 | 2.04E-17 | 1.38E-16 | 28.7056992 |
| **ARPC2** | 1.449033 | 6.079262 | 8.875885 | 2.04E-17 | 1.39E-16 | 28.70253353 |
| **FOXK1** | 0.835228 | 2.906934 | 8.875275 | 2.05E-17 | 1.39E-16 | 28.69798997 |
| **HIRA** | 1.009857 | 3.493515 | 8.874685 | 2.06E-17 | 1.40E-16 | 28.69359717 |
| **GAB1** | 1.17336 | 3.177362 | 8.874047 | 2.07E-17 | 1.41E-16 | 28.68884218 |
| **PSPC1** | 0.962342 | 4.167675 | 8.873608 | 2.08E-17 | 1.41E-16 | 28.6855728 |
| **CXCR4** | 1.094013 | 2.97566 | 8.872661 | 2.09E-17 | 1.42E-16 | 28.6785234 |
| **ATF7** | 0.97585 | 3.336067 | 8.872316 | 2.10E-17 | 1.42E-16 | 28.67595245 |
| **ZNF675** | 0.823788 | 2.011862 | 8.872219 | 2.10E-17 | 1.42E-16 | 28.67522977 |
| **ASTE1** | 0.601205 | 1.613769 | 8.871942 | 2.10E-17 | 1.43E-16 | 28.67316613 |
| **CCDC43** | 0.964421 | 3.093423 | 8.871621 | 2.11E-17 | 1.43E-16 | 28.67077231 |
| **HYOU1** | 1.251504 | 4.105746 | 8.870673 | 2.12E-17 | 1.44E-16 | 28.66371632 |
| **ACOT9** | 0.98632 | 3.357879 | 8.870105 | 2.13E-17 | 1.45E-16 | 28.65949002 |
| **RNF219** | 0.768684 | 1.972745 | 8.869685 | 2.14E-17 | 1.45E-16 | 28.65635796 |
| **NBPF15** | 1.058512 | 2.92471 | 8.86948 | 2.14E-17 | 1.45E-16 | 28.65483481 |
| **CARM1** | 1.080679 | 4.211603 | 8.86911 | 2.15E-17 | 1.45E-16 | 28.65207894 |
| **ZNF343** | 0.650958 | 2.10194 | 8.8689 | 2.15E-17 | 1.46E-16 | 28.6505153 |
| **TPGS2** | 1.329488 | 4.990686 | 8.868655 | 2.16E-17 | 1.46E-16 | 28.64868779 |
| **MAP3K4** | 0.838268 | 3.219692 | 8.868513 | 2.16E-17 | 1.46E-16 | 28.64763591 |
| **PIGS** | 1.170763 | 4.271199 | 8.868481 | 2.16E-17 | 1.46E-16 | 28.64739689 |
| **EGFLAM** | 0.666113 | 1.137065 | 8.867853 | 2.17E-17 | 1.47E-16 | 28.642717 |
| **DND1P1** | 0.643938 | 1.220289 | 8.866912 | 2.19E-17 | 1.48E-16 | 28.63571345 |
| **TGDS** | 0.793102 | 1.894343 | 8.866734 | 2.19E-17 | 1.48E-16 | 28.6343907 |
| **EXOC3L2** | 0.677396 | 0.572625 | 8.866649 | 2.19E-17 | 1.48E-16 | 28.63375499 |
| **AC006011.4** | 1.042903 | 3.713625 | 8.86611 | 2.20E-17 | 1.48E-16 | 28.62974408 |
| **PLEKHO2** | 0.939239 | 3.515261 | 8.866104 | 2.20E-17 | 1.48E-16 | 28.62970207 |
| **EDNRA** | 0.93991 | 1.864996 | 8.865788 | 2.20E-17 | 1.49E-16 | 28.62734935 |
| **VKORC1L1** | 0.870034 | 2.811771 | 8.865565 | 2.21E-17 | 1.49E-16 | 28.62568715 |
| **FNBP4** | 1.24595 | 4.091289 | 8.865284 | 2.21E-17 | 1.49E-16 | 28.62360102 |
| **RELL1** | 1.03977 | 1.539646 | 8.864973 | 2.22E-17 | 1.50E-16 | 28.62128609 |
| **KLF11** | 0.563828 | 0.951617 | 8.8642 | 2.23E-17 | 1.50E-16 | 28.61552597 |
| **RIF1** | 0.74514 | 1.907083 | 8.864065 | 2.23E-17 | 1.50E-16 | 28.6145285 |
| **BMPR1A** | 0.802178 | 2.085089 | 8.864064 | 2.23E-17 | 1.50E-16 | 28.61451908 |
| **GTF2H2B** | 0.677409 | 1.743936 | 8.863862 | 2.24E-17 | 1.51E-16 | 28.61301652 |
| **PRPF38B** | 1.226523 | 3.63904 | 8.863642 | 2.24E-17 | 1.51E-16 | 28.61137699 |
| **NFYA** | 1.024076 | 2.265864 | 8.863636 | 2.24E-17 | 1.51E-16 | 28.61133005 |
| **SRSF6** | 1.335961 | 5.263632 | 8.863608 | 2.24E-17 | 1.51E-16 | 28.61112145 |
| **ZNF711** | 1.232467 | 3.371892 | 8.86339 | 2.24E-17 | 1.51E-16 | 28.60950044 |
| **ACVR2B** | 0.616301 | 1.350927 | 8.863191 | 2.25E-17 | 1.51E-16 | 28.60802107 |
| **CTR9** | 0.963411 | 2.773716 | 8.861719 | 2.27E-17 | 1.53E-16 | 28.59706413 |
| **CD58** | 0.897267 | 2.057837 | 8.861695 | 2.27E-17 | 1.53E-16 | 28.59688805 |
| **KCTD3** | 1.249762 | 3.964054 | 8.860845 | 2.29E-17 | 1.54E-16 | 28.59056227 |
| **SDAD1** | 1.045923 | 2.636959 | 8.860803 | 2.29E-17 | 1.54E-16 | 28.59025447 |
| **TRIM32** | 0.768987 | 1.943339 | 8.859961 | 2.30E-17 | 1.55E-16 | 28.58398644 |
| **ZNF175** | 0.649205 | 1.492531 | 8.85917 | 2.32E-17 | 1.56E-16 | 28.57810044 |
| **CUL1** | 1.083233 | 3.976456 | 8.858602 | 2.33E-17 | 1.56E-16 | 28.57387398 |
| **LINC00938** | 1.009902 | 3.347039 | 8.858557 | 2.33E-17 | 1.56E-16 | 28.57354217 |
| **TUBA1A** | 1.807813 | 9.219393 | 8.85835 | 2.33E-17 | 1.56E-16 | 28.57200207 |
| **KPNA4** | 0.85854 | 2.83914 | 8.857652 | 2.34E-17 | 1.57E-16 | 28.56680758 |
| **CNEP1R1** | 0.906809 | 2.803601 | 8.857611 | 2.34E-17 | 1.57E-16 | 28.5665009 |
| **PITPNC1** | 1.048924 | 3.958392 | 8.857569 | 2.35E-17 | 1.57E-16 | 28.56619168 |
| **PFN1** | 1.671288 | 7.866944 | 8.857016 | 2.36E-17 | 1.58E-16 | 28.56207996 |
| **MTRF1L** | 0.848287 | 3.065372 | 8.856542 | 2.36E-17 | 1.58E-16 | 28.5585494 |
| **LRRC58** | 1.01647 | 2.737548 | 8.856004 | 2.37E-17 | 1.59E-16 | 28.55455413 |
| **SENP8** | 0.561845 | 1.478399 | 8.855456 | 2.38E-17 | 1.59E-16 | 28.55047394 |
| **GNG4** | 1.206337 | 3.970531 | 8.855216 | 2.39E-17 | 1.60E-16 | 28.5486876 |
| **CHML** | 0.665898 | 1.33173 | 8.854025 | 2.41E-17 | 1.61E-16 | 28.53983045 |
| **LIN54** | 0.71164 | 1.693367 | 8.8537 | 2.42E-17 | 1.61E-16 | 28.53741503 |
| **PPP1CB** | 1.4572 | 5.137551 | 8.853626 | 2.42E-17 | 1.61E-16 | 28.53686665 |
| **UBXN7** | 0.850218 | 2.238253 | 8.852979 | 2.43E-17 | 1.62E-16 | 28.53205818 |
| **TBXA2R** | 0.574285 | 1.299472 | 8.852286 | 2.44E-17 | 1.63E-16 | 28.52690232 |
| **WWP2** | 1.010516 | 3.920432 | 8.8519 | 2.45E-17 | 1.63E-16 | 28.52403514 |
| **GSC** | 0.735125 | 1.150928 | 8.851614 | 2.45E-17 | 1.64E-16 | 28.52190292 |
| **C18orf25** | 0.791095 | 2.025109 | 8.850126 | 2.48E-17 | 1.66E-16 | 28.5108389 |
| **EPB41L5** | 0.881912 | 2.322019 | 8.850071 | 2.48E-17 | 1.66E-16 | 28.51043444 |
| **OXA1L** | 1.192464 | 4.966243 | 8.849641 | 2.49E-17 | 1.66E-16 | 28.50723947 |
| **ZNF583** | 0.675097 | 1.456188 | 8.848865 | 2.50E-17 | 1.67E-16 | 28.50146998 |
| **FOPNL** | 0.914162 | 3.332525 | 8.84879 | 2.51E-17 | 1.67E-16 | 28.50090846 |
| **RALGAPA2** | 0.700398 | 1.697922 | 8.848762 | 2.51E-17 | 1.67E-16 | 28.50070035 |
| **KDELC2** | 0.877562 | 1.669738 | 8.848432 | 2.51E-17 | 1.67E-16 | 28.49825088 |
| **CCDC127** | 0.644906 | 2.052878 | 8.847995 | 2.52E-17 | 1.68E-16 | 28.4949979 |
| **KRT8P12** | 0.694133 | 2.293215 | 8.847368 | 2.53E-17 | 1.69E-16 | 28.4903399 |
| **HOXD4** | 0.905081 | 0.963196 | 8.846413 | 2.55E-17 | 1.70E-16 | 28.48324573 |
| **NPM1** | 1.739535 | 7.024832 | 8.846078 | 2.56E-17 | 1.70E-16 | 28.48074976 |
| **LAMTOR3** | 1.217394 | 4.148425 | 8.845527 | 2.57E-17 | 1.71E-16 | 28.47666011 |
| **ZNF432** | 0.879175 | 2.337145 | 8.844992 | 2.58E-17 | 1.72E-16 | 28.4726824 |
| **TAF7** | 1.380076 | 4.978765 | 8.844664 | 2.59E-17 | 1.72E-16 | 28.47024212 |
| **FUT11** | 0.88431 | 3.251149 | 8.844566 | 2.59E-17 | 1.72E-16 | 28.4695177 |
| **RTCA** | 0.940877 | 3.118293 | 8.844542 | 2.59E-17 | 1.72E-16 | 28.46933781 |
| **ZNF160** | 0.808385 | 2.603311 | 8.843586 | 2.61E-17 | 1.73E-16 | 28.46223422 |
| **TANC2** | 1.052183 | 2.99915 | 8.84349 | 2.61E-17 | 1.73E-16 | 28.46151849 |
| **LSM6** | 1.265972 | 4.545514 | 8.842427 | 2.63E-17 | 1.75E-16 | 28.45362057 |
| **SMAD2** | 1.197723 | 3.787893 | 8.842232 | 2.63E-17 | 1.75E-16 | 28.4521706 |
| **PMS2** | 0.856956 | 2.321541 | 8.841205 | 2.65E-17 | 1.76E-16 | 28.44454063 |
| **GOSR2** | 1.077996 | 4.57547 | 8.840788 | 2.66E-17 | 1.77E-16 | 28.44144692 |
| **ICK** | 0.900756 | 2.232023 | 8.839138 | 2.70E-17 | 1.79E-16 | 28.42918973 |
| **GLG1** | 1.147972 | 4.478606 | 8.839032 | 2.70E-17 | 1.79E-16 | 28.42839895 |
| **SLMAP** | 1.04732 | 3.170553 | 8.838824 | 2.70E-17 | 1.79E-16 | 28.42685536 |
| **POLR2J3** | 1.215338 | 5.207283 | 8.8373 | 2.73E-17 | 1.81E-16 | 28.41554056 |
| **SPRED3** | 0.959274 | 1.638599 | 8.837177 | 2.74E-17 | 1.81E-16 | 28.41462622 |
| **NIT2** | 1.0129 | 3.935134 | 8.8365 | 2.75E-17 | 1.82E-16 | 28.40959759 |
| **CDKN2AIP** | 0.917002 | 2.765723 | 8.835876 | 2.76E-17 | 1.83E-16 | 28.40496016 |
| **C6orf62** | 1.336616 | 4.863931 | 8.83548 | 2.77E-17 | 1.83E-16 | 28.40201977 |
| **PTPRS** | 1.14408 | 5.654582 | 8.834914 | 2.78E-17 | 1.84E-16 | 28.39781574 |
| **METTL13** | 0.964113 | 3.863315 | 8.834622 | 2.79E-17 | 1.85E-16 | 28.3956534 |
| **EIF4B** | 1.354747 | 5.651419 | 8.833646 | 2.81E-17 | 1.86E-16 | 28.38839951 |
| **UBE2A** | 1.294025 | 4.470974 | 8.833225 | 2.82E-17 | 1.86E-16 | 28.38527639 |
| **FBXL5** | 1.239108 | 4.523008 | 8.833171 | 2.82E-17 | 1.86E-16 | 28.38487873 |
| **HSPD1** | 1.508147 | 6.057236 | 8.832196 | 2.84E-17 | 1.88E-16 | 28.37764101 |
| **PWP2** | 0.902556 | 3.751976 | 8.832021 | 2.84E-17 | 1.88E-16 | 28.37633744 |
| **ASH2L** | 1.018259 | 3.982261 | 8.831617 | 2.85E-17 | 1.88E-16 | 28.37333923 |
| **CLPX** | 1.060817 | 3.117298 | 8.831222 | 2.86E-17 | 1.89E-16 | 28.37040817 |
| **ZDHHC9** | 1.175926 | 3.817743 | 8.830467 | 2.88E-17 | 1.90E-16 | 28.36480167 |
| **MTMR11** | 0.969971 | 2.341923 | 8.829488 | 2.90E-17 | 1.91E-16 | 28.35753569 |
| **RNPS1** | 1.372066 | 6.193996 | 8.829104 | 2.91E-17 | 1.92E-16 | 28.35468903 |
| **ASAH2B** | 0.663436 | 1.634601 | 8.828882 | 2.91E-17 | 1.92E-16 | 28.35303604 |
| **PTTG1IP** | 1.407512 | 6.451749 | 8.828565 | 2.92E-17 | 1.93E-16 | 28.35068485 |
| **AGO3** | 0.701466 | 1.632775 | 8.828535 | 2.92E-17 | 1.93E-16 | 28.35046083 |
| **ETV1** | 1.481898 | 4.584011 | 8.828444 | 2.92E-17 | 1.93E-16 | 28.34978363 |
| **HHEX** | 0.729194 | 1.510471 | 8.82835 | 2.92E-17 | 1.93E-16 | 28.34909107 |
| **GJA4** | 0.849827 | 1.960209 | 8.828231 | 2.93E-17 | 1.93E-16 | 28.34820497 |
| **RIC8B** | 0.878576 | 2.581102 | 8.827275 | 2.95E-17 | 1.94E-16 | 28.34111266 |
| **DOK2** | 0.752122 | 1.151184 | 8.827067 | 2.95E-17 | 1.94E-16 | 28.33957115 |
| **VPS41** | 1.215939 | 4.013632 | 8.826436 | 2.97E-17 | 1.95E-16 | 28.33488312 |
| **SLC35E1** | 0.876075 | 3.466587 | 8.826131 | 2.97E-17 | 1.96E-16 | 28.33262143 |
| **TFG** | 1.248443 | 4.702413 | 8.825942 | 2.98E-17 | 1.96E-16 | 28.33121949 |
| **PROCR** | 0.756682 | 2.114364 | 8.825158 | 3.00E-17 | 1.97E-16 | 28.32540314 |
| **DDX58** | 0.662482 | 1.640713 | 8.825114 | 3.00E-17 | 1.97E-16 | 28.3250788 |
| **ZNF219** | 1.170205 | 4.717532 | 8.82475 | 3.00E-17 | 1.97E-16 | 28.32237306 |
| **HOXD8** | 1.121026 | 1.312505 | 8.824069 | 3.02E-17 | 1.98E-16 | 28.31731955 |
| **DHX36** | 1.224102 | 3.503667 | 8.823549 | 3.03E-17 | 1.99E-16 | 28.31346229 |
| **BTBD7** | 0.712584 | 1.990458 | 8.8223 | 3.06E-17 | 2.01E-16 | 28.30420056 |
| **ACTN1** | 1.467195 | 3.833884 | 8.822217 | 3.06E-17 | 2.01E-16 | 28.30358112 |
| **SPOP** | 1.246325 | 4.327292 | 8.820928 | 3.09E-17 | 2.03E-16 | 28.29402131 |
| **FAM53C** | 0.93142 | 3.458915 | 8.819944 | 3.12E-17 | 2.04E-16 | 28.28671942 |
| **CACNG4** | 1.497159 | 4.863594 | 8.819415 | 3.13E-17 | 2.05E-16 | 28.28280109 |
| **LARP1B** | 0.754487 | 2.171029 | 8.819173 | 3.13E-17 | 2.05E-16 | 28.28100598 |
| **ITGAV** | 1.250012 | 4.004423 | 8.818989 | 3.14E-17 | 2.06E-16 | 28.27964257 |
| **CCT6P1** | 0.81255 | 1.540491 | 8.817902 | 3.16E-17 | 2.07E-16 | 28.27157696 |
| **RNF135** | 0.862215 | 2.297603 | 8.817561 | 3.17E-17 | 2.08E-16 | 28.26904695 |
| **ZDHHC3** | 1.006456 | 3.865877 | 8.817053 | 3.18E-17 | 2.09E-16 | 28.26528394 |
| **DLD** | 1.405412 | 4.311525 | 8.816949 | 3.19E-17 | 2.09E-16 | 28.26451056 |
| **MSANTD4** | 1.071134 | 2.974584 | 8.816798 | 3.19E-17 | 2.09E-16 | 28.26339198 |
| **GSE1** | 1.106018 | 3.017875 | 8.816633 | 3.19E-17 | 2.09E-16 | 28.26216936 |
| **MBD1** | 1.147888 | 4.568767 | 8.81611 | 3.21E-17 | 2.10E-16 | 28.25828726 |
| **GTF2H2** | 1.008309 | 2.558051 | 8.814999 | 3.23E-17 | 2.11E-16 | 28.25005354 |
| **USP28** | 0.906782 | 2.12215 | 8.814191 | 3.25E-17 | 2.13E-16 | 28.24405925 |
| **ZNF658** | 0.548926 | 1.060419 | 8.813677 | 3.27E-17 | 2.13E-16 | 28.24025211 |
| **CCNE1** | 0.633847 | 1.822169 | 8.812837 | 3.29E-17 | 2.15E-16 | 28.23402565 |
| **GTF2E2** | 0.804582 | 2.926278 | 8.811914 | 3.31E-17 | 2.16E-16 | 28.22718119 |
| **GLDC** | 1.059406 | 3.396318 | 8.811851 | 3.31E-17 | 2.16E-16 | 28.22671238 |
| **SLC35A3** | 0.854877 | 1.819043 | 8.811651 | 3.32E-17 | 2.16E-16 | 28.22523485 |
| **RBM43** | 0.632525 | 1.327221 | 8.810857 | 3.34E-17 | 2.18E-16 | 28.21934849 |
| **SEC13** | 1.256451 | 5.434972 | 8.810847 | 3.34E-17 | 2.18E-16 | 28.21927341 |
| **RPP38** | 0.875175 | 3.175572 | 8.809306 | 3.37E-17 | 2.20E-16 | 28.20785226 |
| **METAP2** | 1.301516 | 4.83988 | 8.809161 | 3.38E-17 | 2.20E-16 | 28.20677486 |
| **IMPAD1** | 1.06513 | 3.283019 | 8.808788 | 3.39E-17 | 2.21E-16 | 28.20401563 |
| **BRWD3** | 0.649307 | 1.280311 | 8.808473 | 3.40E-17 | 2.21E-16 | 28.20168025 |
| **TTC28** | 0.758668 | 2.001519 | 8.808417 | 3.40E-17 | 2.21E-16 | 28.20126421 |
| **FAM43A** | 0.797922 | 1.895914 | 8.807753 | 3.41E-17 | 2.22E-16 | 28.1963421 |
| **NSA2** | 1.345029 | 5.024548 | 8.807263 | 3.43E-17 | 2.23E-16 | 28.19271345 |
| **EIF4A3** | 1.319877 | 5.401956 | 8.806359 | 3.45E-17 | 2.25E-16 | 28.1860181 |
| **ACTA2** | 1.384746 | 4.411123 | 8.806192 | 3.45E-17 | 2.25E-16 | 28.18477777 |
| **GNA11** | 1.101875 | 4.600018 | 8.80575 | 3.47E-17 | 2.26E-16 | 28.1815043 |
| **RP11-673C5.1** | 1.490184 | 5.684772 | 8.805706 | 3.47E-17 | 2.26E-16 | 28.18117992 |
| **CTSS** | 1.254447 | 2.927728 | 8.80559 | 3.47E-17 | 2.26E-16 | 28.18031796 |
| **ATP6V1G1** | 1.457641 | 5.692193 | 8.804693 | 3.49E-17 | 2.27E-16 | 28.17367056 |
| **CEPT1** | 1.112791 | 2.768733 | 8.804673 | 3.49E-17 | 2.27E-16 | 28.17352147 |
| **TMEM185A** | 0.81453 | 3.138971 | 8.803148 | 3.54E-17 | 2.30E-16 | 28.16222781 |
| **GRPEL2** | 0.718299 | 2.030112 | 8.802989 | 3.54E-17 | 2.30E-16 | 28.16104749 |
| **PCF11** | 1.129646 | 3.086832 | 8.802887 | 3.54E-17 | 2.30E-16 | 28.16029722 |
| **EOGT** | 0.636315 | 1.414921 | 8.802574 | 3.55E-17 | 2.31E-16 | 28.15797934 |
| **HAUS4** | 1.02321 | 3.742497 | 8.802203 | 3.56E-17 | 2.31E-16 | 28.15522779 |
| **HMG20A** | 0.966749 | 3.506388 | 8.801731 | 3.57E-17 | 2.32E-16 | 28.15173595 |
| **L3HYPDH** | 1.15707 | 3.396795 | 8.801249 | 3.59E-17 | 2.33E-16 | 28.1481646 |
| **RAD21** | 1.407358 | 4.790588 | 8.800855 | 3.60E-17 | 2.33E-16 | 28.14524209 |
| **PIAS3** | 1.176062 | 3.006479 | 8.800729 | 3.60E-17 | 2.33E-16 | 28.14430853 |
| **ZNRF2** | 0.766234 | 1.933032 | 8.800619 | 3.60E-17 | 2.33E-16 | 28.14349367 |
| **KIAA0947** | 0.906191 | 2.355922 | 8.800617 | 3.60E-17 | 2.33E-16 | 28.14348491 |
| **YAE1D1** | 1.156982 | 4.002352 | 8.800059 | 3.62E-17 | 2.34E-16 | 28.13934814 |
| **NECAP2** | 1.126906 | 4.830097 | 8.79956 | 3.63E-17 | 2.35E-16 | 28.13565709 |
| **SIRT7** | 1.016187 | 4.316827 | 8.799356 | 3.64E-17 | 2.35E-16 | 28.13414324 |
| **TTC4** | 0.884984 | 3.38783 | 8.799321 | 3.64E-17 | 2.35E-16 | 28.13388519 |
| **DTD2** | 1.079172 | 3.052865 | 8.799285 | 3.64E-17 | 2.35E-16 | 28.13361757 |
| **SP100** | 0.97736 | 2.766415 | 8.799179 | 3.64E-17 | 2.36E-16 | 28.13282968 |
| **NFX1** | 0.869489 | 3.122419 | 8.798948 | 3.65E-17 | 2.36E-16 | 28.13112484 |
| **ZNF587B** | 0.547986 | 1.363318 | 8.798483 | 3.66E-17 | 2.37E-16 | 28.12767685 |
| **ZNF484** | 0.621536 | 1.192975 | 8.798457 | 3.66E-17 | 2.37E-16 | 28.1274904 |
| **NODAL** | 0.550609 | 0.655716 | 8.798183 | 3.67E-17 | 2.37E-16 | 28.12545529 |
| **AC004980.7** | 0.676879 | 1.889827 | 8.798012 | 3.67E-17 | 2.37E-16 | 28.12419016 |
| **UBE2Q2** | 0.951034 | 3.57707 | 8.797965 | 3.68E-17 | 2.37E-16 | 28.1238446 |
| **RC3H1** | 0.588689 | 1.528009 | 8.796752 | 3.71E-17 | 2.39E-16 | 28.1148618 |
| **TADA1** | 0.990945 | 2.753054 | 8.796169 | 3.73E-17 | 2.40E-16 | 28.11054563 |
| **YIPF6** | 0.950285 | 3.128648 | 8.796123 | 3.73E-17 | 2.40E-16 | 28.11020318 |
| **UBE2G1** | 1.219452 | 3.979023 | 8.7957 | 3.74E-17 | 2.41E-16 | 28.10707299 |
| **SNRPG** | 1.560575 | 6.247722 | 8.794865 | 3.76E-17 | 2.43E-16 | 28.10089071 |
| **NCBP2** | 1.346147 | 5.214146 | 8.794657 | 3.77E-17 | 2.43E-16 | 28.09935122 |
| **ZNHIT6** | 0.889675 | 2.613467 | 8.79456 | 3.77E-17 | 2.43E-16 | 28.09863439 |
| **IKZF4** | 0.780923 | 1.759747 | 8.793854 | 3.79E-17 | 2.44E-16 | 28.09340773 |
| **TMEM179B** | 1.114673 | 4.391895 | 8.793477 | 3.80E-17 | 2.45E-16 | 28.09061709 |
| **KLHL12** | 0.913629 | 3.391483 | 8.79337 | 3.80E-17 | 2.45E-16 | 28.08982519 |
| **CAPN7** | 0.957765 | 3.080035 | 8.792893 | 3.82E-17 | 2.46E-16 | 28.08629371 |
| **SOS2** | 0.974918 | 2.758628 | 8.792186 | 3.84E-17 | 2.47E-16 | 28.08106435 |
| **PPP4C** | 1.326695 | 5.867318 | 8.791939 | 3.85E-17 | 2.47E-16 | 28.07923376 |
| **MLYCD** | 0.716095 | 2.655521 | 8.79161 | 3.86E-17 | 2.48E-16 | 28.07680451 |
| **PLEK** | 0.942886 | 1.934991 | 8.79133 | 3.86E-17 | 2.48E-16 | 28.07473273 |
| **TCP11L1** | 0.821841 | 2.172467 | 8.791246 | 3.87E-17 | 2.49E-16 | 28.07411124 |
| **ISL2** | 0.522615 | 0.411457 | 8.791219 | 3.87E-17 | 2.49E-16 | 28.07390758 |
| **RP11-61L23.2** | 0.732737 | 1.024622 | 8.790973 | 3.87E-17 | 2.49E-16 | 28.07208712 |
| **UIMC1** | 0.806167 | 3.219315 | 8.790082 | 3.90E-17 | 2.50E-16 | 28.06549562 |
| **MKL1** | 0.929166 | 3.960785 | 8.79003 | 3.90E-17 | 2.51E-16 | 28.0651095 |
| **ZNF589** | 0.772471 | 2.731424 | 8.789644 | 3.91E-17 | 2.51E-16 | 28.06225338 |
| **ZNF407** | 0.516915 | 1.580715 | 8.789287 | 3.92E-17 | 2.52E-16 | 28.05961082 |
| **ERLIN2** | 1.075106 | 3.290058 | 8.788859 | 3.94E-17 | 2.53E-16 | 28.0564414 |
| **MRPL13** | 1.090731 | 4.197765 | 8.787404 | 3.98E-17 | 2.55E-16 | 28.045681 |
| **BCL2L13** | 1.094937 | 3.803376 | 8.787083 | 3.99E-17 | 2.56E-16 | 28.04330676 |
| **ZBTB14** | 0.838712 | 2.390219 | 8.786815 | 4.00E-17 | 2.56E-16 | 28.04132487 |
| **RHOC** | 1.537203 | 7.221214 | 8.785891 | 4.02E-17 | 2.58E-16 | 28.03448929 |
| **RASSF1** | 0.958319 | 3.842194 | 8.78449 | 4.07E-17 | 2.61E-16 | 28.02412066 |
| **CNOT4** | 0.889821 | 2.86269 | 8.784353 | 4.07E-17 | 2.61E-16 | 28.02310991 |
| **PSMC2** | 1.345356 | 5.111155 | 8.784081 | 4.08E-17 | 2.61E-16 | 28.02110039 |
| **ZDHHC2** | 0.879403 | 2.347763 | 8.783516 | 4.10E-17 | 2.62E-16 | 28.01691792 |
| **SETD4** | 0.930513 | 3.685469 | 8.782265 | 4.14E-17 | 2.65E-16 | 28.00767192 |
| **MAN1A2** | 0.839793 | 2.572694 | 8.782001 | 4.14E-17 | 2.65E-16 | 28.00571403 |
| **FANCM** | 0.51937 | 1.10897 | 8.781487 | 4.16E-17 | 2.66E-16 | 28.00191635 |
| **PSMD14** | 1.216379 | 4.41868 | 8.780945 | 4.18E-17 | 2.67E-16 | 27.99790444 |
| **MRPL50** | 0.907197 | 2.786441 | 8.780368 | 4.20E-17 | 2.68E-16 | 27.99364197 |
| **SRGN** | 1.383538 | 4.375863 | 8.779867 | 4.21E-17 | 2.69E-16 | 27.98993507 |
| **ZNF18** | 0.670255 | 2.459718 | 8.779398 | 4.23E-17 | 2.70E-16 | 27.98646891 |
| **SSB** | 1.430152 | 5.459065 | 8.779068 | 4.24E-17 | 2.71E-16 | 27.9840319 |
| **C8orf33** | 1.184103 | 4.478616 | 8.778316 | 4.26E-17 | 2.72E-16 | 27.97847285 |
| **MYOF** | 0.8884 | 1.59923 | 8.777881 | 4.27E-17 | 2.73E-16 | 27.97525253 |
| **THAP6** | 0.847676 | 2.093998 | 8.777607 | 4.28E-17 | 2.73E-16 | 27.97322434 |
| **KIAA2013** | 1.036767 | 4.293851 | 8.777524 | 4.29E-17 | 2.73E-16 | 27.97261376 |
| **WDPCP** | 0.600519 | 1.597021 | 8.777457 | 4.29E-17 | 2.73E-16 | 27.97211557 |
| **SGSH** | 0.899051 | 3.588413 | 8.777049 | 4.30E-17 | 2.74E-16 | 27.9691004 |
| **APLN** | 1.467282 | 3.935597 | 8.776804 | 4.31E-17 | 2.75E-16 | 27.96729137 |
| **C9orf40** | 0.663928 | 2.036781 | 8.776769 | 4.31E-17 | 2.75E-16 | 27.96703297 |
| **PHF20L1** | 1.147267 | 3.354818 | 8.776657 | 4.31E-17 | 2.75E-16 | 27.96620473 |
| **CCDC142** | 0.892867 | 2.19972 | 8.775004 | 4.37E-17 | 2.78E-16 | 27.95398729 |
| **CTDP1** | 0.818576 | 3.360216 | 8.77471 | 4.38E-17 | 2.79E-16 | 27.95181595 |
| **PBDC1** | 1.003533 | 3.961376 | 8.774282 | 4.39E-17 | 2.80E-16 | 27.94864847 |
| **ZNF281** | 0.890796 | 2.16684 | 8.773596 | 4.41E-17 | 2.81E-16 | 27.94357995 |
| **MCCC2** | 1.063378 | 3.36882 | 8.773548 | 4.42E-17 | 2.81E-16 | 27.94322605 |
| **PCGF2** | 1.274965 | 4.693555 | 8.772898 | 4.44E-17 | 2.82E-16 | 27.93842588 |
| **PNP** | 1.094814 | 3.813759 | 8.772788 | 4.44E-17 | 2.82E-16 | 27.93760839 |
| **MTHFD1** | 1.097657 | 4.618223 | 8.772463 | 4.45E-17 | 2.83E-16 | 27.93520686 |
| **REC8** | 1.298444 | 4.860897 | 8.772048 | 4.47E-17 | 2.84E-16 | 27.93213892 |
| **SMIM15** | 1.138313 | 3.293291 | 8.771529 | 4.48E-17 | 2.85E-16 | 27.92830509 |
| **SMCHD1** | 0.985225 | 2.375968 | 8.771513 | 4.48E-17 | 2.85E-16 | 27.92819016 |
| **SH3GL1** | 1.215639 | 5.597757 | 8.771377 | 4.49E-17 | 2.85E-16 | 27.9271803 |
| **BAZ2A** | 1.102543 | 3.342091 | 8.771355 | 4.49E-17 | 2.85E-16 | 27.92702039 |
| **PTOV1-AS1** | 0.700542 | 1.971533 | 8.771307 | 4.49E-17 | 2.85E-16 | 27.92666325 |
| **GAB2** | 1.117115 | 3.504493 | 8.770682 | 4.51E-17 | 2.86E-16 | 27.92204996 |
| **ZDHHC16** | 0.894635 | 3.700778 | 8.769854 | 4.54E-17 | 2.88E-16 | 27.91592968 |
| **TMEM106B** | 1.264372 | 2.988742 | 8.769143 | 4.56E-17 | 2.89E-16 | 27.91068127 |
| **IRF2** | 1.069922 | 2.902964 | 8.768091 | 4.60E-17 | 2.92E-16 | 27.90290574 |
| **JAGN1** | 0.993408 | 3.902334 | 8.76759 | 4.62E-17 | 2.93E-16 | 27.89920714 |
| **ATP5J2-PTCD1** | 0.515219 | 0.699508 | 8.767329 | 4.63E-17 | 2.93E-16 | 27.89727749 |
| **NOLC1** | 1.123232 | 4.009268 | 8.766947 | 4.64E-17 | 2.94E-16 | 27.89445547 |
| **VMA21** | 0.946212 | 3.525391 | 8.76621 | 4.67E-17 | 2.95E-16 | 27.88901049 |
| **UBE2O** | 0.974336 | 3.598588 | 8.765967 | 4.67E-17 | 2.96E-16 | 27.88721565 |
| **API5** | 1.206093 | 4.185365 | 8.765065 | 4.71E-17 | 2.98E-16 | 27.88055487 |
| **RUFY1** | 1.021861 | 4.025298 | 8.764757 | 4.72E-17 | 2.98E-16 | 27.87828318 |
| **PTMA** | 1.679498 | 8.008567 | 8.764686 | 4.72E-17 | 2.99E-16 | 27.877756 |
| **CSTF2T** | 0.988242 | 3.140745 | 8.764577 | 4.72E-17 | 2.99E-16 | 27.87694948 |
| **NCOR2** | 1.2284 | 5.732433 | 8.764292 | 4.73E-17 | 2.99E-16 | 27.87484849 |
| **SLC35A2** | 0.956031 | 3.548492 | 8.764086 | 4.74E-17 | 3.00E-16 | 27.87332723 |
| **VPS4B** | 0.936727 | 2.992447 | 8.762938 | 4.78E-17 | 3.02E-16 | 27.86485277 |
| **RBM22** | 1.077548 | 4.48072 | 8.760445 | 4.87E-17 | 3.08E-16 | 27.84644398 |
| **KARS** | 1.23506 | 5.242061 | 8.760363 | 4.87E-17 | 3.08E-16 | 27.84583644 |
| **DIP2C** | 0.962623 | 2.947756 | 8.760287 | 4.88E-17 | 3.08E-16 | 27.84527645 |
| **ZNF44** | 0.679635 | 2.019695 | 8.759322 | 4.91E-17 | 3.10E-16 | 27.83815295 |
| **ARMC1** | 1.116352 | 3.301983 | 8.75908 | 4.92E-17 | 3.11E-16 | 27.83636588 |
| **TRPM7** | 0.880156 | 1.939778 | 8.758884 | 4.93E-17 | 3.11E-16 | 27.83492162 |
| **JAM3** | 1.148687 | 4.422046 | 8.758161 | 4.96E-17 | 3.13E-16 | 27.82958326 |
| **KAT6A** | 1.036978 | 2.639551 | 8.758135 | 4.96E-17 | 3.13E-16 | 27.8293892 |
| **PEX2** | 1.112737 | 3.792388 | 8.757763 | 4.97E-17 | 3.13E-16 | 27.82664666 |
| **DLAT** | 0.9796 | 2.91274 | 8.756591 | 5.01E-17 | 3.16E-16 | 27.81799309 |
| **ATP8B2** | 0.955971 | 2.778349 | 8.756514 | 5.02E-17 | 3.16E-16 | 27.81742776 |
| **TXLNG** | 0.812137 | 2.335192 | 8.755948 | 5.04E-17 | 3.17E-16 | 27.81324786 |
| **ZNF222** | 0.683956 | 1.81544 | 8.755851 | 5.04E-17 | 3.18E-16 | 27.81253759 |
| **HSPA13** | 1.216493 | 3.567758 | 8.755632 | 5.05E-17 | 3.18E-16 | 27.81091953 |
| **MAP3K14** | 0.790063 | 2.554146 | 8.755449 | 5.06E-17 | 3.18E-16 | 27.80956635 |
| **DAP3** | 1.227348 | 5.38659 | 8.754571 | 5.09E-17 | 3.20E-16 | 27.80308781 |
| **H3F3AP4** | 1.525925 | 5.244324 | 8.754447 | 5.10E-17 | 3.21E-16 | 27.8021697 |
| **EIF2AK1** | 1.303947 | 4.707437 | 8.75431 | 5.10E-17 | 3.21E-16 | 27.80116492 |
| **FAM133B** | 1.145573 | 3.460108 | 8.753684 | 5.12E-17 | 3.22E-16 | 27.79654558 |
| **WDR27** | 1.046384 | 3.714869 | 8.753616 | 5.13E-17 | 3.22E-16 | 27.796041 |
| **UBE2S** | 1.451943 | 5.935918 | 8.753279 | 5.14E-17 | 3.23E-16 | 27.79355619 |
| **ZNF91** | 0.916271 | 2.958136 | 8.753012 | 5.15E-17 | 3.24E-16 | 27.79158112 |
| **USP18** | 0.797321 | 2.018507 | 8.752831 | 5.16E-17 | 3.24E-16 | 27.79025057 |
| **MAPKAP1** | 1.083876 | 5.006833 | 8.752764 | 5.16E-17 | 3.24E-16 | 27.78975341 |
| **ITGAL** | 0.752232 | 1.552436 | 8.752583 | 5.17E-17 | 3.24E-16 | 27.78841813 |
| **NUP93** | 1.116994 | 4.457645 | 8.7524 | 5.17E-17 | 3.25E-16 | 27.78707058 |
| **ZDHHC8** | 1.144302 | 4.368459 | 8.752166 | 5.18E-17 | 3.25E-16 | 27.78534639 |
| **PRKAB2** | 0.93187 | 2.668944 | 8.750816 | 5.24E-17 | 3.28E-16 | 27.77538552 |
| **PPIP5K2** | 1.032379 | 2.945787 | 8.750325 | 5.26E-17 | 3.30E-16 | 27.77175808 |
| **FAM63A** | 0.993229 | 3.63602 | 8.750018 | 5.27E-17 | 3.30E-16 | 27.76949991 |
| **GNB4** | 1.202745 | 2.301369 | 8.749921 | 5.27E-17 | 3.30E-16 | 27.76878291 |
| **GPATCH2L** | 1.055884 | 2.433182 | 8.749898 | 5.27E-17 | 3.30E-16 | 27.76861336 |
| **NUTM2A-AS1** | 0.924513 | 3.536216 | 8.74885 | 5.31E-17 | 3.33E-16 | 27.76088288 |
| **CBWD2** | 1.017967 | 3.479835 | 8.748364 | 5.33E-17 | 3.34E-16 | 27.75729933 |
| **USP9X** | 1.027176 | 2.969191 | 8.748083 | 5.34E-17 | 3.35E-16 | 27.75522167 |
| **ACO1** | 0.89625 | 3.082335 | 8.747117 | 5.38E-17 | 3.37E-16 | 27.74810148 |
| **EFS** | 1.329592 | 5.157757 | 8.747059 | 5.39E-17 | 3.37E-16 | 27.74767013 |
| **PSME4** | 0.871633 | 3.3482 | 8.746975 | 5.39E-17 | 3.37E-16 | 27.74705002 |
| **AGGF1** | 0.945578 | 2.907397 | 8.746635 | 5.40E-17 | 3.38E-16 | 27.74454461 |
| **IGDCC4** | 1.035579 | 2.304788 | 8.746295 | 5.42E-17 | 3.39E-16 | 27.7420392 |
| **SPRY4** | 1.169164 | 1.76426 | 8.74607 | 5.43E-17 | 3.39E-16 | 27.74037991 |
| **PHB2** | 1.439798 | 6.597005 | 8.745758 | 5.44E-17 | 3.40E-16 | 27.7380757 |
| **THOC3** | 0.997611 | 3.655981 | 8.745403 | 5.45E-17 | 3.41E-16 | 27.73546031 |
| **TOX4** | 1.035731 | 3.800359 | 8.74523 | 5.46E-17 | 3.41E-16 | 27.73418819 |
| **RAP1A** | 1.317868 | 4.435108 | 8.745102 | 5.47E-17 | 3.41E-16 | 27.73323861 |
| **RP4-773A18.4** | 0.550992 | 1.332317 | 8.744893 | 5.47E-17 | 3.42E-16 | 27.73170245 |
| **UBE2E1** | 1.361257 | 5.812788 | 8.744876 | 5.47E-17 | 3.42E-16 | 27.73157206 |
| **ZNF277** | 0.849787 | 3.050711 | 8.744516 | 5.49E-17 | 3.43E-16 | 27.72891749 |
| **LRRFIP1** | 1.100986 | 3.65099 | 8.744403 | 5.49E-17 | 3.43E-16 | 27.7280888 |
| **SYNJ2BP** | 0.789628 | 2.063294 | 8.744213 | 5.50E-17 | 3.43E-16 | 27.72668616 |
| **KRIT1** | 1.143004 | 3.403074 | 8.74417 | 5.50E-17 | 3.43E-16 | 27.72636552 |
| **KIAA1328** | 0.54818 | 1.705167 | 8.742249 | 5.58E-17 | 3.48E-16 | 27.71220388 |
| **SLC24A1** | 0.619169 | 1.615962 | 8.740032 | 5.68E-17 | 3.54E-16 | 27.6958648 |
| **CYTL1** | 0.984035 | 2.323648 | 8.739713 | 5.69E-17 | 3.54E-16 | 27.69351367 |
| **TAB2** | 1.121473 | 3.566501 | 8.739645 | 5.69E-17 | 3.55E-16 | 27.69301321 |
| **HPS6** | 0.798207 | 3.062495 | 8.739205 | 5.71E-17 | 3.56E-16 | 27.68976952 |
| **TOP3B** | 0.955967 | 3.628161 | 8.73894 | 5.72E-17 | 3.56E-16 | 27.68781273 |
| **COMMD10** | 0.98933 | 3.046527 | 8.738064 | 5.76E-17 | 3.59E-16 | 27.68135632 |
| **MYH10** | 1.151246 | 3.640696 | 8.737934 | 5.77E-17 | 3.59E-16 | 27.68040183 |
| **FAM129B** | 1.047848 | 4.488945 | 8.737303 | 5.79E-17 | 3.60E-16 | 27.67574981 |
| **SDCBP** | 1.488413 | 5.421526 | 8.736519 | 5.83E-17 | 3.62E-16 | 27.66997505 |
| **RPE** | 1.028519 | 3.392372 | 8.736019 | 5.85E-17 | 3.64E-16 | 27.66628805 |
| **ZNF578** | 0.708645 | 1.05449 | 8.735492 | 5.87E-17 | 3.65E-16 | 27.66240671 |
| **MMP11** | 0.838055 | 1.132022 | 8.735129 | 5.89E-17 | 3.66E-16 | 27.65973235 |
| **CCZ1** | 1.13103 | 4.740477 | 8.734732 | 5.91E-17 | 3.67E-16 | 27.65681013 |
| **DCPS** | 0.894089 | 3.402527 | 8.73441 | 5.92E-17 | 3.68E-16 | 27.65443378 |
| **THAP5** | 0.972007 | 3.170376 | 8.734006 | 5.94E-17 | 3.69E-16 | 27.65145962 |
| **STRIP1** | 1.041533 | 3.697538 | 8.733925 | 5.94E-17 | 3.69E-16 | 27.65086092 |
| **SLC37A2** | 0.726522 | 1.206299 | 8.733872 | 5.94E-17 | 3.69E-16 | 27.65047093 |
| **OLA1** | 1.270997 | 4.594565 | 8.733324 | 5.97E-17 | 3.70E-16 | 27.64643379 |
| **LUM** | 1.085161 | 1.579065 | 8.732683 | 6.00E-17 | 3.72E-16 | 27.64171567 |
| **PLXDC1** | 1.032559 | 3.280354 | 8.732534 | 6.00E-17 | 3.72E-16 | 27.64061586 |
| **DNAJC21** | 0.879272 | 2.577588 | 8.73238 | 6.01E-17 | 3.73E-16 | 27.63947739 |
| **BBS4** | 0.956238 | 3.045646 | 8.732246 | 6.02E-17 | 3.73E-16 | 27.63849622 |
| **PAPD4** | 1.01742 | 3.222602 | 8.732224 | 6.02E-17 | 3.73E-16 | 27.63833175 |
| **PMS2P3** | 0.69796 | 2.688604 | 8.731897 | 6.03E-17 | 3.74E-16 | 27.6359241 |
| **AC013394.2** | 1.42456 | 6.316667 | 8.730645 | 6.09E-17 | 3.77E-16 | 27.62670316 |
| **GRK4** | 0.766864 | 3.203143 | 8.729737 | 6.13E-17 | 3.80E-16 | 27.62001112 |
| **CAPZA2** | 1.494994 | 4.630973 | 8.729125 | 6.16E-17 | 3.81E-16 | 27.61550801 |
| **SLC39A8** | 0.792799 | 2.073769 | 8.7283 | 6.20E-17 | 3.83E-16 | 27.60943426 |
| **CYYR1** | 0.694454 | 1.682179 | 8.72785 | 6.22E-17 | 3.85E-16 | 27.60611472 |
| **ZNF785** | 0.990112 | 3.083092 | 8.727803 | 6.22E-17 | 3.85E-16 | 27.60577007 |
| **CPT1A** | 1.007816 | 2.901453 | 8.72737 | 6.24E-17 | 3.86E-16 | 27.60258507 |
| **ZNF841** | 0.700207 | 1.886428 | 8.727223 | 6.25E-17 | 3.86E-16 | 27.60150107 |
| **PPP3CC** | 0.763187 | 3.09178 | 8.726846 | 6.27E-17 | 3.87E-16 | 27.59872847 |
| **CEP72** | 0.544812 | 1.40984 | 8.726662 | 6.27E-17 | 3.88E-16 | 27.59737071 |
| **CACUL1** | 0.78152 | 2.706496 | 8.726501 | 6.28E-17 | 3.88E-16 | 27.59618222 |
| **ZNF28** | 0.875491 | 1.694534 | 8.726169 | 6.30E-17 | 3.89E-16 | 27.59374013 |
| **SYVN1** | 1.005048 | 3.957855 | 8.725838 | 6.31E-17 | 3.90E-16 | 27.59130599 |
| **BRD8** | 1.203309 | 4.852148 | 8.725497 | 6.33E-17 | 3.91E-16 | 27.58879415 |
| **DCAF5** | 1.00689 | 3.112105 | 8.725433 | 6.33E-17 | 3.91E-16 | 27.58832122 |
| **CCNG2** | 1.190958 | 2.936688 | 8.724879 | 6.36E-17 | 3.92E-16 | 27.58424616 |
| **YKT6** | 1.209547 | 5.702864 | 8.724298 | 6.39E-17 | 3.94E-16 | 27.5799628 |
| **DPM1** | 1.155227 | 4.129587 | 8.723973 | 6.40E-17 | 3.95E-16 | 27.57757229 |
| **RUNX3** | 0.806139 | 1.126213 | 8.723468 | 6.43E-17 | 3.96E-16 | 27.57385341 |
| **UBXN8** | 0.795273 | 2.693129 | 8.723114 | 6.44E-17 | 3.97E-16 | 27.57124788 |
| **CLPTM1L** | 1.133564 | 4.878516 | 8.721983 | 6.50E-17 | 4.00E-16 | 27.56292591 |
| **FAM3C** | 1.424619 | 4.6321 | 8.721849 | 6.50E-17 | 4.00E-16 | 27.56194135 |
| **USP19** | 0.934989 | 4.084485 | 8.721751 | 6.51E-17 | 4.01E-16 | 27.56121781 |
| **RP11-565P22.6** | 0.802749 | 1.012136 | 8.721179 | 6.54E-17 | 4.02E-16 | 27.55701074 |
| **IFFO2** | 0.829385 | 2.50209 | 8.720886 | 6.55E-17 | 4.03E-16 | 27.55485393 |
| **FAM175B** | 0.832947 | 2.083497 | 8.720824 | 6.55E-17 | 4.03E-16 | 27.55439614 |
| **TOX3** | 1.038978 | 2.220596 | 8.719634 | 6.61E-17 | 4.07E-16 | 27.54564138 |
| **SHB** | 0.904813 | 3.285258 | 8.71935 | 6.63E-17 | 4.07E-16 | 27.54354684 |
| **FOXF2** | 0.876512 | 1.986326 | 8.718786 | 6.65E-17 | 4.09E-16 | 27.53939659 |
| **HSF2** | 0.909411 | 2.682452 | 8.718768 | 6.66E-17 | 4.09E-16 | 27.53926905 |
| **FBXO4** | 0.806344 | 2.548102 | 8.718614 | 6.66E-17 | 4.09E-16 | 27.53813114 |
| **POLR3B** | 0.640748 | 1.774283 | 8.718191 | 6.68E-17 | 4.11E-16 | 27.53501749 |
| **CPSF4** | 1.132046 | 5.081195 | 8.717107 | 6.74E-17 | 4.14E-16 | 27.5270416 |
| **PHAX** | 1.094445 | 3.640315 | 8.71708 | 6.74E-17 | 4.14E-16 | 27.52684255 |
| **MRPL47** | 1.057374 | 4.371042 | 8.716996 | 6.74E-17 | 4.14E-16 | 27.52623043 |
| **CDH6** | 0.700913 | 1.63229 | 8.716698 | 6.76E-17 | 4.15E-16 | 27.52403484 |
| **AC007620.3** | 1.277954 | 1.913539 | 8.715732 | 6.81E-17 | 4.17E-16 | 27.51692661 |
| **STRN3** | 0.833152 | 2.483113 | 8.714978 | 6.85E-17 | 4.20E-16 | 27.51138487 |
| **RP11-345J4.5** | 1.017211 | 3.767592 | 8.71478 | 6.86E-17 | 4.20E-16 | 27.50992641 |
| **ROCK1** | 0.962653 | 2.246239 | 8.714636 | 6.86E-17 | 4.21E-16 | 27.508865 |
| **NUDT15** | 0.685207 | 2.132804 | 8.714415 | 6.88E-17 | 4.21E-16 | 27.50724022 |
| **SLC35E2B** | 1.135388 | 3.848823 | 8.71333 | 6.93E-17 | 4.25E-16 | 27.49926349 |
| **HIST2H2BD** | 0.806505 | 1.580912 | 8.713089 | 6.94E-17 | 4.25E-16 | 27.49748831 |
| **MFN1** | 0.956439 | 2.899423 | 8.712578 | 6.97E-17 | 4.27E-16 | 27.49373154 |
| **C20orf196** | 0.762078 | 2.711053 | 8.710966 | 7.05E-17 | 4.32E-16 | 27.48187243 |
| **SLC9A8** | 0.80594 | 2.846411 | 8.710841 | 7.06E-17 | 4.32E-16 | 27.48095388 |
| **MANBA** | 0.846167 | 2.617368 | 8.710516 | 7.08E-17 | 4.33E-16 | 27.47856587 |
| **POLA2** | 0.889942 | 2.99515 | 8.710376 | 7.09E-17 | 4.33E-16 | 27.47753898 |
| **RP3-324O17.4** | 0.629069 | 0.827769 | 8.709164 | 7.15E-17 | 4.37E-16 | 27.4686244 |
| **SNX7** | 0.985394 | 3.241808 | 8.7073 | 7.25E-17 | 4.43E-16 | 27.45491566 |
| **PHF5A** | 1.102737 | 3.877927 | 8.707019 | 7.27E-17 | 4.44E-16 | 27.45285392 |
| **PABPC5** | 0.722267 | 1.271676 | 8.707015 | 7.27E-17 | 4.44E-16 | 27.4528231 |
| **ZDHHC13** | 0.706005 | 1.869611 | 8.706886 | 7.27E-17 | 4.44E-16 | 27.45187382 |
| **ME2** | 1.193452 | 3.562794 | 8.704499 | 7.40E-17 | 4.52E-16 | 27.4343283 |
| **MARK2** | 0.867418 | 3.5033 | 8.704271 | 7.42E-17 | 4.53E-16 | 27.43265746 |
| **GRB2** | 1.379983 | 4.824987 | 8.704166 | 7.42E-17 | 4.53E-16 | 27.43188015 |
| **CRYZ** | 1.062064 | 2.808916 | 8.703596 | 7.45E-17 | 4.55E-16 | 27.42769637 |
| **CKAP5** | 1.237448 | 3.956739 | 8.703438 | 7.46E-17 | 4.55E-16 | 27.42653016 |
| **GLYCTK** | 0.776394 | 2.528525 | 8.702524 | 7.51E-17 | 4.58E-16 | 27.41981488 |
| **ZNF594** | 0.795153 | 1.677094 | 8.702363 | 7.52E-17 | 4.59E-16 | 27.41862832 |
| **LGALS3BP** | 1.320418 | 6.201132 | 8.702333 | 7.52E-17 | 4.59E-16 | 27.41841429 |
| **MAPK6** | 0.994753 | 3.035214 | 8.70146 | 7.57E-17 | 4.61E-16 | 27.41199418 |
| **RNF8** | 0.913283 | 3.29748 | 8.701319 | 7.58E-17 | 4.62E-16 | 27.4109607 |
| **SRI** | 1.666433 | 7.155425 | 8.701195 | 7.59E-17 | 4.62E-16 | 27.41005095 |
| **SEMA5A** | 1.104701 | 2.53077 | 8.70102 | 7.60E-17 | 4.63E-16 | 27.40876512 |
| **SAE1** | 1.178538 | 4.746806 | 8.700541 | 7.63E-17 | 4.64E-16 | 27.40524231 |
| **SLC7A7** | 0.848093 | 2.310674 | 8.70053 | 7.63E-17 | 4.64E-16 | 27.40516137 |
| **KLF12** | 0.826174 | 1.900074 | 8.700494 | 7.63E-17 | 4.64E-16 | 27.40489944 |
| **LOXL1** | 1.033812 | 1.888214 | 8.70035 | 7.64E-17 | 4.65E-16 | 27.40384037 |
| **COA4** | 1.317575 | 5.430587 | 8.699792 | 7.67E-17 | 4.66E-16 | 27.39973935 |
| **GLB1L** | 0.727414 | 2.452118 | 8.699681 | 7.67E-17 | 4.67E-16 | 27.39892586 |
| **ZNF503** | 1.009188 | 2.790191 | 8.699646 | 7.68E-17 | 4.67E-16 | 27.39866956 |
| **EXOC1** | 1.078195 | 3.798061 | 8.699618 | 7.68E-17 | 4.67E-16 | 27.39846337 |
| **C11orf58** | 1.522549 | 5.914629 | 8.699333 | 7.69E-17 | 4.67E-16 | 27.39636456 |
| **ZFAND3** | 1.289354 | 4.866446 | 8.698741 | 7.73E-17 | 4.69E-16 | 27.39201601 |
| **TLE2** | 1.564953 | 5.173927 | 8.698479 | 7.74E-17 | 4.70E-16 | 27.39009222 |
| **CCDC93** | 1.17232 | 3.192044 | 8.697915 | 7.78E-17 | 4.72E-16 | 27.38595002 |
| **TRAPPC2P1** | 0.853591 | 3.308442 | 8.69747 | 7.80E-17 | 4.74E-16 | 27.38268099 |
| **RP11-400F19.6** | 0.807846 | 3.204454 | 8.697191 | 7.82E-17 | 4.74E-16 | 27.38062873 |
| **ZNF322** | 1.008388 | 1.951955 | 8.696254 | 7.87E-17 | 4.78E-16 | 27.37374968 |
| **PARP8** | 0.847788 | 2.623784 | 8.695598 | 7.91E-17 | 4.80E-16 | 27.3689317 |
| **SLC35B4** | 0.790339 | 2.717779 | 8.694085 | 8.00E-17 | 4.85E-16 | 27.35782112 |
| **CHD4** | 1.210072 | 5.847024 | 8.693421 | 8.04E-17 | 4.87E-16 | 27.35294491 |
| **MARCKSL1** | 1.815968 | 7.865449 | 8.693155 | 8.06E-17 | 4.88E-16 | 27.35098678 |
| **TXNDC9** | 0.891444 | 3.276655 | 8.692976 | 8.07E-17 | 4.89E-16 | 27.34967313 |
| **TFB1M** | 0.663995 | 2.420854 | 8.692863 | 8.08E-17 | 4.89E-16 | 27.34884363 |
| **RNF38** | 1.060002 | 3.065894 | 8.692829 | 8.08E-17 | 4.89E-16 | 27.34859477 |
| **ZNF225** | 0.654215 | 1.227125 | 8.692455 | 8.10E-17 | 4.90E-16 | 27.3458461 |
| **NIPBL** | 0.794799 | 3.101325 | 8.692156 | 8.12E-17 | 4.91E-16 | 27.34365152 |
| **SAYSD1** | 0.844643 | 3.106169 | 8.692093 | 8.12E-17 | 4.91E-16 | 27.3431905 |
| **PMEPA1** | 1.172785 | 3.777234 | 8.691822 | 8.14E-17 | 4.92E-16 | 27.34120238 |
| **MR1** | 0.802662 | 1.691775 | 8.691663 | 8.15E-17 | 4.93E-16 | 27.34003096 |
| **2-Sep** | 1.569043 | 6.977259 | 8.690884 | 8.20E-17 | 4.95E-16 | 27.33431424 |
| **AKIRIN1** | 1.221654 | 3.947634 | 8.690719 | 8.21E-17 | 4.96E-16 | 27.33310309 |
| **LINC00998** | 1.337573 | 4.770697 | 8.69045 | 8.22E-17 | 4.97E-16 | 27.33113074 |
| **ADAP2** | 0.949995 | 2.709663 | 8.689985 | 8.25E-17 | 4.98E-16 | 27.32771677 |
| **TMEM87A** | 1.0675 | 3.952472 | 8.689405 | 8.29E-17 | 5.00E-16 | 27.32345574 |
| **TUBB2B** | 1.573854 | 7.809515 | 8.689155 | 8.30E-17 | 5.01E-16 | 27.32161875 |
| **ARIH2** | 1.108489 | 4.48067 | 8.689075 | 8.31E-17 | 5.01E-16 | 27.32103258 |
| **USP22** | 1.127655 | 5.421352 | 8.688945 | 8.31E-17 | 5.02E-16 | 27.32007874 |
| **C19orf52** | 0.832068 | 3.605301 | 8.688469 | 8.34E-17 | 5.03E-16 | 27.31658809 |
| **ATP2A1** | 0.532895 | 1.073871 | 8.687088 | 8.43E-17 | 5.08E-16 | 27.30644483 |
| **5-Mar** | 1.114203 | 3.146889 | 8.685927 | 8.50E-17 | 5.13E-16 | 27.29793024 |
| **TBK1** | 0.928331 | 2.538036 | 8.68529 | 8.54E-17 | 5.15E-16 | 27.29325266 |
| **DCLRE1C** | 0.656189 | 1.614286 | 8.685148 | 8.55E-17 | 5.15E-16 | 27.29221226 |
| **ATL2** | 1.190183 | 2.8336 | 8.684595 | 8.59E-17 | 5.17E-16 | 27.2881537 |
| **RAD50** | 0.979342 | 2.669075 | 8.684434 | 8.60E-17 | 5.18E-16 | 27.2869739 |
| **NPAT** | 0.760779 | 1.768807 | 8.684195 | 8.61E-17 | 5.19E-16 | 27.28521717 |
| **CDK7** | 0.967235 | 3.252933 | 8.683484 | 8.66E-17 | 5.21E-16 | 27.27999973 |
| **PANK2** | 0.869829 | 3.62087 | 8.683424 | 8.66E-17 | 5.21E-16 | 27.27956101 |
| **ATG4A** | 0.666023 | 2.258525 | 8.682905 | 8.70E-17 | 5.23E-16 | 27.27574864 |
| **METTL9** | 1.352582 | 5.514788 | 8.682425 | 8.73E-17 | 5.25E-16 | 27.27222686 |
| **CISD2** | 0.980791 | 3.613671 | 8.682414 | 8.73E-17 | 5.25E-16 | 27.27214584 |
| **TXNDC12** | 1.079081 | 4.330508 | 8.682381 | 8.73E-17 | 5.25E-16 | 27.27190744 |
| **HLTF** | 1.126947 | 2.917453 | 8.681654 | 8.78E-17 | 5.28E-16 | 27.26657417 |
| **TMBIM6** | 1.498272 | 7.084626 | 8.67964 | 8.91E-17 | 5.36E-16 | 27.25179519 |
| **RPA2** | 1.126533 | 4.521438 | 8.67921 | 8.94E-17 | 5.37E-16 | 27.24864702 |
| **DDX60** | 0.72677 | 1.445312 | 8.678672 | 8.98E-17 | 5.39E-16 | 27.24469869 |
| **B4GALNT1** | 1.19257 | 4.068736 | 8.678328 | 9.00E-17 | 5.40E-16 | 27.24217563 |
| **LINC00667** | 1.074307 | 3.741552 | 8.677921 | 9.03E-17 | 5.42E-16 | 27.23919053 |
| **RCE1** | 0.910994 | 3.666719 | 8.677254 | 9.07E-17 | 5.44E-16 | 27.2342966 |
| **APTX** | 0.913493 | 3.923213 | 8.677163 | 9.08E-17 | 5.45E-16 | 27.23362908 |
| **CAMK2D** | 1.146911 | 3.520571 | 8.676622 | 9.11E-17 | 5.47E-16 | 27.22966187 |
| **ZZZ3** | 0.922871 | 2.953052 | 8.676514 | 9.12E-17 | 5.47E-16 | 27.2288677 |
| **PAQR3** | 1.183037 | 2.803576 | 8.675903 | 9.16E-17 | 5.49E-16 | 27.22439088 |
| **KLHL36** | 0.908898 | 3.064143 | 8.675718 | 9.18E-17 | 5.50E-16 | 27.22302995 |
| **PLP2** | 1.125876 | 3.043527 | 8.675619 | 9.18E-17 | 5.50E-16 | 27.22230552 |
| **JKAMP** | 1.14069 | 4.026746 | 8.675462 | 9.19E-17 | 5.51E-16 | 27.22115655 |
| **ZFP14** | 0.610269 | 1.447988 | 8.675332 | 9.20E-17 | 5.51E-16 | 27.22019919 |
| **HNRNPA1P10** | 0.576898 | 0.958192 | 8.674914 | 9.23E-17 | 5.53E-16 | 27.21713599 |
| **LIPT1** | 0.767492 | 2.57984 | 8.67462 | 9.25E-17 | 5.54E-16 | 27.21498175 |
| **CSNK1D** | 1.312157 | 5.678844 | 8.674472 | 9.26E-17 | 5.54E-16 | 27.21389422 |
| **GALK2** | 0.820867 | 2.773549 | 8.674088 | 9.29E-17 | 5.56E-16 | 27.21108246 |
| **AGAP1** | 1.096852 | 3.283471 | 8.673712 | 9.31E-17 | 5.57E-16 | 27.20832584 |
| **RSRC1** | 0.967923 | 3.378417 | 8.673366 | 9.34E-17 | 5.59E-16 | 27.20578676 |
| **MRPL39** | 1.041501 | 3.334166 | 8.673202 | 9.35E-17 | 5.59E-16 | 27.20458324 |
| **MXD4** | 1.18238 | 5.530768 | 8.672929 | 9.37E-17 | 5.60E-16 | 27.20258236 |
| **SECISBP2** | 1.077578 | 3.899745 | 8.672928 | 9.37E-17 | 5.60E-16 | 27.20257783 |
| **KIAA0141** | 1.069406 | 4.403093 | 8.67292 | 9.37E-17 | 5.60E-16 | 27.20252017 |
| **ANP32E** | 1.223949 | 3.93356 | 8.672634 | 9.39E-17 | 5.61E-16 | 27.2004186 |
| **C5orf54** | 0.617909 | 1.854125 | 8.672405 | 9.41E-17 | 5.62E-16 | 27.19874352 |
| **ABL2** | 0.66046 | 2.084637 | 8.672172 | 9.42E-17 | 5.63E-16 | 27.19703655 |
| **LDB1** | 1.401126 | 4.172924 | 8.671668 | 9.46E-17 | 5.65E-16 | 27.19334092 |
| **CAD** | 0.98975 | 3.486232 | 8.671232 | 9.49E-17 | 5.66E-16 | 27.19014008 |
| **ZNF865** | 0.927382 | 3.415581 | 8.671218 | 9.49E-17 | 5.66E-16 | 27.19004134 |
| **MOB3C** | 0.822664 | 2.686352 | 8.671082 | 9.50E-17 | 5.67E-16 | 27.18904152 |
| **UBE2L6** | 1.428316 | 5.761986 | 8.670925 | 9.51E-17 | 5.67E-16 | 27.18789333 |
| **BTN2A3P** | 0.522764 | 0.739376 | 8.670911 | 9.51E-17 | 5.67E-16 | 27.18778882 |
| **GLIS2** | 1.117291 | 3.519952 | 8.670209 | 9.56E-17 | 5.70E-16 | 27.18264624 |
| **SNAPC3** | 1.097101 | 3.173935 | 8.670118 | 9.57E-17 | 5.70E-16 | 27.18197294 |
| **CEP44** | 0.824604 | 2.086509 | 8.67006 | 9.57E-17 | 5.70E-16 | 27.18155064 |
| **MLLT10** | 0.933735 | 2.488796 | 8.66989 | 9.58E-17 | 5.71E-16 | 27.18030591 |
| **CCR1** | 0.879376 | 1.744078 | 8.669547 | 9.61E-17 | 5.72E-16 | 27.17779107 |
| **RAB3GAP2** | 0.908251 | 2.42822 | 8.669457 | 9.61E-17 | 5.72E-16 | 27.17713153 |
| **USP16** | 1.072125 | 3.580172 | 8.66944 | 9.62E-17 | 5.72E-16 | 27.17701065 |
| **IPO11** | 0.737612 | 2.927059 | 8.669131 | 9.64E-17 | 5.73E-16 | 27.17474307 |
| **HDHD1** | 0.777479 | 2.587405 | 8.668401 | 9.69E-17 | 5.76E-16 | 27.16938905 |
| **HTT** | 0.866934 | 3.613784 | 8.668259 | 9.70E-17 | 5.77E-16 | 27.16834928 |
| **ZCCHC9** | 0.980331 | 3.104855 | 8.667647 | 9.74E-17 | 5.79E-16 | 27.16386463 |
| **UBE2Q1** | 1.170265 | 4.213722 | 8.665483 | 9.90E-17 | 5.88E-16 | 27.14800959 |
| **NOP14-AS1** | 0.786103 | 2.898191 | 8.664762 | 9.96E-17 | 5.91E-16 | 27.14272827 |
| **ZNF138** | 0.872595 | 2.383711 | 8.663936 | 1.00E-16 | 5.95E-16 | 27.13667157 |
| **GDF15** | 1.086424 | 1.673037 | 8.663659 | 1.00E-16 | 5.96E-16 | 27.13464814 |
| **ENTPD5** | 0.691092 | 1.781154 | 8.663494 | 1.01E-16 | 5.97E-16 | 27.13343739 |
| **SENP6** | 1.12948 | 3.974076 | 8.663464 | 1.01E-16 | 5.97E-16 | 27.13321592 |
| **FCF1P2** | 0.748442 | 2.171214 | 8.663287 | 1.01E-16 | 5.97E-16 | 27.13191903 |
| **DFNA5** | 1.171786 | 4.090869 | 8.663039 | 1.01E-16 | 5.98E-16 | 27.13010565 |
| **NRF1** | 0.774424 | 3.156999 | 8.662665 | 1.01E-16 | 6.00E-16 | 27.12736032 |
| **TMEM243** | 0.954738 | 3.460572 | 8.662139 | 1.02E-16 | 6.02E-16 | 27.12350827 |
| **TEP1** | 0.827783 | 2.512732 | 8.662091 | 1.02E-16 | 6.02E-16 | 27.12315707 |
| **WASF2** | 1.08337 | 3.714535 | 8.6618 | 1.02E-16 | 6.03E-16 | 27.12102889 |
| **KLHDC10** | 0.934196 | 2.957769 | 8.660671 | 1.03E-16 | 6.08E-16 | 27.11275507 |
| **METAP1D** | 0.789508 | 2.63114 | 8.660576 | 1.03E-16 | 6.08E-16 | 27.11205958 |
| **GIGYF2** | 0.892302 | 3.53119 | 8.658772 | 1.04E-16 | 6.16E-16 | 27.09884959 |
| **MIA3** | 1.092659 | 3.818612 | 8.657148 | 1.05E-16 | 6.24E-16 | 27.0869613 |
| **LINC00909** | 0.928193 | 2.774143 | 8.656531 | 1.06E-16 | 6.27E-16 | 27.08243877 |
| **PRR5L** | 1.014382 | 2.585353 | 8.656214 | 1.06E-16 | 6.28E-16 | 27.08012234 |
| **COPA** | 1.337921 | 4.518676 | 8.656076 | 1.06E-16 | 6.28E-16 | 27.0791072 |
| **PAXIP1-AS1** | 0.89422 | 3.074862 | 8.655255 | 1.07E-16 | 6.32E-16 | 27.07309835 |
| **B2M** | 2.266759 | 10.36333 | 8.655088 | 1.07E-16 | 6.33E-16 | 27.07187749 |
| **SRD5A1** | 0.833937 | 2.678642 | 8.654622 | 1.07E-16 | 6.35E-16 | 27.06846134 |
| **BRD2** | 1.409213 | 5.727719 | 8.654491 | 1.07E-16 | 6.35E-16 | 27.06750493 |
| **HNRNPA1P7** | 0.738559 | 1.51677 | 8.654178 | 1.08E-16 | 6.36E-16 | 27.06521376 |
| **HK2** | 0.891107 | 1.732232 | 8.653293 | 1.08E-16 | 6.41E-16 | 27.05873176 |
| **IER2** | 1.310074 | 5.205608 | 8.653232 | 1.08E-16 | 6.41E-16 | 27.05828905 |
| **MAP2K7** | 0.97405 | 4.261571 | 8.65286 | 1.09E-16 | 6.42E-16 | 27.0555667 |
| **SENP2** | 1.050161 | 3.165686 | 8.651116 | 1.10E-16 | 6.51E-16 | 27.04280273 |
| **WDR35** | 0.832522 | 2.21311 | 8.650827 | 1.10E-16 | 6.52E-16 | 27.04068579 |
| **RP11-697E2.7** | 0.813184 | 1.976754 | 8.650808 | 1.10E-16 | 6.52E-16 | 27.0405471 |
| **IP6K2** | 1.377963 | 5.781762 | 8.649105 | 1.12E-16 | 6.60E-16 | 27.02808259 |
| **XPO7** | 1.034956 | 3.617078 | 8.649074 | 1.12E-16 | 6.60E-16 | 27.02786071 |
| **ELF1** | 1.131495 | 2.412639 | 8.648883 | 1.12E-16 | 6.61E-16 | 27.02645996 |
| **TGFBR2** | 1.039476 | 2.344565 | 8.648086 | 1.13E-16 | 6.64E-16 | 27.02062654 |
| **AKNAD1** | 0.689228 | 1.240019 | 8.647898 | 1.13E-16 | 6.65E-16 | 27.01925124 |
| **PXDC1** | 1.140413 | 4.247531 | 8.647717 | 1.13E-16 | 6.66E-16 | 27.01792471 |
| **9-Mar** | 1.068083 | 4.16666 | 8.646388 | 1.14E-16 | 6.72E-16 | 27.00820617 |
| **CPSF7** | 1.217139 | 4.589329 | 8.64626 | 1.14E-16 | 6.73E-16 | 27.00727104 |
| **IGFBP7** | 1.691331 | 7.429029 | 8.645282 | 1.15E-16 | 6.77E-16 | 27.0001132 |
| **TMED9** | 1.255404 | 5.756469 | 8.644761 | 1.16E-16 | 6.80E-16 | 26.99630129 |
| **RUFY3** | 1.246824 | 5.349313 | 8.644387 | 1.16E-16 | 6.82E-16 | 26.99356573 |
| **ZZEF1** | 0.899033 | 3.037699 | 8.644219 | 1.16E-16 | 6.82E-16 | 26.99233566 |
| **TLR1** | 0.787685 | 1.238944 | 8.64416 | 1.16E-16 | 6.82E-16 | 26.99190987 |
| **ATP5SL** | 1.059305 | 4.223541 | 8.644098 | 1.16E-16 | 6.82E-16 | 26.99145323 |
| **ZNF324** | 0.738896 | 2.081349 | 8.643144 | 1.17E-16 | 6.87E-16 | 26.9844771 |
| **ANAPC7** | 1.091475 | 4.731923 | 8.642976 | 1.17E-16 | 6.88E-16 | 26.98324385 |
| **RLF** | 0.719546 | 1.703949 | 8.642907 | 1.17E-16 | 6.88E-16 | 26.98274311 |
| **ARL15** | 0.892604 | 2.435618 | 8.64264 | 1.17E-16 | 6.89E-16 | 26.98079106 |
| **ABCC4** | 0.70149 | 1.568514 | 8.642597 | 1.17E-16 | 6.89E-16 | 26.98047257 |
| **CSNK1G2** | 1.100524 | 4.911299 | 8.641174 | 1.19E-16 | 6.96E-16 | 26.97006667 |
| **GNL1** | 1.133073 | 5.284529 | 8.640894 | 1.19E-16 | 6.98E-16 | 26.96802123 |
| **PIK3C3** | 0.795057 | 3.782083 | 8.640111 | 1.20E-16 | 7.02E-16 | 26.96229726 |
| **DCX** | 1.067289 | 2.192939 | 8.63952 | 1.20E-16 | 7.05E-16 | 26.95797449 |
| **ZNF449** | 0.695422 | 1.770816 | 8.639464 | 1.20E-16 | 7.05E-16 | 26.95756345 |
| **PFDN4** | 1.198438 | 4.613377 | 8.639228 | 1.20E-16 | 7.06E-16 | 26.95584061 |
| **KIAA1549** | 0.794708 | 1.890453 | 8.638945 | 1.21E-16 | 7.07E-16 | 26.95376897 |
| **C7orf60** | 0.75549 | 2.037698 | 8.638125 | 1.21E-16 | 7.11E-16 | 26.9477711 |
| **TMEM128** | 1.203873 | 4.140451 | 8.63776 | 1.22E-16 | 7.13E-16 | 26.94510666 |
| **YIPF3** | 1.260469 | 6.04288 | 8.637252 | 1.22E-16 | 7.15E-16 | 26.94139087 |
| **SCRN3** | 1.223066 | 3.627586 | 8.636805 | 1.23E-16 | 7.18E-16 | 26.93812231 |
| **SNAPIN** | 1.330473 | 5.036394 | 8.636787 | 1.23E-16 | 7.18E-16 | 26.93799074 |
| **SIRT1** | 0.916017 | 2.512056 | 8.636273 | 1.23E-16 | 7.20E-16 | 26.93423747 |
| **ZNF740** | 0.975991 | 3.135875 | 8.636215 | 1.23E-16 | 7.20E-16 | 26.93380812 |
| **SYNE3** | 0.626077 | 1.162296 | 8.63582 | 1.23E-16 | 7.22E-16 | 26.93092638 |
| **RAD54L2** | 0.719858 | 2.193787 | 8.635145 | 1.24E-16 | 7.26E-16 | 26.9259925 |
| **CA13** | 0.602018 | 1.280613 | 8.635067 | 1.24E-16 | 7.26E-16 | 26.92541943 |
| **TP53I11** | 1.028886 | 3.44093 | 8.633392 | 1.26E-16 | 7.35E-16 | 26.91317474 |
| **LZTR1** | 1.088091 | 5.236833 | 8.632803 | 1.26E-16 | 7.38E-16 | 26.90887088 |
| **PABPC1** | 1.620839 | 7.430787 | 8.632528 | 1.27E-16 | 7.39E-16 | 26.90686448 |
| **THAP9** | 0.645037 | 1.473031 | 8.632497 | 1.27E-16 | 7.39E-16 | 26.9066372 |
| **ACIN1** | 1.314797 | 5.939898 | 8.632189 | 1.27E-16 | 7.41E-16 | 26.90438743 |
| **CEP97** | 0.793995 | 1.858227 | 8.632119 | 1.27E-16 | 7.41E-16 | 26.90387384 |
| **LARP7** | 1.180161 | 4.284328 | 8.631879 | 1.27E-16 | 7.42E-16 | 26.90212236 |
| **BBS7** | 0.800624 | 2.54028 | 8.631425 | 1.28E-16 | 7.44E-16 | 26.89880234 |
| **ZKSCAN1** | 0.738114 | 3.610773 | 8.6312 | 1.28E-16 | 7.45E-16 | 26.89715802 |
| **AP3S2** | 0.967807 | 3.654819 | 8.631074 | 1.28E-16 | 7.46E-16 | 26.89624142 |
| **MED18** | 0.724921 | 2.777678 | 8.630429 | 1.29E-16 | 7.49E-16 | 26.89152607 |
| **XRCC1** | 0.988941 | 3.841404 | 8.63029 | 1.29E-16 | 7.50E-16 | 26.89050768 |
| **MRPL19** | 1.198249 | 3.277661 | 8.63025 | 1.29E-16 | 7.50E-16 | 26.89021996 |
| **MKRN1** | 1.195764 | 5.178098 | 8.630015 | 1.29E-16 | 7.51E-16 | 26.88849872 |
| **PYGO1** | 0.696784 | 1.489827 | 8.629544 | 1.29E-16 | 7.54E-16 | 26.88505887 |
| **MAZ** | 1.41964 | 6.659623 | 8.628724 | 1.30E-16 | 7.58E-16 | 26.87906902 |
| **GALNT7** | 0.954062 | 2.228276 | 8.628407 | 1.30E-16 | 7.59E-16 | 26.87675437 |
| **PPIL1** | 1.115883 | 4.461846 | 8.627524 | 1.31E-16 | 7.64E-16 | 26.87030326 |
| **NKAIN1** | 0.974162 | 2.69763 | 8.627462 | 1.31E-16 | 7.64E-16 | 26.86984833 |
| **MED21** | 0.989087 | 2.839826 | 8.626354 | 1.32E-16 | 7.70E-16 | 26.86175407 |
| **PLEKHA4** | 1.630173 | 4.513177 | 8.625928 | 1.33E-16 | 7.73E-16 | 26.85864617 |
| **EHHADH** | 0.535366 | 1.203857 | 8.625753 | 1.33E-16 | 7.74E-16 | 26.85736911 |
| **RP11-178G16.5** | 0.99562 | 2.558624 | 8.625155 | 1.34E-16 | 7.77E-16 | 26.8530001 |
| **ZNF497** | 0.587622 | 1.789769 | 8.624958 | 1.34E-16 | 7.78E-16 | 26.85155993 |
| **ZCCHC8** | 0.978288 | 2.863462 | 8.624188 | 1.35E-16 | 7.82E-16 | 26.84593744 |
| **FBXO21** | 1.155382 | 3.940072 | 8.624091 | 1.35E-16 | 7.82E-16 | 26.84522765 |
| **SLC25A43** | 0.747435 | 1.39867 | 8.623968 | 1.35E-16 | 7.83E-16 | 26.84432987 |
| **BOP1** | 1.329598 | 4.956555 | 8.623957 | 1.35E-16 | 7.83E-16 | 26.84425373 |
| **SEMA4C** | 1.066167 | 4.069148 | 8.623539 | 1.35E-16 | 7.85E-16 | 26.84119527 |
| **SNHG1** | 1.32575 | 5.57262 | 8.623277 | 1.36E-16 | 7.86E-16 | 26.83928136 |
| **SCAF11** | 1.088961 | 3.567177 | 8.623267 | 1.36E-16 | 7.86E-16 | 26.8392127 |
| **M6PR** | 1.217969 | 5.105255 | 8.623229 | 1.36E-16 | 7.86E-16 | 26.83893777 |
| **RP11-3J10.4** | 0.778292 | 1.399955 | 8.622681 | 1.36E-16 | 7.89E-16 | 26.83493167 |
| **GOLIM4** | 1.399332 | 4.315963 | 8.622606 | 1.36E-16 | 7.89E-16 | 26.83438403 |
| **PSMD9** | 1.079732 | 4.855108 | 8.622582 | 1.36E-16 | 7.89E-16 | 26.83420653 |
| **CCDC82** | 1.282922 | 3.91666 | 8.622417 | 1.36E-16 | 7.90E-16 | 26.8330049 |
| **ATXN3** | 0.840912 | 2.115772 | 8.621916 | 1.37E-16 | 7.93E-16 | 26.82934859 |
| **RNF169** | 0.701161 | 1.274036 | 8.621519 | 1.37E-16 | 7.95E-16 | 26.82644584 |
| **DPH5** | 1.078598 | 4.309518 | 8.621336 | 1.37E-16 | 7.96E-16 | 26.82511071 |
| **KNOP1** | 1.016441 | 3.80666 | 8.621211 | 1.38E-16 | 7.97E-16 | 26.82419947 |
| **WIBG** | 1.088058 | 4.379512 | 8.621039 | 1.38E-16 | 7.97E-16 | 26.82294734 |
| **GPR56** | 1.391356 | 6.899701 | 8.620549 | 1.38E-16 | 8.00E-16 | 26.81936478 |
| **HNRNPA1P48** | 0.925824 | 3.352697 | 8.620439 | 1.38E-16 | 8.01E-16 | 26.81856707 |
| **SMO** | 1.009764 | 3.27798 | 8.619698 | 1.39E-16 | 8.05E-16 | 26.81315236 |
| **HIST1H2BK** | 0.978012 | 3.719554 | 8.618993 | 1.40E-16 | 8.09E-16 | 26.80800822 |
| **PLEKHM1** | 0.861024 | 3.932407 | 8.618782 | 1.40E-16 | 8.10E-16 | 26.80646904 |
| **SLC10A7** | 0.682754 | 1.328005 | 8.618377 | 1.41E-16 | 8.12E-16 | 26.8035136 |
| **TTC30B** | 0.514326 | 1.191873 | 8.618281 | 1.41E-16 | 8.12E-16 | 26.80280779 |
| **LINC00152** | 1.271861 | 3.373328 | 8.617916 | 1.41E-16 | 8.14E-16 | 26.80014698 |
| **SNX12** | 1.052619 | 4.711325 | 8.617727 | 1.41E-16 | 8.15E-16 | 26.79876786 |
| **RDX** | 1.309741 | 4.663763 | 8.616879 | 1.42E-16 | 8.20E-16 | 26.7925801 |
| **TTC9C** | 1.079727 | 4.244196 | 8.616131 | 1.43E-16 | 8.24E-16 | 26.78711572 |
| **RPL15** | 1.609499 | 9.015531 | 8.615622 | 1.43E-16 | 8.27E-16 | 26.78340419 |
| **WAC** | 1.271076 | 4.169859 | 8.614744 | 1.44E-16 | 8.32E-16 | 26.77699344 |
| **DIEXF** | 0.569096 | 1.425159 | 8.614348 | 1.45E-16 | 8.34E-16 | 26.77410621 |
| **BCAP29** | 1.355928 | 4.3081 | 8.614226 | 1.45E-16 | 8.35E-16 | 26.77321911 |
| **PRPF4B** | 0.984566 | 3.816656 | 8.614086 | 1.45E-16 | 8.35E-16 | 26.77219635 |
| **ZNF493** | 0.875061 | 2.658419 | 8.613609 | 1.46E-16 | 8.38E-16 | 26.76871134 |
| **GRPEL1** | 0.911665 | 3.618409 | 8.612573 | 1.47E-16 | 8.44E-16 | 26.76115621 |
| **SUPT6H** | 1.000741 | 4.503148 | 8.610909 | 1.49E-16 | 8.55E-16 | 26.74901221 |
| **POLG** | 1.019784 | 4.195874 | 8.609827 | 1.50E-16 | 8.61E-16 | 26.74112446 |
| **WLS** | 1.438371 | 5.422949 | 8.609666 | 1.50E-16 | 8.62E-16 | 26.73994512 |
| **CCNC** | 1.005096 | 3.582486 | 8.609598 | 1.50E-16 | 8.62E-16 | 26.73945588 |
| **PLRG1** | 1.101736 | 4.431554 | 8.609548 | 1.50E-16 | 8.62E-16 | 26.73909011 |
| **TRIM35** | 0.671946 | 2.466727 | 8.609497 | 1.50E-16 | 8.63E-16 | 26.73871471 |
| **ACAD10** | 0.826962 | 3.619596 | 8.609364 | 1.50E-16 | 8.63E-16 | 26.7377487 |
| **RP11-480I12.5** | 0.539014 | 1.245414 | 8.608648 | 1.51E-16 | 8.68E-16 | 26.73252419 |
| **ESYT1** | 0.994082 | 3.555473 | 8.608386 | 1.51E-16 | 8.69E-16 | 26.73061303 |
| **TMTC4** | 1.064203 | 2.912909 | 8.608166 | 1.52E-16 | 8.70E-16 | 26.72900923 |
| **PEAK1** | 0.985761 | 2.83925 | 8.608001 | 1.52E-16 | 8.71E-16 | 26.72780195 |
| **PTPRO** | 0.888558 | 2.40659 | 8.606839 | 1.53E-16 | 8.78E-16 | 26.71933178 |
| **UBE2D2** | 1.167753 | 5.134613 | 8.606456 | 1.54E-16 | 8.81E-16 | 26.71653405 |
| **SNX24** | 0.834452 | 2.937216 | 8.606134 | 1.54E-16 | 8.82E-16 | 26.71418962 |
| **PPP2R1B** | 0.800108 | 2.138723 | 8.605905 | 1.54E-16 | 8.84E-16 | 26.71251786 |
| **SEC22B** | 0.987236 | 1.969709 | 8.604158 | 1.56E-16 | 8.95E-16 | 26.69978304 |
| **KIAA0195** | 1.115522 | 5.780388 | 8.602857 | 1.58E-16 | 9.03E-16 | 26.69029725 |
| **ELOVL5** | 1.371305 | 4.584376 | 8.602839 | 1.58E-16 | 9.03E-16 | 26.69016669 |
| **RASSF3** | 0.741528 | 1.811544 | 8.601638 | 1.59E-16 | 9.11E-16 | 26.68141027 |
| **ALG13** | 1.014998 | 3.658076 | 8.601532 | 1.59E-16 | 9.12E-16 | 26.68063704 |
| **SLC43A1** | 0.657035 | 2.083791 | 8.601262 | 1.60E-16 | 9.13E-16 | 26.67866613 |
| **FAM134C** | 0.812767 | 3.727949 | 8.60064 | 1.60E-16 | 9.17E-16 | 26.67413297 |
| **ACAD9** | 0.945517 | 4.248655 | 8.600322 | 1.61E-16 | 9.19E-16 | 26.67181601 |
| **CTD-2368P22.1** | 0.937546 | 2.4486 | 8.59942 | 1.62E-16 | 9.25E-16 | 26.66523964 |
| **SLC36A4** | 0.995586 | 2.244856 | 8.599018 | 1.62E-16 | 9.27E-16 | 26.66231292 |
| **ZNF513** | 1.079419 | 4.377025 | 8.597831 | 1.64E-16 | 9.35E-16 | 26.65366178 |
| **EDC4** | 0.984998 | 4.336366 | 8.597519 | 1.64E-16 | 9.37E-16 | 26.65138675 |
| **FKBP1A** | 1.507271 | 6.465141 | 8.597335 | 1.64E-16 | 9.38E-16 | 26.65004962 |
| **EFCAB11** | 0.710373 | 2.21988 | 8.59705 | 1.65E-16 | 9.40E-16 | 26.64797482 |
| **RSRC2** | 1.318989 | 4.529523 | 8.595681 | 1.66E-16 | 9.49E-16 | 26.63799889 |
| **AFF4** | 1.061464 | 2.950983 | 8.595628 | 1.66E-16 | 9.50E-16 | 26.63761056 |
| **EMP1** | 1.338121 | 3.823341 | 8.59558 | 1.66E-16 | 9.50E-16 | 26.63726177 |
| **RP3-399L15.3** | 0.533423 | 1.494829 | 8.5955 | 1.66E-16 | 9.50E-16 | 26.63667426 |
| **DCTN4** | 1.110434 | 4.467843 | 8.593913 | 1.68E-16 | 9.61E-16 | 26.62511932 |
| **IBA57** | 0.561127 | 1.676679 | 8.593488 | 1.69E-16 | 9.64E-16 | 26.62202007 |
| **MPZL2** | 0.583826 | 0.698192 | 8.593427 | 1.69E-16 | 9.64E-16 | 26.62157884 |
| **ING4** | 1.181321 | 5.419562 | 8.592816 | 1.70E-16 | 9.68E-16 | 26.6171239 |
| **THOC6** | 1.013617 | 4.086522 | 8.592804 | 1.70E-16 | 9.68E-16 | 26.61703662 |
| **RBM4** | 1.27899 | 6.24359 | 8.592199 | 1.71E-16 | 9.72E-16 | 26.61263158 |
| **XXbac-BPG252P9.9** | 1.941896 | 3.588804 | 8.591604 | 1.71E-16 | 9.76E-16 | 26.60829586 |
| **CANX** | 1.419347 | 6.288645 | 8.591461 | 1.72E-16 | 9.77E-16 | 26.60725215 |
| **XRCC3** | 0.80489 | 3.154717 | 8.591053 | 1.72E-16 | 9.80E-16 | 26.60428417 |
| **C6orf211** | 0.822261 | 2.41534 | 8.590918 | 1.72E-16 | 9.80E-16 | 26.60330201 |
| **ATG5** | 0.75495 | 2.794386 | 8.589142 | 1.75E-16 | 9.93E-16 | 26.59036899 |
| **TOP2B** | 1.374795 | 5.203991 | 8.588107 | 1.76E-16 | 1.00E-15 | 26.5828298 |
| **XPNPEP1** | 0.99664 | 4.38232 | 8.588066 | 1.76E-16 | 1.00E-15 | 26.5825317 |
| **CUL2** | 0.968938 | 2.833501 | 8.587232 | 1.77E-16 | 1.01E-15 | 26.57646212 |
| **CTNNA1** | 1.31637 | 6.024535 | 8.587112 | 1.77E-16 | 1.01E-15 | 26.57558163 |
| **RP11-284F21.10** | 0.665791 | 1.764331 | 8.586397 | 1.78E-16 | 1.01E-15 | 26.57037777 |
| **HECA** | 0.75465 | 2.40512 | 8.585663 | 1.79E-16 | 1.02E-15 | 26.56503763 |
| **TPRKB** | 1.197387 | 4.766847 | 8.585316 | 1.80E-16 | 1.02E-15 | 26.56251062 |
| **DIP2A** | 1.069748 | 3.540862 | 8.5853 | 1.80E-16 | 1.02E-15 | 26.56239487 |
| **PEX12** | 0.8943 | 2.507194 | 8.585052 | 1.80E-16 | 1.02E-15 | 26.5605896 |
| **TFAP2A** | 0.996079 | 1.824274 | 8.584247 | 1.81E-16 | 1.03E-15 | 26.55472941 |
| **CPD** | 0.885913 | 2.282206 | 8.584243 | 1.81E-16 | 1.03E-15 | 26.55469599 |
| **ZBTB22** | 0.929209 | 3.576765 | 8.583957 | 1.81E-16 | 1.03E-15 | 26.55261601 |
| **PAPOLG** | 0.746134 | 1.775649 | 8.582358 | 1.83E-16 | 1.04E-15 | 26.54097743 |
| **KCTD21-AS1** | 0.563179 | 1.458805 | 8.581853 | 1.84E-16 | 1.04E-15 | 26.53730314 |
| **IFIH1** | 0.729701 | 1.612497 | 8.580301 | 1.86E-16 | 1.06E-15 | 26.52600896 |
| **TNPO2** | 1.194155 | 4.792166 | 8.580008 | 1.87E-16 | 1.06E-15 | 26.52387599 |
| **BRD7** | 1.222237 | 5.073014 | 8.579698 | 1.87E-16 | 1.06E-15 | 26.52161867 |
| **ZNF879** | 0.554064 | 1.253248 | 8.5796 | 1.87E-16 | 1.06E-15 | 26.52091125 |
| **USP3** | 0.923786 | 3.673447 | 8.57956 | 1.87E-16 | 1.06E-15 | 26.52061592 |
| **PUM2** | 0.991524 | 3.918408 | 8.578565 | 1.89E-16 | 1.07E-15 | 26.51337851 |
| **TUBGCP3** | 1.11354 | 3.336338 | 8.578451 | 1.89E-16 | 1.07E-15 | 26.51254387 |
| **MRPL15** | 1.159253 | 4.540971 | 8.578019 | 1.89E-16 | 1.07E-15 | 26.50940514 |
| **KIAA0319L** | 0.99778 | 3.998091 | 8.577545 | 1.90E-16 | 1.08E-15 | 26.50595442 |
| **ZNF271** | 0.868277 | 3.122262 | 8.577321 | 1.90E-16 | 1.08E-15 | 26.50432543 |
| **DNAJC10** | 1.110667 | 3.321402 | 8.577146 | 1.91E-16 | 1.08E-15 | 26.50305025 |
| **ASH1L-AS1** | 0.579959 | 1.904821 | 8.577062 | 1.91E-16 | 1.08E-15 | 26.50244568 |
| **ABHD2** | 1.072327 | 3.568882 | 8.576836 | 1.91E-16 | 1.08E-15 | 26.50079689 |
| **PPP1R14B** | 1.588708 | 7.155235 | 8.576668 | 1.91E-16 | 1.08E-15 | 26.49957903 |
| **ZNF772** | 0.756864 | 1.553853 | 8.576618 | 1.91E-16 | 1.08E-15 | 26.49921054 |
| **MREG** | 0.92633 | 1.636446 | 8.576513 | 1.92E-16 | 1.08E-15 | 26.49844671 |
| **GTF2I** | 1.305475 | 6.846139 | 8.576051 | 1.92E-16 | 1.09E-15 | 26.49508535 |
| **SAMD1** | 1.230716 | 5.215394 | 8.576047 | 1.92E-16 | 1.09E-15 | 26.4950596 |
| **EIF4G3** | 1.136875 | 3.605568 | 8.575793 | 1.93E-16 | 1.09E-15 | 26.49321048 |
| **PACS1** | 1.05968 | 4.343538 | 8.575439 | 1.93E-16 | 1.09E-15 | 26.4906378 |
| **R3HCC1L** | 0.54676 | 1.918186 | 8.575289 | 1.93E-16 | 1.09E-15 | 26.48954228 |
| **TATDN1** | 1.068221 | 4.137265 | 8.575033 | 1.94E-16 | 1.09E-15 | 26.48768562 |
| **VRK3** | 0.918181 | 3.735639 | 8.574333 | 1.95E-16 | 1.10E-15 | 26.48258799 |
| **AP5S1** | 0.75972 | 2.955139 | 8.574095 | 1.95E-16 | 1.10E-15 | 26.48085887 |
| **PMS1** | 0.805768 | 2.975986 | 8.57358 | 1.96E-16 | 1.10E-15 | 26.47711538 |
| **C1orf27** | 0.832716 | 2.736948 | 8.572626 | 1.97E-16 | 1.11E-15 | 26.47017605 |
| **TSSK6** | 0.510113 | 1.262504 | 8.572205 | 1.98E-16 | 1.11E-15 | 26.46711128 |
| **DERA** | 1.087803 | 3.853175 | 8.571205 | 1.99E-16 | 1.12E-15 | 26.45984521 |
| **MECOM** | 0.653627 | 1.100659 | 8.571065 | 1.99E-16 | 1.12E-15 | 26.45882416 |
| **RP11-46D6.1** | 0.694778 | 1.705431 | 8.570459 | 2.00E-16 | 1.13E-15 | 26.45441623 |
| **ITPKC** | 0.812438 | 2.681882 | 8.570056 | 2.01E-16 | 1.13E-15 | 26.45149074 |
| **PTPN13** | 1.073392 | 2.413718 | 8.569662 | 2.02E-16 | 1.13E-15 | 26.44862689 |
| **PICALM** | 1.127287 | 4.917334 | 8.569281 | 2.02E-16 | 1.14E-15 | 26.44585362 |
| **LRBA** | 0.828002 | 2.201732 | 8.56923 | 2.02E-16 | 1.14E-15 | 26.44548113 |
| **CRMP1** | 1.396817 | 6.134228 | 8.569072 | 2.02E-16 | 1.14E-15 | 26.44433224 |
| **TEAD1** | 1.100538 | 2.501383 | 8.5689 | 2.03E-16 | 1.14E-15 | 26.44308226 |
| **H1F0** | 1.519326 | 6.289724 | 8.56815 | 2.04E-16 | 1.15E-15 | 26.4376292 |
| **RAB2A** | 1.400289 | 5.349373 | 8.567092 | 2.05E-16 | 1.15E-15 | 26.42993924 |
| **INO80C** | 1.00852 | 3.952195 | 8.56704 | 2.05E-16 | 1.15E-15 | 26.42956068 |
| **NAGLU** | 1.063247 | 4.523951 | 8.567028 | 2.05E-16 | 1.15E-15 | 26.42947579 |
| **FAM20C** | 1.260606 | 4.726992 | 8.566431 | 2.06E-16 | 1.16E-15 | 26.42513457 |
| **FURIN** | 0.974556 | 3.788119 | 8.566128 | 2.07E-16 | 1.16E-15 | 26.42293538 |
| **DEPDC7** | 0.595813 | 1.395824 | 8.565462 | 2.08E-16 | 1.17E-15 | 26.41809544 |
| **ZNF737** | 0.98513 | 1.894443 | 8.56504 | 2.09E-16 | 1.17E-15 | 26.41502563 |
| **EN1** | 0.797223 | 0.686134 | 8.564204 | 2.10E-16 | 1.18E-15 | 26.40894892 |
| **IGFBP5** | 1.509526 | 4.114858 | 8.563336 | 2.11E-16 | 1.18E-15 | 26.40264138 |
| **PHF3** | 0.969535 | 2.667209 | 8.56326 | 2.11E-16 | 1.18E-15 | 26.40208936 |
| **DCK** | 1.041664 | 2.974843 | 8.563024 | 2.12E-16 | 1.19E-15 | 26.40037664 |
| **RPA3** | 1.038467 | 4.028176 | 8.562806 | 2.12E-16 | 1.19E-15 | 26.39879076 |
| **CREBZF** | 1.341987 | 4.095745 | 8.562721 | 2.12E-16 | 1.19E-15 | 26.39817423 |
| **PIM3** | 0.997463 | 3.8005 | 8.562358 | 2.13E-16 | 1.19E-15 | 26.39553863 |
| **LRRK2** | 0.984981 | 1.634465 | 8.562186 | 2.13E-16 | 1.19E-15 | 26.39428429 |
| **ARID4B** | 0.97194 | 3.045509 | 8.561562 | 2.14E-16 | 1.20E-15 | 26.38974928 |
| **NBPF9** | 0.941928 | 2.832228 | 8.56149 | 2.14E-16 | 1.20E-15 | 26.38922579 |
| **ATP11B** | 0.842534 | 1.956119 | 8.561076 | 2.15E-16 | 1.20E-15 | 26.38622139 |
| **THAP11** | 1.090383 | 4.500678 | 8.55946 | 2.17E-16 | 1.22E-15 | 26.37448189 |
| **CHTOP** | 1.204676 | 5.340945 | 8.55931 | 2.18E-16 | 1.22E-15 | 26.37338966 |
| **USP49** | 0.911833 | 2.132573 | 8.559207 | 2.18E-16 | 1.22E-15 | 26.37263982 |
| **CHUK** | 0.833673 | 2.438337 | 8.55896 | 2.18E-16 | 1.22E-15 | 26.37085051 |
| **LRCH1** | 0.696516 | 1.638316 | 8.558194 | 2.19E-16 | 1.23E-15 | 26.36528707 |
| **ZBTB1** | 0.977588 | 2.868094 | 8.557749 | 2.20E-16 | 1.23E-15 | 26.36205167 |
| **SEC11A** | 1.350608 | 5.76575 | 8.556855 | 2.22E-16 | 1.24E-15 | 26.35555578 |
| **RP11-295K3.1** | 0.746569 | 1.570523 | 8.55635 | 2.22E-16 | 1.24E-15 | 26.35188865 |
| **RP11-85F14.5** | 0.750194 | 2.224391 | 8.556213 | 2.23E-16 | 1.24E-15 | 26.35089849 |
| **TMEM107** | 0.928021 | 3.332144 | 8.554497 | 2.25E-16 | 1.26E-15 | 26.33843293 |
| **FBXO22** | 0.942536 | 2.558346 | 8.554364 | 2.26E-16 | 1.26E-15 | 26.33747249 |
| **ZXDA** | 0.588104 | 1.199259 | 8.553619 | 2.27E-16 | 1.27E-15 | 26.33205819 |
| **HPS4** | 0.972319 | 3.925213 | 8.553039 | 2.28E-16 | 1.27E-15 | 26.32784823 |
| **JARID2** | 0.783582 | 2.263309 | 8.552796 | 2.28E-16 | 1.27E-15 | 26.32608309 |
| **GZF1** | 0.855625 | 2.720274 | 8.55231 | 2.29E-16 | 1.28E-15 | 26.32255882 |
| **PDGFC** | 0.921445 | 2.480879 | 8.551981 | 2.30E-16 | 1.28E-15 | 26.32016651 |
| **SMS** | 1.158895 | 4.42905 | 8.551814 | 2.30E-16 | 1.28E-15 | 26.31895081 |
| **LONRF1** | 0.9499 | 2.962546 | 8.551659 | 2.30E-16 | 1.28E-15 | 26.31782645 |
| **MED19** | 1.081978 | 4.254594 | 8.551262 | 2.31E-16 | 1.29E-15 | 26.31494487 |
| **C19orf57** | 0.937041 | 3.25543 | 8.550904 | 2.31E-16 | 1.29E-15 | 26.31234454 |
| **SNRK** | 0.912199 | 2.673366 | 8.550348 | 2.32E-16 | 1.29E-15 | 26.30830965 |
| **ZKSCAN7** | 0.616423 | 1.646312 | 8.550008 | 2.33E-16 | 1.30E-15 | 26.30584282 |
| **TMEM64** | 0.771382 | 1.801348 | 8.549507 | 2.34E-16 | 1.30E-15 | 26.30220474 |
| **C9orf156** | 0.817921 | 3.033262 | 8.54865 | 2.35E-16 | 1.31E-15 | 26.29598741 |
| **ZNF780B** | 0.805208 | 2.189757 | 8.547519 | 2.37E-16 | 1.32E-15 | 26.28777722 |
| **C19orf12** | 0.934956 | 3.435759 | 8.547427 | 2.37E-16 | 1.32E-15 | 26.2871121 |
| **ARHGEF7** | 1.184275 | 4.757525 | 8.54737 | 2.38E-16 | 1.32E-15 | 26.28669849 |
| **ADAMTS1** | 0.936597 | 1.757134 | 8.547176 | 2.38E-16 | 1.32E-15 | 26.2852885 |
| **HELZ** | 0.784141 | 1.883926 | 8.546916 | 2.38E-16 | 1.32E-15 | 26.2834039 |
| **PROM1** | 0.876927 | 1.966439 | 8.546775 | 2.39E-16 | 1.33E-15 | 26.28237908 |
| **DNAJA3** | 1.000316 | 4.033916 | 8.54661 | 2.39E-16 | 1.33E-15 | 26.28118373 |
| **CBX2** | 0.857601 | 2.649654 | 8.546583 | 2.39E-16 | 1.33E-15 | 26.28098517 |
| **LIG1** | 1.006303 | 3.931189 | 8.546292 | 2.39E-16 | 1.33E-15 | 26.27887233 |
| **FOS** | 1.58675 | 5.232443 | 8.545091 | 2.42E-16 | 1.34E-15 | 26.27016005 |
| **AIMP2** | 1.129977 | 4.131828 | 8.545026 | 2.42E-16 | 1.34E-15 | 26.2696846 |
| **EIF3I** | 1.296252 | 5.79313 | 8.54488 | 2.42E-16 | 1.34E-15 | 26.26862709 |
| **GS1-124K5.12** | 0.654152 | 1.548989 | 8.544025 | 2.44E-16 | 1.35E-15 | 26.26242555 |
| **PDIA3** | 1.259265 | 6.204467 | 8.543381 | 2.45E-16 | 1.36E-15 | 26.25775483 |
| **PBX2** | 1.144801 | 4.157913 | 8.542349 | 2.47E-16 | 1.37E-15 | 26.25026477 |
| **VIMP** | 1.207106 | 4.97828 | 8.542055 | 2.47E-16 | 1.37E-15 | 26.24813493 |
| **PTEN** | 0.848638 | 3.214826 | 8.541868 | 2.47E-16 | 1.37E-15 | 26.24677781 |
| **ZMYM2** | 1.094677 | 3.585618 | 8.541245 | 2.49E-16 | 1.38E-15 | 26.24225933 |
| **NCOA6** | 0.896826 | 3.315541 | 8.541062 | 2.49E-16 | 1.38E-15 | 26.24093066 |
| **RAB32** | 0.979142 | 2.796603 | 8.541007 | 2.49E-16 | 1.38E-15 | 26.24052805 |
| **RBM4B** | 1.120337 | 4.18455 | 8.540989 | 2.49E-16 | 1.38E-15 | 26.24040294 |
| **ARHGEF11** | 1.008516 | 3.393425 | 8.540089 | 2.51E-16 | 1.39E-15 | 26.23387051 |
| **ZDHHC6** | 1.01008 | 3.585379 | 8.539253 | 2.52E-16 | 1.40E-15 | 26.22781059 |
| **PANK4** | 0.96849 | 3.874504 | 8.539206 | 2.52E-16 | 1.40E-15 | 26.22746658 |
| **MLLT1** | 1.36045 | 3.661125 | 8.539093 | 2.53E-16 | 1.40E-15 | 26.22665166 |
| **TM9SF3** | 1.111539 | 3.894752 | 8.538823 | 2.53E-16 | 1.40E-15 | 26.22468809 |
| **KDSR** | 1.070906 | 3.414352 | 8.538748 | 2.53E-16 | 1.40E-15 | 26.22414954 |
| **RPL26L1** | 1.2261 | 4.989651 | 8.538402 | 2.54E-16 | 1.40E-15 | 26.2216397 |
| **RAB9A** | 1.122104 | 3.908694 | 8.538116 | 2.54E-16 | 1.41E-15 | 26.21956202 |
| **AC083799.1** | 0.504722 | 1.341035 | 8.537501 | 2.56E-16 | 1.41E-15 | 26.21510592 |
| **ZC3H11A** | 1.236854 | 4.380509 | 8.537276 | 2.56E-16 | 1.41E-15 | 26.21347331 |
| **CDK19** | 0.900832 | 3.060123 | 8.536843 | 2.57E-16 | 1.42E-15 | 26.2103295 |
| **EHD2** | 1.080432 | 3.580803 | 8.536829 | 2.57E-16 | 1.42E-15 | 26.21023287 |
| **SLC30A9** | 1.094795 | 3.804907 | 8.536661 | 2.57E-16 | 1.42E-15 | 26.20901204 |
| **VAPA** | 1.57066 | 5.165534 | 8.536229 | 2.58E-16 | 1.42E-15 | 26.20588193 |
| **FAM96A** | 1.252936 | 4.737629 | 8.535604 | 2.59E-16 | 1.43E-15 | 26.20134865 |
| **TTC21B** | 0.722557 | 2.402547 | 8.535587 | 2.59E-16 | 1.43E-15 | 26.20122375 |
| **ARL13B** | 0.863862 | 1.843364 | 8.535104 | 2.60E-16 | 1.43E-15 | 26.19772261 |
| **MED13L** | 1.11473 | 2.812425 | 8.535077 | 2.60E-16 | 1.43E-15 | 26.19753138 |
| **ANKHD1** | 1.098676 | 4.334985 | 8.534849 | 2.61E-16 | 1.44E-15 | 26.19587363 |
| **CASP7** | 0.812376 | 1.933048 | 8.534466 | 2.61E-16 | 1.44E-15 | 26.19310202 |
| **AP5M1** | 0.982159 | 2.90124 | 8.533411 | 2.63E-16 | 1.45E-15 | 26.18545026 |
| **SP140L** | 0.697609 | 1.432216 | 8.53293 | 2.64E-16 | 1.46E-15 | 26.18196614 |
| **LAPTM4A** | 1.576125 | 5.682291 | 8.531542 | 2.67E-16 | 1.47E-15 | 26.17190121 |
| **C11orf54** | 0.926646 | 3.331342 | 8.531056 | 2.68E-16 | 1.47E-15 | 26.16837958 |
| **SAR1A** | 1.028577 | 4.560517 | 8.530846 | 2.68E-16 | 1.48E-15 | 26.16686184 |
| **PRDM10** | 0.553889 | 1.281134 | 8.530507 | 2.69E-16 | 1.48E-15 | 26.16440139 |
| **ORMDL2** | 1.081655 | 4.166777 | 8.529933 | 2.70E-16 | 1.49E-15 | 26.16023932 |
| **NEDD4** | 0.69384 | 1.305815 | 8.529527 | 2.71E-16 | 1.49E-15 | 26.15729761 |
| **ERICH1** | 0.857298 | 2.997924 | 8.529259 | 2.72E-16 | 1.49E-15 | 26.15536168 |
| **TRAPPC10** | 0.878374 | 2.721359 | 8.529252 | 2.72E-16 | 1.49E-15 | 26.15530577 |
| **CTC1** | 0.906009 | 3.129667 | 8.528468 | 2.73E-16 | 1.50E-15 | 26.14962392 |
| **RP11-277P12.9** | 0.533561 | 0.476402 | 8.527946 | 2.74E-16 | 1.51E-15 | 26.14584365 |
| **VAT1** | 1.32026 | 5.818293 | 8.527426 | 2.75E-16 | 1.51E-15 | 26.14207966 |
| **RP11-84C13.1** | 0.581183 | 0.990334 | 8.527271 | 2.76E-16 | 1.51E-15 | 26.14095106 |
| **RAB5A** | 1.274414 | 4.612181 | 8.527176 | 2.76E-16 | 1.51E-15 | 26.14026831 |
| **ST6GALNAC4** | 0.921945 | 3.670963 | 8.526756 | 2.77E-16 | 1.52E-15 | 26.13722272 |
| **ATP11A** | 1.040485 | 2.304452 | 8.526222 | 2.78E-16 | 1.52E-15 | 26.13335214 |
| **ORC4** | 1.064059 | 3.468773 | 8.526073 | 2.78E-16 | 1.53E-15 | 26.13227285 |
| **PPFIA1** | 1.172007 | 4.014205 | 8.525851 | 2.78E-16 | 1.53E-15 | 26.13066872 |
| **ADIPOR1** | 1.144908 | 4.350876 | 8.525121 | 2.80E-16 | 1.54E-15 | 26.1253797 |
| **GNG10** | 1.148873 | 4.614029 | 8.524852 | 2.80E-16 | 1.54E-15 | 26.12342794 |
| **SLTM** | 1.071031 | 4.84738 | 8.52459 | 2.81E-16 | 1.54E-15 | 26.12153403 |
| **BRD9** | 0.999562 | 4.415991 | 8.524251 | 2.82E-16 | 1.54E-15 | 26.11907247 |
| **GIMAP2** | 0.851104 | 2.050229 | 8.524077 | 2.82E-16 | 1.55E-15 | 26.11781805 |
| **SMAD1** | 1.475467 | 2.816665 | 8.523456 | 2.83E-16 | 1.55E-15 | 26.11331403 |
| **TMED4** | 1.209261 | 4.868174 | 8.523334 | 2.84E-16 | 1.55E-15 | 26.11243443 |
| **FBXO30** | 0.738774 | 1.70145 | 8.522911 | 2.84E-16 | 1.56E-15 | 26.1093674 |
| **NRBF2** | 0.893728 | 2.601368 | 8.522847 | 2.85E-16 | 1.56E-15 | 26.10890678 |
| **IFRD1** | 1.064454 | 4.19656 | 8.521255 | 2.88E-16 | 1.58E-15 | 26.09738019 |
| **ZNF682** | 0.708709 | 2.264586 | 8.521187 | 2.88E-16 | 1.58E-15 | 26.09688375 |
| **ZNF596** | 0.812082 | 2.011885 | 8.521091 | 2.88E-16 | 1.58E-15 | 26.09618696 |
| **XPO4** | 0.738526 | 1.664404 | 8.520856 | 2.89E-16 | 1.58E-15 | 26.09448834 |
| **GUSBP3** | 0.876843 | 2.274651 | 8.520838 | 2.89E-16 | 1.58E-15 | 26.09436009 |
| **CD164** | 1.262996 | 4.325872 | 8.520676 | 2.89E-16 | 1.58E-15 | 26.09318114 |
| **SEC61G** | 1.811495 | 7.302581 | 8.520617 | 2.89E-16 | 1.58E-15 | 26.09275918 |
| **KIAA0753** | 0.751172 | 2.201009 | 8.519675 | 2.91E-16 | 1.59E-15 | 26.08593433 |
| **MTMR4** | 1.005816 | 3.28773 | 8.518922 | 2.93E-16 | 1.60E-15 | 26.0804824 |
| **ZNF608** | 0.964084 | 3.026796 | 8.518783 | 2.93E-16 | 1.60E-15 | 26.07947738 |
| **PML** | 0.947289 | 3.884652 | 8.51876 | 2.93E-16 | 1.60E-15 | 26.07930808 |
| **TGOLN2** | 1.144946 | 3.980874 | 8.518194 | 2.95E-16 | 1.61E-15 | 26.07521487 |
| **LSM14B** | 1.144111 | 4.473396 | 8.518051 | 2.95E-16 | 1.61E-15 | 26.07417858 |
| **NACC1** | 0.974897 | 4.559738 | 8.517934 | 2.95E-16 | 1.61E-15 | 26.07333069 |
| **ESM1** | 1.055404 | 0.931913 | 8.517511 | 2.96E-16 | 1.62E-15 | 26.07026694 |
| **REXO2** | 1.150498 | 4.281489 | 8.51728 | 2.97E-16 | 1.62E-15 | 26.0685965 |
| **ADPRHL2** | 1.037365 | 4.255386 | 8.517145 | 2.97E-16 | 1.62E-15 | 26.06762029 |
| **TRPC4AP** | 1.034634 | 4.673972 | 8.516457 | 2.98E-16 | 1.63E-15 | 26.06263618 |
| **EIF3H** | 1.310659 | 5.737439 | 8.515944 | 2.99E-16 | 1.63E-15 | 26.05892183 |
| **RP11-440D17.3** | 0.619227 | 1.410195 | 8.515746 | 3.00E-16 | 1.63E-15 | 26.05749413 |
| **GTF2A1** | 0.915263 | 2.458293 | 8.515298 | 3.01E-16 | 1.64E-15 | 26.05424872 |
| **NFAM1** | 0.567395 | 1.234196 | 8.515216 | 3.01E-16 | 1.64E-15 | 26.05365408 |
| **DCAF4** | 0.811469 | 2.594526 | 8.514786 | 3.02E-16 | 1.64E-15 | 26.05054584 |
| **TIMP1** | 1.93282 | 5.615226 | 8.514598 | 3.02E-16 | 1.65E-15 | 26.04918471 |
| **UBE2Z** | 1.040462 | 4.443695 | 8.514206 | 3.03E-16 | 1.65E-15 | 26.04634698 |
| **DENND4C** | 0.867338 | 2.247029 | 8.513831 | 3.04E-16 | 1.65E-15 | 26.04362912 |
| **ZNF70** | 0.533737 | 1.115096 | 8.512189 | 3.08E-16 | 1.67E-15 | 26.03174892 |
| **RBFA** | 0.93267 | 3.795037 | 8.512166 | 3.08E-16 | 1.67E-15 | 26.03158232 |
| **KSR1** | 1.290391 | 3.794177 | 8.512104 | 3.08E-16 | 1.67E-15 | 26.03113529 |
| **IL4I1** | 0.788035 | 1.315558 | 8.511838 | 3.09E-16 | 1.68E-15 | 26.02920872 |
| **URM1** | 1.193282 | 5.680339 | 8.511791 | 3.09E-16 | 1.68E-15 | 26.02886861 |
| **WNK1** | 1.283814 | 4.354931 | 8.511752 | 3.09E-16 | 1.68E-15 | 26.02858903 |
| **CTC-338M12.4** | 0.729805 | 2.378961 | 8.511073 | 3.10E-16 | 1.69E-15 | 26.02367035 |
| **SNX5** | 1.196367 | 5.095841 | 8.509782 | 3.13E-16 | 1.70E-15 | 26.01433357 |
| **STEAP3** | 0.959999 | 1.770061 | 8.509568 | 3.14E-16 | 1.70E-15 | 26.01278208 |
| **COG5** | 1.022143 | 2.690836 | 8.50861 | 3.16E-16 | 1.72E-15 | 26.00585677 |
| **HNRNPC** | 1.508243 | 7.753458 | 8.507885 | 3.18E-16 | 1.72E-15 | 26.00060742 |
| **IL4R** | 0.830304 | 2.447088 | 8.507655 | 3.18E-16 | 1.73E-15 | 25.9989441 |
| **CDH24** | 1.035488 | 3.646361 | 8.506988 | 3.20E-16 | 1.73E-15 | 25.99412233 |
| **CCNT2** | 1.059766 | 2.791065 | 8.506572 | 3.21E-16 | 1.74E-15 | 25.99111295 |
| **SVIL** | 0.725917 | 1.164416 | 8.506408 | 3.21E-16 | 1.74E-15 | 25.98992584 |
| **OGFOD3** | 0.964653 | 4.080109 | 8.506131 | 3.22E-16 | 1.74E-15 | 25.98792263 |
| **MAPKAPK3** | 0.952162 | 3.547547 | 8.505693 | 3.23E-16 | 1.75E-15 | 25.9847568 |
| **EMILIN2** | 1.07576 | 2.157681 | 8.50543 | 3.24E-16 | 1.75E-15 | 25.98285297 |
| **JRK** | 0.776869 | 2.282595 | 8.505045 | 3.24E-16 | 1.76E-15 | 25.98006925 |
| **ARHGAP1** | 1.112391 | 4.560731 | 8.504846 | 3.25E-16 | 1.76E-15 | 25.97862564 |
| **HDAC8** | 0.814995 | 3.419118 | 8.504317 | 3.26E-16 | 1.77E-15 | 25.97480427 |
| **TIAL1** | 1.226979 | 4.986419 | 8.503897 | 3.27E-16 | 1.77E-15 | 25.97176625 |
| **ZNF592** | 0.841938 | 2.948453 | 8.503771 | 3.27E-16 | 1.77E-15 | 25.97085142 |
| **DCTN2** | 1.341804 | 6.154843 | 8.503707 | 3.28E-16 | 1.77E-15 | 25.97039392 |
| **ARHGEF2** | 1.139173 | 5.633043 | 8.502266 | 3.31E-16 | 1.79E-15 | 25.95997481 |
| **MTPN** | 1.109736 | 4.907504 | 8.501453 | 3.33E-16 | 1.80E-15 | 25.95409471 |
| **ZNF182** | 0.615931 | 1.522161 | 8.501094 | 3.34E-16 | 1.81E-15 | 25.95150219 |
| **LRWD1** | 1.103005 | 4.739595 | 8.500069 | 3.37E-16 | 1.82E-15 | 25.9440883 |
| **SAP30** | 1.048501 | 4.06925 | 8.499927 | 3.37E-16 | 1.82E-15 | 25.94305956 |
| **NOD1** | 0.602274 | 1.599154 | 8.499764 | 3.37E-16 | 1.82E-15 | 25.94188698 |
| **CD2AP** | 0.626272 | 1.452102 | 8.499719 | 3.37E-16 | 1.82E-15 | 25.94155791 |
| **ZMYM3** | 0.907831 | 3.206243 | 8.498802 | 3.40E-16 | 1.83E-15 | 25.93493267 |
| **ZNF426** | 0.827496 | 1.277428 | 8.498275 | 3.41E-16 | 1.84E-15 | 25.93111962 |
| **TRAPPC13** | 0.868419 | 3.10136 | 8.497509 | 3.43E-16 | 1.85E-15 | 25.92558346 |
| **LRRC40** | 0.929891 | 2.272068 | 8.496947 | 3.44E-16 | 1.86E-15 | 25.92152588 |
| **EPB41L4A** | 0.590824 | 1.310403 | 8.496868 | 3.45E-16 | 1.86E-15 | 25.92095301 |
| **ARMCX3** | 1.324439 | 4.521563 | 8.496137 | 3.46E-16 | 1.87E-15 | 25.91566725 |
| **LOH12CR1** | 0.7205 | 2.499106 | 8.49564 | 3.48E-16 | 1.87E-15 | 25.91207835 |
| **WDR20** | 0.940701 | 3.198647 | 8.495197 | 3.49E-16 | 1.88E-15 | 25.90887367 |
| **PURB** | 0.85819 | 2.998725 | 8.495005 | 3.49E-16 | 1.88E-15 | 25.90749071 |
| **RNASEH2B** | 0.844645 | 3.91051 | 8.494687 | 3.50E-16 | 1.89E-15 | 25.90519195 |
| **TMEM167B** | 1.050091 | 3.392959 | 8.494526 | 3.51E-16 | 1.89E-15 | 25.90403085 |
| **AVEN** | 0.661278 | 2.554234 | 8.494519 | 3.51E-16 | 1.89E-15 | 25.90398008 |
| **NAMPTL** | 0.553164 | 0.611137 | 8.494249 | 3.51E-16 | 1.89E-15 | 25.90202551 |
| **FCGBP** | 1.157255 | 2.13176 | 8.494144 | 3.51E-16 | 1.89E-15 | 25.90126908 |
| **C8orf31** | 0.667663 | 2.288307 | 8.494072 | 3.52E-16 | 1.89E-15 | 25.90074799 |
| **MCU** | 0.741962 | 2.399876 | 8.493753 | 3.53E-16 | 1.90E-15 | 25.89844497 |
| **CCDC34** | 0.903064 | 2.581429 | 8.493312 | 3.54E-16 | 1.90E-15 | 25.89525676 |
| **SAMD9L** | 0.833557 | 1.407327 | 8.492579 | 3.56E-16 | 1.91E-15 | 25.88996395 |
| **ZNF768** | 0.98138 | 4.54942 | 8.491813 | 3.58E-16 | 1.92E-15 | 25.88442614 |
| **PAN3** | 0.92755 | 2.751846 | 8.491759 | 3.58E-16 | 1.92E-15 | 25.88403955 |
| **HCFC2** | 0.893818 | 2.04748 | 8.491457 | 3.59E-16 | 1.93E-15 | 25.88185631 |
| **OTUD4** | 0.852104 | 2.343051 | 8.491449 | 3.59E-16 | 1.93E-15 | 25.88180018 |
| **YY1AP1** | 0.680201 | 4.839479 | 8.490985 | 3.60E-16 | 1.93E-15 | 25.87844266 |
| **NFKB2** | 0.887229 | 3.031372 | 8.490954 | 3.60E-16 | 1.93E-15 | 25.87821849 |
| **FAM46B** | 0.594797 | 0.712248 | 8.4909 | 3.60E-16 | 1.93E-15 | 25.87782795 |
| **TRIM62** | 0.791358 | 2.345454 | 8.490888 | 3.60E-16 | 1.93E-15 | 25.87774362 |
| **LPAR6** | 1.023804 | 3.147374 | 8.490511 | 3.61E-16 | 1.94E-15 | 25.87501793 |
| **MMADHC** | 1.236783 | 4.251844 | 8.490485 | 3.61E-16 | 1.94E-15 | 25.87483048 |
| **INPP5E** | 0.983432 | 3.357356 | 8.489949 | 3.62E-16 | 1.95E-15 | 25.87096275 |
| **DAB2IP** | 1.069195 | 4.285581 | 8.489768 | 3.63E-16 | 1.95E-15 | 25.86965374 |
| **XRRA1** | 0.626289 | 2.457949 | 8.488812 | 3.66E-16 | 1.96E-15 | 25.86275197 |
| **RIOK2** | 0.846992 | 3.207901 | 8.488572 | 3.66E-16 | 1.96E-15 | 25.86101863 |
| **MRPL32** | 1.178745 | 4.632253 | 8.488376 | 3.67E-16 | 1.97E-15 | 25.85959883 |
| **MSI2** | 1.136568 | 5.043312 | 8.486516 | 3.72E-16 | 1.99E-15 | 25.84616791 |
| **FBXO8** | 0.993851 | 3.042864 | 8.485872 | 3.74E-16 | 2.00E-15 | 25.84152189 |
| **TRA2A** | 1.295432 | 4.739244 | 8.485788 | 3.74E-16 | 2.00E-15 | 25.84091588 |
| **DHX34** | 0.825715 | 3.126555 | 8.485215 | 3.75E-16 | 2.01E-15 | 25.83677727 |
| **TBC1D14** | 1.245574 | 4.615859 | 8.485032 | 3.76E-16 | 2.01E-15 | 25.83545573 |
| **SSH2** | 0.773062 | 2.910548 | 8.48476 | 3.77E-16 | 2.02E-15 | 25.83349014 |
| **RPL17-C18orf32** | 0.762244 | 1.622402 | 8.484328 | 3.78E-16 | 2.02E-15 | 25.83037372 |
| **RP11-73M18.10** | 0.829229 | 2.169233 | 8.484086 | 3.78E-16 | 2.03E-15 | 25.82862189 |
| **CCNL1** | 1.226584 | 4.437255 | 8.48387 | 3.79E-16 | 2.03E-15 | 25.82706574 |
| **IFIT5** | 0.862354 | 2.549194 | 8.483758 | 3.79E-16 | 2.03E-15 | 25.82625661 |
| **NDNL2** | 1.048962 | 3.503595 | 8.482748 | 3.82E-16 | 2.04E-15 | 25.81896332 |
| **GLCE** | 0.74228 | 1.893579 | 8.48248 | 3.83E-16 | 2.05E-15 | 25.8170295 |
| **TSPAN18** | 1.007648 | 1.640394 | 8.48143 | 3.86E-16 | 2.06E-15 | 25.80945079 |
| **FAM203A** | 0.853441 | 3.435052 | 8.481155 | 3.87E-16 | 2.07E-15 | 25.80746627 |
| **PRDM15** | 0.509883 | 1.663275 | 8.480854 | 3.88E-16 | 2.07E-15 | 25.80529339 |
| **TDRKH** | 0.838613 | 2.362506 | 8.480424 | 3.89E-16 | 2.08E-15 | 25.80219296 |
| **CLK3** | 1.042216 | 5.095794 | 8.479546 | 3.91E-16 | 2.09E-15 | 25.79585085 |
| **IGF1R** | 0.778647 | 2.27809 | 8.479067 | 3.93E-16 | 2.10E-15 | 25.792394 |
| **ZNF713** | 0.776547 | 1.723588 | 8.478862 | 3.93E-16 | 2.10E-15 | 25.79092148 |
| **GTPBP1** | 0.887969 | 4.047638 | 8.477845 | 3.96E-16 | 2.11E-15 | 25.78357642 |
| **LEF1** | 0.895878 | 2.442802 | 8.477735 | 3.96E-16 | 2.12E-15 | 25.78278584 |
| **FAM98B** | 0.877968 | 3.06206 | 8.477602 | 3.97E-16 | 2.12E-15 | 25.78182462 |
| **PRTFDC1** | 1.148132 | 4.385884 | 8.477506 | 3.97E-16 | 2.12E-15 | 25.78113 |
| **TRAF6** | 0.65114 | 1.422999 | 8.477116 | 3.98E-16 | 2.12E-15 | 25.77831597 |
| **IGFBP3** | 1.42201 | 3.253892 | 8.476474 | 4.00E-16 | 2.13E-15 | 25.7736895 |
| **TOMM70A** | 0.979942 | 3.635746 | 8.47555 | 4.03E-16 | 2.15E-15 | 25.76702144 |
| **TMF1** | 0.849329 | 1.953531 | 8.475099 | 4.04E-16 | 2.15E-15 | 25.76376688 |
| **AHR** | 0.839682 | 1.408371 | 8.474757 | 4.05E-16 | 2.16E-15 | 25.76130201 |
| **LDOC1L** | 1.104411 | 3.476806 | 8.474689 | 4.05E-16 | 2.16E-15 | 25.76081121 |
| **FSCN1** | 1.371702 | 6.42428 | 8.474588 | 4.06E-16 | 2.16E-15 | 25.76008352 |
| **BCAT1** | 0.932041 | 1.658174 | 8.474026 | 4.07E-16 | 2.17E-15 | 25.75602721 |
| **UBA5** | 1.052452 | 3.87328 | 8.473197 | 4.10E-16 | 2.18E-15 | 25.75004582 |
| **ATXN1L** | 0.926465 | 2.276204 | 8.473163 | 4.10E-16 | 2.18E-15 | 25.74980283 |
| **PEX19** | 1.202113 | 4.493426 | 8.473024 | 4.10E-16 | 2.18E-15 | 25.74880291 |
| **ST7-AS1** | 0.808649 | 2.591615 | 8.472913 | 4.11E-16 | 2.18E-15 | 25.7480005 |
| **PPT1** | 1.303191 | 5.471974 | 8.472102 | 4.13E-16 | 2.20E-15 | 25.74215081 |
| **HEG1** | 1.001561 | 2.485393 | 8.471492 | 4.15E-16 | 2.21E-15 | 25.73775248 |
| **CLCN7** | 1.272007 | 5.699621 | 8.471439 | 4.15E-16 | 2.21E-15 | 25.73736656 |
| **JUNB** | 1.462931 | 5.459387 | 8.471422 | 4.15E-16 | 2.21E-15 | 25.73724341 |
| **PIK3C2B** | 0.894641 | 2.463905 | 8.470944 | 4.17E-16 | 2.21E-15 | 25.73379823 |
| **VPS35** | 1.216974 | 3.956086 | 8.470916 | 4.17E-16 | 2.21E-15 | 25.73359519 |
| **CNIH1** | 1.186246 | 4.382569 | 8.470107 | 4.19E-16 | 2.23E-15 | 25.72776484 |
| **AIF1L** | 1.259168 | 5.251343 | 8.470046 | 4.19E-16 | 2.23E-15 | 25.72732479 |
| **FAHD1** | 0.965093 | 3.824272 | 8.469738 | 4.20E-16 | 2.23E-15 | 25.72510163 |
| **CTSB** | 1.459909 | 7.254128 | 8.469625 | 4.21E-16 | 2.23E-15 | 25.72428487 |
| **SETX** | 0.898768 | 3.312823 | 8.469463 | 4.21E-16 | 2.23E-15 | 25.72311932 |
| **SLC38A6** | 0.714192 | 2.667147 | 8.469162 | 4.22E-16 | 2.24E-15 | 25.7209482 |
| **ZNF611** | 0.675096 | 1.409915 | 8.469025 | 4.23E-16 | 2.24E-15 | 25.71995883 |
| **LRRCC1** | 0.945262 | 2.307081 | 8.468947 | 4.23E-16 | 2.24E-15 | 25.71939982 |
| **IL2RG** | 0.728613 | 1.418957 | 8.468832 | 4.23E-16 | 2.24E-15 | 25.71856708 |
| **POP4** | 1.164764 | 4.758398 | 8.468525 | 4.24E-16 | 2.25E-15 | 25.7163564 |
| **NAA25** | 0.837023 | 2.353441 | 8.467856 | 4.26E-16 | 2.26E-15 | 25.71152927 |
| **ADAMTS15** | 0.858101 | 1.149896 | 8.467777 | 4.27E-16 | 2.26E-15 | 25.71096457 |
| **AP3D1** | 1.106815 | 5.10828 | 8.467602 | 4.27E-16 | 2.26E-15 | 25.70969791 |
| **RP11-342K6.1** | 0.659978 | 2.044133 | 8.466209 | 4.31E-16 | 2.28E-15 | 25.69966113 |
| **IKBKAP** | 1.110314 | 4.127261 | 8.466096 | 4.32E-16 | 2.29E-15 | 25.69884603 |
| **CCDC41** | 0.867574 | 2.327024 | 8.465555 | 4.34E-16 | 2.29E-15 | 25.69494158 |
| **GTF2H1** | 0.951794 | 3.516152 | 8.464149 | 4.38E-16 | 2.32E-15 | 25.68480694 |
| **ANKRD11** | 1.076055 | 4.449026 | 8.463571 | 4.40E-16 | 2.33E-15 | 25.68064445 |
| **ZNF213** | 0.797335 | 3.262697 | 8.463431 | 4.40E-16 | 2.33E-15 | 25.679633 |
| **RPS6KA3** | 0.866873 | 1.900654 | 8.463227 | 4.41E-16 | 2.33E-15 | 25.6781664 |
| **FBXL19-AS1** | 0.52707 | 0.751581 | 8.462988 | 4.42E-16 | 2.34E-15 | 25.67644507 |
| **THUMPD2** | 0.83076 | 2.801821 | 8.462842 | 4.42E-16 | 2.34E-15 | 25.67538776 |
| **SLC25A46** | 1.160329 | 3.280741 | 8.462808 | 4.42E-16 | 2.34E-15 | 25.67514386 |
| **TAX1BP1** | 1.311857 | 4.564559 | 8.461735 | 4.46E-16 | 2.35E-15 | 25.66741116 |
| **HEYL** | 0.828006 | 1.619177 | 8.460971 | 4.48E-16 | 2.37E-15 | 25.66190904 |
| **PTPRE** | 1.040297 | 2.91515 | 8.460557 | 4.50E-16 | 2.37E-15 | 25.65892183 |
| **EIF3EP1** | 0.659113 | 1.404958 | 8.460029 | 4.51E-16 | 2.38E-15 | 25.65512278 |
| **SRSF12** | 0.682208 | 1.958768 | 8.459416 | 4.53E-16 | 2.39E-15 | 25.6507005 |
| **BCL2A1** | 0.855848 | 1.345254 | 8.459343 | 4.54E-16 | 2.39E-15 | 25.65017916 |
| **ZNF148** | 0.705926 | 2.112608 | 8.458948 | 4.55E-16 | 2.40E-15 | 25.64732982 |
| **FBXO45** | 0.747493 | 2.425276 | 8.458728 | 4.56E-16 | 2.40E-15 | 25.64574394 |
| **PPP1R2** | 0.965945 | 3.573157 | 8.458697 | 4.56E-16 | 2.40E-15 | 25.64552689 |
| **LTBR** | 0.942243 | 2.775367 | 8.458419 | 4.57E-16 | 2.41E-15 | 25.64351777 |
| **COA1** | 1.180645 | 4.924383 | 8.458328 | 4.57E-16 | 2.41E-15 | 25.64286588 |
| **MED8** | 0.87376 | 3.59768 | 8.458078 | 4.58E-16 | 2.41E-15 | 25.64106202 |
| **TMEM230** | 1.38683 | 5.183772 | 8.458048 | 4.58E-16 | 2.41E-15 | 25.64084699 |
| **DHTKD1** | 0.948351 | 3.003382 | 8.45799 | 4.58E-16 | 2.41E-15 | 25.64042826 |
| **RP11-427H3.3** | 0.640424 | 1.965107 | 8.457845 | 4.59E-16 | 2.42E-15 | 25.63938381 |
| **SYF2** | 1.164353 | 4.769187 | 8.456987 | 4.62E-16 | 2.43E-15 | 25.63320706 |
| **DUS3L** | 1.040987 | 4.774463 | 8.456852 | 4.62E-16 | 2.43E-15 | 25.63223325 |
| **ARID3B** | 0.54547 | 1.432386 | 8.45657 | 4.63E-16 | 2.44E-15 | 25.63020246 |
| **RHOT1** | 0.962493 | 3.636418 | 8.456004 | 4.65E-16 | 2.45E-15 | 25.62612679 |
| **SEC63** | 1.063731 | 4.277412 | 8.454747 | 4.69E-16 | 2.47E-15 | 25.61707069 |
| **PSMC4** | 1.18905 | 5.567343 | 8.454634 | 4.70E-16 | 2.47E-15 | 25.61625768 |
| **ANP32B** | 1.393449 | 5.769098 | 8.453989 | 4.72E-16 | 2.48E-15 | 25.61161162 |
| **DDX1** | 1.220008 | 4.894053 | 8.452856 | 4.76E-16 | 2.50E-15 | 25.60345291 |
| **EIF2A** | 1.230736 | 4.724773 | 8.452749 | 4.76E-16 | 2.50E-15 | 25.60268173 |
| **XPC** | 1.051792 | 3.623383 | 8.45254 | 4.77E-16 | 2.51E-15 | 25.60117896 |
| **EIF1AD** | 0.828166 | 3.580141 | 8.451524 | 4.80E-16 | 2.52E-15 | 25.59386002 |
| **CETN3** | 1.007675 | 3.500381 | 8.451382 | 4.81E-16 | 2.53E-15 | 25.59284158 |
| **RCN2** | 1.329041 | 4.890482 | 8.451268 | 4.81E-16 | 2.53E-15 | 25.59202342 |
| **PRPF39** | 0.967604 | 3.169838 | 8.451088 | 4.82E-16 | 2.53E-15 | 25.59072475 |
| **IFIT2** | 1.060631 | 2.890035 | 8.450288 | 4.85E-16 | 2.54E-15 | 25.58496635 |
| **UBE4B** | 1.051825 | 3.251788 | 8.450135 | 4.85E-16 | 2.55E-15 | 25.5838616 |
| **SOCS5** | 0.74201 | 2.013343 | 8.449932 | 4.86E-16 | 2.55E-15 | 25.58239993 |
| **FBXL14** | 1.07489 | 2.863314 | 8.449871 | 4.86E-16 | 2.55E-15 | 25.58196098 |
| **GFOD2** | 0.885677 | 3.814798 | 8.449156 | 4.89E-16 | 2.56E-15 | 25.57681676 |
| **MSL3** | 0.968616 | 3.573072 | 8.44885 | 4.90E-16 | 2.57E-15 | 25.57461243 |
| **FYN** | 1.427348 | 7.058316 | 8.448839 | 4.90E-16 | 2.57E-15 | 25.57453515 |
| **VBP1** | 1.279189 | 4.981772 | 8.448657 | 4.91E-16 | 2.57E-15 | 25.57322688 |
| **TXNL1** | 1.161833 | 4.791921 | 8.448608 | 4.91E-16 | 2.57E-15 | 25.57286879 |
| **AC003665.1** | 0.541396 | 1.494689 | 8.448112 | 4.93E-16 | 2.58E-15 | 25.56930301 |
| **NFXL1** | 0.651117 | 1.884201 | 8.448 | 4.93E-16 | 2.58E-15 | 25.56849363 |
| **STAM2** | 0.873744 | 2.380716 | 8.447971 | 4.93E-16 | 2.58E-15 | 25.56828611 |
| **SLC35B1** | 1.159539 | 5.013654 | 8.447633 | 4.94E-16 | 2.59E-15 | 25.56585253 |
| **DCUN1D1** | 0.923691 | 2.558741 | 8.446987 | 4.97E-16 | 2.60E-15 | 25.56120237 |
| **DNASE1** | 0.831979 | 3.234209 | 8.446851 | 4.97E-16 | 2.60E-15 | 25.56022256 |
| **SLC35E2** | 0.840141 | 2.120091 | 8.445405 | 5.02E-16 | 2.63E-15 | 25.54981873 |
| **IRS1** | 0.650326 | 1.423014 | 8.445111 | 5.03E-16 | 2.63E-15 | 25.54770433 |
| **PDGFRB** | 0.95607 | 3.098314 | 8.4444 | 5.06E-16 | 2.65E-15 | 25.54258495 |
| **MCFD2** | 1.091184 | 3.830065 | 8.443867 | 5.08E-16 | 2.66E-15 | 25.53875375 |
| **KRBA1** | 0.783227 | 3.202885 | 8.443575 | 5.09E-16 | 2.66E-15 | 25.5366518 |
| **DENND4B** | 1.084629 | 4.512199 | 8.443446 | 5.10E-16 | 2.66E-15 | 25.5357197 |
| **CHAC2** | 0.606005 | 1.54668 | 8.443092 | 5.11E-16 | 2.67E-15 | 25.53317759 |
| **YWHAQ** | 1.561335 | 6.633063 | 8.441548 | 5.17E-16 | 2.70E-15 | 25.52206751 |
| **CSPG4** | 1.166969 | 3.42516 | 8.440305 | 5.22E-16 | 2.72E-15 | 25.51312928 |
| **ZNF664** | 1.247002 | 4.829912 | 8.440061 | 5.22E-16 | 2.73E-15 | 25.51136847 |
| **PRPSAP1** | 1.068925 | 4.611611 | 8.440046 | 5.22E-16 | 2.73E-15 | 25.51126016 |
| **ZNF302** | 1.193651 | 3.894949 | 8.439913 | 5.23E-16 | 2.73E-15 | 25.51031015 |
| **KDM3B** | 1.220201 | 3.469878 | 8.43931 | 5.25E-16 | 2.74E-15 | 25.5059676 |
| **C17orf51** | 0.760425 | 2.05757 | 8.438916 | 5.27E-16 | 2.75E-15 | 25.50313259 |
| **NCOA4** | 1.27378 | 4.674621 | 8.438213 | 5.30E-16 | 2.76E-15 | 25.49808259 |
| **PAK4** | 1.163324 | 4.063191 | 8.438025 | 5.30E-16 | 2.76E-15 | 25.49672858 |
| **EMR2** | 0.513347 | 0.683923 | 8.437667 | 5.32E-16 | 2.77E-15 | 25.4941555 |
| **RP11-203J24.8** | 0.678339 | 1.304594 | 8.43752 | 5.32E-16 | 2.77E-15 | 25.49309533 |
| **SMUG1** | 0.938913 | 4.435165 | 8.437475 | 5.32E-16 | 2.77E-15 | 25.49277394 |
| **GLIPR2** | 1.261917 | 4.992005 | 8.437445 | 5.33E-16 | 2.77E-15 | 25.49255939 |
| **EIF5** | 1.410728 | 5.419487 | 8.436429 | 5.36E-16 | 2.79E-15 | 25.4852484 |
| **LAG3** | 0.685299 | 1.280918 | 8.436085 | 5.38E-16 | 2.80E-15 | 25.48278003 |
| **SMPD4** | 1.098698 | 4.317635 | 8.436064 | 5.38E-16 | 2.80E-15 | 25.48262805 |
| **GPATCH11** | 0.920481 | 2.73728 | 8.434648 | 5.44E-16 | 2.83E-15 | 25.47244806 |
| **PPIL3** | 1.177125 | 4.48707 | 8.433646 | 5.48E-16 | 2.85E-15 | 25.46524292 |
| **ATMIN** | 0.889796 | 3.23853 | 8.432816 | 5.51E-16 | 2.86E-15 | 25.4592772 |
| **TAMM41** | 0.670958 | 2.75023 | 8.432544 | 5.52E-16 | 2.87E-15 | 25.45731939 |
| **SMG5** | 1.009223 | 4.81802 | 8.432507 | 5.52E-16 | 2.87E-15 | 25.45704977 |
| **TSEN15** | 1.115647 | 4.169177 | 8.431998 | 5.54E-16 | 2.88E-15 | 25.45339773 |
| **ADCY7** | 0.921378 | 2.578151 | 8.431886 | 5.55E-16 | 2.88E-15 | 25.45259179 |
| **NOP9** | 0.764517 | 3.179917 | 8.431497 | 5.56E-16 | 2.89E-15 | 25.44978965 |
| **INPP5K** | 0.991447 | 4.364282 | 8.431471 | 5.56E-16 | 2.89E-15 | 25.44960392 |
| **VWF** | 1.024669 | 3.168409 | 8.431258 | 5.57E-16 | 2.89E-15 | 25.44807799 |
| **LY96** | 1.064739 | 3.198009 | 8.431151 | 5.58E-16 | 2.89E-15 | 25.44730518 |
| **ERCC3** | 1.10165 | 4.339976 | 8.431115 | 5.58E-16 | 2.89E-15 | 25.44705018 |
| **PKMYT1** | 1.345579 | 4.753529 | 8.43109 | 5.58E-16 | 2.89E-15 | 25.44687039 |
| **AUTS2** | 1.112275 | 2.951556 | 8.430454 | 5.60E-16 | 2.91E-15 | 25.44229338 |
| **PPP2CA** | 1.4954 | 5.146078 | 8.43015 | 5.62E-16 | 2.91E-15 | 25.44011122 |
| **HS1BP3** | 0.890903 | 3.772712 | 8.430033 | 5.62E-16 | 2.91E-15 | 25.43927148 |
| **SFXN1** | 1.102123 | 3.885569 | 8.429753 | 5.63E-16 | 2.92E-15 | 25.43726069 |
| **OGG1** | 0.914663 | 3.940183 | 8.429298 | 5.65E-16 | 2.93E-15 | 25.43398803 |
| **TSPAN11** | 1.12597 | 3.353492 | 8.429014 | 5.66E-16 | 2.93E-15 | 25.43194628 |
| **ZC3H12A** | 0.588538 | 1.118371 | 8.428762 | 5.67E-16 | 2.94E-15 | 25.43013386 |
| **PHF16** | 0.756448 | 1.874018 | 8.428594 | 5.68E-16 | 2.94E-15 | 25.42892723 |
| **NCKAP1L** | 0.824751 | 2.047712 | 8.42853 | 5.68E-16 | 2.94E-15 | 25.42846892 |
| **PREB** | 1.003475 | 4.544991 | 8.4281 | 5.70E-16 | 2.95E-15 | 25.42537933 |
| **UBR5** | 1.100721 | 4.266426 | 8.42778 | 5.71E-16 | 2.96E-15 | 25.42307976 |
| **LYSMD3** | 0.780956 | 1.895725 | 8.427774 | 5.72E-16 | 2.96E-15 | 25.42303685 |
| **ABRACL** | 0.966958 | 2.782801 | 8.427661 | 5.72E-16 | 2.96E-15 | 25.42222241 |
| **TPM3P9** | 0.586045 | 1.539013 | 8.427623 | 5.72E-16 | 2.96E-15 | 25.42195396 |
| **ARHGAP39** | 0.934358 | 3.585058 | 8.427596 | 5.72E-16 | 2.96E-15 | 25.42175971 |
| **FKRP** | 0.948479 | 3.212661 | 8.42759 | 5.72E-16 | 2.96E-15 | 25.42171197 |
| **FAM21A** | 0.928062 | 3.447711 | 8.427509 | 5.73E-16 | 2.96E-15 | 25.42112991 |
| **UBA3** | 1.198373 | 4.268337 | 8.426547 | 5.77E-16 | 2.98E-15 | 25.41421856 |
| **PSMF1** | 1.378802 | 5.489583 | 8.426422 | 5.77E-16 | 2.98E-15 | 25.41332097 |
| **SURF6** | 0.965138 | 4.334466 | 8.426409 | 5.77E-16 | 2.98E-15 | 25.41323206 |
| **CLEC2D** | 0.917595 | 2.411556 | 8.426385 | 5.77E-16 | 2.98E-15 | 25.4130587 |
| **PGRMC2** | 1.105191 | 4.188442 | 8.425691 | 5.80E-16 | 2.99E-15 | 25.4080706 |
| **ACP1** | 1.242964 | 5.098842 | 8.425638 | 5.80E-16 | 3.00E-15 | 25.40769238 |
| **CD68** | 1.430516 | 4.511389 | 8.425601 | 5.81E-16 | 3.00E-15 | 25.40742244 |
| **MAP4K5** | 1.231484 | 3.910493 | 8.42559 | 5.81E-16 | 3.00E-15 | 25.40734184 |
| **KIAA1715** | 0.987746 | 2.520339 | 8.42492 | 5.84E-16 | 3.01E-15 | 25.40252975 |
| **BECN1** | 1.160653 | 4.60937 | 8.424616 | 5.85E-16 | 3.02E-15 | 25.40034879 |
| **USO1** | 1.171218 | 3.738524 | 8.424291 | 5.86E-16 | 3.02E-15 | 25.39801315 |
| **ALG5** | 1.012172 | 4.089067 | 8.424024 | 5.87E-16 | 3.03E-15 | 25.39609712 |
| **OLIG2** | 1.542315 | 6.772613 | 8.423807 | 5.88E-16 | 3.03E-15 | 25.39453467 |
| **TMEM161B** | 0.84548 | 2.404839 | 8.423778 | 5.88E-16 | 3.03E-15 | 25.39432638 |
| **CTD-2619J13.14** | 0.746641 | 2.300968 | 8.423321 | 5.90E-16 | 3.04E-15 | 25.39104754 |
| **RCCD1** | 0.902965 | 3.245219 | 8.423046 | 5.92E-16 | 3.05E-15 | 25.38907408 |
| **MAGED2** | 1.258787 | 6.356717 | 8.423034 | 5.92E-16 | 3.05E-15 | 25.38898343 |
| **TATDN3** | 0.841872 | 3.092539 | 8.422675 | 5.93E-16 | 3.05E-15 | 25.38640709 |
| **GPBP1** | 1.128418 | 4.754804 | 8.422616 | 5.93E-16 | 3.05E-15 | 25.38598028 |
| **ZNF30** | 0.565995 | 1.720549 | 8.422566 | 5.94E-16 | 3.05E-15 | 25.38562335 |
| **FNIP2** | 0.844579 | 2.246661 | 8.422331 | 5.95E-16 | 3.06E-15 | 25.38393282 |
| **RP11-357C3.3** | 0.91507 | 3.504946 | 8.422021 | 5.96E-16 | 3.06E-15 | 25.38171032 |
| **TOMM5** | 1.424056 | 5.779526 | 8.421492 | 5.98E-16 | 3.08E-15 | 25.37790616 |
| **FILIP1L** | 0.889932 | 1.60789 | 8.421388 | 5.99E-16 | 3.08E-15 | 25.37716017 |
| **FCER1G** | 1.503017 | 5.085748 | 8.420259 | 6.04E-16 | 3.10E-15 | 25.36905423 |
| **ZCCHC2** | 0.621403 | 1.946412 | 8.419853 | 6.06E-16 | 3.11E-15 | 25.3661381 |
| **SETD5-AS1** | 1.15332 | 4.198006 | 8.419306 | 6.08E-16 | 3.12E-15 | 25.36221031 |
| **MRPS14** | 1.085295 | 4.231805 | 8.419138 | 6.09E-16 | 3.13E-15 | 25.36100778 |
| **PNPT1** | 1.009334 | 2.922919 | 8.419111 | 6.09E-16 | 3.13E-15 | 25.36081411 |
| **DIMT1** | 1.062258 | 3.452397 | 8.418906 | 6.10E-16 | 3.13E-15 | 25.35933742 |
| **B3GALNT2** | 0.888448 | 1.63565 | 8.418509 | 6.12E-16 | 3.14E-15 | 25.35648765 |
| **ANAPC4** | 0.913915 | 3.481768 | 8.418289 | 6.12E-16 | 3.14E-15 | 25.35490996 |
| **C22orf23** | 0.670852 | 1.967382 | 8.418036 | 6.14E-16 | 3.15E-15 | 25.35308987 |
| **INTS2** | 0.759777 | 1.807104 | 8.41767 | 6.15E-16 | 3.16E-15 | 25.35046621 |
| **MRPL45** | 0.967545 | 3.9307 | 8.417628 | 6.15E-16 | 3.16E-15 | 25.35016486 |
| **NLGN3** | 1.196459 | 4.635463 | 8.417001 | 6.18E-16 | 3.17E-15 | 25.34565972 |
| **MLTK** | 0.820371 | 1.689451 | 8.416884 | 6.19E-16 | 3.17E-15 | 25.34482319 |
| **CNNM4** | 0.683937 | 1.855305 | 8.415887 | 6.23E-16 | 3.19E-15 | 25.33766526 |
| **SLC25A44** | 0.89051 | 3.715538 | 8.414817 | 6.28E-16 | 3.22E-15 | 25.32998462 |
| **MRPS23** | 1.213581 | 4.915243 | 8.414716 | 6.29E-16 | 3.22E-15 | 25.32926149 |
| **MEIS1** | 0.830611 | 2.041271 | 8.414438 | 6.30E-16 | 3.23E-15 | 25.32726569 |
| **RP5-935K16.1** | 0.731924 | 2.954157 | 8.413793 | 6.33E-16 | 3.24E-15 | 25.32263366 |
| **CTD-2291D10.4** | 0.535024 | 0.562923 | 8.41332 | 6.35E-16 | 3.25E-15 | 25.31924058 |
| **STK38L** | 1.033504 | 2.741116 | 8.413317 | 6.35E-16 | 3.25E-15 | 25.31921656 |
| **MAMLD1** | 0.87595 | 2.773998 | 8.412882 | 6.37E-16 | 3.26E-15 | 25.31609672 |
| **EIF1AX** | 1.138727 | 4.144408 | 8.411096 | 6.46E-16 | 3.30E-15 | 25.30328085 |
| **PRKACA** | 1.184313 | 4.730783 | 8.410843 | 6.47E-16 | 3.31E-15 | 25.30146389 |
| **ARSK** | 0.535954 | 1.303849 | 8.410483 | 6.48E-16 | 3.31E-15 | 25.29888029 |
| **RNF111** | 0.81089 | 2.656194 | 8.410321 | 6.49E-16 | 3.32E-15 | 25.29772072 |
| **RND3** | 0.983756 | 2.533994 | 8.409427 | 6.53E-16 | 3.34E-15 | 25.29130812 |
| **BMP7** | 1.230723 | 4.530704 | 8.409381 | 6.54E-16 | 3.34E-15 | 25.29097375 |
| **COMMD5** | 1.036172 | 4.66125 | 8.408929 | 6.56E-16 | 3.35E-15 | 25.28773496 |
| **LSM1** | 1.185698 | 4.473064 | 8.408865 | 6.56E-16 | 3.35E-15 | 25.28727221 |
| **RP11-849H4.2** | 0.518862 | 1.419668 | 8.408738 | 6.57E-16 | 3.35E-15 | 25.28636261 |
| **HPS5** | 0.818395 | 2.339568 | 8.407033 | 6.65E-16 | 3.39E-15 | 25.2741324 |
| **SERAC1** | 0.652833 | 1.827061 | 8.405832 | 6.71E-16 | 3.42E-15 | 25.26551923 |
| **PDCD7** | 0.938523 | 3.118132 | 8.405766 | 6.71E-16 | 3.42E-15 | 25.26504417 |
| **SUN1** | 1.188082 | 5.13703 | 8.405202 | 6.74E-16 | 3.44E-15 | 25.26100178 |
| **GOLPH3L** | 0.806752 | 2.525295 | 8.404572 | 6.77E-16 | 3.45E-15 | 25.25647848 |
| **C15orf57** | 0.889625 | 3.267114 | 8.404177 | 6.79E-16 | 3.46E-15 | 25.25364637 |
| **FAM122B** | 1.009653 | 3.269211 | 8.40413 | 6.79E-16 | 3.46E-15 | 25.25331162 |
| **TMEM206** | 1.038239 | 3.640916 | 8.403707 | 6.81E-16 | 3.47E-15 | 25.25027911 |
| **SUPT4H1** | 1.272032 | 5.71238 | 8.403324 | 6.83E-16 | 3.48E-15 | 25.24753056 |
| **RPS2** | 1.803591 | 9.690787 | 8.402135 | 6.89E-16 | 3.51E-15 | 25.23900328 |
| **WDR82** | 1.203781 | 4.235661 | 8.401733 | 6.91E-16 | 3.52E-15 | 25.23612224 |
| **MXRA5** | 0.601647 | 0.580993 | 8.4016 | 6.92E-16 | 3.52E-15 | 25.23517237 |
| **ZNF776** | 0.974305 | 2.134817 | 8.401453 | 6.93E-16 | 3.52E-15 | 25.23411743 |
| **LMCD1** | 0.895588 | 2.890908 | 8.400965 | 6.95E-16 | 3.54E-15 | 25.2306179 |
| **LINC00461** | 1.360257 | 4.818838 | 8.400234 | 6.99E-16 | 3.55E-15 | 25.22537838 |
| **OR7E7P** | 0.508957 | 0.980222 | 8.40007 | 7.00E-16 | 3.56E-15 | 25.22420333 |
| **PDK1** | 0.588433 | 1.475915 | 8.39995 | 7.00E-16 | 3.56E-15 | 25.22333998 |
| **GPR160** | 0.519516 | 1.087791 | 8.399648 | 7.02E-16 | 3.57E-15 | 25.2211771 |
| **CYB5B** | 1.180135 | 4.507557 | 8.399593 | 7.02E-16 | 3.57E-15 | 25.22078229 |
| **MXD1** | 0.63872 | 1.946711 | 8.399575 | 7.02E-16 | 3.57E-15 | 25.22065182 |
| **RPL23AP79** | 0.928229 | 3.628102 | 8.399224 | 7.04E-16 | 3.57E-15 | 25.21813912 |
| **MANF** | 1.260634 | 5.086629 | 8.39914 | 7.04E-16 | 3.57E-15 | 25.21753735 |
| **MTOR** | 0.947922 | 3.38072 | 8.398942 | 7.05E-16 | 3.58E-15 | 25.21611279 |
| **LARP1** | 1.196197 | 4.510456 | 8.39842 | 7.08E-16 | 3.59E-15 | 25.21237665 |
| **ZNF181** | 1.028069 | 2.698048 | 8.398361 | 7.08E-16 | 3.59E-15 | 25.21195329 |
| **LMBR1** | 1.089681 | 3.414806 | 8.398281 | 7.09E-16 | 3.59E-15 | 25.21137494 |
| **IMMT** | 1.026635 | 4.469232 | 8.396443 | 7.18E-16 | 3.64E-15 | 25.19820428 |
| **STK36** | 1.227538 | 4.201371 | 8.39643 | 7.18E-16 | 3.64E-15 | 25.19811293 |
| **ZNF615** | 0.751424 | 1.639988 | 8.396423 | 7.18E-16 | 3.64E-15 | 25.19806305 |
| **FCHSD2** | 1.171209 | 3.960604 | 8.396131 | 7.20E-16 | 3.65E-15 | 25.19597135 |
| **ARHGEF15** | 0.560981 | 1.261624 | 8.396118 | 7.20E-16 | 3.65E-15 | 25.19587166 |
| **SDF4** | 1.207126 | 5.966958 | 8.396112 | 7.20E-16 | 3.65E-15 | 25.19582868 |
| **ID3** | 1.624488 | 6.625964 | 8.395873 | 7.21E-16 | 3.65E-15 | 25.19412265 |
| **APPL1** | 0.947432 | 2.965307 | 8.395447 | 7.24E-16 | 3.66E-15 | 25.19106628 |
| **FAM135A** | 0.868937 | 3.125656 | 8.395344 | 7.24E-16 | 3.67E-15 | 25.19032861 |
| **MRPS18B** | 1.109793 | 4.706222 | 8.394869 | 7.27E-16 | 3.68E-15 | 25.18692601 |
| **FZD6** | 0.675239 | 1.145524 | 8.394545 | 7.28E-16 | 3.68E-15 | 25.18460045 |
| **GUF1** | 0.8621 | 3.381091 | 8.392858 | 7.37E-16 | 3.73E-15 | 25.17251688 |
| **ARCN1** | 1.075774 | 4.6086 | 8.392719 | 7.38E-16 | 3.73E-15 | 25.17152146 |
| **STX12** | 1.169007 | 4.454016 | 8.392669 | 7.38E-16 | 3.73E-15 | 25.17116038 |
| **COX19** | 0.654385 | 2.357408 | 8.392609 | 7.39E-16 | 3.73E-15 | 25.17072952 |
| **DNAJC8** | 1.332648 | 5.428071 | 8.392606 | 7.39E-16 | 3.73E-15 | 25.17071298 |
| **MMGT1** | 0.90519 | 2.892446 | 8.392343 | 7.40E-16 | 3.74E-15 | 25.16882978 |
| **EXOSC10** | 1.131713 | 4.429007 | 8.392311 | 7.40E-16 | 3.74E-15 | 25.16859553 |
| **LEMD3** | 0.851945 | 2.558089 | 8.391835 | 7.43E-16 | 3.75E-15 | 25.165189 |
| **ZMYND8** | 0.89986 | 3.285344 | 8.39166 | 7.44E-16 | 3.76E-15 | 25.1639324 |
| **CBX1** | 1.295799 | 5.226137 | 8.391064 | 7.47E-16 | 3.77E-15 | 25.15966574 |
| **AMOTL1** | 0.786601 | 2.464221 | 8.390734 | 7.49E-16 | 3.78E-15 | 25.15730024 |
| **SLC39A7** | 1.215312 | 5.606282 | 8.390727 | 7.49E-16 | 3.78E-15 | 25.15724948 |
| **C1D** | 1.020514 | 3.855385 | 8.390016 | 7.53E-16 | 3.80E-15 | 25.15215674 |
| **SF3B14** | 1.442879 | 5.931535 | 8.389852 | 7.54E-16 | 3.80E-15 | 25.1509816 |
| **TCOF1** | 0.780835 | 3.177742 | 8.388955 | 7.59E-16 | 3.82E-15 | 25.14455518 |
| **HDAC7** | 0.985127 | 3.87898 | 8.388468 | 7.61E-16 | 3.84E-15 | 25.14106882 |
| **PLA2G15** | 0.828341 | 3.436336 | 8.388015 | 7.64E-16 | 3.85E-15 | 25.13782426 |
| **ATF3** | 1.108603 | 2.689692 | 8.387165 | 7.68E-16 | 3.87E-15 | 25.13174062 |
| **FAM173B** | 0.951121 | 3.144304 | 8.38675 | 7.71E-16 | 3.88E-15 | 25.12876812 |
| **YWHAZ** | 1.539349 | 6.451007 | 8.386325 | 7.73E-16 | 3.89E-15 | 25.12572097 |
| **ZNF34** | 0.681413 | 2.479283 | 8.385574 | 7.77E-16 | 3.91E-15 | 25.12034762 |
| **ZNF211** | 0.898423 | 2.687628 | 8.385441 | 7.78E-16 | 3.92E-15 | 25.11939715 |
| **GFM1** | 1.050464 | 3.373005 | 8.385251 | 7.79E-16 | 3.92E-15 | 25.11803171 |
| **R3HDM4** | 1.090002 | 4.899712 | 8.385078 | 7.80E-16 | 3.93E-15 | 25.11679716 |
| **PRMT3** | 1.028837 | 2.977081 | 8.384994 | 7.81E-16 | 3.93E-15 | 25.11619686 |
| **NNT** | 1.089702 | 3.543061 | 8.384231 | 7.85E-16 | 3.95E-15 | 25.11073437 |
| **PTPN7** | 0.626952 | 0.975909 | 8.38411 | 7.86E-16 | 3.95E-15 | 25.10986451 |
| **CD3EAP** | 0.608323 | 1.371682 | 8.383169 | 7.91E-16 | 3.98E-15 | 25.10312894 |
| **RPL7AP6** | 0.785088 | 2.311875 | 8.382415 | 7.96E-16 | 4.00E-15 | 25.09773377 |
| **TCP1** | 1.297481 | 5.774459 | 8.382285 | 7.96E-16 | 4.00E-15 | 25.09680209 |
| **RAB13** | 1.496606 | 6.076351 | 8.381792 | 7.99E-16 | 4.02E-15 | 25.09327526 |
| **TRAF3IP2-AS1** | 0.903196 | 3.597738 | 8.38146 | 8.01E-16 | 4.02E-15 | 25.09089792 |
| **GGCX** | 0.864177 | 3.768356 | 8.38108 | 8.03E-16 | 4.03E-15 | 25.08817451 |
| **ZNF519** | 0.830539 | 1.880512 | 8.379631 | 8.12E-16 | 4.08E-15 | 25.07780236 |
| **FOXC1** | 0.845753 | 1.830757 | 8.379544 | 8.12E-16 | 4.08E-15 | 25.07718383 |
| **UBXN4** | 1.403821 | 3.796673 | 8.37935 | 8.13E-16 | 4.08E-15 | 25.07579827 |
| **CTTNBP2** | 1.096947 | 3.098382 | 8.378692 | 8.17E-16 | 4.10E-15 | 25.07108524 |
| **TMEM203** | 1.116176 | 4.938159 | 8.378597 | 8.18E-16 | 4.10E-15 | 25.07040784 |
| **C5orf51** | 0.955864 | 2.476267 | 8.377368 | 8.25E-16 | 4.14E-15 | 25.06161193 |
| **CAPZB** | 1.447334 | 6.36324 | 8.377132 | 8.27E-16 | 4.15E-15 | 25.05992291 |
| **NEDD9** | 0.817436 | 1.968886 | 8.376623 | 8.30E-16 | 4.16E-15 | 25.05628018 |
| **CD14** | 1.400355 | 4.855038 | 8.376028 | 8.33E-16 | 4.18E-15 | 25.05202337 |
| **HNRNPUL2-BSCL2** | 1.430813 | 2.247048 | 8.375772 | 8.35E-16 | 4.18E-15 | 25.0501936 |
| **ARPC1B** | 1.342367 | 5.500491 | 8.375612 | 8.36E-16 | 4.19E-15 | 25.04905142 |
| **PSMG1** | 1.032442 | 4.128238 | 8.375289 | 8.38E-16 | 4.20E-15 | 25.04673766 |
| **RGS17** | 0.635023 | 1.259702 | 8.374206 | 8.44E-16 | 4.23E-15 | 25.03898983 |
| **FAM110A** | 0.882842 | 2.931882 | 8.373803 | 8.47E-16 | 4.24E-15 | 25.0361113 |
| **BBS10** | 0.922478 | 2.34812 | 8.373793 | 8.47E-16 | 4.24E-15 | 25.03603879 |
| **PDGFB** | 0.915981 | 2.709789 | 8.373093 | 8.51E-16 | 4.26E-15 | 25.03103347 |
| **LHFPL3** | 1.607607 | 4.318628 | 8.372822 | 8.53E-16 | 4.27E-15 | 25.02909018 |
| **HSPBAP1** | 0.748441 | 2.411498 | 8.372791 | 8.53E-16 | 4.27E-15 | 25.02887338 |
| **ZNF7** | 0.913278 | 3.870188 | 8.371377 | 8.62E-16 | 4.31E-15 | 25.01875951 |
| **BLZF1** | 0.81256 | 2.500279 | 8.370666 | 8.66E-16 | 4.33E-15 | 25.01367407 |
| **PBX1** | 1.149767 | 3.718733 | 8.370502 | 8.68E-16 | 4.34E-15 | 25.01249878 |
| **FAM103A1** | 0.924821 | 3.213891 | 8.370013 | 8.71E-16 | 4.35E-15 | 25.00900606 |
| **CROT** | 0.923628 | 2.781524 | 8.369679 | 8.73E-16 | 4.36E-15 | 25.00661746 |
| **ZNF529** | 0.791582 | 2.717328 | 8.368788 | 8.78E-16 | 4.39E-15 | 25.00024291 |
| **PSMG2** | 1.240208 | 4.897413 | 8.368624 | 8.79E-16 | 4.39E-15 | 24.99907452 |
| **SYNC** | 0.763372 | 1.734075 | 8.368619 | 8.79E-16 | 4.39E-15 | 24.99903431 |
| **VPS29** | 1.363949 | 5.437157 | 8.366765 | 8.91E-16 | 4.45E-15 | 24.98578578 |
| **NEU3** | 0.69292 | 1.284615 | 8.366759 | 8.91E-16 | 4.45E-15 | 24.98574295 |
| **TWIST1** | 0.982046 | 1.844748 | 8.366234 | 8.95E-16 | 4.47E-15 | 24.98198746 |
| **ARL1** | 1.118506 | 3.757212 | 8.36596 | 8.97E-16 | 4.48E-15 | 24.98002513 |
| **MRPL10** | 1.114108 | 4.428401 | 8.36562 | 8.99E-16 | 4.49E-15 | 24.97759681 |
| **WDTC1** | 0.895212 | 3.499578 | 8.36547 | 9.00E-16 | 4.49E-15 | 24.97652387 |
| **SLFN5** | 0.62665 | 1.120225 | 8.365279 | 9.01E-16 | 4.49E-15 | 24.97515787 |
| **MTERFD2** | 1.016118 | 3.950875 | 8.364858 | 9.04E-16 | 4.51E-15 | 24.97215308 |
| **COL5A1** | 1.032066 | 1.48949 | 8.364667 | 9.05E-16 | 4.51E-15 | 24.97078435 |
| **ERCC6** | 0.506635 | 1.201433 | 8.364153 | 9.08E-16 | 4.53E-15 | 24.9671129 |
| **MAPK8** | 0.993976 | 3.222226 | 8.363883 | 9.10E-16 | 4.54E-15 | 24.96517981 |
| **SEMA6A** | 1.199885 | 3.769237 | 8.363874 | 9.10E-16 | 4.54E-15 | 24.96511548 |
| **SLC1A5** | 0.932282 | 2.345436 | 8.363864 | 9.10E-16 | 4.54E-15 | 24.96504979 |
| **DLL1** | 1.386369 | 4.245202 | 8.36314 | 9.15E-16 | 4.56E-15 | 24.95987096 |
| **PRMT5** | 1.075397 | 4.806005 | 8.363095 | 9.16E-16 | 4.56E-15 | 24.95954964 |
| **KXD1** | 1.162778 | 5.689959 | 8.362543 | 9.19E-16 | 4.58E-15 | 24.95560588 |
| **HOXA10** | 0.775308 | 0.547506 | 8.362394 | 9.20E-16 | 4.58E-15 | 24.95454251 |
| **AKR1B1** | 1.318808 | 6.201883 | 8.362261 | 9.21E-16 | 4.58E-15 | 24.95359397 |
| **PROX1** | 0.737497 | 1.452406 | 8.362108 | 9.22E-16 | 4.59E-15 | 24.95249611 |
| **LHX9** | 0.541744 | 0.462961 | 8.359684 | 9.38E-16 | 4.67E-15 | 24.93517987 |
| **STAG3L1** | 1.07096 | 3.493718 | 8.359466 | 9.40E-16 | 4.68E-15 | 24.93362263 |
| **GSTK1** | 1.276423 | 5.63374 | 8.358102 | 9.49E-16 | 4.72E-15 | 24.92387788 |
| **HS3ST1** | 0.811791 | 1.448347 | 8.358083 | 9.49E-16 | 4.72E-15 | 24.92374398 |
| **ACTR8** | 0.808652 | 3.406779 | 8.357505 | 9.53E-16 | 4.74E-15 | 24.91961014 |
| **LMO2** | 1.298221 | 4.789934 | 8.357473 | 9.54E-16 | 4.74E-15 | 24.91938436 |
| **PTPRJ** | 0.929571 | 1.674219 | 8.357464 | 9.54E-16 | 4.74E-15 | 24.91931927 |
| **TNIP1** | 1.019363 | 4.601845 | 8.357267 | 9.55E-16 | 4.74E-15 | 24.91791158 |
| **RCBTB2** | 0.951933 | 3.189469 | 8.355863 | 9.65E-16 | 4.79E-15 | 24.9078839 |
| **ZNF33A** | 0.733179 | 2.273659 | 8.35477 | 9.73E-16 | 4.83E-15 | 24.90007985 |
| **DPF2** | 1.054651 | 4.77367 | 8.354563 | 9.74E-16 | 4.84E-15 | 24.89860047 |
| **LRRC47** | 1.064674 | 4.745326 | 8.353963 | 9.78E-16 | 4.86E-15 | 24.8943165 |
| **ZYX** | 1.381744 | 5.967149 | 8.35353 | 9.81E-16 | 4.87E-15 | 24.89122773 |
| **TCF20** | 0.823403 | 2.469553 | 8.35306 | 9.85E-16 | 4.88E-15 | 24.88786572 |
| **WDR48** | 1.078703 | 3.543052 | 8.352245 | 9.91E-16 | 4.91E-15 | 24.8820482 |
| **SLMO2** | 1.122596 | 3.642735 | 8.352116 | 9.91E-16 | 4.92E-15 | 24.88113103 |
| **LRP10** | 1.12898 | 4.031678 | 8.351897 | 9.93E-16 | 4.92E-15 | 24.87956695 |
| **TMEM241** | 0.590034 | 1.679896 | 8.351409 | 9.97E-16 | 4.94E-15 | 24.87608433 |
| **PRKCH** | 0.666817 | 1.756342 | 8.351373 | 9.97E-16 | 4.94E-15 | 24.87582219 |
| **CCDC120** | 0.774429 | 2.360404 | 8.351278 | 9.98E-16 | 4.94E-15 | 24.87514664 |
| **TBCC** | 0.934917 | 3.576282 | 8.349491 | 1.01E-15 | 5.01E-15 | 24.86239276 |
| **NNT-AS1** | 0.928542 | 3.110744 | 8.348815 | 1.02E-15 | 5.03E-15 | 24.85756147 |
| **OXSM** | 0.750948 | 2.755532 | 8.348682 | 1.02E-15 | 5.03E-15 | 24.85661601 |
| **NUDT4** | 1.184783 | 4.910365 | 8.347259 | 1.03E-15 | 5.08E-15 | 24.84645698 |
| **SNRPF** | 1.192198 | 4.773214 | 8.346732 | 1.03E-15 | 5.10E-15 | 24.84269852 |
| **RHOA** | 1.581393 | 6.836698 | 8.346044 | 1.04E-15 | 5.13E-15 | 24.83778484 |
| **NUFIP1** | 0.668112 | 1.542064 | 8.345227 | 1.04E-15 | 5.16E-15 | 24.83195601 |
| **MORF4L2** | 1.585597 | 5.882368 | 8.344895 | 1.04E-15 | 5.17E-15 | 24.82959256 |
| **BCL7A** | 1.112031 | 3.223941 | 8.344582 | 1.05E-15 | 5.18E-15 | 24.82735265 |
| **GNA12** | 1.293161 | 4.878758 | 8.344487 | 1.05E-15 | 5.18E-15 | 24.82668065 |
| **SLC25A25** | 1.040338 | 3.314579 | 8.344342 | 1.05E-15 | 5.19E-15 | 24.82564313 |
| **PTCH1** | 1.096534 | 2.906316 | 8.344264 | 1.05E-15 | 5.19E-15 | 24.82508486 |
| **CEP68** | 0.851834 | 2.763924 | 8.343629 | 1.05E-15 | 5.21E-15 | 24.82055691 |
| **RAPH1** | 0.664112 | 1.346627 | 8.342272 | 1.06E-15 | 5.26E-15 | 24.81087791 |
| **RP11-345J4.8** | 0.525056 | 0.906422 | 8.342256 | 1.07E-15 | 5.26E-15 | 24.8107651 |
| **FERMT2** | 1.179309 | 4.124599 | 8.342219 | 1.07E-15 | 5.26E-15 | 24.81049502 |
| **MYO19** | 0.918552 | 3.25836 | 8.341677 | 1.07E-15 | 5.28E-15 | 24.80662889 |
| **UNC5B** | 1.039623 | 2.876991 | 8.341518 | 1.07E-15 | 5.29E-15 | 24.80549452 |
| **PINX1** | 0.780005 | 3.245096 | 8.341412 | 1.07E-15 | 5.29E-15 | 24.80474358 |
| **C8orf59** | 1.202457 | 4.87483 | 8.340865 | 1.08E-15 | 5.31E-15 | 24.80083599 |
| **ATAT1** | 1.192654 | 4.891464 | 8.339641 | 1.09E-15 | 5.35E-15 | 24.79210683 |
| **DMTF1** | 1.259644 | 4.498674 | 8.339432 | 1.09E-15 | 5.36E-15 | 24.79061787 |
| **SNX11** | 0.790091 | 3.494259 | 8.339006 | 1.09E-15 | 5.38E-15 | 24.78757892 |
| **C16orf58** | 1.151469 | 5.080855 | 8.337493 | 1.10E-15 | 5.43E-15 | 24.77679013 |
| **IRX1** | 1.357347 | 2.225386 | 8.337389 | 1.10E-15 | 5.44E-15 | 24.77605084 |
| **STAT2** | 1.180947 | 4.844164 | 8.336992 | 1.11E-15 | 5.45E-15 | 24.77321652 |
| **RANBP9** | 1.031697 | 3.687021 | 8.336929 | 1.11E-15 | 5.45E-15 | 24.77276772 |
| **ZNF641** | 0.806581 | 2.262357 | 8.336814 | 1.11E-15 | 5.46E-15 | 24.77195146 |
| **EIF2B3** | 0.887233 | 3.639332 | 8.336751 | 1.11E-15 | 5.46E-15 | 24.7714974 |
| **VPS26B** | 1.129206 | 4.920331 | 8.336597 | 1.11E-15 | 5.46E-15 | 24.77040006 |
| **PSEN2** | 0.782615 | 3.183316 | 8.336563 | 1.11E-15 | 5.46E-15 | 24.77016327 |
| **PTMS** | 1.716369 | 8.776335 | 8.336069 | 1.11E-15 | 5.48E-15 | 24.76663668 |
| **NBPF11** | 0.90416 | 2.050549 | 8.33585 | 1.12E-15 | 5.49E-15 | 24.76507537 |
| **UCHL5** | 1.034405 | 3.108035 | 8.335709 | 1.12E-15 | 5.49E-15 | 24.76407224 |
| **DBNL** | 1.086448 | 5.803812 | 8.335055 | 1.12E-15 | 5.52E-15 | 24.7594059 |
| **MGRN1** | 1.101979 | 4.774278 | 8.334995 | 1.12E-15 | 5.52E-15 | 24.75897814 |
| **LDHA** | 1.504973 | 5.504555 | 8.334938 | 1.12E-15 | 5.52E-15 | 24.75857457 |
| **NME2** | 1.201905 | 4.251655 | 8.334456 | 1.13E-15 | 5.54E-15 | 24.75513472 |
| **GLT8D1** | 1.193533 | 4.795381 | 8.334057 | 1.13E-15 | 5.55E-15 | 24.75229733 |
| **TERF1** | 1.025439 | 3.454137 | 8.33278 | 1.14E-15 | 5.60E-15 | 24.74319162 |
| **TTC3** | 1.266023 | 5.162521 | 8.332767 | 1.14E-15 | 5.60E-15 | 24.7430995 |
| **RPL15P3** | 0.784103 | 1.807725 | 8.331879 | 1.15E-15 | 5.64E-15 | 24.73676748 |
| **TSPAN6** | 1.160579 | 3.949124 | 8.331239 | 1.15E-15 | 5.66E-15 | 24.73220951 |
| **NOL6** | 0.8456 | 3.650075 | 8.330531 | 1.16E-15 | 5.69E-15 | 24.72715998 |
| **TCEA1** | 1.280499 | 4.488019 | 8.329738 | 1.17E-15 | 5.72E-15 | 24.72151464 |
| **TRAPPC11** | 0.873685 | 3.109335 | 8.329623 | 1.17E-15 | 5.73E-15 | 24.72068822 |
| **FMNL3** | 0.871248 | 3.201982 | 8.329501 | 1.17E-15 | 5.73E-15 | 24.7198248 |
| **GNB1** | 1.443883 | 6.34316 | 8.329168 | 1.17E-15 | 5.74E-15 | 24.71744727 |
| **AP1M1** | 1.0692 | 4.857937 | 8.32841 | 1.18E-15 | 5.77E-15 | 24.71205128 |
| **NREP** | 1.358073 | 5.187628 | 8.328337 | 1.18E-15 | 5.77E-15 | 24.71152637 |
| **SEL1L** | 1.066994 | 3.06614 | 8.328177 | 1.18E-15 | 5.78E-15 | 24.71039176 |
| **SLC7A1** | 1.09746 | 3.4845 | 8.327998 | 1.18E-15 | 5.79E-15 | 24.70911421 |
| **ARL10** | 0.785892 | 2.763478 | 8.327902 | 1.18E-15 | 5.79E-15 | 24.70843224 |
| **IRX5** | 0.674577 | 0.60557 | 8.326856 | 1.19E-15 | 5.83E-15 | 24.70097599 |
| **HOOK3** | 0.825296 | 2.454781 | 8.326523 | 1.19E-15 | 5.84E-15 | 24.69860649 |
| **C6orf89** | 0.990316 | 4.021729 | 8.326488 | 1.19E-15 | 5.85E-15 | 24.69835615 |
| **JUP** | 0.875858 | 2.748804 | 8.326288 | 1.20E-15 | 5.85E-15 | 24.69693084 |
| **FREM2** | 0.569744 | 0.588957 | 8.326158 | 1.20E-15 | 5.86E-15 | 24.69600142 |
| **EFCAB7** | 0.777122 | 2.13926 | 8.325937 | 1.20E-15 | 5.86E-15 | 24.69443035 |
| **GLIPR1** | 0.851923 | 1.89988 | 8.325925 | 1.20E-15 | 5.86E-15 | 24.69434403 |
| **KPNA6** | 0.854462 | 2.778973 | 8.325704 | 1.20E-15 | 5.87E-15 | 24.69276757 |
| **ENTHD2** | 1.051585 | 4.274389 | 8.325681 | 1.20E-15 | 5.87E-15 | 24.69260877 |
| **TAB3** | 0.736062 | 1.820568 | 8.325341 | 1.20E-15 | 5.89E-15 | 24.69018694 |
| **ASAP2** | 0.985381 | 1.867875 | 8.325315 | 1.20E-15 | 5.89E-15 | 24.68999894 |
| **MRPL9** | 1.185942 | 5.127894 | 8.325229 | 1.20E-15 | 5.89E-15 | 24.68938682 |
| **LTA4H** | 0.99613 | 4.214661 | 8.324446 | 1.21E-15 | 5.92E-15 | 24.68380838 |
| **POGZ** | 1.159472 | 4.043016 | 8.323954 | 1.22E-15 | 5.94E-15 | 24.68030506 |
| **AGPAT1** | 1.206913 | 5.455056 | 8.323904 | 1.22E-15 | 5.94E-15 | 24.67994848 |
| **RBM34** | 1.314968 | 5.700006 | 8.323145 | 1.22E-15 | 5.97E-15 | 24.67454 |
| **DDX59** | 0.93014 | 3.254097 | 8.322463 | 1.23E-15 | 6.00E-15 | 24.66968563 |
| **ZNF717** | 0.591429 | 1.541835 | 8.322433 | 1.23E-15 | 6.00E-15 | 24.66947409 |
| **PLA2G12A** | 0.942139 | 2.837746 | 8.322059 | 1.23E-15 | 6.02E-15 | 24.66680621 |
| **SEC23B** | 1.128676 | 4.08754 | 8.321842 | 1.23E-15 | 6.02E-15 | 24.66526247 |
| **RP11-418J17.1** | 0.721107 | 2.518634 | 8.321185 | 1.24E-15 | 6.05E-15 | 24.6605864 |
| **NEU1** | 0.95019 | 4.067891 | 8.321153 | 1.24E-15 | 6.05E-15 | 24.66035671 |
| **STRA6** | 0.686118 | 1.126137 | 8.320515 | 1.25E-15 | 6.08E-15 | 24.65581616 |
| **ZNF783** | 0.876088 | 3.097068 | 8.320463 | 1.25E-15 | 6.08E-15 | 24.65544508 |
| **UEVLD** | 0.887024 | 2.397683 | 8.320338 | 1.25E-15 | 6.08E-15 | 24.65455083 |
| **TAPSAR1** | 0.912798 | 2.879296 | 8.320106 | 1.25E-15 | 6.09E-15 | 24.65290445 |
| **GPATCH8** | 1.00464 | 2.881942 | 8.320098 | 1.25E-15 | 6.09E-15 | 24.6528454 |
| **RGMB** | 1.252102 | 3.771991 | 8.318961 | 1.26E-15 | 6.14E-15 | 24.64475338 |
| **ATP1B3** | 1.277285 | 5.319533 | 8.318594 | 1.26E-15 | 6.16E-15 | 24.64213797 |
| **AMACR** | 0.853079 | 2.647955 | 8.318297 | 1.27E-15 | 6.17E-15 | 24.64002124 |
| **ALG12** | 0.912399 | 2.502363 | 8.317802 | 1.27E-15 | 6.19E-15 | 24.63650116 |
| **SPAG9** | 1.382921 | 5.022412 | 8.316021 | 1.29E-15 | 6.27E-15 | 24.6238247 |
| **FOXJ3** | 0.912273 | 3.079285 | 8.315858 | 1.29E-15 | 6.27E-15 | 24.62266079 |
| **LIPA** | 1.285154 | 4.580448 | 8.315603 | 1.29E-15 | 6.28E-15 | 24.62084327 |
[truncated: 629,769 more chars]
